# Major oscillations in spontaneous home-cage activity in C57BL/6 mice housed under constant conditions

**DOI:** 10.1038/s41598-021-84141-9

**Published:** 2021-03-02

**Authors:** Karin Pernold, Eric Rullman, Brun Ulfhake

**Affiliations:** Division Clinical Physiology, Department of Laboratory Medicine, Karolinska Institutet, Solna, Sweden

**Keywords:** Neuroscience, Physiology

## Abstract

The mouse is the most important mammalian model in life science research and the behavior of the mouse is a key read-out of experimental interventions and genetic manipulations. To serve this purpose a solid understanding of the mouse normal behavior is a prerequisite. Using 14–19 months of cumulative 24/7 home-cage activity recorded with a non-intrusive technique, evidence is here provided for a highly significant circannual oscillation in spontaneous activity (1–2 SD of the mean, on average 65% higher during peak of highs than lows; P = 7E−50) of male and female C57BL/6 mice held under constant conditions. The periodicity of this hitherto not recognized oscillation is in the range of 2–4 months (average estimate was 97 days across cohorts of cages). It off-sets responses to environmental stimuli and co-varies with the feeding behavior but does not significantly alter the preference for being active during the dark hours. The absence of coordination of this rhythmicity between cages with mice or seasons of the year suggest that the oscillation of physical activity is generated by a free-running intrinsic oscillator devoid of external timer. Due to the magnitude of this rhythmic variation it may be a serious confounder in experiments on mice if left unrecognized.

## Introduction

Strains originating from the house mouse are currently the prevailing mammalian models in life science research. The spontaneous behavior and behavioral responses to stimuli are key components in the assessment of experimental interventions and genetic manipulations. The validity of this read-out rests on the assumption that we have a solid understanding of the normal behavior of the mouse^1–8^. The behavior of organisms show distinct rhythmicities governed by internal oscillators which synchronizes (entrain) to external timers (zeitgebers) such as light^9–12^. Most well characterized is the circadian rhythm (reviewed in^13,14^) but gravitational (tidal earth push and pull), seasonal, biannual and annual rhythms have also been described^15–20^. In brief, the free-running circadian rhythm (21–27 h) is generated endogenously by feedback loops involving key clock genes expressed by neurons in the Suprachiasmatic nucleus (SCN) of the hypothalamus that synchronizes with the earth’s solar day (24 h). The entrainment of the circadian rhythm to a day occurs in mammals through retinal neurons directly responding to photic stimuli, or indirectly through retinal cones and rods, and is transmitted to several brain regions, importantly the SCN and, further, to the epithalamus. Here cells of the pineal body translate and feedback the message by suppressing the conversion of serotonin (an amine that also serve as a major neurotransmitter in the nervous system) to melatonin^21,22^ which is secreted during the dark hours only^17,23–26^. A number of biorhythms have a higher frequency than the circadian and are collectively referred to as supraradian biorhythms. While some high-frequency rhythms are well established like the sleep–wake cycle and the different recurring sleep states, most supraradian rhythmicities appears to have an unknown origin. At the other end of the spectrum we have the slow biorhythms (> 1 day), referred to as infraradian (tidal, lunar, seasonal, annual and bi-annual). Typical examples are the reproductive cycle, hibernation and seasonal migration to name a few. Zucker^9^ categorized slow biorhythms into being endogenous rhythms that synchronizes to an external timer (zeitgeber) (type I), rhythms that are purely driven by an endogenous generator (type II), and those that are exclusively driven by re-occurring environmental changes interacting with the geno- and phenotype of an organism such as seasonal allergic complications or the stress and behavioral reaction of small rodent responding to regular husbandry routines like the cage-change at an animal facility (type III).

Laboratory mice used in experiments are commonly bred and maintained under well-controlled and standardized barrier conditions. While husbandry routines provide light (dark and light period of the day; DL) to which the circadian rhythm entrains, these mice are not purposely subject to seasonal or annual variations in the environment. Albeit a few studies have presented data suggesting that both exploring and pain-related behaviors in small laboratory rodents show seasonal variations^27,28^, there is a prevailing skepticism towards the existence of seasonal/circannual behaviors in laboratory mice housed under constant conditions^29^. This notion is building on the successful migration of the house mouse to almost every corner of the world attributed to the mouse capacity to adapt and reproduce in different environments across seasons^30,31^. In line with this both the standardized care and use of laboratory mice commonly consider strain, age (seasons of the life-span), sex and the circadian rhythm as significant parameters but not seasonal (circannual) variations^30,32^.

The aspiration to obtain cumulative records of laboratory animal behaviors in a “non-intrusive home-cage like style” dates back more than a century (for an historical re-collection see^33^) but was until recently restricted to a limited set of tools like the running wheel integrated in the home cage (served critically for the understanding of the circadian rhythm in small rodents). Over the past few years a number of techniques^3,7,34–46^ have become available for automated non-intrusive 24/7 monitoring of in-cage activity^47^ of single housed^7,35,40,44^ and group housed^8,34,36,37,43,45,48,49^ small rodents. Recently we analyzed data of cage floor activity of group-held mice using a DVC system to characterize daily rhythms of spontaneous in-cage activity and impact by husbandry routines^8^. In this study we noted a significant variation in activity across weeks during the 3 months of cumulative records at the three participating sites suggesting the presence of a slow undulation in base-line physical activity^8^. With the time window of three months it was, however, not possible to resolve any pattern of this oscillation (*idem*). Using the same DVC platform (Supportive information Fig. S1) we here report recordings from 14 cages with male (n = 52 in 11 cages) and female (n = 12 in 3 cages) C57BL/6 mice starting at age 7–12 weeks until ~ 16 months of age (n = 11) and in three of these cages up to age ~ 700 days (see Methods) that were then available and therefore stayed in the DVC for an extended period with the purpose to reveal alteration of spontaneous in-cage activity and the pattern of such oscillations over time.

## Results

**Figure 1:**
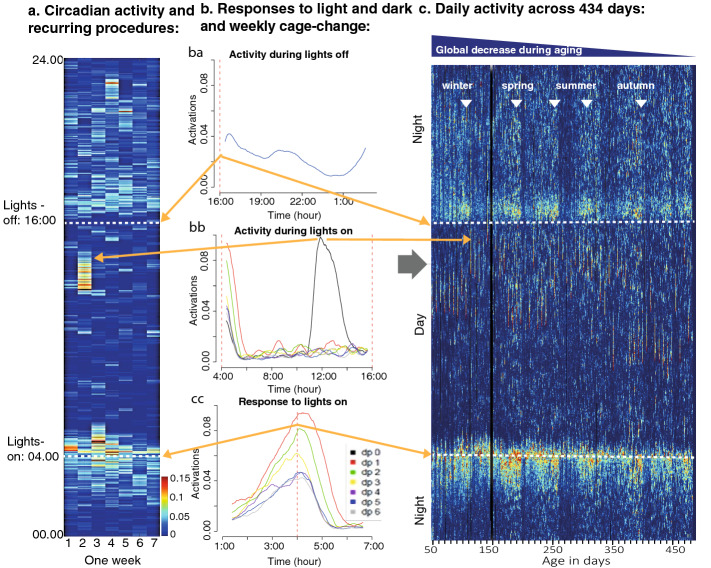
(**a**) is a heat map with seven columns corresponding to the days of one calendar week (day 1 to 7) with color coded activations (for conversion see scale to the right) illustrating the daily rhythmicity in activity synchronized to lights-on (4 AM, white stippled line in **a**,**c**) and lights-off (4 PM, white stippled line in a and c) in one cage (A04) with 5 male mice. (**b**) Details of the response to lights-on is shown for each weekday in **bc** (separate color coded trace for each week day, with dp0 being the day of the cage); followed in **bb** by the day-time resting pattern (middle graph; each day is color coded as in **bc**). The cage is changed once a week (day 2 in (**a**) and dp0 in graph) which instigates a dramatic increase in activity with a carry-over impact on the next following days (see **bc**). (**ba**) shows the response to lights off and night time activity and is the average across weeks. In (**c**) the whole data-set of activations across the 434 days (see Table 1) is shown for the same cage (A04). The ordinate is the same as in (**a**) while the abscissa is animal age in days (49 to 483 days of age). The elements of the circadian rhythm across day and night in (**a**) is also evident in (**c**) (orange arrows to aid navigation). There is an over-all decrease in activity, albeit small yet statistically highly significant (− 4% for this cage; P = 2E−10; see Supportive information Table S1). Inspections of the long term records of activations (**c**) revealed a marked slow oscillation of peaks (highs; indicated by filled inverted triangles) with intervening lows.

**Figure 2:**
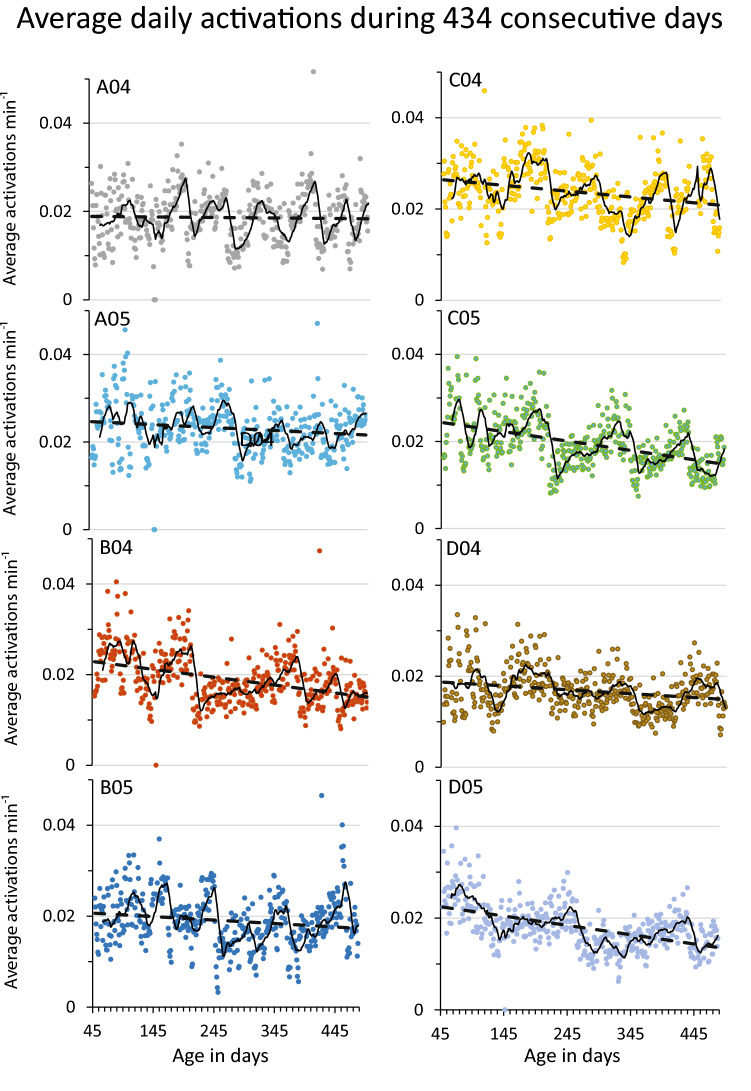
Plot of daily average activations min^−1^ for each of the cumulative data sets in Group 1; A04 depicted also in Fig. 1 is the top left panel. Cage ID is in each panel in the top left corner. The overall activity decreases with age as indicated by the interrupted black line (for linear regression details see Supportive information Table S1). The continuous black line of each panel represents the moving average using a window of one week (= cage-change cycle). As evident from the distribution of data points in each panel and the moving average trace, highs and lows are present in all cages but the dates for highs and lows are not synchronized across cages (for cage A04 the inverted triangles in Fig. 1c are the first five peaks). Ordinate is average daily activation min^−1^. Abscissa at the bottom of each column is valid for all its panels and shows the age of the animals.

The DVC output record (average number of activations across all 12 electrodes min^-1^) of each cage was first plotted as a heat map with full resolution (Fig. 1a,c). Inspection of these heat maps revealed the circadian rhythmicity in activity synchronized to lights on (Fig. 1b,c) and lights off (Fig. 1b,a). In addition, the response to the weekly cage-change was readily identified (Fig. 1b,b)^8^. Closer inspection revealed a significant decline in over-all activity over time (Figs. 1c and 2; see also Fig. 4a–c). On average, the daily activity changed by − 18% (group 3), − 22% (group 1) and − 28% (group 2), respectively, from age 70 to age 470 days. As evident from the slopes of the linear regression (Supportive information, Table S1) of the cumulative records of activity for each cage, there was a large cage-to-cage variability in the age-associated decline in activity (from < − 1% to − 43%) without any corresponding clinical sign of compromised health or welfare among the animals.

An even more conspicuous feature of the heat maps (Fig. 1c) and activation plots (see Supportive information Fig. S3a-n) is the recurring periods with higher over-all in-cage activity (‘highs’; triangles in Fig. 1c) intervened by periods with lower over-all activity (‘lows’). This pattern of oscillation of in-cage activity was present in all cages examined (*idem*) and Fig. 2 depicts the eight cages of group 1 showing the magnitude of the activity-shift between highs and subsequent lows (for plots of data with minute resolution of all 14 cages please see Supportive information Figs S3a-n). On average, the difference in activity between the peak of a high and the subsequent low was 1.6 standard deviations of the mean activity, with a variance between oscillations of ± 0.61 standard deviations. Both the heat maps and activation plots of in-cage activity suggested that activity cluster at different frequencies possible to resolve by spectral analysis and functional analysis.

**Figure 3:**
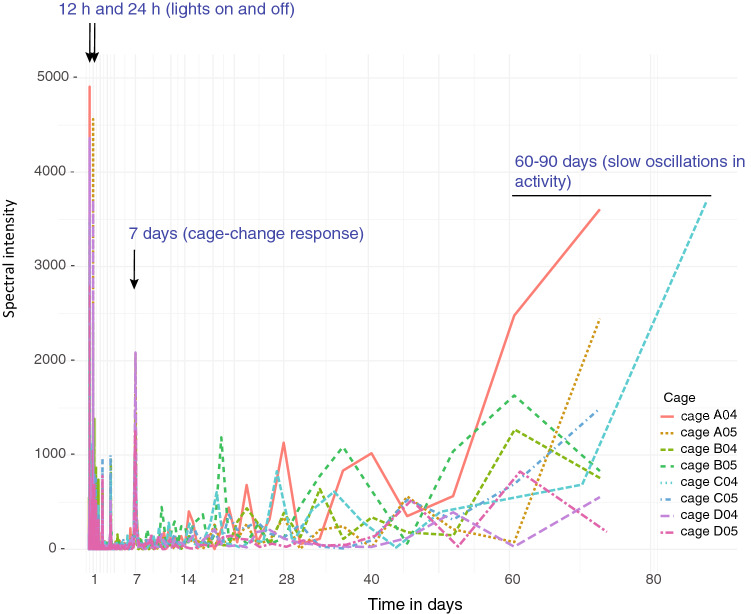
Superimposed spectrograms of activations clustering at different frequencies of eight cages (color coded; key is to the right in the spectrogram) with male mice in group 1 (see Methods). Note the precise timing across cages of the behaviors triggered by environmental stimuli, while the slow oscillations having a periodicity in the range of 60–90 days are not synchronized across cages**.** The slow peaks are generated by only 3–6 instances in the cumulative data records while e.g. lights-off occurs 434 times in each of these data sets.

**Figure 4:**
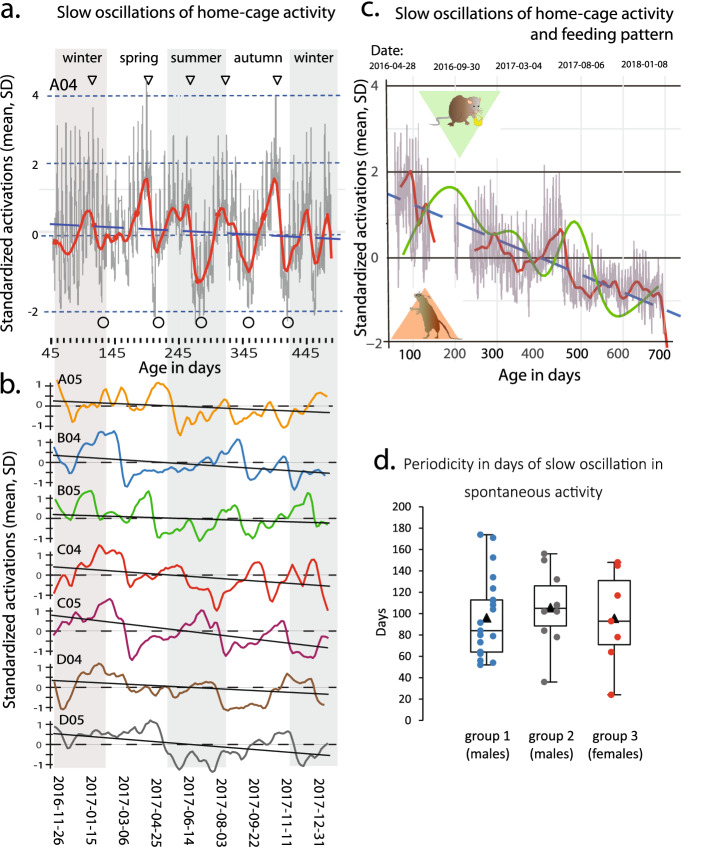
(**a**) Upper panel shows plot of normalized (see Methods) activations (ordinate is mean and standard deviations of the mean) for cage A04 also shown in Figs. 1c and 2, with highs indicated by inverted triangles (same as in Fig. 1c) and lows by open green circles at the bottom. On top of the slow rhythm and having a similar amplitude is the recurring response to the cage-change which occurs with the period of 1 week (for further details of the plot see Supportive information Fig. S3). The slow rhythmicity is indicated by the red curve and the interrupted blue line is the linear regression showing the over-all decrease in activations with advancing age. The data has been plotted against dates (abscissa bottom), age in days (abscissa middle) and with the seasons of the year as background (abscissa at the top, intervened grey-and-white vertical stripes). (**b**) Lower panel is the slow rhythmicity (continuous line with different color for each cage) of the complementary 7 cages in Group 1 along with the linear regression of the each cumulative record showing the decrease with advancing age (black line). The abscissa ordinates are the same as in a. (**c**) shows normalized activations (as in a) up to an age of 699 days in one cage belonging to group 2 (Cage E; activations data available in Supportive information Fig. S2I) where food consumptions (average across one week; see Methods) was measured (green line). The red curve indicates the slow oscillation in activations while the interrupted blue line is the regression of overall change in activity with advancing age. The slow oscillations in food consumption (green) and activations (red) are highly correlated r^2^ = 0.72. (**d)** Boxplots visualizing the period of slow oscillations in the three groups of cages. The observation from each cage in each of the groups have been indicated by separate color-filled circles. The average period in group 1–3 is 96, 106 and 96 days, respectively.

Using spectral analysis, the full data-set with minute resolution of each cage was analyzed to reveal clustering of activity with different periodicity and plotted as a periodogram (Fig. 3). From these plots it is evident that high levels of activity peak around certain expected frequencies such as lights-on and lights-off (circadian rhythm) at 12 and 24 h. (C.f. Fig. 1a,ba,bc) and at 168 h. (7 days; Fig. 1a,bb) which is the instant impact on activity by the weekly cage-change. In addition we observed both low-amplitude high-frequency peaks of unknown origin and a high amplitude cluster with long wave-length (Fig. 3). The peaks associated with the identified external triggers and several of the low intensity high-frequency peaks were synchronized across cages (Fig. 3) while the high intensity low-frequency cluster seems to be asynchronous across cages (Figs. 3, see also Figs. 2 and 4a,b).

To deepen the analyses of the low frequency oscillation in spontaneous in-cage activity, we generated smoothed time-series centered on the mean of the minute-averaged activation data for each day and cage (see Material and methods) and plotted daily averaged activity together with the smoothed activity data against, age of the animals, calendar date and season of the year (Fig. 4a,b; see also Supportive information Fig. S5). The calendar dates of the slow-frequency highs and lows can be extracted directly from the plots (or by the script) and indicates that the period is in the range of 2–4 months, with an average of 97 days for all groups of cages in this study (Fig. 4d) and, furthermore, that the slow oscillation in activity level is not synchronized between cages or locked to seasons of the year albeit having a frequency of about 4 cycles per year (*idem*).

**Figure 5:**
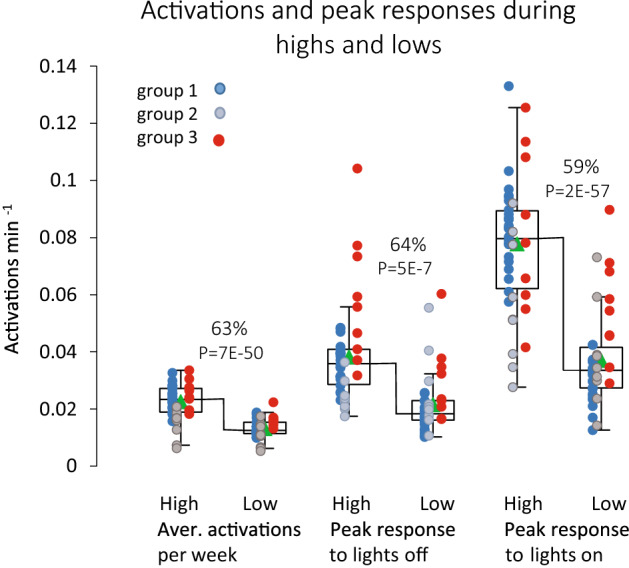
Boxplots of weekly average activations during weeks of highs and lows, respectively, followed by peak response in activations (above weekly average) in the responses to lights-off (middle) and lights-on (right) for the three groups of cages during weeks of highs and lows, respectively. Cages belonging to the different groups (Material and Methods) have been indicated with different colors; blue is group 1, grey is group 2 and red is group 3.

Using the calendar dates of highs and lows, we calculated that the weekly average in-cage activity during highs was 63% higher than during weeks of lows (P = 7E−50, Fig. 5). We also confirm that female mice have a higher in-cage activity than males (P = 1E−3 and interaction sex*highs-lows P = 2E−3; see Material and Methods). Comparisons of the responses to lights-off and lights-on during highs and lows (Fig. 5) revealed that response amplitude was scaled to the difference in the over-all activity and during weeks of highs was 64% and 59%, respectively, above the response recorded during lows (P = 5E−7 and P = 2E−57, respectively). Thus these responses are more vigorous in terms of peak activations during highs than lows (*idem*).

**Figure 6:**
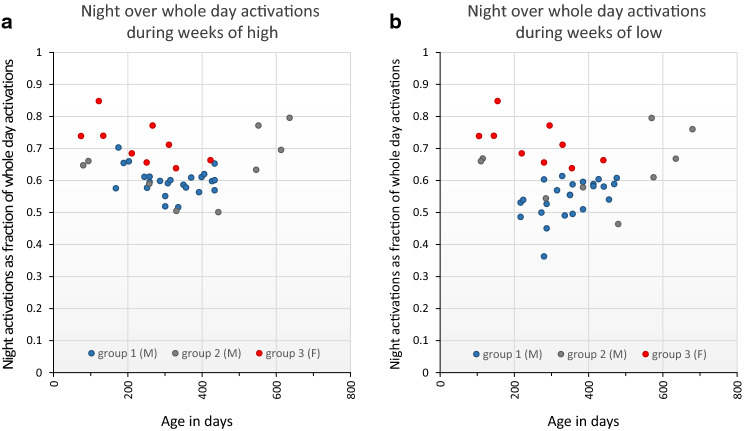
Plots of the fraction of the daily home-cage activity that occurs during the dark hours (night) over weeks with high (left panel (**a**); average 63%) or low (right panel (**b**); average 60%) level of home-cage activity, respectively. Ordinate is fraction of total daily activations occurring during the dark period and abscissa indicates age in days of the animals. The cumulative data records covers 434 days for group 1, 426 days for group 3, and 565 days for group 2. Key to cohort group at the bottom of the graphs.

Laboratory mouse strains are considered nocturnal^50–52^, i.e. show the greatest activity during the dark hours. However some mouse strains have a preference for being active day time, i.e. they are diurnal^53^, and there is evidence that nocturnality vs diurnality may at least to some extent be context dependent^12^. Commonly used laboratory strains like the C57BL/6 are under facility housing conditions nocturnally active while day time is the main period for resting^8^. Here we analyzed on a weekly basis the proportion of total daily activity that occurred during the dark hours and could not detect any consistent difference between weeks of highs and lows (P = 0.11) or over time (P = 0.10); 63% (during highs) and 60% (during lows) of the total daily activity occurred during the dark hours (Fig. 6a,b). In contrast, there was a significant impact by sex (P = 2E-10), with females showing a stronger preference for activity during the dark hours (72%) than males. Thus, the circadian rhythm of these nocturnal mice are essentially preserved between highs and lows.

In 6 of the 14 cages analyzed we had access to food consumption (see Materials and methods) and the ad libitum feeding behavior also show a recurring variability over time with a periodicity similar to the slow oscillation in spontaneous activity. As depicted in Fig. 4c there was a close covariation between these two metrics with the increase in feeding following upon a period with increased activity. In contrast body weight (i.e. in this study the sum of body weights per cage) increased up to an age of about 500 days without any major variation corresponding to the alterations in activity and is thus inversely correlated to over-all in-cage activity with only small alterations across consecutive weeks (See supportive information Fig. S5a-e). We also analyzed the week-to-week change in summed body weight cage^-1^ and in-cage activity, respectively, and could not detect any consistent correlation between these two metrics which appear to alternate between being in-phase and phase-shifted (See supportive information Fig. S5).

## Discussion

Using DVC records of cage-floor activity extending over more than one year of group-held female and male C57BL/6 mice, we provide evidence for a slow season-like oscillation in over-all activity with an amplitude of 1–2 standard deviations of the mean and a periodicity of on average of 97 days, range: 2–4 months (Figs. 1b, 2–4; and Supportive information Fig. S3a-n). We show that spectral and functional time-series analysis using open source applications (see Materials and methods) are useful approaches to characterize in-cage activity and we envisage that this approach can be elaborated further and expanded to time–space since the DVC™ provide a spatial resolution as well (see also^54^). Our results also confirm previous data that female mice of the C57BL/6 strain are more active than male mice^8^ and add that a further sex difference is that females allocate a significantly larger fraction (72% ± 5%SD) of the daily activity to the dark hours than male mice do (60% ± 8%SD). The season-like oscillations in spontaneous activity described here did not alter the preference for being nocturnally active and to rest during day time (Fig. 6). The male mice used here were delivered by two different vendors and should be considered sub-strains of the C57BL/6 strain which may differ in several respects^55–60^. However, our main finding of an infraradian rhythmicity in activity was evident and similar in all three groups of cages with mice studied suggesting that this trait is not overtly vendor or sex sensitive. The rather small groups of cages does not enable an in-depth analysis of minor differences between vendors or sex with sufficient power but has to await amassment of more recordings and then preferentially amassed from different facilities.

Spectral analysis of activity clustering with different periods (Fig. 3) revealed a number of supraradian frequencies with major peaks at lights on/off every 12 h followed by the circadian rhythm at 24 h (*idem*). High frequency rhythms were often synchronized across cages signaling that they had an external timer. We do not know the origin of most of these short frequency clusters yet but by using spectral analysis and time series combined with more detailed annotation of parameters in the holding room (staff mobility, cages in/out of the rack, vibrations, noise, spectral or intensity fluctuation of the illumination, etc.)^24,25^ we may be able to map their origin in the future. For example, short bursts of recurring activity during daytime in between periods of rest may be a trailing effect, where vibration and noise from one cage trigger activity in the next cage and so on; such patterns being close to synchronized may be possible to track down with the DVC recording technique. In the long wave-length spectrum, the synchronized response to the weekly cage change and the slow season-like oscillation were the peaks with the highest amplitudes (Fig. 3). The latter is not synchronized across cages and as shown by the time-series analysis (Fig. 4) disclose some variability in period both within and between cages (see also below). Because of this and that these clusters occur only 3–5 times in the cumulative record of each cage, the spectral analysis could not determine the period of the slow oscillation with any precision except for having a period longer than 60 days. As indicated by our time series analysis the period is in the range of 2 to 4 months with an average period close to 100 days. This slow—season like—oscillation in spontaneous activity was recorded from cages with male and female C57BL/6 mice maintained under constant conditions (Material and methods) and is of unknown origin. A number of external factors such as holding temperature could be triggers of this rhythmicity. The average temperature difference between winter-spring and summer in the holding room is small and was less than 0.5 °C during 2018, i.e. very similar to the recorded weekly variation in the holding room temperature (Supportive information Fig. S2). The narrowness of the temperature clamp was confirmed in the records for 2020 (data available on request). Visual, sound/vibrations or olfactory cues could pass the barrier protection under which they were housed and signal seasons or other recurring events in the facility environment. However, the lack of synchronization between cages or to the seasons of the year (Figs. 2 and 4; see also Supportive information Fig. S2) suggest that the slow oscillation in daily activity is intrinsic to the group of mice in the cage and may be a behavioral trait of this laboratory mouse strain^9^. As such it should be taken into consideration in a range of experiments were mice behaviors are used as a bio-assay^1^ and may also be a metric of value in the context of animal welfare surveillance. Mice are group-living, social creatures thus it is not surprising that behavioral rhythmicities of daily activity is coherent among the group members especially since their living quarters are confined to the cage^3,12,61^. Although we have no information on in-cage activity among mice held in open-cages, the relative isolation that the IVC system provides for a cage is probably significant for the observed asynchronicity between cages.

Even though we have no evidence, this seasonal-like rhythmicity could either be generated by an endogenous oscillator which do not synchronize with an external timer (type II rhythm^9^) or a free-running endogenous generator lacking external timer to synchronize with (type I rhythm^9^). In favor of the latter is the variability in period (2–4 months) of the slow rhythmicity which bears resemblance to the variation of the circadian rhythm when being devoid of external timer to synchronize with (~ 21–27 h c.f. 24 h of the earth’s solar day).

Seasonal variation is a common phenomenon in natural settings of mammalian and non-mammalian species and includes reproduction cycle, hibernation, migration and moulting^9,11,17,62^. There are considerable evidence showing that seasons may be internalized by photoperiodic stimuli^11,17,63^. Thus, difference in photic stimulus (longer/shorter nights) over seasons impacts the production of melatonin in the pineal body so that with increasing length of the dark hours, the duration of melatonin secretion increases and vice versa^17,23^. “To prepare in advance” is an important capacity for survival in the wild, for example foraging in advance of seasonal hibernation. The free-running circadian rhythm (21 h-27 h) is generated endogenously by feedback loops involving mainly 10 gene products (Clock, Bmal1, Per 1–3, Cry1 and 2, Ror-α, -β and -γ)^13,14^ expressed in neurons of the Suprachiasmatic nucleus (SCN) that normally synchronizes with the earths solar day (24 h). The entrainment of the circadian rhythm to a day occurs by suppressing the conversion of serotonin to melatonin^21,22^ which is secreted during the dark hours only^17,23,51^. While mice of the C57BL/6 strain expresses all the clock genes in the SCN and peripheral organs it carries a loss-of-function mutation in the arylalkylamine N-acetyltransferase gene, encoding a rate-limiting enzyme in the conversion of serotonin to melatonin^64–66^. Thus the common mechanism to decode earth’s solar day or internalization of seasons through photoperiodic stimuli does not work in C57BL/6 mice. Nevertheless, these mice respond to lights-on and lights-off and as shown elsewhere phase-shift of the circadian rhythm can also be induced by non-photic stimulus^67^.

The oscillation of the key clock gene products is not exclusive to the SCN where direct interaction occurs with the neuroendocrine (HPA) system within hypothalamus and other key regions of the brain^25^, but take place in peripheral tissues of the body as well^14^ explaining the circadian oscillators’ profound impact on bodily functions. The concerted impact of the circadian rhythm generator on peripheral tissues is mediated through secreted melatonin, endocrine signals and the autonomic nervous system^13,25^. In contrast to the circadian rhythm, the onward signaling to the target organs (ovary and testis in the case of reproduction) of circannual rhythms are not fully understood. The TSH (thyroxin stimulating hormone; subunit B)-and Tx (Thyroxin; T3 and T4) axis via the thyroid hormone activating (Dio2) and deactivating (Dio3) enzymes is one candidate system that has attracted interest^68–70^, and experimental evidence supports a link between melatonin secretion and circannual oscillations of this axis (*idem*). Further, the TSH-Tx axis has the capacity to impact gonadal hormones as well as growth and metabolism of peripheral tissues. Although adaptation to seasons often appears to make use of the time-keeping machinery of the circadian clock i.e. photoperiodic stimuli converts to changes in the production and secretion of melatonin, the seasonal variation in the TSH-Tx axis is independent of the clock gene Per 2^70^. Moreover, for non-mammalian species infraradian rhythmicities independent of the circadian clock machinery have been discovered^18,19^. Of interest to our observed covariation between feeding rhythm and slow oscillation in spontaneous activity, is the food-anticipatory-activity (FAA) generated by the food-entrained-oscillator (FEO). FAA rhythmicity has been shown to be independent of the Per 1–3 clock genes and suggested to use a different time-keeping mechanism than the circadian clock^71^. Taken together there are evidence suggesting that infraradian biorhythms can depend on, or be independent of, the time-keeping of the circadian clock.

Our data, including the covariation of the amplitude of stimuli responses with the general activity level during highs and lows (Fig. 5), provide unbiased support to earlier observations indicating that behaviors show season-like (circannual) variations in laboratory mice kept under constant conditions^27,28^. In addition, there are also variances in mice behavioral responses within the phases of the circadian rhythm^72^ and infraradian variations in activity linked to the circadian circuitry^48^. Taken together, rhythmicities need to be considered in behavioral assessments used as the high-end readout of organismal function and in other bioassays of disease models and interventions, as well as in phenotyping screens^1,4,6,72–74^. Strains originating from the house mouse (*mus musculus*) are currently the most widely used mammalian animal models in experimental research, a main reason being that strains of this species were very useful in the infancy of genetic engineering and because of this will be the first mammalian species having all its genes characterized^21^. The fact that most of the laboratory strains of the house mouse used in research show aberrations in melatonin secretion governed by photoperiodic rhythmicity and yet disclose a circadian rhythmicity synchronized to lights on and lights off, and as shown here infraradian biorhythmicity in activity, remains a challenge to investigate^24,65^.

Although the driving engine of the slow oscillation in spontaneous activity described here remains unknown, the large amplitude of the recurring variation in activity should have an impact at the system level. By using the feed consumption data available for 6 of the 14 cages studied, we found that feeding and activity level co-varied to a significant degree linking the metabolic state of the animals with the alternating base-line activity level (Fig. 4c). In contrast, body weight did not show a corresponding slow rhythmicity but was inversely correlated with activity level, at least up to an age of 500 days. This is not entirely surprising because body weight is a complex metric reflecting the balance between energy uptake, storage and energy expenditure.

In this context it should also be mentioned that observations on somatic variations in inflammatory responses, host-parasite interaction as well as epidemiology of ulcerous dermatitis common to the C57BL/6 strain, show distinct season-like variations during the year^75–77^ suggesting that circannual oscillations are not limited to behaviors in laboratory mice. Season-like variations in behaviors and somatic state of the widely used C57BL/6 mouse have hitherto not received much recognition but will need to be seriously considered in future experimental designs.

## Materials and methods

### Mouse strain, sex and age

Cohorts of specific pathogen free (SPF, according to FELASAs exclusion list^78,79^) male and female C57BL/6 mice were delivered from Charles River (strain C57BL/6 J), Germany, or Janvier labs (strain C57BL/6Rj), France, (Table 1). Transported by car and upon arrival subjected to a brief health check, at 6–8 weeks of age mice were randomly allotted to cages and grouped 4 or 5 per cage.

**Table 1:** Table of animals used, grouping, DVC recordings and husbandry conditions.

Cohort	C57BL/6 J males, Grp 1	C57BL/6Rj males, Grp2	C57BL/6 J females, Grp3
No. cages	8	3	3
No. animals	40	12	12
Vendor	Charles River, Germany	Janvier, France	Charles River, Germany
Arrival	Arrival date: 2016-Nov-22	Arrival date: 2016-May-10	Arrival date: 2016-Nov-29
Acclimatization	DVC—acclimatization 1–2 week–5/cage	DVC—acclimatization 1–2 weeks–4/cage	DVC—acclimatization 1–2 weeks–4/cage
DVC record start	DVC recordings started at age 49 days	DVC recordings started at age 70 days	DVC recordings started at age 50 days
DVC record length	DVC recordings until age 483 days	DVC recordings until age 699 days. Animals were out of DVC during 64 days	DVC recordings until age 476 days
Length of DVC record	Record length is 434 days	Record length is 565 days	Record length is 426 days
	**Husbandry conditions and routines**		
Bedding (autoclaved)	Aspen chips	Aspen chips	Aspen chips
Food (irradiated)	SDS RM3	SDS RM3	SDS RM3
Enrichment	Red house, Sizzle nest	Red house, Sizzle nest	Red house, Sizzle nest
Humidity holding room	40–60%	40–60%	40–60%
Temperature holding room	19–23 °C	19–23 °C	19–23 °C
Dark/light cycle	12/12	12/12	12/12
Other husbandry routines	Weekly cage-change, no weighing	Weekly cage-change incl. body and feed weighing	Weekly cage-change incl. body and feed weighing
Microbiological status	FELASA SPF	FELASA SPF	FELASA SPF
Health check	Daily	Daily	Daily
Handling	Forceps or cupped hand	Forceps or cupped hand	Forceps or cupped hand

### Holding and husbandry conditions

Mice were kept in GM500 (Tecniplast SpA, Italy; type IIL) cages in a DVC system (Tecniplast) (see Supportive information Fig. S1). IVC cages are individually ventilated with 75 HEPA14 filtered air exchanges per hour, the air is taken from the holding room and let out through a separated outlet. Holding room air temperature was clamped at 21 ± 2 °C (Fig. S2), and air humidity within 40–60%. However, for most of the time the temperature clamp was narrower varying on average with ≤ 0.5 °C across season and during the calendar week (Fig. S2); including single peaks the range was 1.5 °C during 2018 (idem). According to our own experience, this variation is similar to the variation registered in-cage at our facility across day (low) and night (high) time. The holding room had a 12-12 h dark/light (DL) cycle with instant switch between light (white light 15–40 lx inside the cage) and dark. Ad libitum access to food (SDS RM3 irradiated pellets) and weakly chlorinated water through water bottle that is changed every week. Cages had 100 g aspen chips (Tapavei, Finland) as bedding, sizzle nest and red polycarbonate mouse house (Tecniplast Spa) as enrichment. The husbandry routines included weekly cage change (whole cage was changed but red house and some of the soiled bedding were moved along with the animals to the new cage) and in two of the cohorts (group 2 and 3) included body weighing also weighing of feed on a fixed weekday (Table 1). Handling of the mice by staff was either by using cupped hand or by forceps at the tail root, all mice in the different groups were subjected to both handling routines. The holding unit was subject to health inventories according to FELASAs recommendation for a sentinel reporting system (i.e. the subjects of the study was not directly affected by the health inventory) four times a year^78,79^ and during the study period the output from the sentinel system in use met the FELASA exclusion list for specific pathogen free animals (SPF). Surveillance of health and welfare included daily check-ups and weekly individual examination during the cage-change and weighing. Health is assessed according to a scoring list deployed at all facilities on Karolinska Institutet, amended by special requirements stated in the ethic permit. When we weighed the animals and the feed, these metrics along with the scoring list formed the basis of the welfare and health check-ups. As needed the designated veterinarian of the facility was consulted.

### Ethical considerations

Both husbandry routines and applied procedures followed applicable guide-lines and were agreed upon, reviewed and approved by the Regional Ethics Council, Stockholms Regionala Djurförsöksetiska nämnd; project licenses N116-15, N115/15 and N184/15 plus amendments. No special requirements for health and welfare checks beyond those already implemented at the facility (see above) were required by the permits.

### DVC recordings

In total recordings were collected from 14 cages maintained at the facility for an extended period of time. At the time we noted that there appears to be an oscillation of in-cage base-line activity, we decided to maintain available cages in the DVC for an extended period of time albeit they originally were allocated to different subprojects. This transfer was executed before any intervention had been conducted on these animals. In doing so we saved in number of animals used and also could exploit for this purpose the DVC records thus far obtained. The cumulative DVC records belong to 3 different groups (Table 1). Group 1 with 8 cages each populated with 5 male mice and this group is age- and season matched with group 3 having 3 cages with 4 females in each cage (Table 1). Group 2 with 3 cages housing 4 male mice per cage arrived at the facility about 6 months earlier than the two other groups (Table 1).

The core of the DVC system is an electronic sensor board installed externally and below each standard IVC cage of a rack (Fig. S1). The sensor board is composed of an array of 12 capacitive-based planar sensing electrodes. A proximity sensor measures the electrical capacitance of each of the 12 electrodes 4 times per second (i.e. every 250 ms). The electrical capacitance is influenced by the dielectric properties of matter in close proximity to the electrode, leading to measurable capacitance changes due to the presence/movement of animals in the cage above. Thus, movements across the electrode array are detected and recorded as alterations in capacitance (Fig. S1). By applying custom designed algorithms to the collected data we can infer information regarding in-cage animal activity^8^. For this study, we used the first-order difference of the raw signal (i.e., capacitance measured every 250 ms) as the basic metric of animal activity. More specifically, we take the absolute value of the difference between two successive measurements for each electrode (signals spaced 250 ms apart) and compare it against a set threshold (capacitance variations due to noise) to define an activity event. This metric thus considers any animal movement that generates a significant alteration in capacitance, an activity event^8^. In this study we have used the average number of activations across all 12 electrodes per minute, i.e. the average number of activation events across the 240 time-slots per minutes of all twelve electrodes (for activation plots see Supportive information Fig. S1). For further details on use of the spatial resolution provided by the electrode array, please see^8,80^. Note, this activity metric represents the overall in-cage activity generated by all mice in a cage from any electrode and is not tracking activity of individual group-housed animals (for activation plots of the cages used in this study see Supportive information Fig. S3A-N). However, as shown elsewhere there is a close correlation between the metric activations used here and locomotor activity of the animals in the cage^80^. Moreover, the DVC records activity on the cage floor only.

### Data processing and statistics

Data were processed through scripts in R (version 3.5.0). We used spectral analysis, functional data analysis, linear regression and statistical analysis of longitudinal data; all scripts are open source and available for R.

The data file from each cage (see below for data availability) was plotted with minute resolution as activation plots (Supportive information Fig. S3A-N) or heat maps (see example in Fig. 1a,c) to visualize variations in activations across day and night (circadian rhythm; Fig. 1a), and over time (Fig. 1c). Heat maps were constructed using ‘ComplexHeatmap’ script (version 2.0.0) available for R^81^. By linear-regression across the whole recording period, the over-all change in activity was assessed for each cage and expressed as coefficient of determination (r^2^) with level of significance (Figs. 2 and 4, and Supportive information Table S1).

Spectral analysis and periodograms were generated using the periodogram-function from the ‘Time Series Analysis’ (TSA) library v 1.2^82^. Minute-averaged activity data (across electrodes and samples min^-1^) was used and each cage was analyzed separately. In order to account for the small but consistent decline in activity over time and make the time-series stationary, a linear model was fitted (Activity ~ Time) for each time-series where the residuals of the model was used as de-trended times-series. Stationarity was confirmed using Augmented Dickey-Fuller Test where a p-value of < 0.01 was considered a stationary time-series. The resulting periodogram of the de-trended activity-data was plotted with wavelength (i.e. time between peaks) on the x-axis and spectral intensity on the y-axis (Fig. 3). In the same fashion, spectral intensity as a function of time was analyzed for each cage using the Spectro-function from the ‘seewave’ library v 2.1.4^83^ with a window-length of 7 days (10 080 min) and a smoothing overlap of 25%. To improve readability, the output was divided into three separate graphs covering wavelengths 0–12 h, 12–240 h and 240 h (10 days) to Infinity. Pre-set graphical parameters were used where red–green–blue-black denote higher to lower spectral intensity.

For the generation of smoothed time-series, minute-averaged activity data from each cage was centered and scaled to a mean of 0 with standard-deviation as principal unit where 1 is activity 1 standard-deviation above the average activity throughout the observation period. Smoothing was achieved by applying a 24 h rolling mean followed by a 5% loess-regression using the loess Fit-function from the ‘Linear Models for Microarray Data’ (limma) library v. 3.42.0^84^. Graphs were thereafter generated by plotting 24 h averaged activity over time together with the smoothed activity data (Fig. 4a,c and Supportive information Fig. S4).

The peak response to lights-on^8^ was expressed as the highest number of activations min^-1^ minus base-line activity (see below) during 180 min of recordings prior and subsequent to the time at which lights-on took place^8^ the day after cage-change (Figs. 1a,bc, and 5). Records with missing data (exceeding 59 min) or any procedures, or any other non-regular holding unit event interfering with the recording period of 360 min, were excluded.

Like-wise the peak response (activations min^-1^ minus base-line activity min^-1^) to lights off covered records  240 min subsequent to time of lights off and is the average peak response during a cage-change cycle at a high or a low (Figs. 1a,bc and 5). Exclusion criteria were the same as for lights on.

Day and night time activity, respectively, was expressed as the average activations min^−1^ during the period with lights-on and lights off, respectively, while daily activity is the average activations min^−1^ across 24 h. Weekly average activations per minute is the corresponding value calculated for seven days (= cage-change cycle) and used to express base-line in-cage activity during weeks of highs and lows, respectively (Fig. 5 and below). The fraction of the daily activity occurring during lights-off was calculated from these metrics (Fig. 6). Box plots indicate median, 25–75% quartiles with max and min as bars. Filled triangles indicate mean value.

We used the rank-based analysis of variance-type statistic (ATS) to test differences within and between cages and sex^85^. Analyses were implemented using the nparLD package in R statistical software^86^. npar-LD is a non-parametric test for longitudinal data that does not require strong assumptions as the Repeated Measures ANOVA^8^. We considered cages as subjects, high-or-low and observation-order as within-subject factors (“sub-plot” repeated factors), and sex as between-subject factors (“whole-plot” factor). According to authors’ terminology^86^, we used F1-LD-F2 (sex as the whole-plot factor and with high-or-low and observation order (age) as the repeated ones). Since high-or-lows are arranged chronologically the observation-order is interpreted as the age-factor.

### Data files and data availability

All 14 data sets (one for each cage used) arranged in three groups (1–3) are available as three comma delimited csv files at: datadryad.org (https://doi.org/10.5061/dryad.n5tb2rbsf).

All data files have the following column headings for each cage:

Example from file group 1.
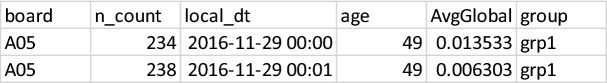


Where **board** is the cage ID, **n_count** is number of samples min^-1^, **local**_**dt** is local date followed by time stamp (CET), **age** is the age in days of the animals inside the cage**, AvgGlobal** is the average number of activations min^-1^ across all twelve electrodes, and **group** is the cage cohort that the cage belongs to.

The CSV files have been arranged to not violate the maximum number of input-lines (1 million lines) of certain applications, thus, data of each cage within each group have been listed in columns side by side.

Lights-on and Lights-off, respectively, happened every day at 04:00 (h:min; zeitgeber time 0) and 16:00 (h:min; zeitgeber time 12) dt (local).

### Animal testing ethics

The experimental procedures were agreed upon, reviewed and approved by the Regional Ethics Council Stockholms Djurförsöksetiska nämnd; project licenses N116-15, N115/15 and N184/15 plus amendments. In the reporting we adhere to the ARRIVE guide-lines.

## Supplementary Information

Supplementary Information 1.

Supplementary Information 2.

## Data Availability

All data files used for analysis will be made available on Dryad.org once the paper is considered for publication: datadryad org. https://doi.org/10.5061/dryad.n5tb2rbsf. Ms. is available on preprint server: bioRxiv 2020.09.09.290148; https://doi.org/10.1101/2020.09.09.290148.
