# Supplementary material for: Major oscillations in spontaneous home-cage activity in C57BL/6 mice housed under constant conditions
Source: Sci Rep. 2021 Mar 2;11:4961. doi: 10.1038/s41598-021-84141-9 (PMC7925671; doi:10.1038/s41598-021-84141-9)
Supplement: Supplementary file 1 — Supplementary Information 1. [file 41598_2021_84141_MOESM1_ESM.pdf]

Manuscript:
Major oscillations in spontaneous home-cage activity in C57BL/6 mice housed under constant conditions
Karin Pernold, Eric Rullman and Brun Ulfhake, Clinical physiology, Department of Laboratory medicine, Karolinska Institutet

Supportive information 1 (1:2)

Contents:

Figure S1 DVC system description and illustration of the metric *activations* as a plot and as heat map.

Figure S2 A, B Plot of the temperature log for the holding room during winter-spring-summer of 2018.

Figs. S3 A-N see Supportive information 2:2.

Figure S4 A, B High resolution plot of the time-series of activations recorded from cage A04

Figure S5 A-M Change in body weight of animals in group 2 and 3 across the study period, and plots of weekly change in body weight and average activity per cage over time.

Table S1 Linear regression of average daily activity as a function of age in days for each cage.

Figure S1

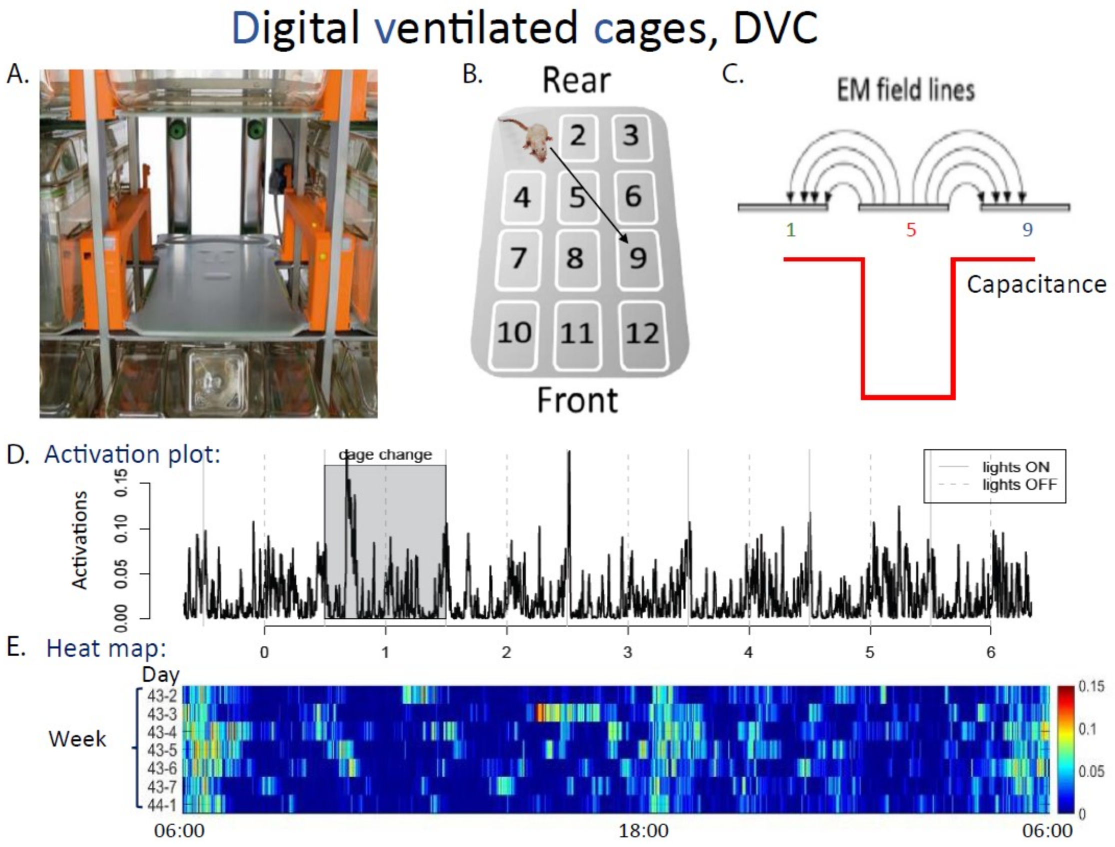

Legend to Fig. S1 A-E

System description, the metric activations and illustration of plot and heat map of activations.
(A) Top left panel shows the grey DVC plate inserted into its slot beneath the cage. (B) The DVC plate holds 12 capacitance recording electrodes front to rear and generates a weak electromagnetic field (C; EMF)[1]. When a mouse moves (B) over the cage bottom from electrode #1, crosses over electrode #5 to reach electrode #9 (arrow in B), this is picked up as a transient drop in capacitance of electrode #5 (red trace in C). Discrete alterations of electrode capacitance is annotated as *activations*[2] and correlates to CCD validated locomotor behavior of mice inside the cage[3] and is the metric used here as indicator of home-cage activity. Activations are displayed either as plots of activations against time (D) showing average activation min^{-1} plotted versus weekday or as heat maps (E) where average activations min^{-1} have been color coded according to scale at the right end of the panel. E (modified after [2]).

Manuscript:
Major oscillations in spontaneous home-cage activity in C57BL/6 mice housed under constant conditions
Karin Pernold, Eric Rullman and Brun Ulfhake, Clinical physiology, Department of Laboratory medicine, Karolinska Institutet

Figure S2 A, B

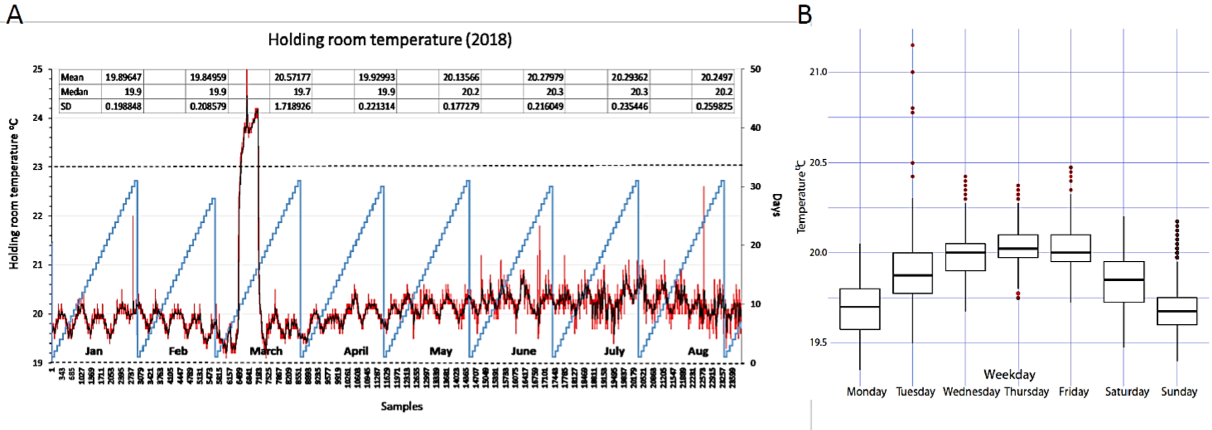

Legend to Fig. S2 A, B

Plot of the temperature log for the holding room during winter-spring-summer of 2018.
To comply with the national regulations of Sweden the holding room climate control allows a variation of no more two centigrade around 21°C. As shown in (A) in reality the temperature clamp is much narrower for most of the time. The plot (A) shows the variation in holding room temperature with hour resolution from January through August 2018. Left ordinate is holding room temperature and system readings per hour indicated in red with the moving average shown as a black trace; the abscissa is samples and right ordinate is days of the months indicated by the light blue ladder for each months. On top of the diagram is a table showing average, median and standard deviation of the temperature for each month. In (B) boxplots of holding room temperature (ordinate) for each weekday (abscissa) across the recorded period.
Except for 1½ week in March when the control system was out of order and single spikes throughout the period, the temperature variation is within 19-21 °C, with winter-spring versus summer showing a shift of ~0.4 °C in average temperature of the holding room. This seasonal variation has about the same magnitude as the variation across the work week (B), with temperature being at a low on Monday morning building up to top levels Thursday-Friday. The variation in temperature during a week probably reflects the use of the building by staff and various equipments related to staff work.

Figure S4 A, B

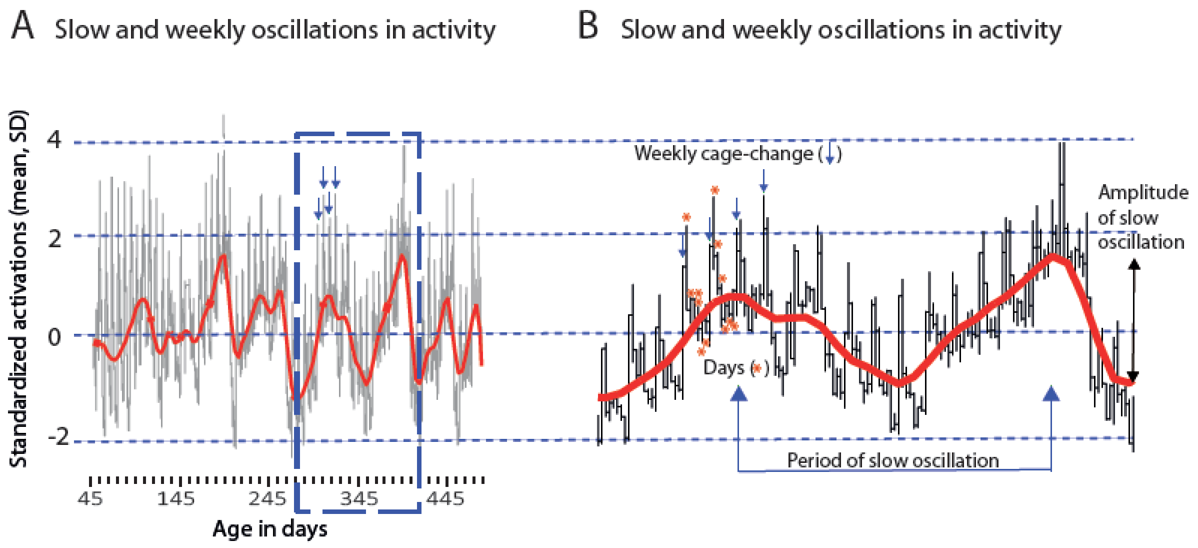

Legend to Fig. S4 A, B

Time-series plot of activations recorded from cage A04. A shows a medium resolution plot of the weekly and circannual rhythms of in-cage activity recorded with the DVC (also shown in Fig. 4A). On the ordinate is deviations from the mean activity of the full time-series (mean=0), where plus or minus one standard deviation implies a higher or lower level of activity corresponding to one standard deviation away from the mean activity (for further information see Material and methods). Age in days is shown on the abscissa. The framed area (blue) in **A** is shown at higher resolution in panel **B**. Note that in A, on top of the slow circannual oscillation in activations min^{-1} (red line), a second oscillation is evident with a similar amplitude as the slow but with a period of 7 days (arrows). This oscillation correspond to the animals' response to the weekly cage-change. Oscillations in activations with higher frequencies (as diurnal-nocturnal rhythms) cannot be separated at this level of resolution. In **B** which shows the framed area in A at higher resolution, an oscillation in activity level across days (indicated by red stars) in-between cage-changes (blue arrows) can be seen. This oscillation repeats every cage-change cycle. In addition, the period and amplitude of the slow rhythmicity have been indicated.

Figure S5 A-M

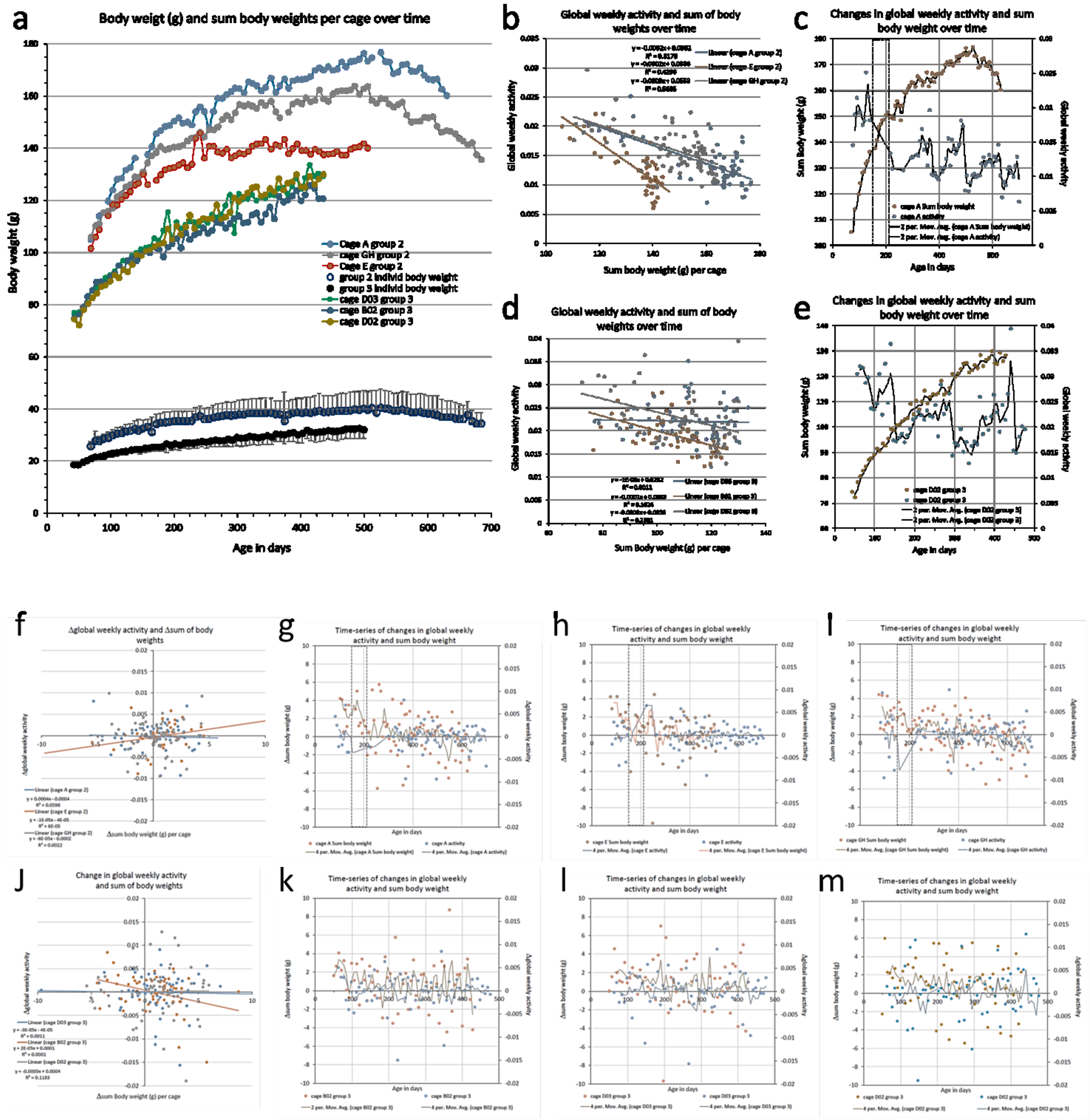

Legend to Fig. S5 a-m

Change in body weight of animals in group 2 and 3 across the study period, and plots of body weight and weekly average activity per cage over time.

In (a), the body weight (mean and SD) of the individual mice in group 2 and 3, respectively, have been indicated (females with filled circles and males with open circles). Weight in grams on the ordinate and age in days is indicated on the abscissa. In the same diagram the week by week change in the sum of body weights per cage for group 2 (males in cage A, D and GH) and group 3 (females in cage B02, D02 and D03) are shown and each cage indicated with different color. Body weight increases up to an age of about 500 days, at more advanced age (only males) there is a drop in body weight (see panel **(a)**, at 600-700 days of age) while activity is unchanged or decreases.

There was a significant negative correlation between the sum of body weights per cage and the average weekly activity in 5 of the 6 cages (panels (b), (d)). To examine if changes to the sum of body weights of a cage co-varied with the slow oscillation of in-cage activity, the time-series of these two metrics were plotted (two examples are shown in panels (c) and (e)) showing that there is no close covariation between sum of body weights (orange filled circles, black trace is moving average) and weekly average in-cage activity (blue filled circles; black trace is moving average). Rectangle in (e) with interrupted line indicates period when the cage was not in DVC, i.e. activity data is missing.

f and **j** shows the correlation between weekly change (Δ) in activity and sum of body weights, respectively, for three male (F) and three female (J) cages of group 2 and3 (key to color coding in panel). Except for one female and one male cage there is no correlation. **g-i** and **k-m** show the time-series of weekly change in activity (blue filled circles, blue trace is the moving average) and sum of body weights (yellow filled circles; yellow trace is the moving average) for the same male and female cages as in F and J, illustrating that these two metrics showed alternating periods of being in-phase and phase-shifted over time. Note, in these panels age on the abscissa covers 700 days for male cages and 500 days for female cages. Rectangle indicated by interrupted line (G-I) shows period when the cage was not in DVC, i.e. activity data is missing.

Table S1

Linear regression of average daily activity as a function of age in days for each cage.

Group - Cage ID	slope	R2	*P*
1-A04	-2E-6	0.00007	1E-10
1-A05	-7E-6	0.0009	0
1-B04	-2E-5	0.0075	0
1-B05	-6E-6	0.0008	0
1-C04	-1E-5	0.0011	0
1-C05	-2E-5	0.0097	0
1-D04	-7E-6	0.0016	0
1-D05	-2E-5	0.0097	0
2-A07	-1E-5	0.0162	1E-5
2-A08	-2E-5	0.0435	2E-5
2-GH	-1E-5	0.0101	1E-5
3-B02	-2E-5	0.0055	2E-5
3-D02	-1E-5	0.0034	1E-5
3-D03	-6E-9	6E-10	5E-7

References

1. Recordati C, De Maglie M, Marsella G, et al. Long-Term Study on the Effects of Housing C57BL/6NCrl Mice in Cages Equipped With Wireless Technology Generating Extremely Low-Intensity Electromagnetic Fields. *Toxicol Pathol.* 2019;47(5):598-611.
2. Pernold K, Iannello F, Low BE, et al. Towards large scale automated cage monitoring - Diurnal rhythm and impact of interventions on in-cage activity of C57BL/6J mice recorded 24/7 with a non-disrupting capacitive-based technique. *PLoS One.* 2019;14(2):e0211063.
3. Iannello F. Non-intrusive high throughput automated data collection from the home cage. *Heliyon.* 2019;5(4):e01454.
