# Supplementary material for: Major oscillations in spontaneous home-cage activity in C57BL/6 mice housed under constant conditions
Source: Sci Rep. 2021 Mar 2;11:4961. doi: 10.1038/s41598-021-84141-9 (PMC7925671; doi:10.1038/s41598-021-84141-9)

Manuscript:
Major oscillations in spontaneous home-cage activity in C57BL/6 mice housed under constant conditions
Karin Pernold, Eric Rullman and Brun Ulfhake, Clinical physiology, Department of Laboratory medicine, Karolinska Institutet

Supportive information 2 (2)

Contents:

Figs. S3 A-N Activation plots of each day of the cumulative records of activations with minute resolution for all 14 cages (A-N) in groups 1 to 3.

General information

Data plotted in A-N are available as csv files at datadryad org. doi:10.5061/dryad.n5tb2rbsf

Legend to Fig. S3 A

Plot of average activations min^{-1} (ordinate) across 371 days to display the activity levels in cage A04 of group 1.
Each row represents one week, i.e. one cage change cycle starting on the morning of cage change day. Vertical red and blue lines indicate time for lights-on and lights-off, respectively, in the holding room. Grey shaded area shows the lights-on part of the cage change day. Each plot has a title stating cage ID, age in days on the first day of the cycle.
Scrolling through the figure using Adobe Acrobate Reader and single-page view very clearly displays variations in activity levels over several weeks.

Legend to Fig. S3 B

Plot of average activations min_{-1} (ordinate) across 371 days to display the activity levels in cage A05 of group 1.
Each row represents one week, i.e. one cage change cycle starting on the morning of cage change day. Vertical red and blue lines indicate time for lights-on and lights-off, respectively, in the holding room. Grey shaded area shows the lights-on part of the cage change day. Each plot has a title stating cage ID, age in days on the first day of the cycle.
Scrolling through the figure using Adobe Acrobate Reader and single-page view very clearly displays variations in activity levels over several weeks.

Legend to Fig. S3 C

Plot of average activations min_{-1} (ordinate) across 371 days to display the activity levels in cage B04 of group 1.
Each row represents one week, i.e. one cage change cycle starting on the morning of cage change day. Vertical red and blue lines indicate time for lights-on and lights-off, respectively, in the holding room. Grey shaded area shows the lights-on part of the cage change day. Each plot has a title stating cage ID, age in days on the first day of the cycle.
Scrolling through the figure using Adobe Acrobate Reader and single-page view very clearly displays variations in activity levels over several weeks.

Legend to Fig. S3 D

Plot of average activations min_{-1} (ordinate) across 371 days to display the activity levels in cage B05 of group 1.
Each row represents one week, i.e. one cage change cycle starting on the morning of cage change day. Vertical red and blue lines indicate time for lights-on and lights-off, respectively, in the holding room. Grey shaded area shows the lights-on part of the cage change day. Each plot has a title stating cage ID, age in days on the first day of the cycle.
Scrolling through the figure using Adobe Acrobate Reader and single-page view very clearly displays variations in activity levels over several weeks.

Scrolling through the figure can be done using Adobe Acrobate Reader and single-page view which very clearly displays variations in activity levels over several weeks.

Legend to Fig. S3 E

Plot of average activations min$_{-1}$ (ordinate) across 371 days to display the activity levels in cage C04 of group 1.
Each row represents one week, i.e. one cage change cycle starting on the morning of cage change day. Vertical red and blue lines indicate time for lights-on and lights-off, respectively, in the holding room. Grey shaded area shows the lights-on part of the cage change day. Each plot has a title stating cage ID, age in days on the first day of the cycle.
Scrolling through the figure using Adobe Acrobate Reader and single-page view very clearly displays variations in activity levels over several weeks.

Legend to Fig. S3 F

Each row represents one week, i.e. one cage change cycle starting on the morning of cage change day. Vertical red and blue lines indicate time for lights-on and lights-off, respectively, in the holding room. Grey shaded area shows the lights-on part of the cage change day. Each plot has a title stating cage ID, age in days on the first day of the cycle.
Scrolling through the figure using Adobe Acrobate Reader and single-page view very clearly displays variations in activity levels over several weeks.

Legend to Fig. S3 G

Plot of average activations min$_{-1}$ (ordinate) across 371 days to display the activity levels in cage D04 of group 1.
Each row represents one week, i.e. one cage change cycle starting on the morning of cage change day. Vertical red and blue lines indicate time for lights-on and lights-off, respectively, in the holding room. Grey shaded area shows the lights-on part of the cage change day. Each plot has a title stating cage ID, age in days on the first day of the cycle.
Scrolling through the figure using Adobe Acrobate Reader and single-page view very clearly displays variations in activity levels over several weeks.

Legend to Fig. S3 H

Plot of average activations min$_{-1}$ (ordinate) across 371 days to display the activity levels in cage D05 of group 1.
Each row represents one week, i.e. one cage change cycle starting on the morning of cage change day. Vertical red and blue lines indicate time for lights-on and lights-off, respectively, in the holding room. Grey shaded area shows the lights-on part of the cage change day. Each plot has a title stating cage ID, age in days on the first day of the cycle.
Scrolling through the figure using Adobe Acrobate Reader and single-page view very clearly displays variations in activity levels over several weeks.

Legend to Fig. S3 I

Plot of average activations min$_{-1}$ (ordinate) across 623 days to display the activity levels in cage A of group 2.
Each row represents one week, i.e. one cage change cycle starting on the morning of cage change day. Vertical red and blue lines indicate time for lights-on and lights-off, respectively, in the holding room. Grey shaded area shows the lights-on part of the cage change day. Each plot has a title stating cage ID, age in days on the first day of the cycle.

Scrolling through the figure using Adobe Acrobate Reader and single-page view very clearly displays variations in activity levels over several weeks.

Legend to Fig. S3 J

Plot of average activations min^{-1} (ordinate) across 623 days to display the activity levels in cage E of group 2.
Each row represents one week, i.e. one cage change cycle starting on the morning of cage change day. Vertical red and blue lines indicate time for lights-on and lights-off, respectively, in the holding room. Grey shaded area shows the lights-on part of the cage change day. Each plot has a title stating cage ID, age in days on the first day of the cycle.
Scrolling through the figure using Adobe Acrobate Reader and single-page view very clearly displays variations in activity levels over several weeks.

Legend to Fig. S3 K

Plot of average activations min^{-1} (ordinate) across 623 days to display the activity levels in cage GH of group 2.
Each row represents one week, i.e. one cage change cycle starting on the morning of cage change day. Vertical red and blue lines indicate time for lights-on and lights-off, respectively, in the holding room. Grey shaded area shows the lights-on part of the cage change day. Each plot has a title stating cage ID, age in days on the first day of the cycle.
Scrolling through the figure using Adobe Acrobate Reader and single-page view very clearly displays variations in activity levels over several weeks.

Legend to Fig. S3 L

Plot of average activations min^{-1} (ordinate) across 427 days to display the activity levels in cage B02 of group 3.
Each row represents one week, i.e. one cage change cycle starting on the morning of cage change day. Vertical red and blue lines indicate time for lights-on and lights-off, respectively, in the holding room. Grey shaded area shows the lights-on part of the cage change day. Each plot has a title stating cage ID, age in days on the first day of the cycle.
Scrolling through the figure using Adobe Acrobate Reader and single-page view very clearly displays variations in activity levels over several weeks.

Legend to Fig. S3 M

Plot of average activations min^{-1} (ordinate) across 427 days to display the activity levels in cage D02 of group 3.
Each row represents one week, i.e. one cage change cycle starting on the morning of cage change day. Vertical red and blue lines indicate time for lights-on and lights-off, respectively, in the holding room. Grey shaded area shows the lights-on part of the cage change day. Each plot has a title stating cage ID, age in days on the first day of the cycle.
Scrolling through the figure using Adobe Acrobate Reader and single-page view very clearly displays variations in activity levels over several weeks.

Legend to Fig. S3 N

Plot of average activations min^{-1} (ordinate) across 427 days to display the activity levels in cage D03 of group 3.

Manuscript:
Major oscillations in spontaneous home-cage activity in C57BL/6 mice housed under constant conditions
Karin Pernold, Eric Rullman and Brun Ulfhake, Clinical physiology, Department of Laboratory medicine, Karolinska Institutet

Each row represents one week, i.e. one cage change cycle starting on the morning of cage change day. Vertical red and blue lines indicate time for lights-on and lights-off, respectively, in the holding room. Grey shaded area shows the lights-on part of the cage change day. Each plot has a title stating cage ID, age in days on the first day of the cycle.
Scrolling through the figure using Adobe Acrobate Reader and single-page view very clearly displays variations in activity levels over several weeks.

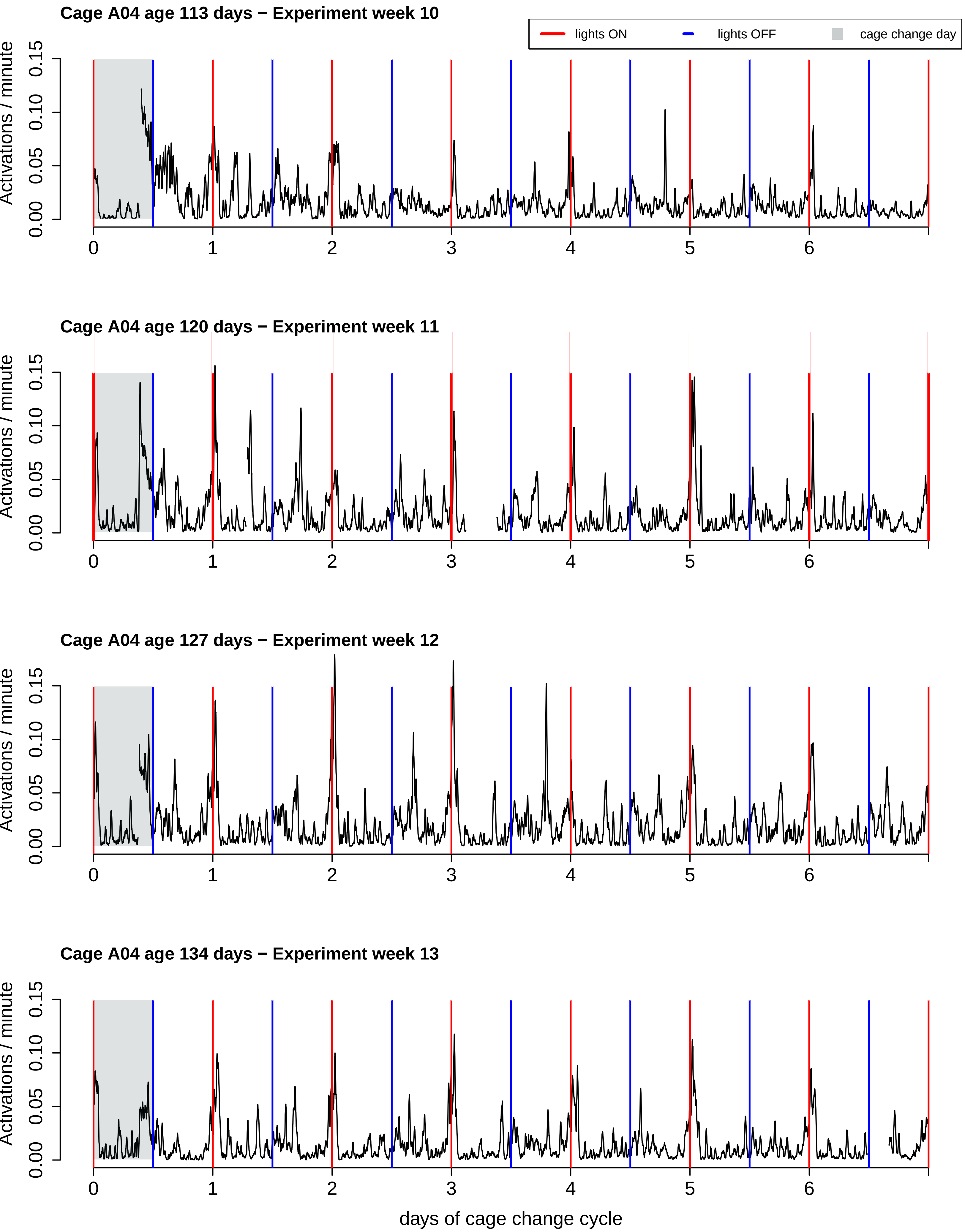

Cage A04 age 113 days − Experiment week 10
lights ON
lights OFF
cage change day
Cage A04 age 120 days − Experiment week 11
Cage A04 age 127 days − Experiment week 12
Cage A04 age 134 days − Experiment week 13
Activations / minute
0.00
0.05
0.10
0.15
0
1
2
3
4
5
6
days of cage change cycle

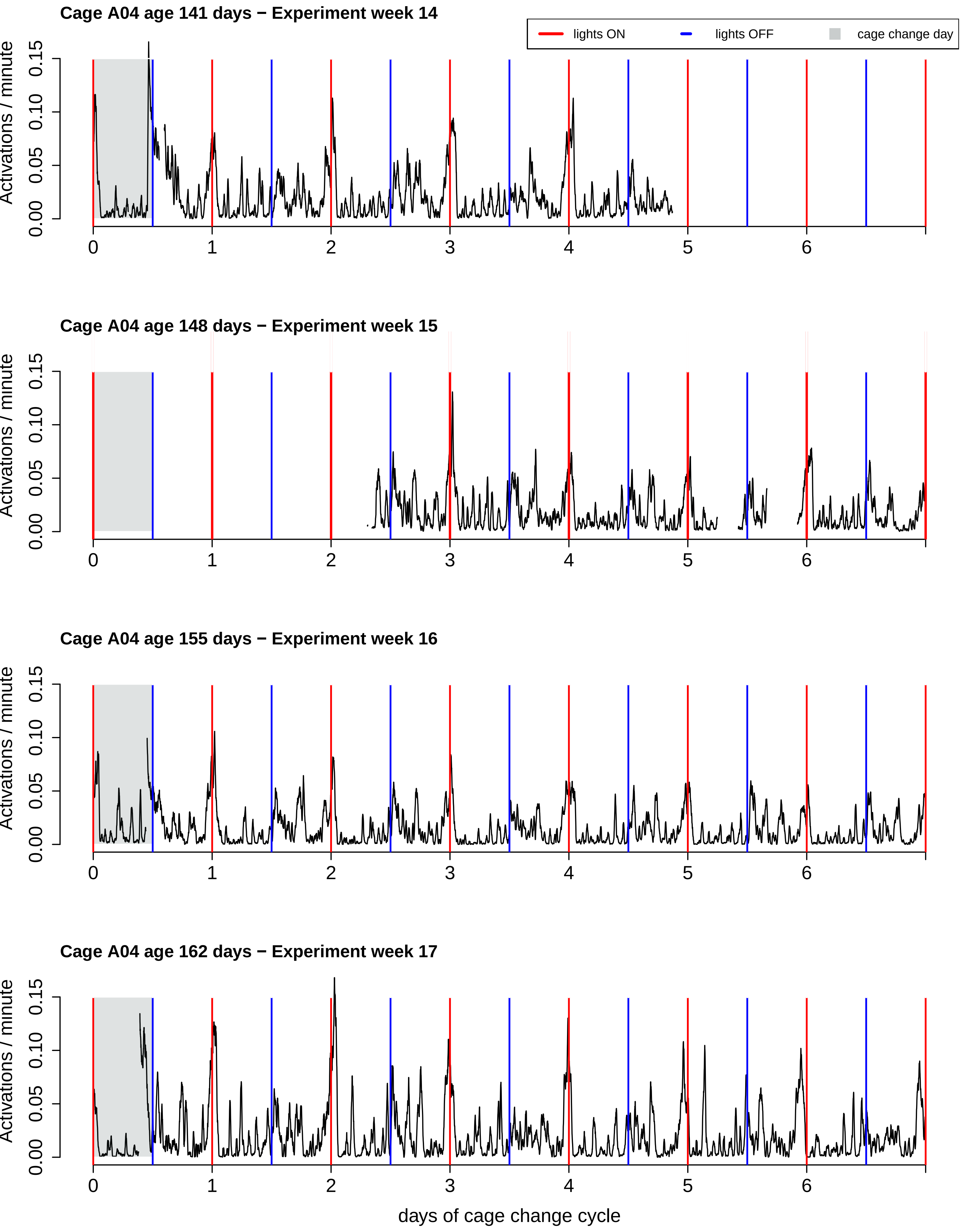

Cage A04 age 141 days − Experiment week 14
lights ON
lights OFF
cage change day
Cage A04 age 148 days − Experiment week 15
Cage A04 age 155 days − Experiment week 16
Cage A04 age 162 days − Experiment week 17
Activations / minute
0.00
0.05
0.10
0.15
0
1
2
3
4
5
6
days of cage change cycle

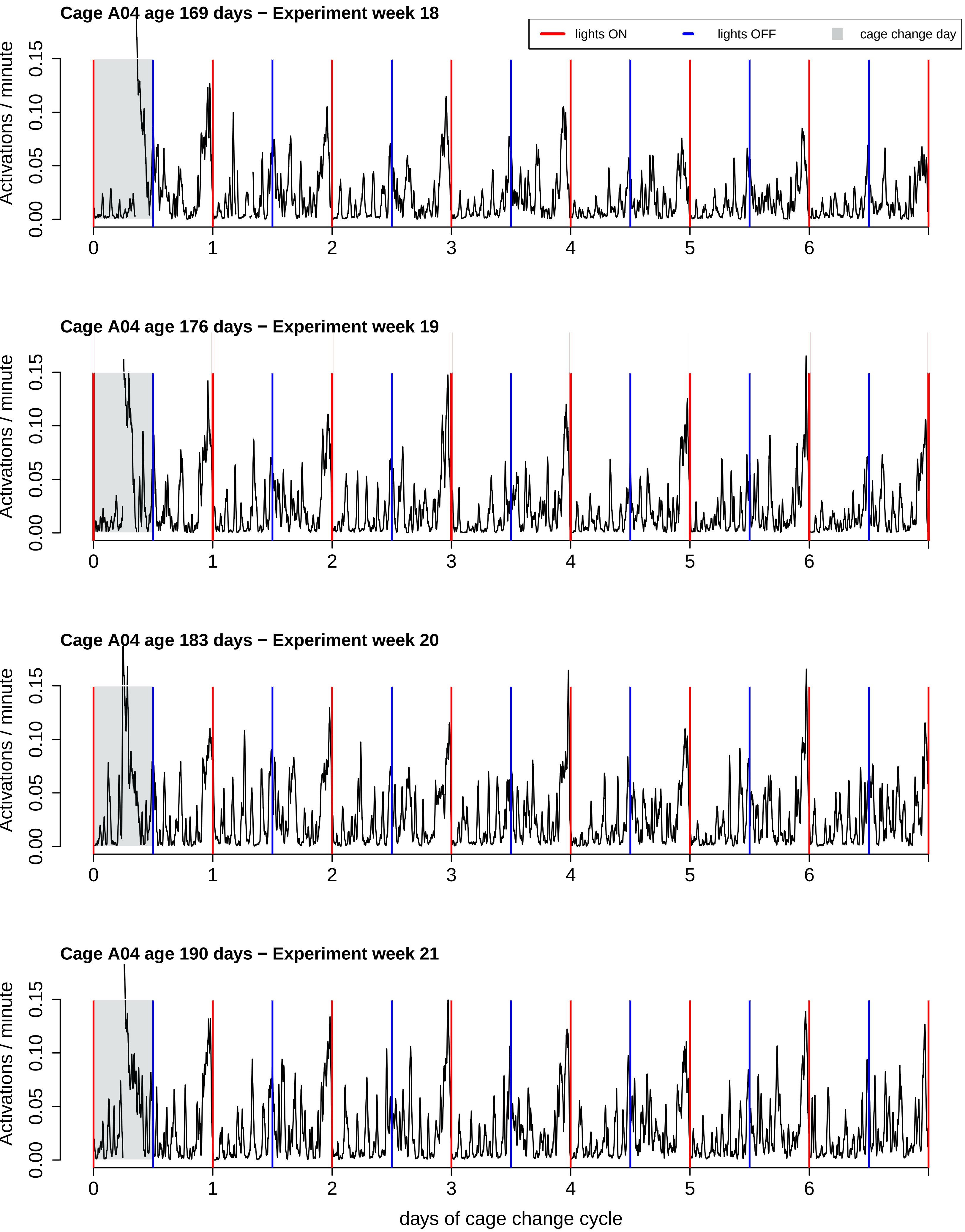
Cage A04 age 169 days − Experiment week 18
lights ON
lights OFF
cage change day
Activations / minute
0.00
0.05
0.10
0.15
0
1
2
3
4
5
6
Cage A04 age 176 days − Experiment week 19
Cage A04 age 183 days − Experiment week 20
Cage A04 age 190 days − Experiment week 21
days of cage change cycle

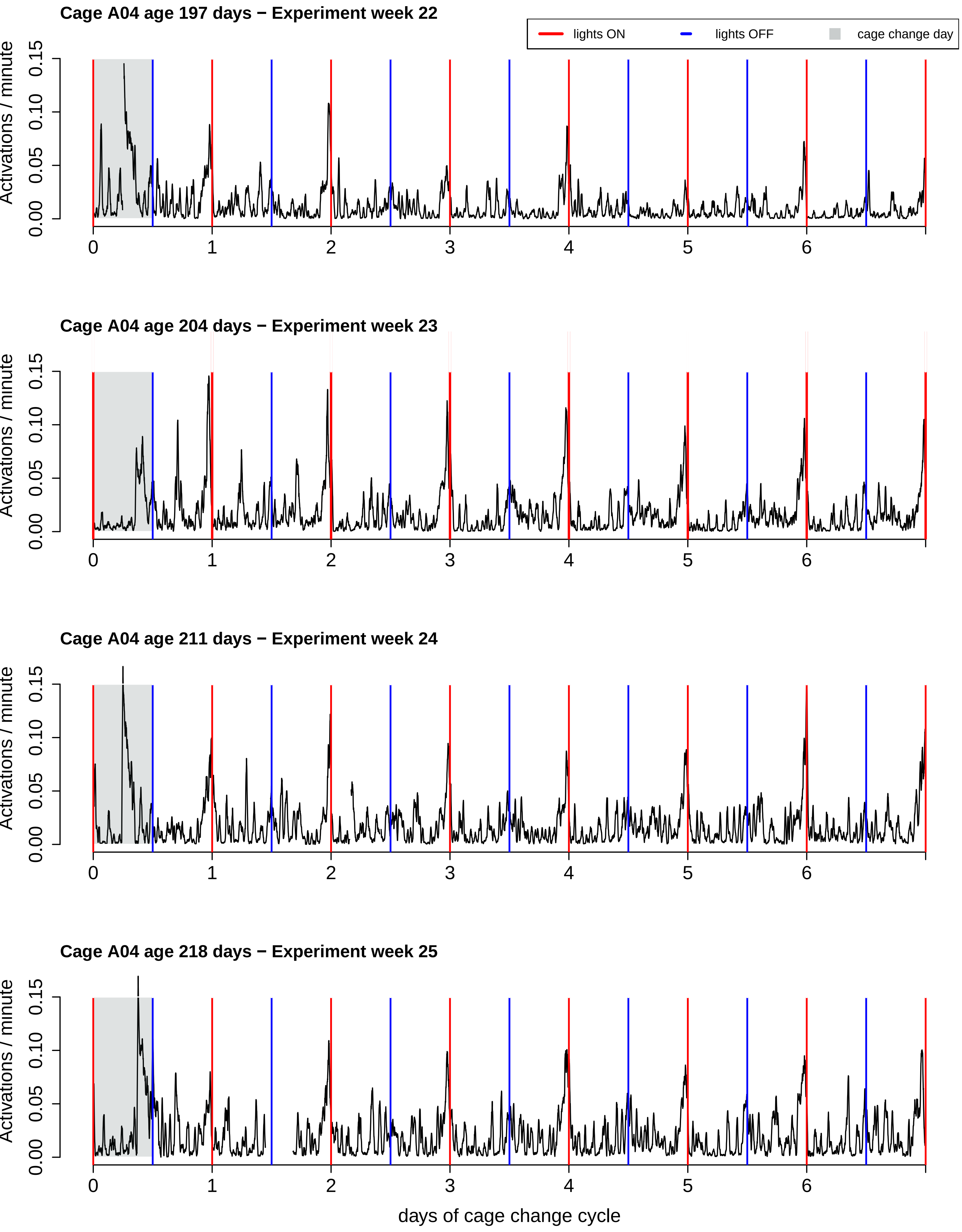
Cage A04 age 197 days − Experiment week 22
lights ON
lights OFF
cage change day
Activations / minute
0.00
0.05
0.10
0.15
0
1
2
3
4
5
6
Cage A04 age 204 days − Experiment week 23
Activations / minute
0.00
0.05
0.10
0.15
0
1
2
3
4
5
6
Cage A04 age 211 days − Experiment week 24
Activations / minute
0.00
0.05
0.10
0.15
0
1
2
3
4
5
6
Cage A04 age 218 days − Experiment week 25
Activations / minute
0.00
0.05
0.10
0.15
0
1
2
3
4
5
6
days of cage change cycle

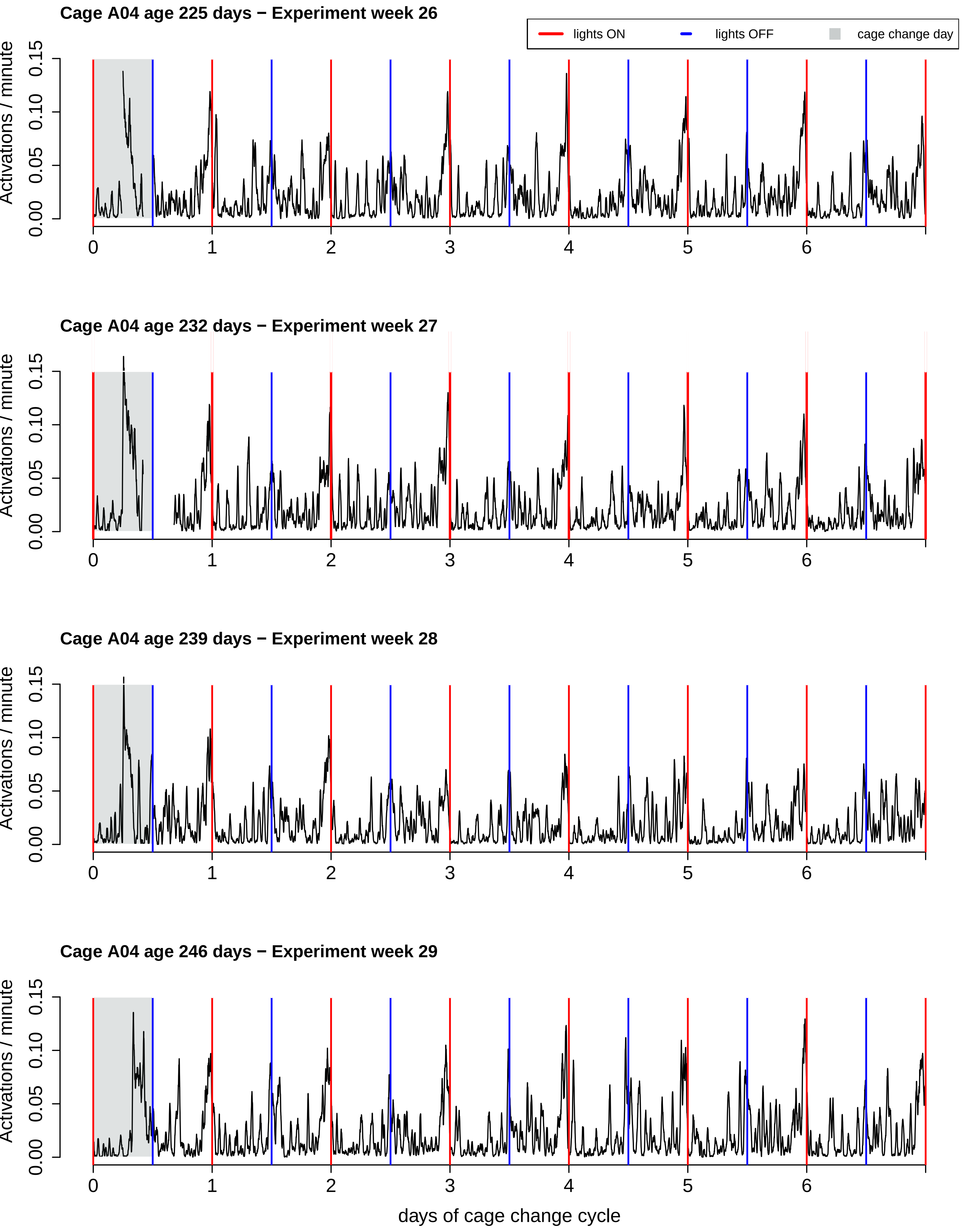

Cage A04 age 225 days − Experiment week 26
lights ON
lights OFF
cage change day
Activations / minute
0.00
0.05
0.10
0.15
0
1
2
3
4
5
6
Cage A04 age 232 days − Experiment week 27
Cage A04 age 239 days − Experiment week 28
Cage A04 age 246 days − Experiment week 29
days of cage change cycle

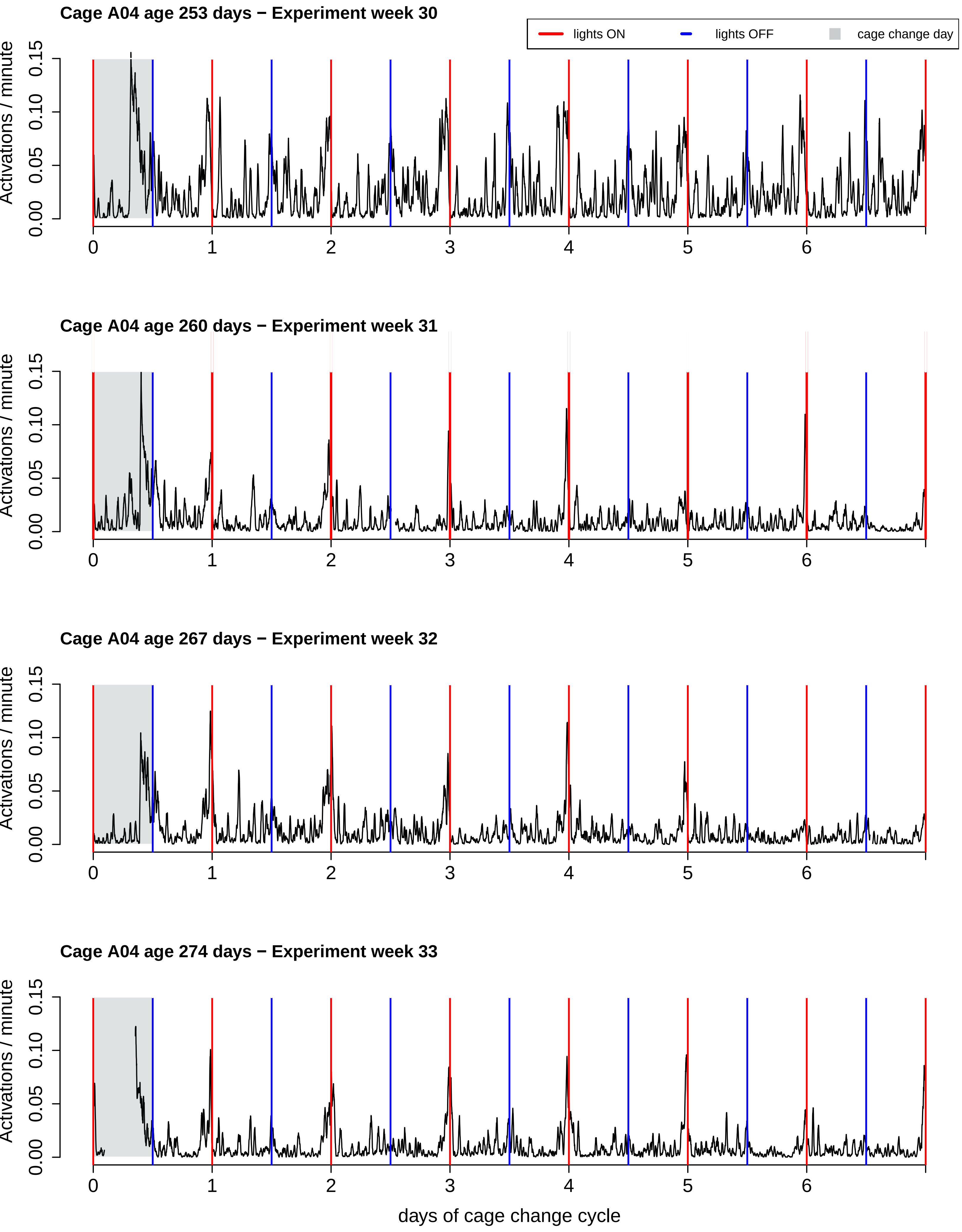

Cage A04 age 253 days − Experiment week 30
lights ON
lights OFF
cage change day
Activations / minute
0.00
0.05
0.10
0.15
0
1
2
3
4
5
6
Cage A04 age 260 days − Experiment week 31
Cage A04 age 267 days − Experiment week 32
Cage A04 age 274 days − Experiment week 33
days of cage change cycle

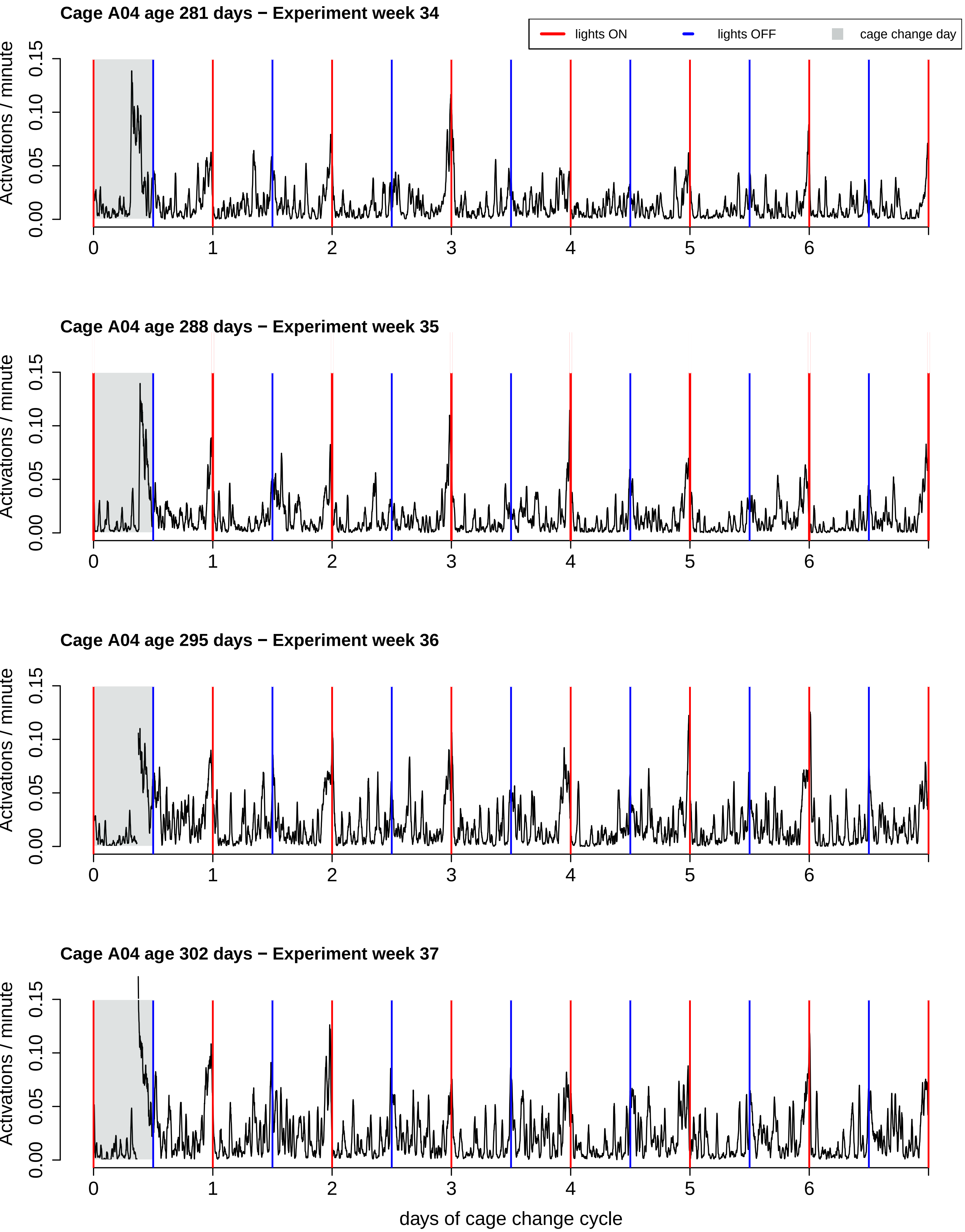
Cage A04 age 281 days − Experiment week 34
lights ON
lights OFF
cage change day
Activations / minute
0.00
0.05
0.10
0.15
0
1
2
3
4
5
6
Cage A04 age 288 days − Experiment week 35
Cage A04 age 295 days − Experiment week 36
Cage A04 age 302 days − Experiment week 37
days of cage change cycle

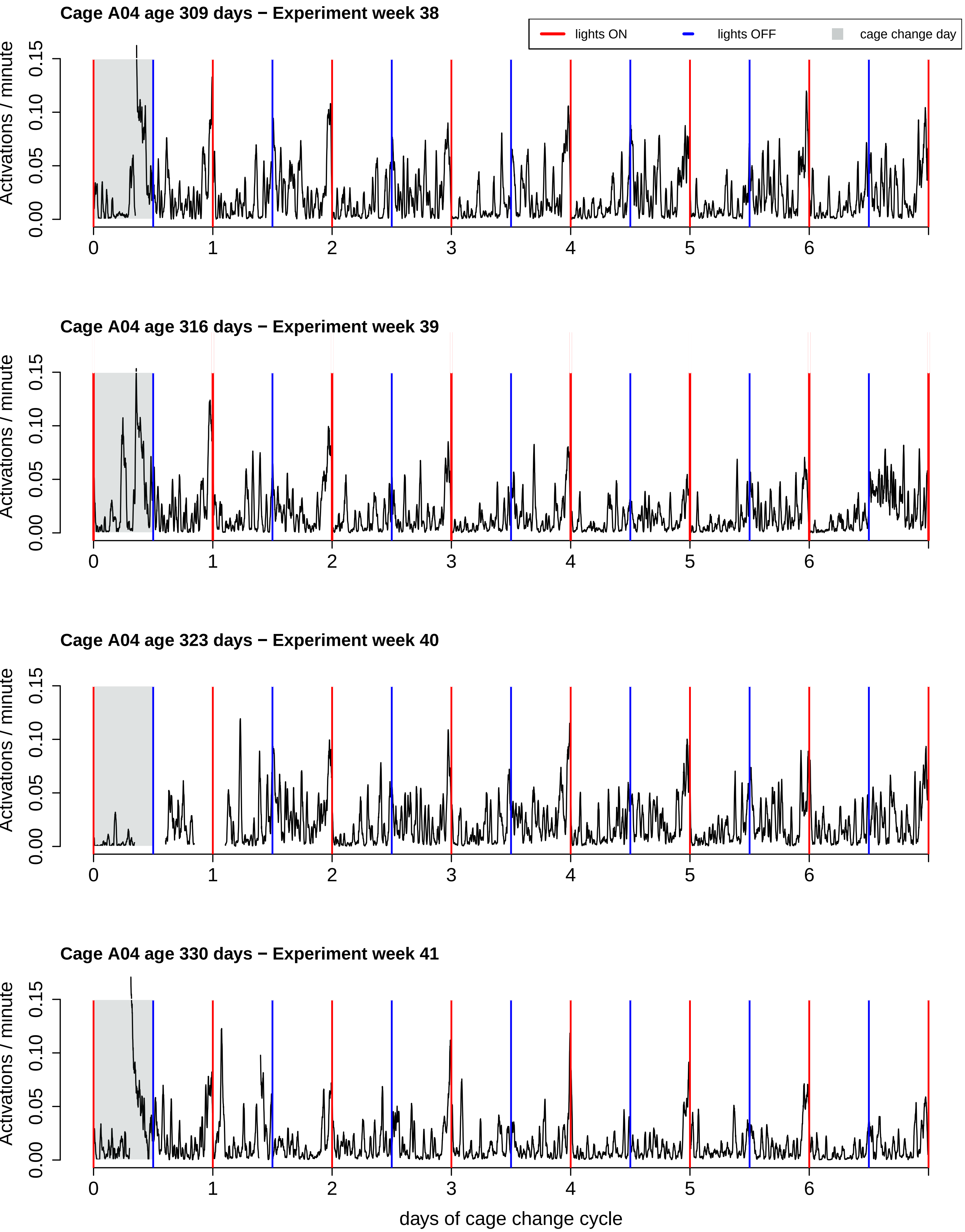
Cage A04 age 309 days − Experiment week 38
lights ON
lights OFF
cage change day
Activations / minute
0.00
0.05
0.10
0.15
0
1
2
3
4
5
6
Cage A04 age 316 days − Experiment week 39
Activations / minute
0.00
0.05
0.10
0.15
0
1
2
3
4
5
6
Cage A04 age 323 days − Experiment week 40
Activations / minute
0.00
0.05
0.10
0.15
0
1
2
3
4
5
6
Cage A04 age 330 days − Experiment week 41
Activations / minute
0.00
0.05
0.10
0.15
0
1
2
3
4
5
6
days of cage change cycle

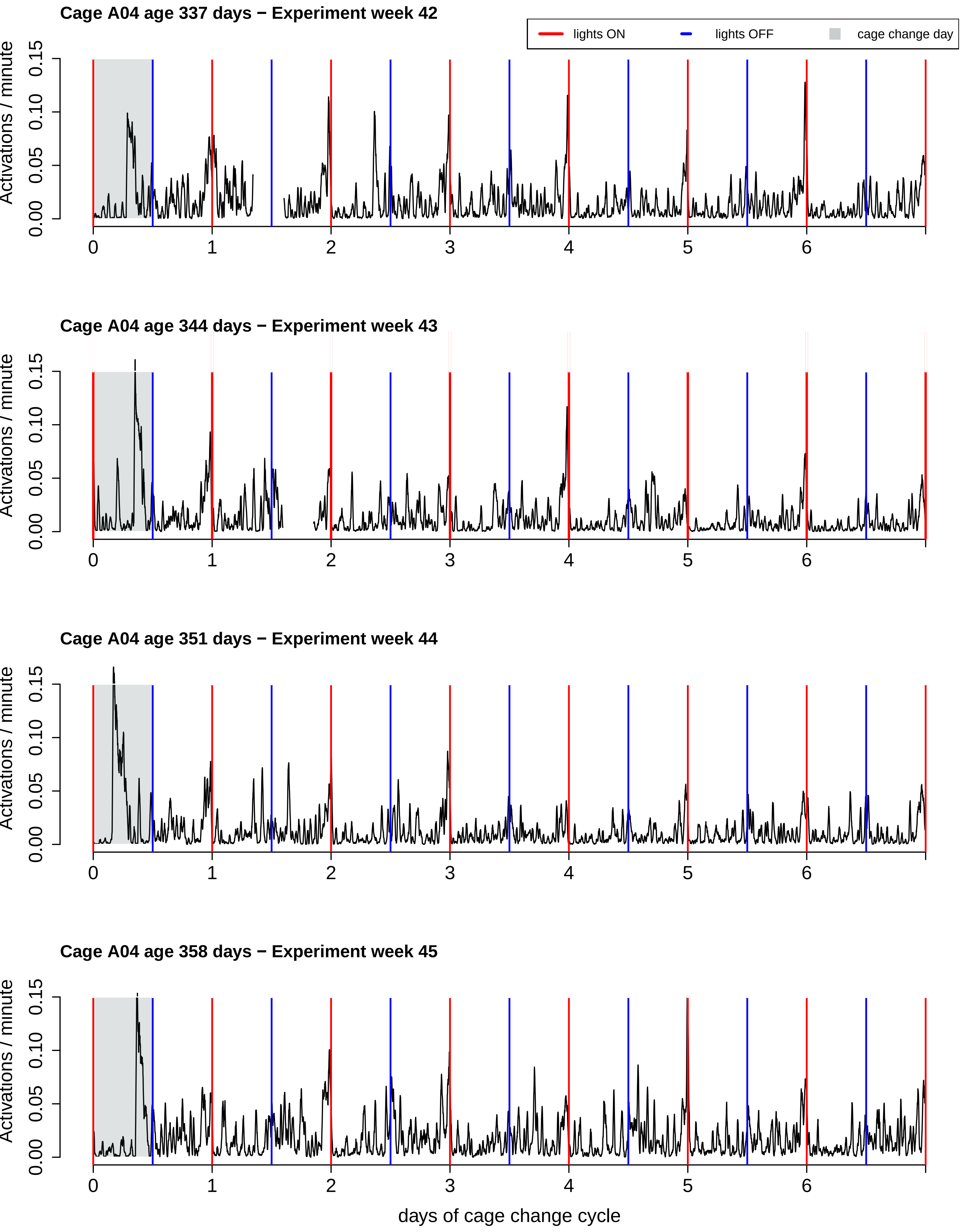
Cage A04 age 337 days − Experiment week 42
lights ON
lights OFF
cage change day
Activations / minute
0.00
0.05
0.10
0.15
0
1
2
3
4
5
6
Cage A04 age 344 days − Experiment week 43
Cage A04 age 351 days − Experiment week 44
Cage A04 age 358 days − Experiment week 45
days of cage change cycle

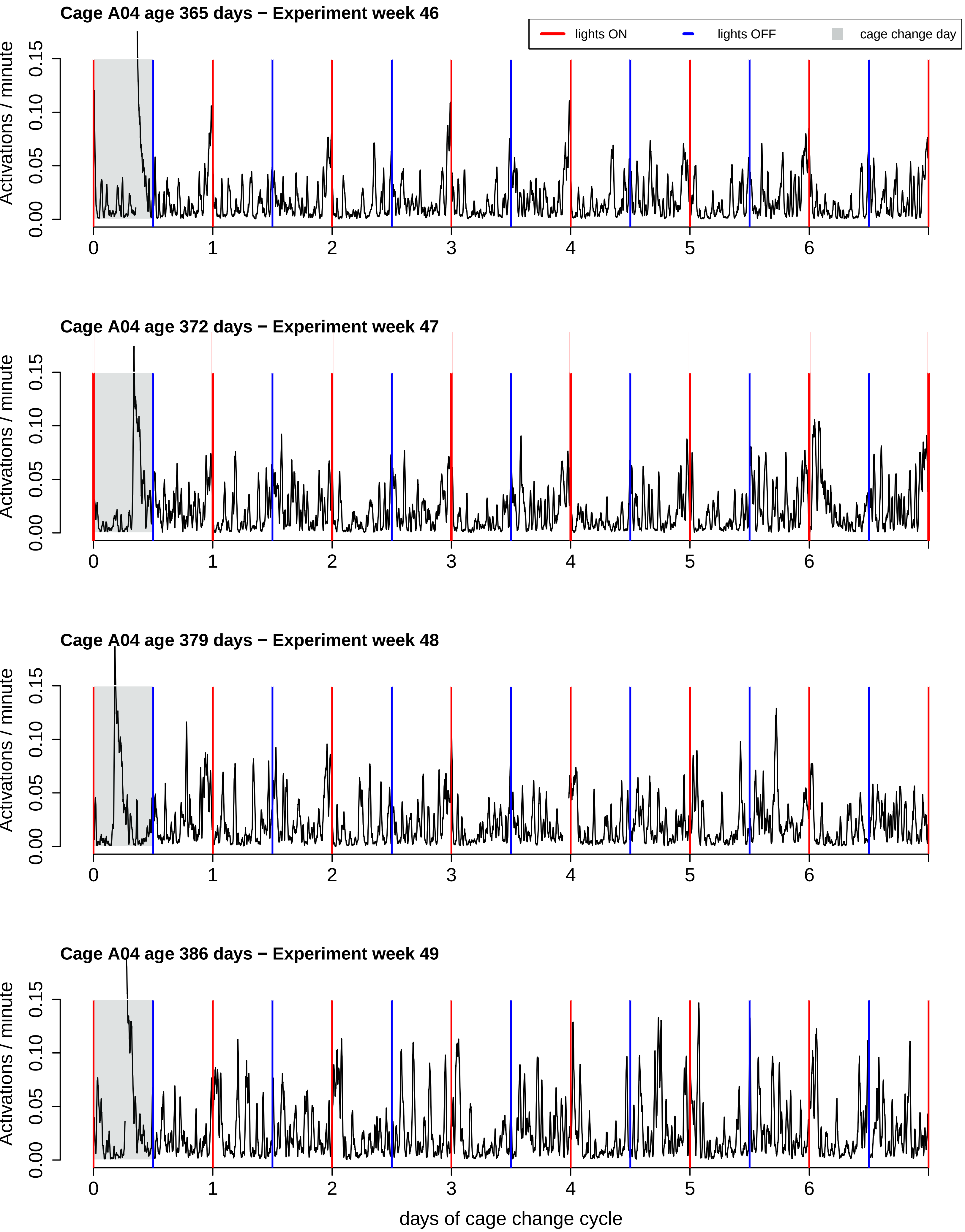
Cage A04 age 365 days − Experiment week 46
lights ON
lights OFF
cage change day
Activations / minute
0.00
0.05
0.10
0.15
0
1
2
3
4
5
6
Cage A04 age 372 days − Experiment week 47
Cage A04 age 379 days − Experiment week 48
Cage A04 age 386 days − Experiment week 49
days of cage change cycle

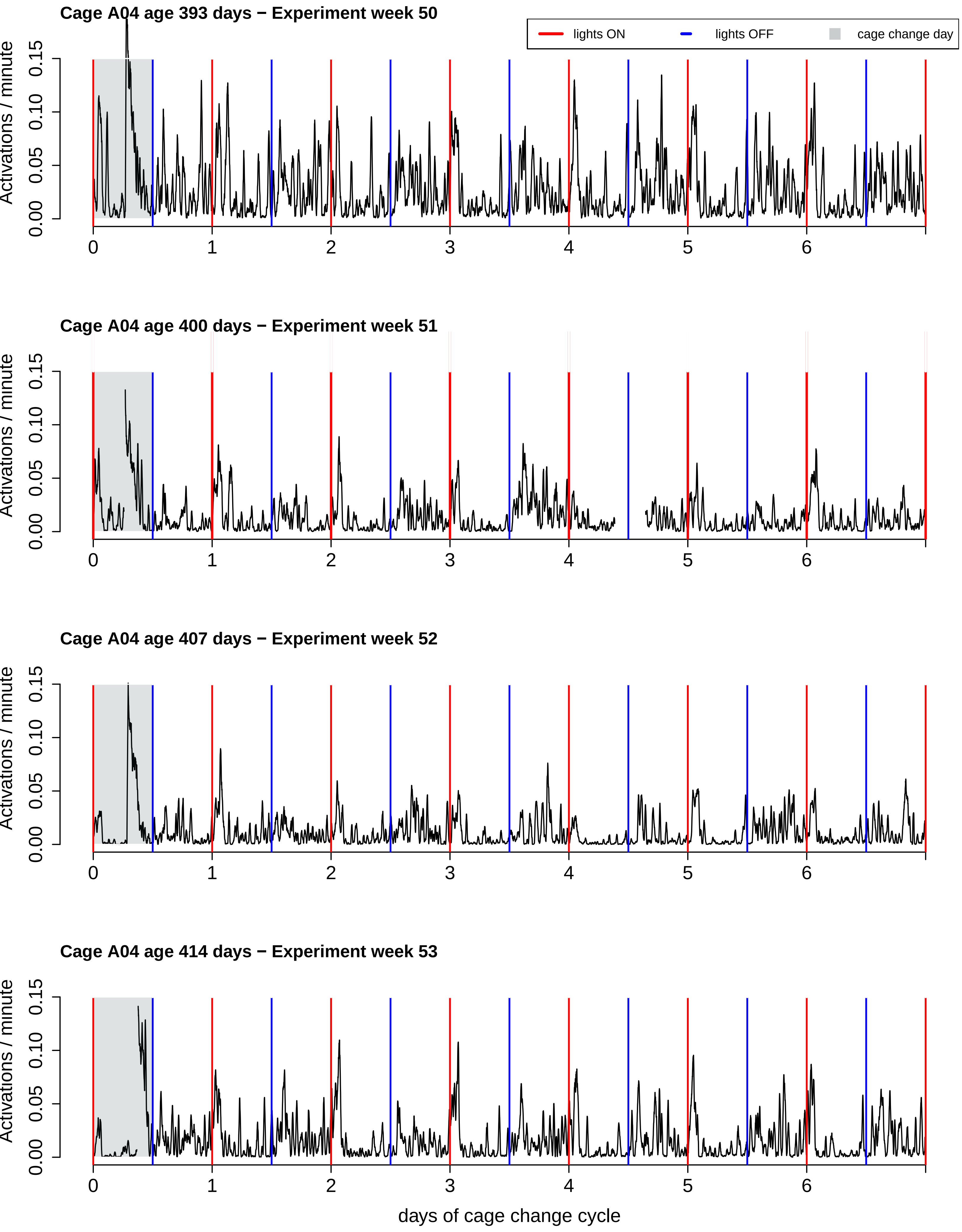
Cage A04 age 393 days − Experiment week 50
lights ON
lights OFF
cage change day
Activations / minute
0.00
0.05
0.10
0.15
0
1
2
3
4
5
6
Cage A04 age 400 days − Experiment week 51
Cage A04 age 407 days − Experiment week 52
Cage A04 age 414 days − Experiment week 53
days of cage change cycle

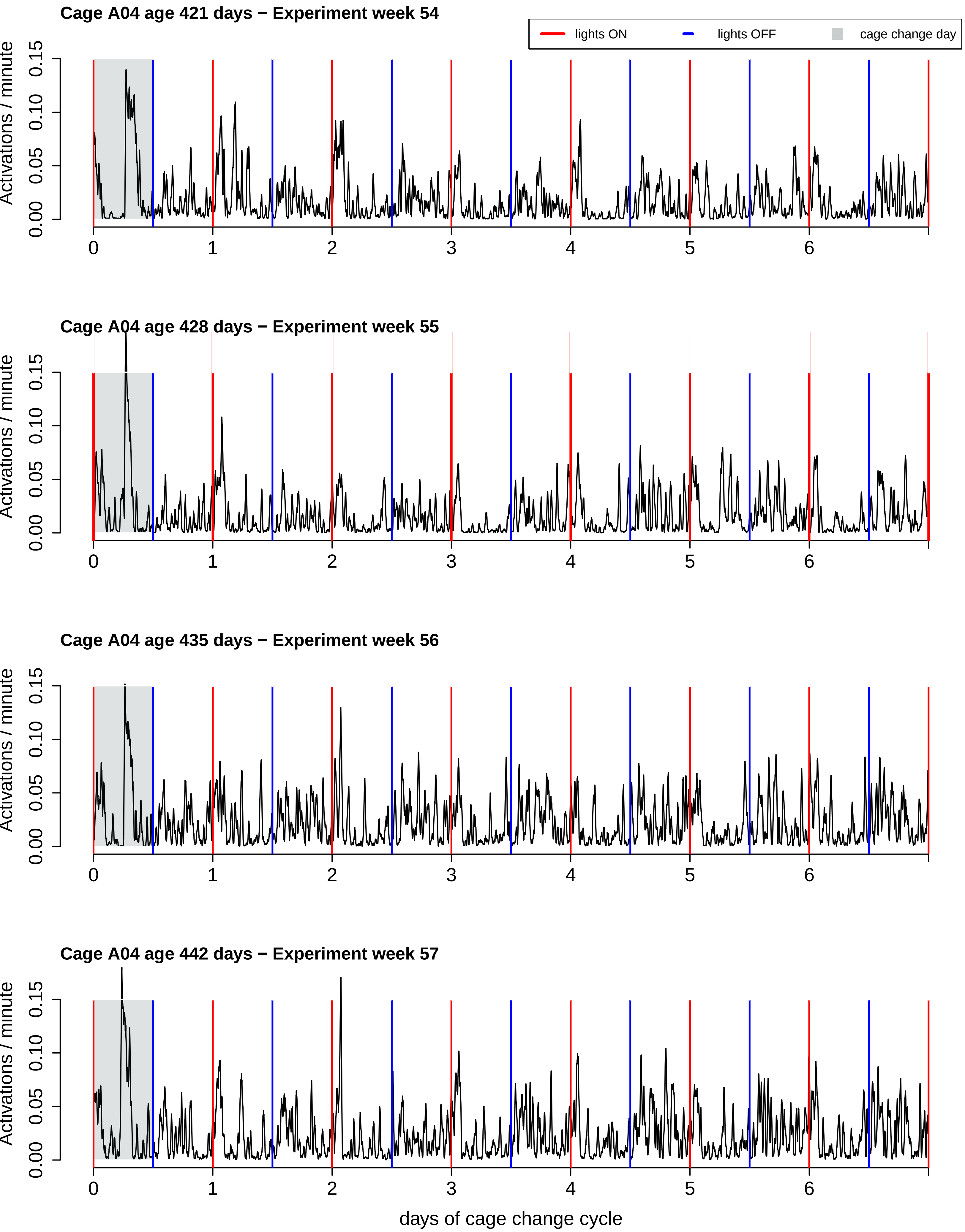
Cage A04 age 421 days − Experiment week 54
lights ON
lights OFF
cage change day
Activations / minute
0.00
0.05
0.10
0.15
0
1
2
3
4
5
6
Cage A04 age 428 days − Experiment week 55
Cage A04 age 435 days − Experiment week 56
Cage A04 age 442 days − Experiment week 57
days of cage change cycle

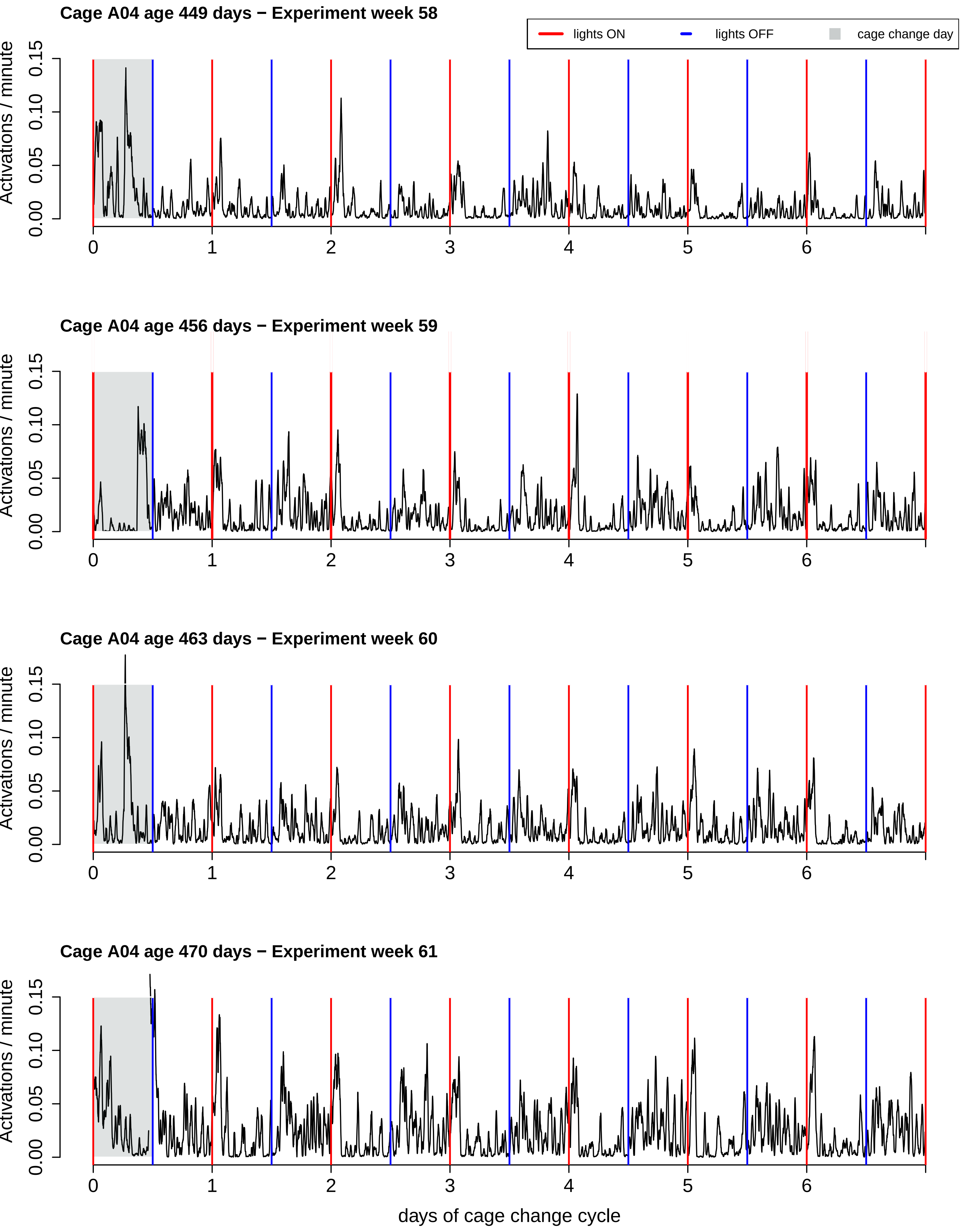

Cage A04 age 449 days − Experiment week 58
lights ON
lights OFF
cage change day
Activations / minute
0.00
0.05
0.10
0.15
0
1
2
3
4
5
6
Cage A04 age 456 days − Experiment week 59
Cage A04 age 463 days − Experiment week 60
Cage A04 age 470 days − Experiment week 61
days of cage change cycle

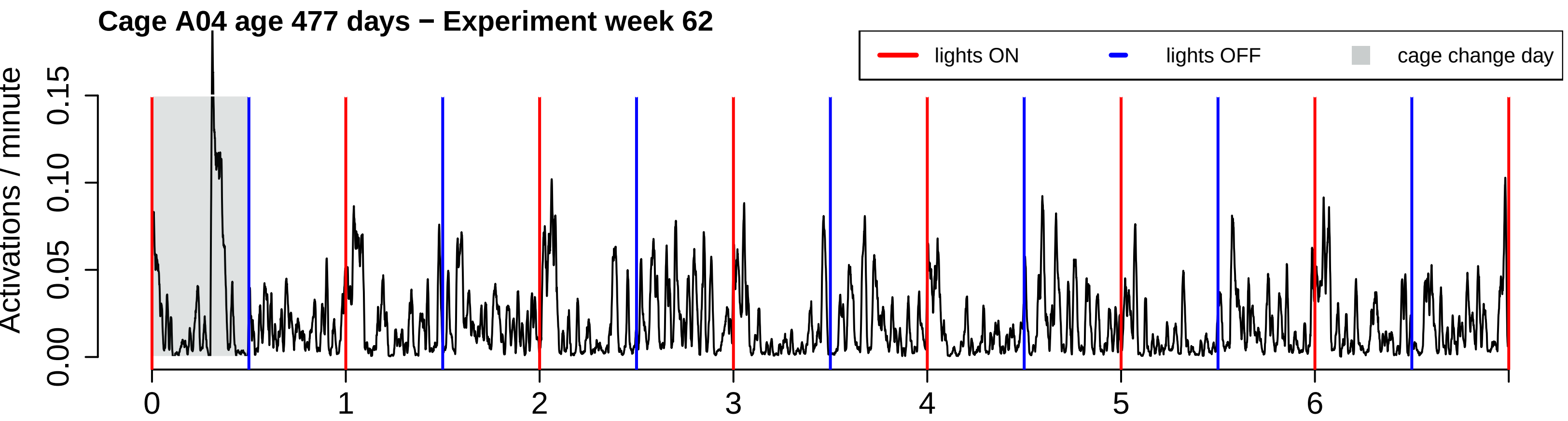

Cage A04 age 477 days − Experiment week 62
lights ON
lights OFF
cage change day
Activations / minute
0.00
0.05
0.10
0.15
0
1
2
3
4
5
6

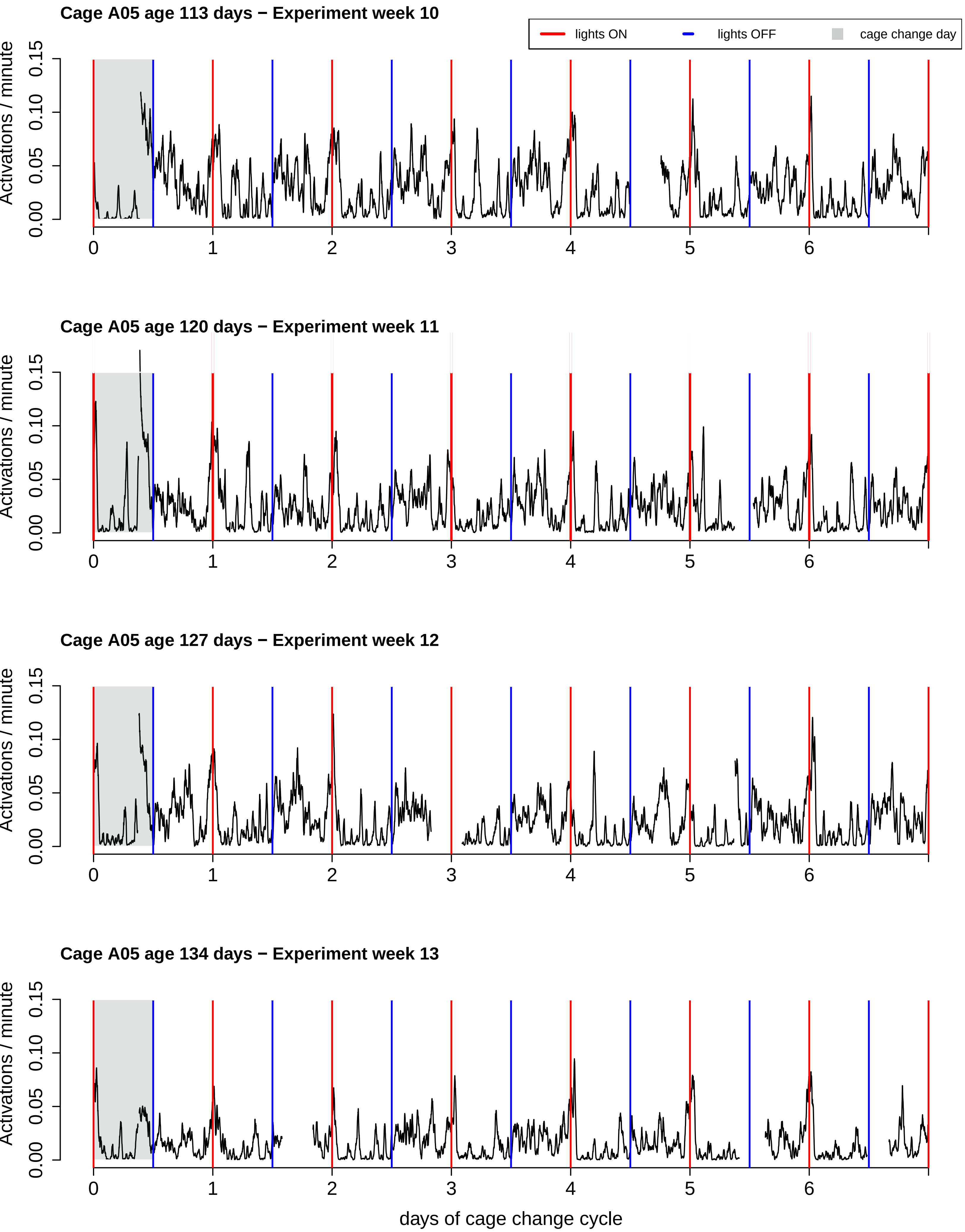
Cage A05 age 113 days − Experiment week 10
lights ON
lights OFF
cage change day
Activations / minute
0.00
0.05
0.10
0.15
0
1
2
3
4
5
6
Cage A05 age 120 days − Experiment week 11
Cage A05 age 127 days − Experiment week 12
Cage A05 age 134 days − Experiment week 13
days of cage change cycle

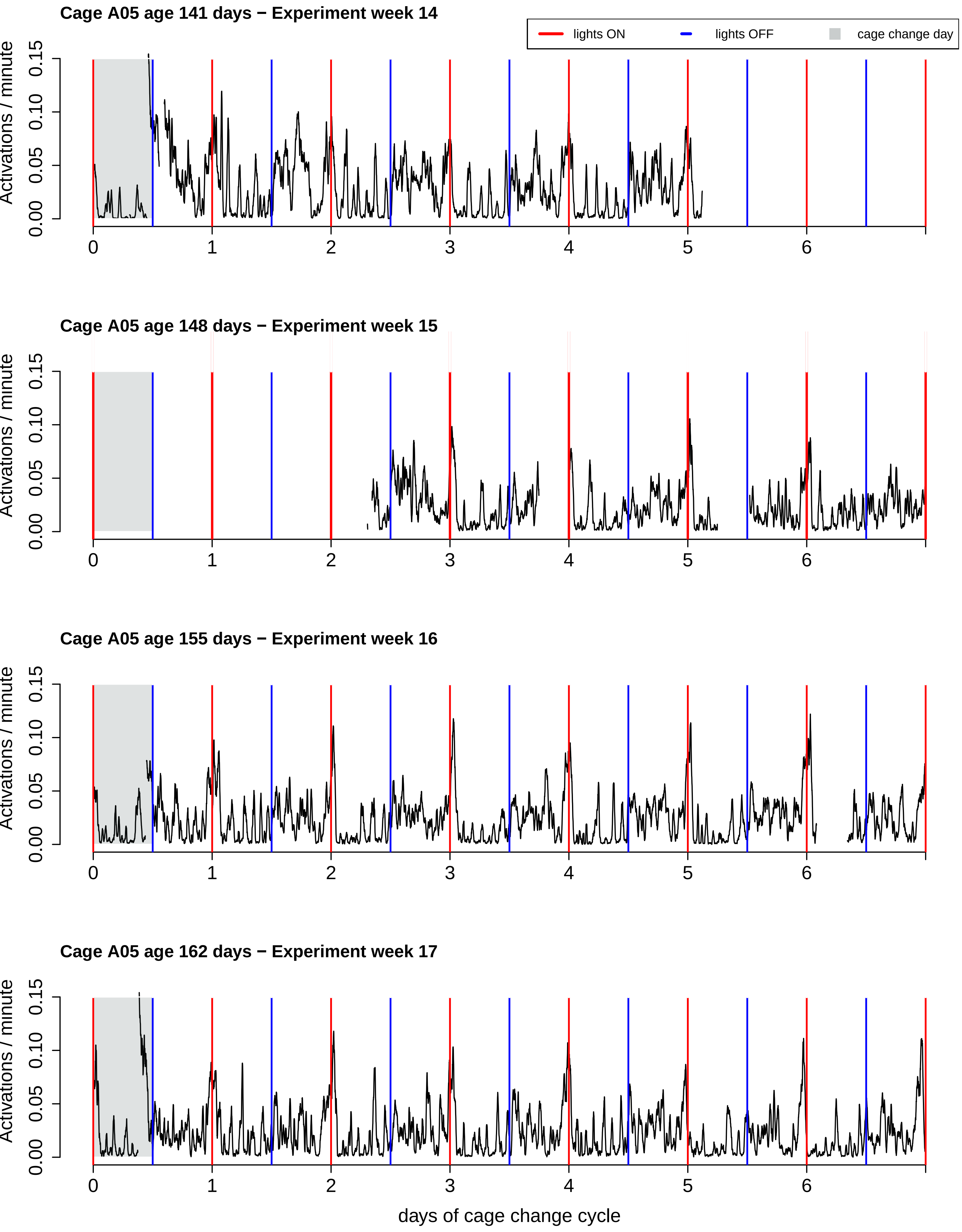
Cage A05 age 141 days − Experiment week 14
lights ON
lights OFF
cage change day
Activations / minute
0.00
0.05
0.10
0.15
0
1
2
3
4
5
6
Cage A05 age 148 days − Experiment week 15
Cage A05 age 155 days − Experiment week 16
Cage A05 age 162 days − Experiment week 17
days of cage change cycle

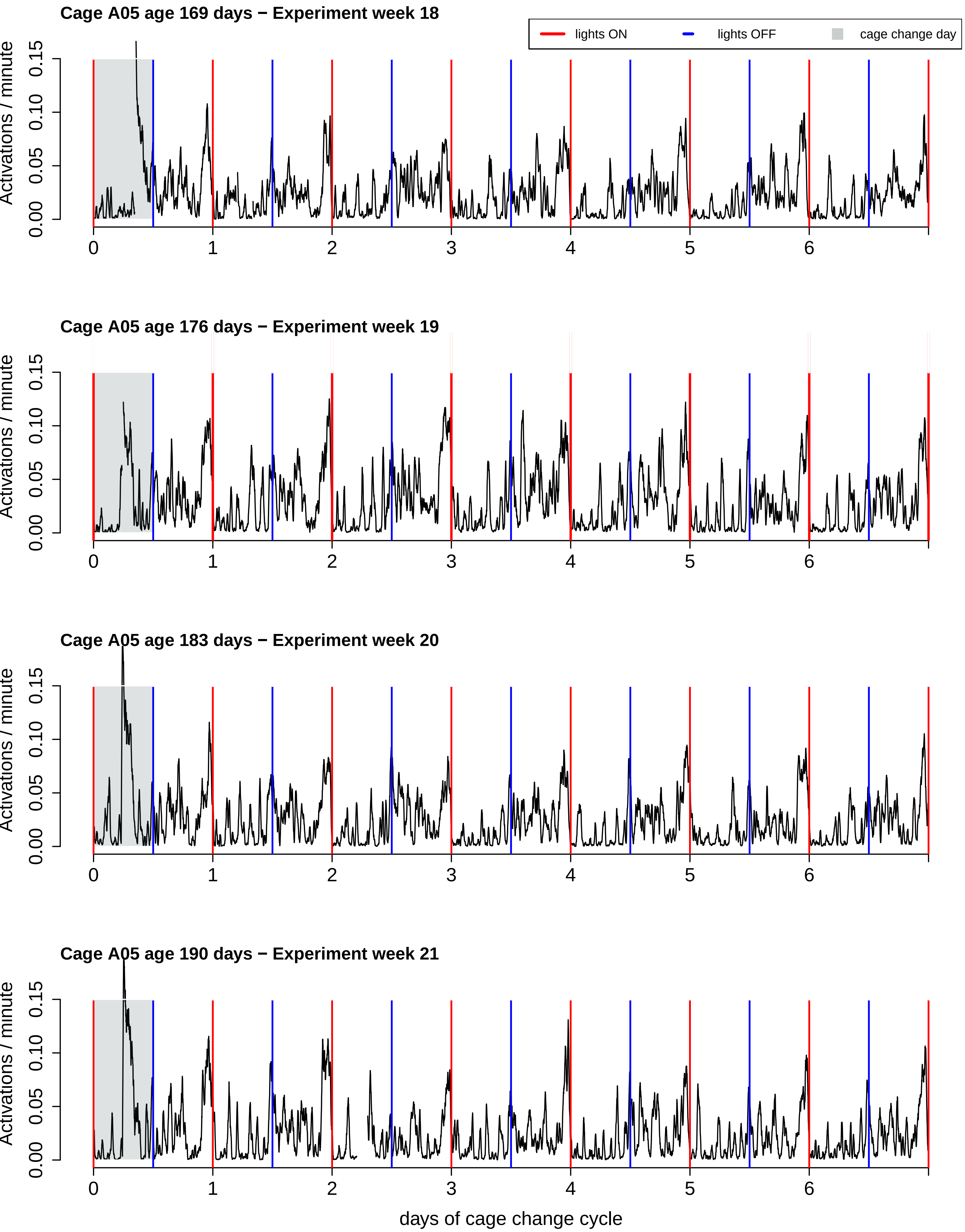
Cage A05 age 169 days − Experiment week 18
lights ON
lights OFF
cage change day
Activations / minute
0.00
0.05
0.10
0.15
0
1
2
3
4
5
6
Cage A05 age 176 days − Experiment week 19
Cage A05 age 183 days − Experiment week 20
Cage A05 age 190 days − Experiment week 21
days of cage change cycle

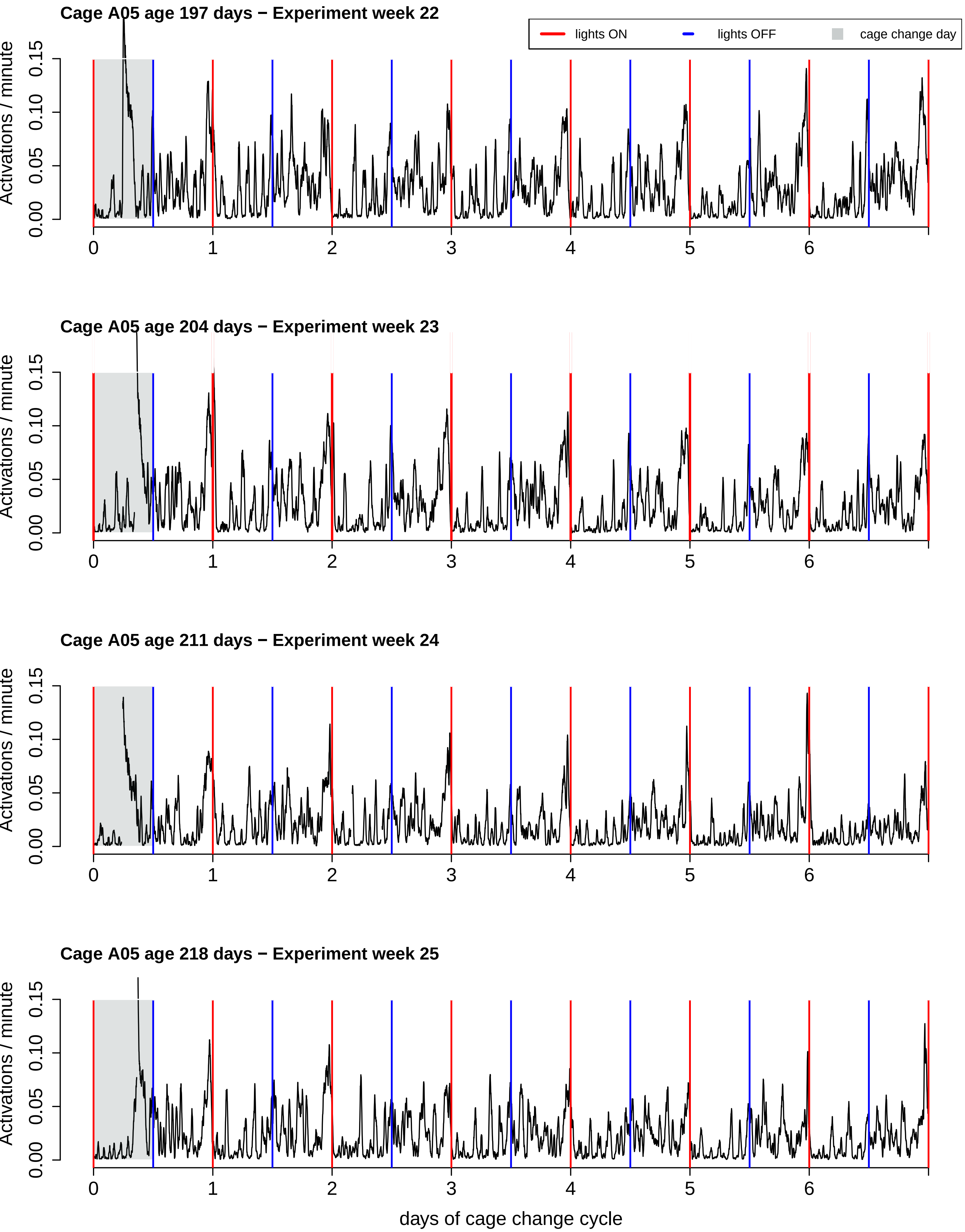
Cage A05 age 197 days − Experiment week 22
lights ON
lights OFF
cage change day
Activations / minute
0.00
0.05
0.10
0.15
0
1
2
3
4
5
6
Cage A05 age 204 days − Experiment week 23
Activations / minute
Cage A05 age 211 days − Experiment week 24
Activations / minute
Cage A05 age 218 days − Experiment week 25
Activations / minute
days of cage change cycle

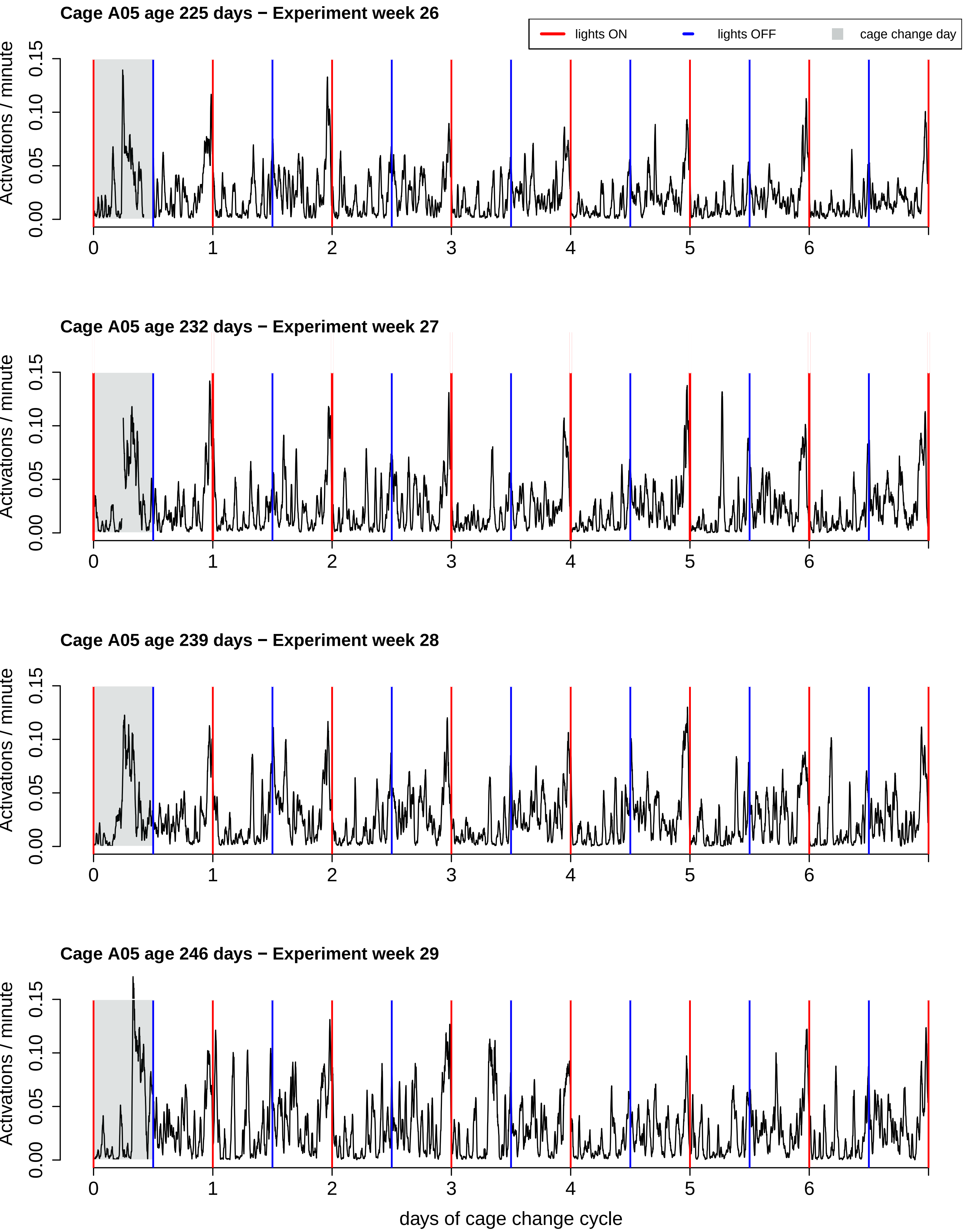

Cage A05 age 225 days − Experiment week 26
lights ON
lights OFF
cage change day
Activations / minute
0.00
0.05
0.10
0.15
0
1
2
3
4
5
6
Cage A05 age 232 days − Experiment week 27
Cage A05 age 239 days − Experiment week 28
Cage A05 age 246 days − Experiment week 29
days of cage change cycle

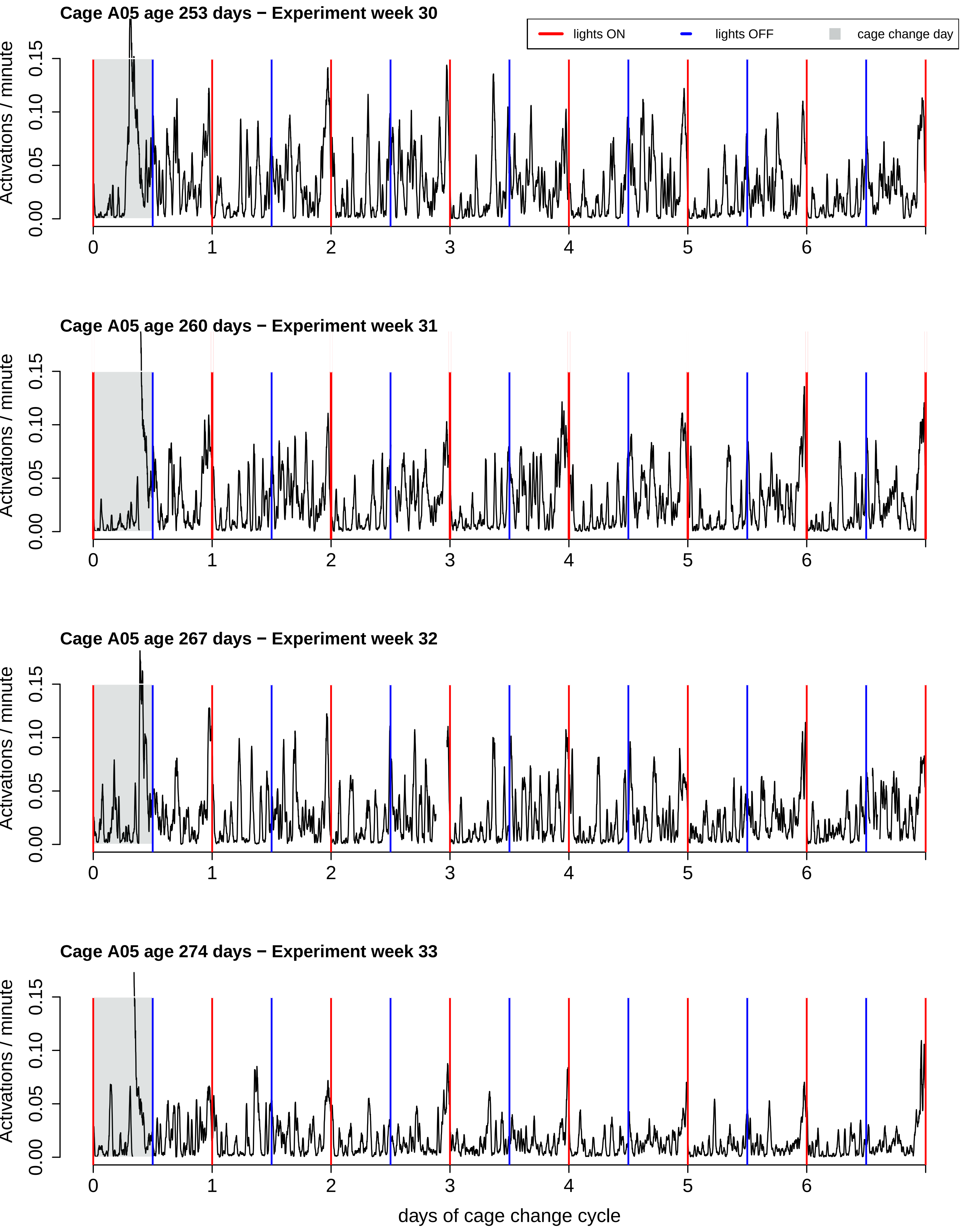
Cage A05 age 253 days − Experiment week 30
lights ON
lights OFF
cage change day
Activations / minute
0.00
0.05
0.10
0.15
0
1
2
3
4
5
6
Cage A05 age 260 days − Experiment week 31
Cage A05 age 267 days − Experiment week 32
Cage A05 age 274 days − Experiment week 33
days of cage change cycle

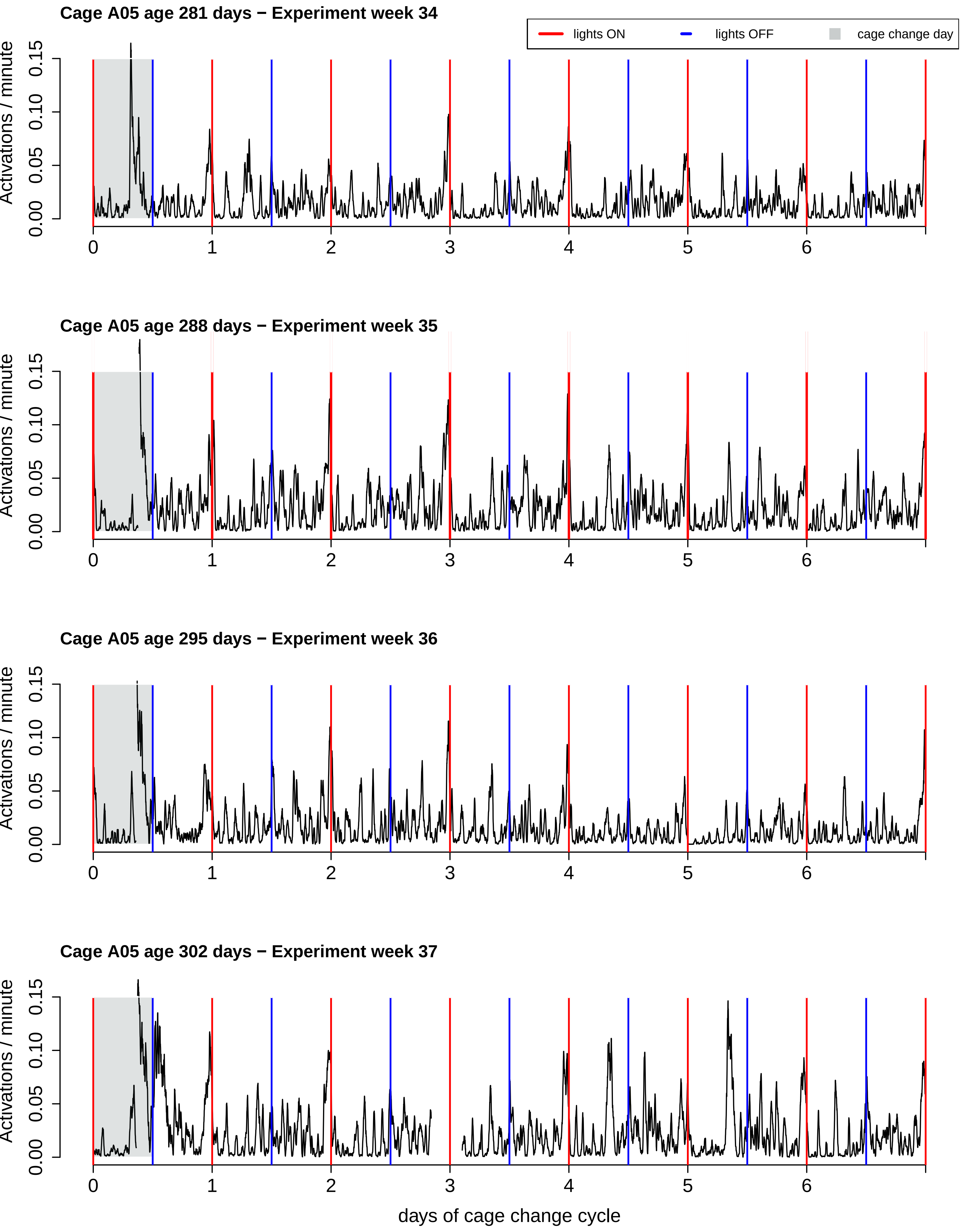
Cage A05 age 281 days − Experiment week 34
lights ON
lights OFF
cage change day
Activations / minute
0.00
0.05
0.10
0.15
0
1
2
3
4
5
6
Cage A05 age 288 days − Experiment week 35
Cage A05 age 295 days − Experiment week 36
Cage A05 age 302 days − Experiment week 37
days of cage change cycle

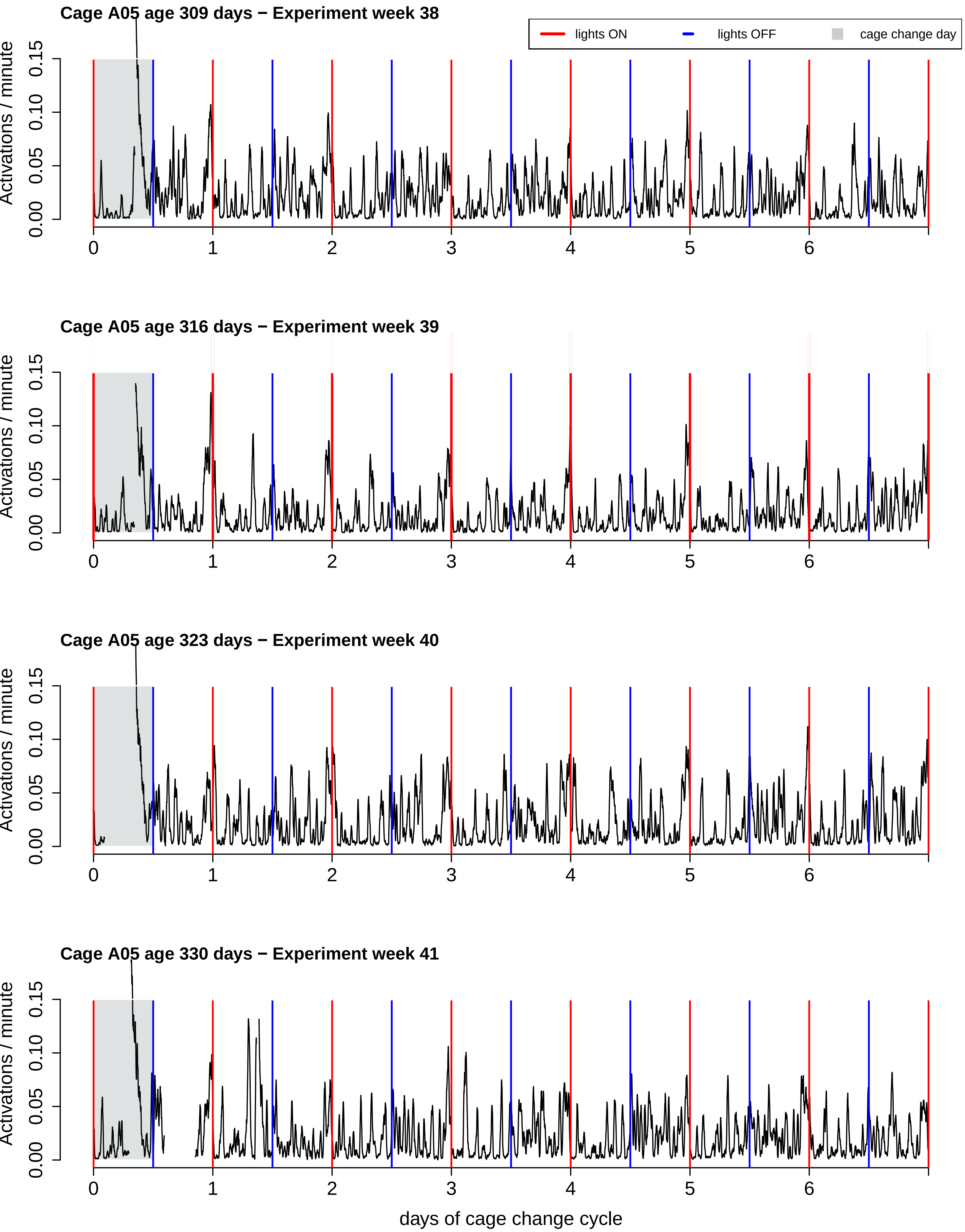
Cage A05 age 309 days − Experiment week 38
Cage A05 age 316 days − Experiment week 39
Cage A05 age 323 days − Experiment week 40
Cage A05 age 330 days − Experiment week 41
lights ON
lights OFF
cage change day
Activations / minute
0.00
0.05
0.10
0.15
0
1
2
3
4
5
6
days of cage change cycle

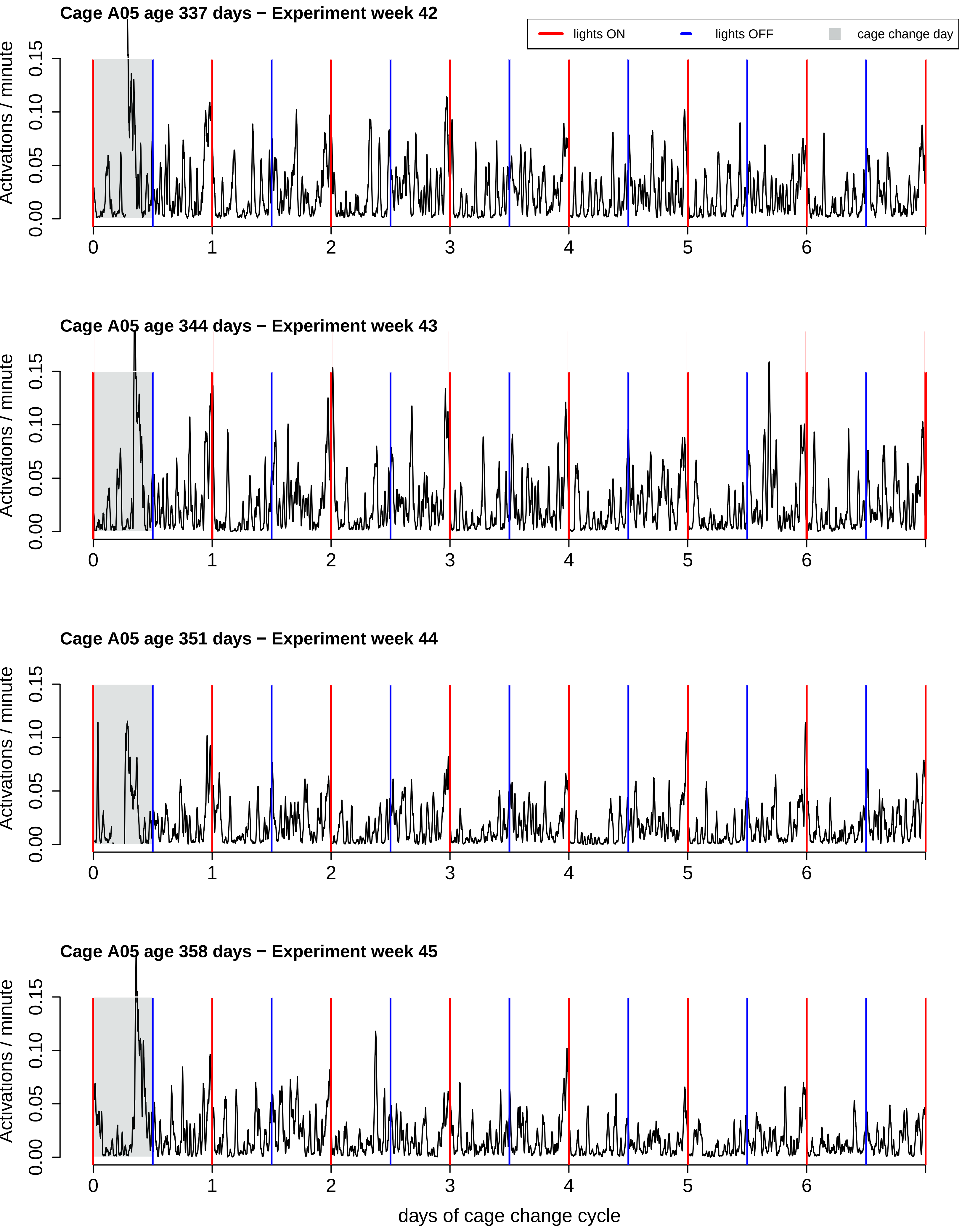

Cage A05 age 337 days − Experiment week 42
lights ON
lights OFF
cage change day
Activations / minute
0.00
0.05
0.10
0.15
0
1
2
3
4
5
6
Cage A05 age 344 days − Experiment week 43
Activations / minute
0.00
0.05
0.10
0.15
0
1
2
3
4
5
6
Cage A05 age 351 days − Experiment week 44
Activations / minute
0.00
0.05
0.10
0.15
0
1
2
3
4
5
6
Cage A05 age 358 days − Experiment week 45
Activations / minute
0.00
0.05
0.10
0.15
0
1
2
3
4
5
6
days of cage change cycle

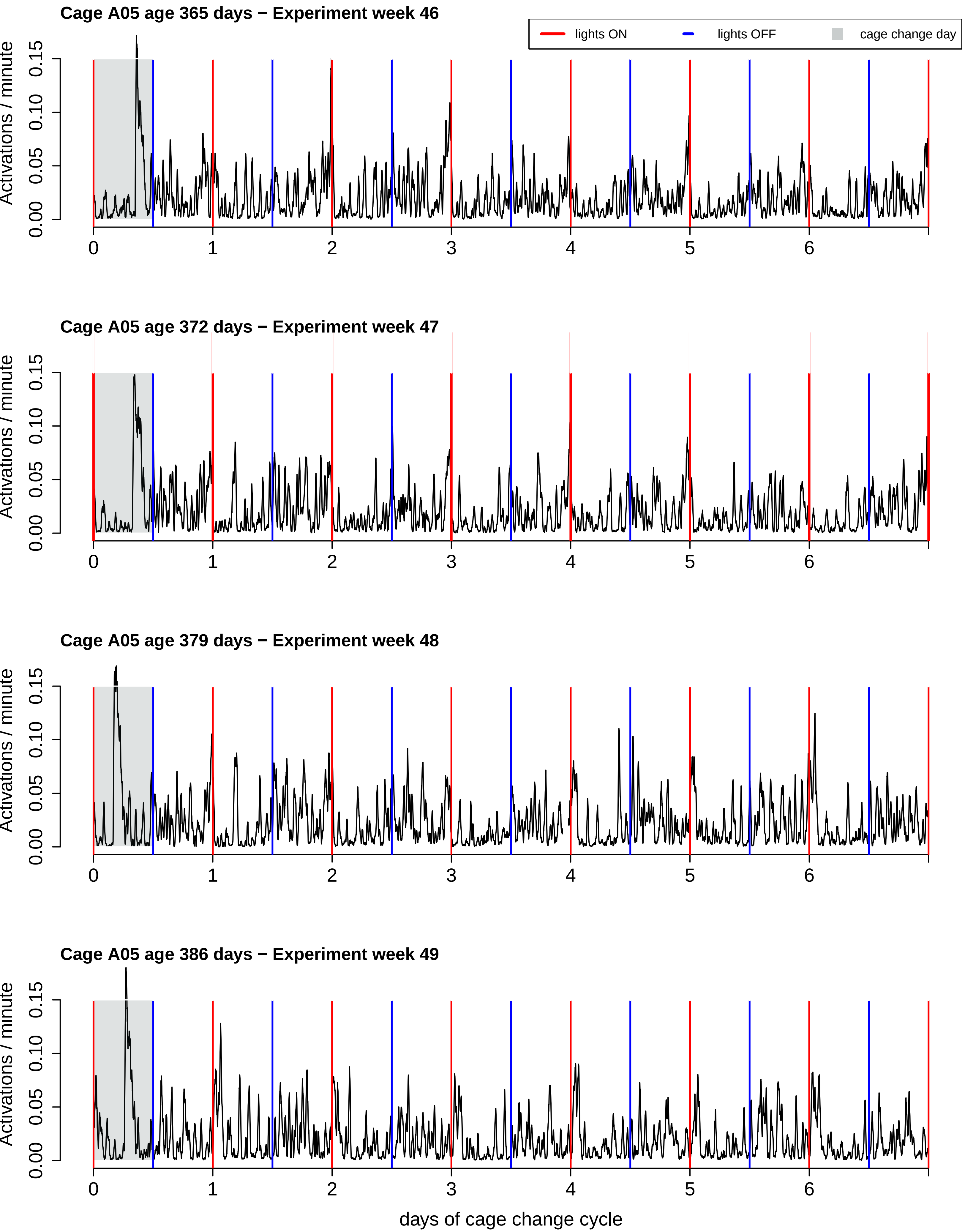

Cage A05 age 365 days − Experiment week 46
lights ON
lights OFF
cage change day
Activations / minute
0.00
0.05
0.10
0.15
0
1
2
3
4
5
6
Cage A05 age 372 days − Experiment week 47
Cage A05 age 379 days − Experiment week 48
Cage A05 age 386 days − Experiment week 49
days of cage change cycle

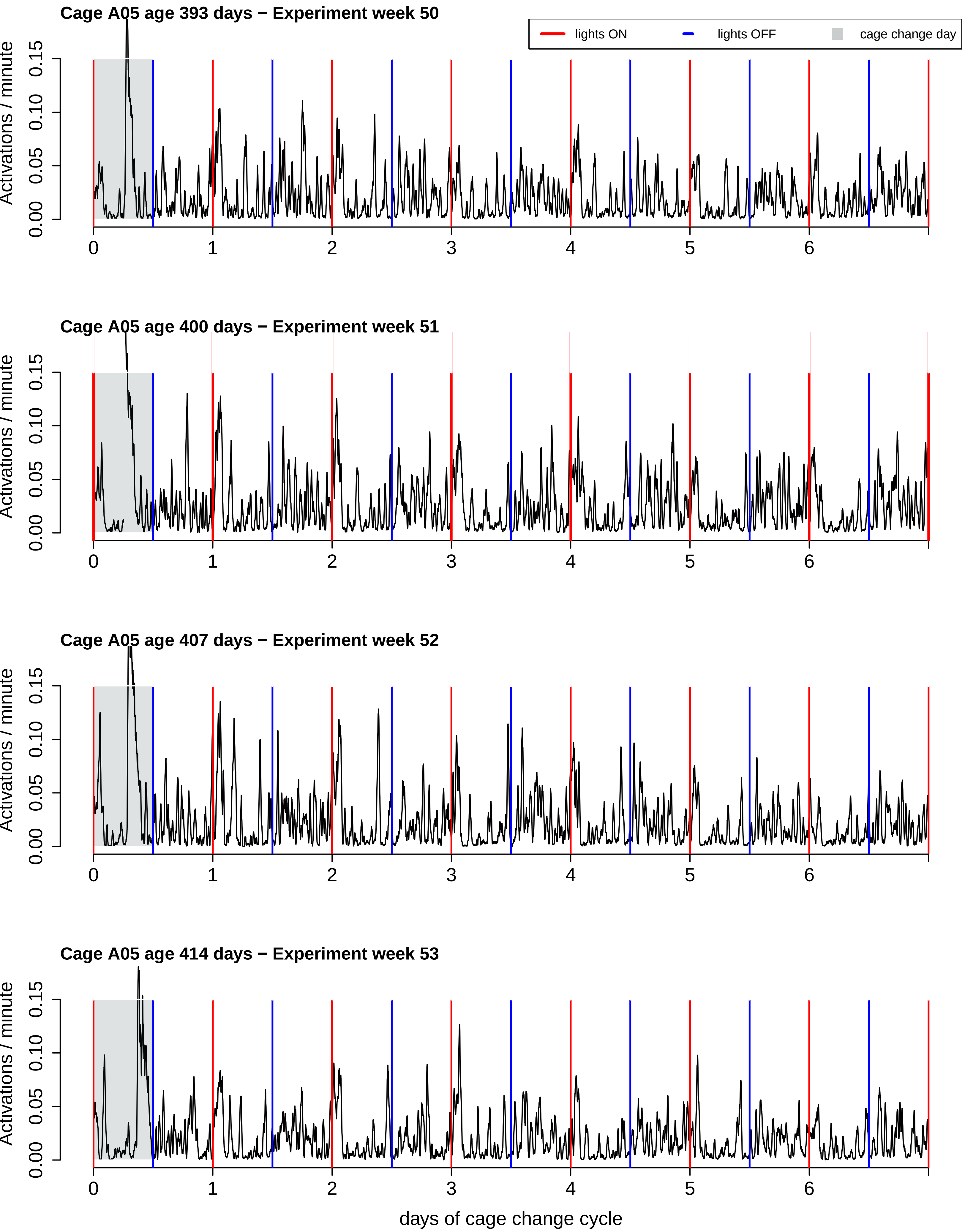

Cage A05 age 393 days − Experiment week 50
lights ON
lights OFF
cage change day
Activations / minute
0.00
0.05
0.10
0.15
0
1
2
3
4
5
6
Cage A05 age 400 days − Experiment week 51
Activations / minute
Cage A05 age 407 days − Experiment week 52
Activations / minute
Cage A05 age 414 days − Experiment week 53
Activations / minute
days of cage change cycle

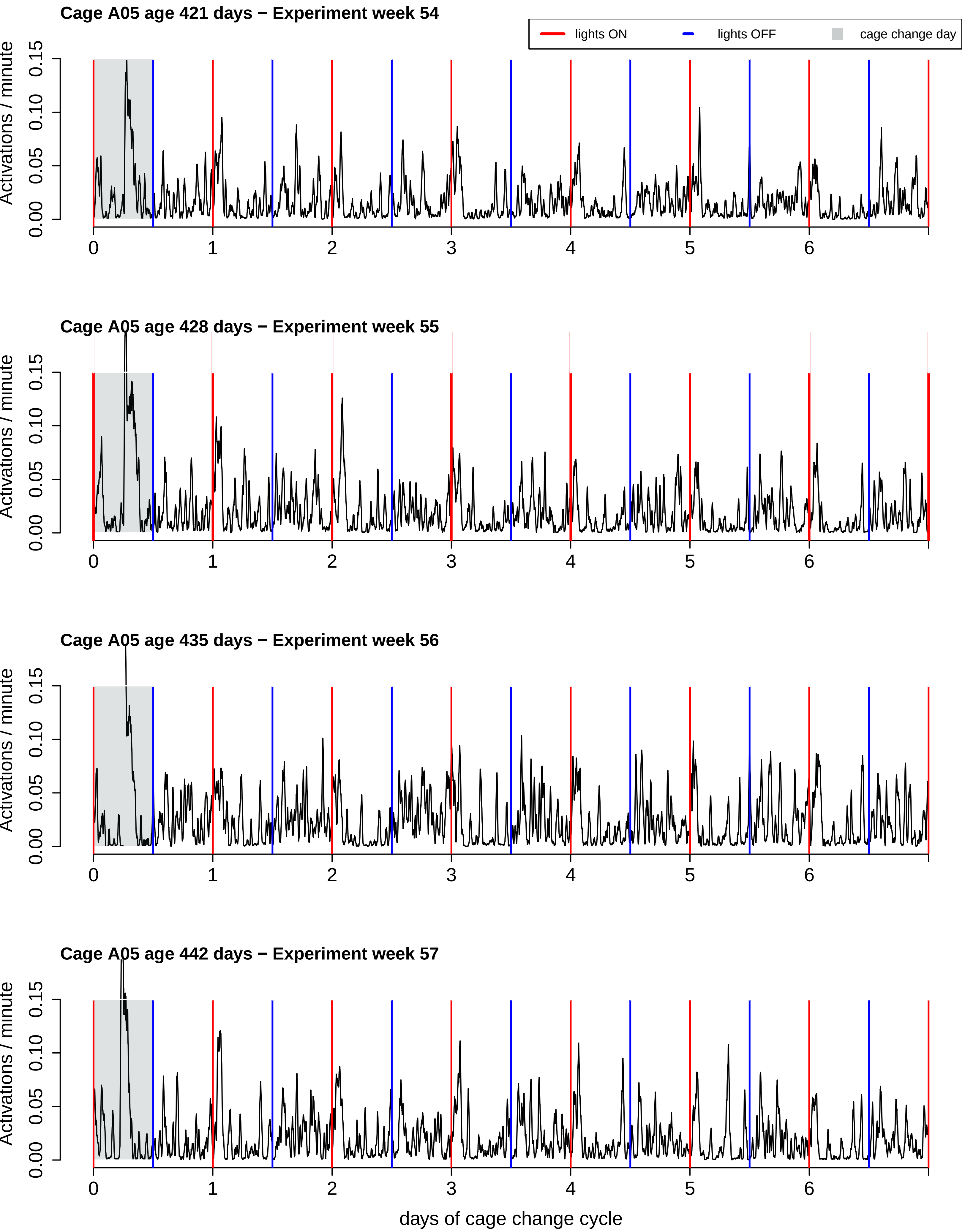

Cage A05 age 421 days − Experiment week 54
lights ON
lights OFF
cage change day
Activations / minute
0.00
0.05
0.10
0.15
0
1
2
3
4
5
6
Cage A05 age 428 days − Experiment week 55
Cage A05 age 435 days − Experiment week 56
Cage A05 age 442 days − Experiment week 57
days of cage change cycle

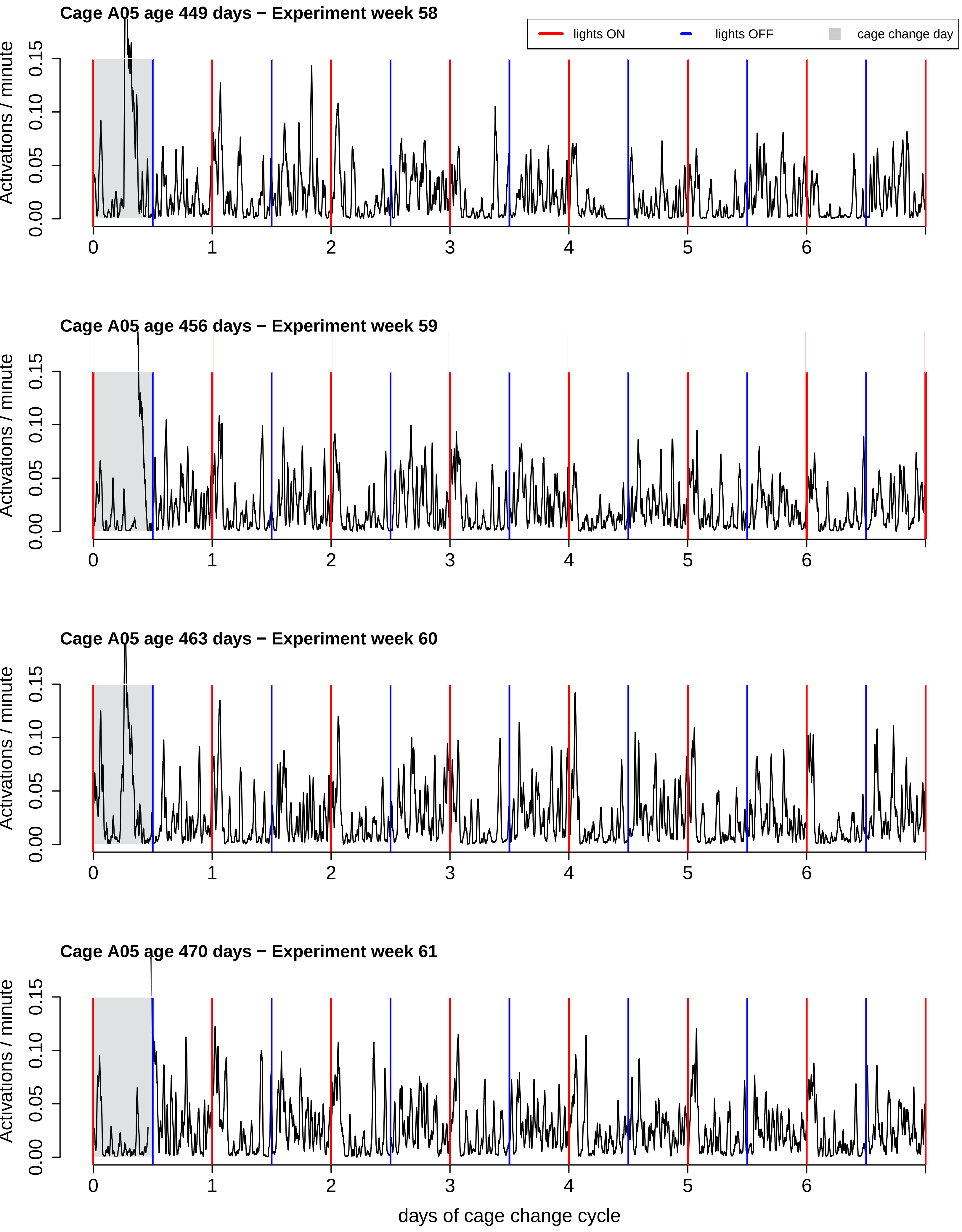
Cage A05 age 449 days − Experiment week 58
lights ON
lights OFF
cage change day
Activations / minute
0.00
0.05
0.10
0.15
0
1
2
3
4
5
6
Cage A05 age 456 days − Experiment week 59
Cage A05 age 463 days − Experiment week 60
Cage A05 age 470 days − Experiment week 61
days of cage change cycle

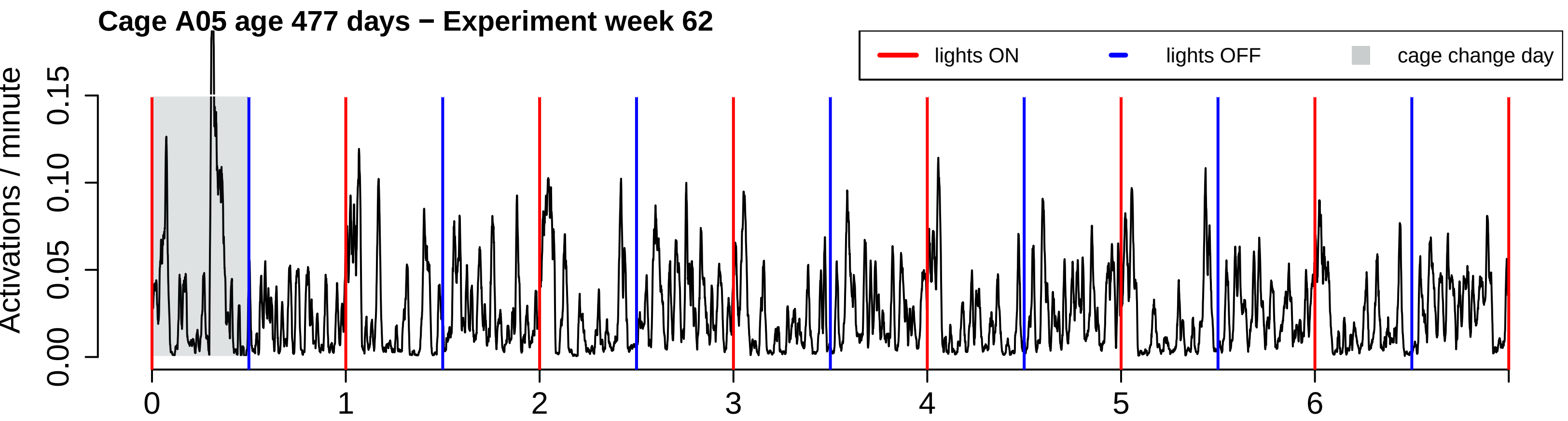

Cage A05 age 477 days − Experiment week 62
lights ON
lights OFF
cage change day
Activations / minute
0.00
0.05
0.10
0.15
0
1
2
3
4
5
6

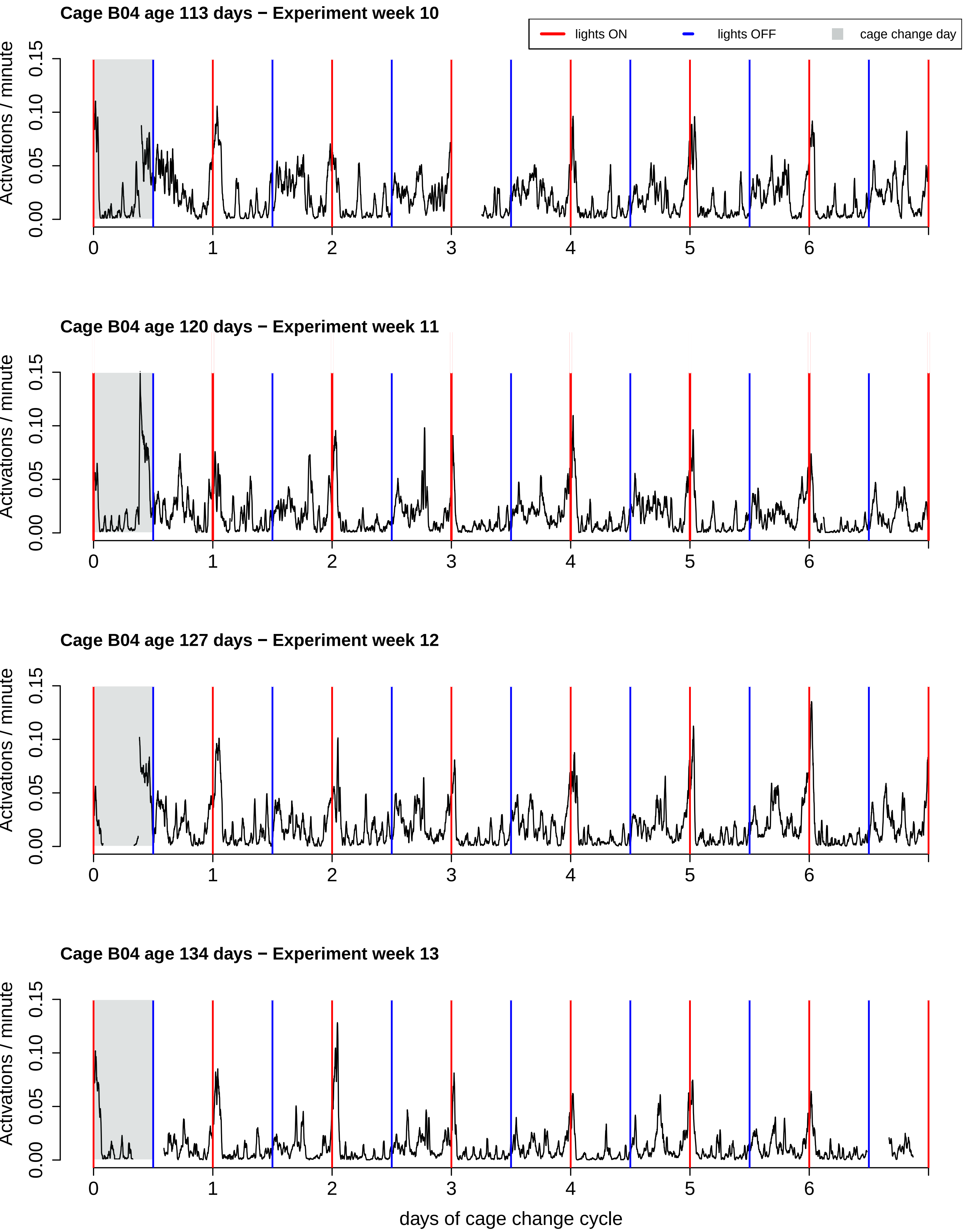
Cage B04 age 113 days − Experiment week 10
lights ON
lights OFF
cage change day
Activations / minute
0.00
0.05
0.10
0.15
0
1
2
3
4
5
6
Cage B04 age 120 days − Experiment week 11
Cage B04 age 127 days − Experiment week 12
Cage B04 age 134 days − Experiment week 13
days of cage change cycle

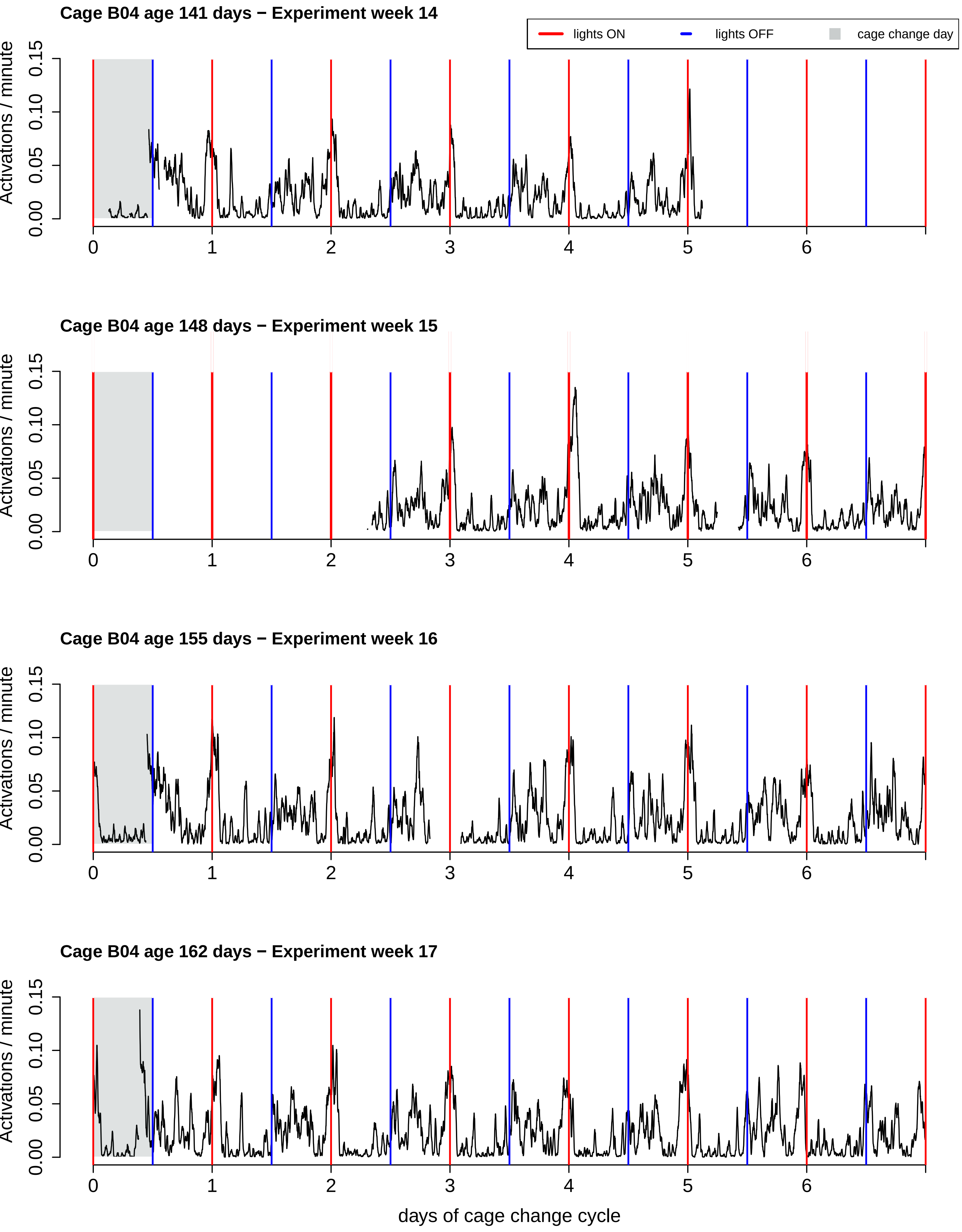

Cage B04 age 141 days − Experiment week 14
lights ON
lights OFF
cage change day
Activations / minute
0.00
0.05
0.10
0.15
0
1
2
3
4
5
6
Cage B04 age 148 days − Experiment week 15
Cage B04 age 155 days − Experiment week 16
Cage B04 age 162 days − Experiment week 17
days of cage change cycle

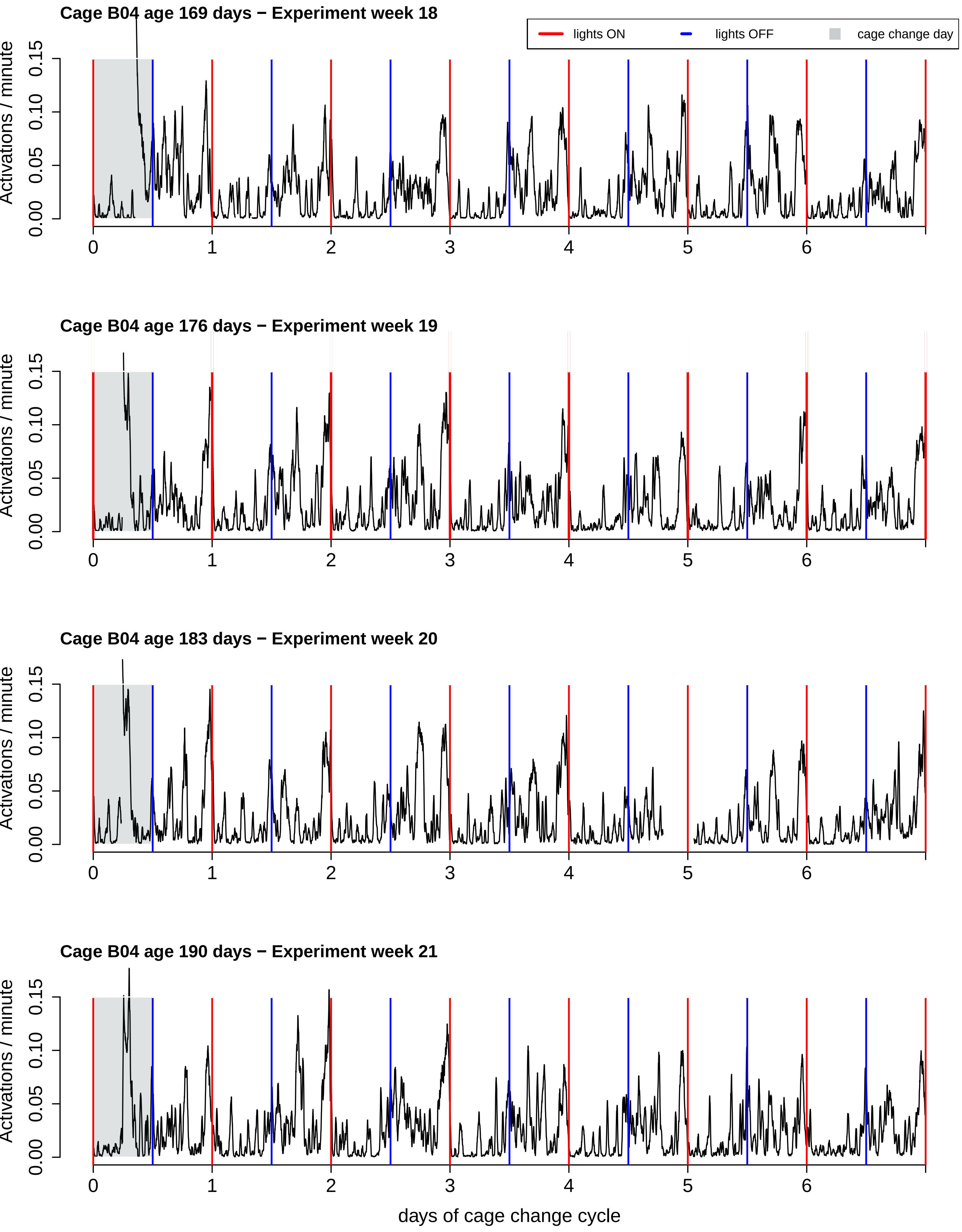

Cage B04 age 169 days − Experiment week 18
lights ON
lights OFF
cage change day
Activations / minute
0.00
0.05
0.10
0.15
0
1
2
3
4
5
6
Cage B04 age 176 days − Experiment week 19
Cage B04 age 183 days − Experiment week 20
Cage B04 age 190 days − Experiment week 21
days of cage change cycle

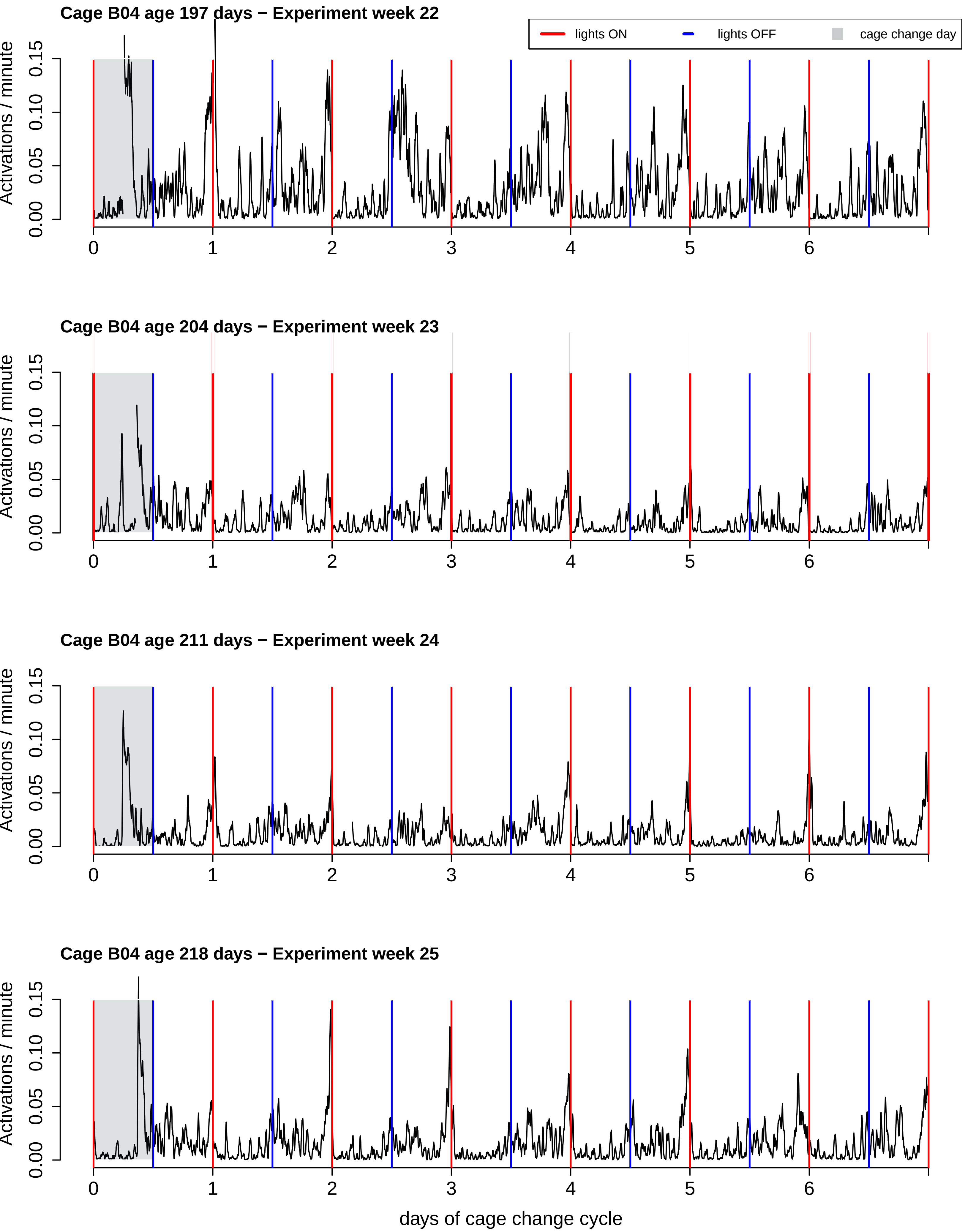

Cage B04 age 197 days − Experiment week 22
lights ON
lights OFF
cage change day
Activations / minute
0.00
0.05
0.10
0.15
0
1
2
3
4
5
6
Cage B04 age 204 days − Experiment week 23
Cage B04 age 211 days − Experiment week 24
Cage B04 age 218 days − Experiment week 25
days of cage change cycle

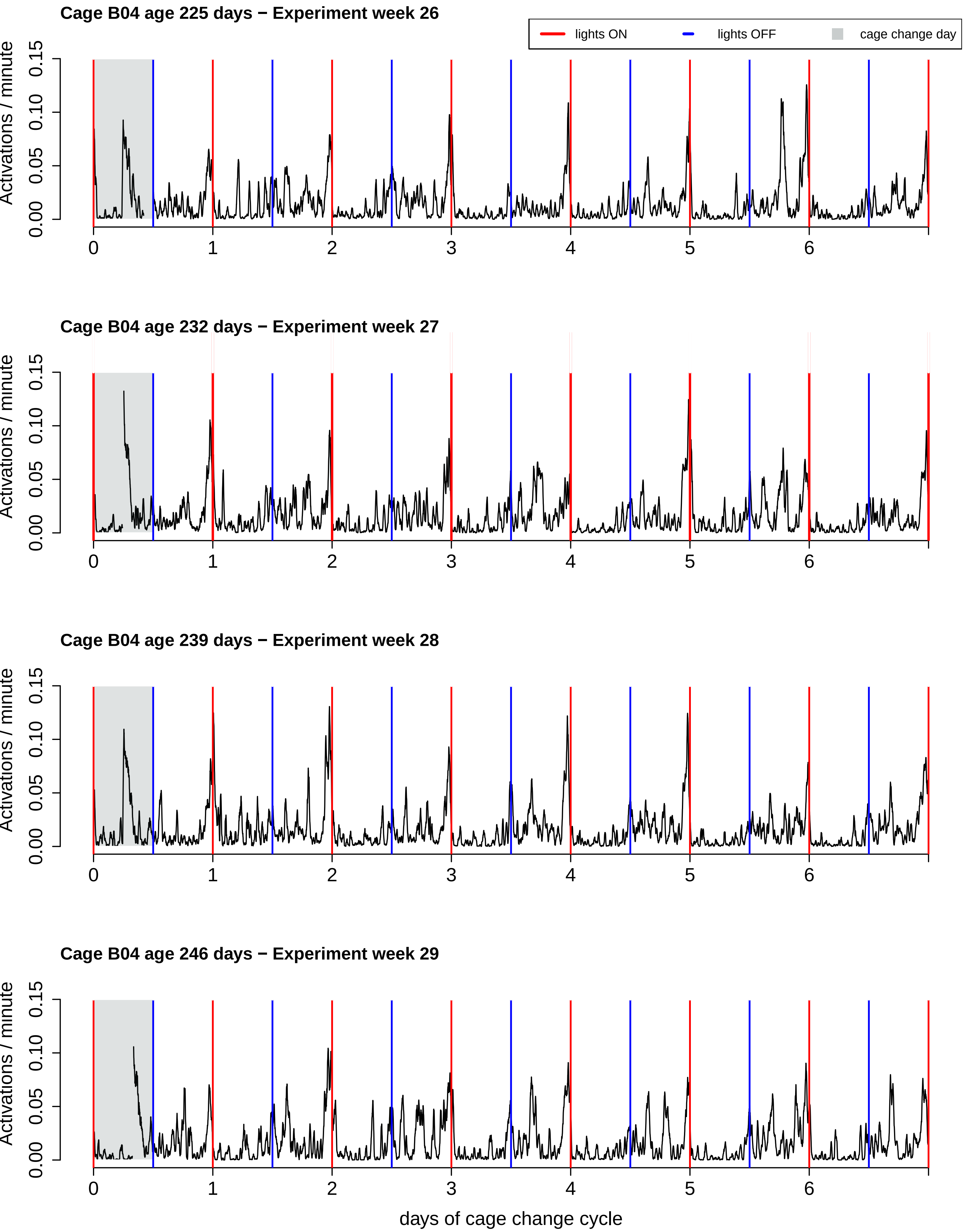
Cage B04 age 225 days − Experiment week 26
lights ON
lights OFF
cage change day
Activations / minute
0.00
0.05
0.10
0.15
0
1
2
3
4
5
6
Cage B04 age 232 days − Experiment week 27
Cage B04 age 239 days − Experiment week 28
Cage B04 age 246 days − Experiment week 29
days of cage change cycle

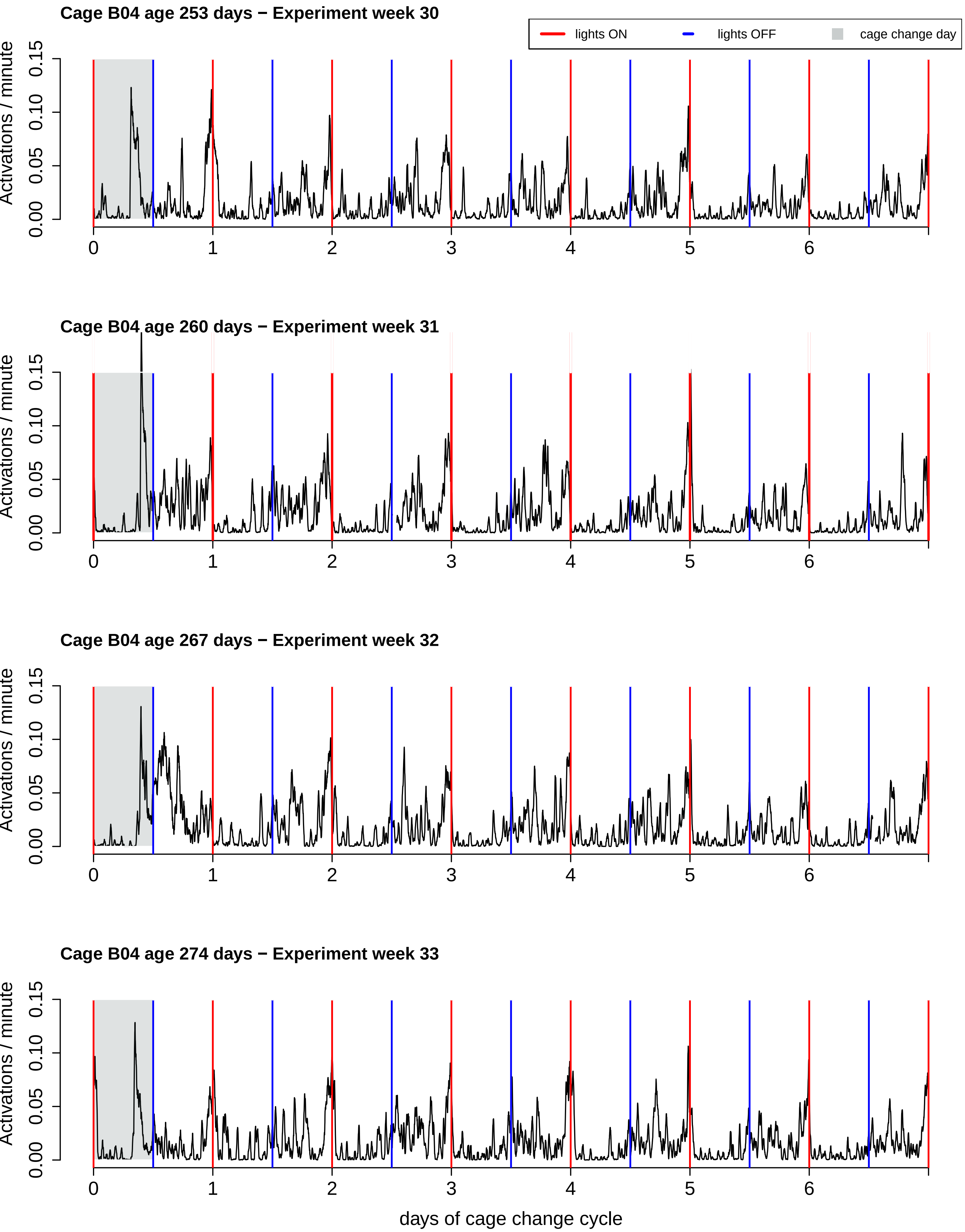
Cage B04 age 253 days − Experiment week 30
lights ON
lights OFF
cage change day
Activations / minute
0.00
0.05
0.10
0.15
0
1
2
3
4
5
6
Cage B04 age 260 days − Experiment week 31
Cage B04 age 267 days − Experiment week 32
Cage B04 age 274 days − Experiment week 33
days of cage change cycle

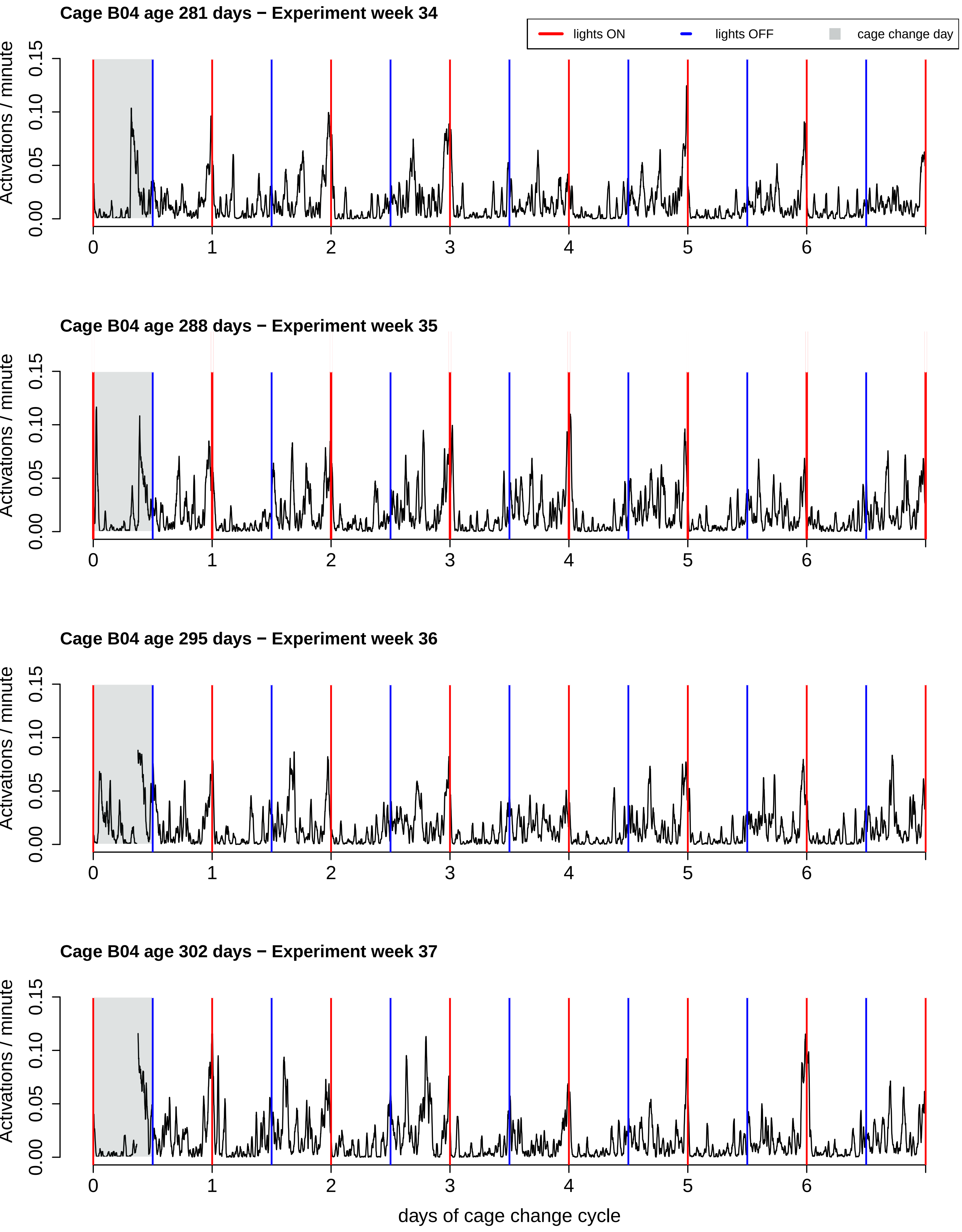

Cage B04 age 281 days − Experiment week 34
lights ON
lights OFF
cage change day
Activations / minute
0.00
0.05
0.10
0.15
0
1
2
3
4
5
6
Cage B04 age 288 days − Experiment week 35
Cage B04 age 295 days − Experiment week 36
Cage B04 age 302 days − Experiment week 37
days of cage change cycle

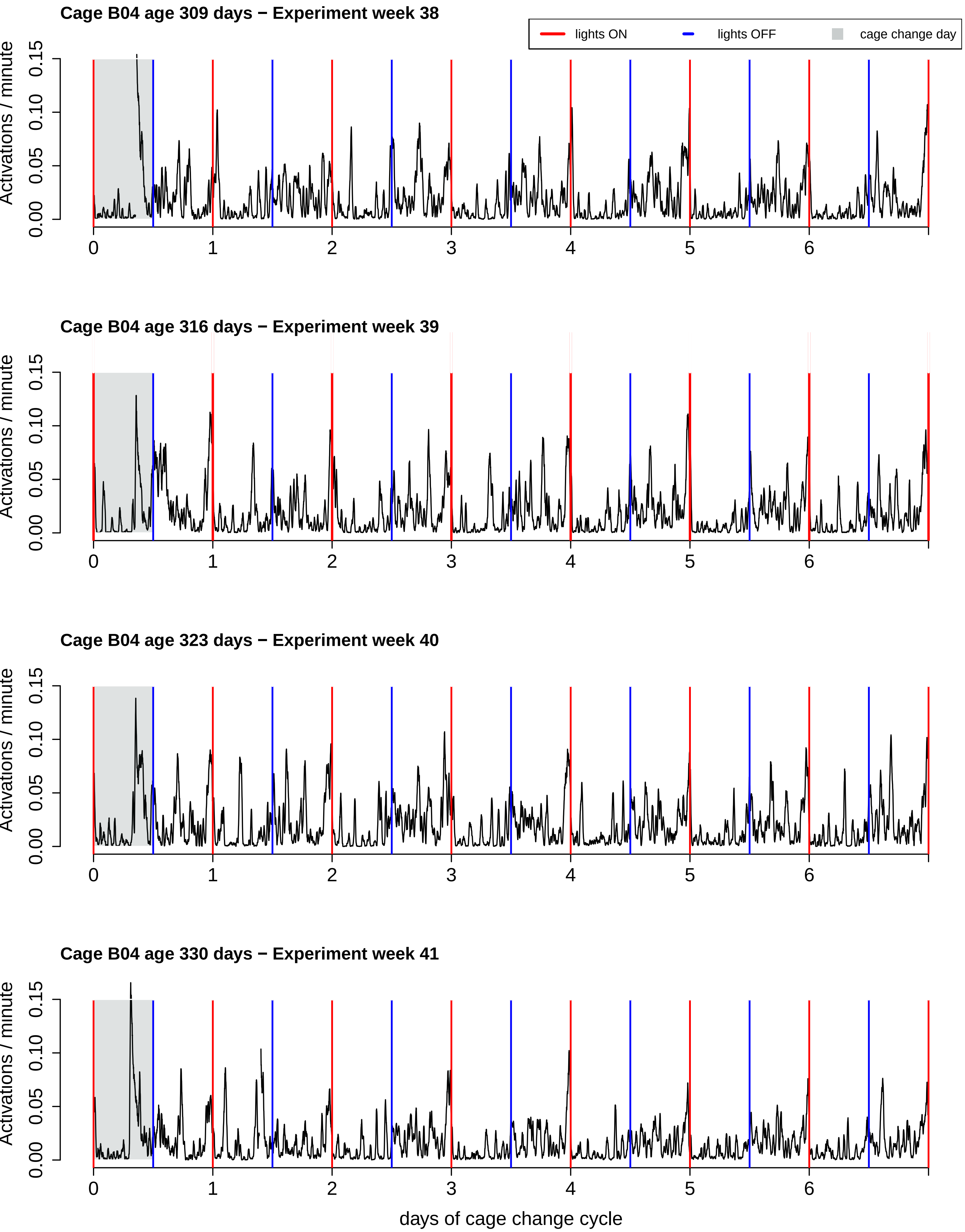
Cage B04 age 309 days − Experiment week 38
lights ON
lights OFF
cage change day
Activations / minute
0.00
0.05
0.10
0.15
0
1
2
3
4
5
6
Cage B04 age 316 days − Experiment week 39
Cage B04 age 323 days − Experiment week 40
Cage B04 age 330 days − Experiment week 41
days of cage change cycle

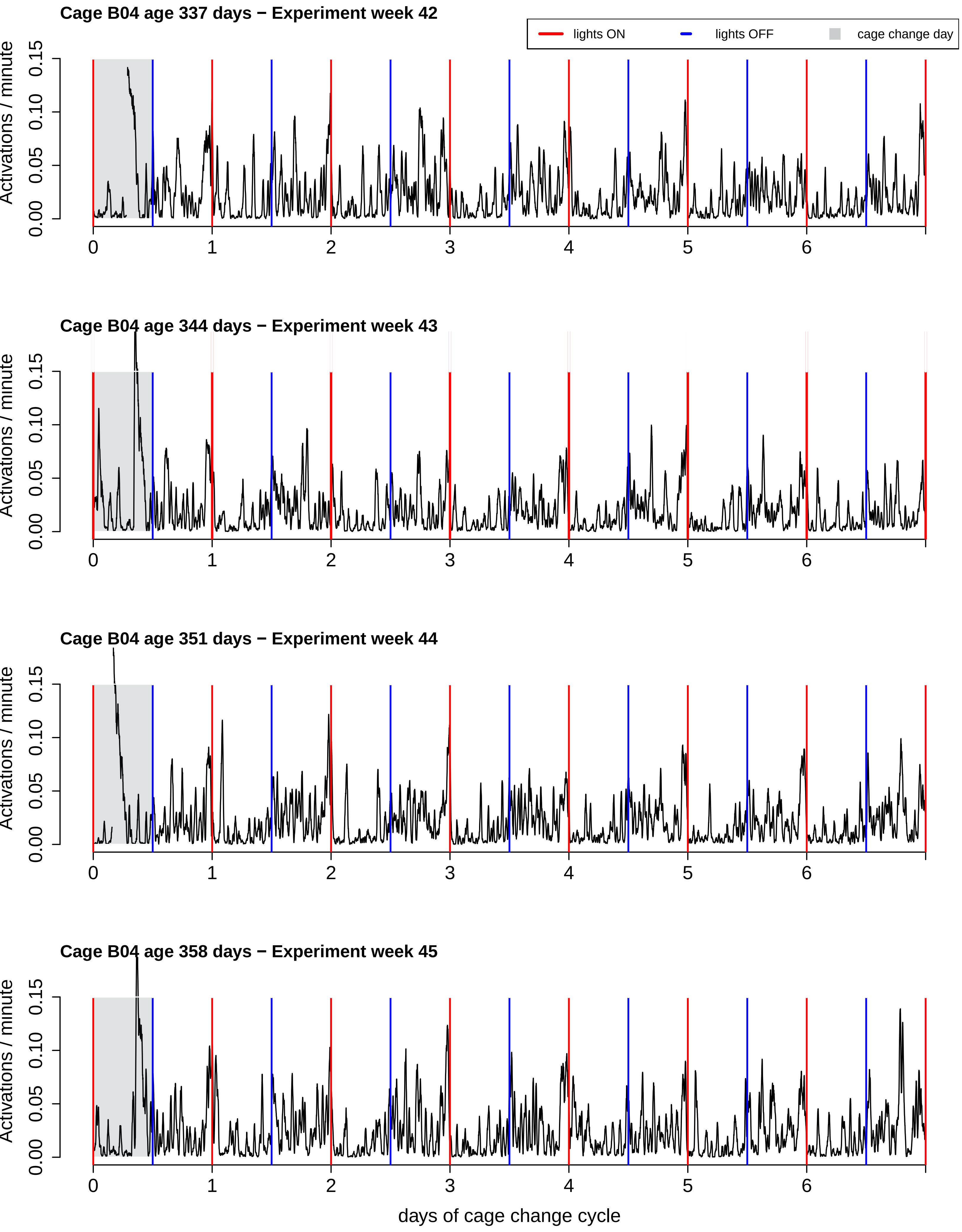
Cage B04 age 337 days − Experiment week 42
lights ON
lights OFF
cage change day
Activations / minute
0.00
0.05
0.10
0.15
0
1
2
3
4
5
6
Cage B04 age 344 days − Experiment week 43
Cage B04 age 351 days − Experiment week 44
Cage B04 age 358 days − Experiment week 45
days of cage change cycle

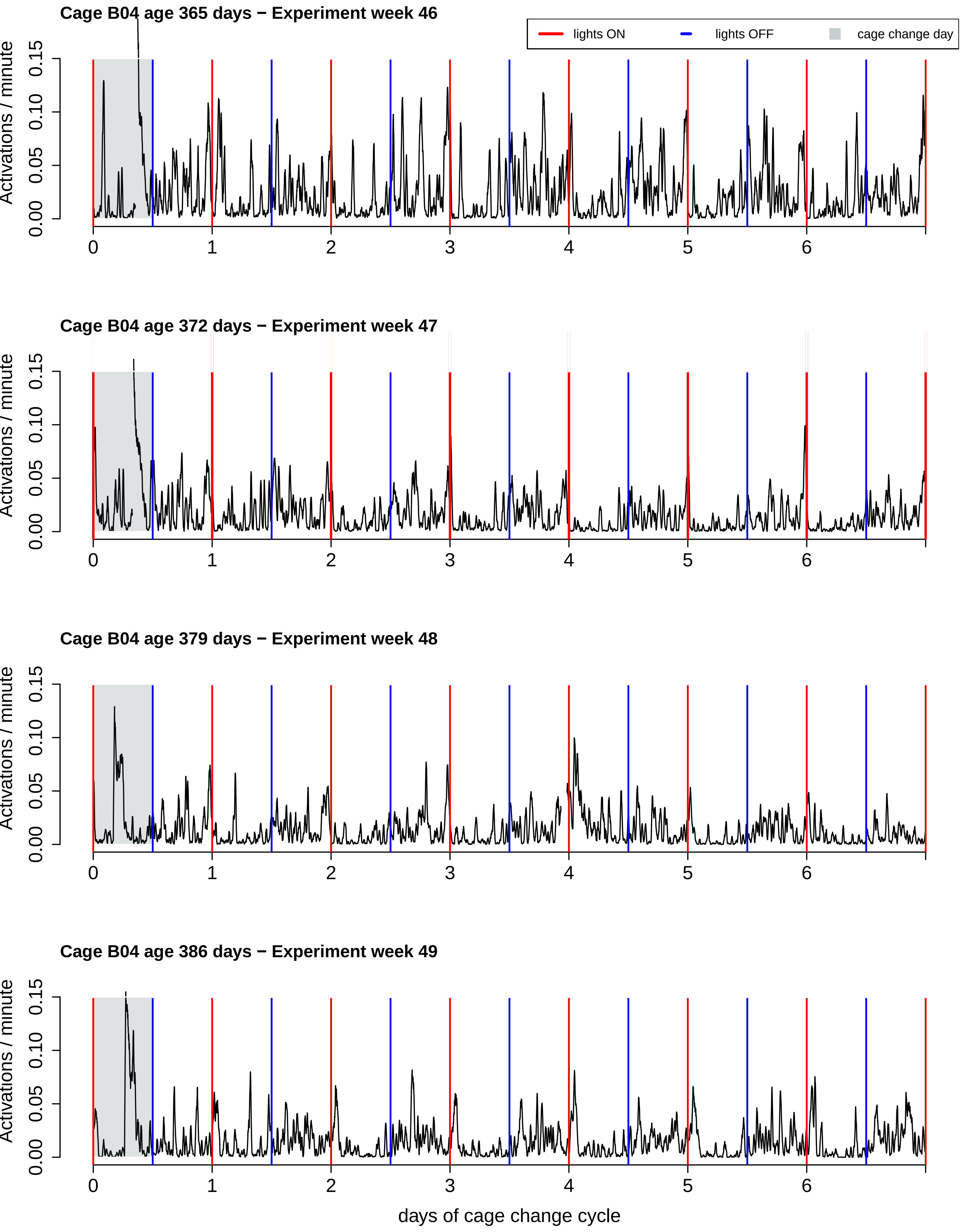
Cage B04 age 365 days − Experiment week 46
lights ON
lights OFF
cage change day
Activations / minute
0.00
0.05
0.10
0.15
0
1
2
3
4
5
6
Cage B04 age 372 days − Experiment week 47
Cage B04 age 379 days − Experiment week 48
Cage B04 age 386 days − Experiment week 49
days of cage change cycle

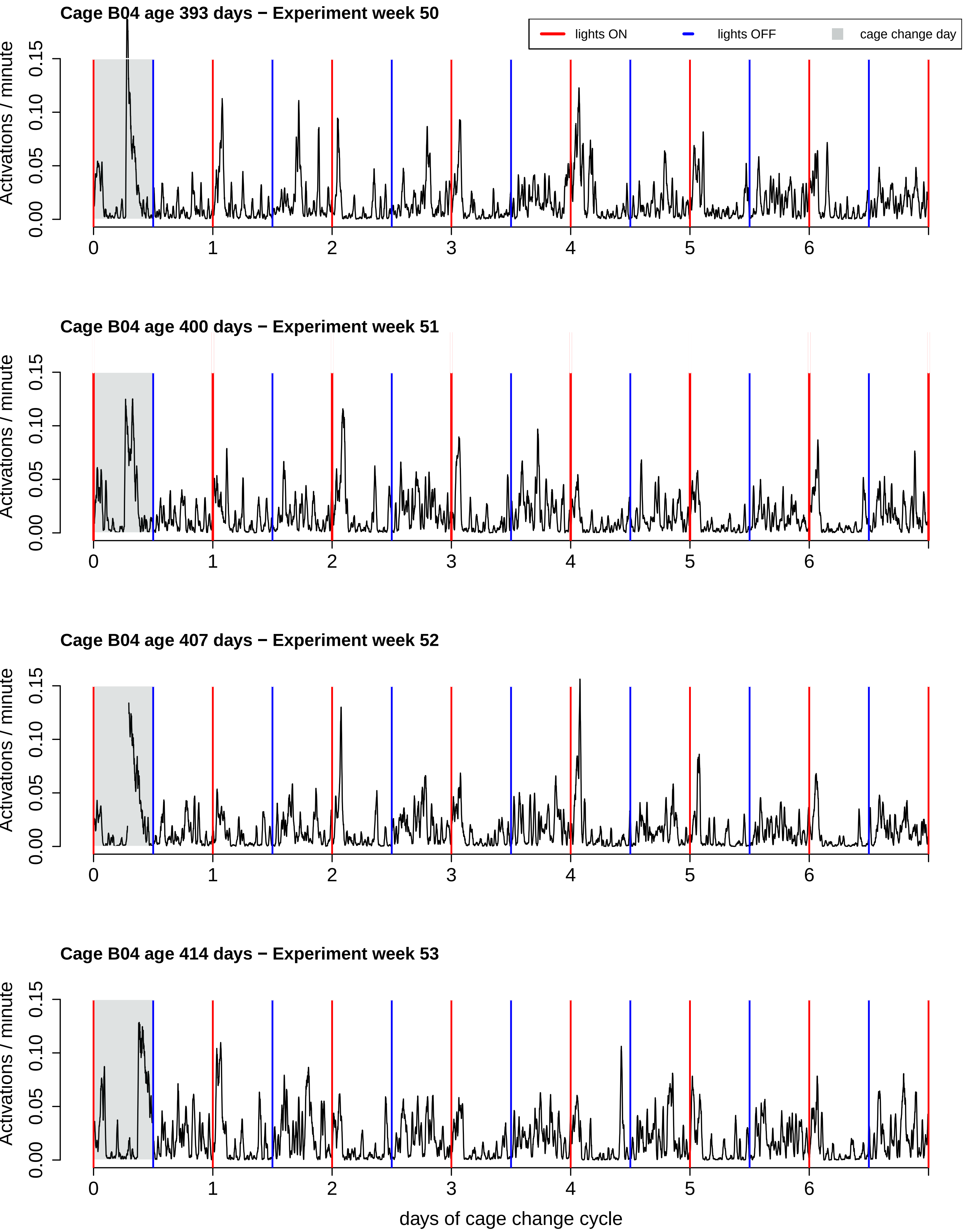
Cage B04 age 393 days − Experiment week 50
lights ON
lights OFF
cage change day
Activations / minute
0.00
0.05
0.10
0.15
0
1
2
3
4
5
6
Cage B04 age 400 days − Experiment week 51
Cage B04 age 407 days − Experiment week 52
Cage B04 age 414 days − Experiment week 53
days of cage change cycle

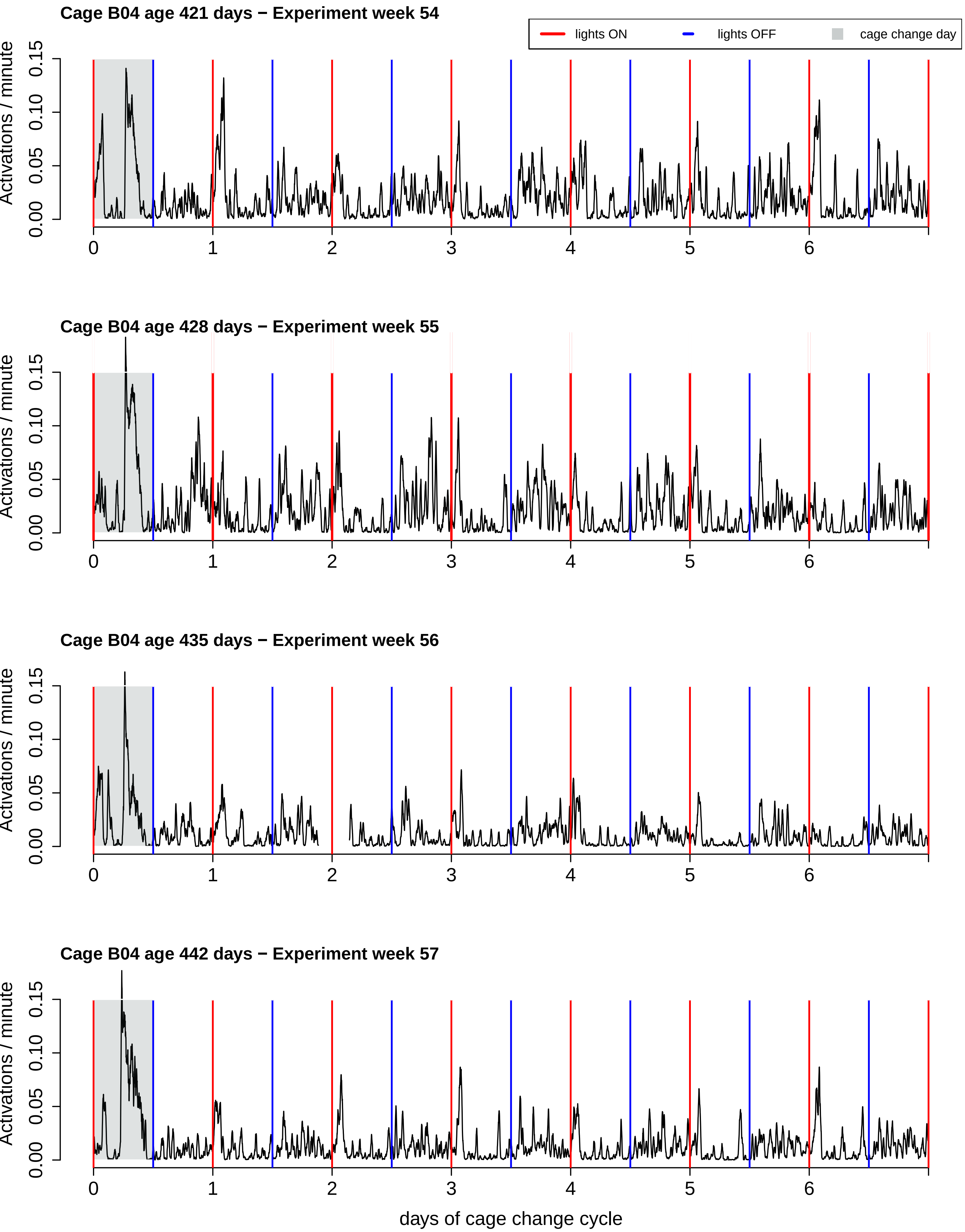
Cage B04 age 421 days − Experiment week 54
lights ON
lights OFF
cage change day
Activations / minute
0.00
0.05
0.10
0.15
0
1
2
3
4
5
6
Cage B04 age 428 days − Experiment week 55
Cage B04 age 435 days − Experiment week 56
Cage B04 age 442 days − Experiment week 57
days of cage change cycle

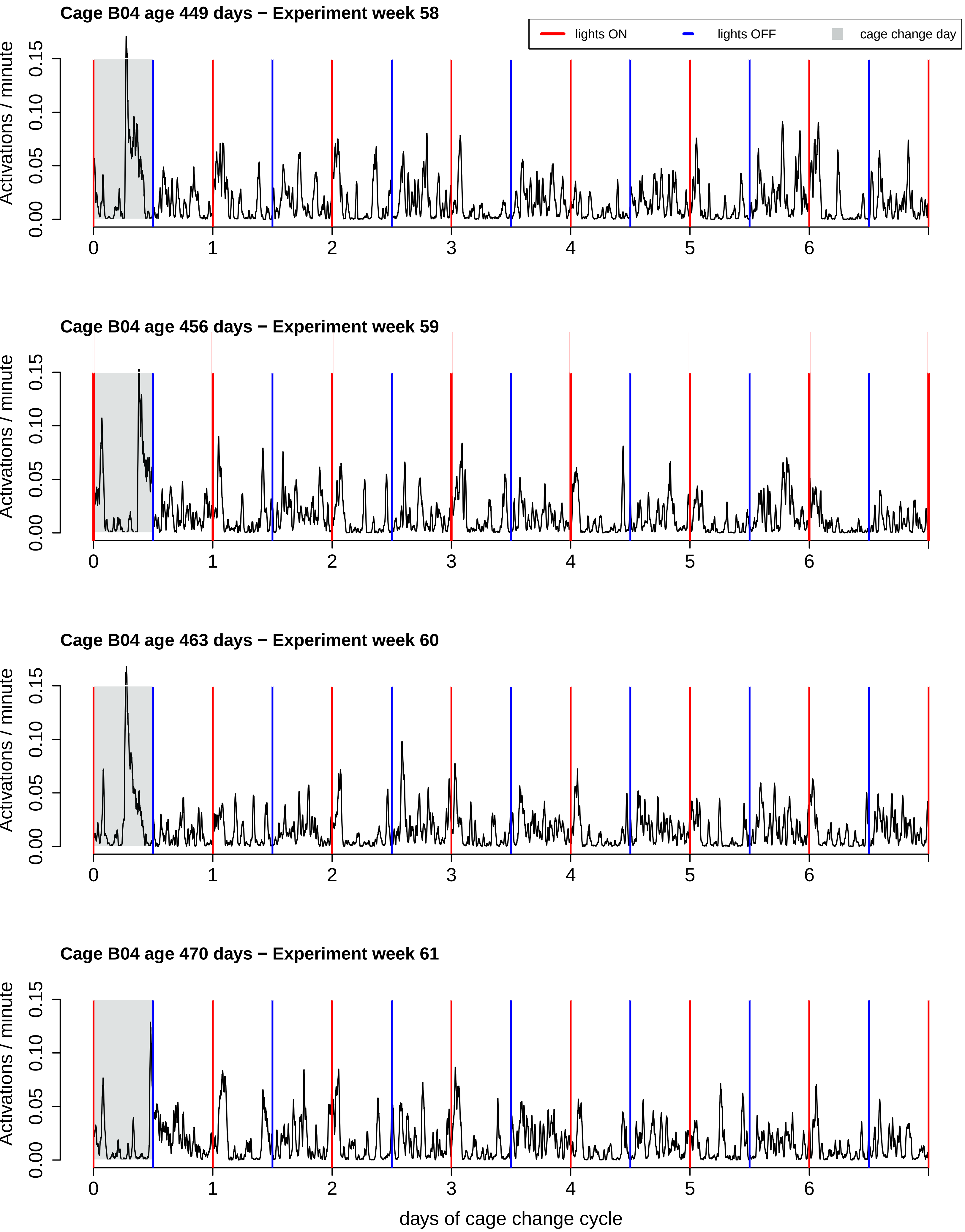
Cage B04 age 449 days − Experiment week 58
lights ON
lights OFF
cage change day
Activations / minute
0.00
0.05
0.10
0.15
0
1
2
3
4
5
6
Cage B04 age 456 days − Experiment week 59
Cage B04 age 463 days − Experiment week 60
Cage B04 age 470 days − Experiment week 61
days of cage change cycle

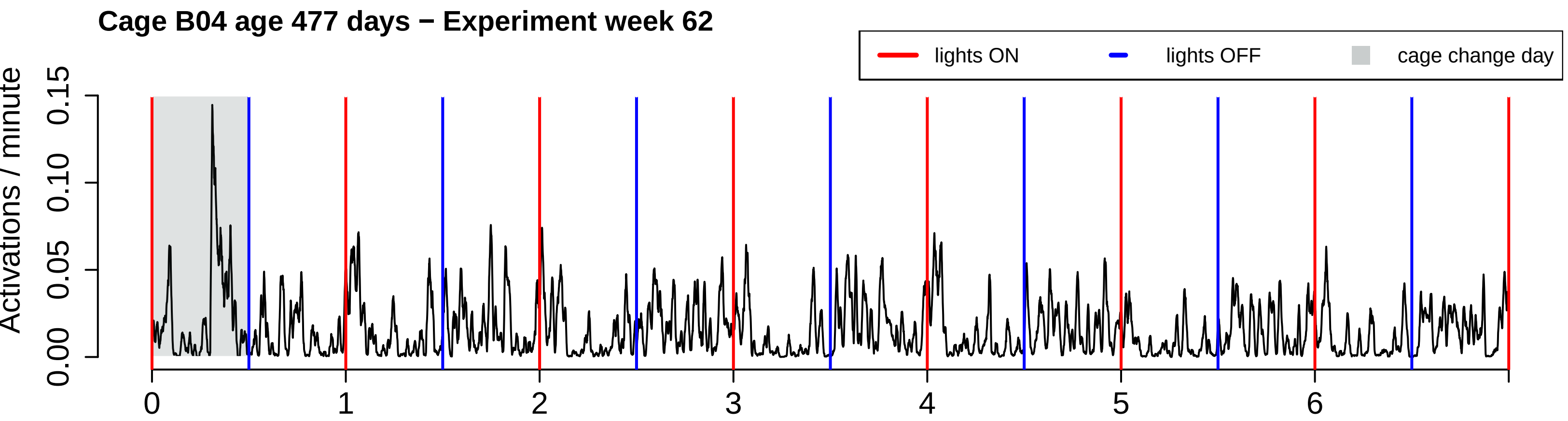
Cage B04 age 477 days − Experiment week 62
lights ON
lights OFF
cage change day
Activations / minute
0.00
0.05
0.10
0.15
0
1
2
3
4
5
6

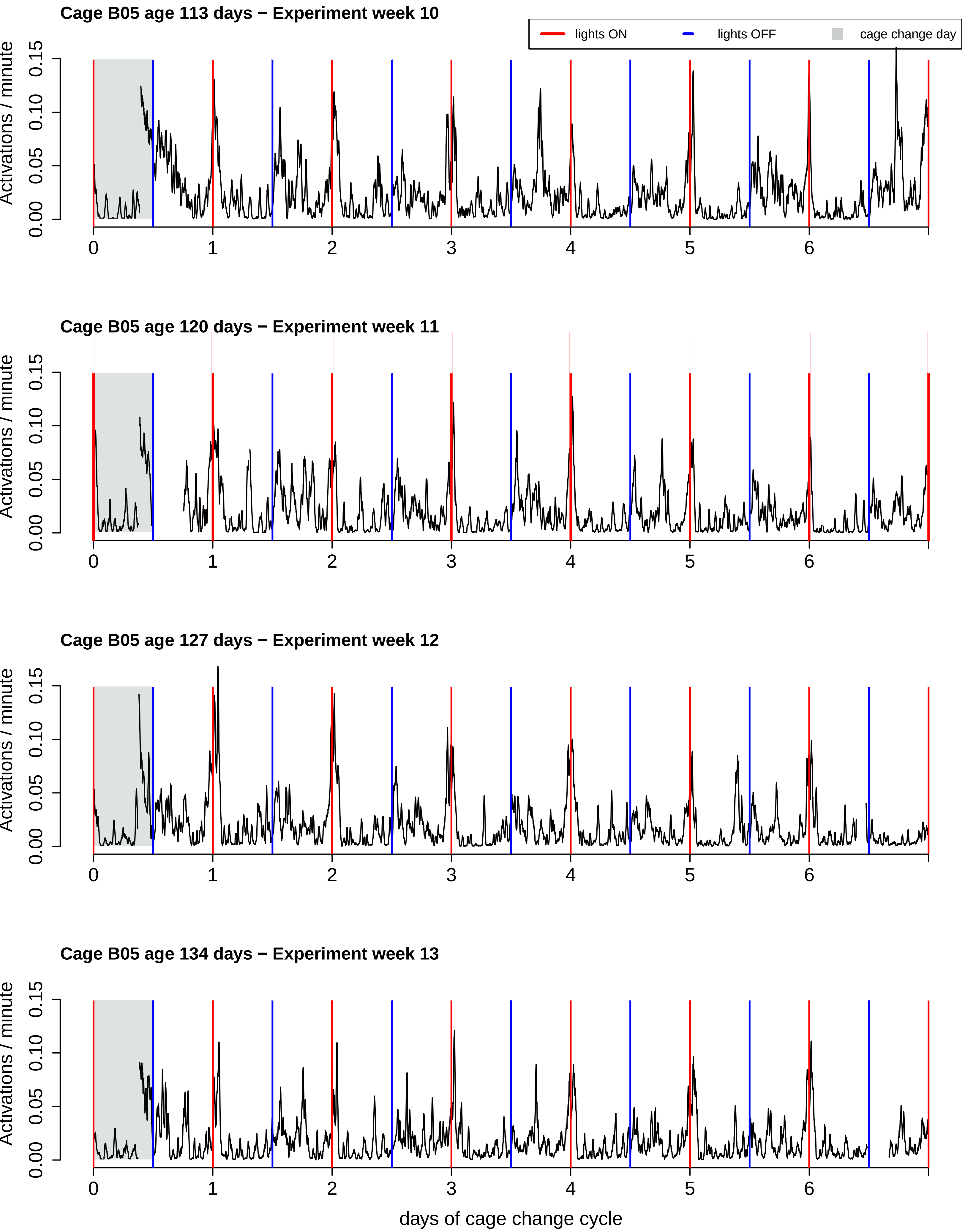
Cage B05 age 113 days − Experiment week 10
lights ON
lights OFF
cage change day
Activations / minute
0.00
0.05
0.10
0.15
0
1
2
3
4
5
6
Cage B05 age 120 days − Experiment week 11
Activations / minute
0.00
0.05
0.10
0.15
0
1
2
3
4
5
6
Cage B05 age 127 days − Experiment week 12
Activations / minute
0.00
0.05
0.10
0.15
0
1
2
3
4
5
6
Cage B05 age 134 days − Experiment week 13
Activations / minute
0.00
0.05
0.10
0.15
0
1
2
3
4
5
6
days of cage change cycle

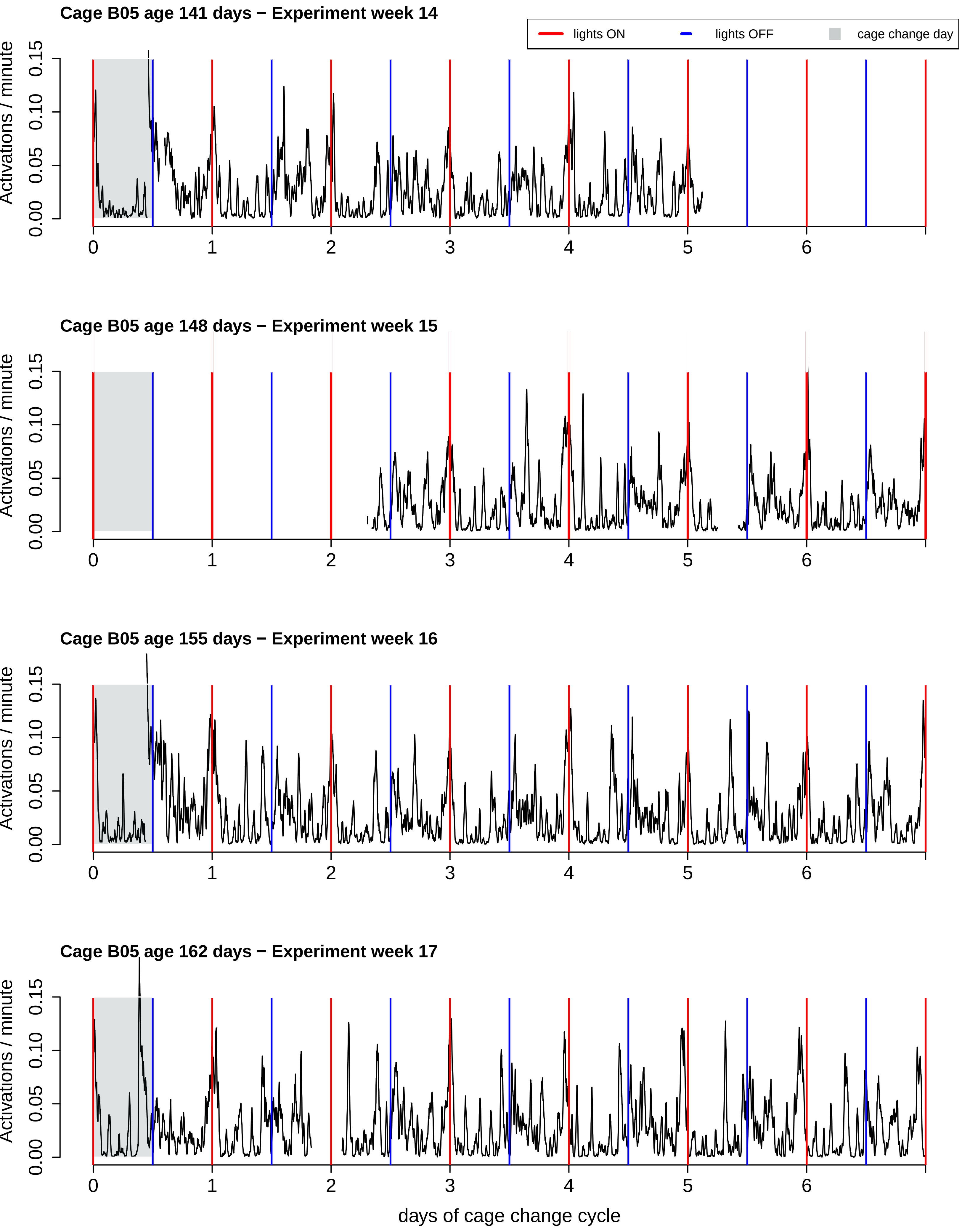

Cage B05 age 141 days − Experiment week 14
lights ON
lights OFF
cage change day
Activations / minute
0.00
0.05
0.10
0.15
0
1
2
3
4
5
6
Cage B05 age 148 days − Experiment week 15
Cage B05 age 155 days − Experiment week 16
Cage B05 age 162 days − Experiment week 17
days of cage change cycle

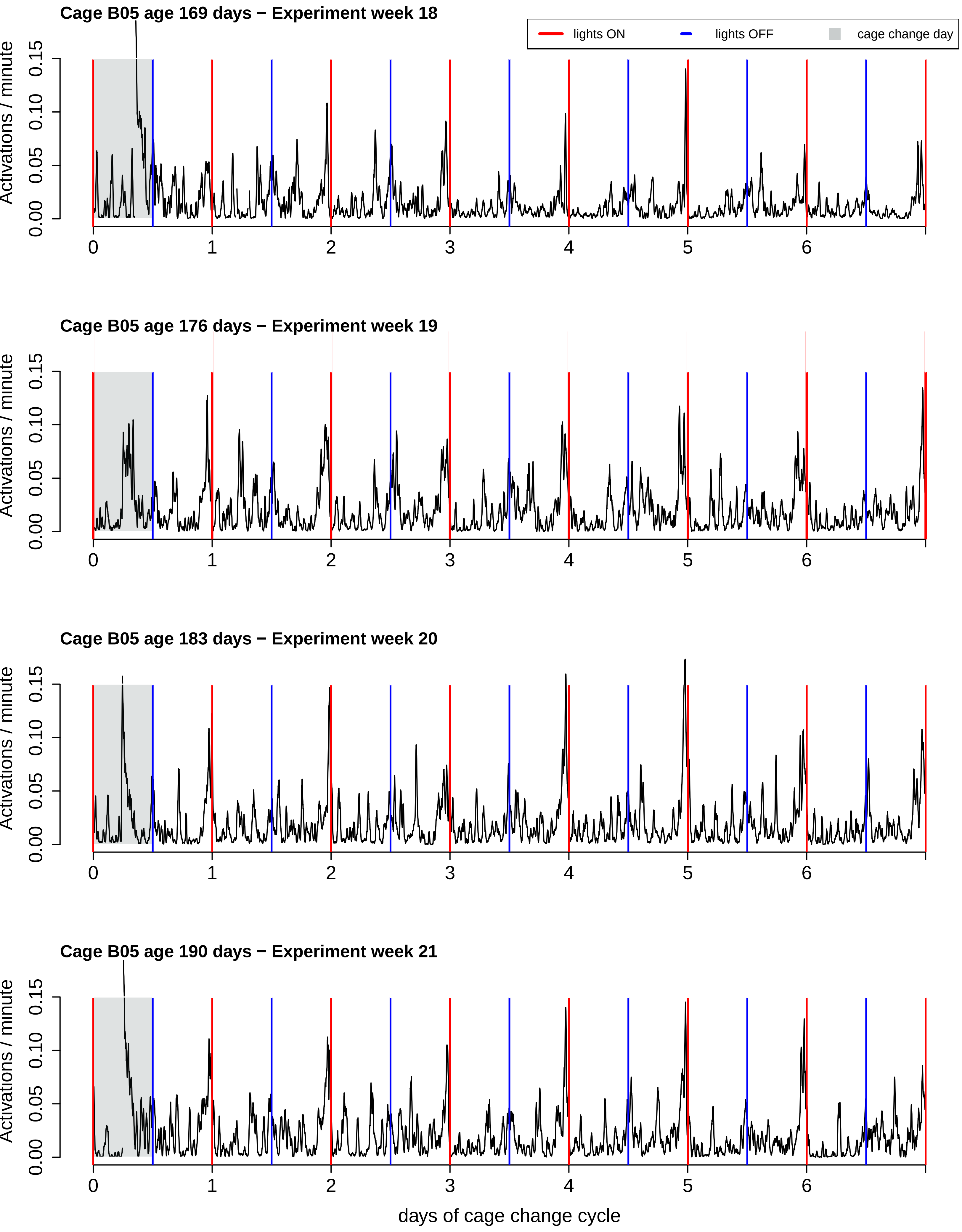

Cage B05 age 169 days − Experiment week 18
lights ON
lights OFF
cage change day
Activations / minute
Cage B05 age 176 days − Experiment week 19
Cage B05 age 183 days − Experiment week 20
Cage B05 age 190 days − Experiment week 21
days of cage change cycle

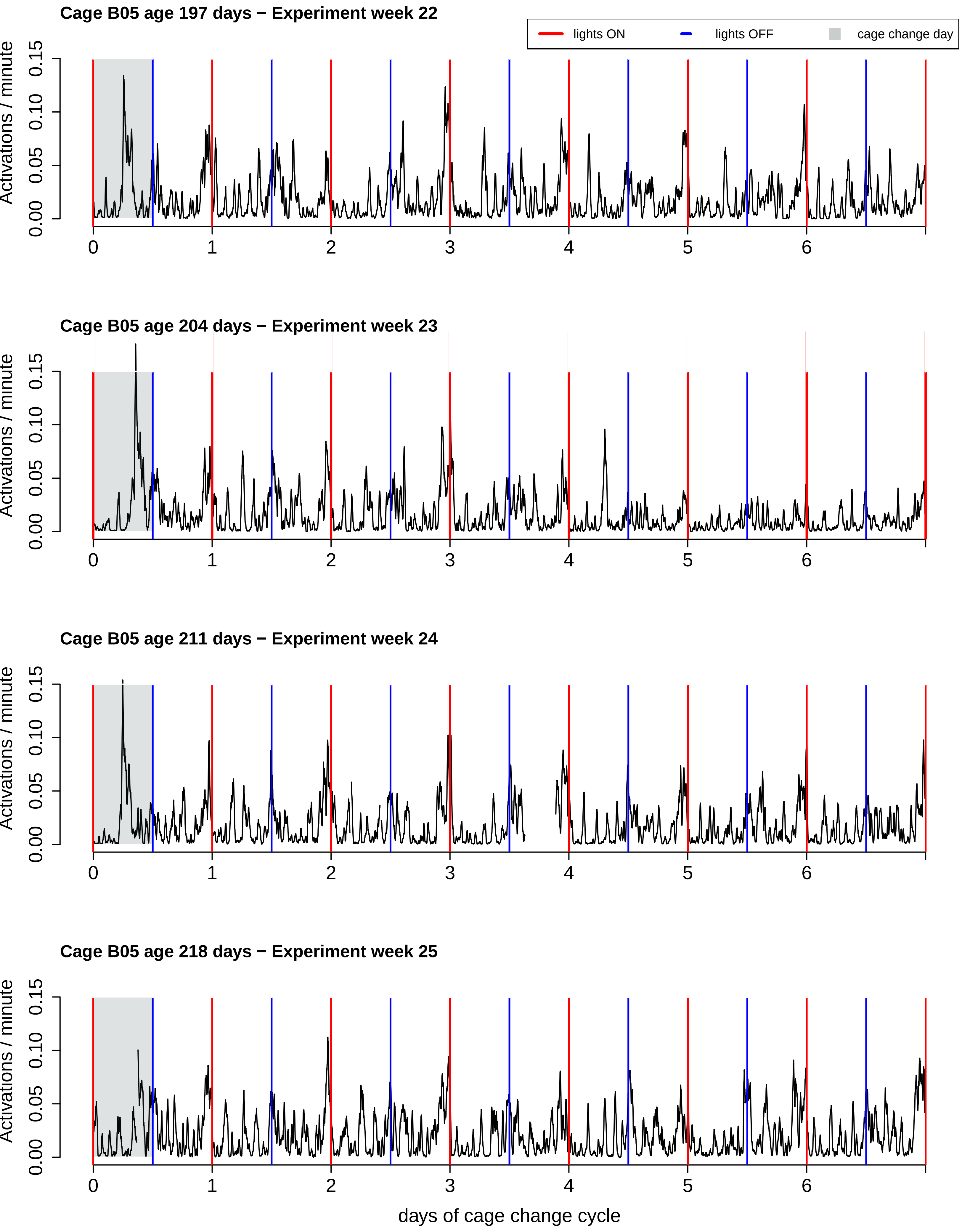
Cage B05 age 197 days − Experiment week 22
lights ON
lights OFF
cage change day
Activations / minute
0.00
0.05
0.10
0.15
0
1
2
3
4
5
6
Cage B05 age 204 days − Experiment week 23
Cage B05 age 211 days − Experiment week 24
Cage B05 age 218 days − Experiment week 25
days of cage change cycle

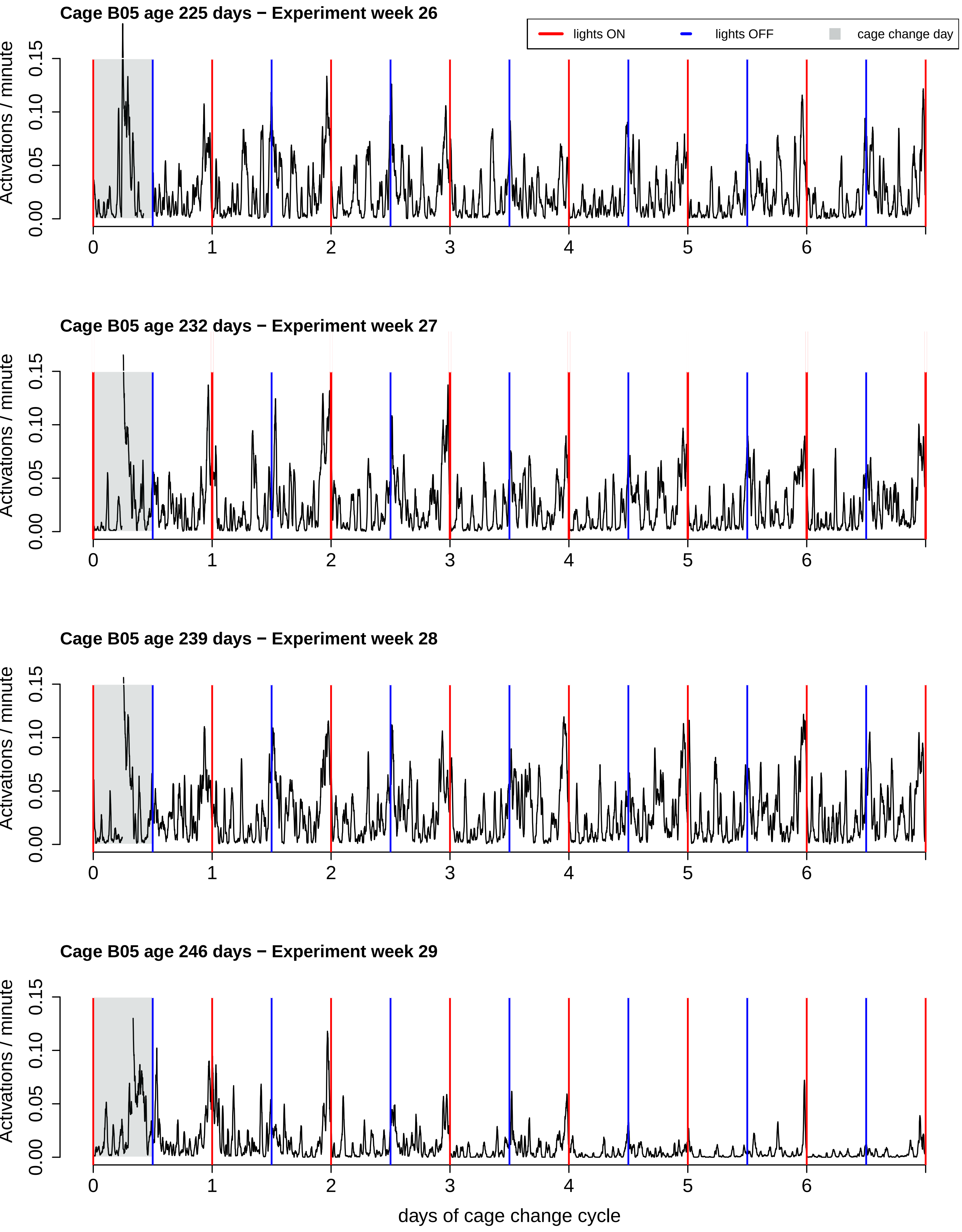
Cage B05 age 225 days − Experiment week 26
lights ON
lights OFF
cage change day
Activations / minute
0.00
0.05
0.10
0.15
0
1
2
3
4
5
6
Cage B05 age 232 days − Experiment week 27
Cage B05 age 239 days − Experiment week 28
Cage B05 age 246 days − Experiment week 29
days of cage change cycle

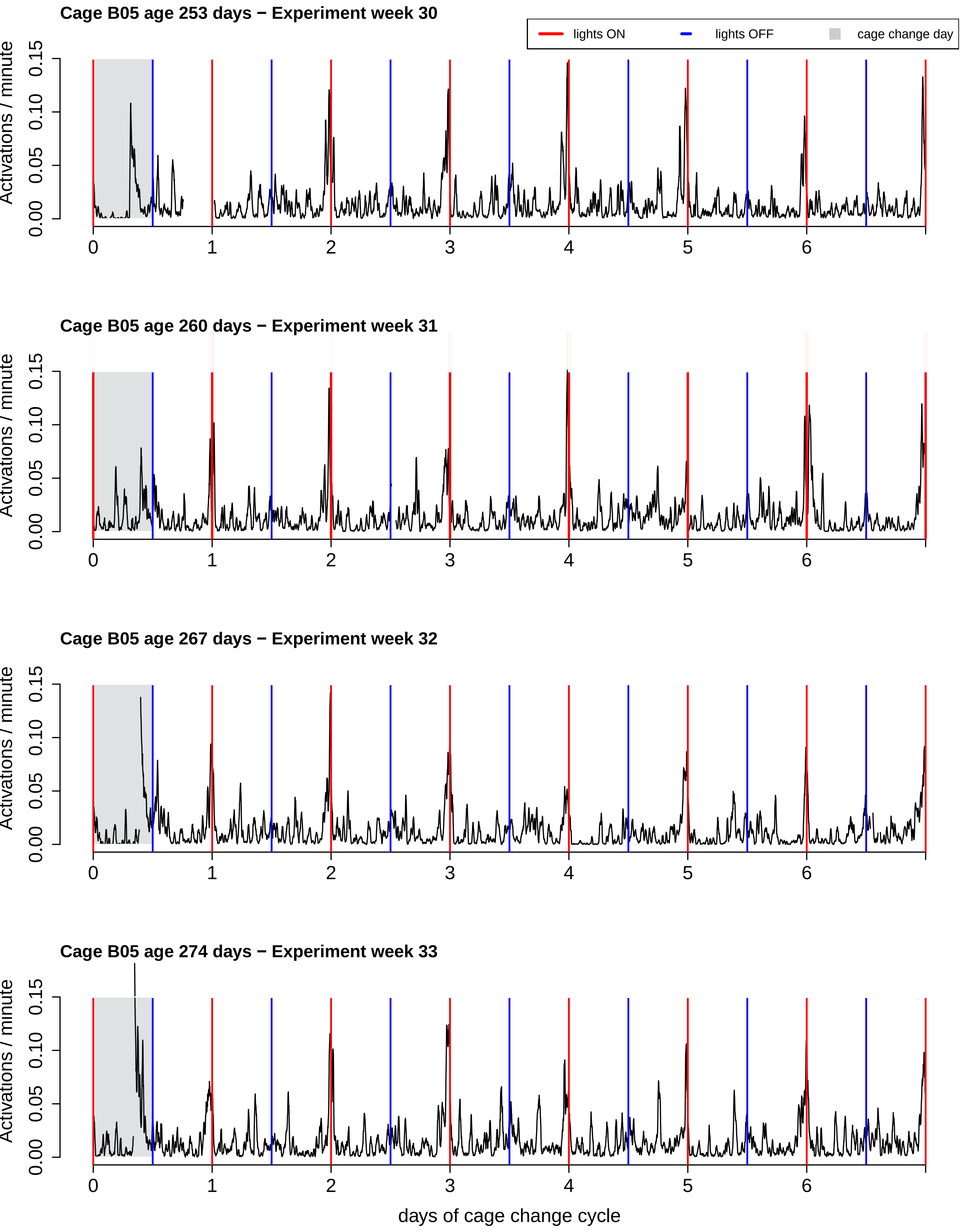

Cage B05 age 253 days − Experiment week 30
lights ON
lights OFF
cage change day
Activations / minute
0.00
0.05
0.10
0.15
0
1
2
3
4
5
6
Cage B05 age 260 days − Experiment week 31
Cage B05 age 267 days − Experiment week 32
Cage B05 age 274 days − Experiment week 33
days of cage change cycle

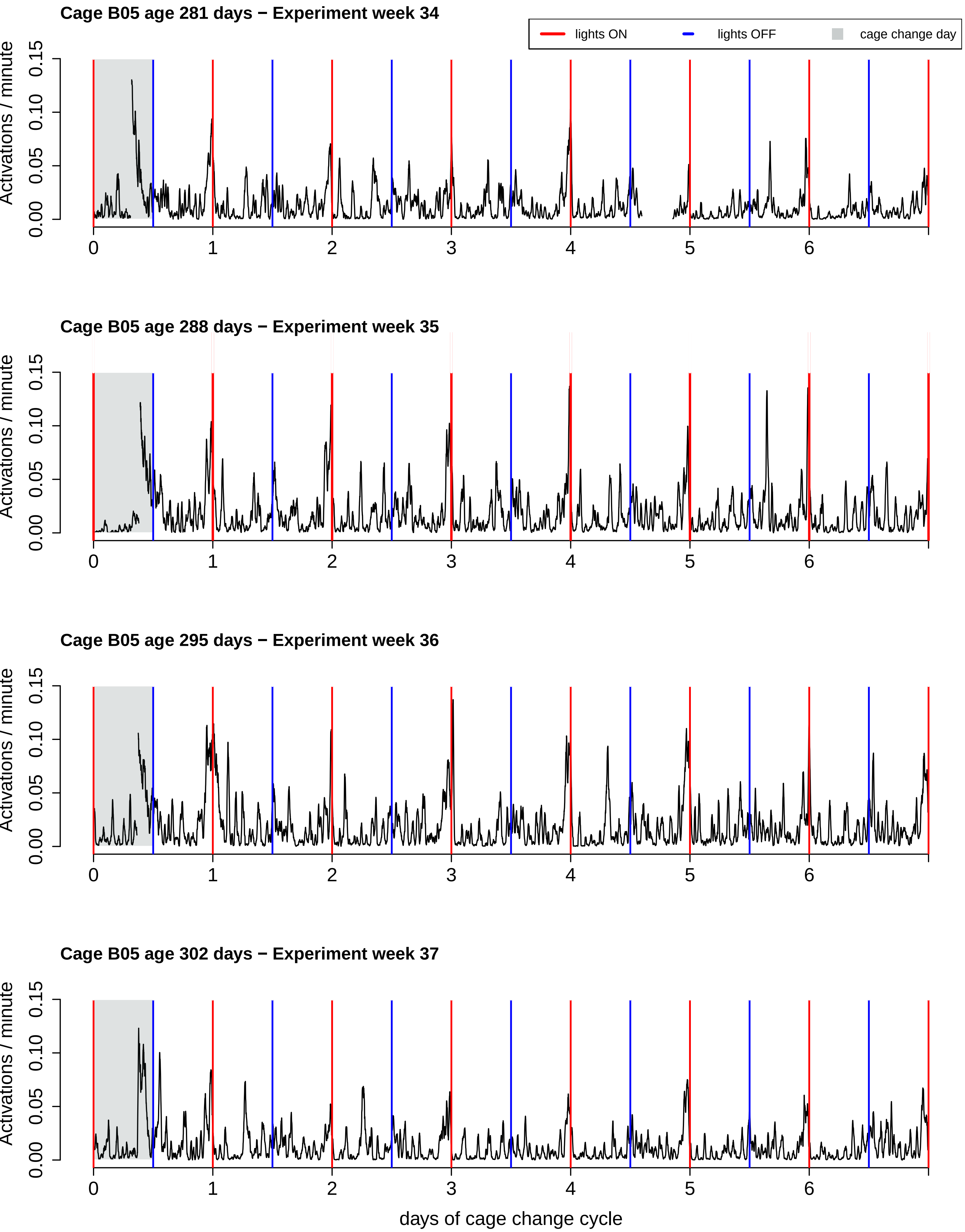

Cage B05 age 281 days − Experiment week 34
lights ON
lights OFF
cage change day
Activations / minute
0.00
0.05
0.10
0.15
0
1
2
3
4
5
6
Cage B05 age 288 days − Experiment week 35
Cage B05 age 295 days − Experiment week 36
Cage B05 age 302 days − Experiment week 37
days of cage change cycle

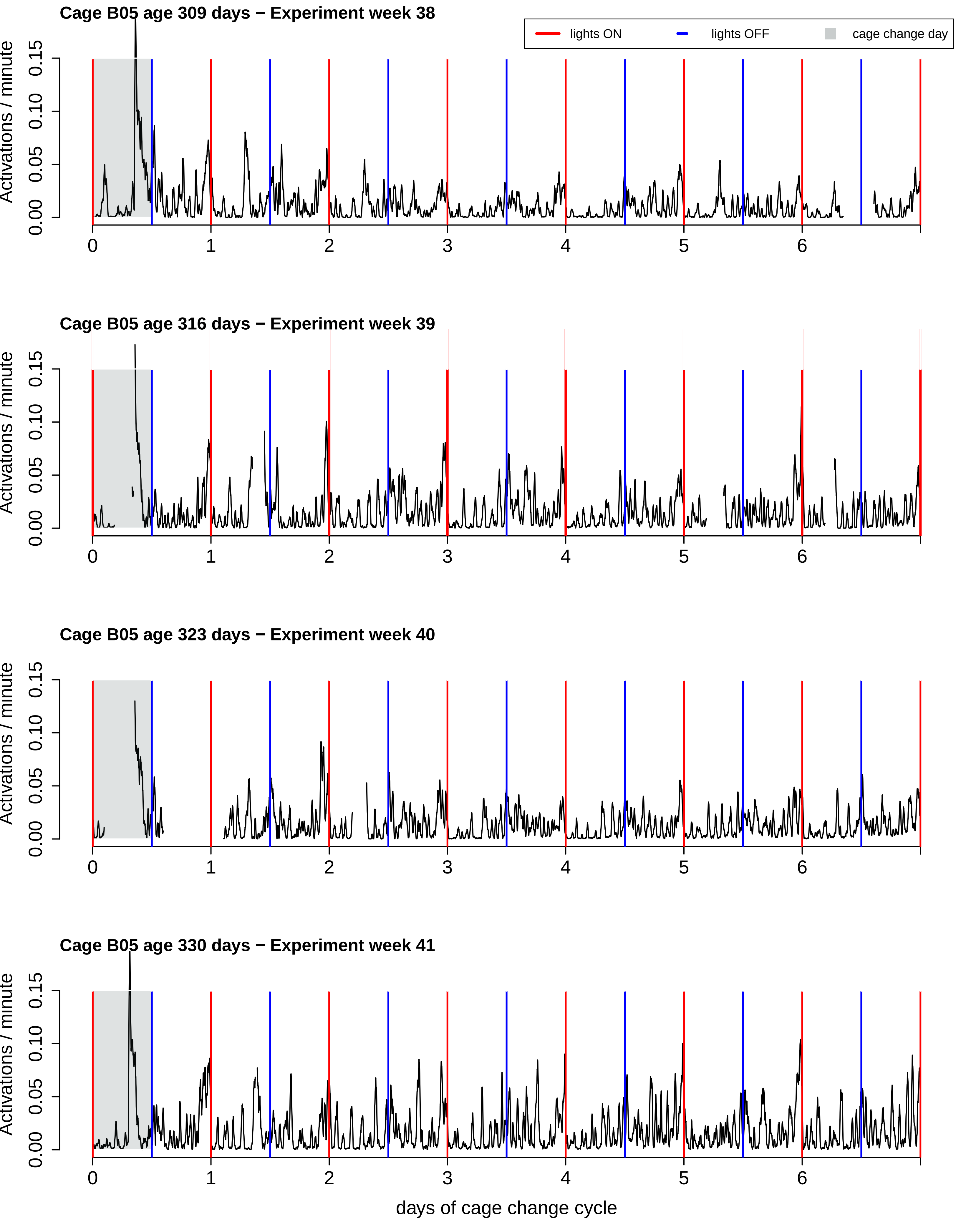

Cage B05 age 309 days − Experiment week 38
lights ON
lights OFF
cage change day
Activations / minute
0.00
0.05
0.10
0.15
0
1
2
3
4
5
6
Cage B05 age 316 days − Experiment week 39
Cage B05 age 323 days − Experiment week 40
Cage B05 age 330 days − Experiment week 41
days of cage change cycle

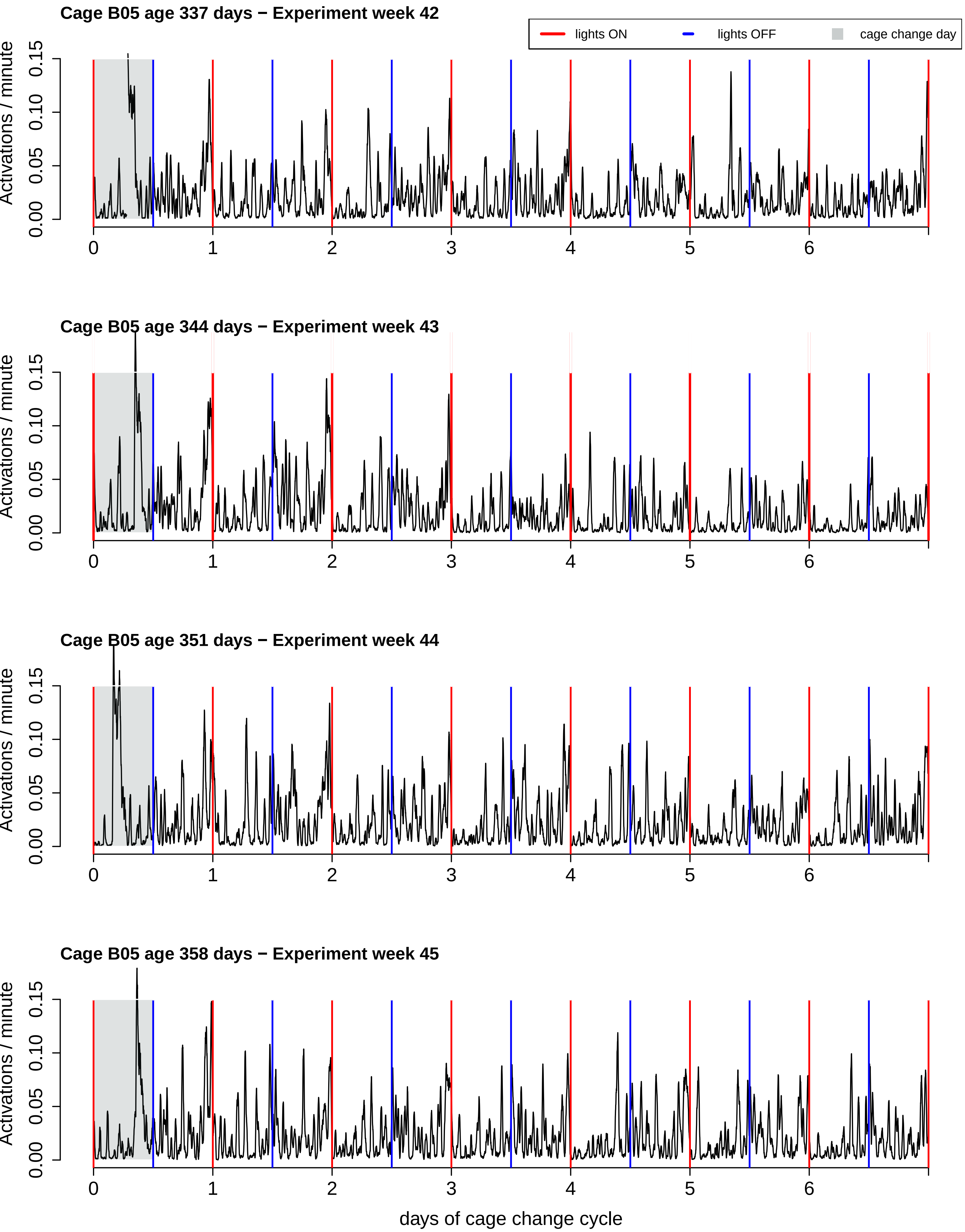

Cage B05 age 337 days − Experiment week 42
lights ON
lights OFF
cage change day
Activations / minute
0.00
0.05
0.10
0.15
0
1
2
3
4
5
6
Cage B05 age 344 days − Experiment week 43
Cage B05 age 351 days − Experiment week 44
Cage B05 age 358 days − Experiment week 45
days of cage change cycle

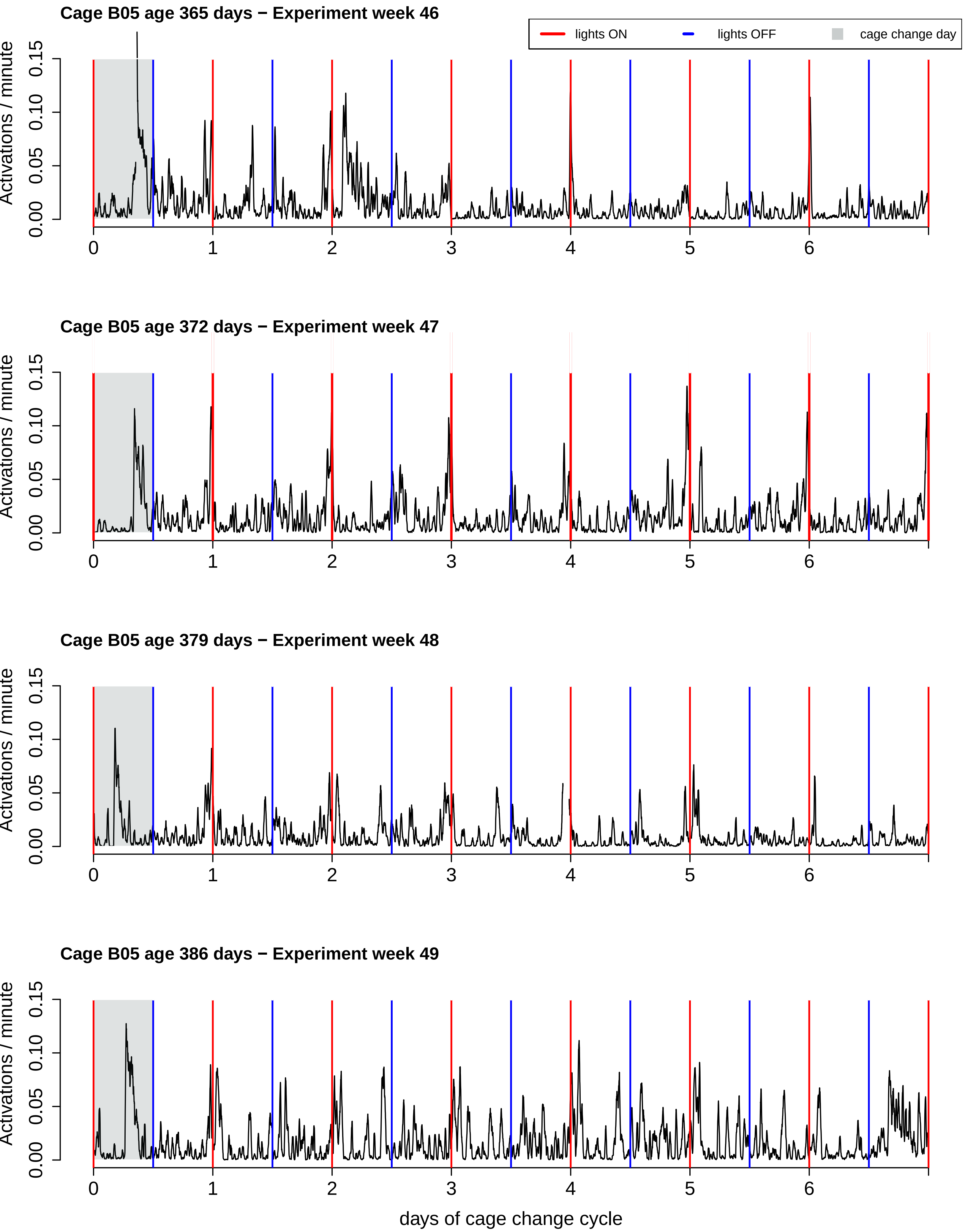

Cage B05 age 365 days − Experiment week 46
lights ON
lights OFF
cage change day
Cage B05 age 372 days − Experiment week 47
Cage B05 age 379 days − Experiment week 48
Cage B05 age 386 days − Experiment week 49
Activations / minute
0.00
0.05
0.10
0.15
0
1
2
3
4
5
6
days of cage change cycle

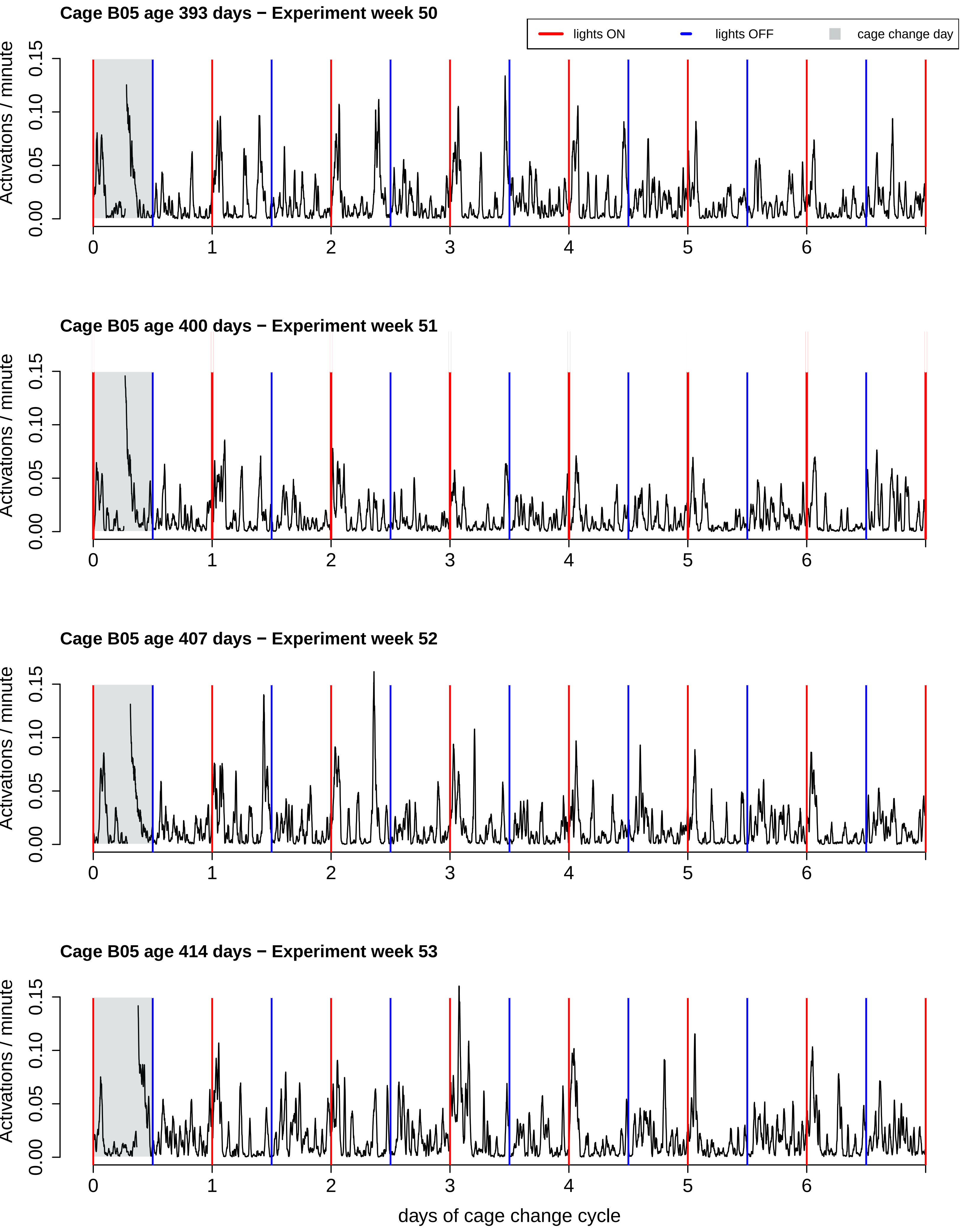
Cage B05 age 393 days − Experiment week 50
lights ON
lights OFF
cage change day
Activations / minute
0.00
0.05
0.10
0.15
0
1
2
3
4
5
6
Cage B05 age 400 days − Experiment week 51
Cage B05 age 407 days − Experiment week 52
Cage B05 age 414 days − Experiment week 53
days of cage change cycle

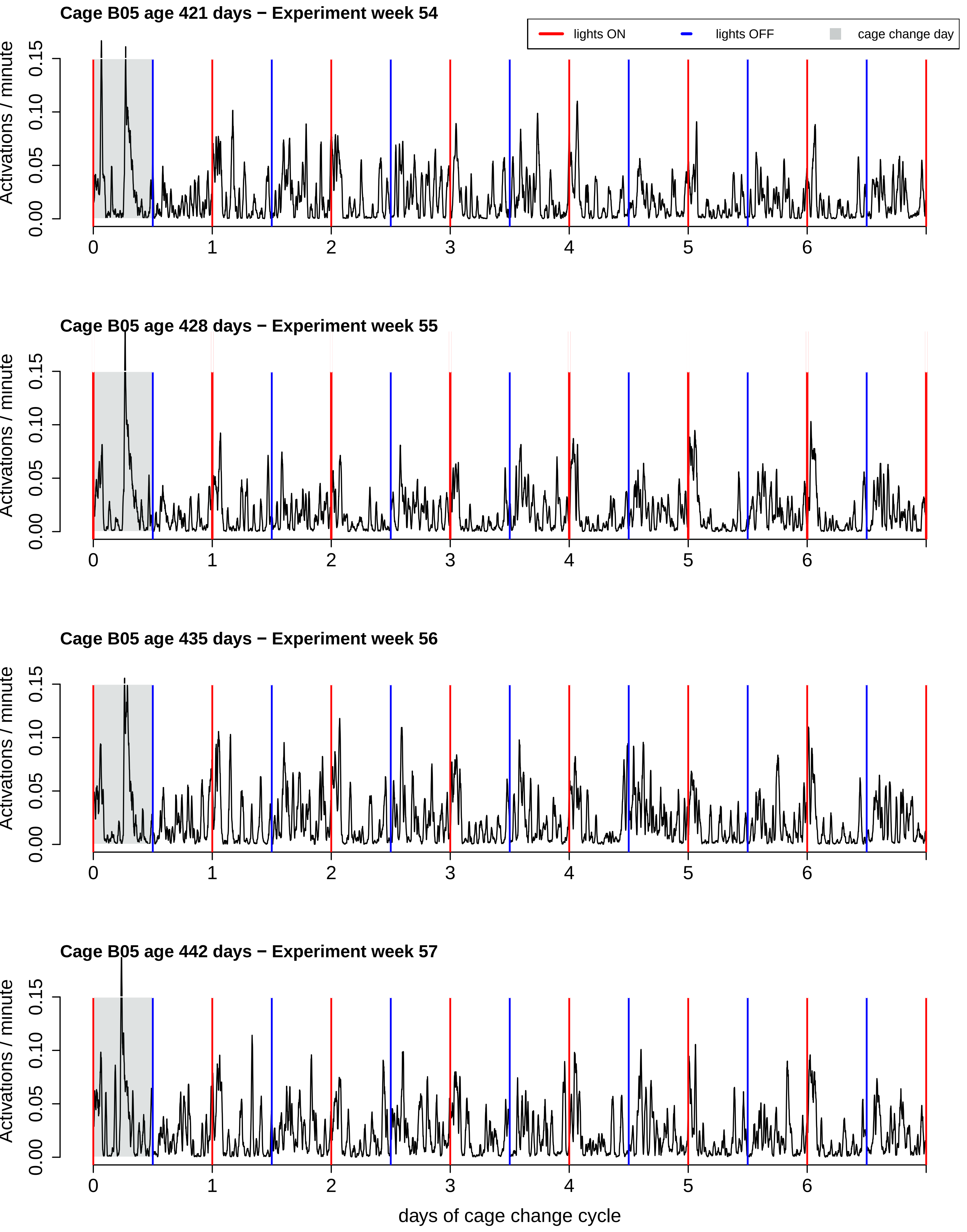
Cage B05 age 421 days − Experiment week 54
lights ON
lights OFF
cage change day
Activations / minute
0.00
0.05
0.10
0.15
0
1
2
3
4
5
6
Cage B05 age 428 days − Experiment week 55
Cage B05 age 435 days − Experiment week 56
Cage B05 age 442 days − Experiment week 57
days of cage change cycle

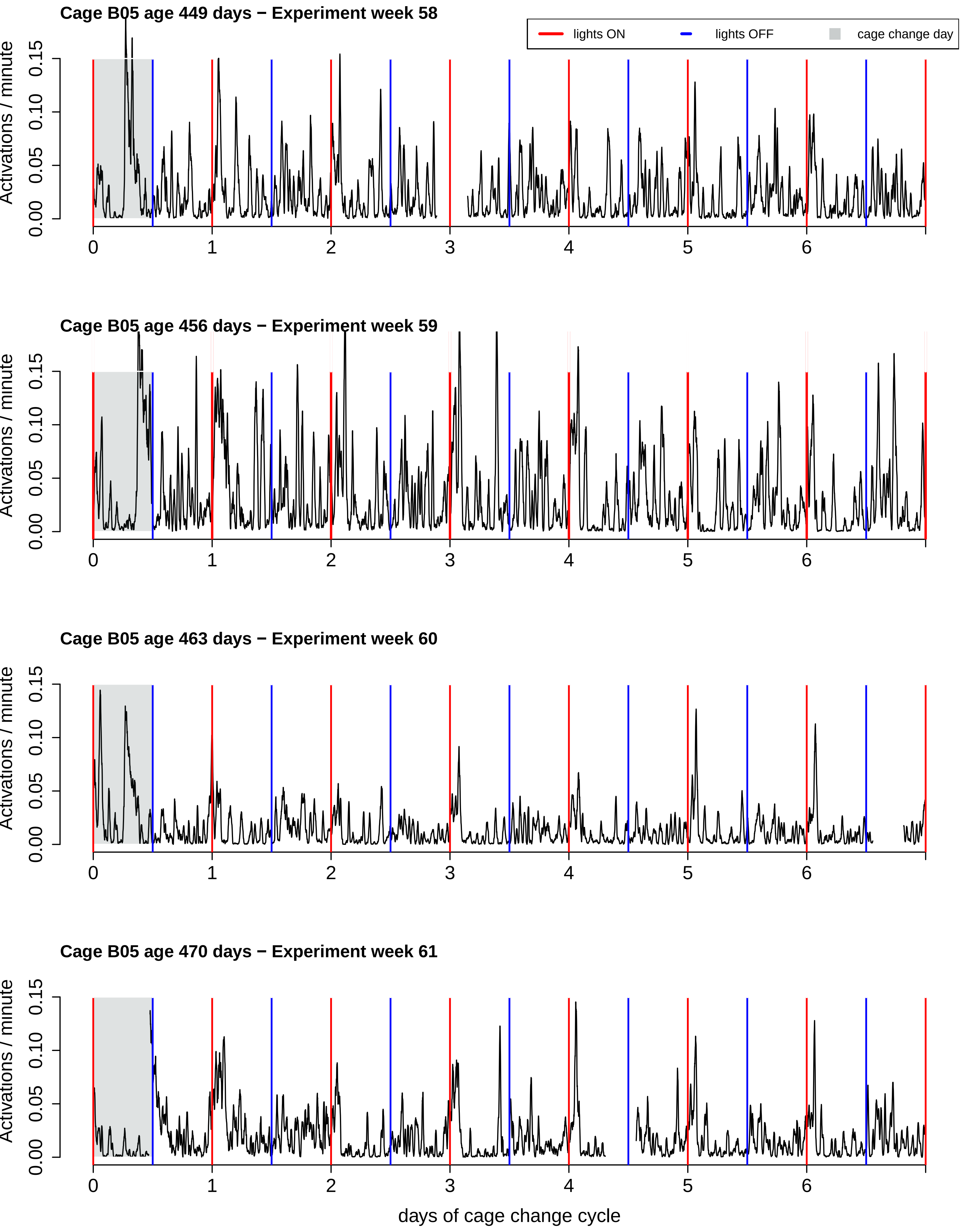
Cage B05 age 449 days − Experiment week 58
lights ON
lights OFF
cage change day
Activations / minute
0.00
0.05
0.10
0.15
0
1
2
3
4
5
6
Cage B05 age 456 days − Experiment week 59
Cage B05 age 463 days − Experiment week 60
Cage B05 age 470 days − Experiment week 61
days of cage change cycle

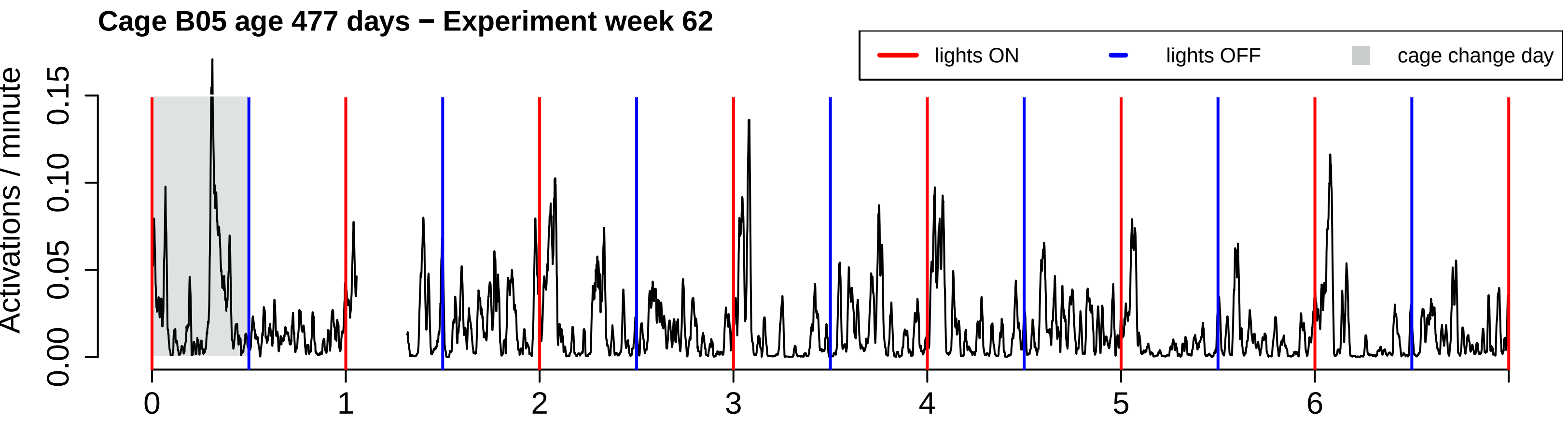

Cage B05 age 477 days − Experiment week 62
lights ON
lights OFF
cage change day
Activations / minute
0.00
0.05
0.10
0.15
0
1
2
3
4
5
6

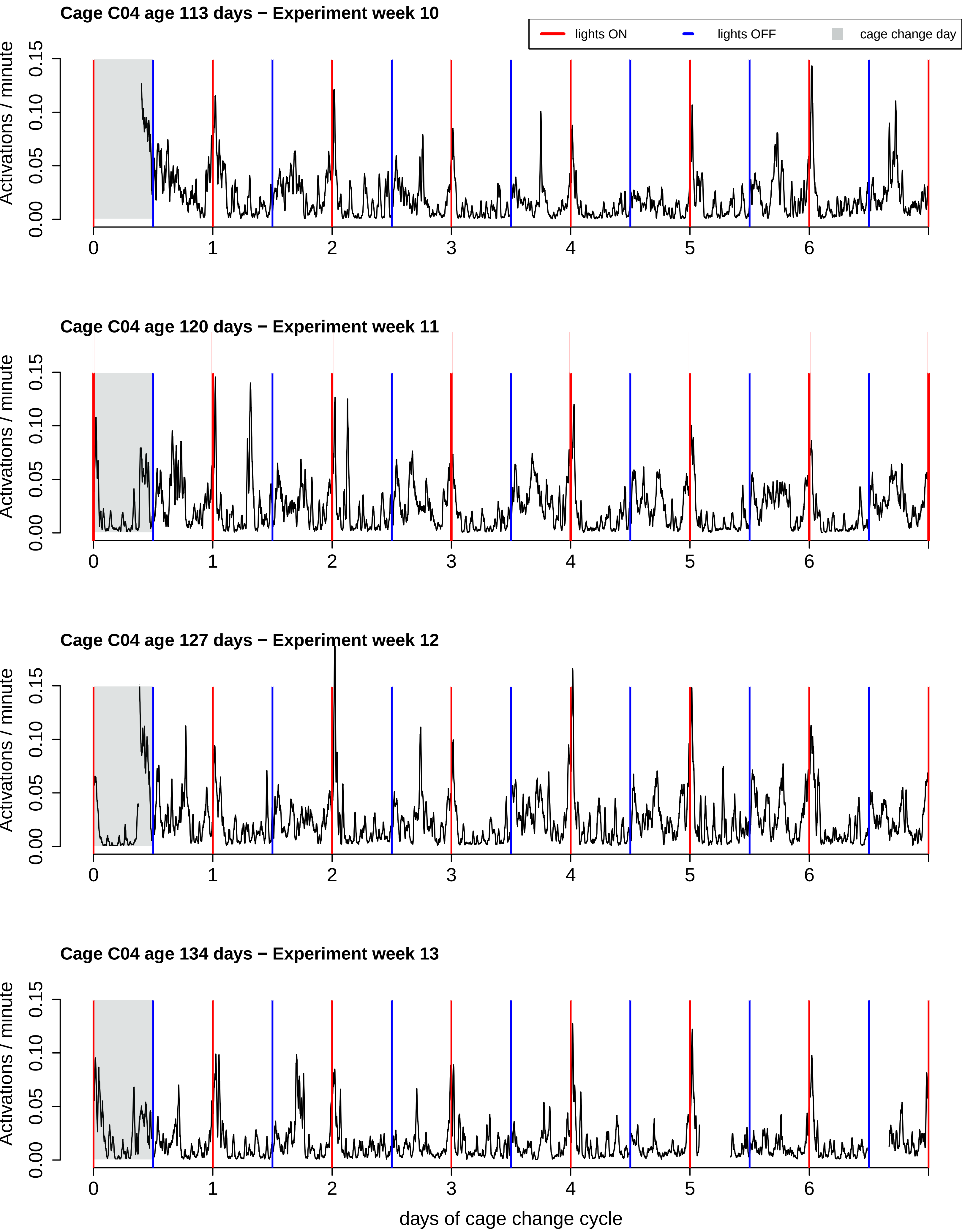
Cage C04 age 113 days − Experiment week 10
lights ON
lights OFF
cage change day
Activations / minute
0.00
0.05
0.10
0.15
0
1
2
3
4
5
6
Cage C04 age 120 days − Experiment week 11
Cage C04 age 127 days − Experiment week 12
Cage C04 age 134 days − Experiment week 13
days of cage change cycle

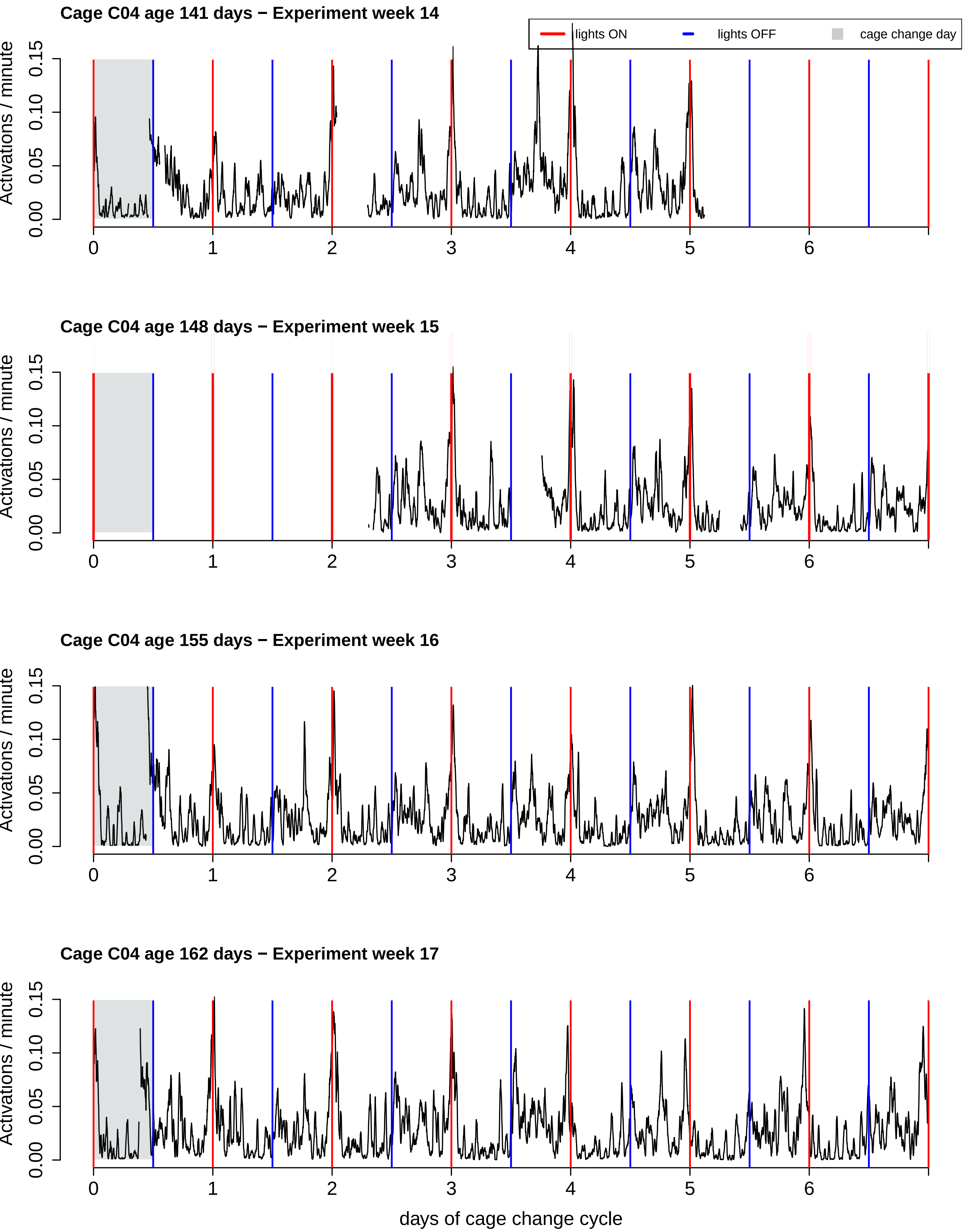

Cage C04 age 141 days − Experiment week 14
lights ON
lights OFF
cage change day
Cage C04 age 148 days − Experiment week 15
Cage C04 age 155 days − Experiment week 16
Cage C04 age 162 days − Experiment week 17
Activations / minute
0.00
0.05
0.10
0.15
0
1
2
3
4
5
6
days of cage change cycle

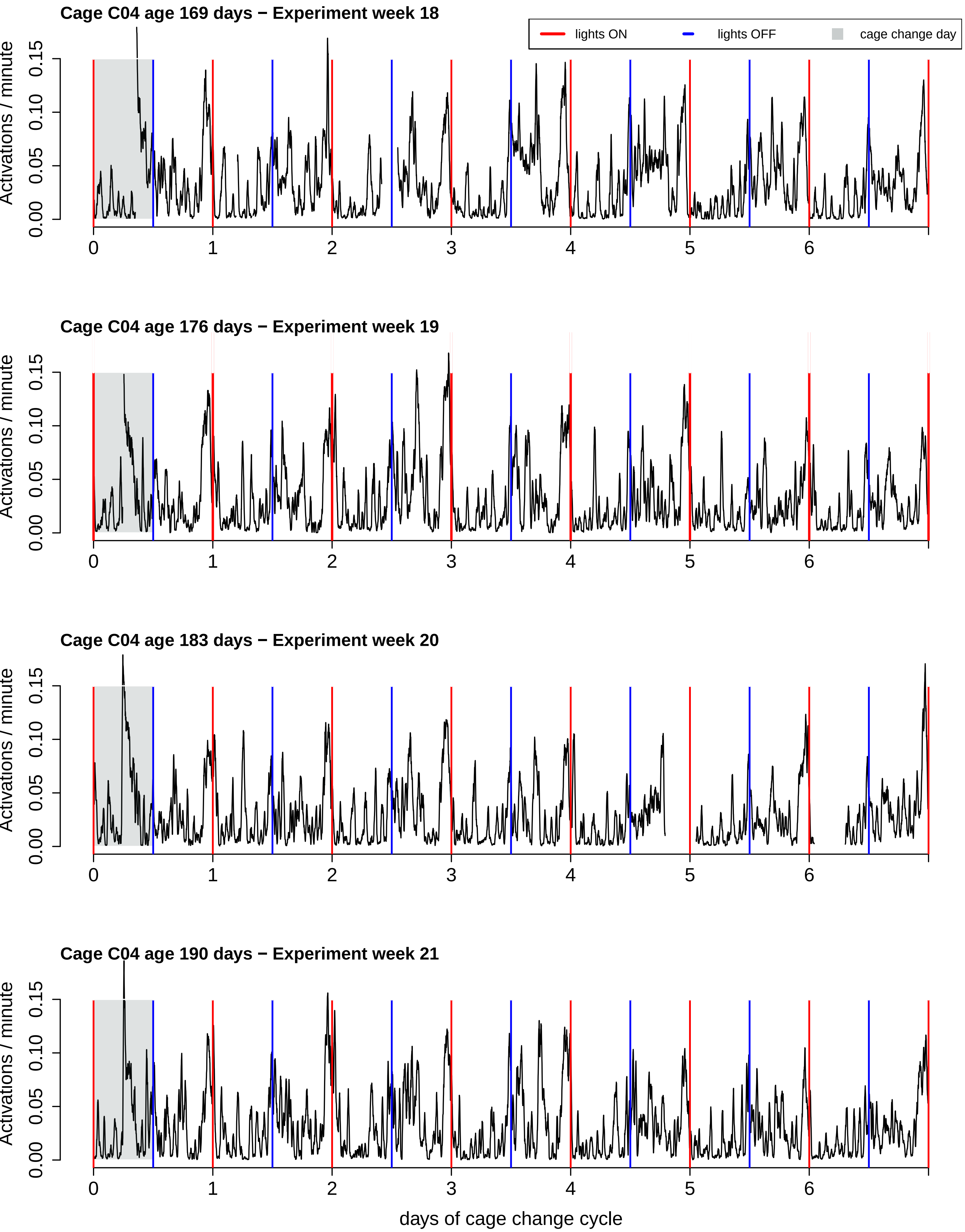
Cage C04 age 169 days − Experiment week 18
lights ON
lights OFF
cage change day
Cage C04 age 176 days − Experiment week 19
Cage C04 age 183 days − Experiment week 20
Cage C04 age 190 days − Experiment week 21
Activations / minute
0.00
0.05
0.10
0.15
0
1
2
3
4
5
6
days of cage change cycle

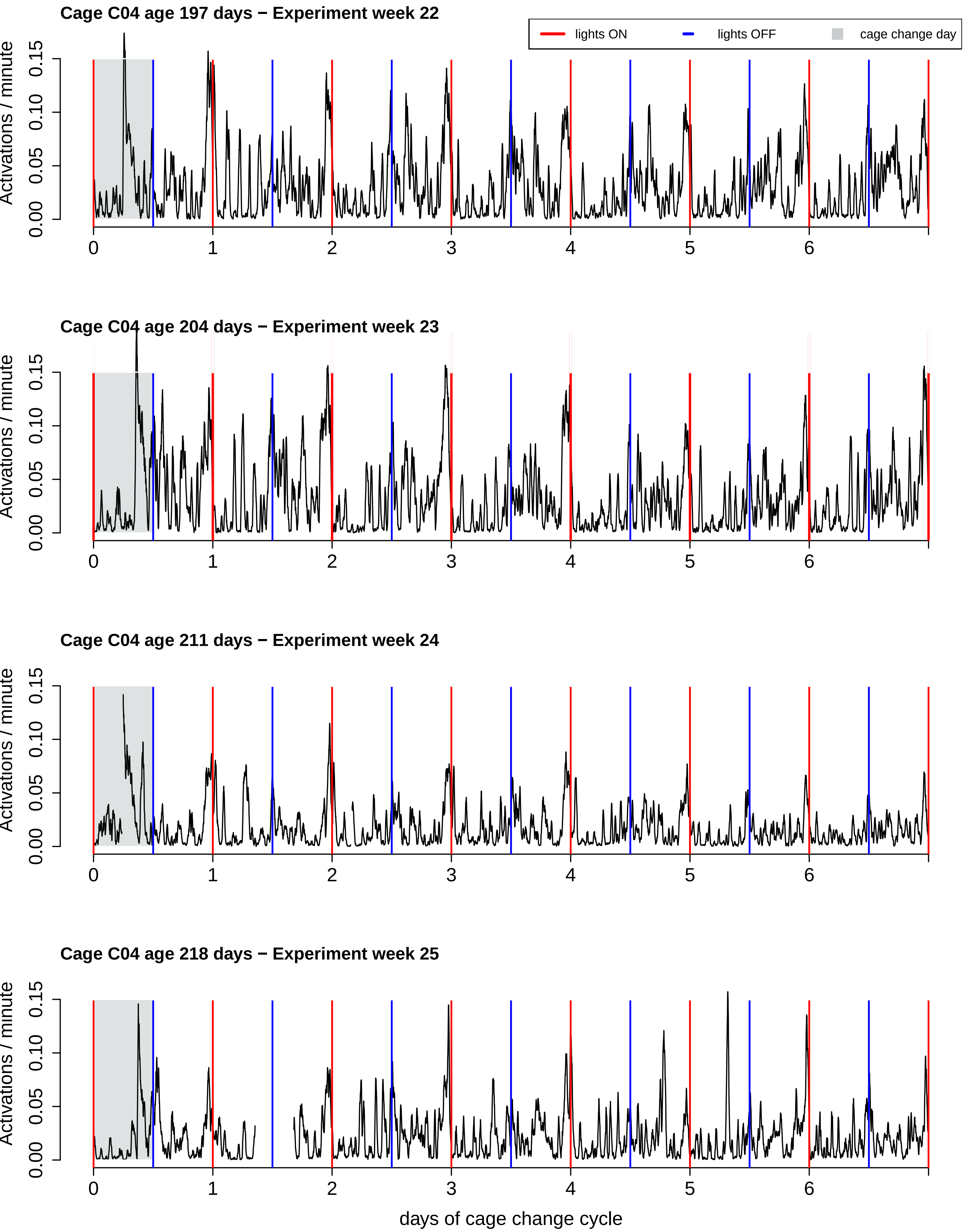

Cage C04 age 197 days − Experiment week 22
lights ON
lights OFF
cage change day
Activations / minute
0.00
0.05
0.10
0.15
0
1
2
3
4
5
6
Cage C04 age 204 days − Experiment week 23
Cage C04 age 211 days − Experiment week 24
Cage C04 age 218 days − Experiment week 25
days of cage change cycle

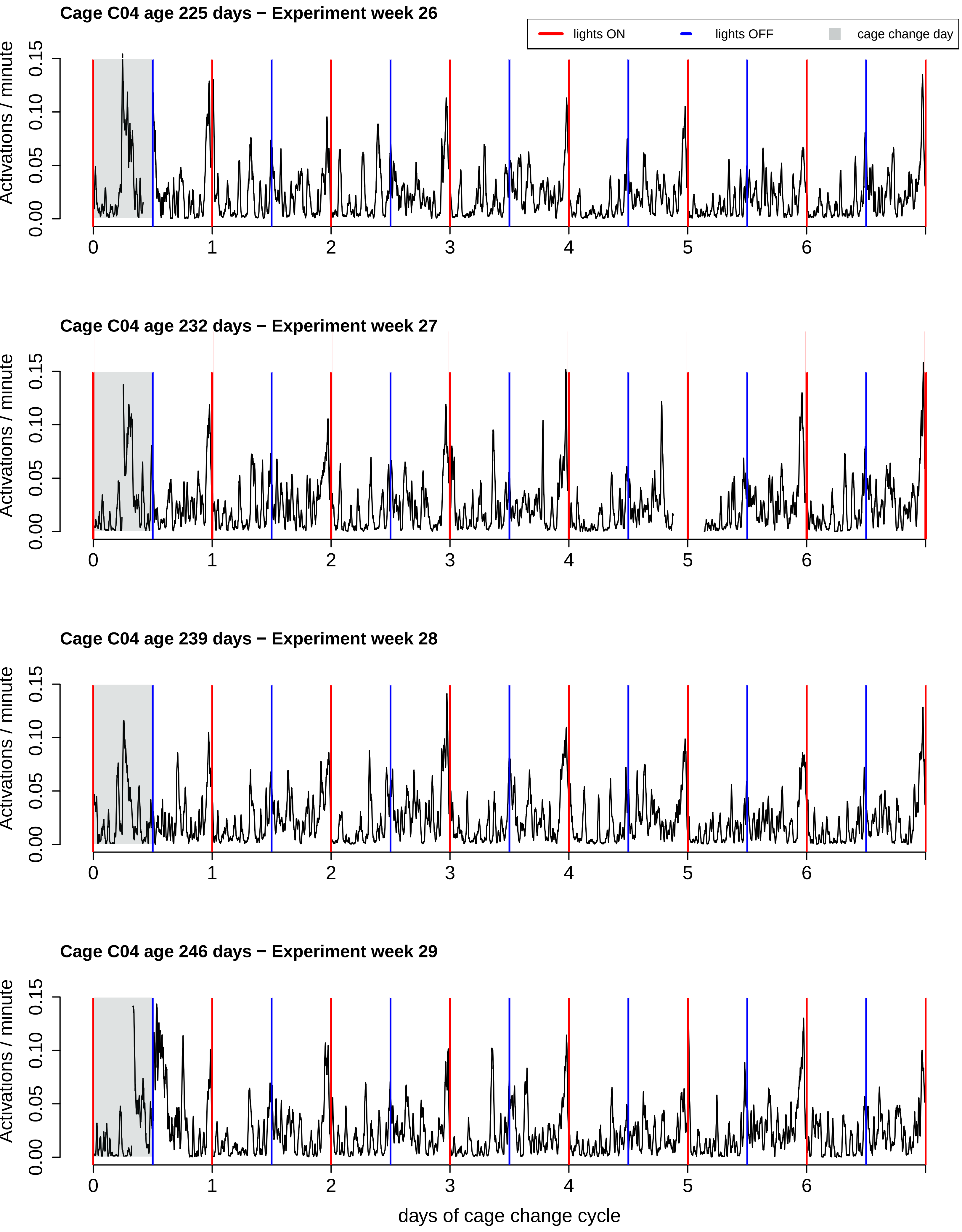
Cage C04 age 225 days − Experiment week 26
lights ON
lights OFF
cage change day
Activations / minute
0.00
0.05
0.10
0.15
0
1
2
3
4
5
6
Cage C04 age 232 days − Experiment week 27
Cage C04 age 239 days − Experiment week 28
Cage C04 age 246 days − Experiment week 29
days of cage change cycle

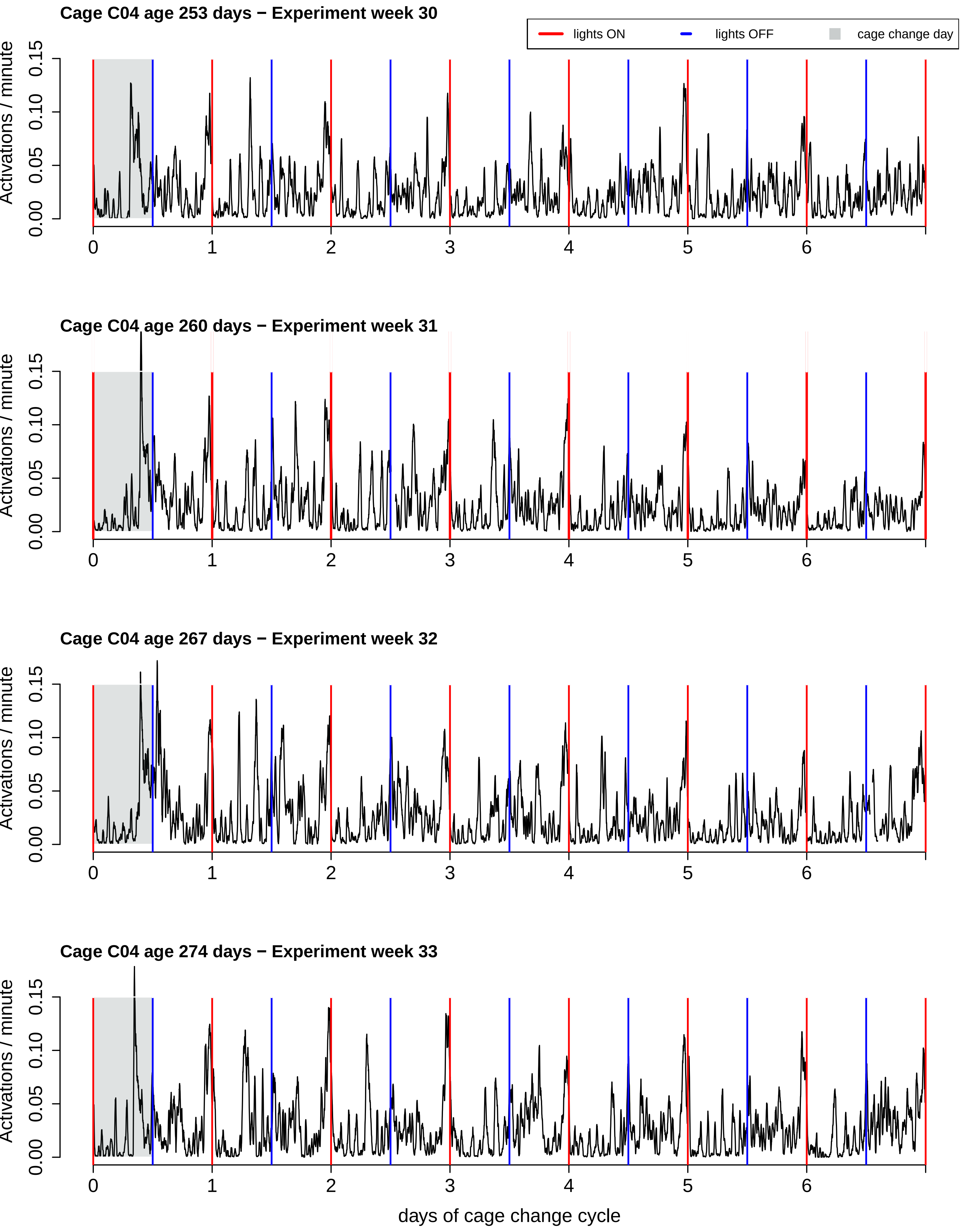

Cage C04 age 253 days − Experiment week 30
lights ON
lights OFF
cage change day
Activations / minute
0.00
0.05
0.10
0.15
0
1
2
3
4
5
6
Cage C04 age 260 days − Experiment week 31
Cage C04 age 267 days − Experiment week 32
Cage C04 age 274 days − Experiment week 33
days of cage change cycle

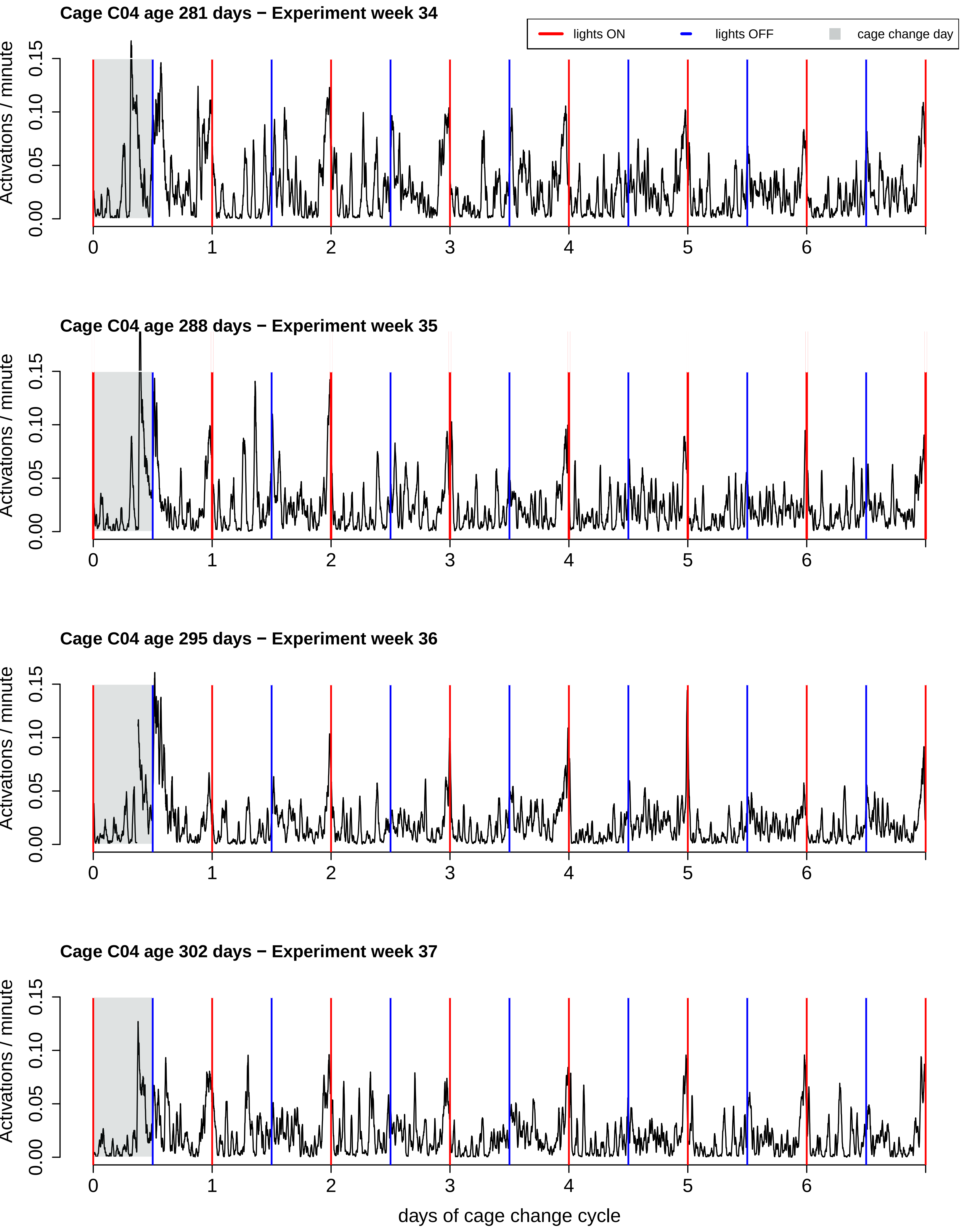
Cage C04 age 281 days − Experiment week 34
lights ON
lights OFF
cage change day
Cage C04 age 288 days − Experiment week 35
Cage C04 age 295 days − Experiment week 36
Cage C04 age 302 days − Experiment week 37
Activations / minute
0.00
0.05
0.10
0.15
0
1
2
3
4
5
6
days of cage change cycle

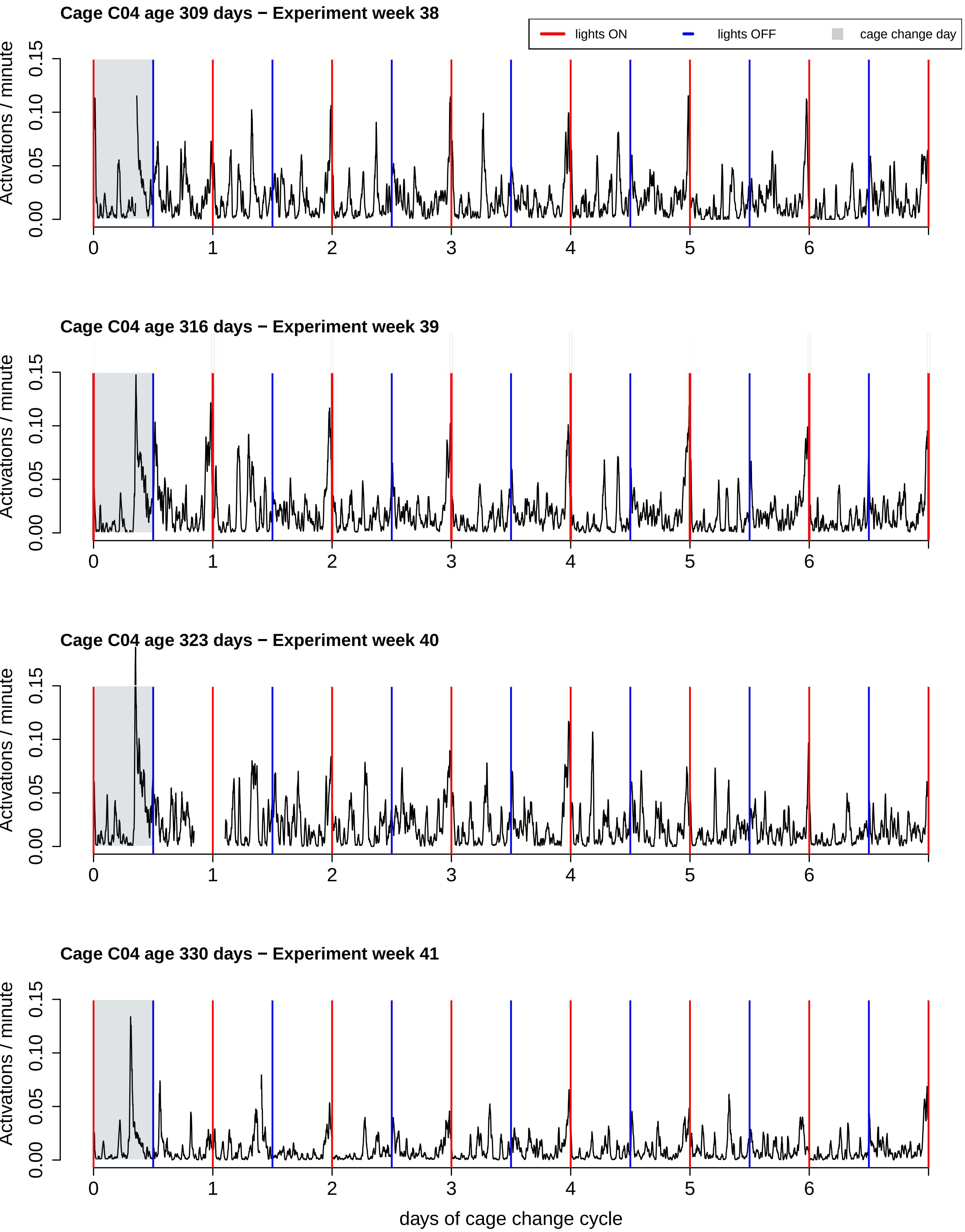
Cage C04 age 309 days − Experiment week 38
lights ON
lights OFF
cage change day
Cage C04 age 316 days − Experiment week 39
Cage C04 age 323 days − Experiment week 40
Cage C04 age 330 days − Experiment week 41
Activations / minute
0.00
0.05
0.10
0.15
0
1
2
3
4
5
6
days of cage change cycle

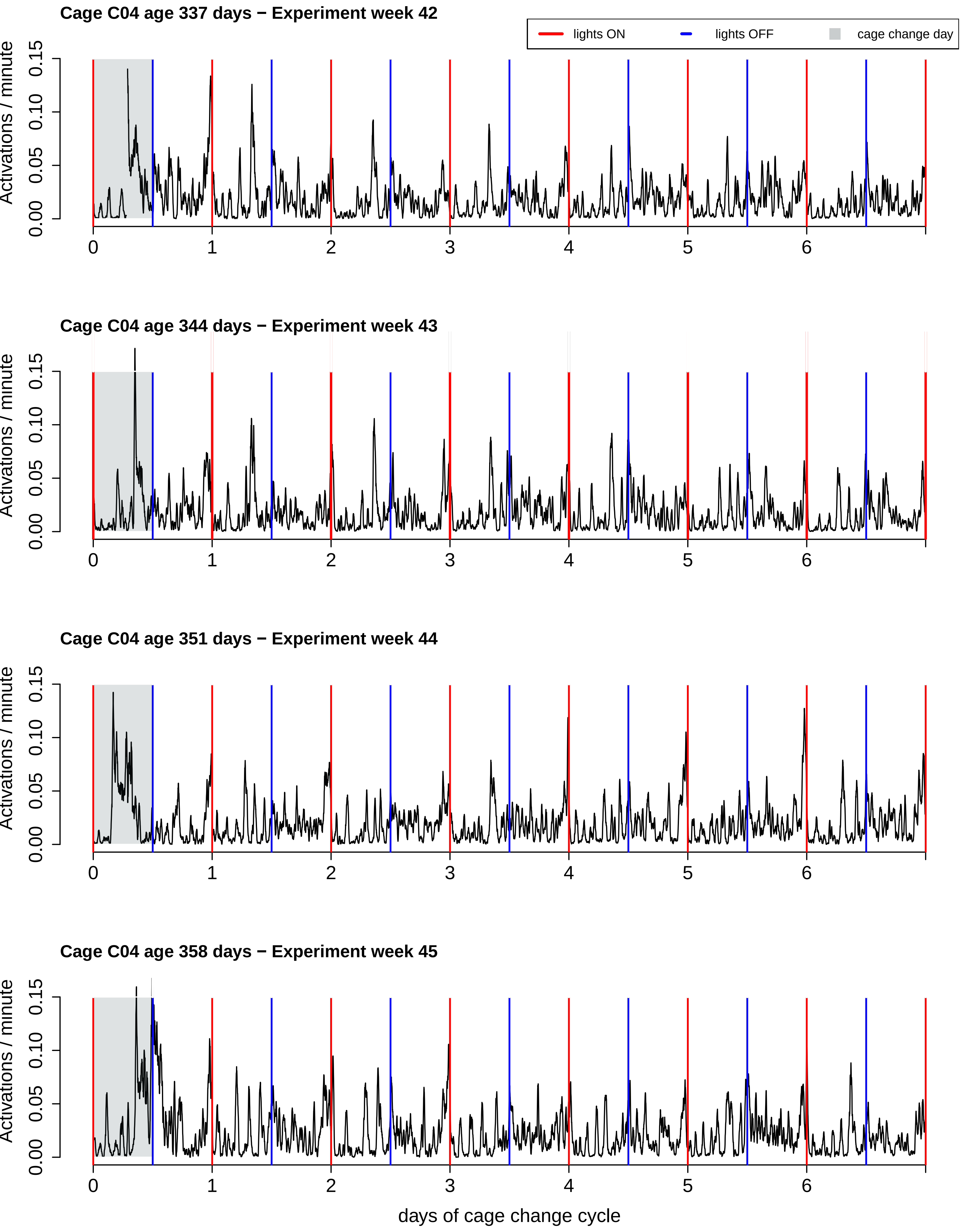
Cage C04 age 337 days − Experiment week 42
lights ON
lights OFF
cage change day
Activations / minute
0.00
0.05
0.10
0.15
0
1
2
3
4
5
6
Cage C04 age 344 days − Experiment week 43
Cage C04 age 351 days − Experiment week 44
Cage C04 age 358 days − Experiment week 45
days of cage change cycle

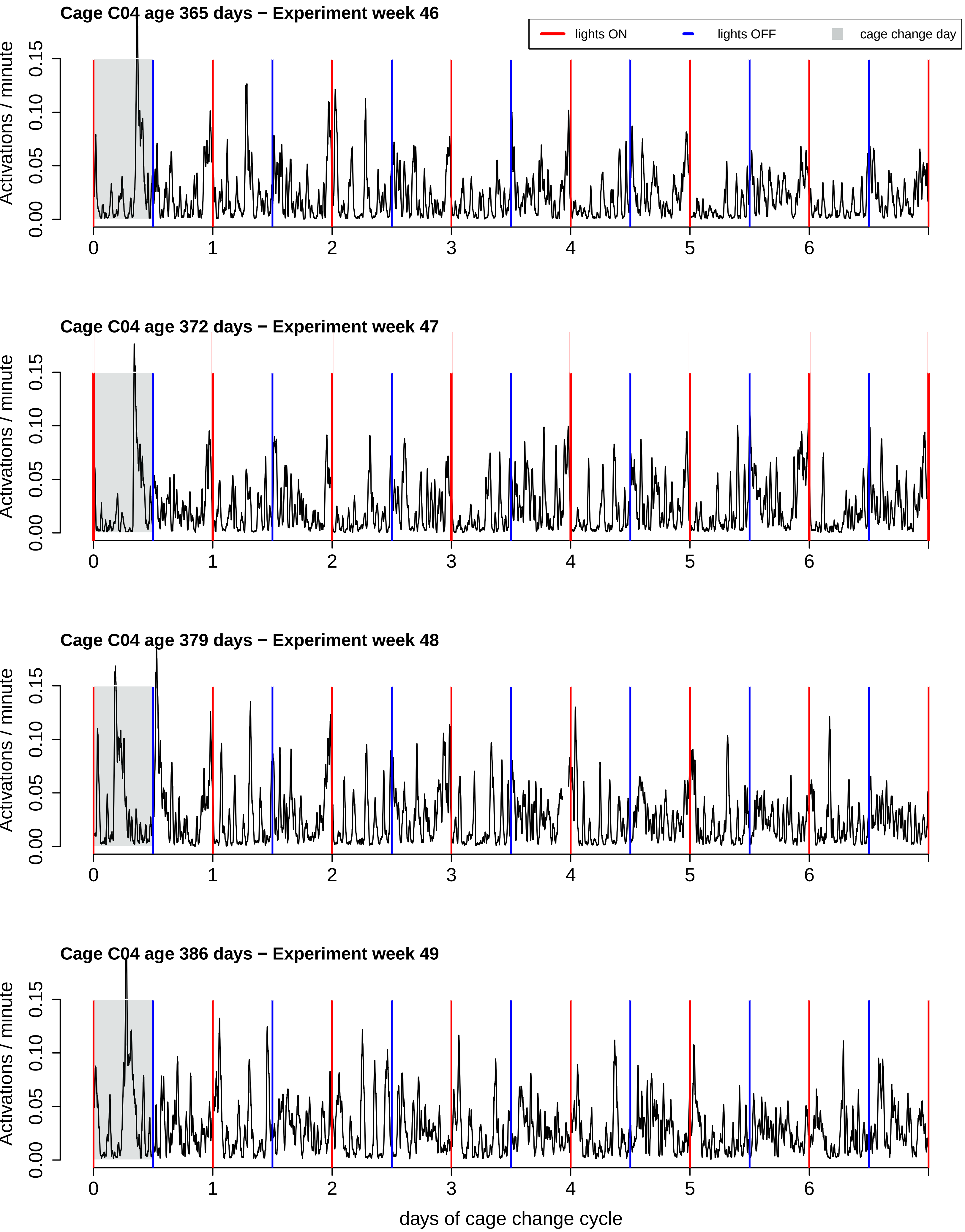
Cage C04 age 365 days − Experiment week 46
lights ON
lights OFF
cage change day
Activations / minute
0.00
0.05
0.10
0.15
0
1
2
3
4
5
6
Cage C04 age 372 days − Experiment week 47
Cage C04 age 379 days − Experiment week 48
Cage C04 age 386 days − Experiment week 49
days of cage change cycle

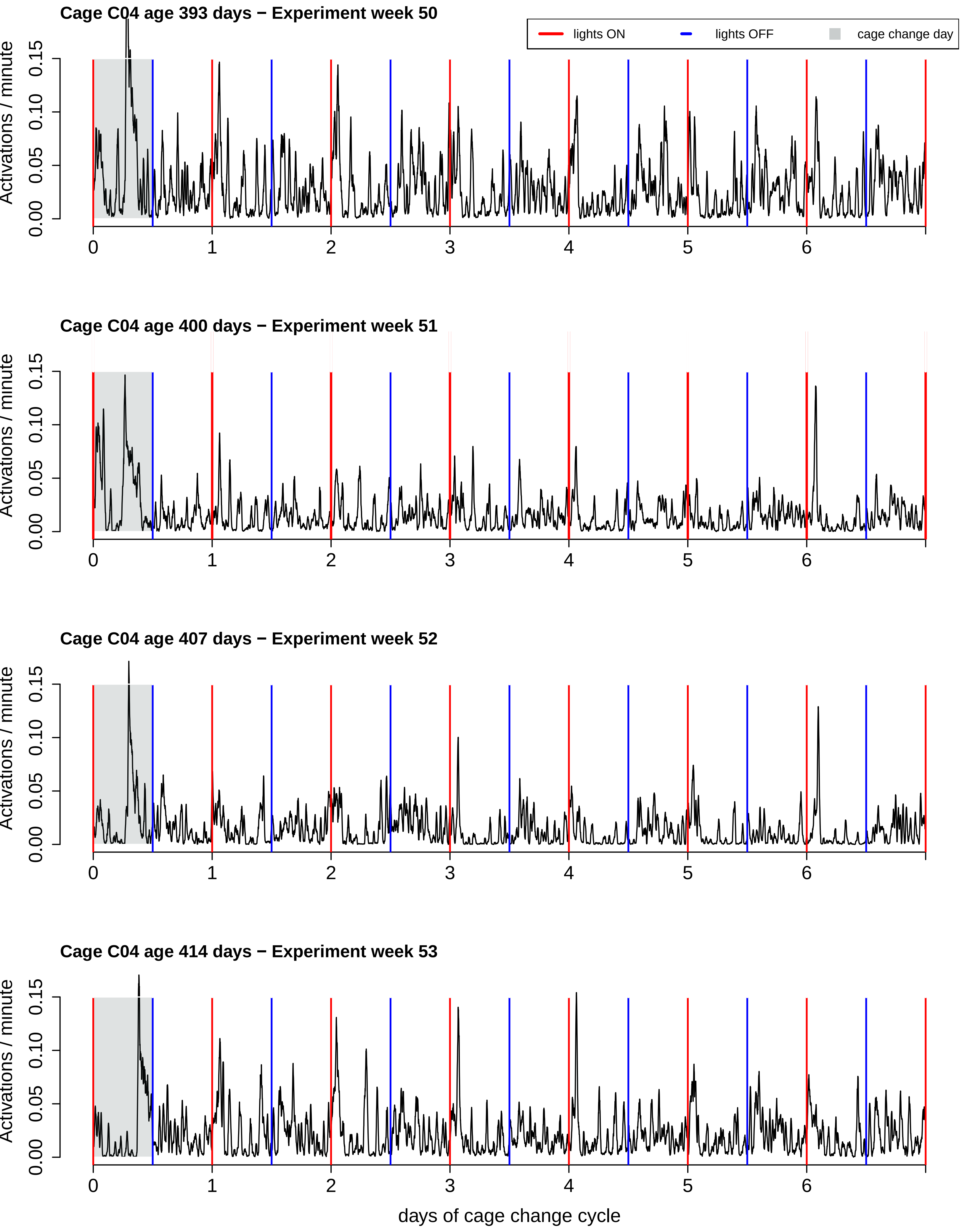
Cage C04 age 393 days − Experiment week 50
lights ON
lights OFF
cage change day
Activations / minute
0.00 0.05 0.10 0.15
0 1 2 3 4 5 6
Cage C04 age 400 days − Experiment week 51
Cage C04 age 407 days − Experiment week 52
Cage C04 age 414 days − Experiment week 53
days of cage change cycle

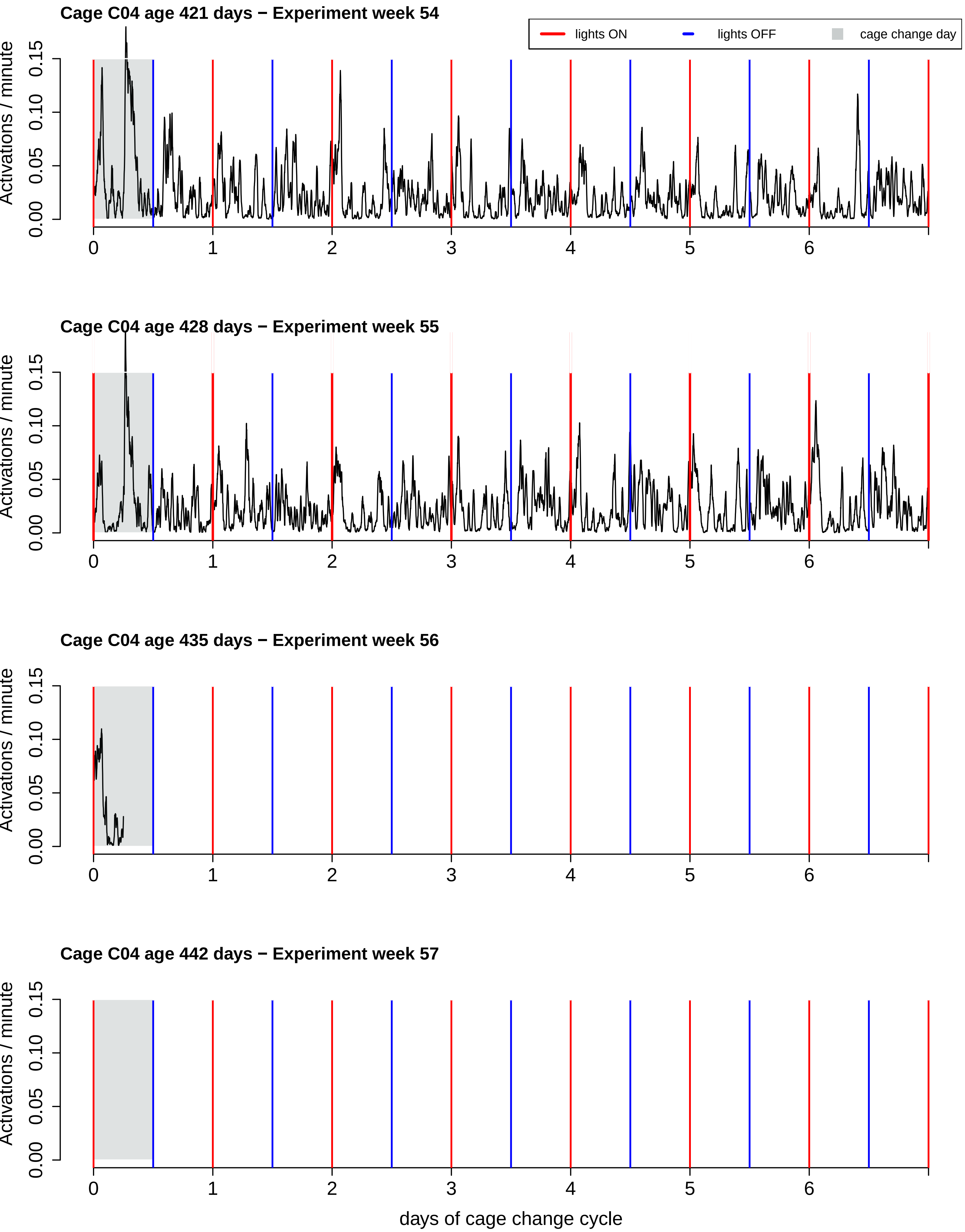

Cage C04 age 421 days − Experiment week 54
lights ON
lights OFF
cage change day
Activations / minute
0.00
0.05
0.10
0.15
0
1
2
3
4
5
6
Cage C04 age 428 days − Experiment week 55
Cage C04 age 435 days − Experiment week 56
Cage C04 age 442 days − Experiment week 57
days of cage change cycle

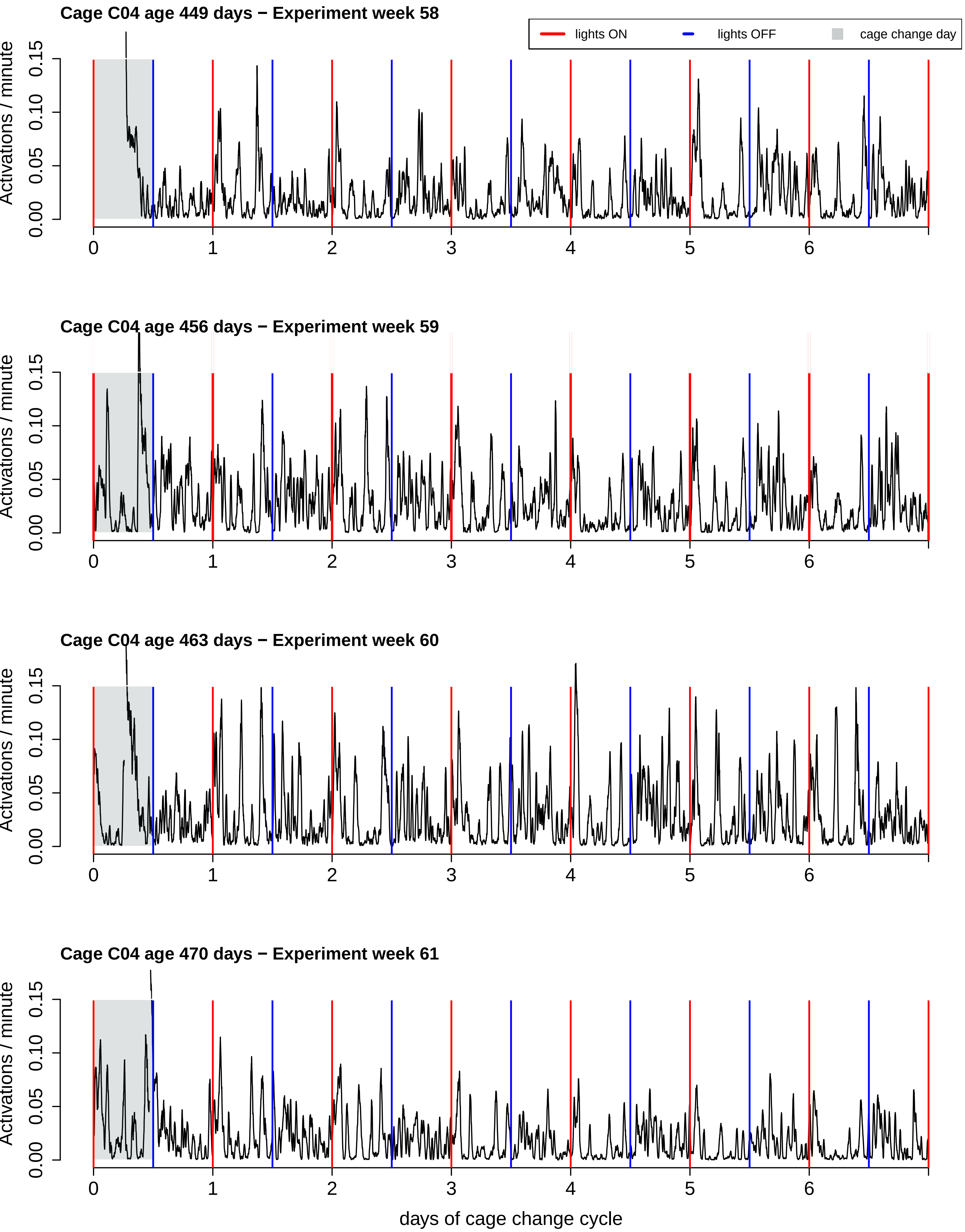

Cage C04 age 449 days − Experiment week 58
lights ON
lights OFF
cage change day
Activations / minute
0.00
0.05
0.10
0.15
0
1
2
3
4
5
6
Cage C04 age 456 days − Experiment week 59
Cage C04 age 463 days − Experiment week 60
Cage C04 age 470 days − Experiment week 61
days of cage change cycle

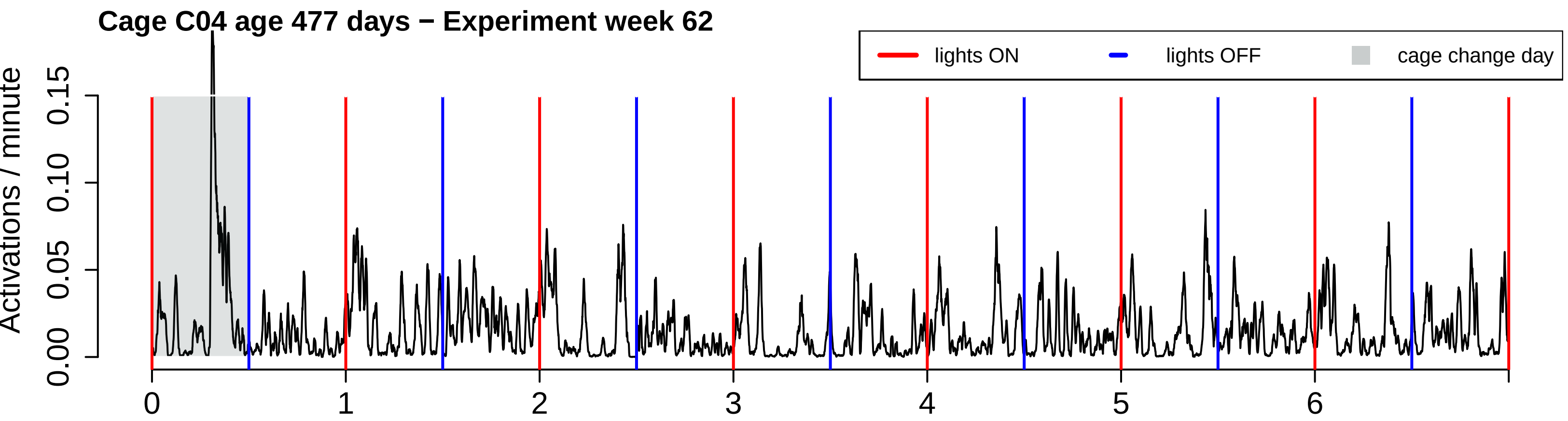

Cage C04 age 477 days − Experiment week 62
lights ON
lights OFF
cage change day
Activations / minute
0.00
0.05
0.10
0.15
0
1
2
3
4
5
6

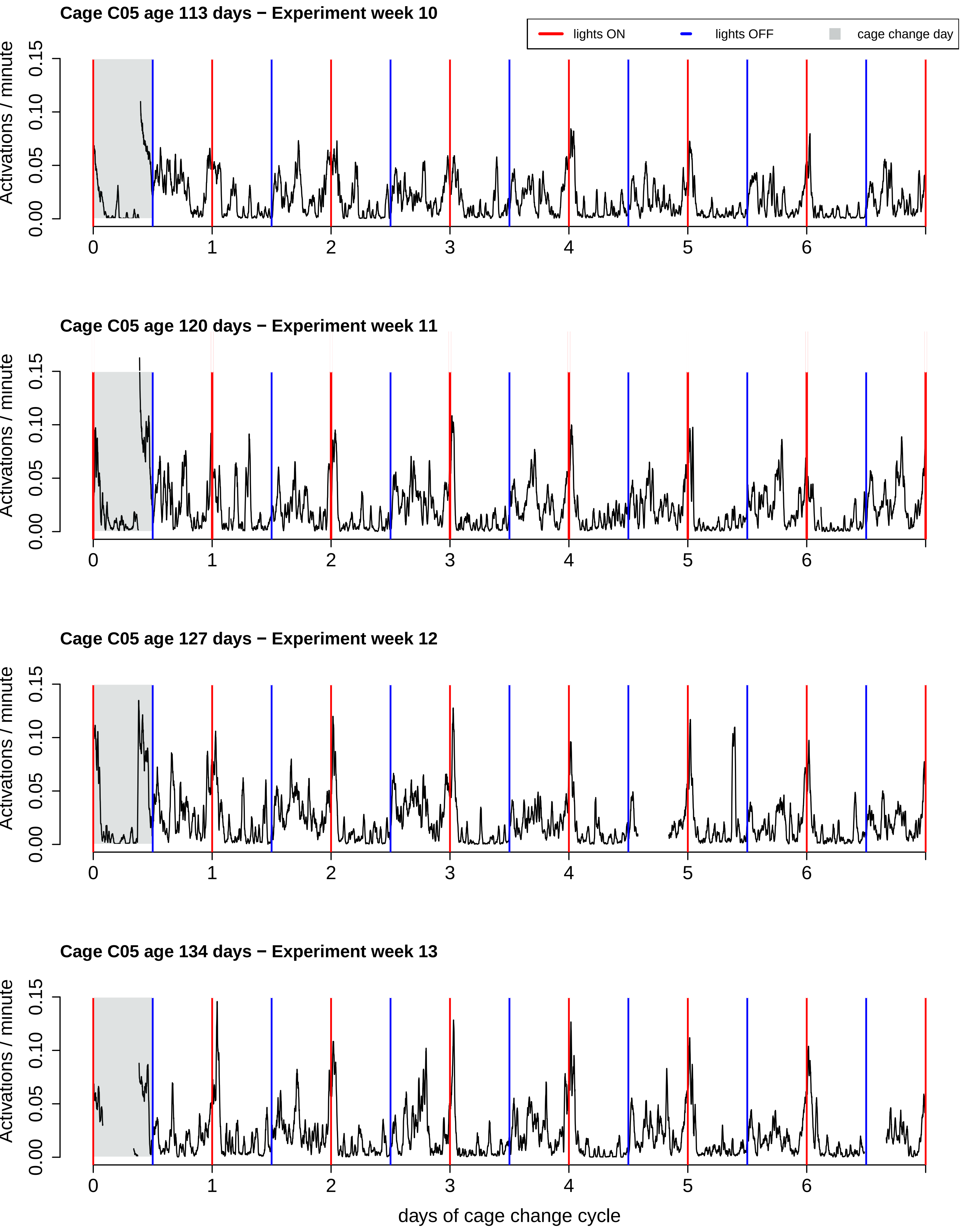

Cage C05 age 113 days − Experiment week 10
lights ON
lights OFF
cage change day
Activations / minute
0.00
0.05
0.10
0.15
0 1 2 3 4 5 6
Cage C05 age 120 days − Experiment week 11
Cage C05 age 127 days − Experiment week 12
Cage C05 age 134 days − Experiment week 13
days of cage change cycle

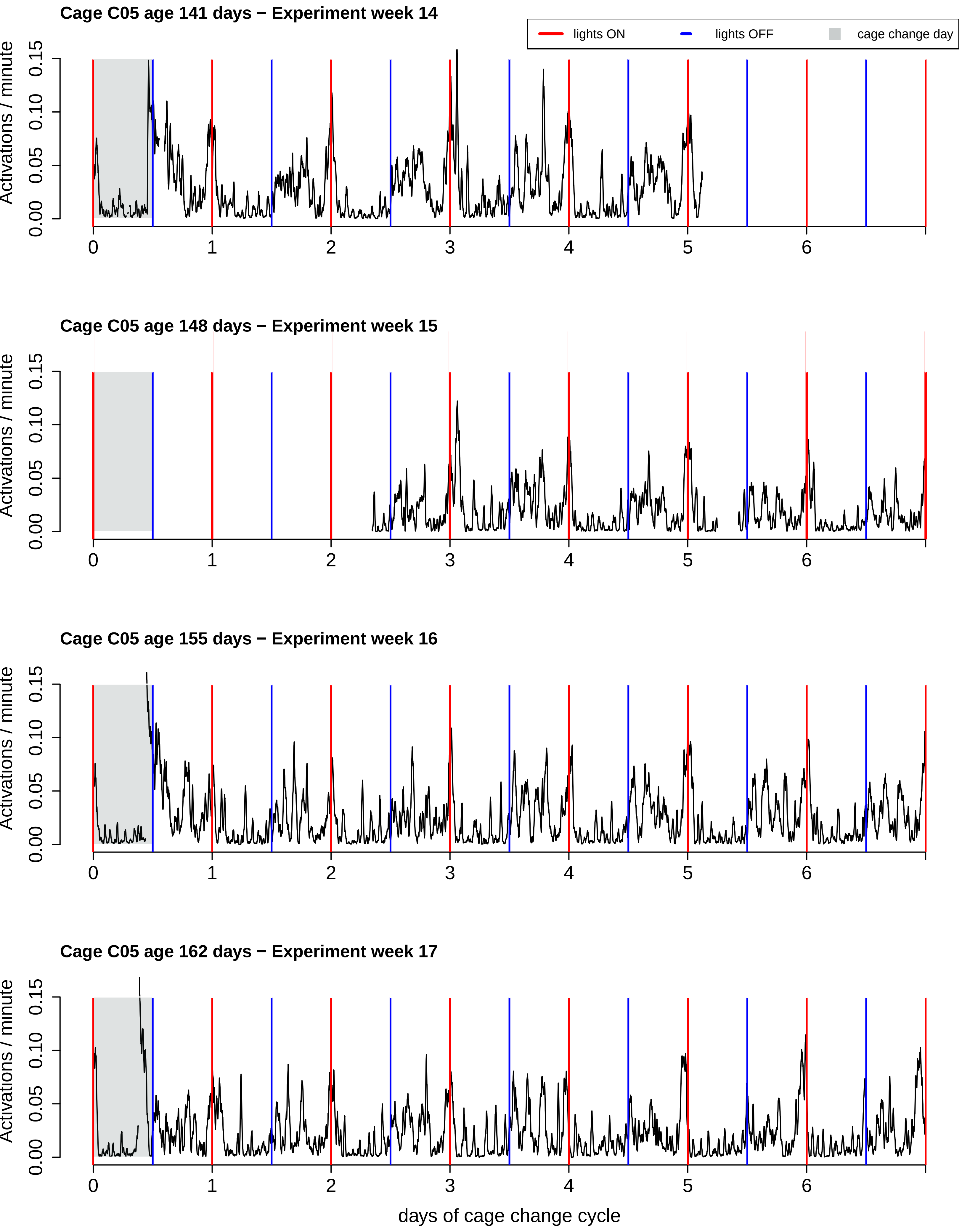

Cage C05 age 141 days − Experiment week 14
lights ON
lights OFF
cage change day
Activations / minute
0.00
0.05
0.10
0.15
0
1
2
3
4
5
6
Cage C05 age 148 days − Experiment week 15
Cage C05 age 155 days − Experiment week 16
Cage C05 age 162 days − Experiment week 17
days of cage change cycle

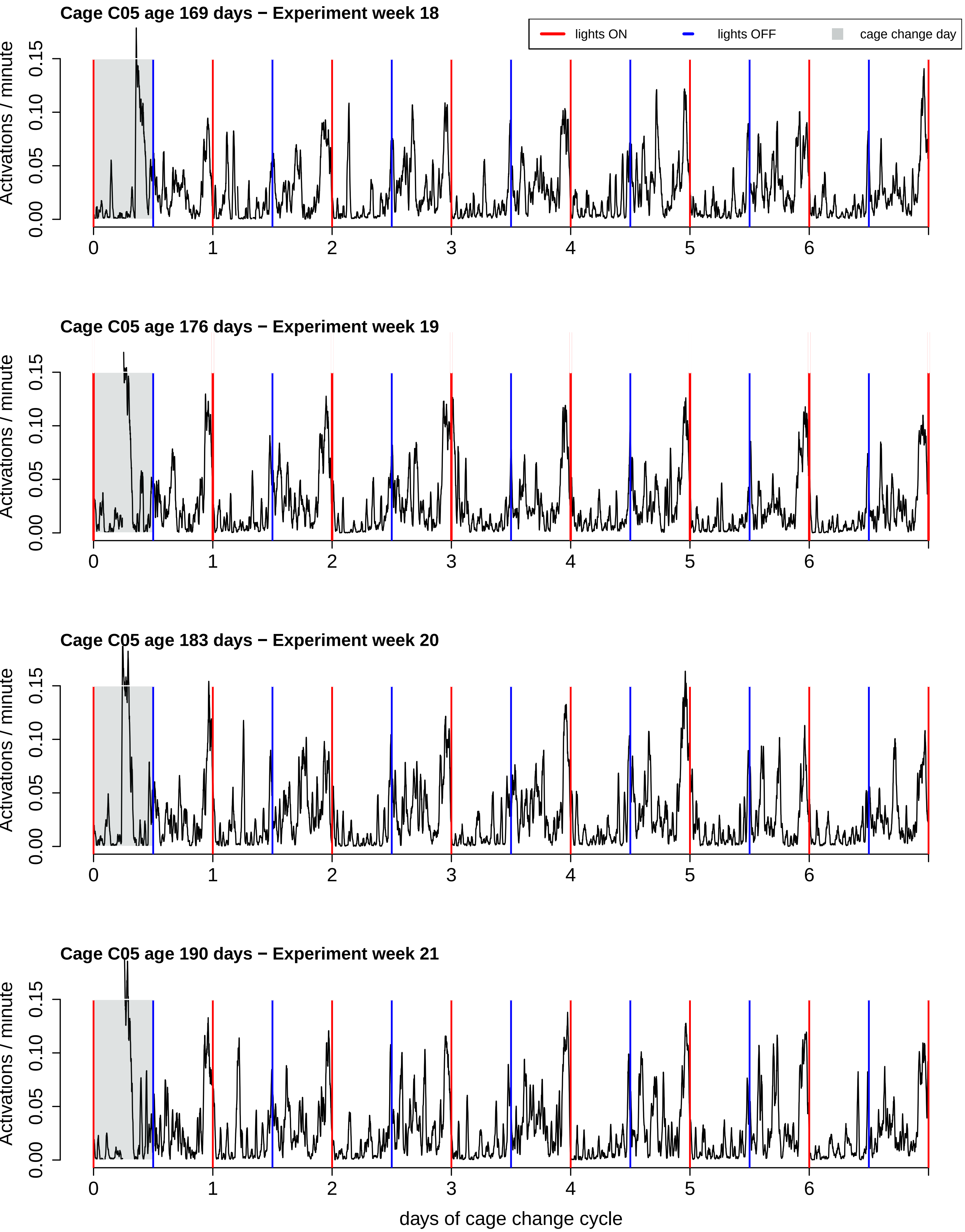

Cage C05 age 169 days − Experiment week 18
lights ON
lights OFF
cage change day
Activations / minute
0.00
0.05
0.10
0.15
0
1
2
3
4
5
6
Cage C05 age 176 days − Experiment week 19
Cage C05 age 183 days − Experiment week 20
Cage C05 age 190 days − Experiment week 21
days of cage change cycle

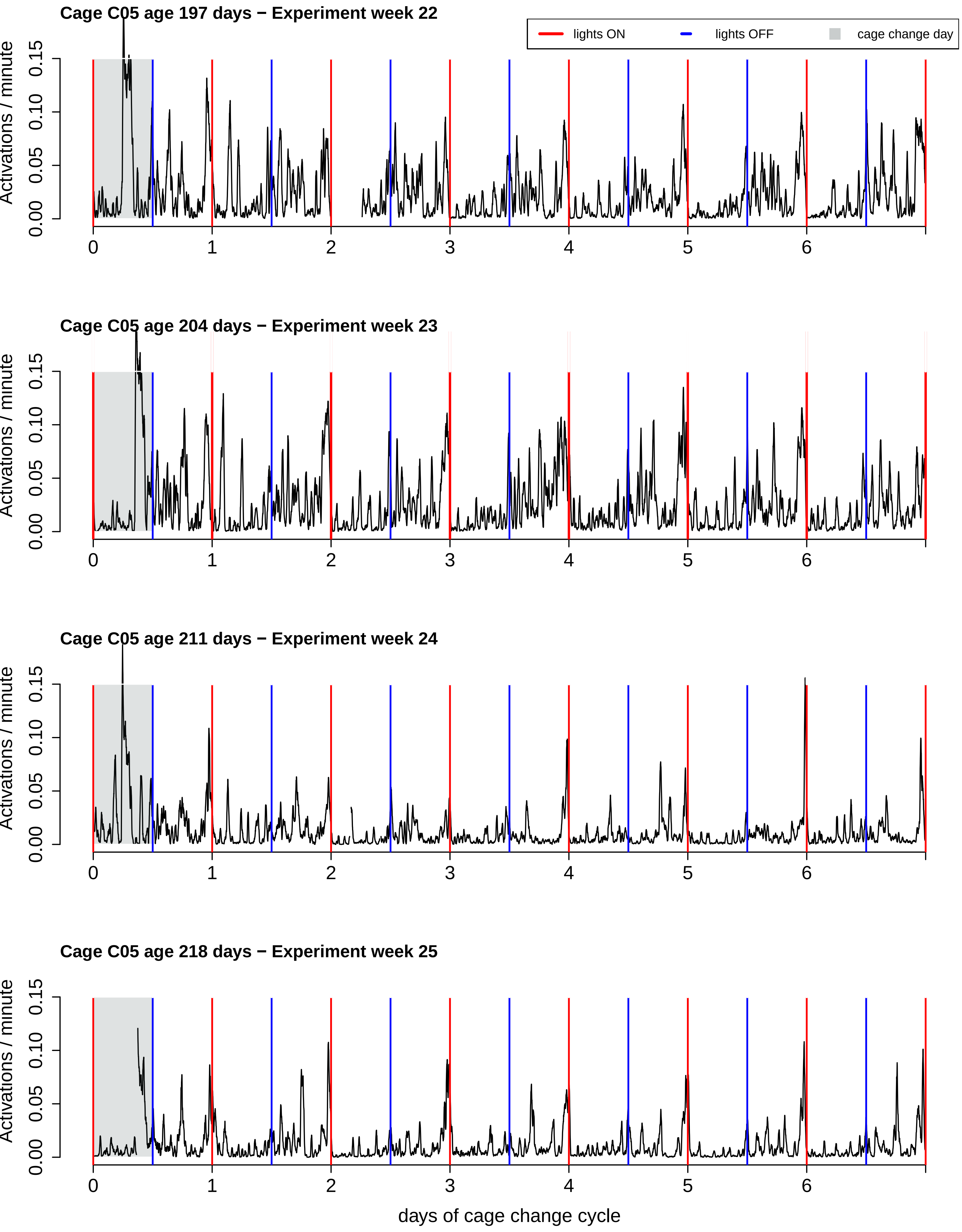

Cage C05 age 197 days − Experiment week 22
lights ON
lights OFF
cage change day
Activations / minute
0.00
0.05
0.10
0.15
0
1
2
3
4
5
6
Cage C05 age 204 days − Experiment week 23
Cage C05 age 211 days − Experiment week 24
Cage C05 age 218 days − Experiment week 25
days of cage change cycle

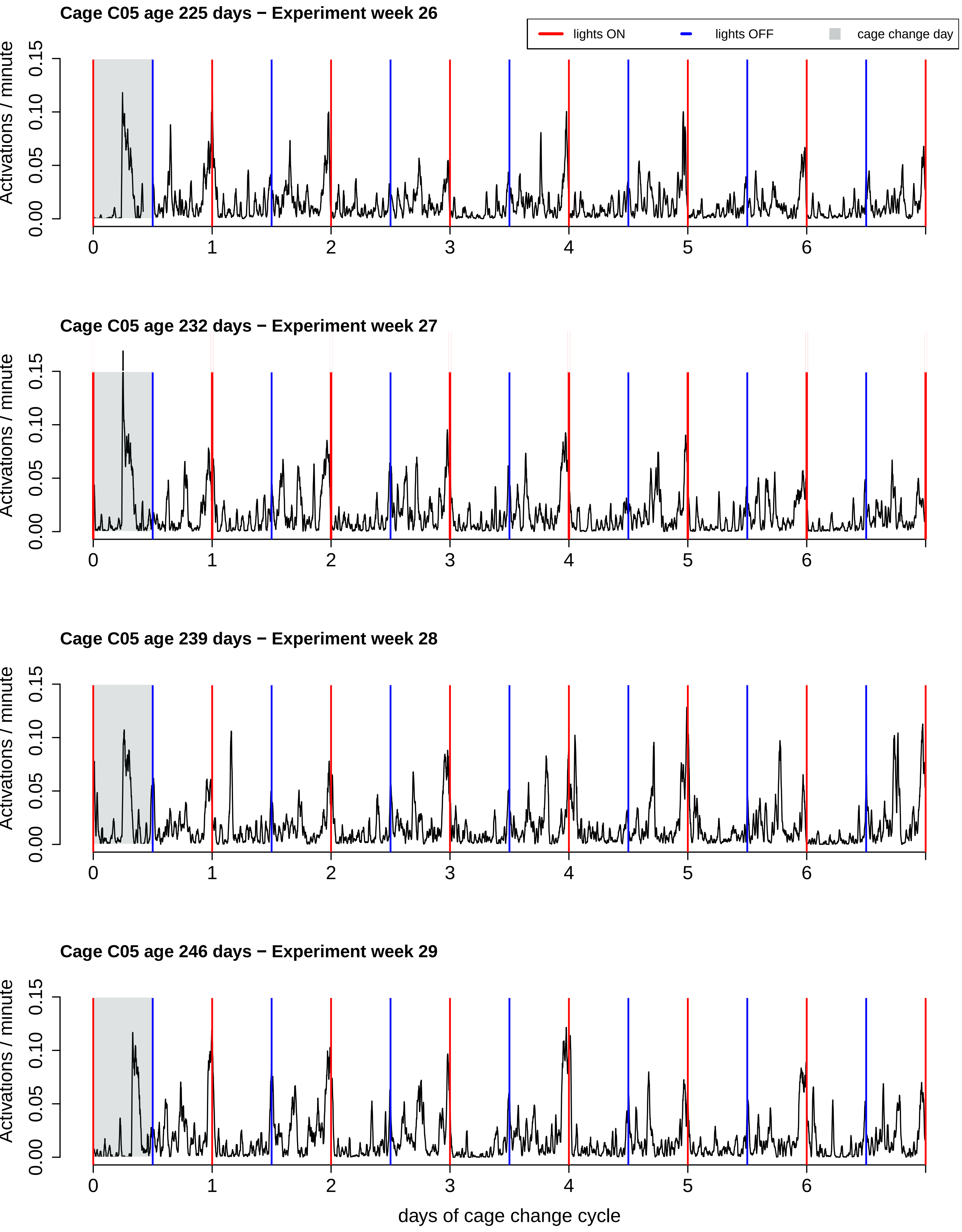
Cage C05 age 225 days − Experiment week 26
lights ON
lights OFF
cage change day
Activations / minute
0.00
0.05
0.10
0.15
0
1
2
3
4
5
6
Cage C05 age 232 days − Experiment week 27
Cage C05 age 239 days − Experiment week 28
Cage C05 age 246 days − Experiment week 29
days of cage change cycle

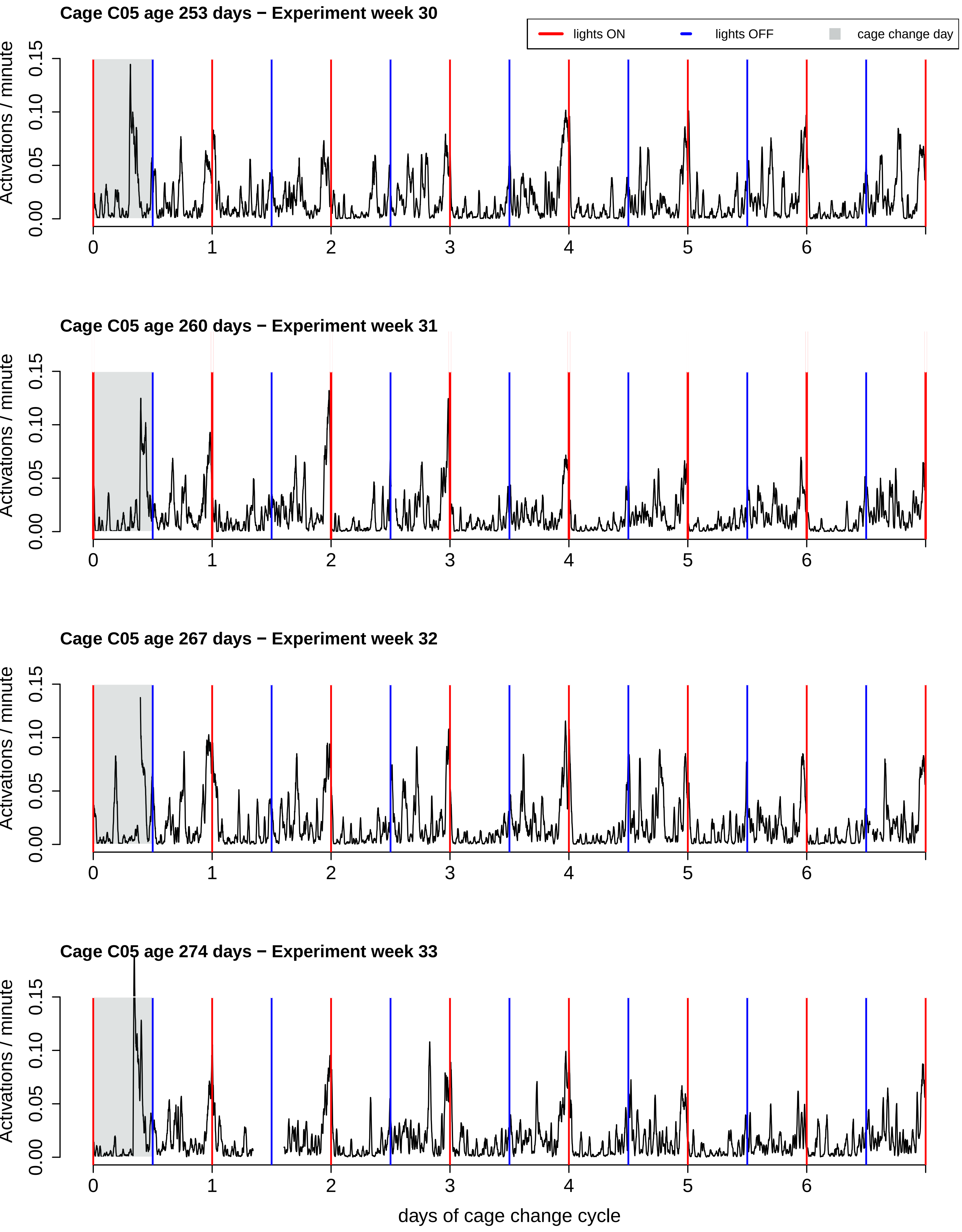

Cage C05 age 253 days − Experiment week 30
lights ON
lights OFF
cage change day
Activations / minute
0.00
0.05
0.10
0.15
0
1
2
3
4
5
6
Cage C05 age 260 days − Experiment week 31
Cage C05 age 267 days − Experiment week 32
Cage C05 age 274 days − Experiment week 33
days of cage change cycle

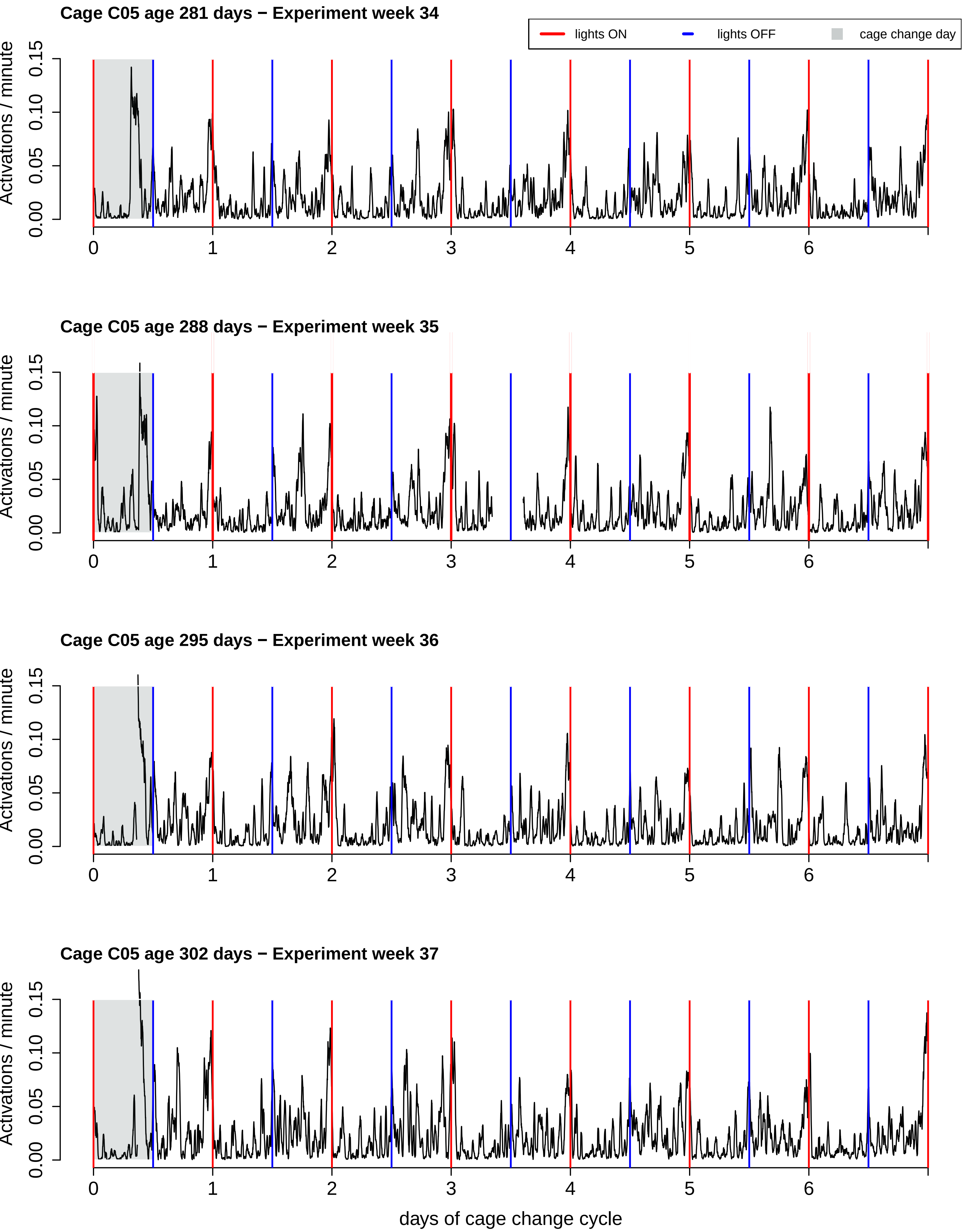
Cage C05 age 281 days − Experiment week 34
lights ON
lights OFF
cage change day
Activations / minute
0.00
0.05
0.10
0.15
0
1
2
3
4
5
6
Cage C05 age 288 days − Experiment week 35
Cage C05 age 295 days − Experiment week 36
Cage C05 age 302 days − Experiment week 37
days of cage change cycle

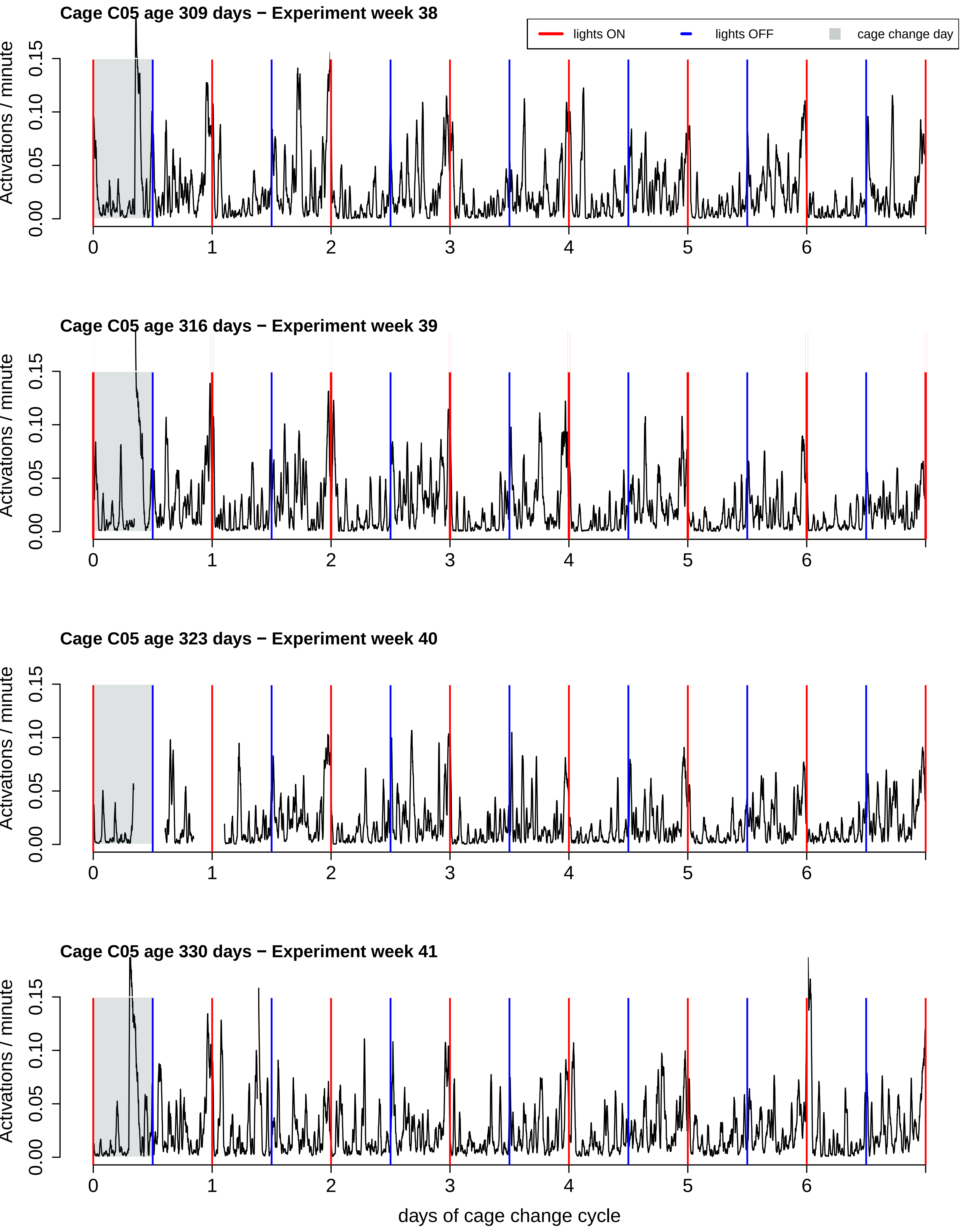

Cage C05 age 309 days − Experiment week 38
lights ON
lights OFF
cage change day
Activations / minute
0.00
0.05
0.10
0.15
0
1
2
3
4
5
6
Cage C05 age 316 days − Experiment week 39
Cage C05 age 323 days − Experiment week 40
Cage C05 age 330 days − Experiment week 41
days of cage change cycle

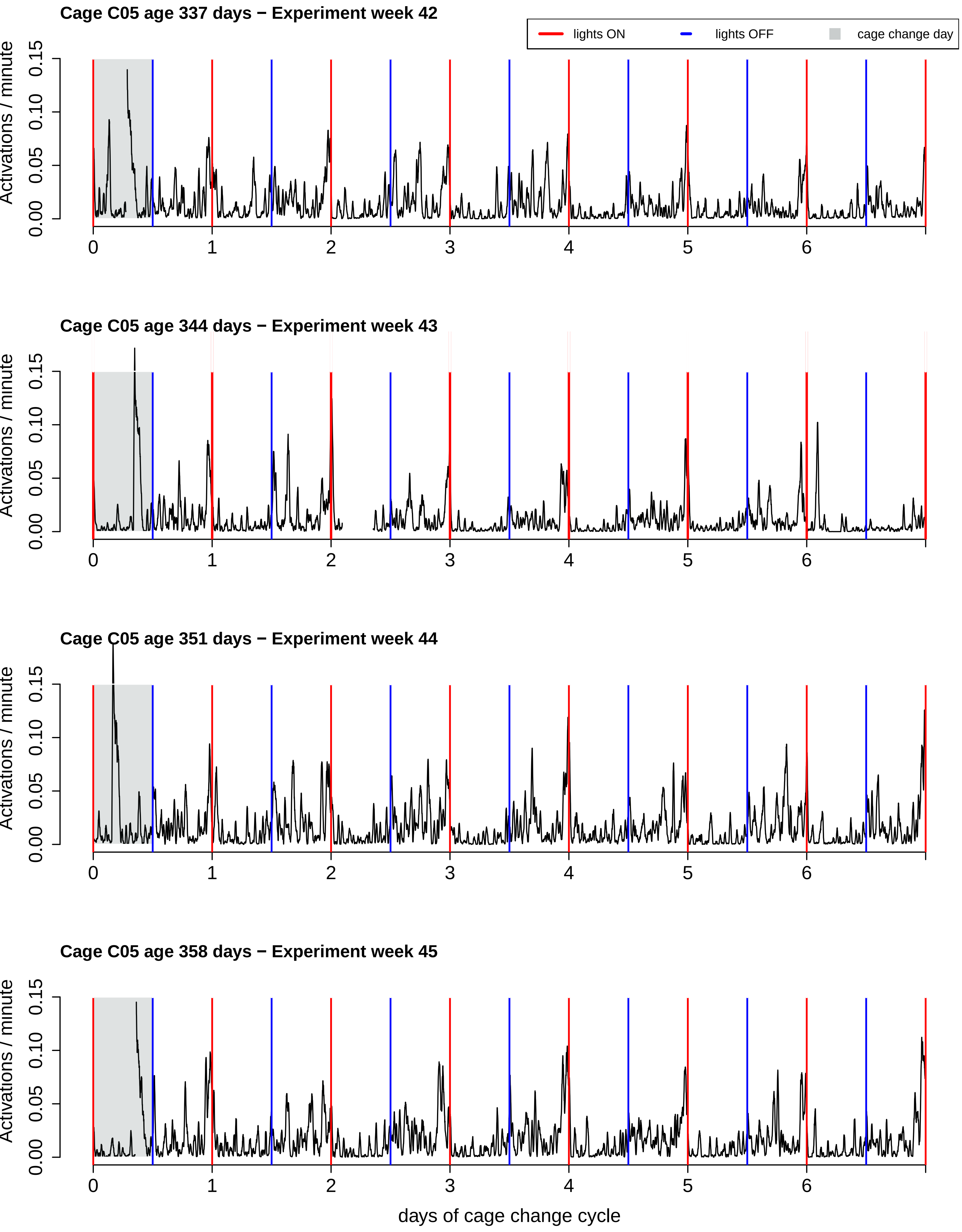
Cage C05 age 337 days − Experiment week 42
lights ON
lights OFF
cage change day
Activations / minute
0.00
0.05
0.10
0.15
0
1
2
3
4
5
6
Cage C05 age 344 days − Experiment week 43
Cage C05 age 351 days − Experiment week 44
Cage C05 age 358 days − Experiment week 45
days of cage change cycle

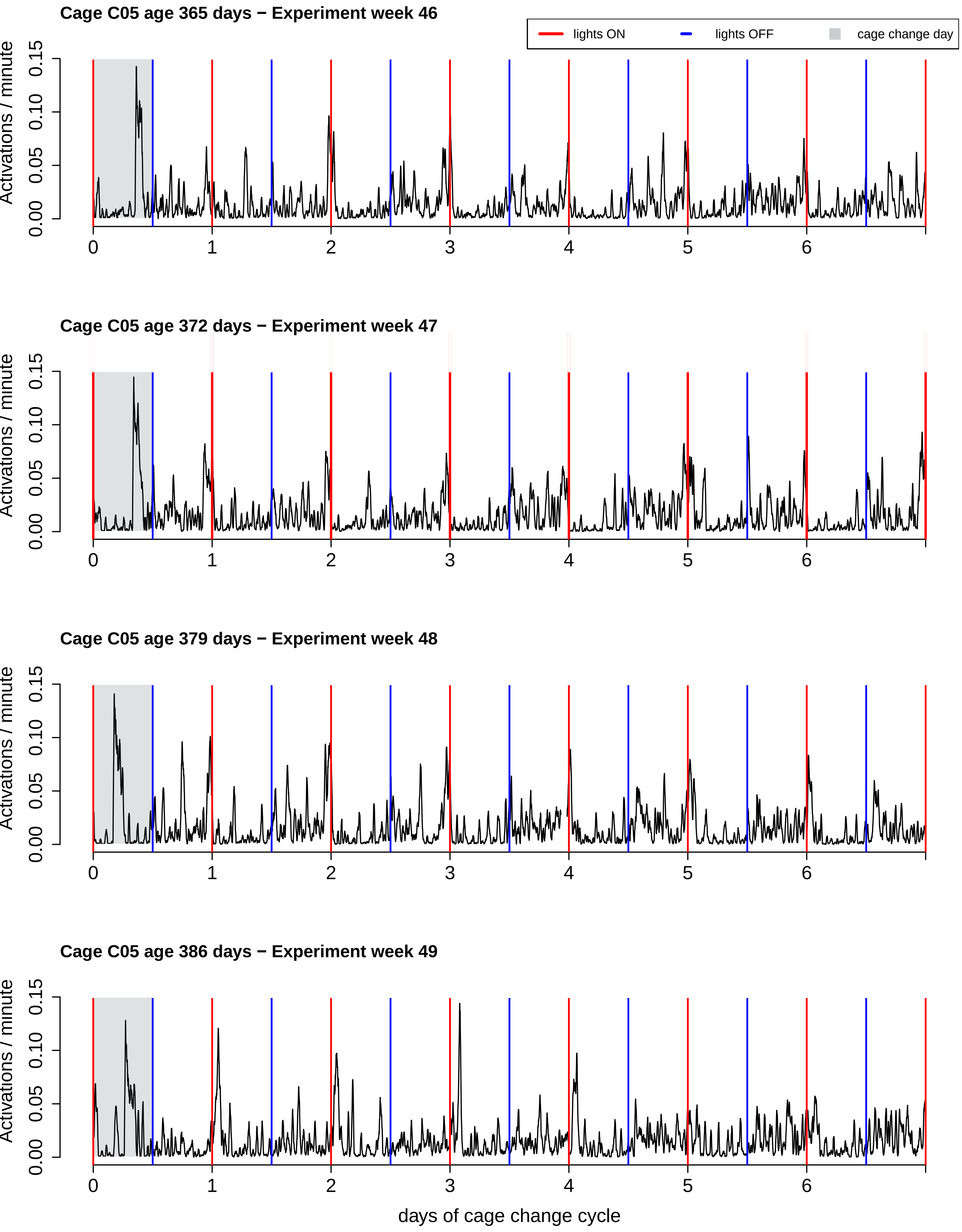
Cage C05 age 365 days − Experiment week 46
lights ON
lights OFF
cage change day
Activations / minute
0.00
0.05
0.10
0.15
0
1
2
3
4
5
6
Cage C05 age 372 days − Experiment week 47
Cage C05 age 379 days − Experiment week 48
Cage C05 age 386 days − Experiment week 49
days of cage change cycle

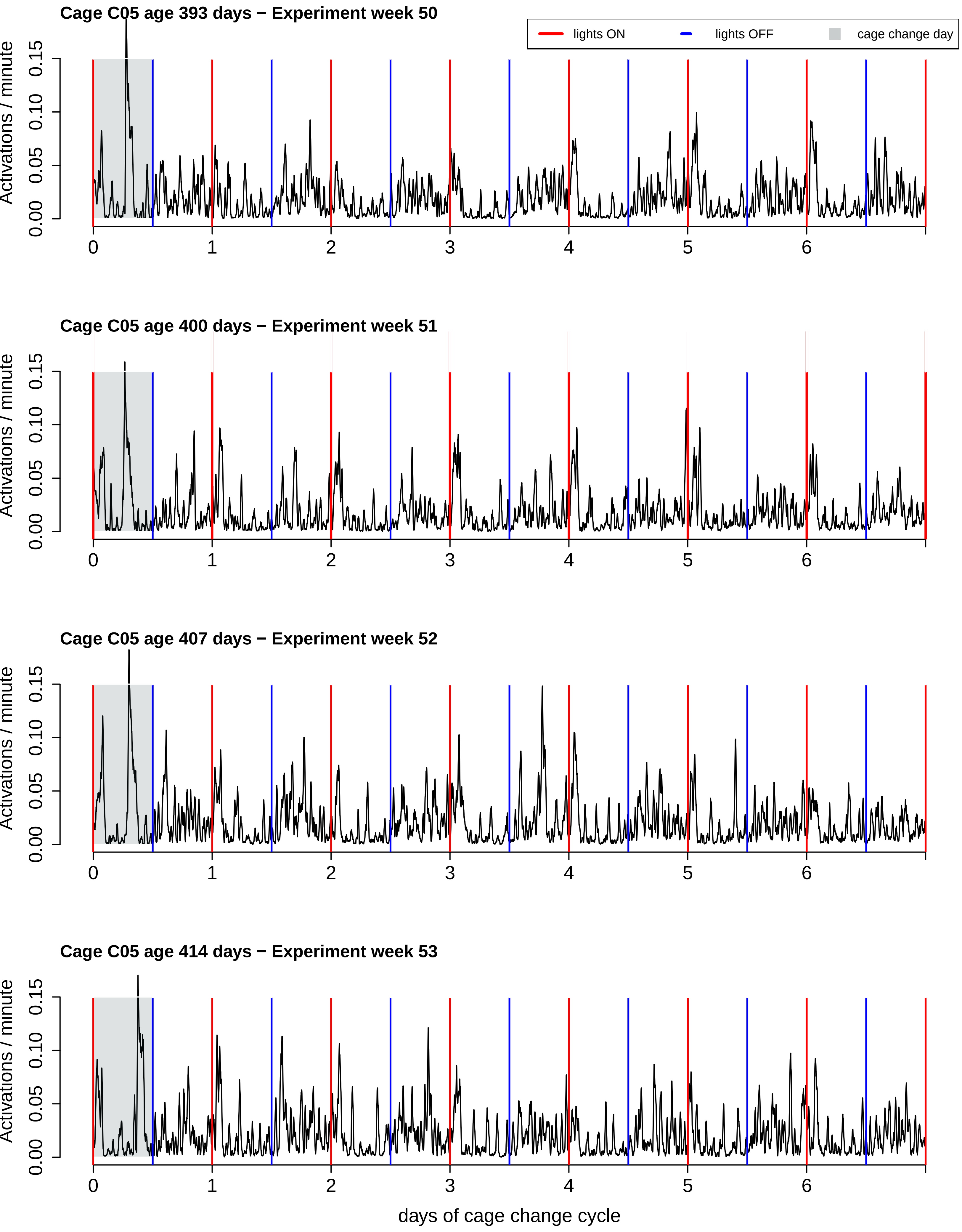
Cage C05 age 393 days − Experiment week 50
lights ON
lights OFF
cage change day
Activations / minute
0.00
0.05
0.10
0.15
0
1
2
3
4
5
6
Cage C05 age 400 days − Experiment week 51
Activations / minute
0.00
0.05
0.10
0.15
0
1
2
3
4
5
6
Cage C05 age 407 days − Experiment week 52
Activations / minute
0.00
0.05
0.10
0.15
0
1
2
3
4
5
6
Cage C05 age 414 days − Experiment week 53
Activations / minute
0.00
0.05
0.10
0.15
0
1
2
3
4
5
6
days of cage change cycle

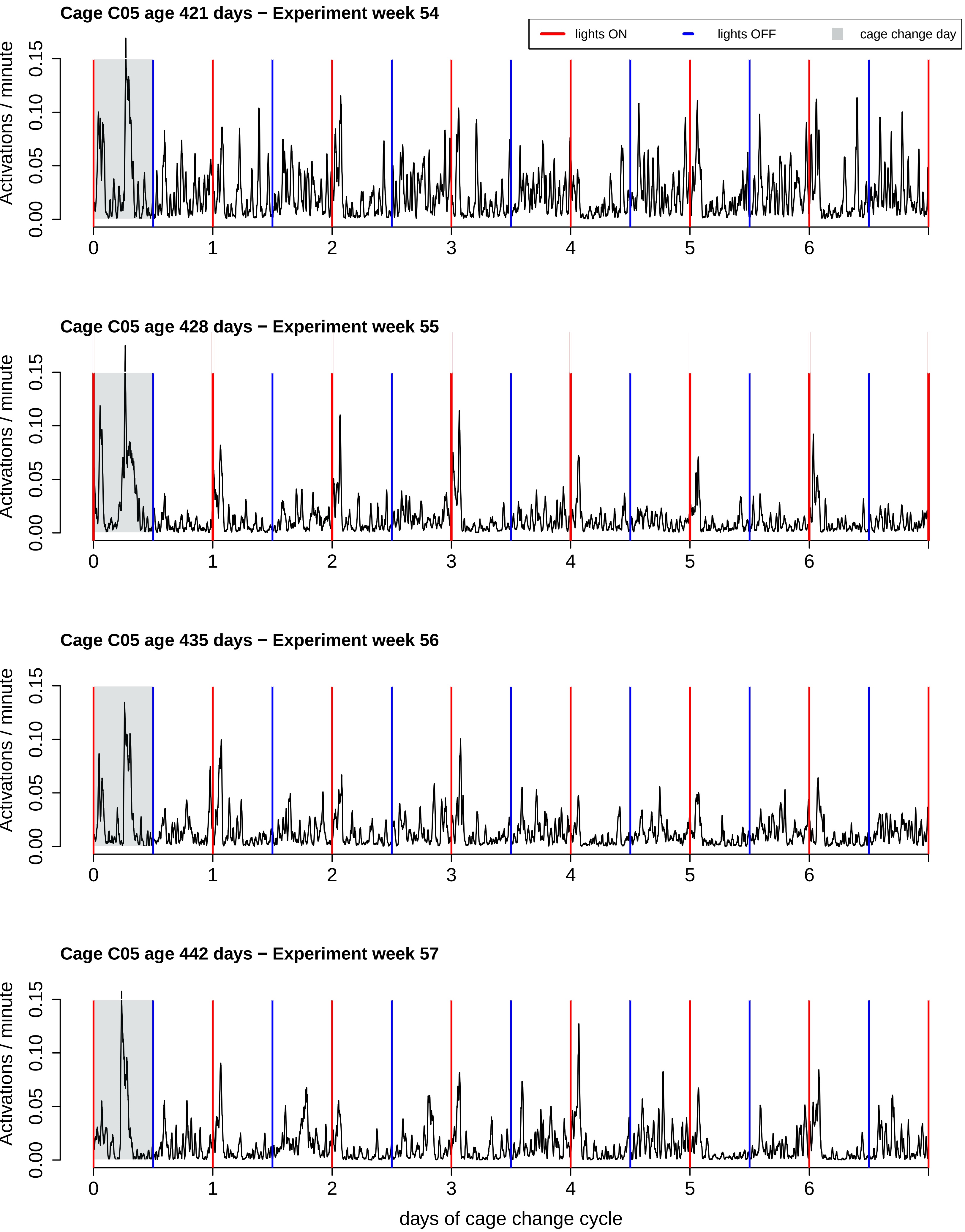

Cage C05 age 421 days − Experiment week 54
lights ON
lights OFF
cage change day
Cage C05 age 428 days − Experiment week 55
Cage C05 age 435 days − Experiment week 56
Cage C05 age 442 days − Experiment week 57
Activations / minute
0.00
0.05
0.10
0.15
0
1
2
3
4
5
6
days of cage change cycle

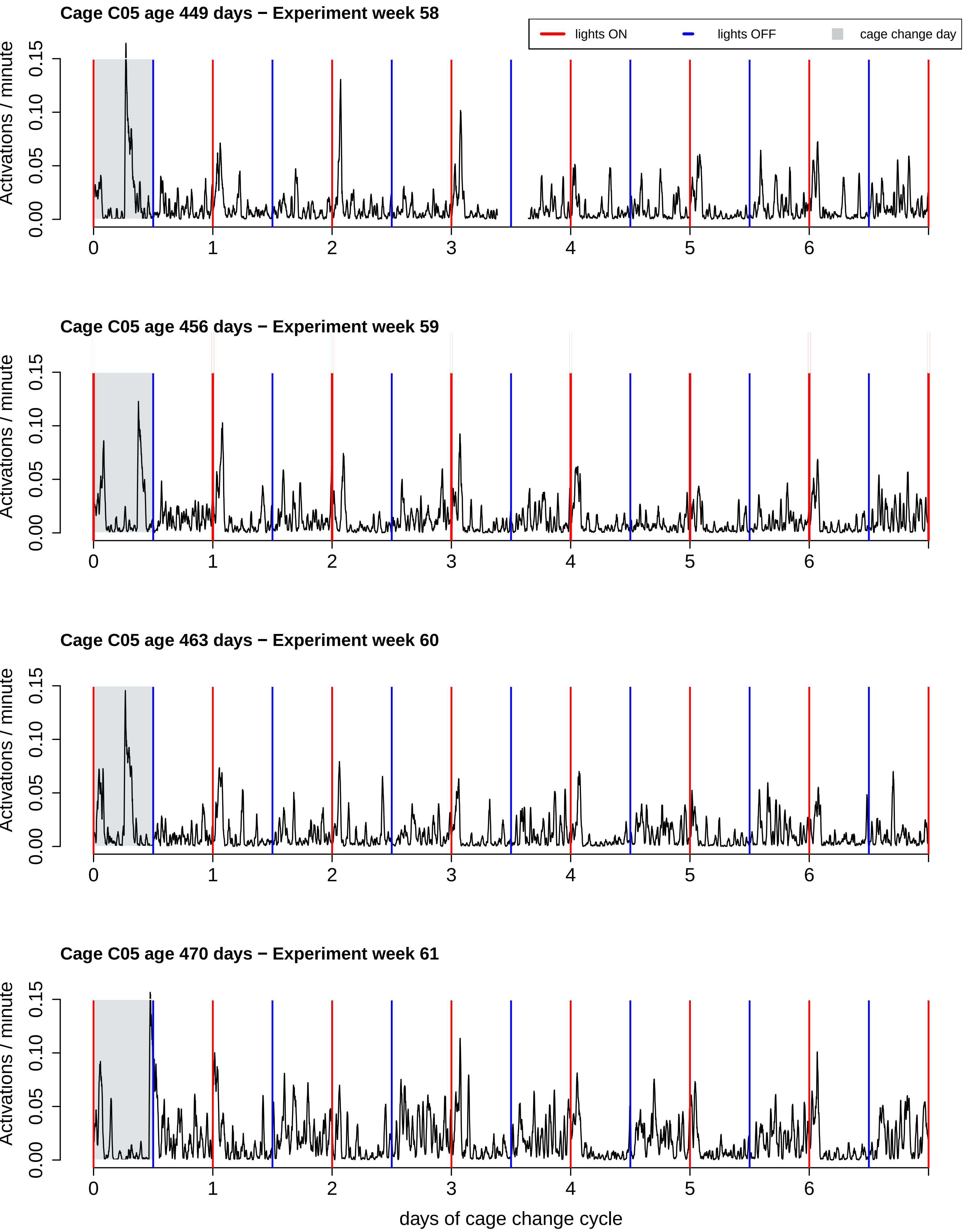

Cage C05 age 449 days − Experiment week 58
lights ON
lights OFF
cage change day
Activations / minute
0.00
0.05
0.10
0.15
0
1
2
3
4
5
6
Cage C05 age 456 days − Experiment week 59
Cage C05 age 463 days − Experiment week 60
Cage C05 age 470 days − Experiment week 61
days of cage change cycle

Cage C05 age 477 days − Experiment week 62

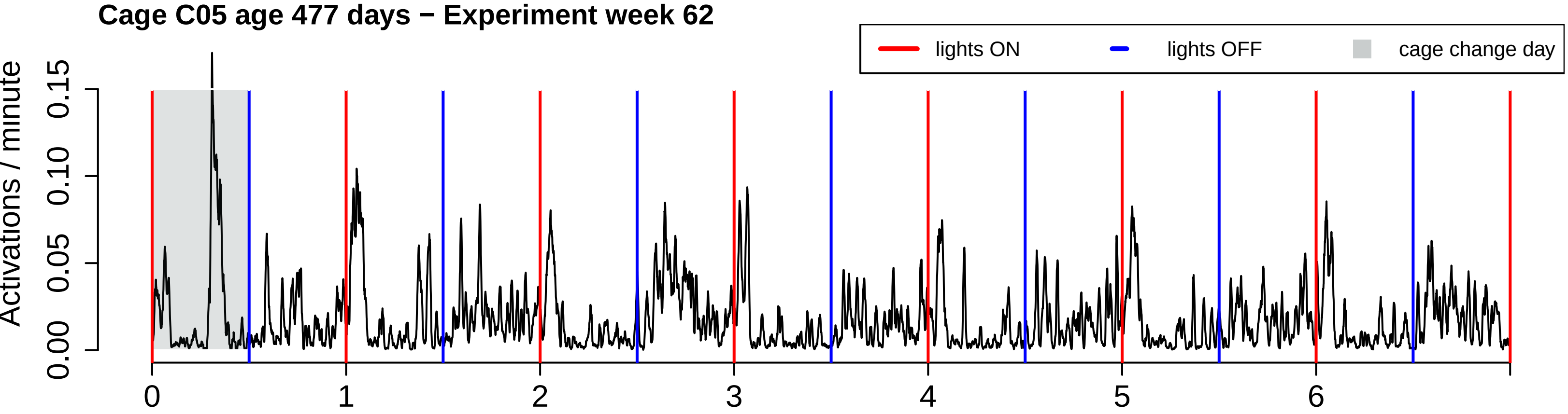

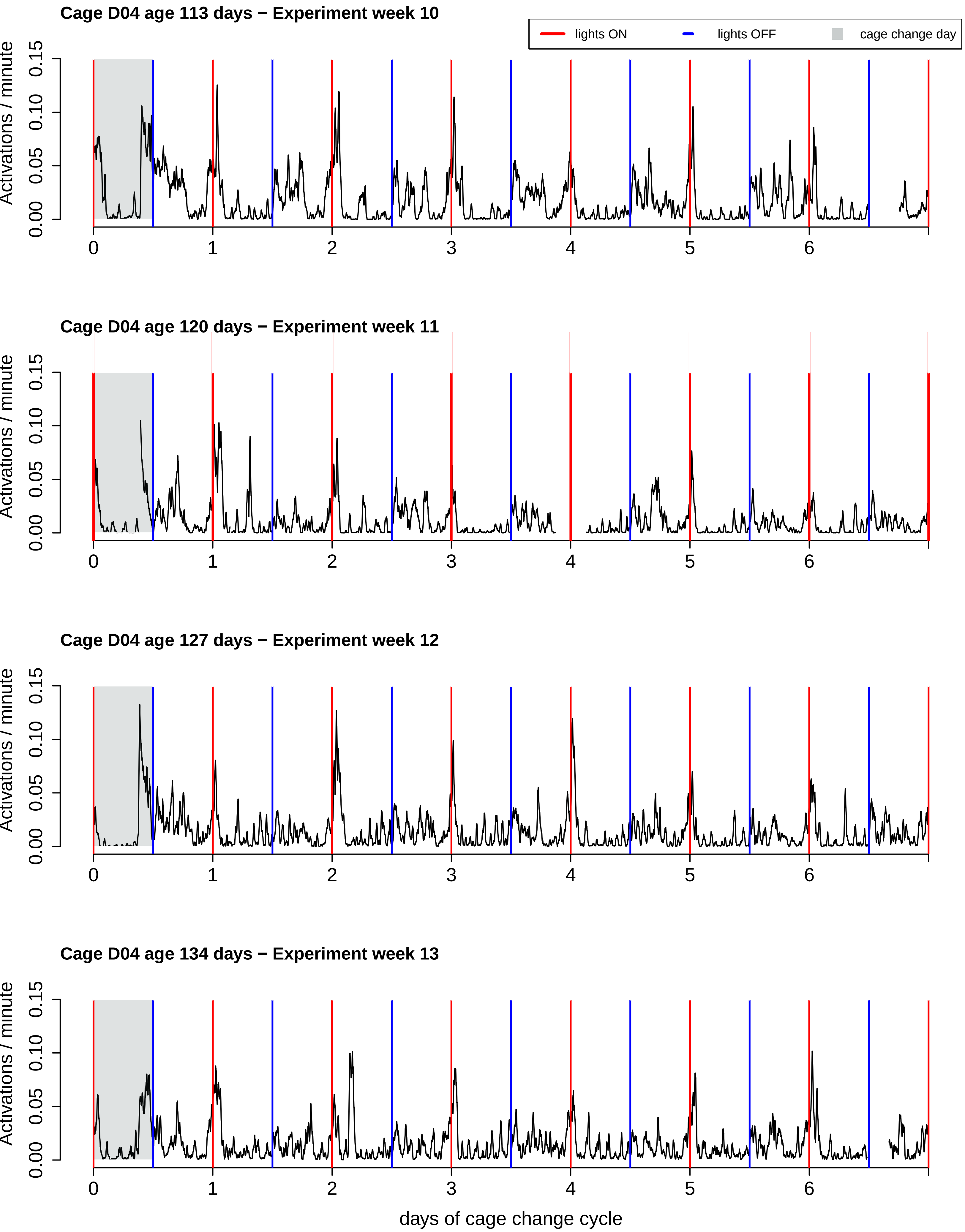

Cage D04 age 113 days − Experiment week 10
lights ON
lights OFF
cage change day
Activations / minute
0.00
0.05
0.10
0.15
0
1
2
3
4
5
6
Cage D04 age 120 days − Experiment week 11
Cage D04 age 127 days − Experiment week 12
Cage D04 age 134 days − Experiment week 13
days of cage change cycle

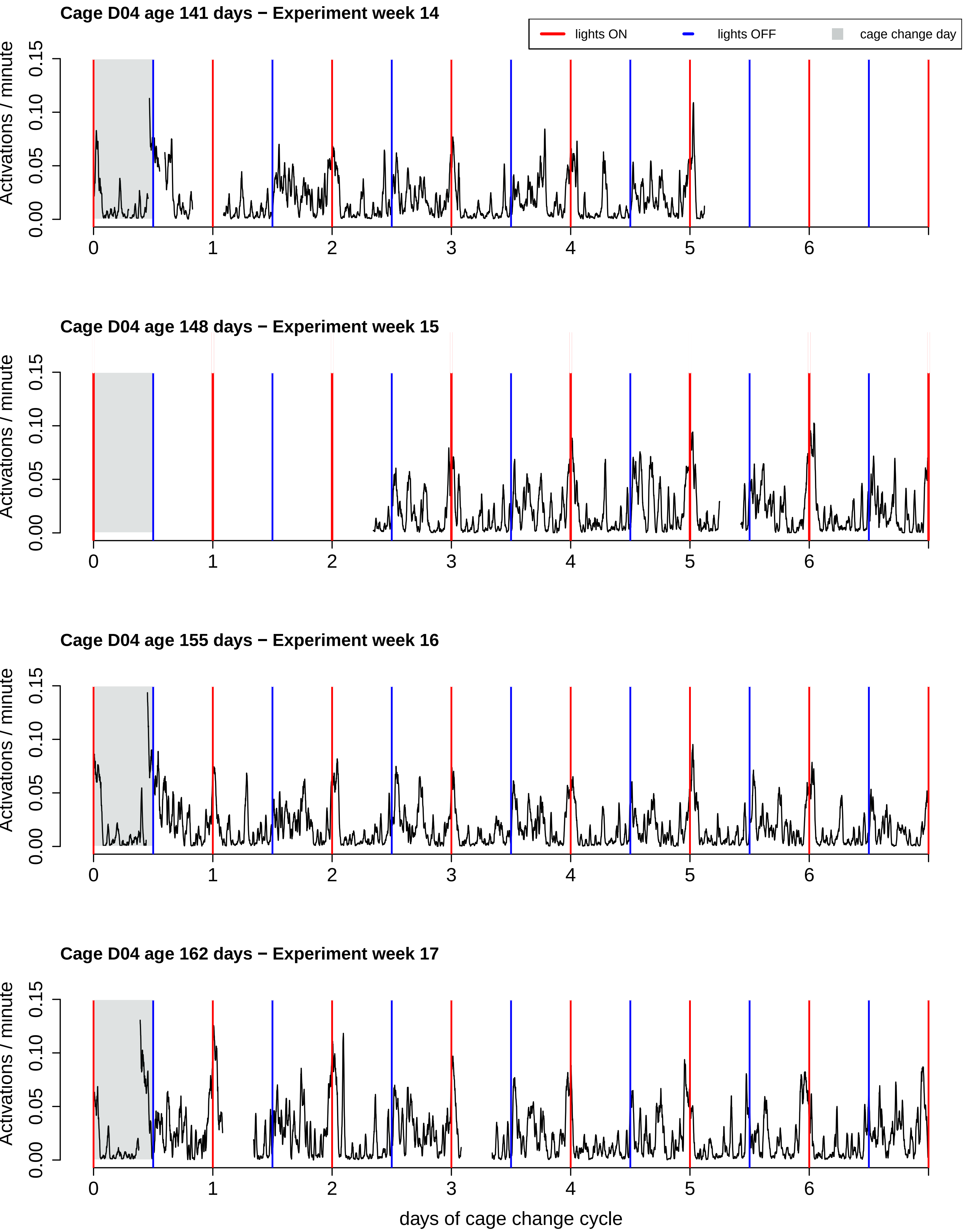

Cage D04 age 141 days − Experiment week 14
lights ON
lights OFF
cage change day
Activations / minute
0.00
0.05
0.10
0.15
0
1
2
3
4
5
6
Cage D04 age 148 days − Experiment week 15
Cage D04 age 155 days − Experiment week 16
Cage D04 age 162 days − Experiment week 17
days of cage change cycle

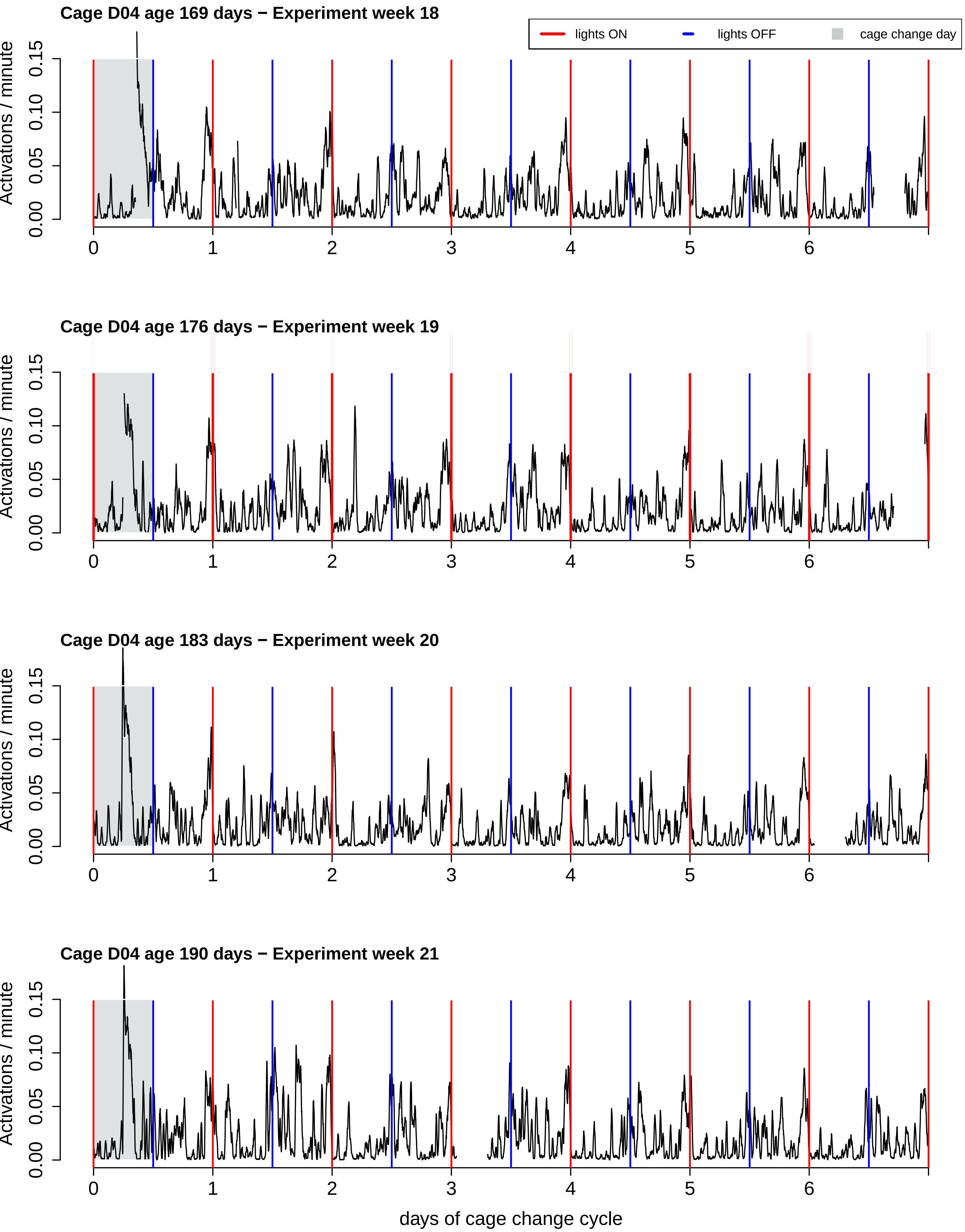

Cage D04 age 169 days − Experiment week 18
lights ON
lights OFF
cage change day
Activations / minute
0.00
0.05
0.10
0.15
0
1
2
3
4
5
6
Cage D04 age 176 days − Experiment week 19
Cage D04 age 183 days − Experiment week 20
Cage D04 age 190 days − Experiment week 21
days of cage change cycle

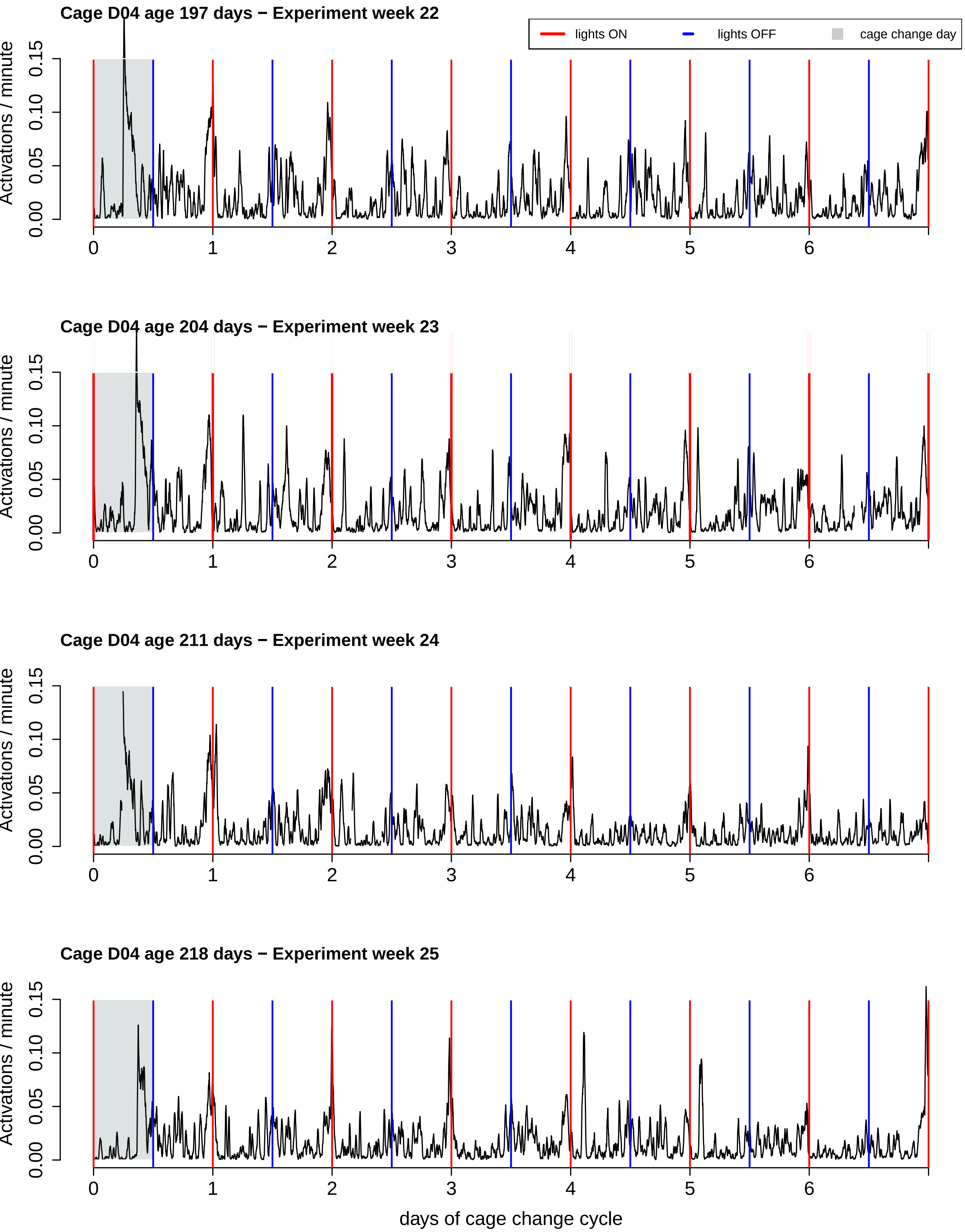
Cage D04 age 197 days − Experiment week 22
lights ON
lights OFF
cage change day
Activations / minute
0.00
0.05
0.10
0.15
0
1
2
3
4
5
6
Cage D04 age 204 days − Experiment week 23
Cage D04 age 211 days − Experiment week 24
Cage D04 age 218 days − Experiment week 25
days of cage change cycle

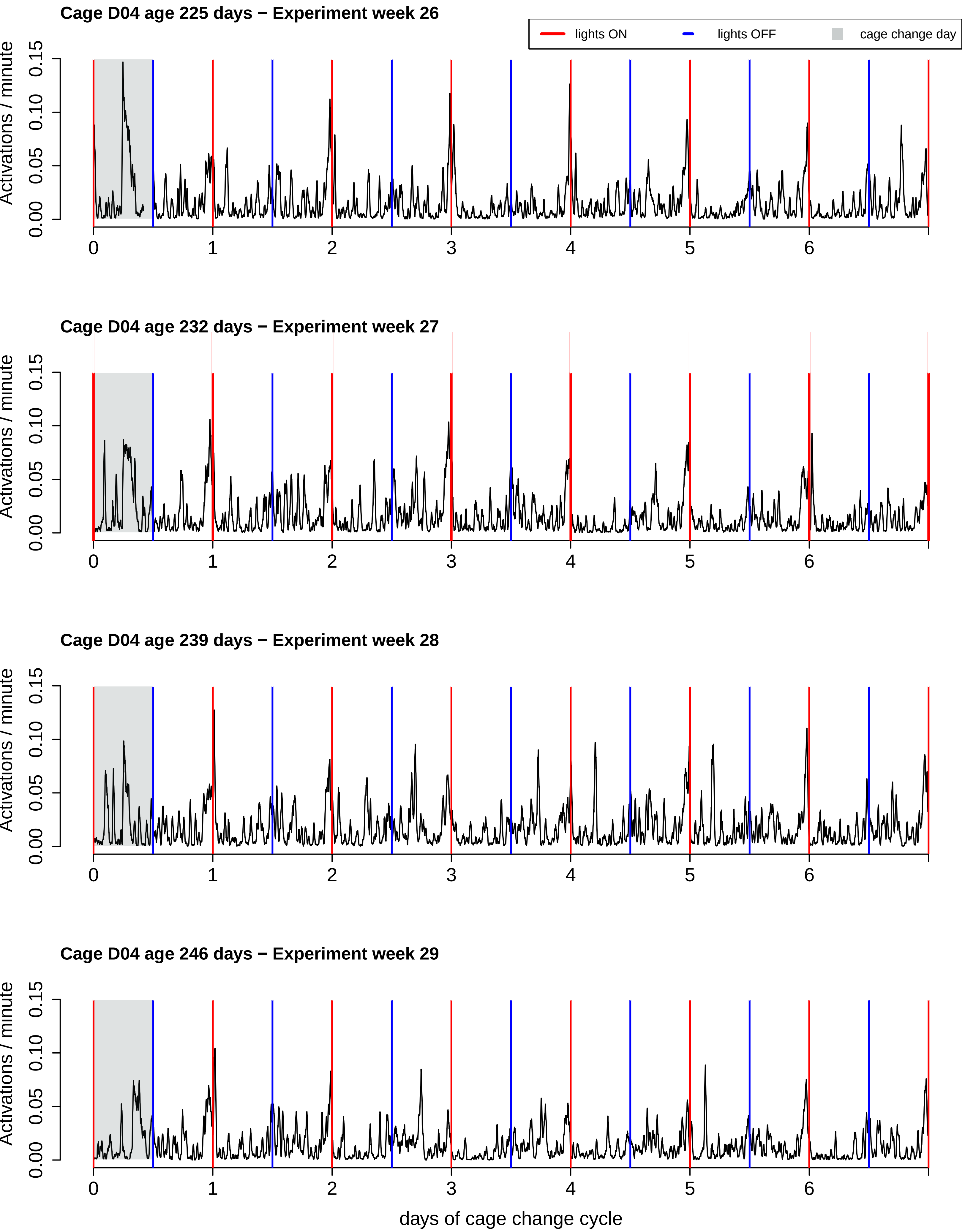

Cage D04 age 225 days − Experiment week 26
lights ON
lights OFF
cage change day
Cage D04 age 232 days − Experiment week 27
Cage D04 age 239 days − Experiment week 28
Cage D04 age 246 days − Experiment week 29
Activations / minute
0.00
0.05
0.10
0.15
0
1
2
3
4
5
6
days of cage change cycle

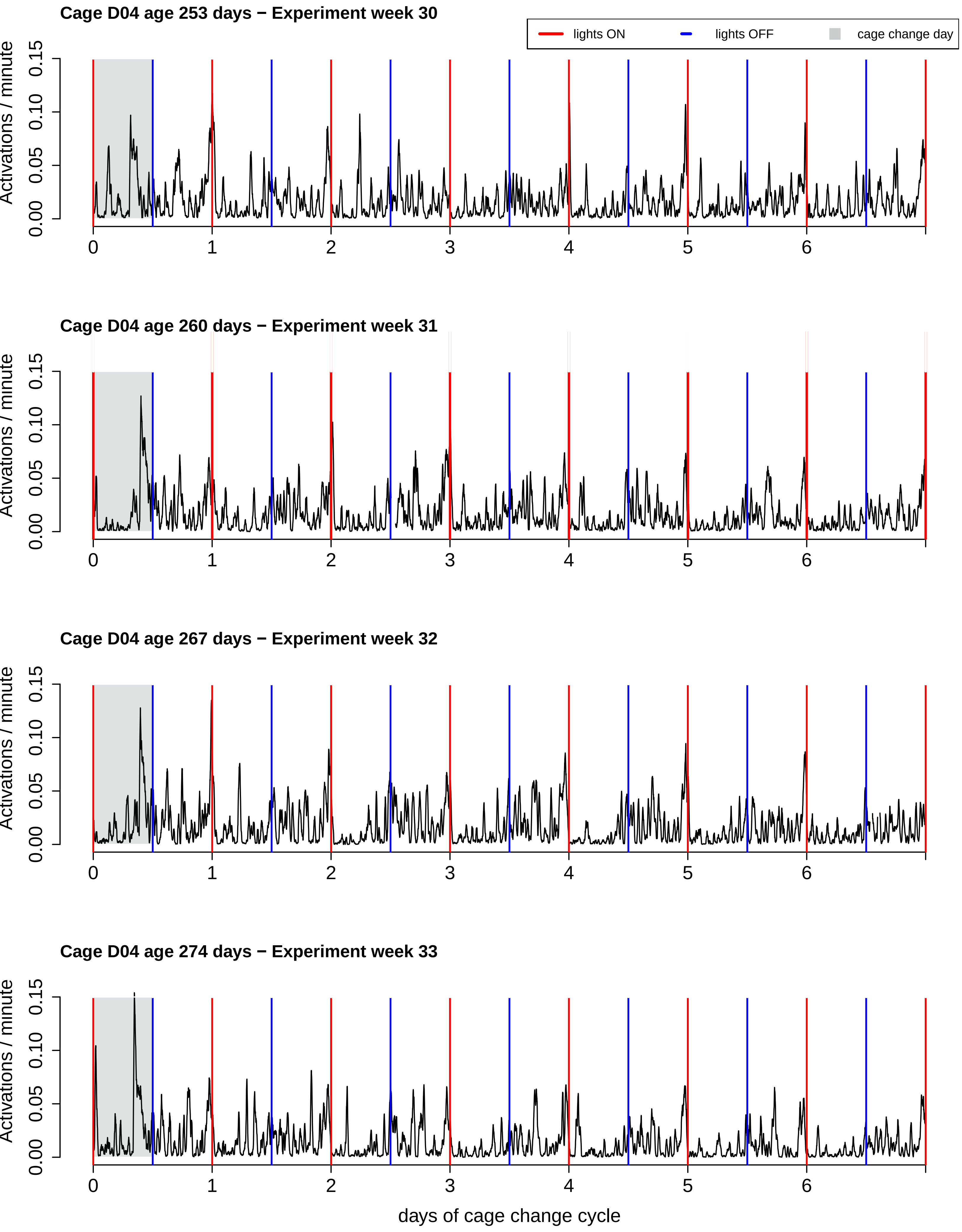
Cage D04 age 253 days − Experiment week 30
lights ON
lights OFF
cage change day
Cage D04 age 260 days − Experiment week 31
Cage D04 age 267 days − Experiment week 32
Cage D04 age 274 days − Experiment week 33
Activations / minute
0.00
0.05
0.10
0.15
0
1
2
3
4
5
6
days of cage change cycle

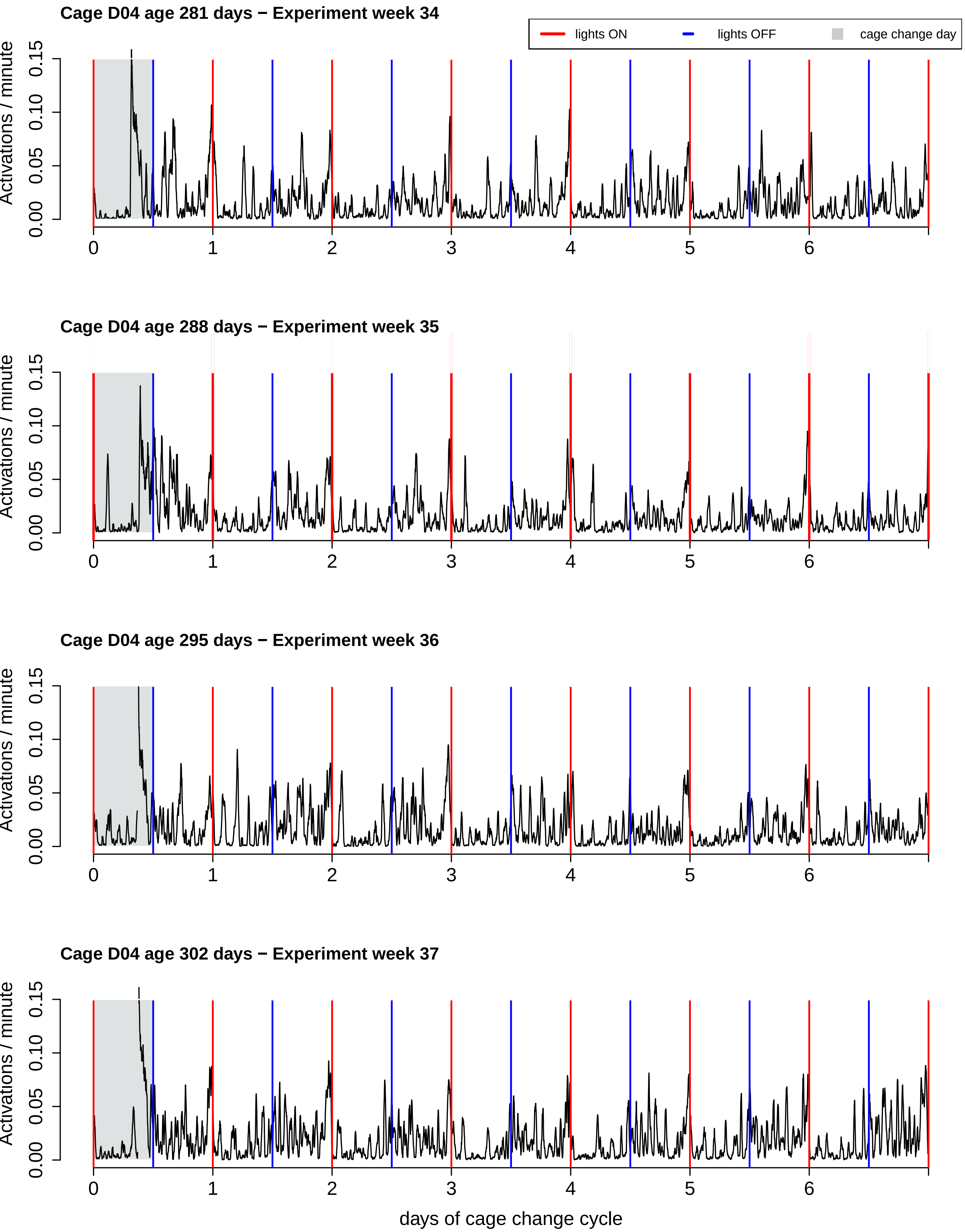

Cage D04 age 281 days − Experiment week 34
lights ON
lights OFF
cage change day
Activations / minute
0.00
0.05
0.10
0.15
0
1
2
3
4
5
6
Cage D04 age 288 days − Experiment week 35
Cage D04 age 295 days − Experiment week 36
Cage D04 age 302 days − Experiment week 37
days of cage change cycle

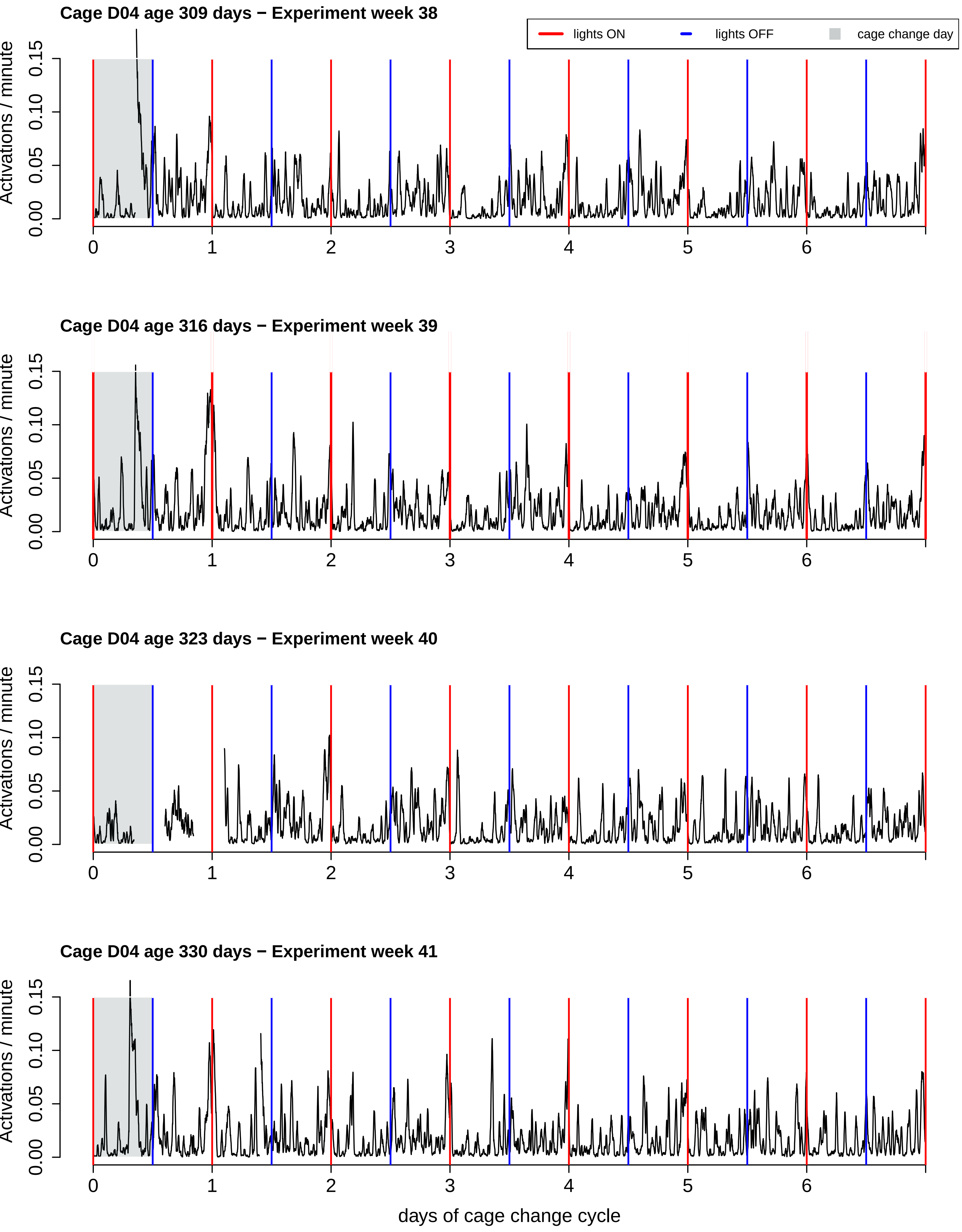
Cage D04 age 309 days − Experiment week 38
lights ON
lights OFF
cage change day
Activations / minute
0.00
0.05
0.10
0.15
0
1
2
3
4
5
6
Cage D04 age 316 days − Experiment week 39
Cage D04 age 323 days − Experiment week 40
Cage D04 age 330 days − Experiment week 41
days of cage change cycle

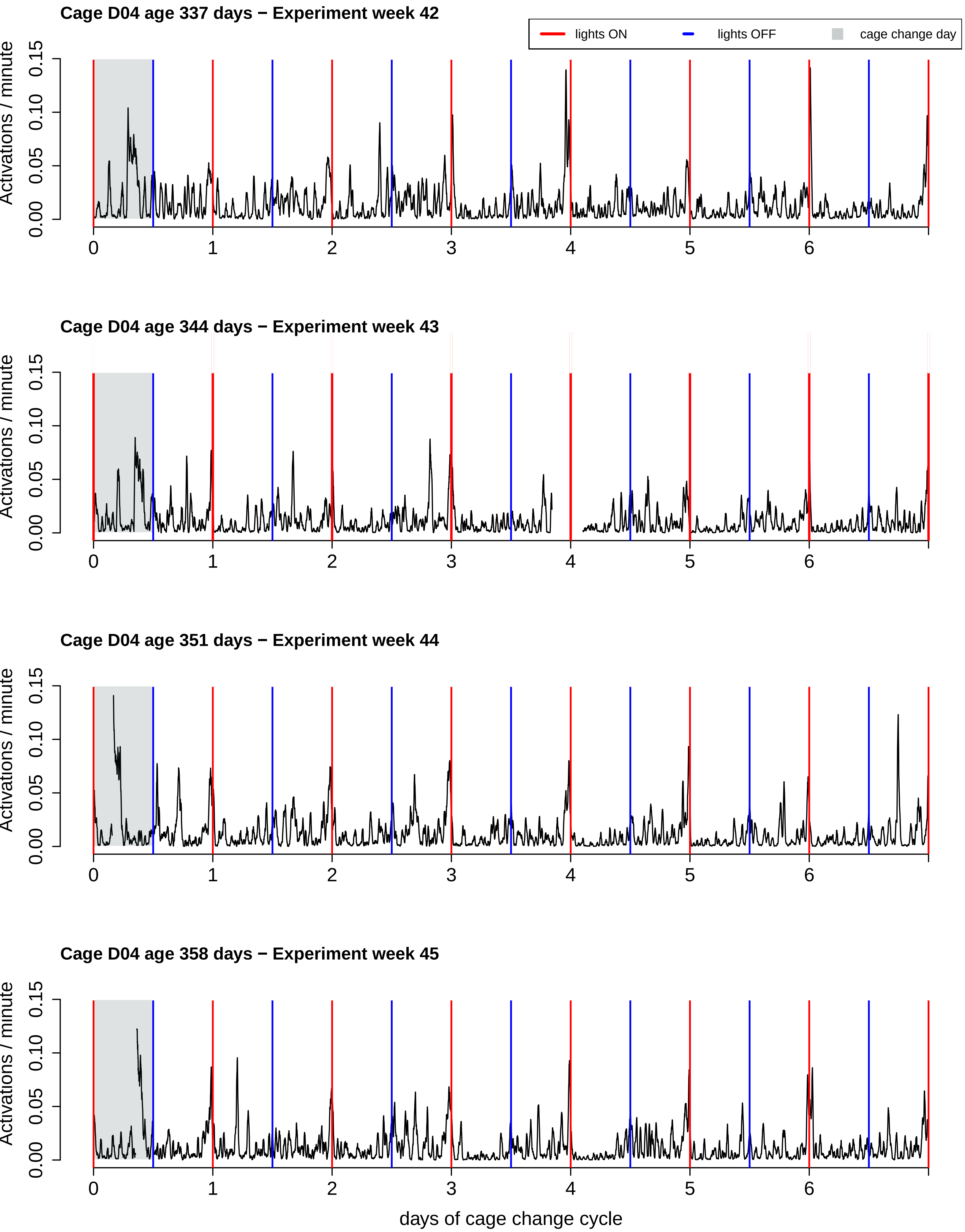

Cage D04 age 337 days − Experiment week 42
lights ON
lights OFF
cage change day
Activations / minute
0.00
0.05
0.10
0.15
0
1
2
3
4
5
6
Cage D04 age 344 days − Experiment week 43
Cage D04 age 351 days − Experiment week 44
Cage D04 age 358 days − Experiment week 45
days of cage change cycle

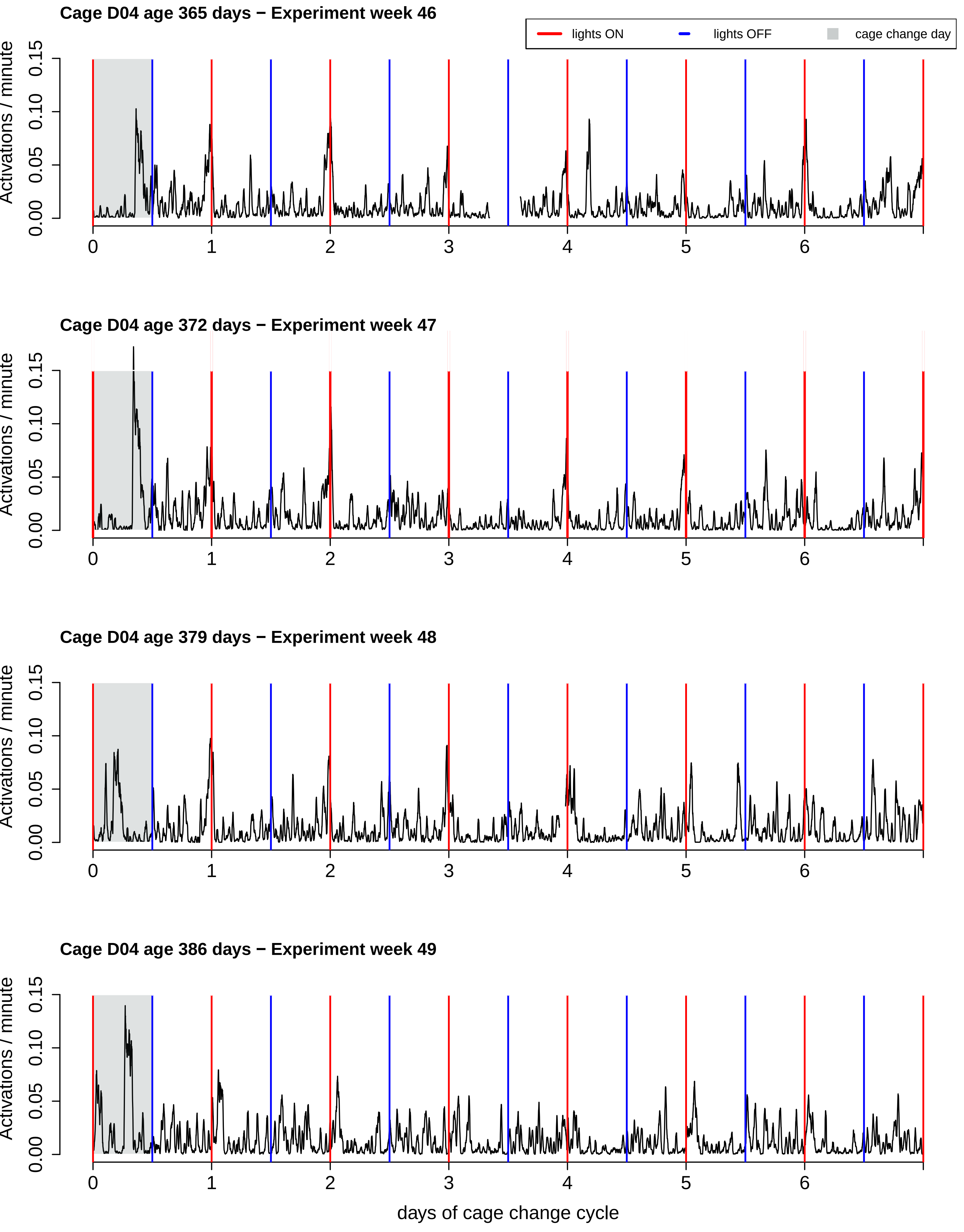
Cage D04 age 365 days − Experiment week 46
lights ON
lights OFF
cage change day
Activations / minute
0.00
0.05
0.10
0.15
0
1
2
3
4
5
6
Cage D04 age 372 days − Experiment week 47
Cage D04 age 379 days − Experiment week 48
Cage D04 age 386 days − Experiment week 49
days of cage change cycle

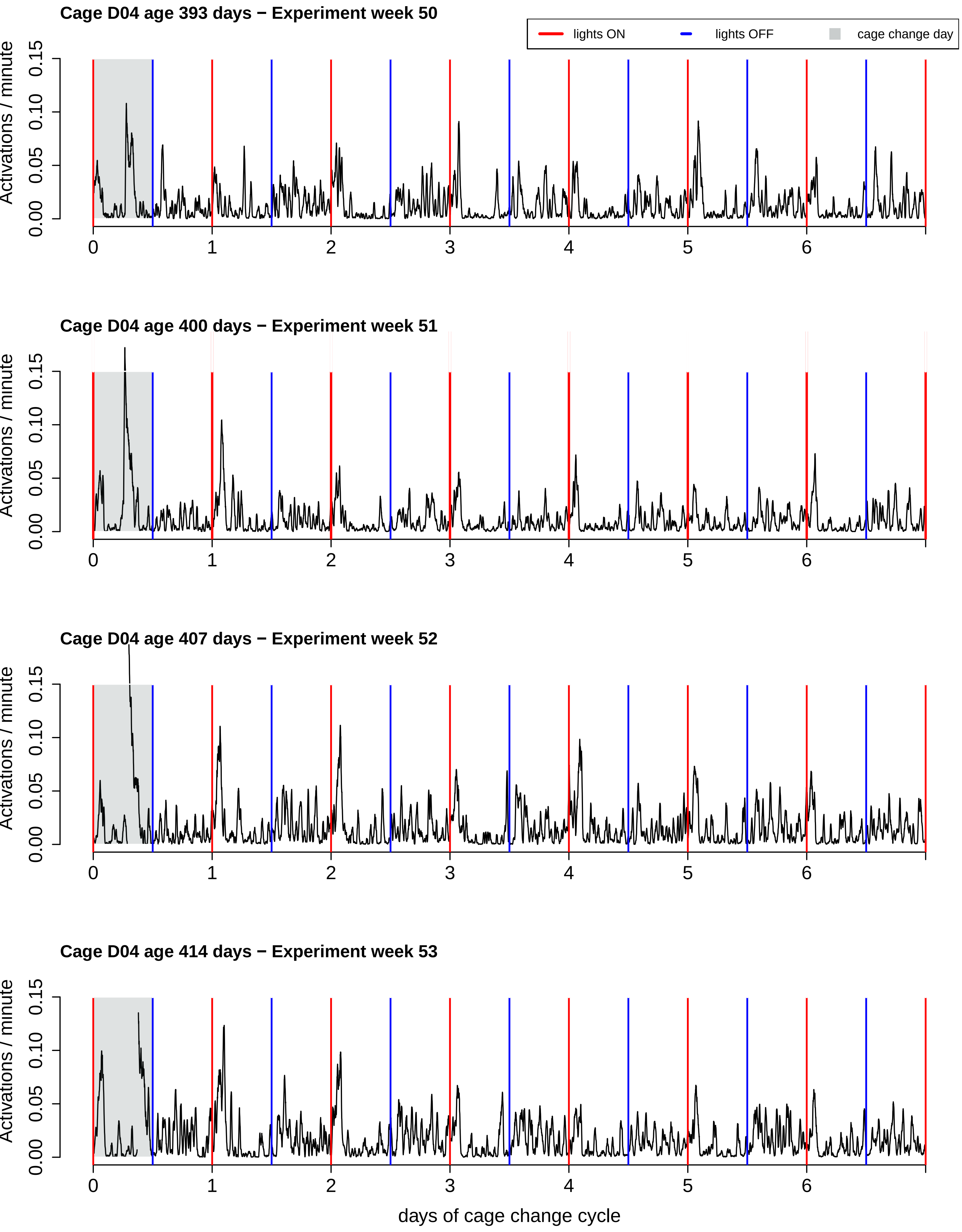
Cage D04 age 393 days − Experiment week 50
lights ON
lights OFF
cage change day
Activations / minute
0.00
0.05
0.10
0.15
0
1
2
3
4
5
6
Cage D04 age 400 days − Experiment week 51
Activations / minute
0.00
0.05
0.10
0.15
0
1
2
3
4
5
6
Cage D04 age 407 days − Experiment week 52
Activations / minute
0.00
0.05
0.10
0.15
0
1
2
3
4
5
6
Cage D04 age 414 days − Experiment week 53
Activations / minute
0.00
0.05
0.10
0.15
0
1
2
3
4
5
6
days of cage change cycle

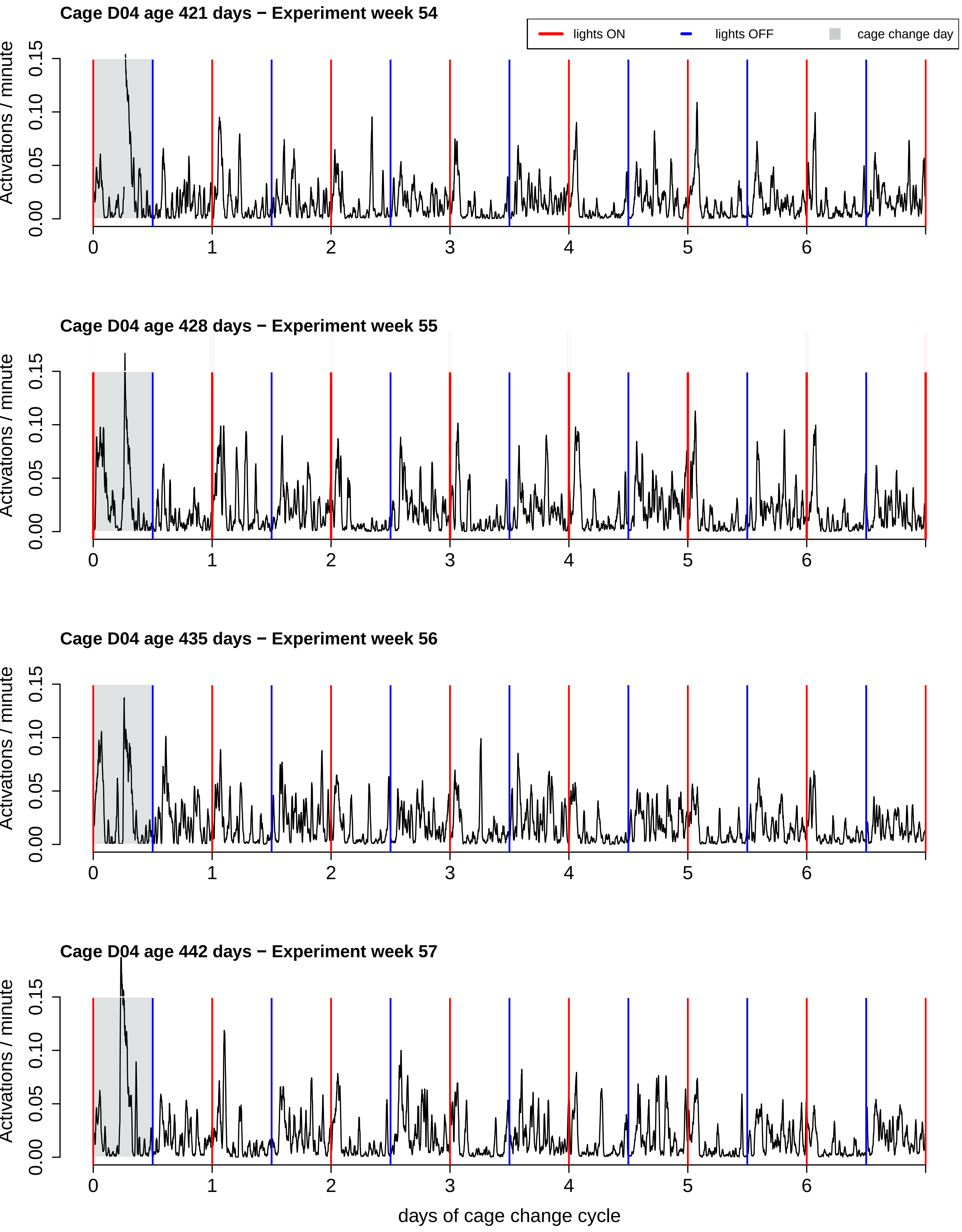
Cage D04 age 421 days − Experiment week 54
lights ON
lights OFF
cage change day
Activations / minute
0.00
0.05
0.10
0.15
0
1
2
3
4
5
6
Cage D04 age 428 days − Experiment week 55
Cage D04 age 435 days − Experiment week 56
Cage D04 age 442 days − Experiment week 57
days of cage change cycle

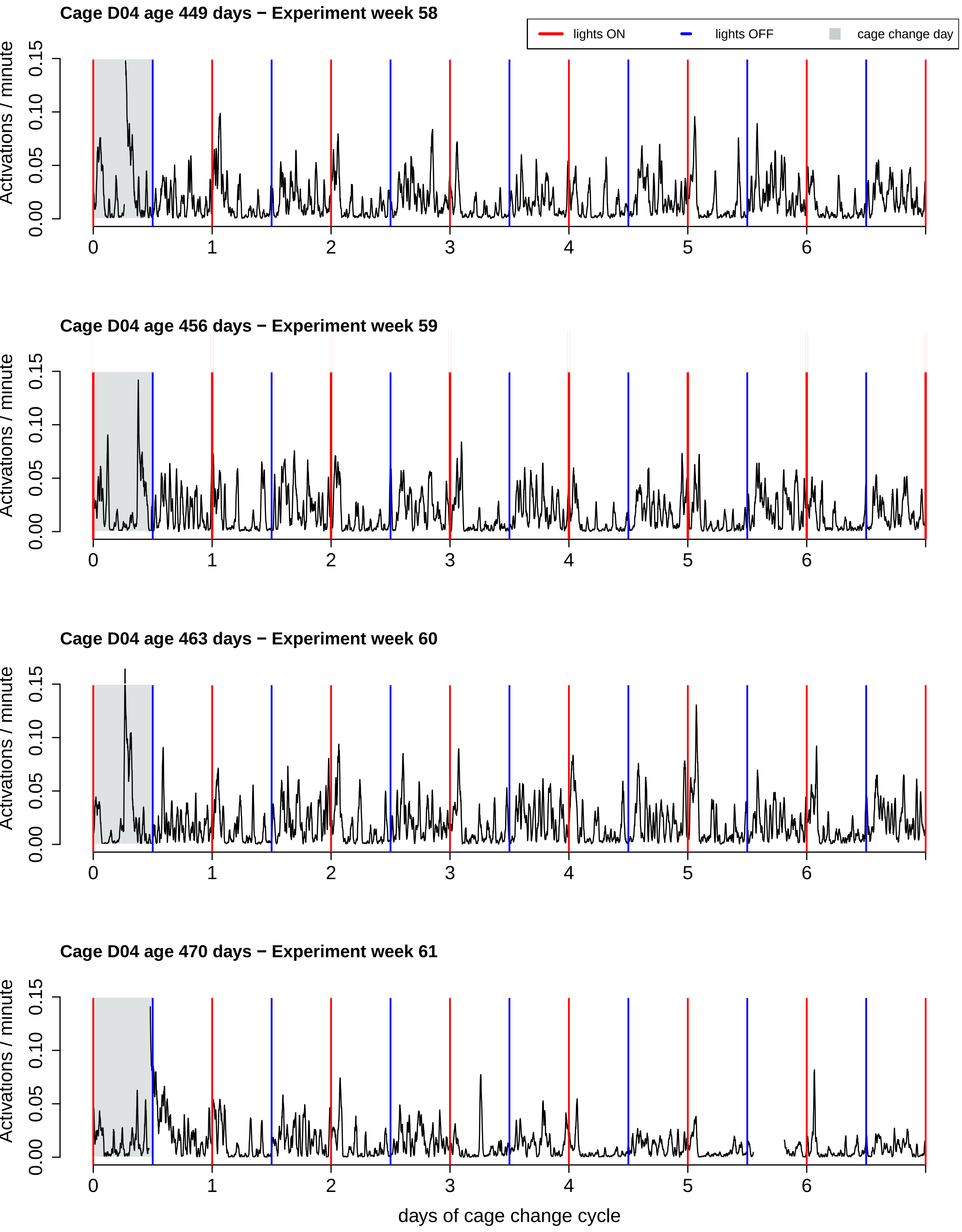
Cage D04 age 449 days − Experiment week 58
lights ON
lights OFF
cage change day
Activations / minute
0.00
0.05
0.10
0.15
0
1
2
3
4
5
6
Cage D04 age 456 days − Experiment week 59
Cage D04 age 463 days − Experiment week 60
Cage D04 age 470 days − Experiment week 61
days of cage change cycle

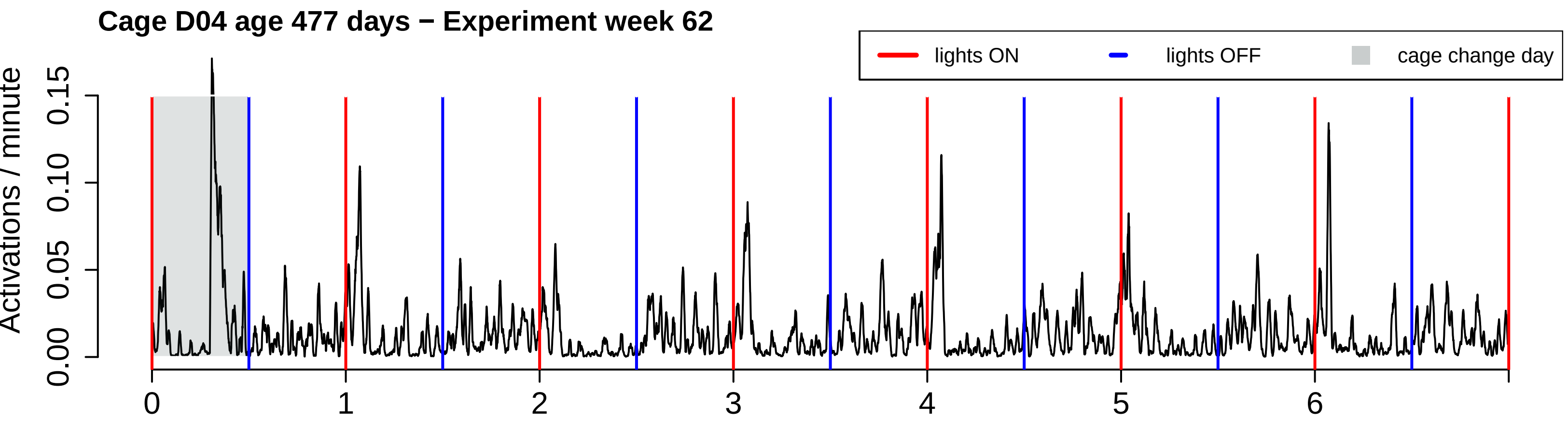

Cage D04 age 477 days − Experiment week 62
lights ON
lights OFF
cage change day
Activations / minute
0.00
0.05
0.10
0.15
0
1
2
3
4
5
6

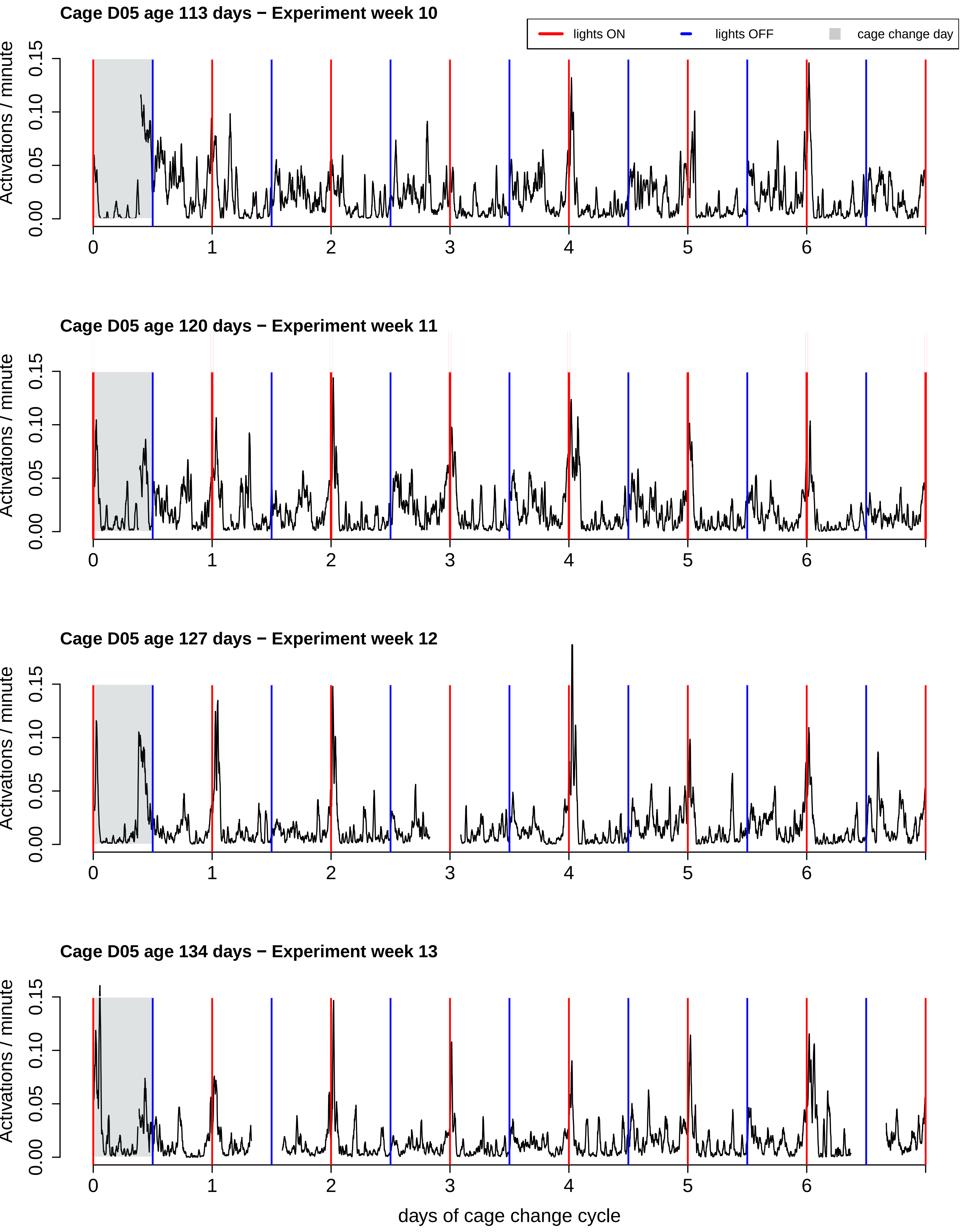
Cage D05 age 113 days − Experiment week 10
lights ON
lights OFF
cage change day
Cage D05 age 120 days − Experiment week 11
Cage D05 age 127 days − Experiment week 12
Cage D05 age 134 days − Experiment week 13
Activations / minute
0.00
0.05
0.10
0.15
0
1
2
3
4
5
6
days of cage change cycle

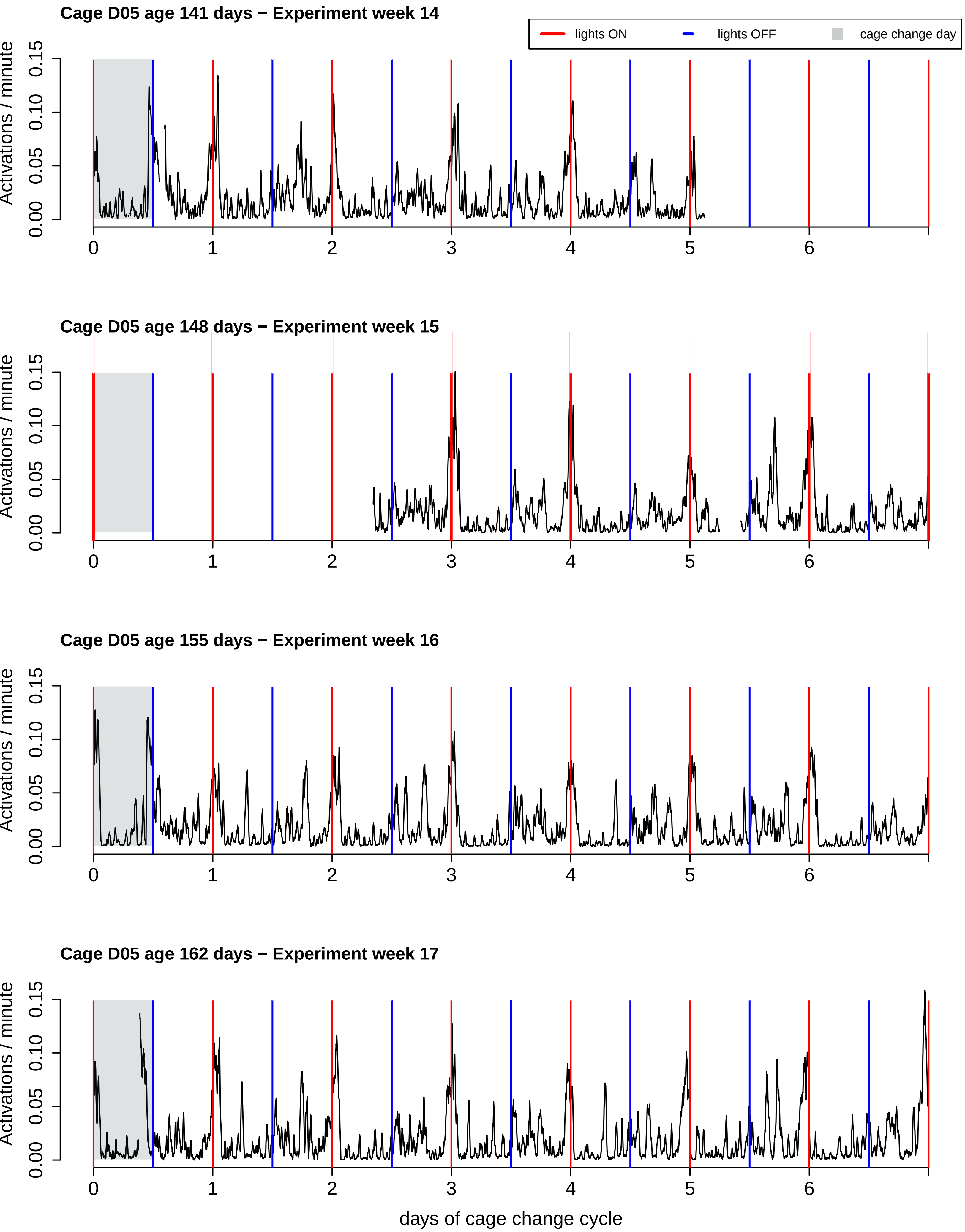

Cage D05 age 141 days − Experiment week 14
lights ON
lights OFF
cage change day
Activations / minute
0.00
0.05
0.10
0.15
0 1 2 3 4 5 6
Cage D05 age 148 days − Experiment week 15
Activations / minute
0.00
0.05
0.10
0.15
0 1 2 3 4 5 6
Cage D05 age 155 days − Experiment week 16
Activations / minute
0.00
0.05
0.10
0.15
0 1 2 3 4 5 6
Cage D05 age 162 days − Experiment week 17
Activations / minute
0.00
0.05
0.10
0.15
0 1 2 3 4 5 6
days of cage change cycle

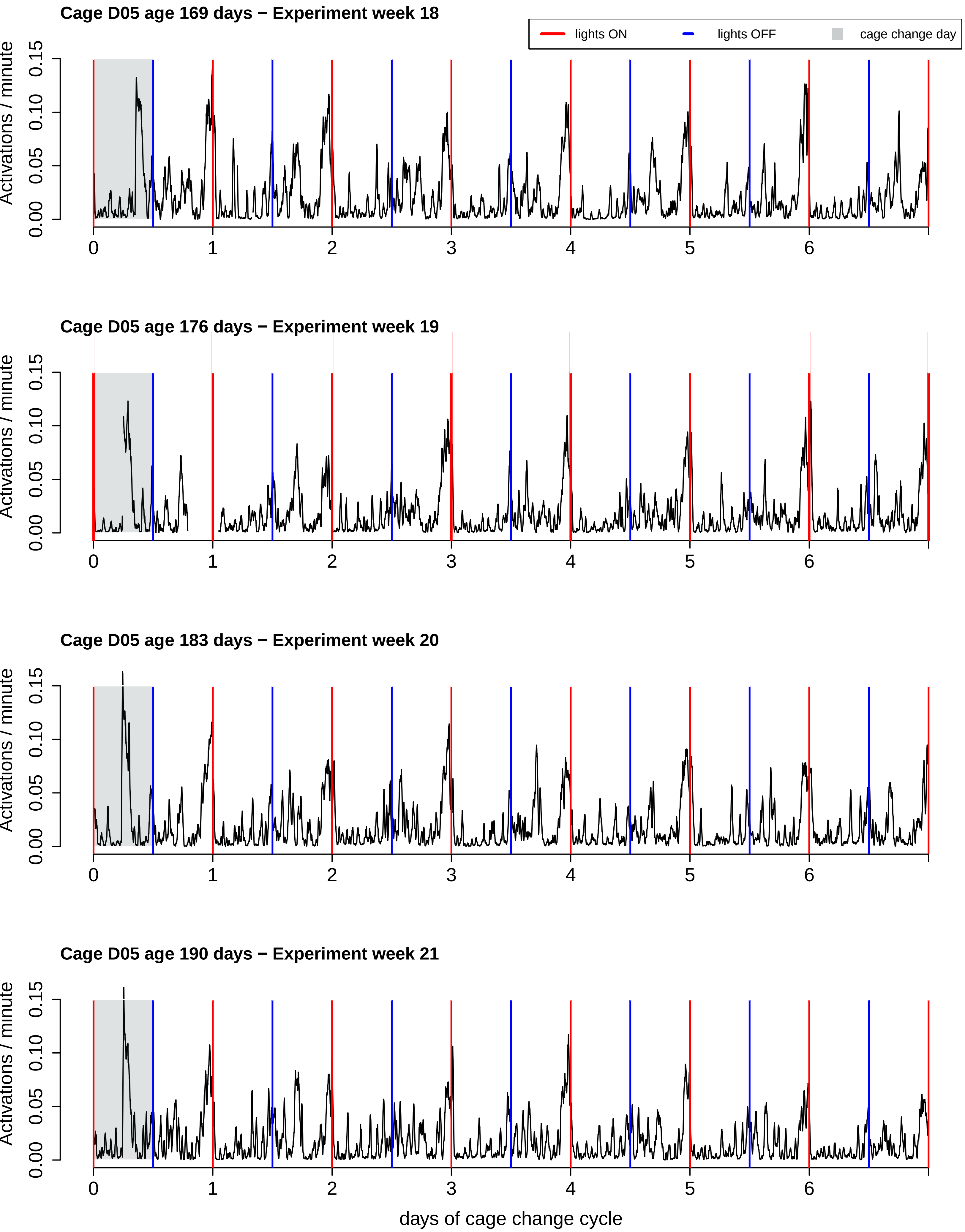
Cage D05 age 169 days − Experiment week 18
lights ON
lights OFF
cage change day
Cage D05 age 176 days − Experiment week 19
Cage D05 age 183 days − Experiment week 20
Cage D05 age 190 days − Experiment week 21
Activations / minute
days of cage change cycle

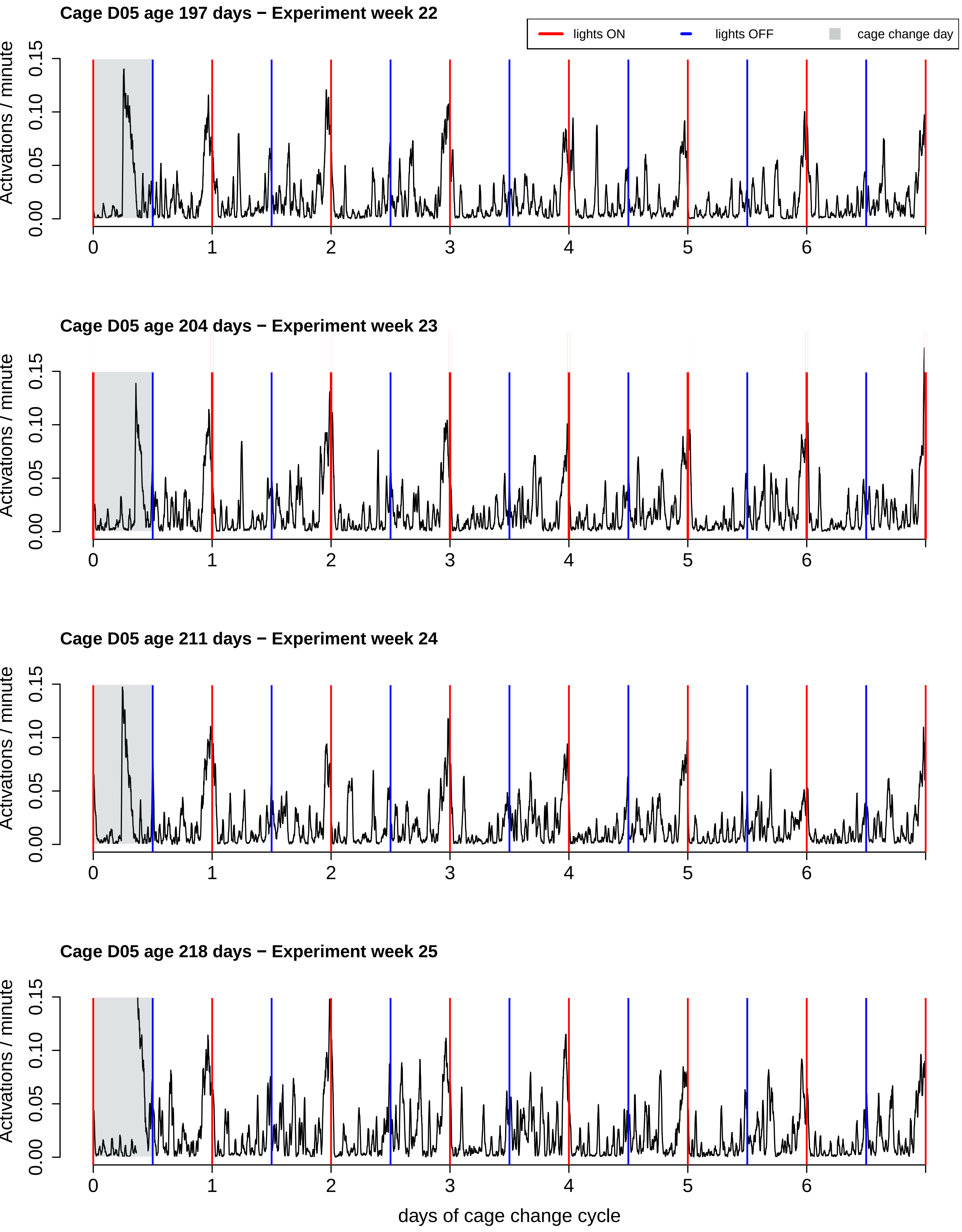

Cage D05 age 197 days − Experiment week 22
lights ON
lights OFF
cage change day
Cage D05 age 204 days − Experiment week 23
Cage D05 age 211 days − Experiment week 24
Cage D05 age 218 days − Experiment week 25
Activations / minute
0.00
0.05
0.10
0.15
0
1
2
3
4
5
6
days of cage change cycle

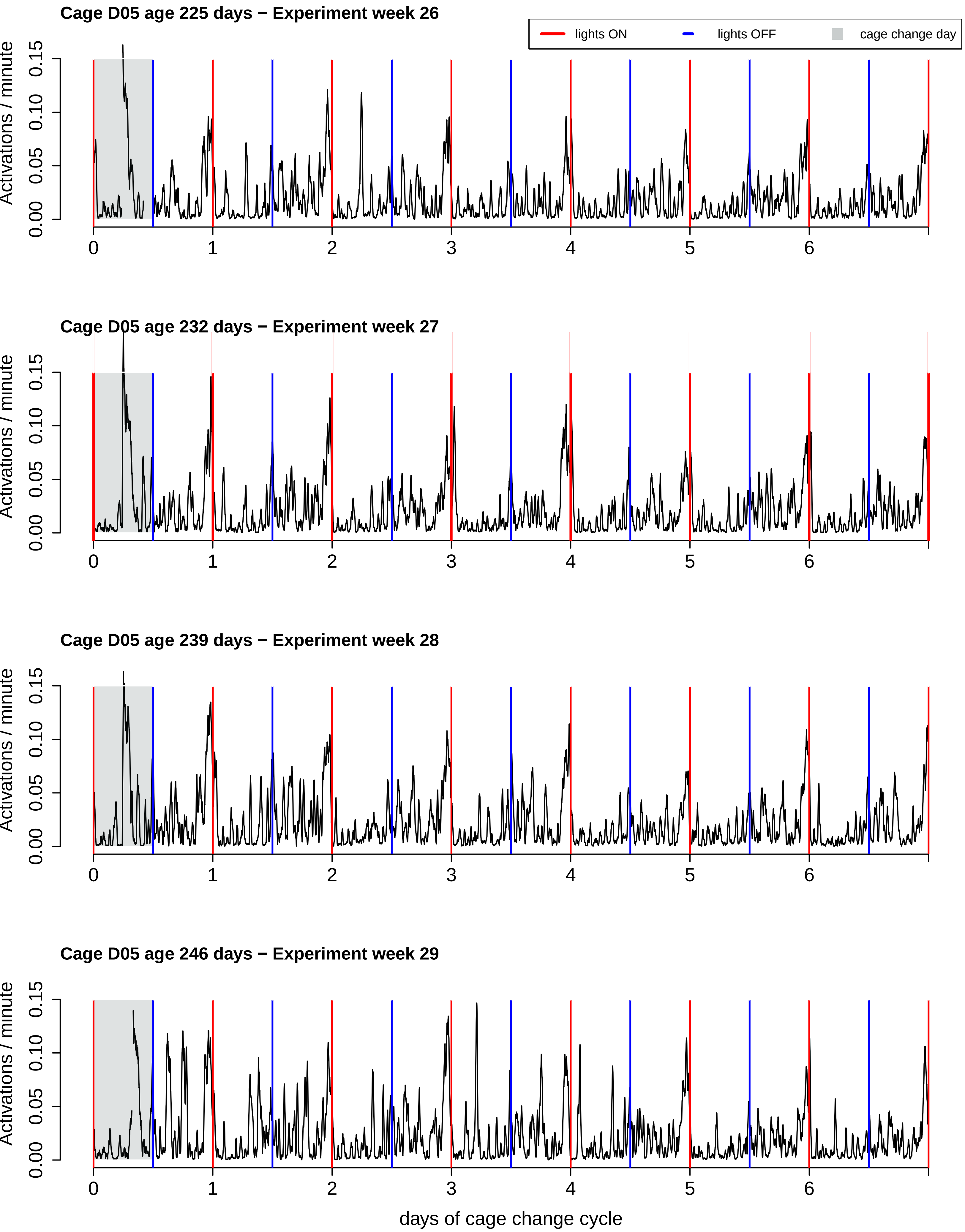
Cage D05 age 225 days − Experiment week 26
lights ON
lights OFF
cage change day
Activations / minute
0.00
0.05
0.10
0.15
0
1
2
3
4
5
6
Cage D05 age 232 days − Experiment week 27
Activations / minute
0.00
0.05
0.10
0.15
0
1
2
3
4
5
6
Cage D05 age 239 days − Experiment week 28
Activations / minute
0.00
0.05
0.10
0.15
0
1
2
3
4
5
6
Cage D05 age 246 days − Experiment week 29
Activations / minute
0.00
0.05
0.10
0.15
0
1
2
3
4
5
6
days of cage change cycle

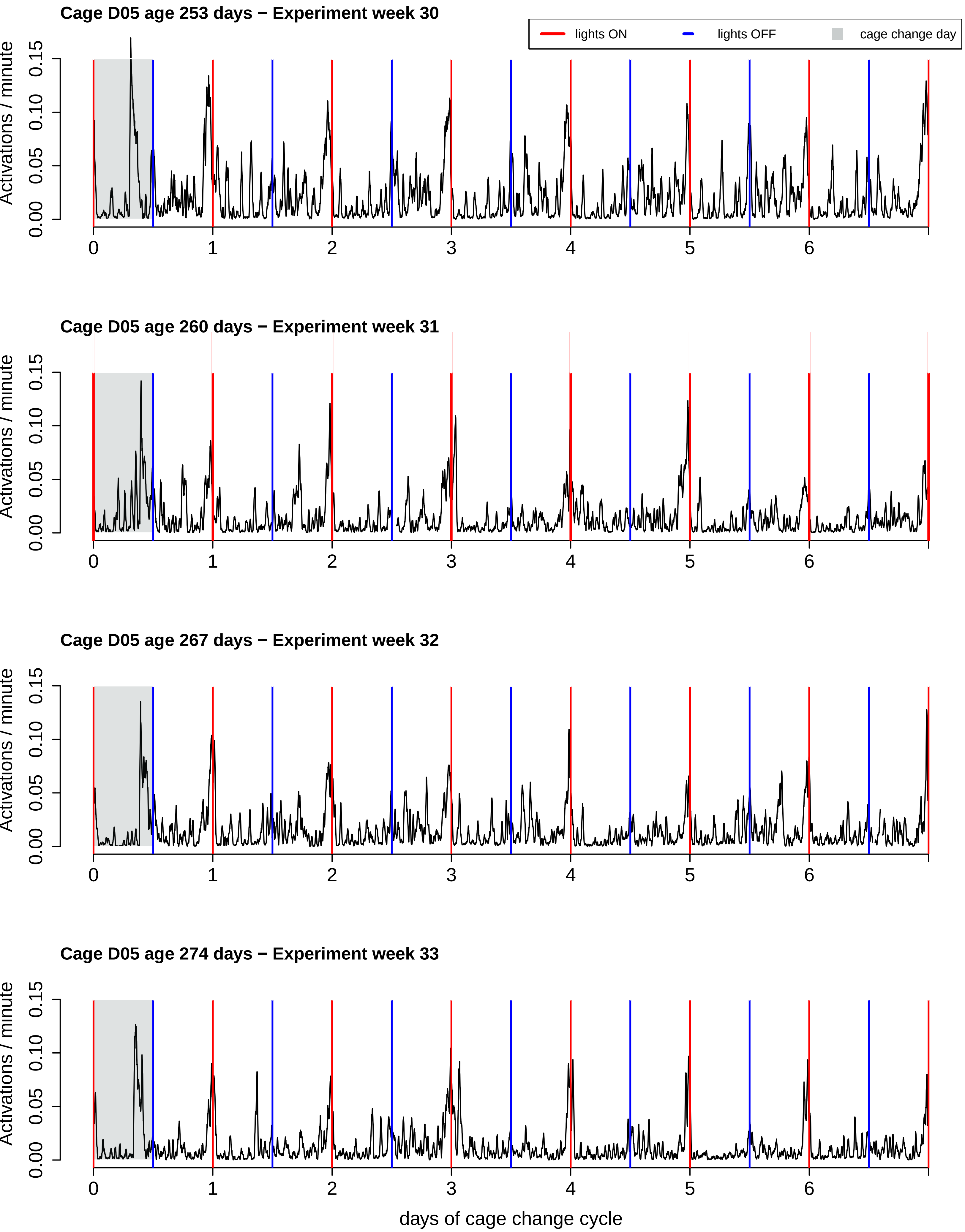
Cage D05 age 253 days − Experiment week 30
lights ON
lights OFF
cage change day
Activations / minute
0.00
0.05
0.10
0.15
0
1
2
3
4
5
6
Cage D05 age 260 days − Experiment week 31
Cage D05 age 267 days − Experiment week 32
Cage D05 age 274 days − Experiment week 33
days of cage change cycle

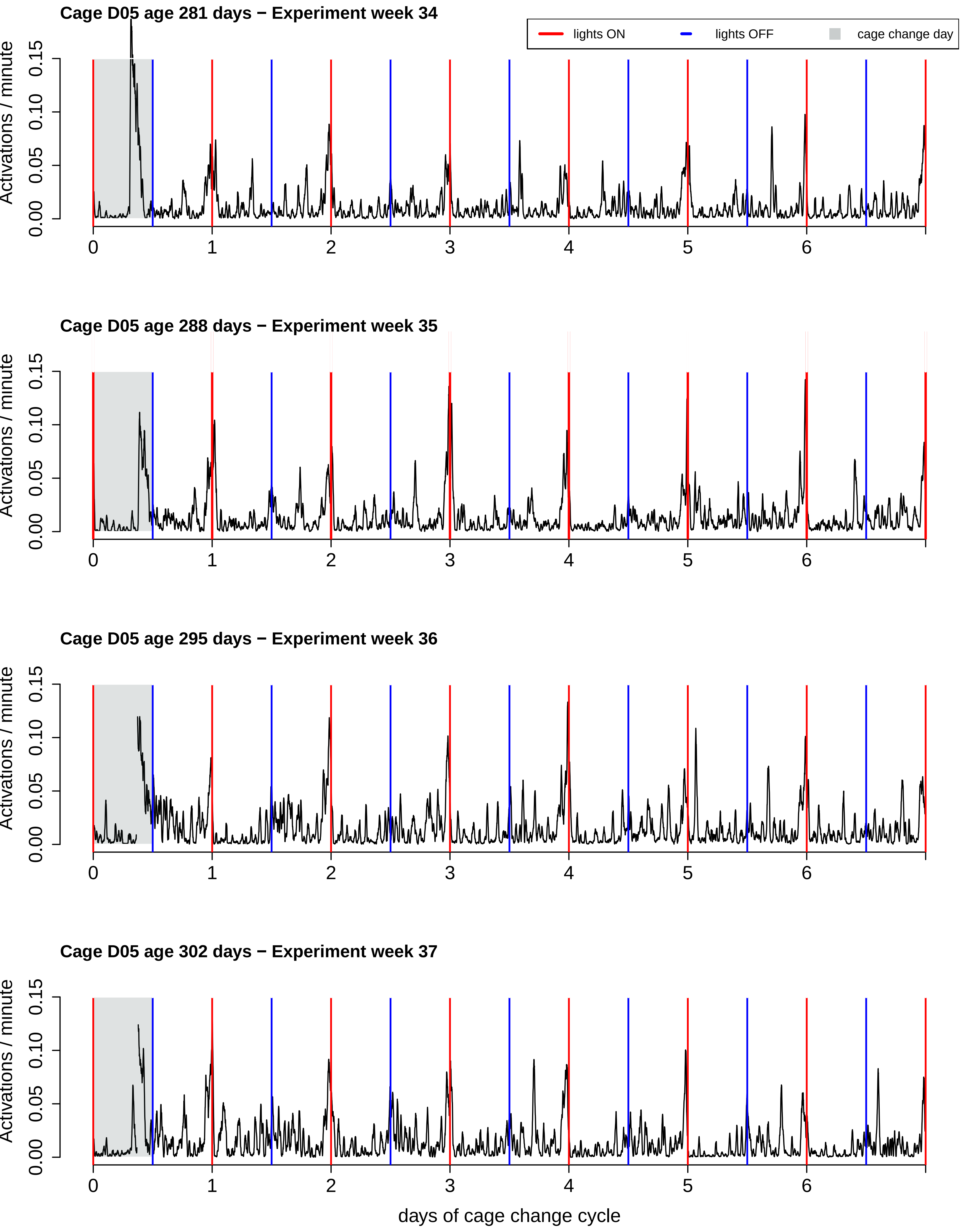
Cage D05 age 281 days − Experiment week 34
lights ON
lights OFF
cage change day
Activations / minute
0.00
0.05
0.10
0.15
0
1
2
3
4
5
6
Cage D05 age 288 days − Experiment week 35
Activations / minute
0.00
0.05
0.10
0.15
0
1
2
3
4
5
6
Cage D05 age 295 days − Experiment week 36
Activations / minute
0.00
0.05
0.10
0.15
0
1
2
3
4
5
6
Cage D05 age 302 days − Experiment week 37
Activations / minute
0.00
0.05
0.10
0.15
0
1
2
3
4
5
6
days of cage change cycle

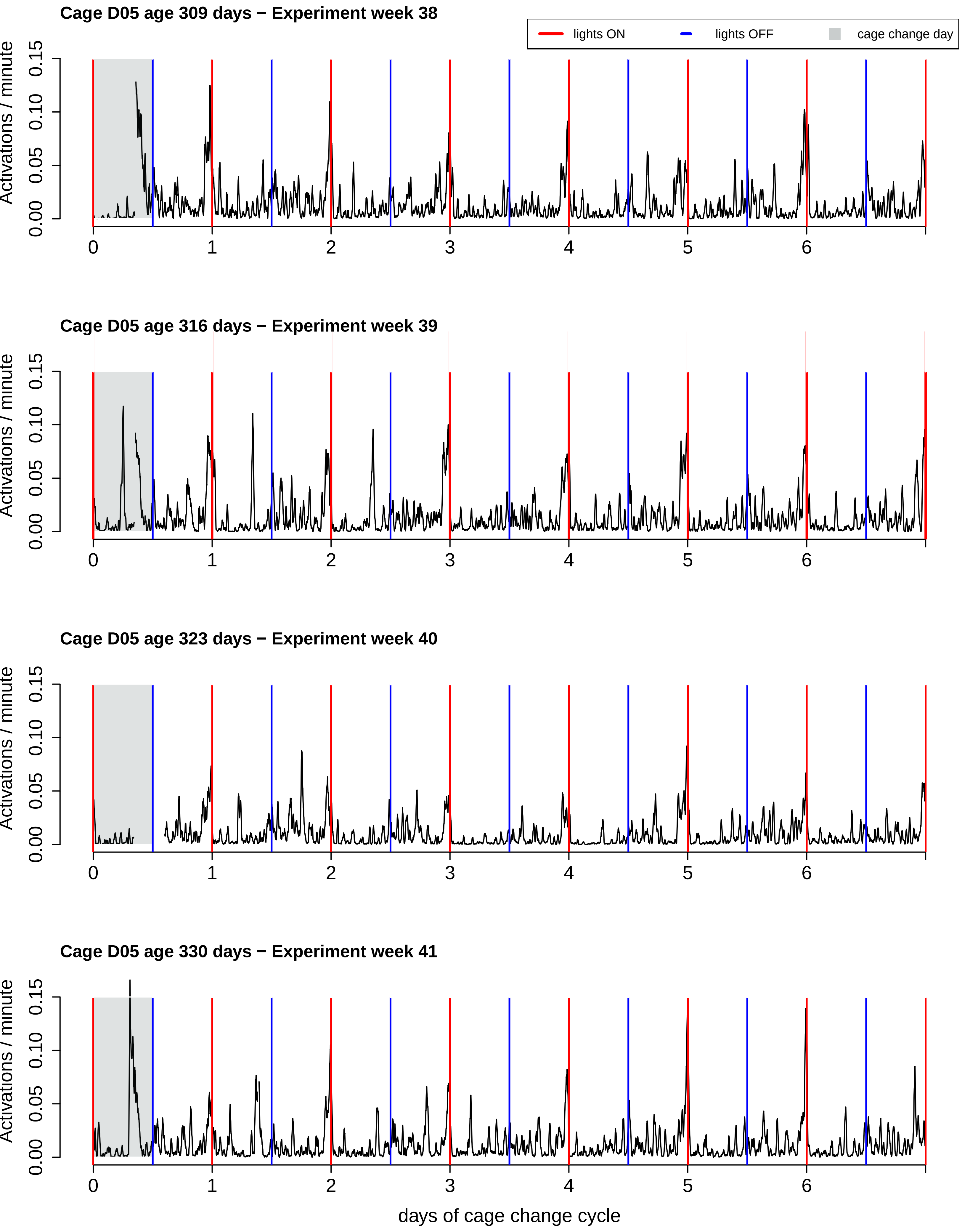
Cage D05 age 309 days − Experiment week 38
lights ON
lights OFF
cage change day
Activations / minute
0.00
0.05
0.10
0.15
0
1
2
3
4
5
6
Cage D05 age 316 days − Experiment week 39
Cage D05 age 323 days − Experiment week 40
Cage D05 age 330 days − Experiment week 41
days of cage change cycle

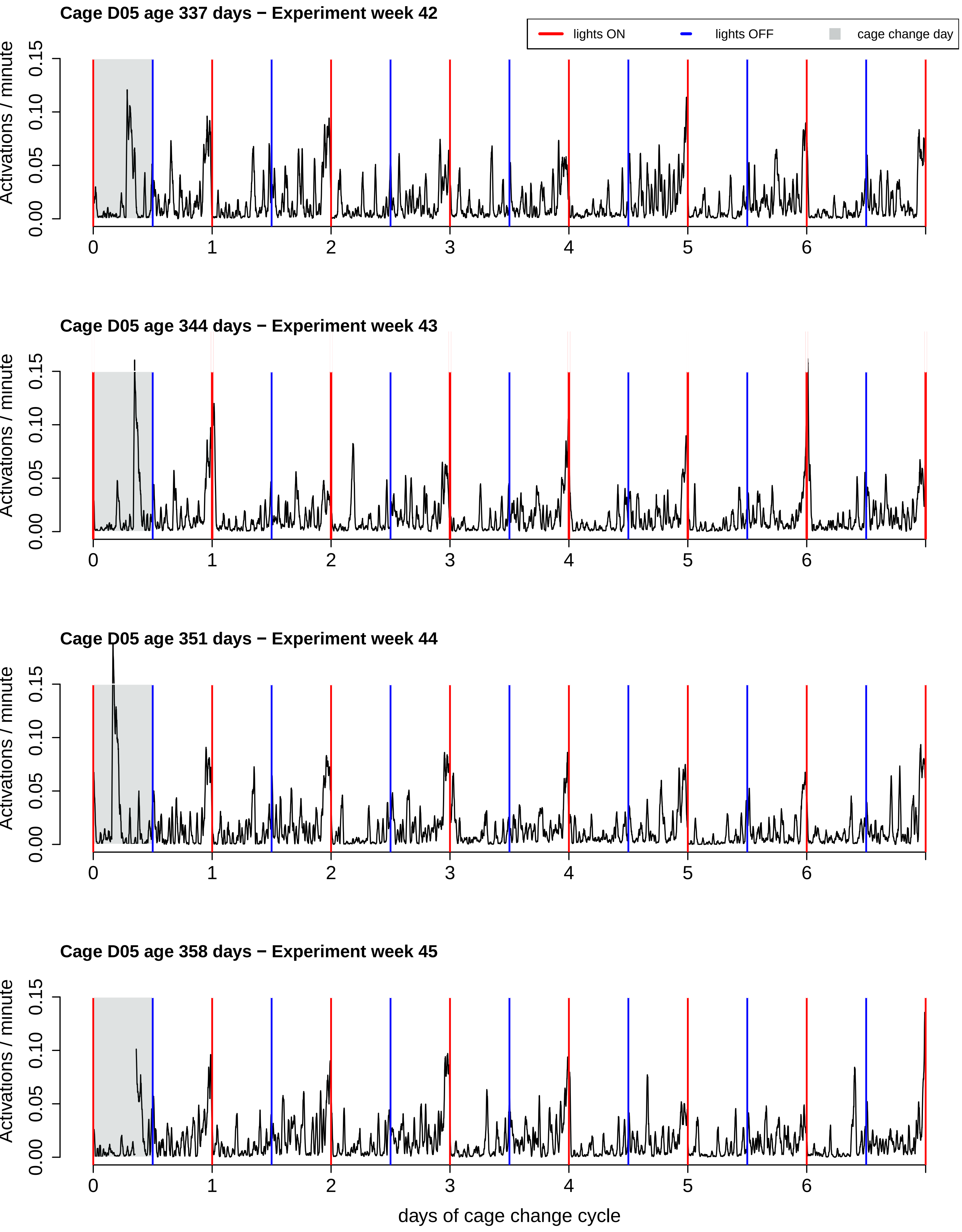

Cage D05 age 337 days − Experiment week 42
lights ON
lights OFF
cage change day
Activations / minute
0.00
0.05
0.10
0.15
0
1
2
3
4
5
6
Cage D05 age 344 days − Experiment week 43
Cage D05 age 351 days − Experiment week 44
Cage D05 age 358 days − Experiment week 45
days of cage change cycle

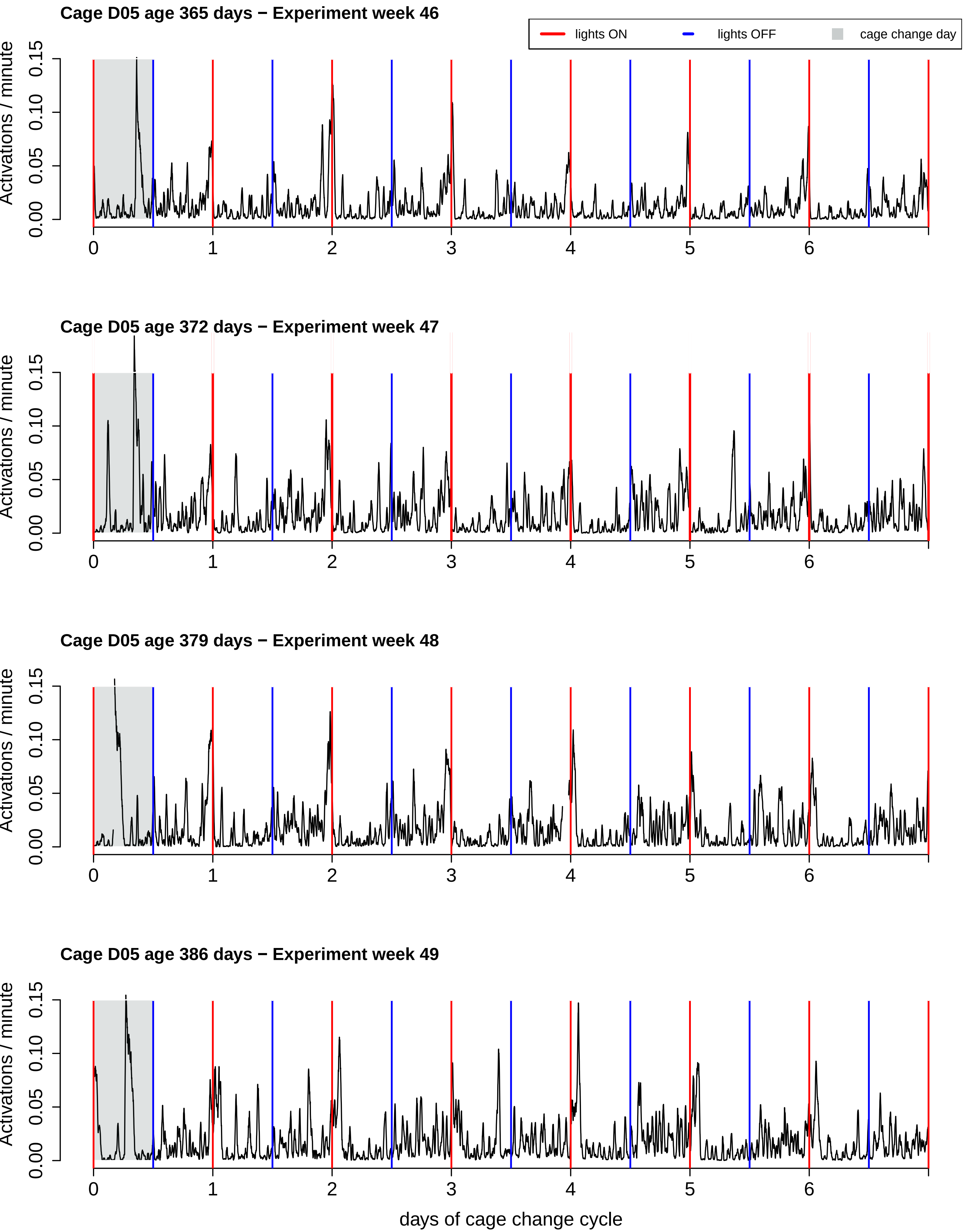

Cage D05 age 365 days − Experiment week 46
lights ON
lights OFF
cage change day
Cage D05 age 372 days − Experiment week 47
Cage D05 age 379 days − Experiment week 48
Cage D05 age 386 days − Experiment week 49
Activations / minute
0.00
0.05
0.10
0.15
0
1
2
3
4
5
6
days of cage change cycle

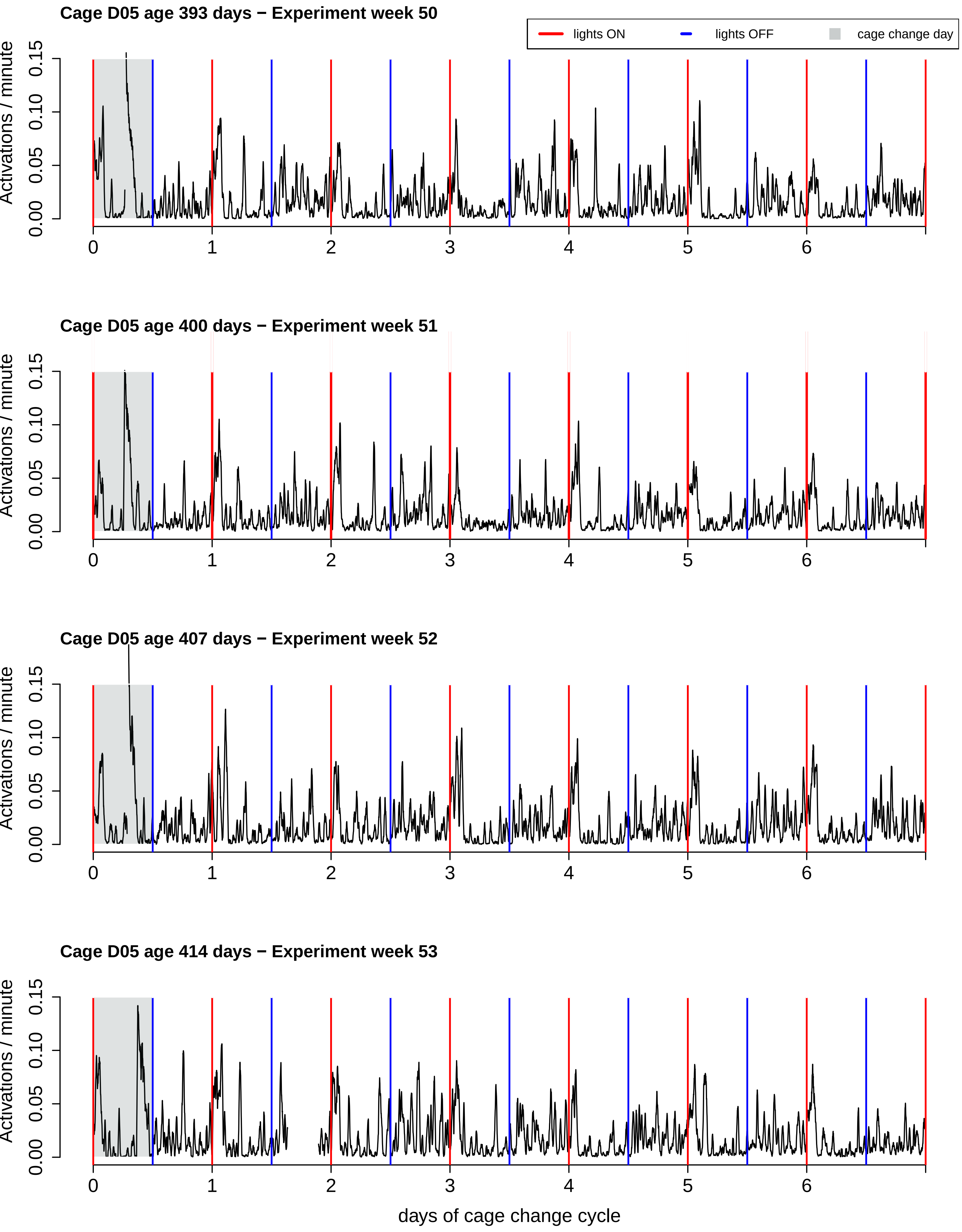

Cage D05 age 393 days − Experiment week 50
lights ON
lights OFF
cage change day
Cage D05 age 400 days − Experiment week 51
Cage D05 age 407 days − Experiment week 52
Cage D05 age 414 days − Experiment week 53
Activations / minute
0.00
0.05
0.10
0.15
0
1
2
3
4
5
6
days of cage change cycle

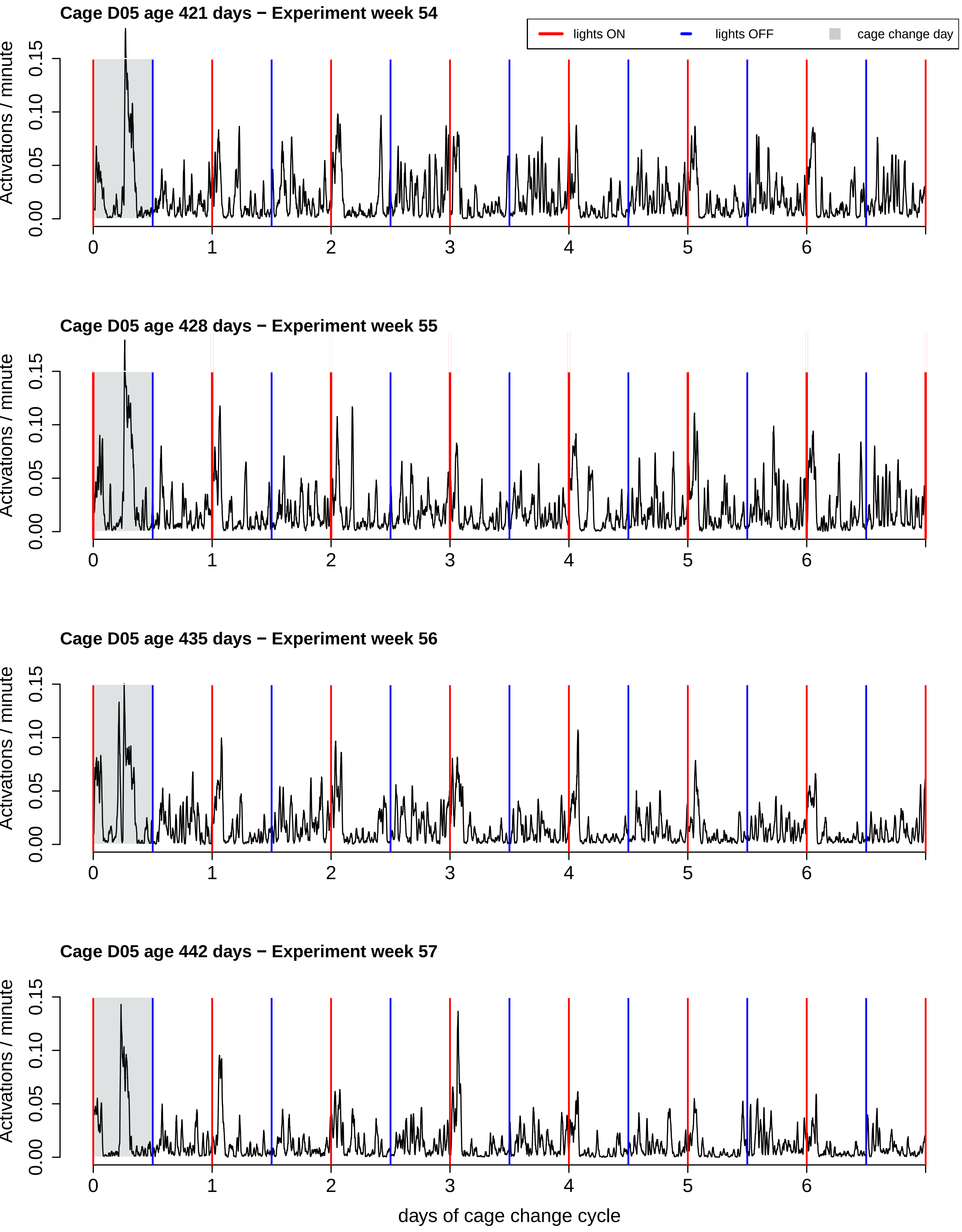

Cage D05 age 421 days − Experiment week 54
lights ON
lights OFF
cage change day
Activations / minute
0.00
0.05
0.10
0.15
0
1
2
3
4
5
6
Cage D05 age 428 days − Experiment week 55
Activations / minute
Cage D05 age 435 days − Experiment week 56
Activations / minute
Cage D05 age 442 days − Experiment week 57
Activations / minute
days of cage change cycle

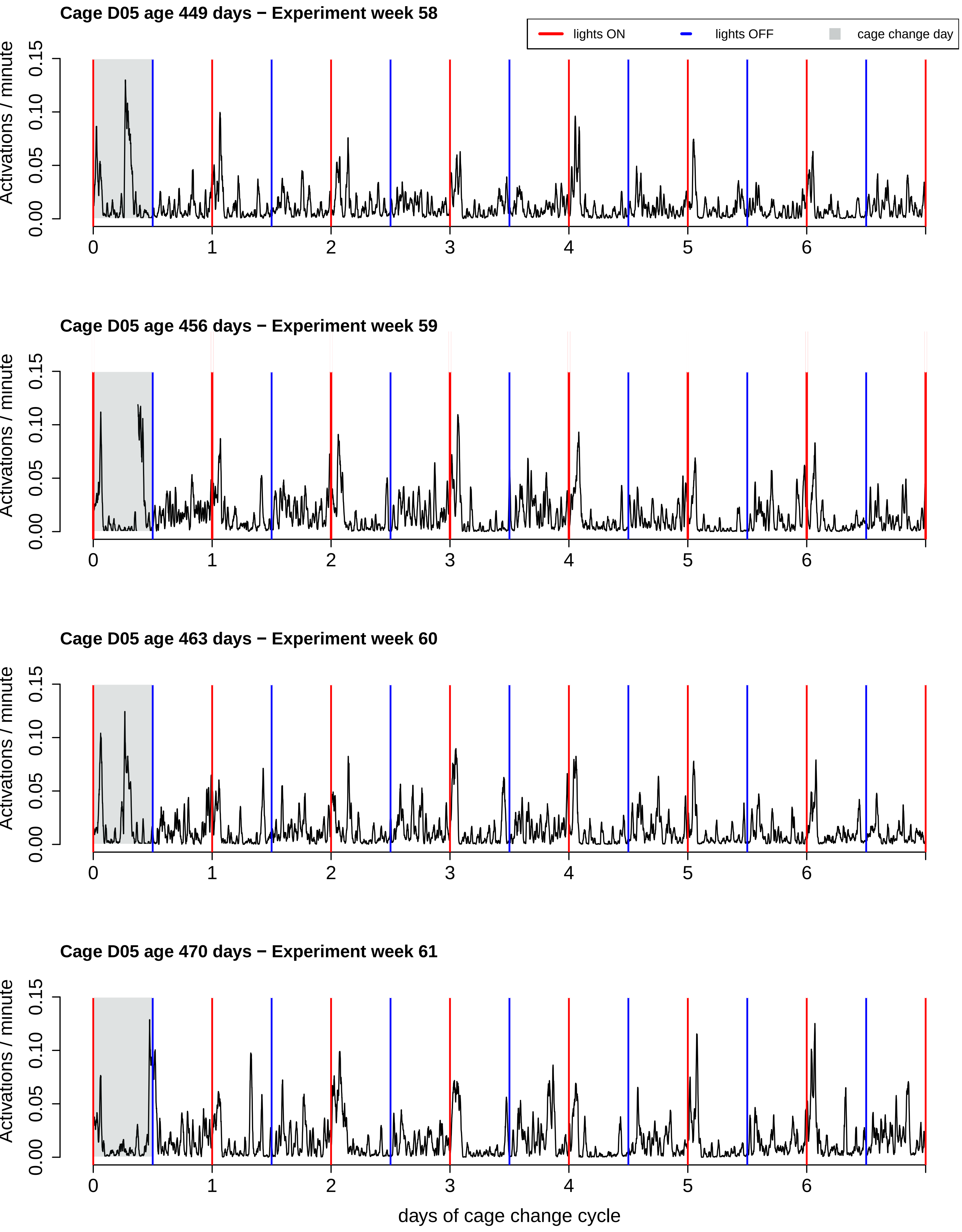
Cage D05 age 449 days − Experiment week 58
lights ON
lights OFF
cage change day
Activations / minute
0.00
0.05
0.10
0.15
0
1
2
3
4
5
6
Cage D05 age 456 days − Experiment week 59
Cage D05 age 463 days − Experiment week 60
Cage D05 age 470 days − Experiment week 61
days of cage change cycle

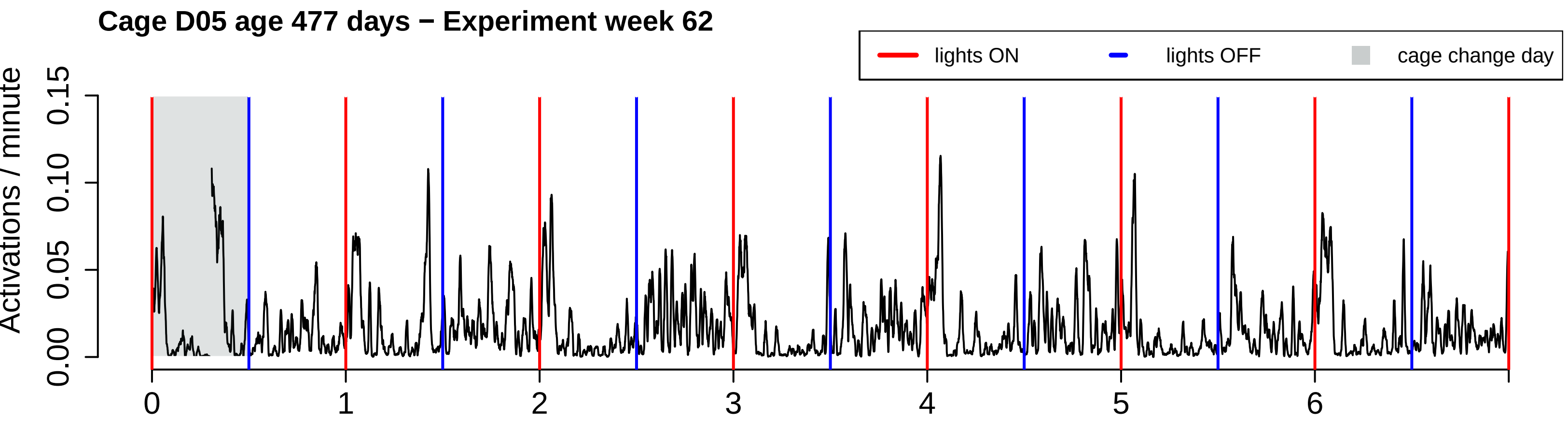
Cage D05 age 477 days − Experiment week 62
lights ON
lights OFF
cage change day
Activations / minute
0.00
0.05
0.10
0.15
0
1
2
3
4
5
6

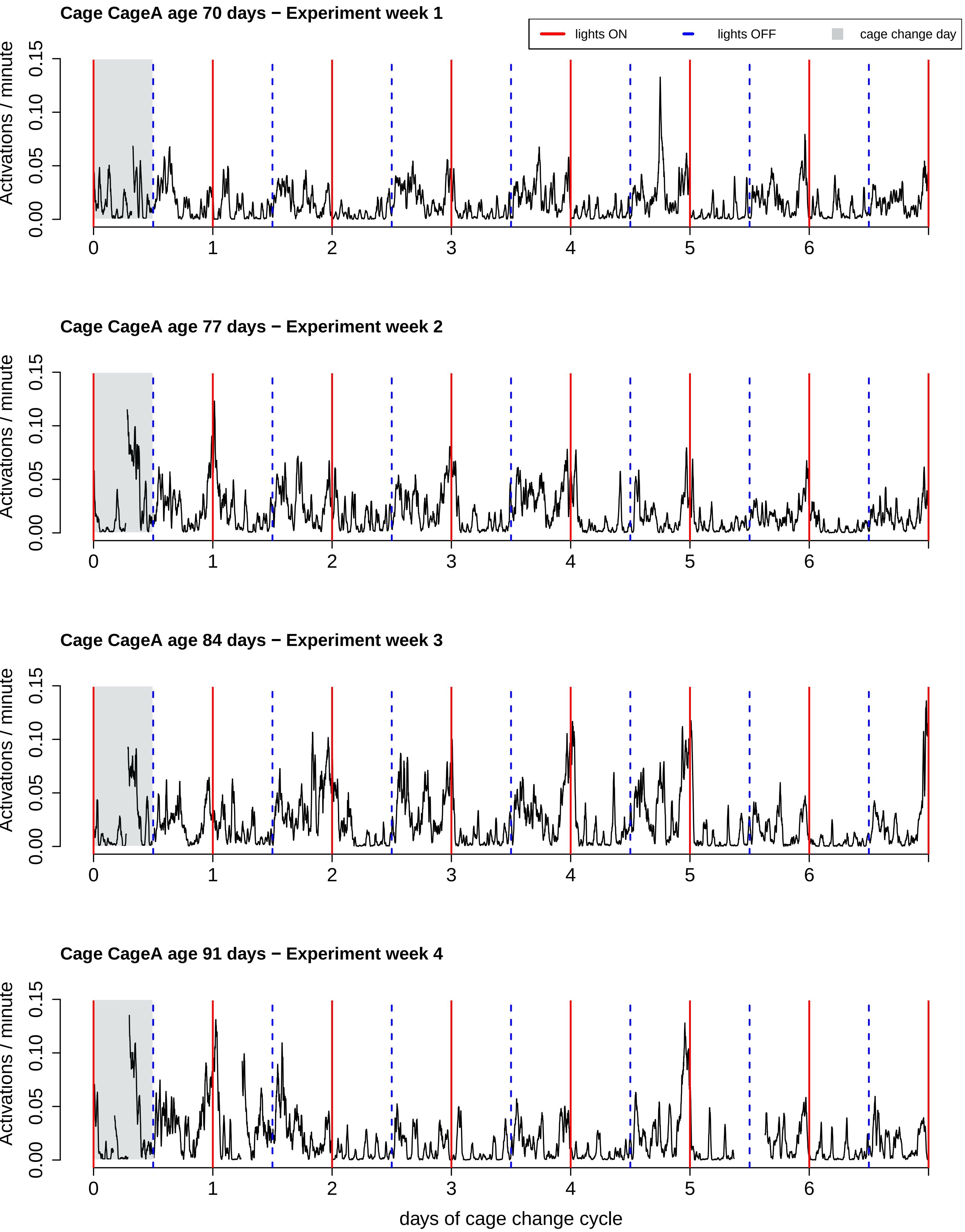
Cage CageA age 70 days − Experiment week 1
lights ON
lights OFF
cage change day
Activations / minute
0.00
0.05
0.10
0.15
0
1
2
3
4
5
6
Cage CageA age 77 days − Experiment week 2
Cage CageA age 84 days − Experiment week 3
Cage CageA age 91 days − Experiment week 4
days of cage change cycle

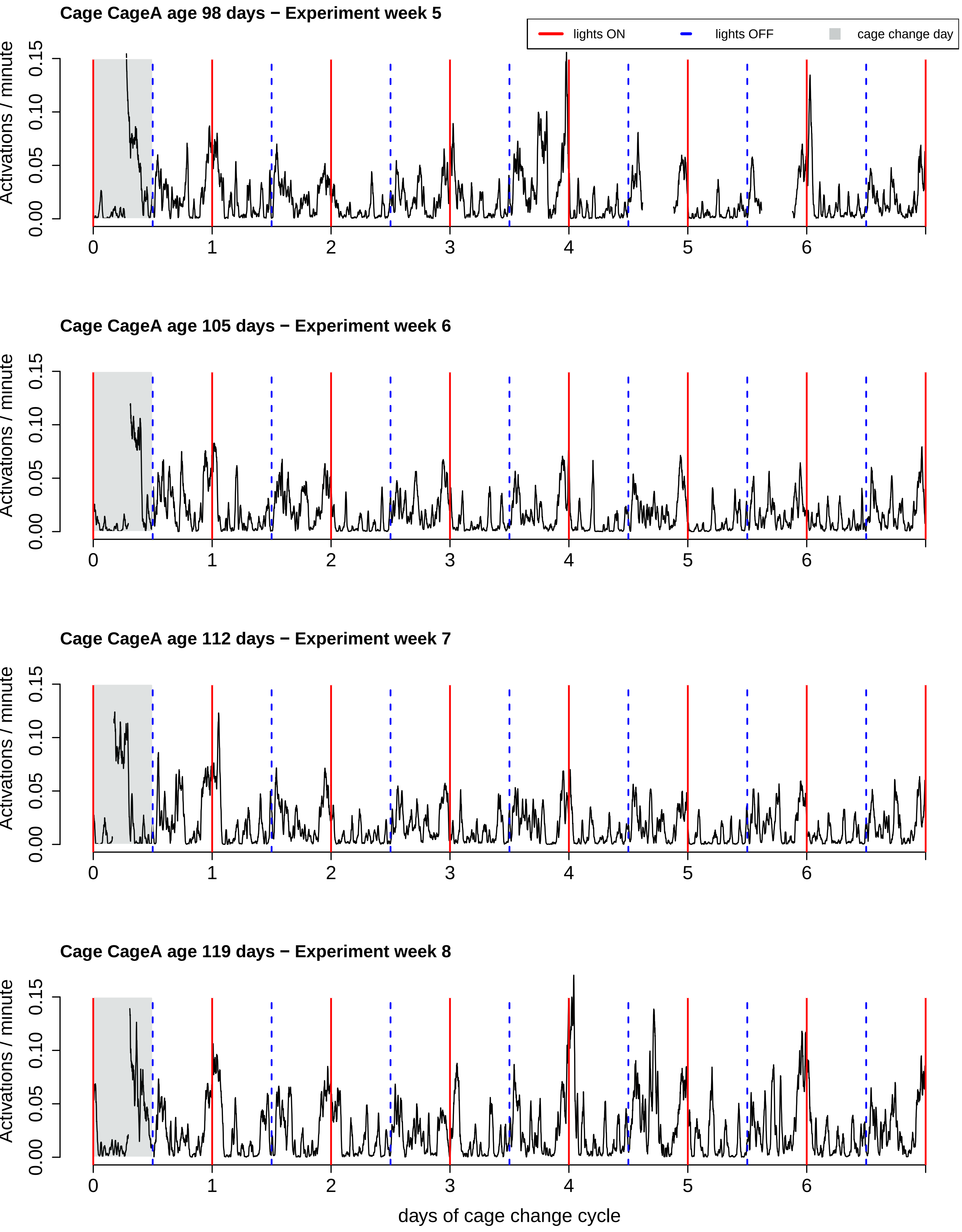

Cage CageA age 98 days − Experiment week 5
lights ON
lights OFF
cage change day
Activations / minute
0.00
0.05
0.10
0.15
0
1
2
3
4
5
6
Cage CageA age 105 days − Experiment week 6
Cage CageA age 112 days − Experiment week 7
Cage CageA age 119 days − Experiment week 8
days of cage change cycle

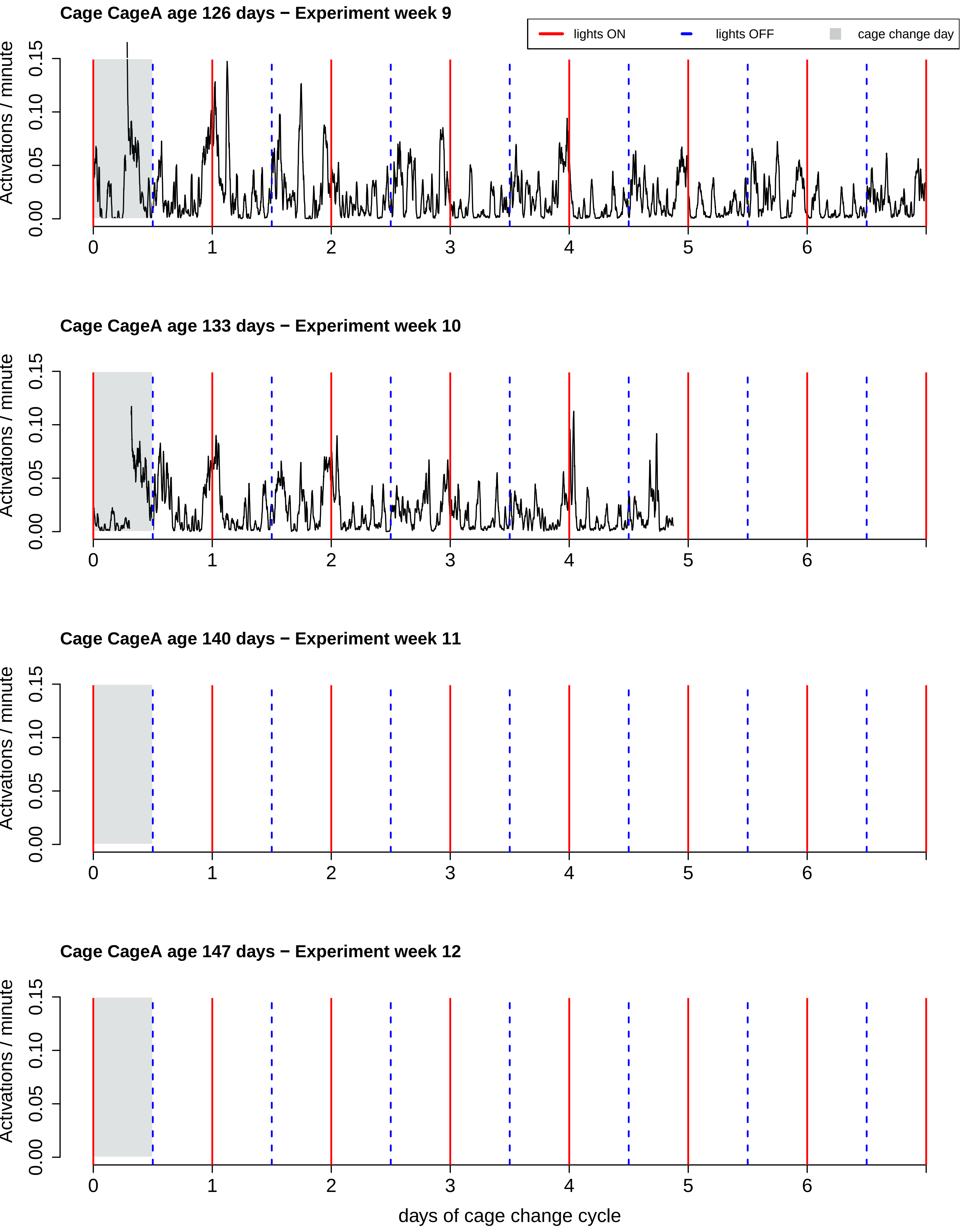

Cage CageA age 126 days − Experiment week 9
lights ON
lights OFF
cage change day
Activations / minute
0.00
0.05
0.10
0.15
0
1
2
3
4
5
6
Cage CageA age 133 days − Experiment week 10
Cage CageA age 140 days − Experiment week 11
Cage CageA age 147 days − Experiment week 12
days of cage change cycle

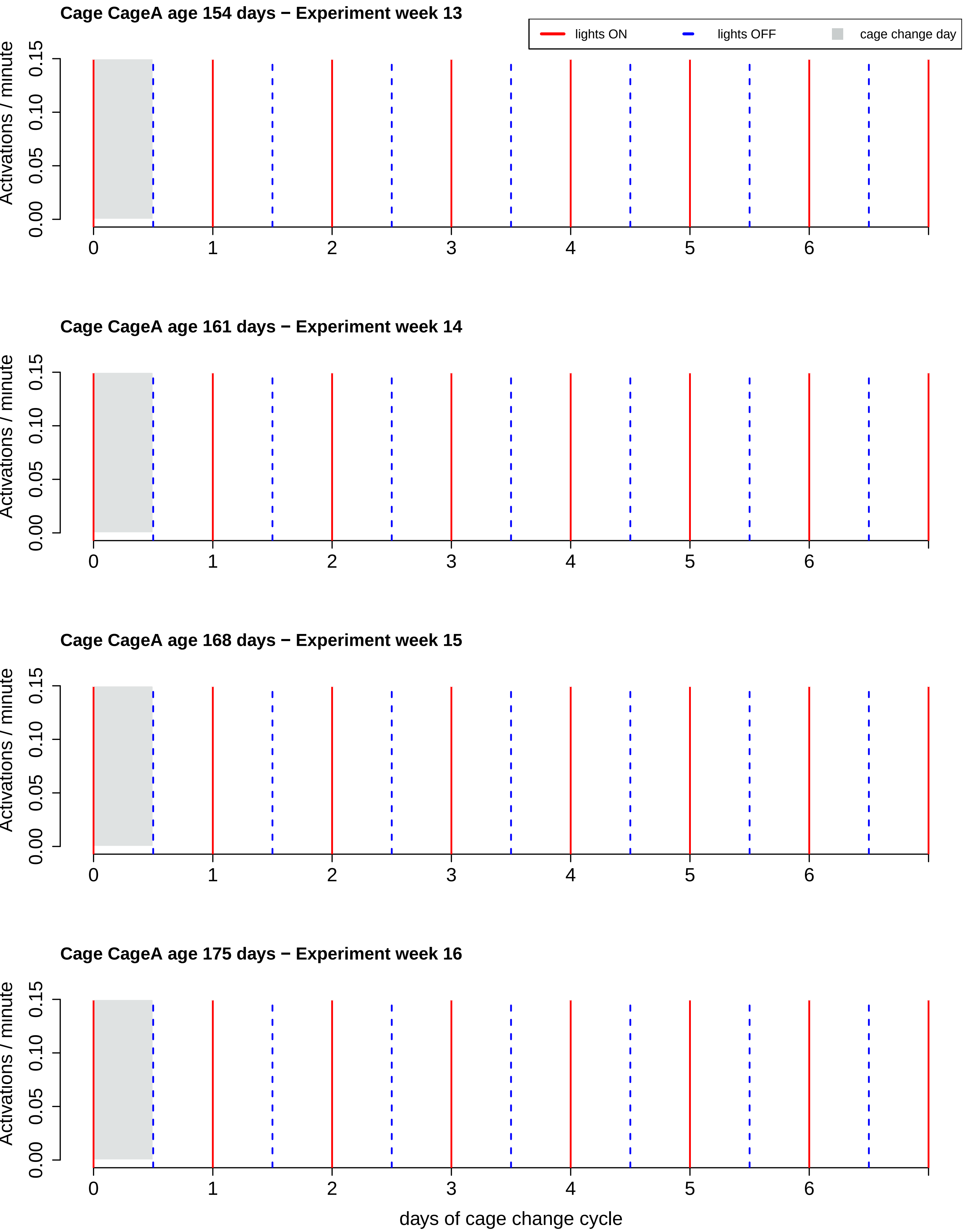
Cage CageA age 154 days − Experiment week 13
lights ON
lights OFF
cage change day
Activations / minute
0.00
0.05
0.10
0.15
0
1
2
3
4
5
6
Cage CageA age 161 days − Experiment week 14
Cage CageA age 168 days − Experiment week 15
Cage CageA age 175 days − Experiment week 16
days of cage change cycle

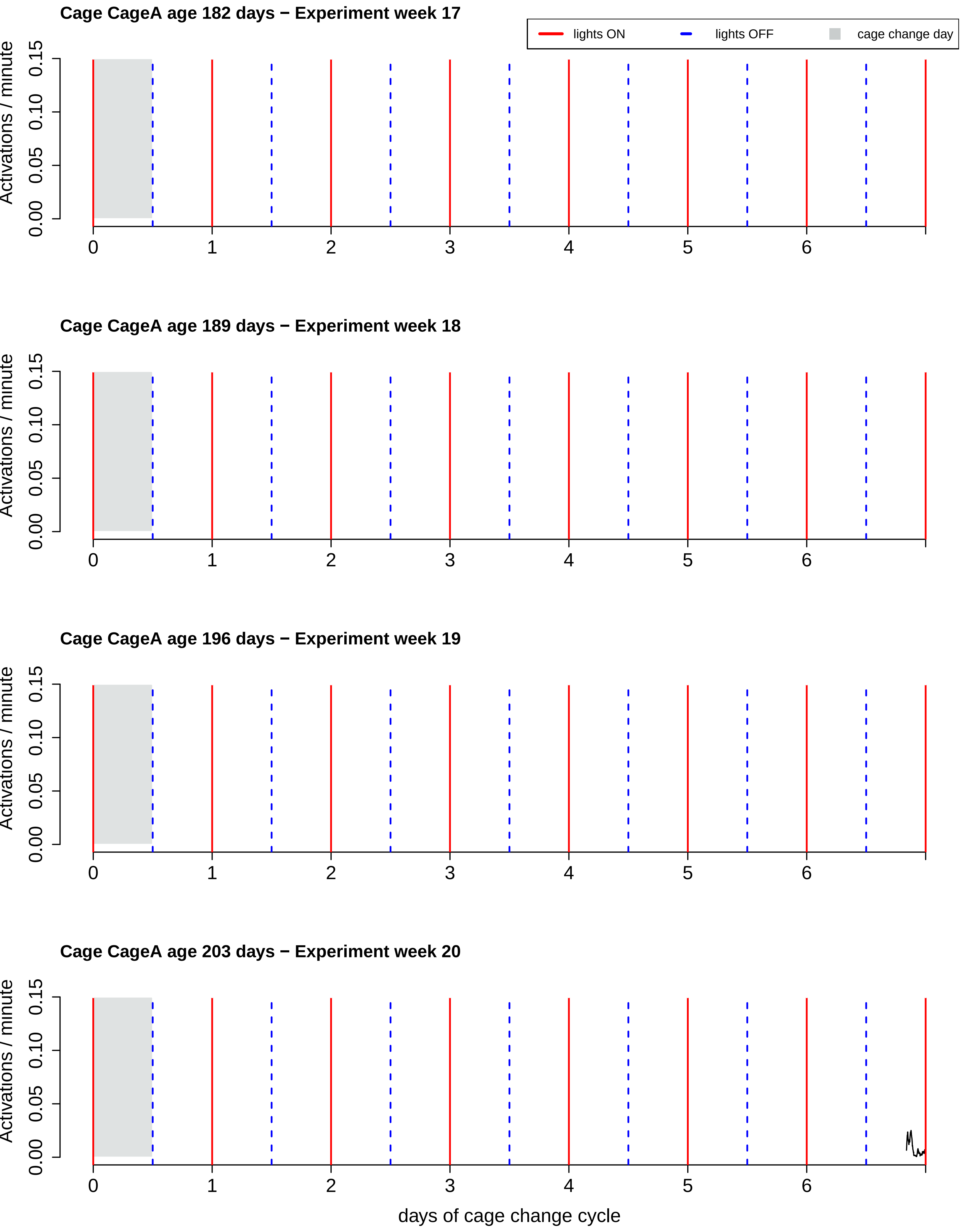
Cage CageA age 182 days − Experiment week 17
lights ON
lights OFF
cage change day
Activations / minute
0.00
0.05
0.10
0.15
0
1
2
3
4
5
6
Cage CageA age 189 days − Experiment week 18
Activations / minute
0.00
0.05
0.10
0.15
0
1
2
3
4
5
6
Cage CageA age 196 days − Experiment week 19
Activations / minute
0.00
0.05
0.10
0.15
0
1
2
3
4
5
6
Cage CageA age 203 days − Experiment week 20
Activations / minute
0.00
0.05
0.10
0.15
0
1
2
3
4
5
6
days of cage change cycle

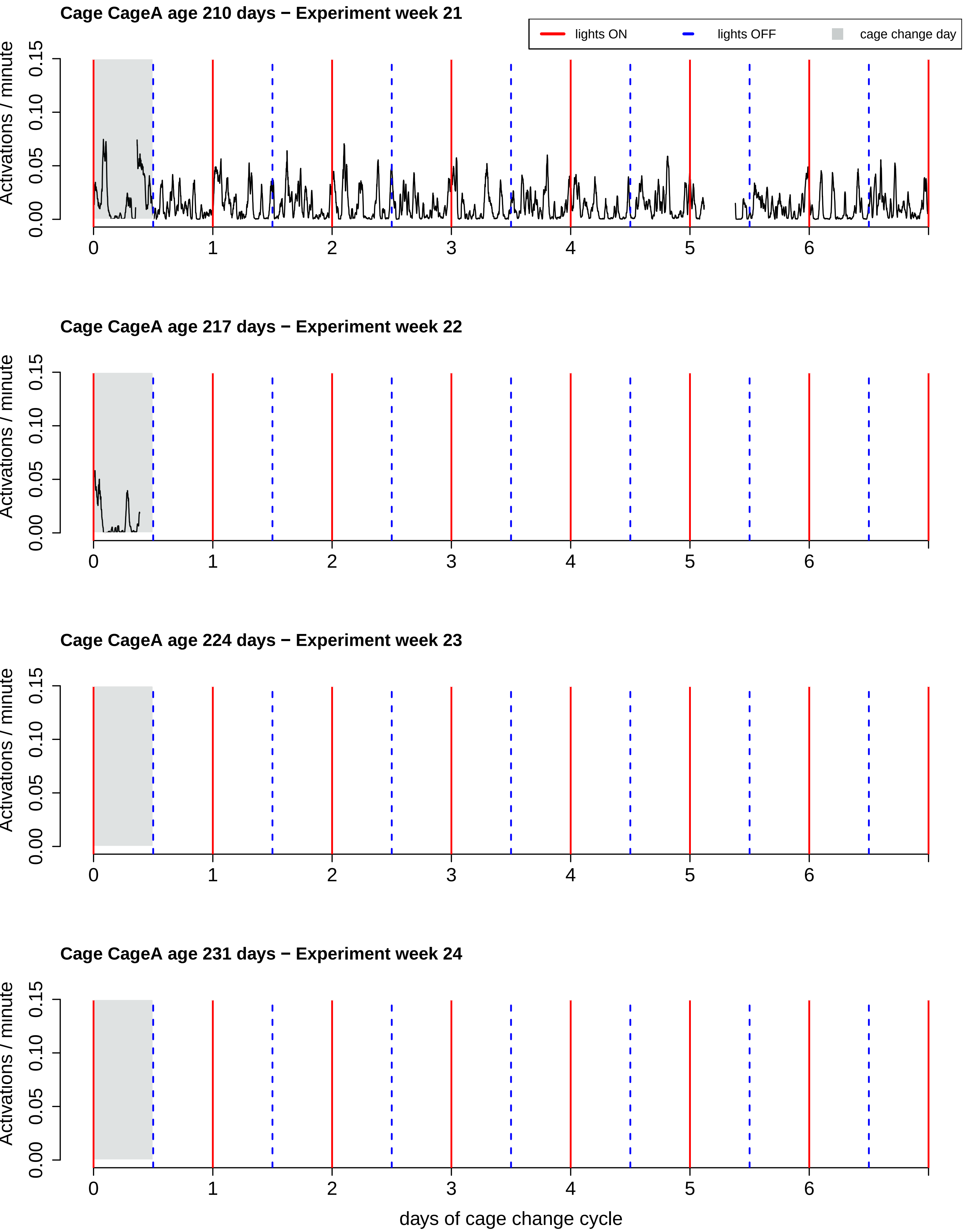
Cage CageA age 210 days − Experiment week 21
lights ON
lights OFF
cage change day
Activations / minute
0.00
0.05
0.10
0.15
0
1
2
3
4
5
6
Cage CageA age 217 days − Experiment week 22
Activations / minute
0.00
0.05
0.10
0.15
0
1
2
3
4
5
6
Cage CageA age 224 days − Experiment week 23
Activations / minute
0.00
0.05
0.10
0.15
0
1
2
3
4
5
6
Cage CageA age 231 days − Experiment week 24
Activations / minute
0.00
0.05
0.10
0.15
0
1
2
3
4
5
6
days of cage change cycle

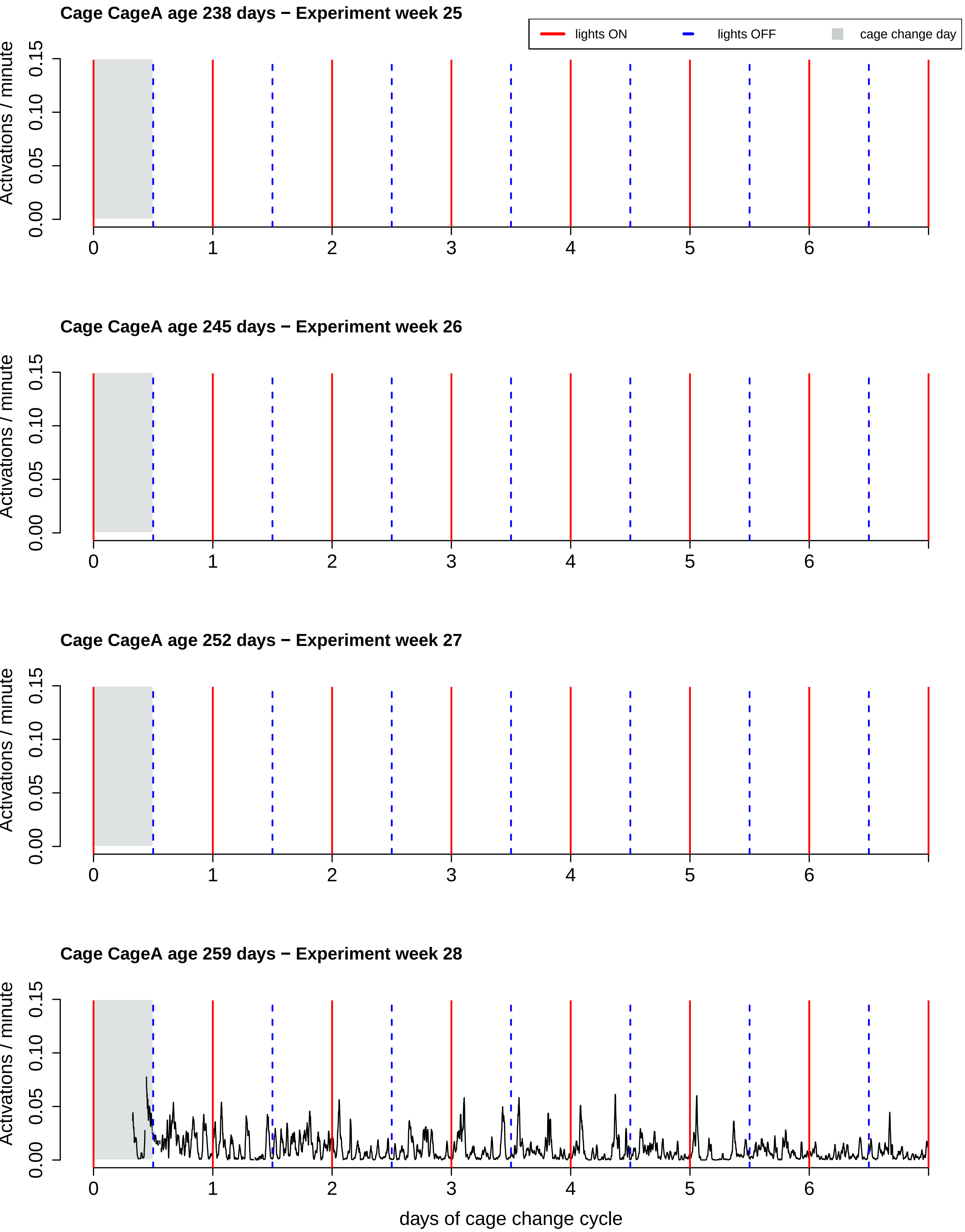
Cage CageA age 238 days − Experiment week 25
lights ON
lights OFF
cage change day
Activations / minute
0.00
0.05
0.10
0.15
0
1
2
3
4
5
6
Cage CageA age 245 days − Experiment week 26
Cage CageA age 252 days − Experiment week 27
Cage CageA age 259 days − Experiment week 28
days of cage change cycle

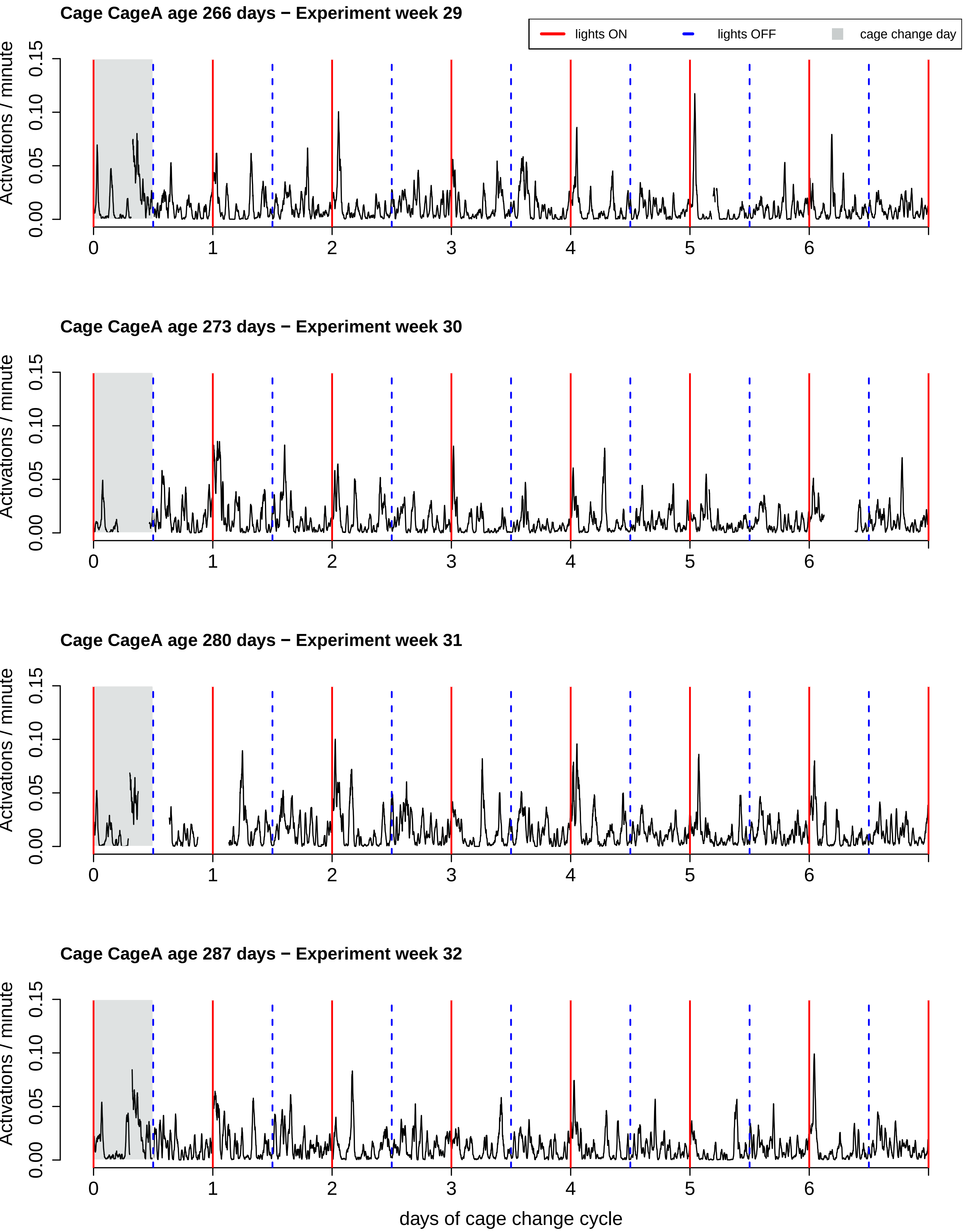

Cage CageA age 266 days − Experiment week 29
lights ON
lights OFF
cage change day
Activations / minute
0.00
0.05
0.10
0.15
0
1
2
3
4
5
6
Cage CageA age 273 days − Experiment week 30
Activations / minute
Cage CageA age 280 days − Experiment week 31
Activations / minute
Cage CageA age 287 days − Experiment week 32
Activations / minute
days of cage change cycle

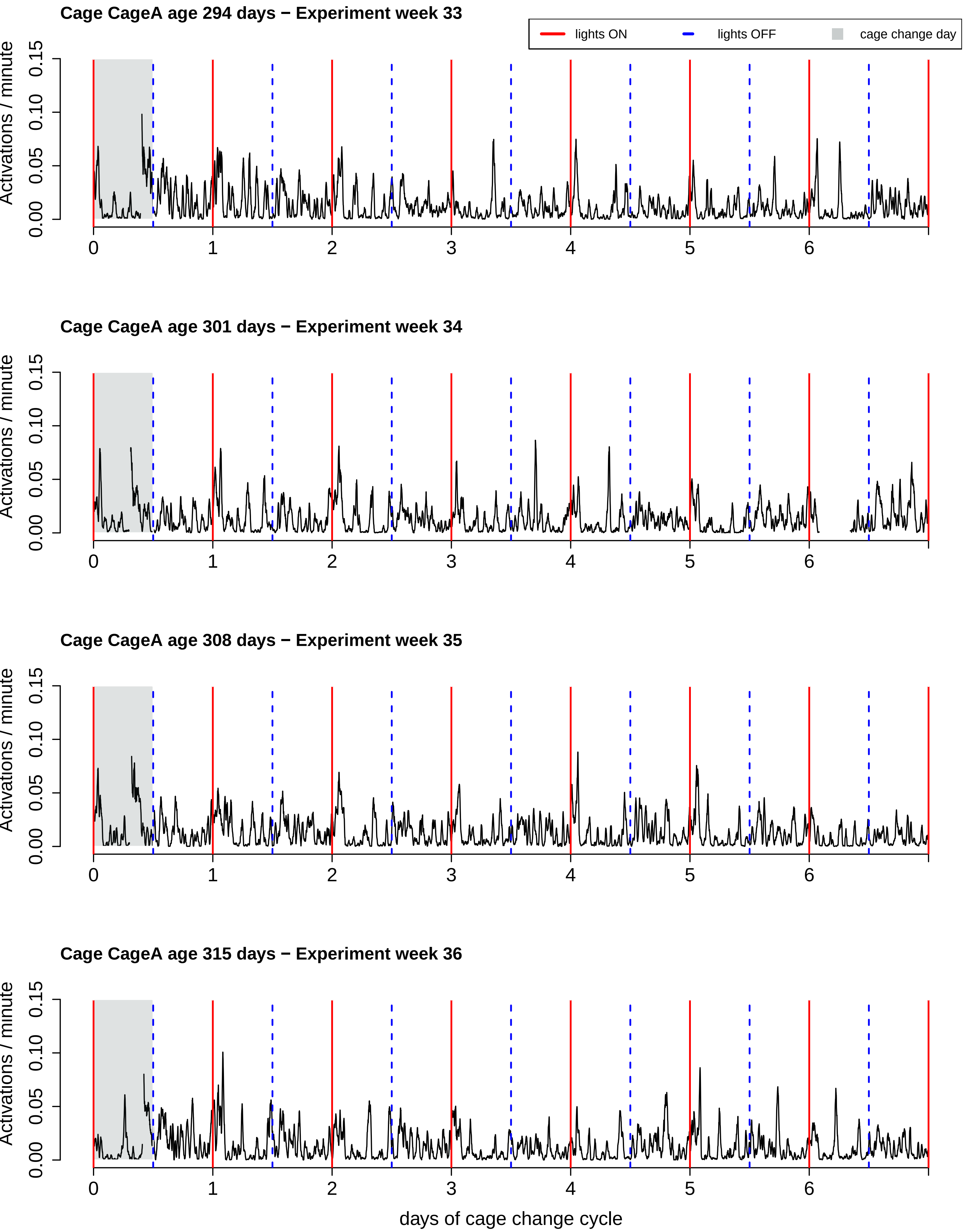
Cage CageA age 294 days − Experiment week 33
lights ON
lights OFF
cage change day
Activations / minute
0.00
0.05
0.10
0.15
0
1
2
3
4
5
6
Cage CageA age 301 days − Experiment week 34
Cage CageA age 308 days − Experiment week 35
Cage CageA age 315 days − Experiment week 36
days of cage change cycle

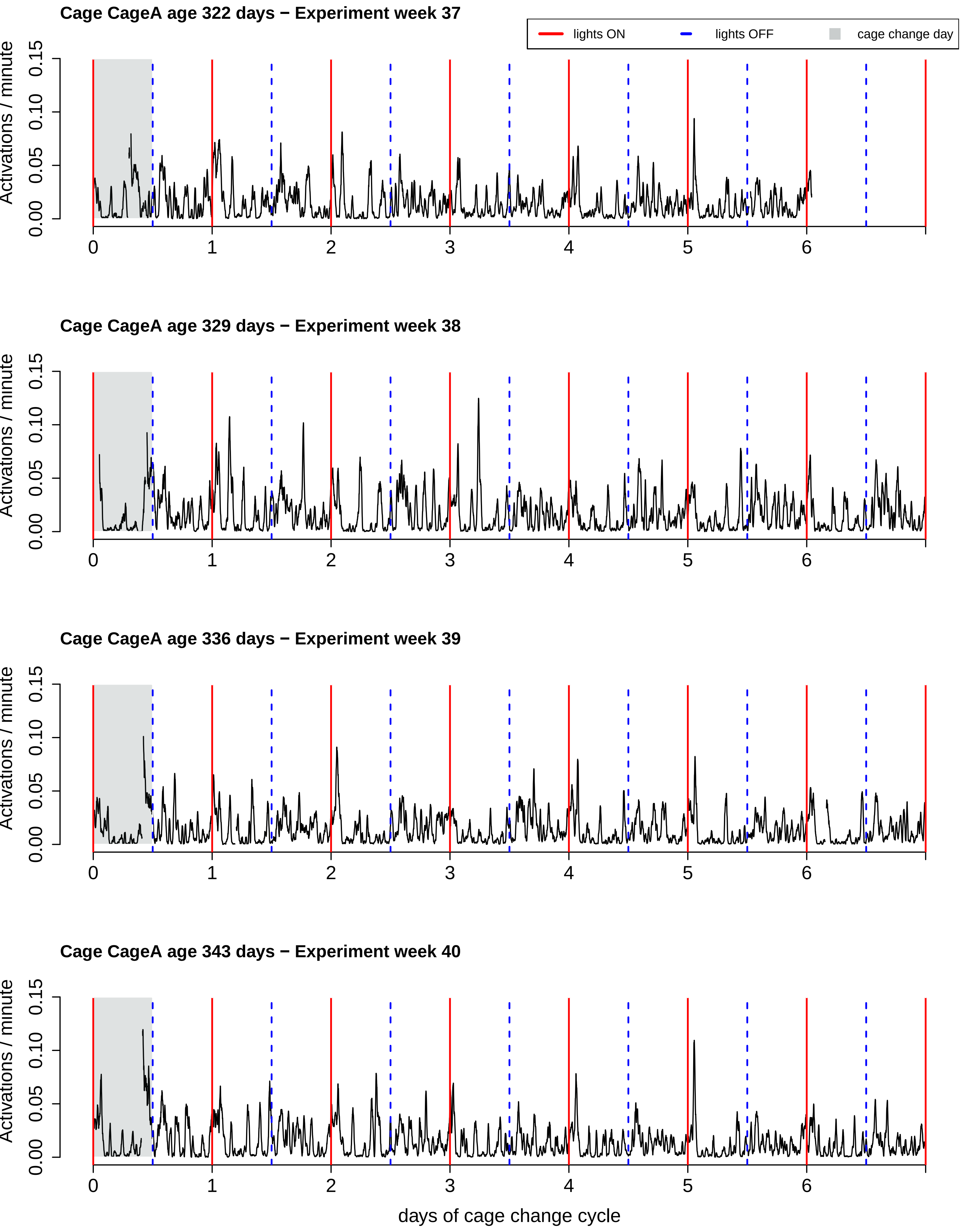

Cage CageA age 322 days − Experiment week 37
lights ON
lights OFF
cage change day
Cage CageA age 329 days − Experiment week 38
Cage CageA age 336 days − Experiment week 39
Cage CageA age 343 days − Experiment week 40
Activations / minute
0.00
0.05
0.10
0.15
0
1
2
3
4
5
6
days of cage change cycle

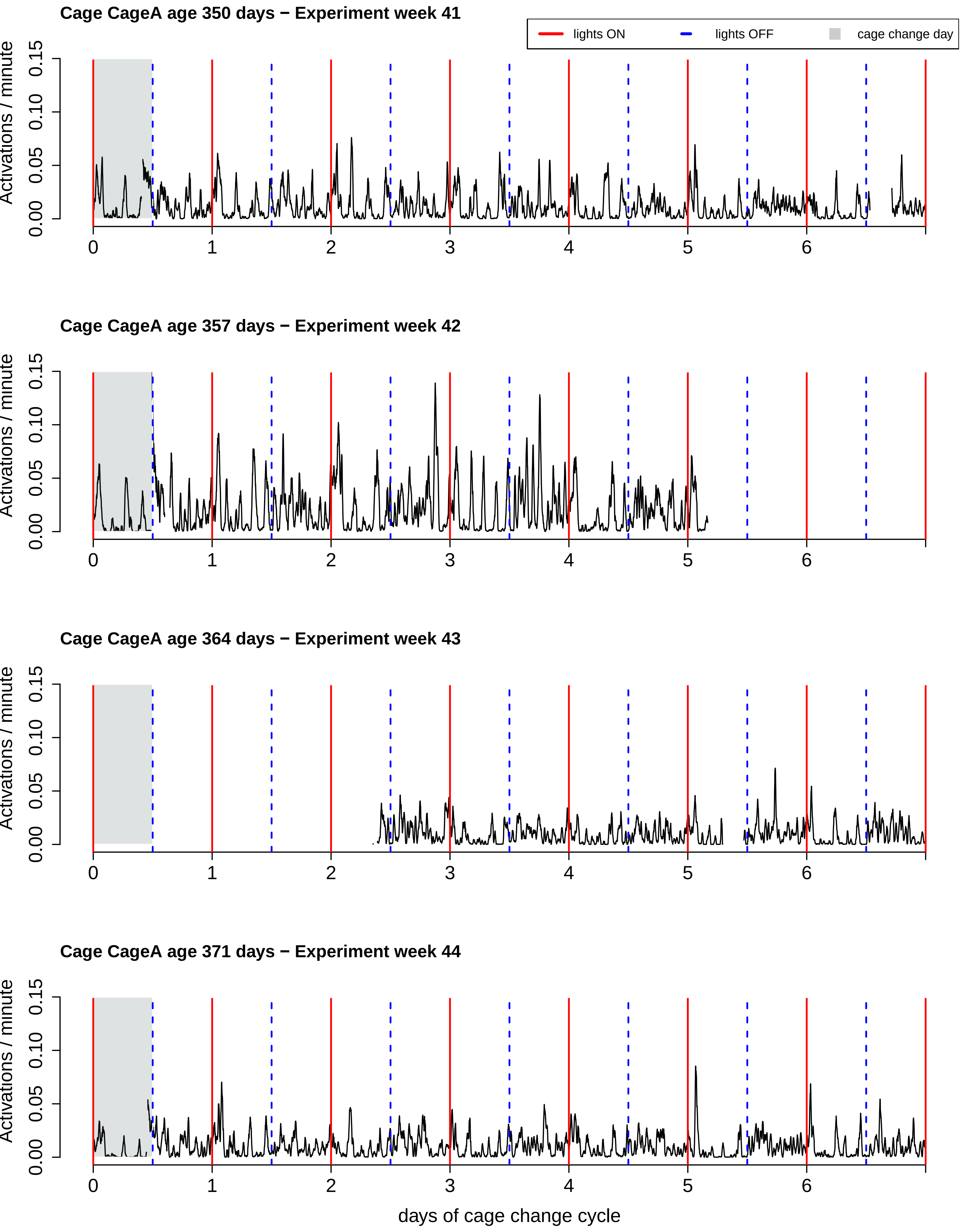

Cage CageA age 350 days − Experiment week 41
lights ON
lights OFF
cage change day
Cage CageA age 357 days − Experiment week 42
Cage CageA age 364 days − Experiment week 43
Cage CageA age 371 days − Experiment week 44
Activations / minute
0.00
0.05
0.10
0.15
0
1
2
3
4
5
6
days of cage change cycle

Cage CageA age 378 days − Experiment week 45

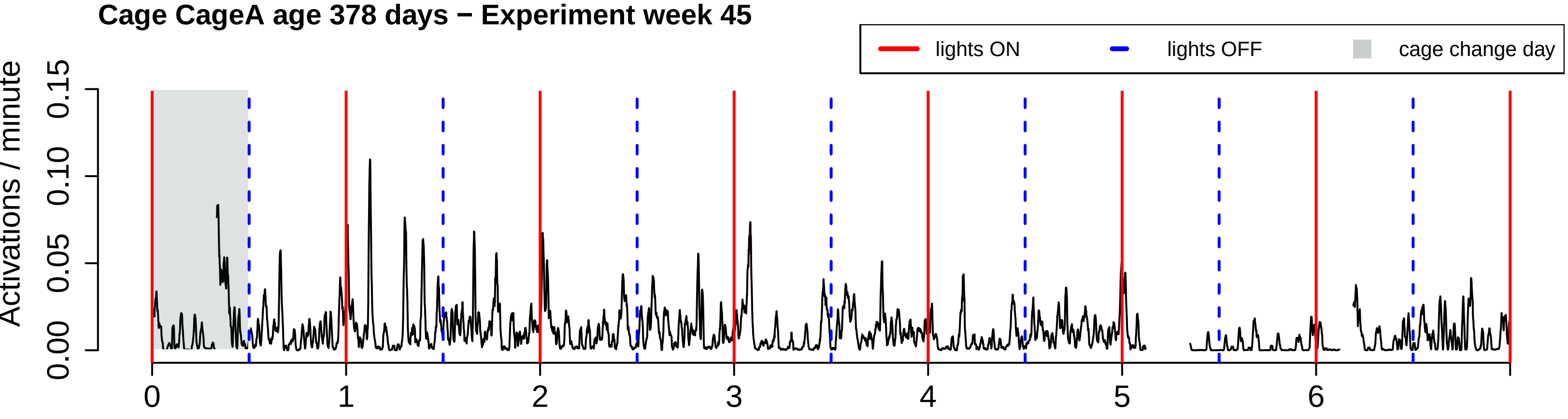

Cage CageA age 385 days − Experiment week 46

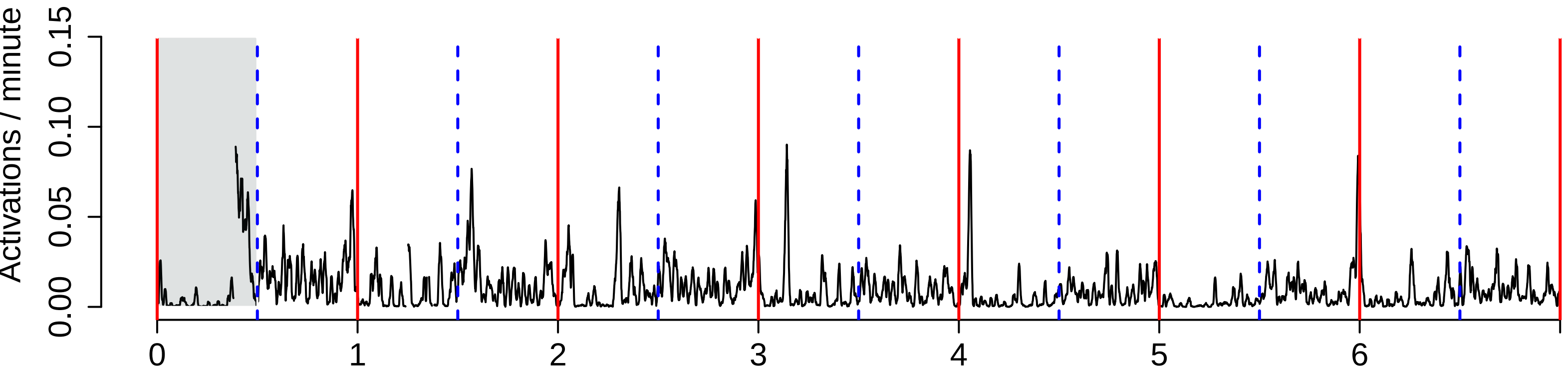

Cage CageA age 392 days − Experiment week 47

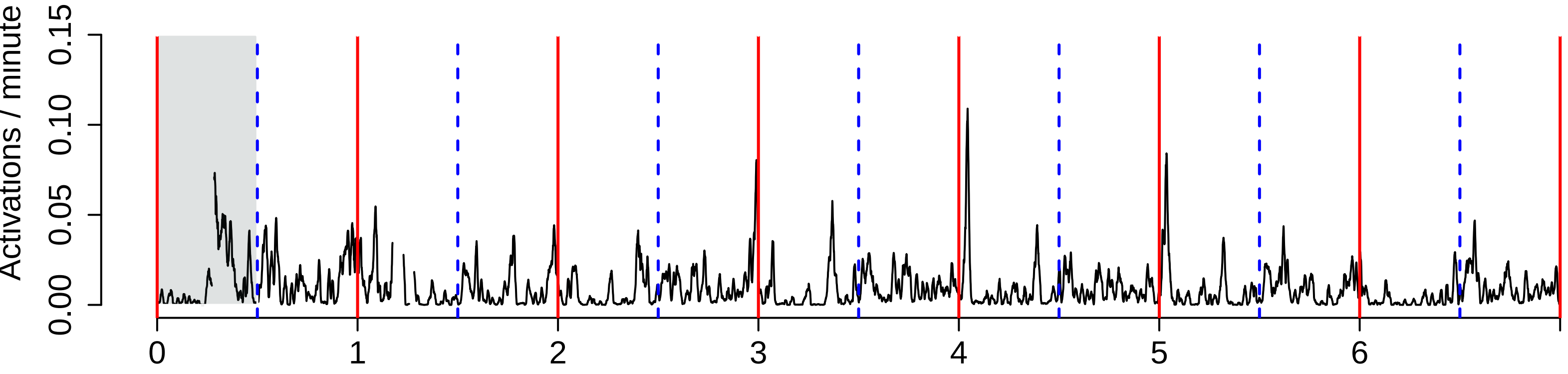

Cage CageA age 399 days − Experiment week 48

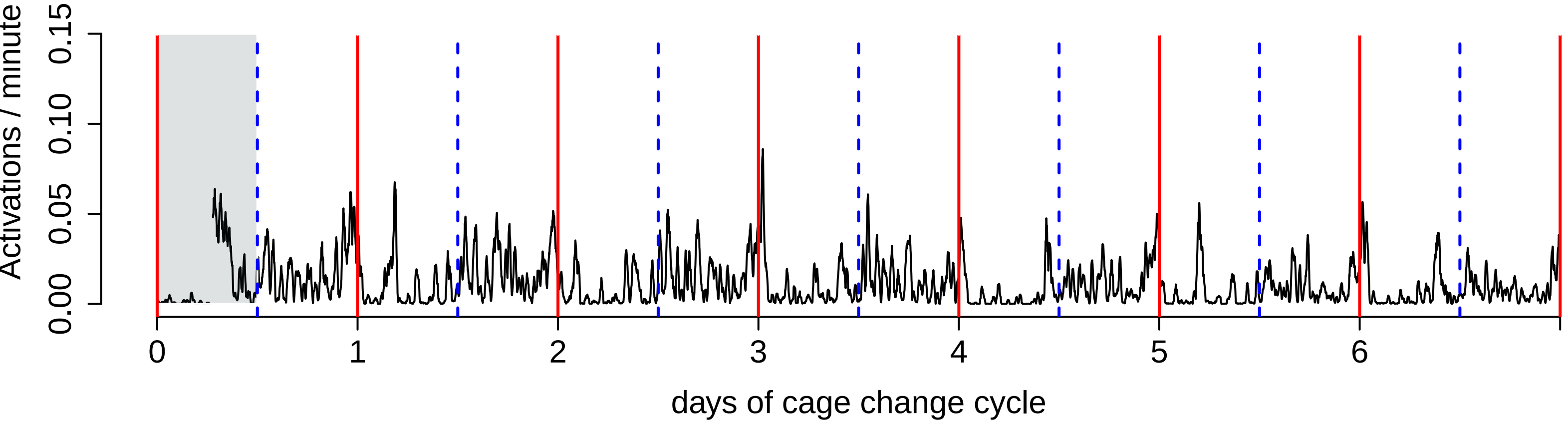

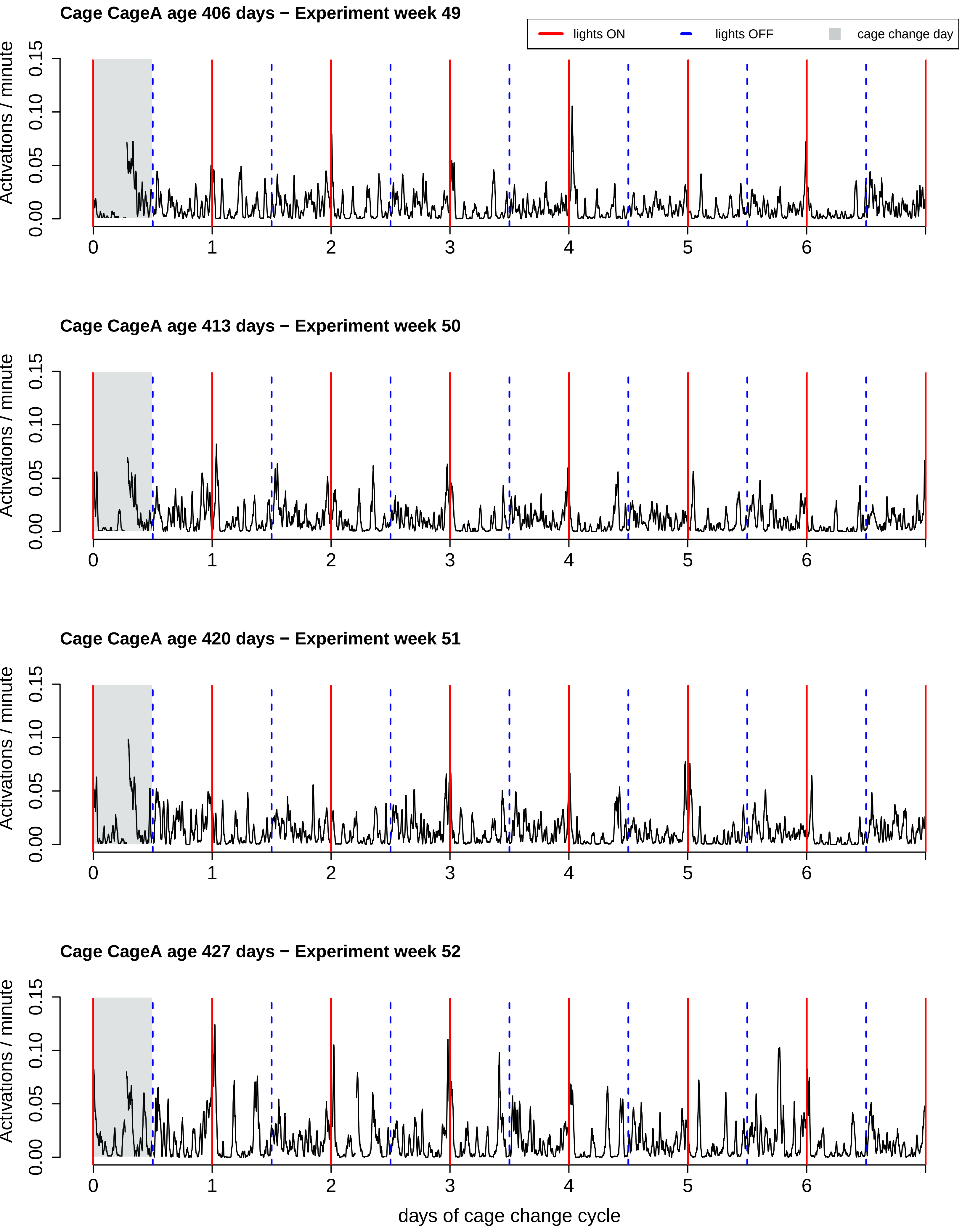

Cage CageA age 406 days − Experiment week 49
lights ON
lights OFF
cage change day
Activations / minute
0.00
0.05
0.10
0.15
0
1
2
3
4
5
6
Cage CageA age 413 days − Experiment week 50
Cage CageA age 420 days − Experiment week 51
Cage CageA age 427 days − Experiment week 52
days of cage change cycle

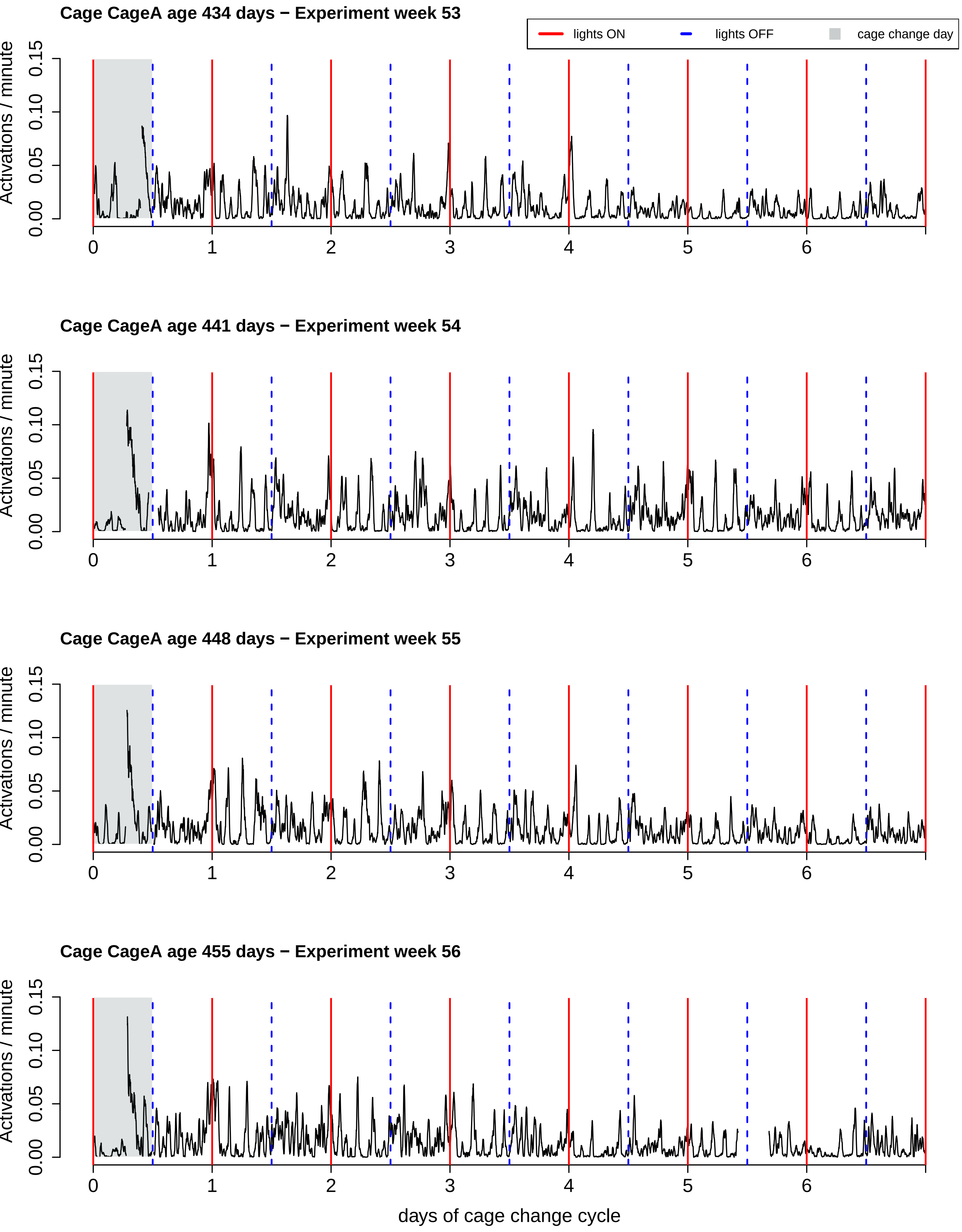

Cage CageA age 434 days − Experiment week 53
lights ON
lights OFF
cage change day
Activations / minute
0.00
0.05
0.10
0.15
0
1
2
3
4
5
6
Cage CageA age 441 days − Experiment week 54
Cage CageA age 448 days − Experiment week 55
Cage CageA age 455 days − Experiment week 56
days of cage change cycle

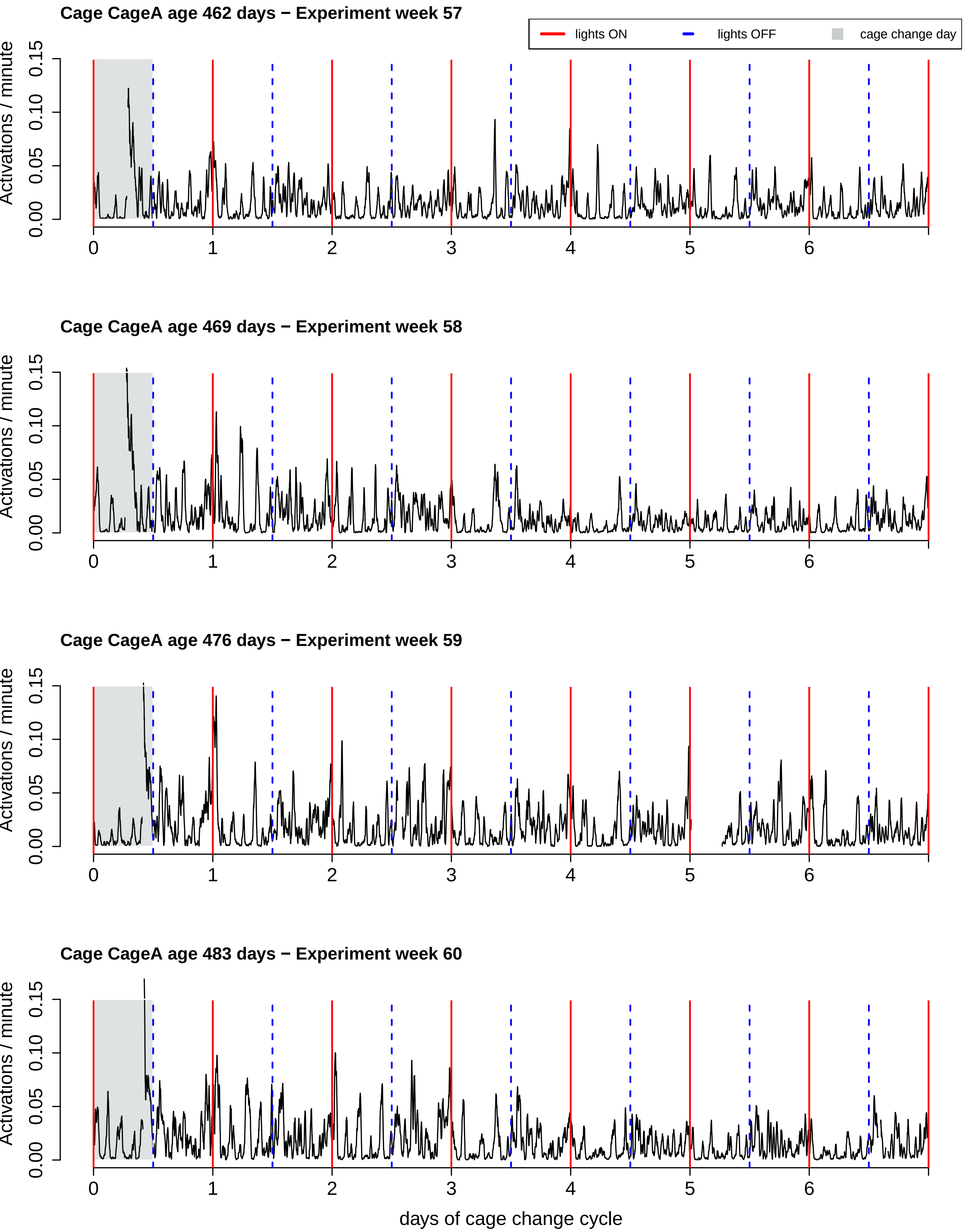
Cage CageA age 462 days − Experiment week 57
lights ON
lights OFF
cage change day
Activations / minute
0.00
0.05
0.10
0.15
0
1
2
3
4
5
6
Cage CageA age 469 days − Experiment week 58
Cage CageA age 476 days − Experiment week 59
Cage CageA age 483 days − Experiment week 60
days of cage change cycle

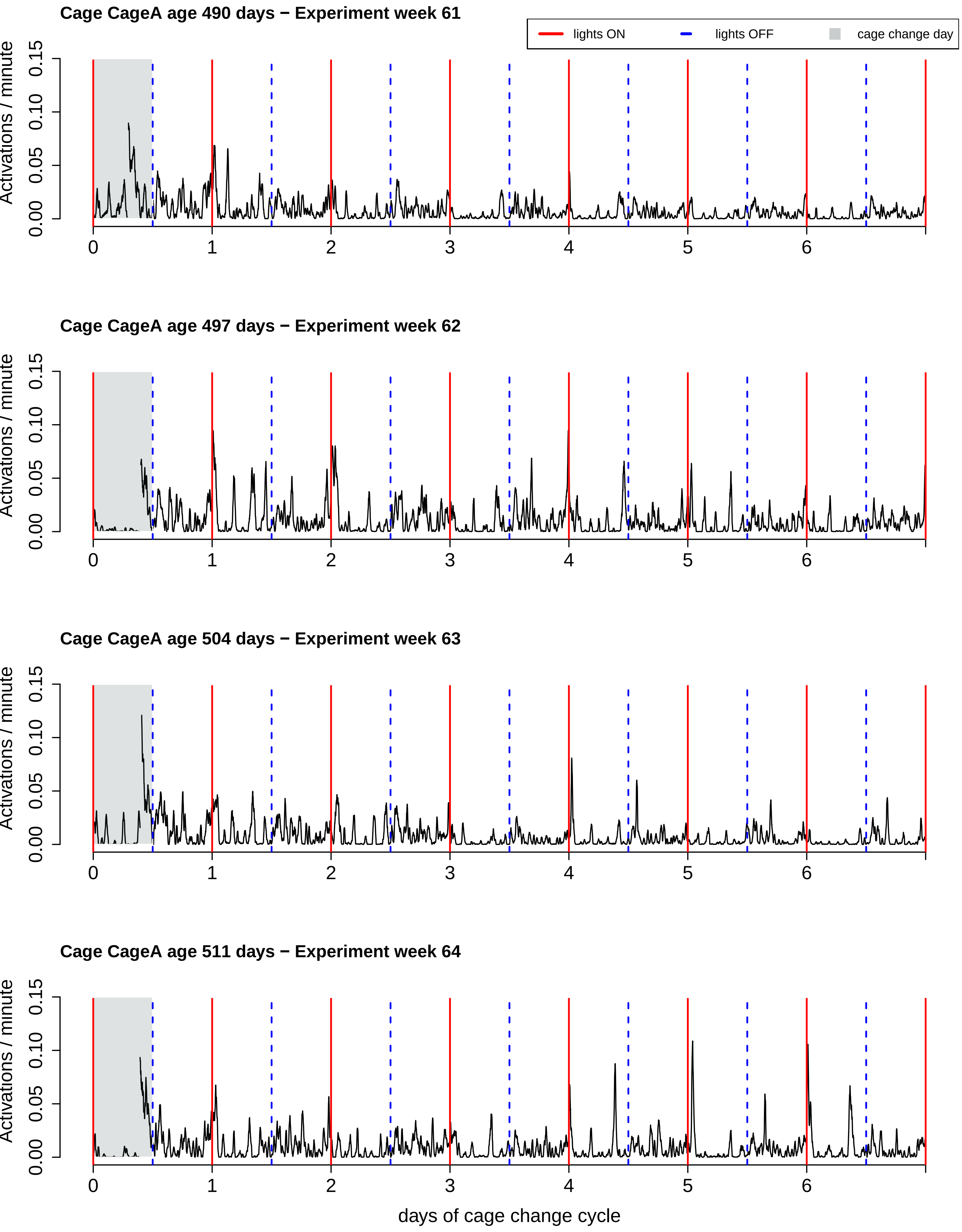

Cage CageA age 490 days − Experiment week 61
lights ON
lights OFF
cage change day
Activations / minute
0.00
0.05
0.10
0.15
0
1
2
3
4
5
6
Cage CageA age 497 days − Experiment week 62
Cage CageA age 504 days − Experiment week 63
Cage CageA age 511 days − Experiment week 64
days of cage change cycle

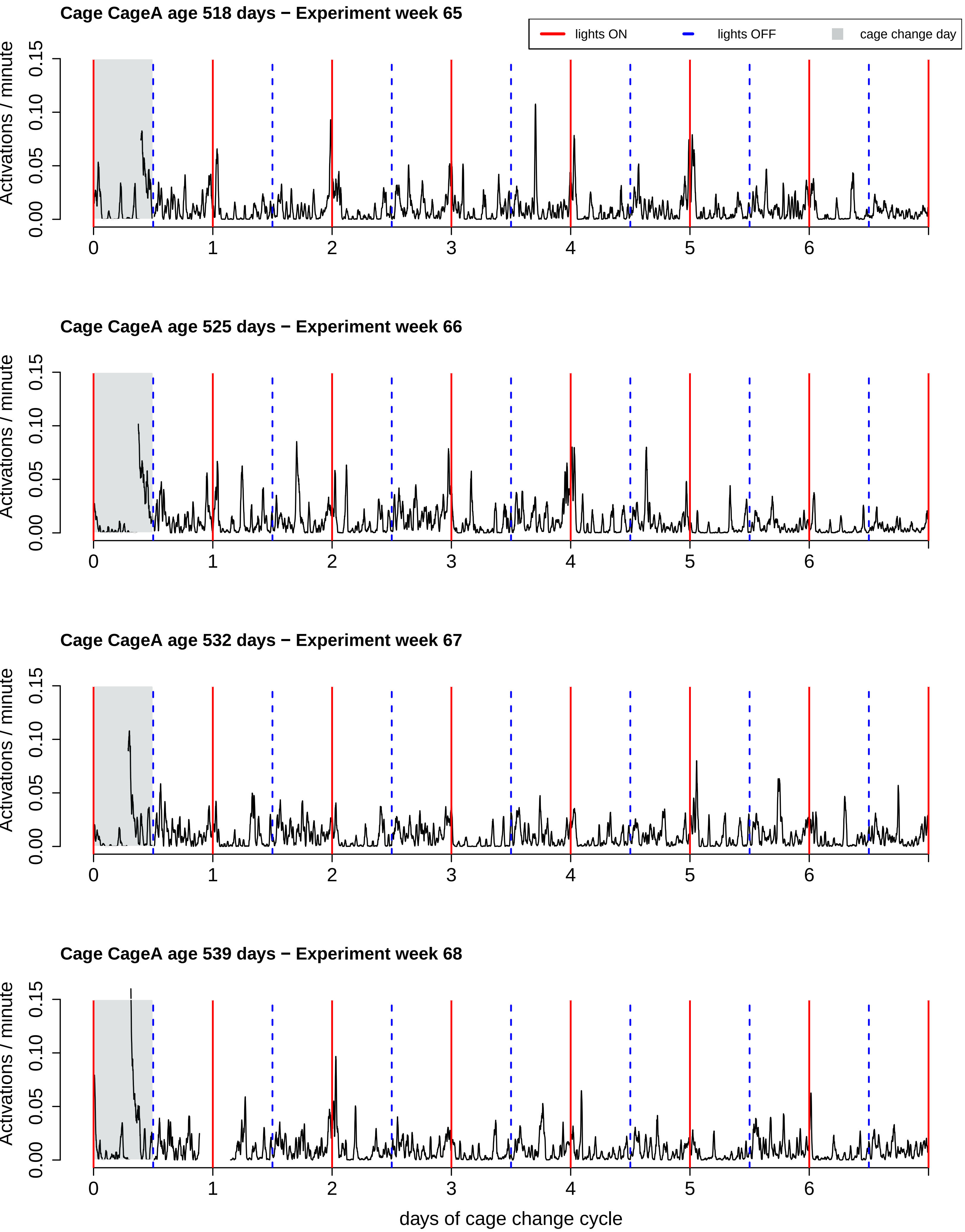

Cage CageA age 518 days − Experiment week 65
lights ON
lights OFF
cage change day
Activations / minute
0.00
0.05
0.10
0.15
0
1
2
3
4
5
6
Cage CageA age 525 days − Experiment week 66
Cage CageA age 532 days − Experiment week 67
Cage CageA age 539 days − Experiment week 68
days of cage change cycle

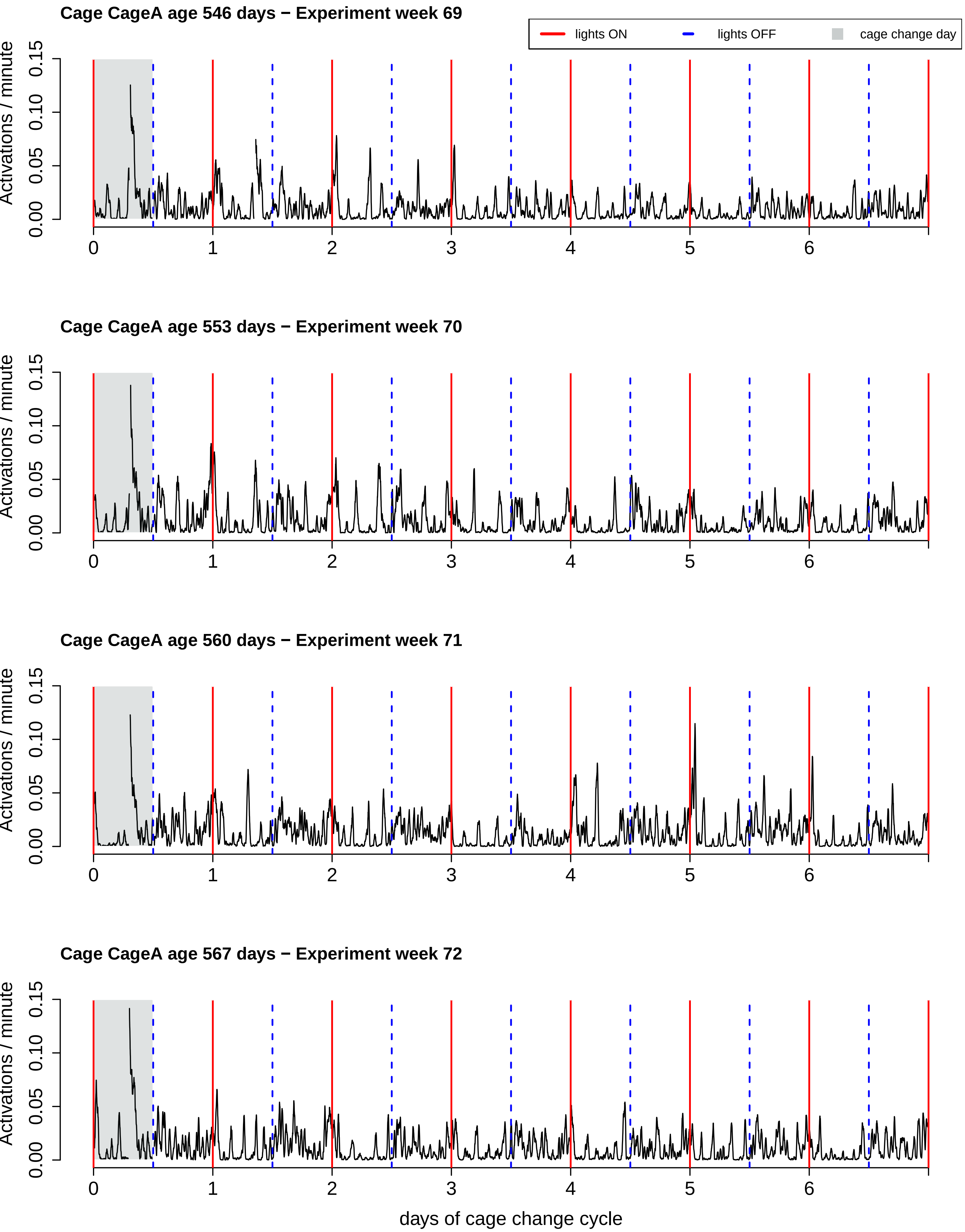

Cage CageA age 546 days − Experiment week 69
lights ON
lights OFF
cage change day
Activations / minute
Cage CageA age 553 days − Experiment week 70
Activations / minute
Cage CageA age 560 days − Experiment week 71
Activations / minute
Cage CageA age 567 days − Experiment week 72
Activations / minute
days of cage change cycle

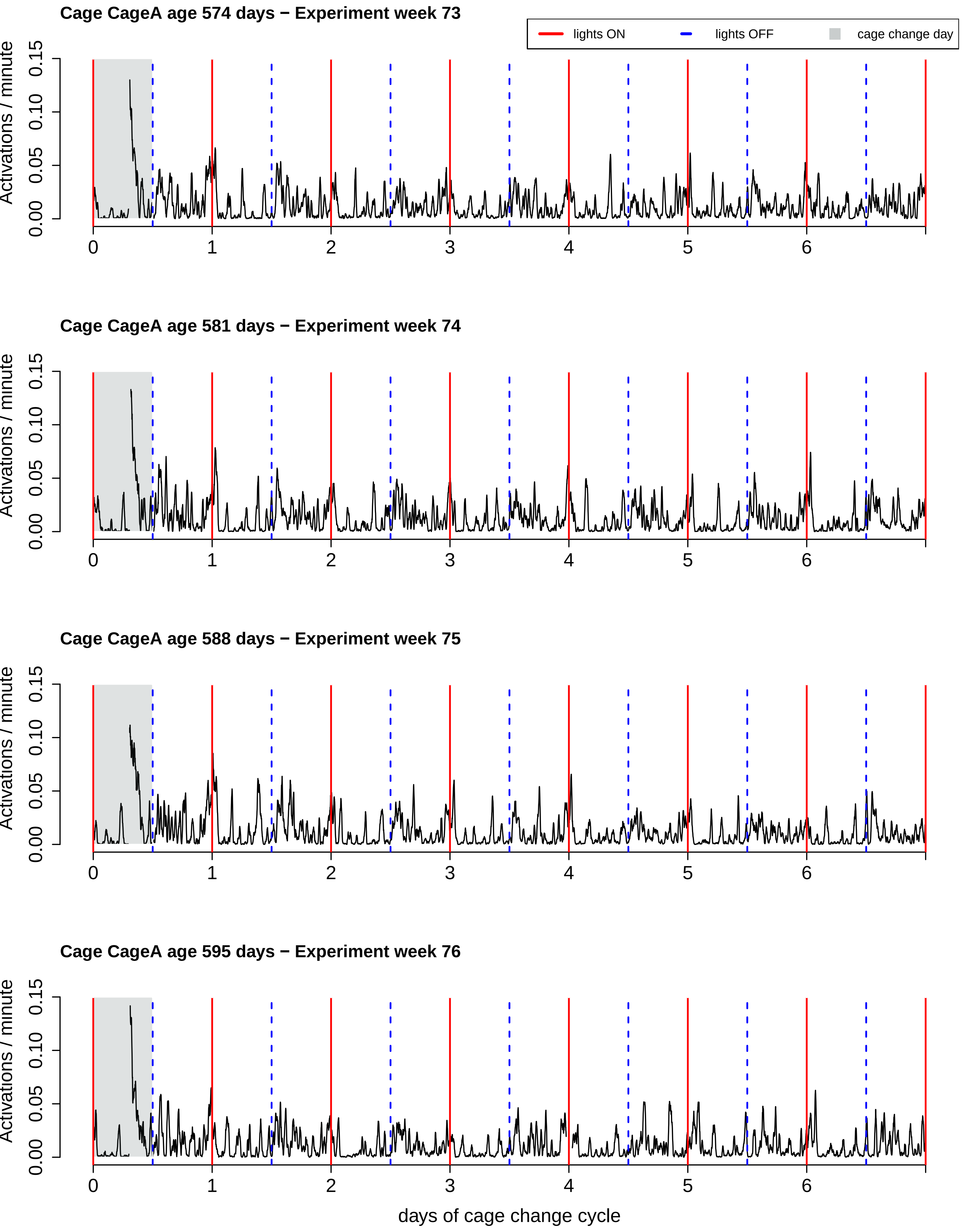
Cage CageA age 574 days − Experiment week 73
lights ON
lights OFF
cage change day
Activations / minute
0.00
0.05
0.10
0.15
0
1
2
3
4
5
6
Cage CageA age 581 days − Experiment week 74
Cage CageA age 588 days − Experiment week 75
Cage CageA age 595 days − Experiment week 76
days of cage change cycle

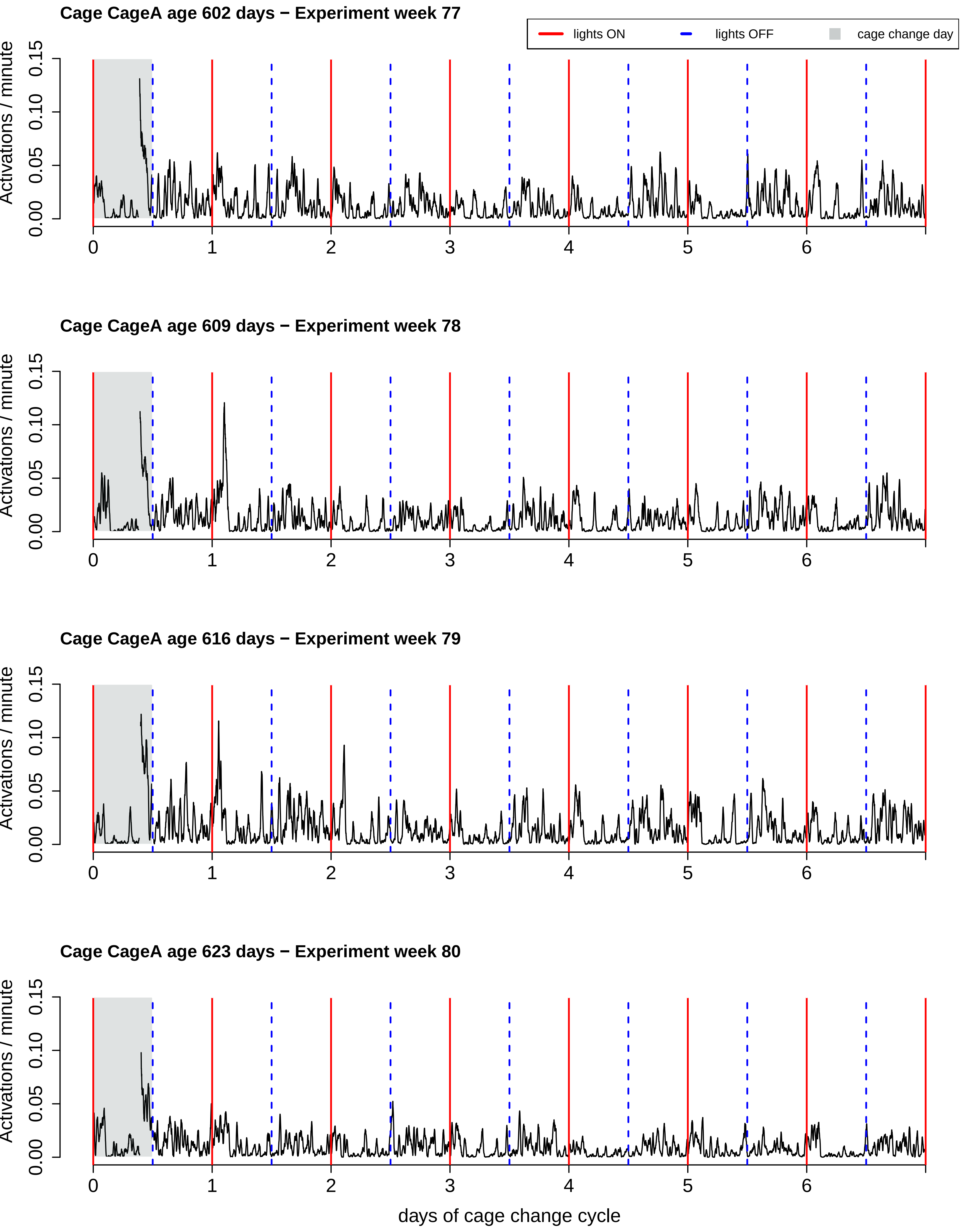
Cage CageA age 602 days − Experiment week 77
lights ON
lights OFF
cage change day
Activations / minute
0.00 0.05 0.10 0.15
0 1 2 3 4 5 6
Cage CageA age 609 days − Experiment week 78
Activations / minute
0.00 0.05 0.10 0.15
0 1 2 3 4 5 6
Cage CageA age 616 days − Experiment week 79
Activations / minute
0.00 0.05 0.10 0.15
0 1 2 3 4 5 6
Cage CageA age 623 days − Experiment week 80
Activations / minute
0.00 0.05 0.10 0.15
0 1 2 3 4 5 6
days of cage change cycle

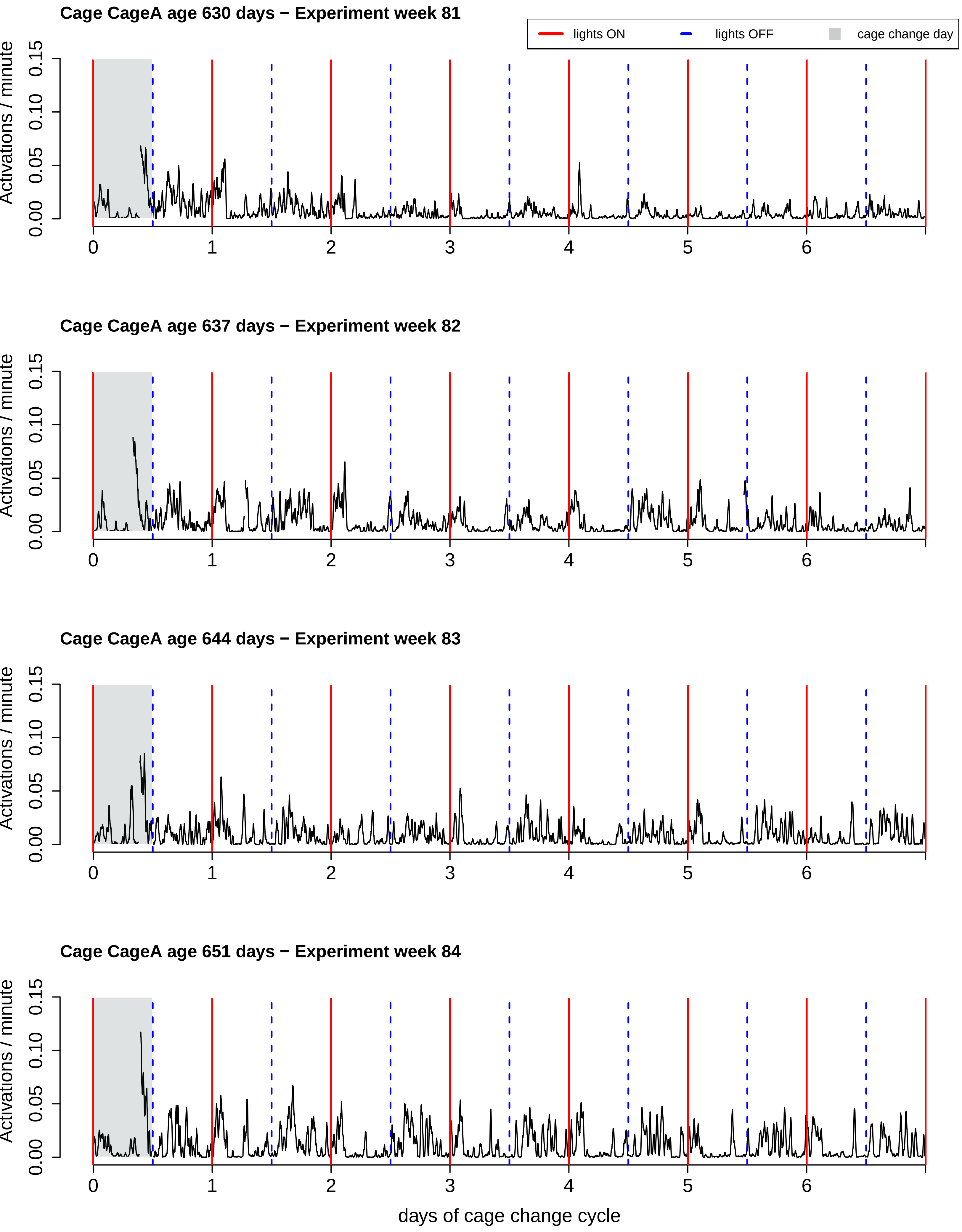
Cage CageA age 630 days − Experiment week 81
lights ON
lights OFF
cage change day
Activations / minute
0.00
0.05
0.10
0.15
0
1
2
3
4
5
6
Cage CageA age 637 days − Experiment week 82
Cage CageA age 644 days − Experiment week 83
Cage CageA age 651 days − Experiment week 84
days of cage change cycle

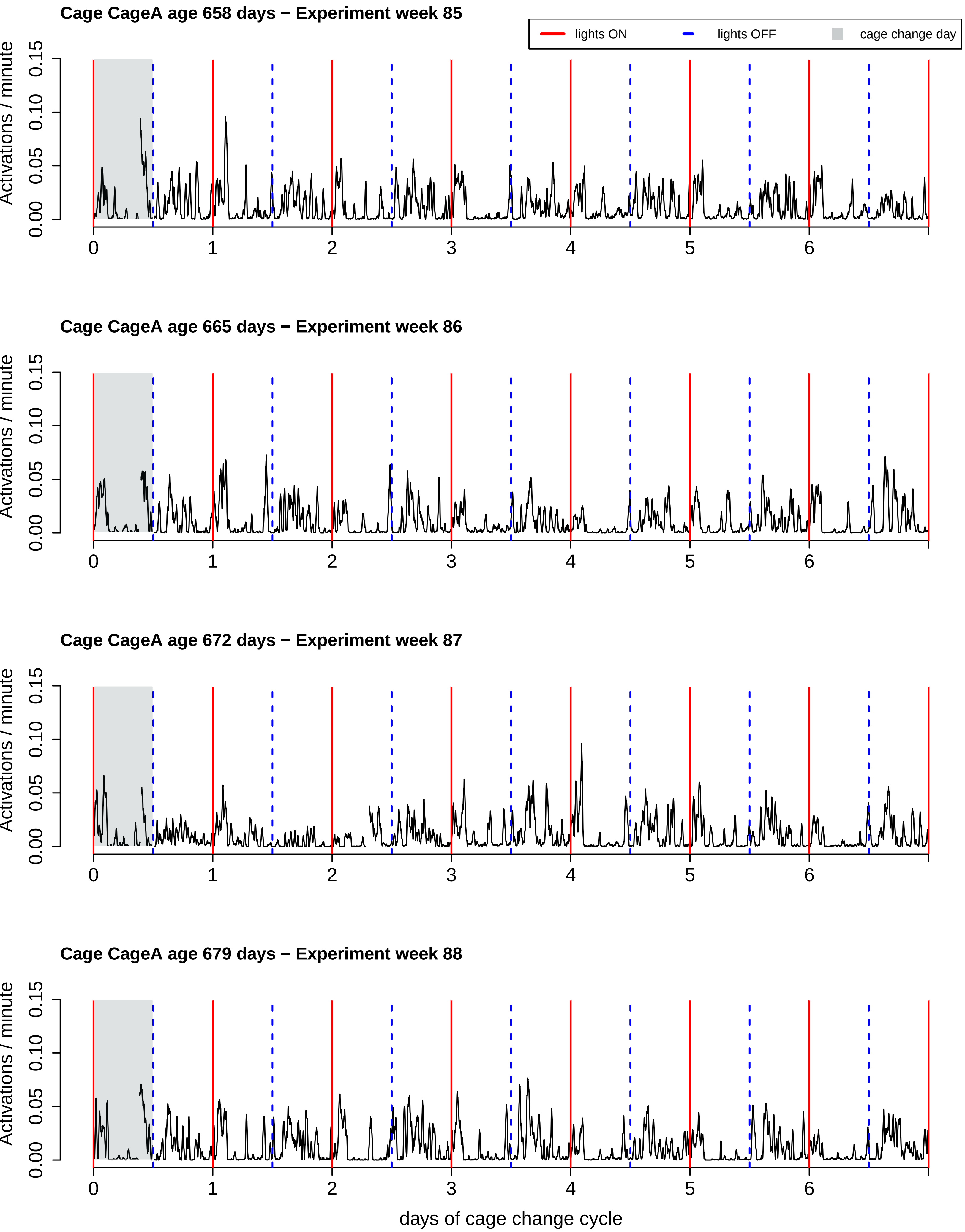
Cage CageA age 658 days − Experiment week 85
lights ON
lights OFF
cage change day
Activations / minute
0.00
0.05
0.10
0.15
0
1
2
3
4
5
6
Cage CageA age 665 days − Experiment week 86
Cage CageA age 672 days − Experiment week 87
Cage CageA age 679 days − Experiment week 88
days of cage change cycle

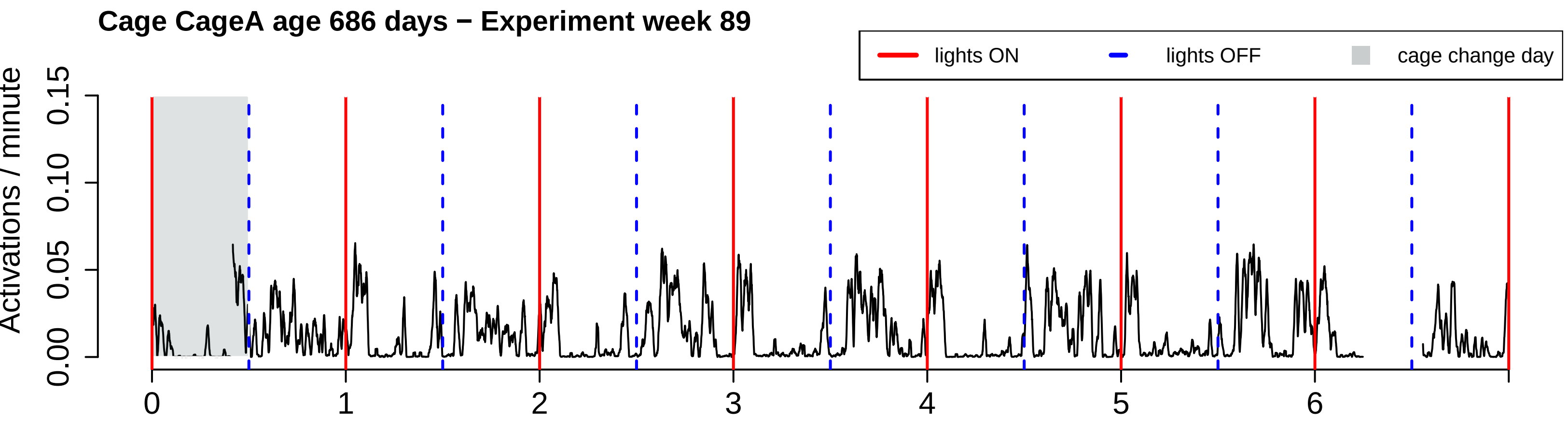
Cage CageA age 686 days − Experiment week 89
lights ON
lights OFF
cage change day
Activations / minute
0.00
0.05
0.10
0.15
0
1
2
3
4
5
6

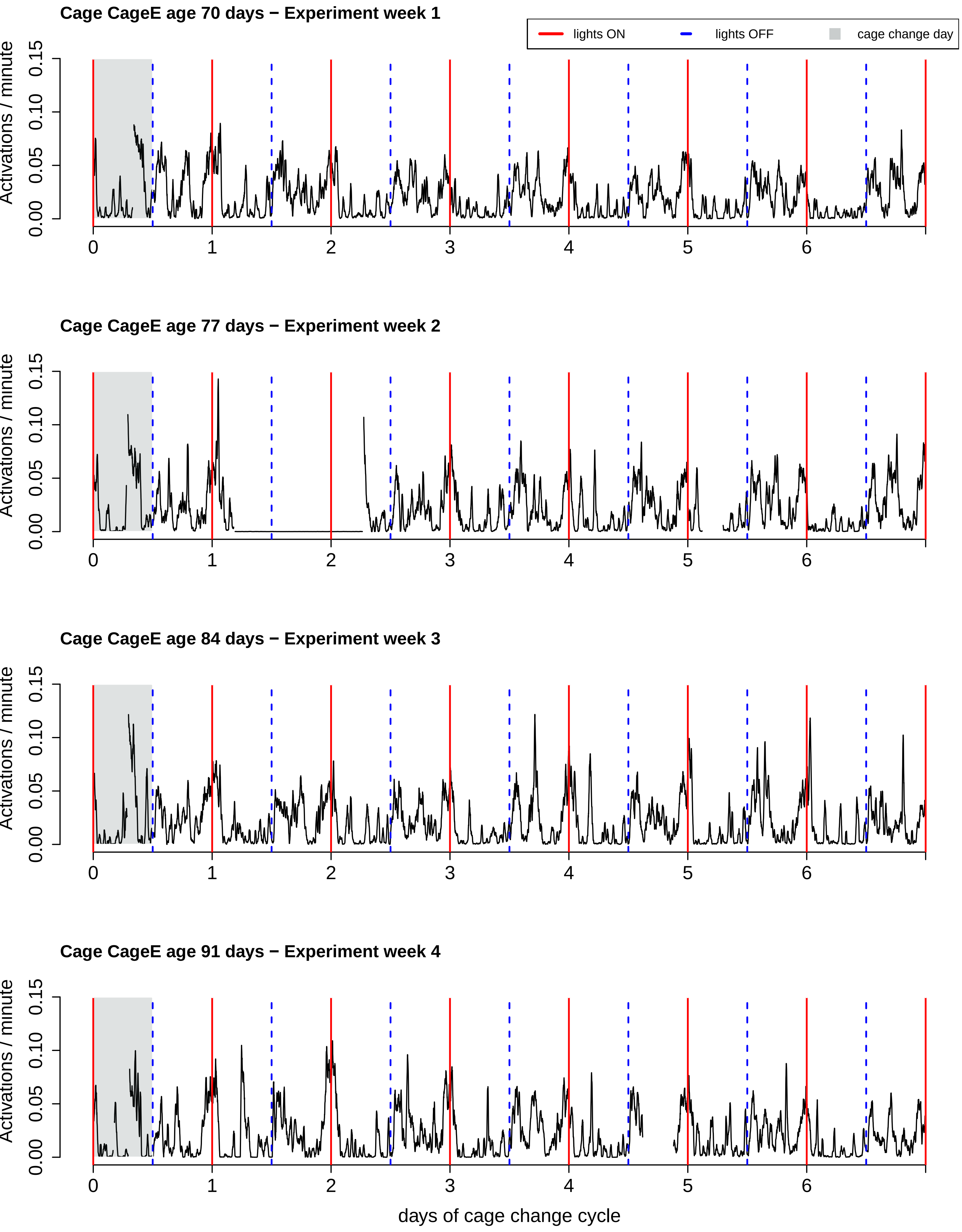
Cage CageE age 70 days − Experiment week 1
lights ON
lights OFF
cage change day
Activations / minute
0.00
0.05
0.10
0.15
0
1
2
3
4
5
6
Cage CageE age 77 days − Experiment week 2
Cage CageE age 84 days − Experiment week 3
Cage CageE age 91 days − Experiment week 4
days of cage change cycle

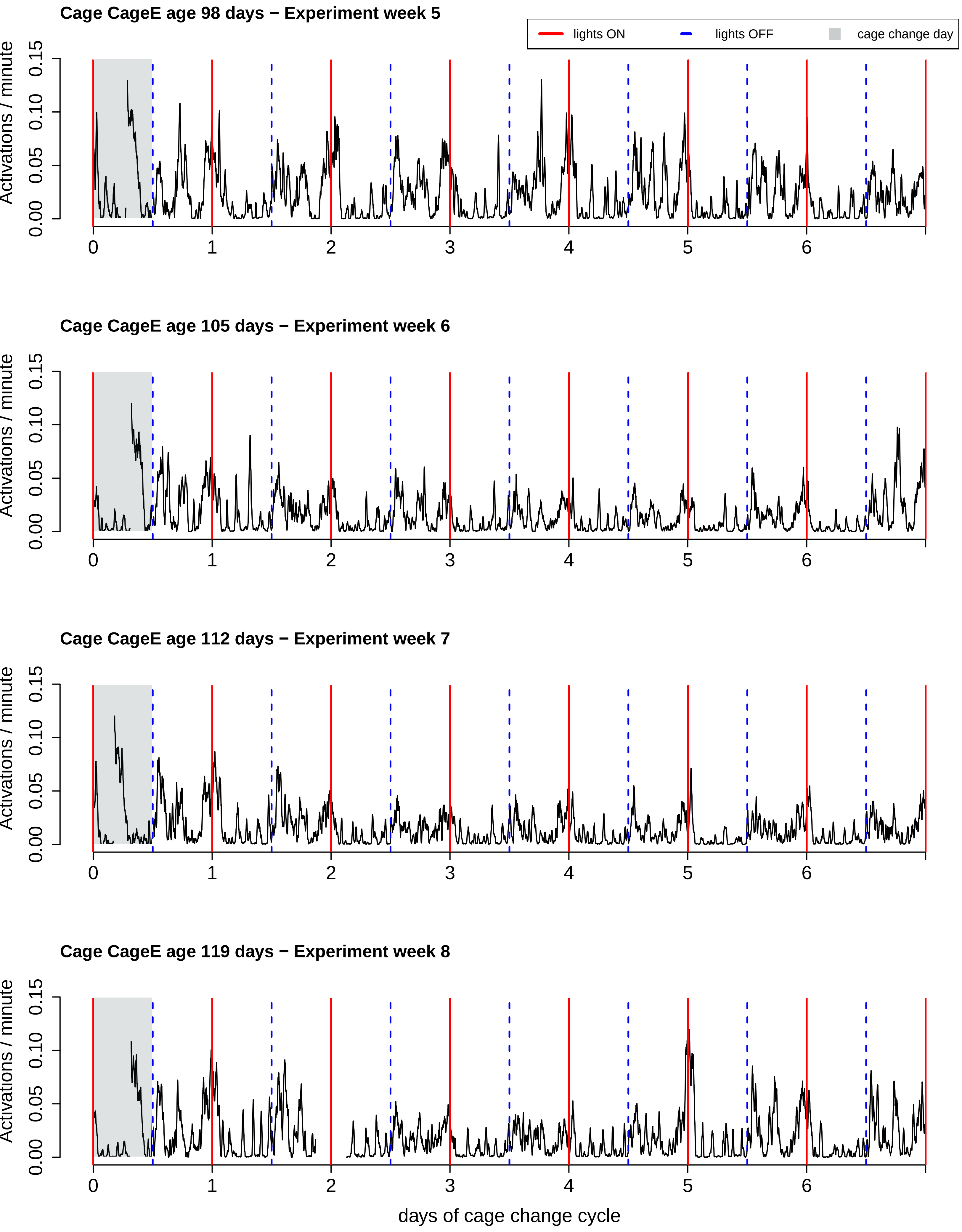
Cage CageE age 98 days − Experiment week 5
lights ON
lights OFF
cage change day
Activations / minute
0.00
0.05
0.10
0.15
0
1
2
3
4
5
6
Cage CageE age 105 days − Experiment week 6
Activations / minute
Cage CageE age 112 days − Experiment week 7
Activations / minute
Cage CageE age 119 days − Experiment week 8
Activations / minute
days of cage change cycle

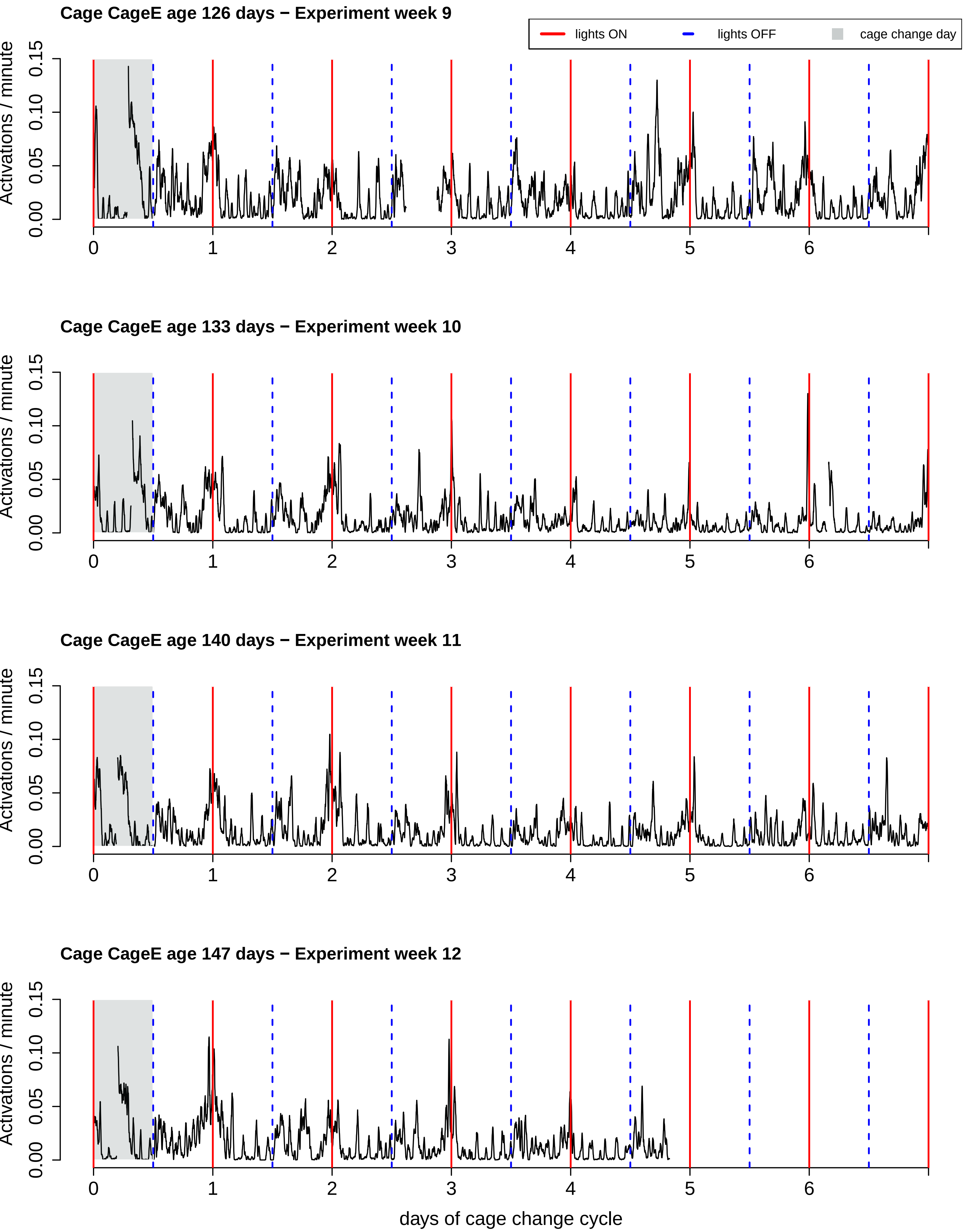
Cage CageE age 126 days − Experiment week 9
lights ON
lights OFF
cage change day
Activations / minute
0.00
0.05
0.10
0.15
0
1
2
3
4
5
6
Cage CageE age 133 days − Experiment week 10
Activations / minute
0.00
0.05
0.10
0.15
0
1
2
3
4
5
6
Cage CageE age 140 days − Experiment week 11
Activations / minute
0.00
0.05
0.10
0.15
0
1
2
3
4
5
6
Cage CageE age 147 days − Experiment week 12
Activations / minute
0.00
0.05
0.10
0.15
0
1
2
3
4
5
6
days of cage change cycle

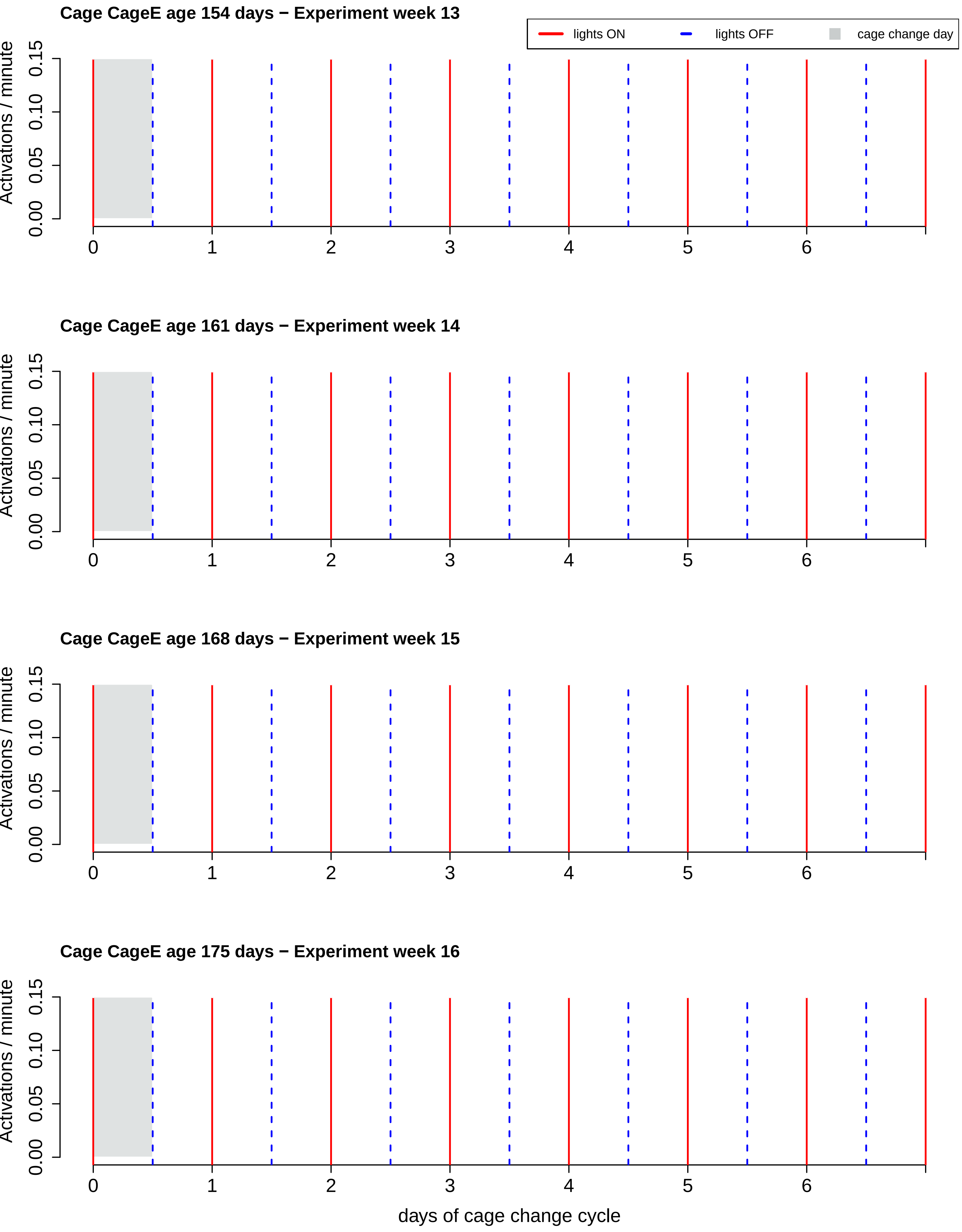
Cage CageE age 154 days − Experiment week 13
lights ON
lights OFF
cage change day
Activations / minute
0.00
0.05
0.10
0.15
0 1 2 3 4 5 6
Cage CageE age 161 days − Experiment week 14
Activations / minute
0.00
0.05
0.10
0.15
0 1 2 3 4 5 6
Cage CageE age 168 days − Experiment week 15
Activations / minute
0.00
0.05
0.10
0.15
0 1 2 3 4 5 6
Cage CageE age 175 days − Experiment week 16
Activations / minute
0.00
0.05
0.10
0.15
0 1 2 3 4 5 6
days of cage change cycle

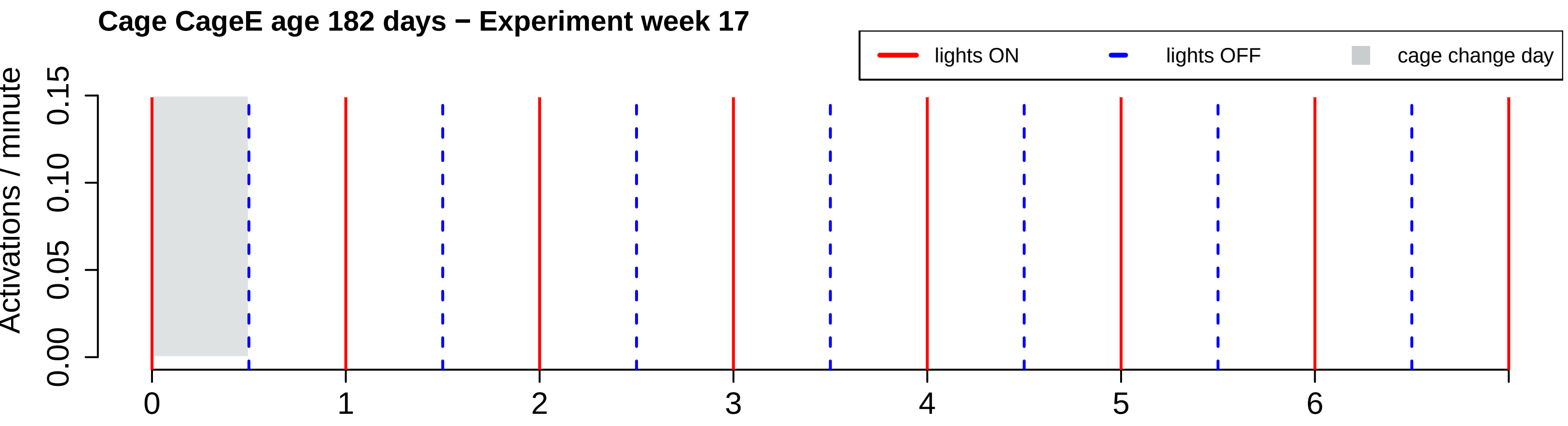
Cage CageE age 182 days − Experiment week 17
lights ON
lights OFF
cage change day
Activations / minute
0.00
0.05
0.10
0.15
0
1
2
3
4
5
6

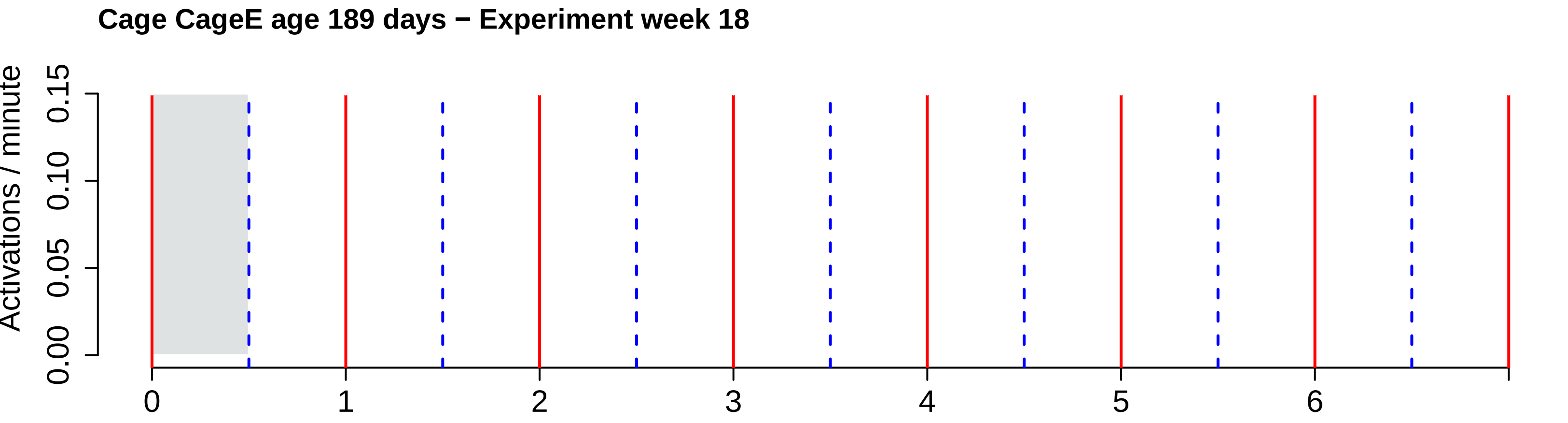
Cage CageE age 189 days − Experiment week 18
Activations / minute
0.00
0.05
0.10
0.15
0
1
2
3
4
5
6

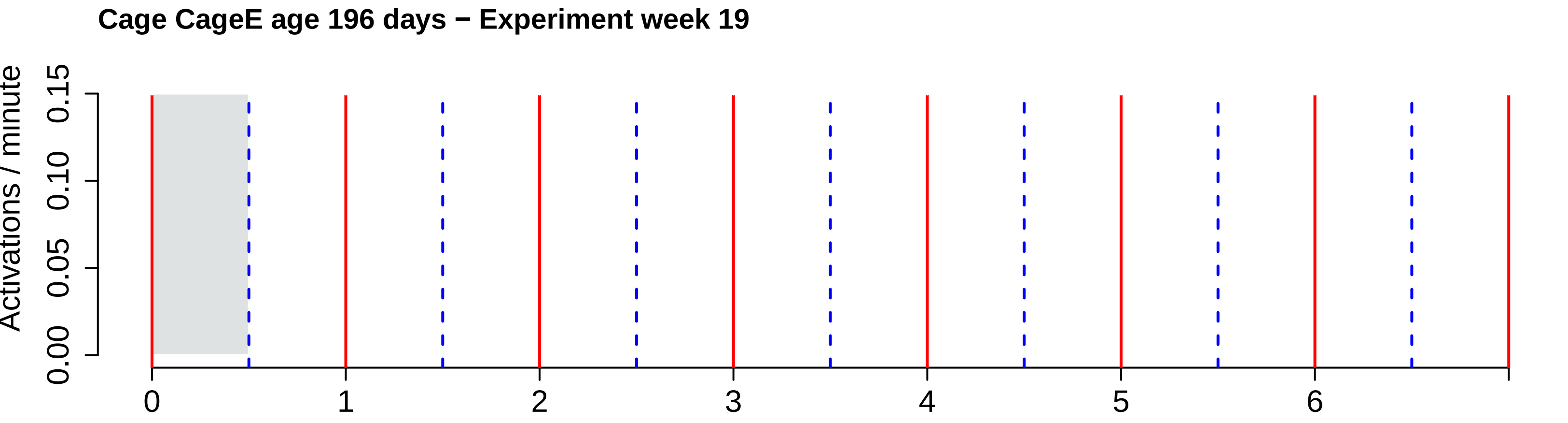
Cage CageE age 196 days − Experiment week 19
Activations / minute
0.00
0.05
0.10
0.15
0
1
2
3
4
5
6

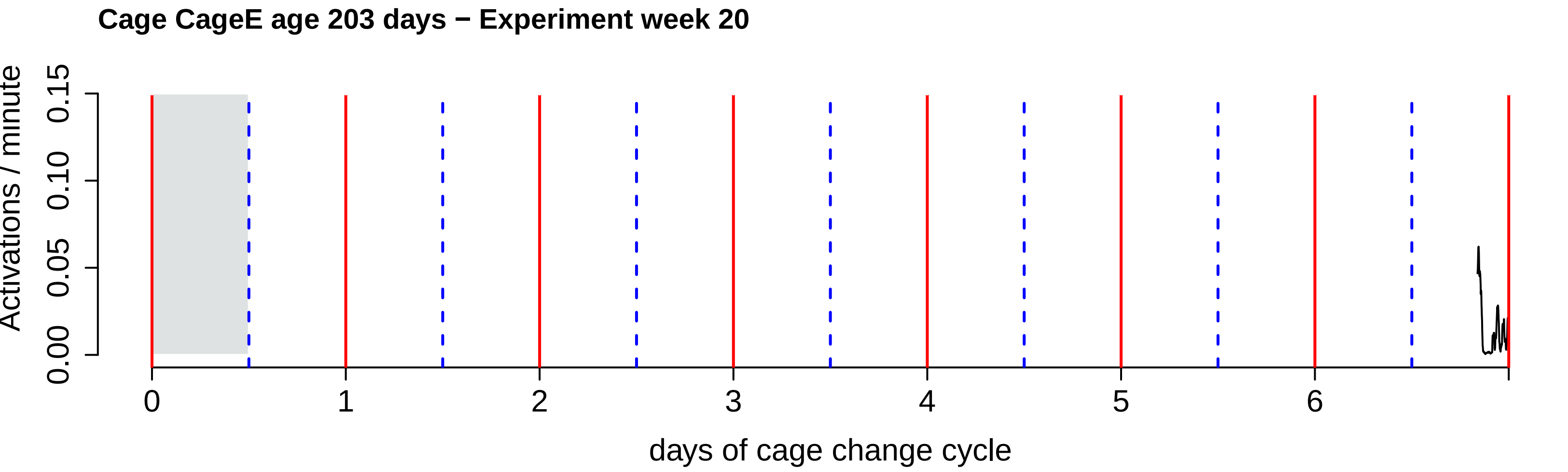
Cage CageE age 203 days − Experiment week 20
Activations / minute
0.00
0.05
0.10
0.15
0
1
2
3
4
5
6
days of cage change cycle

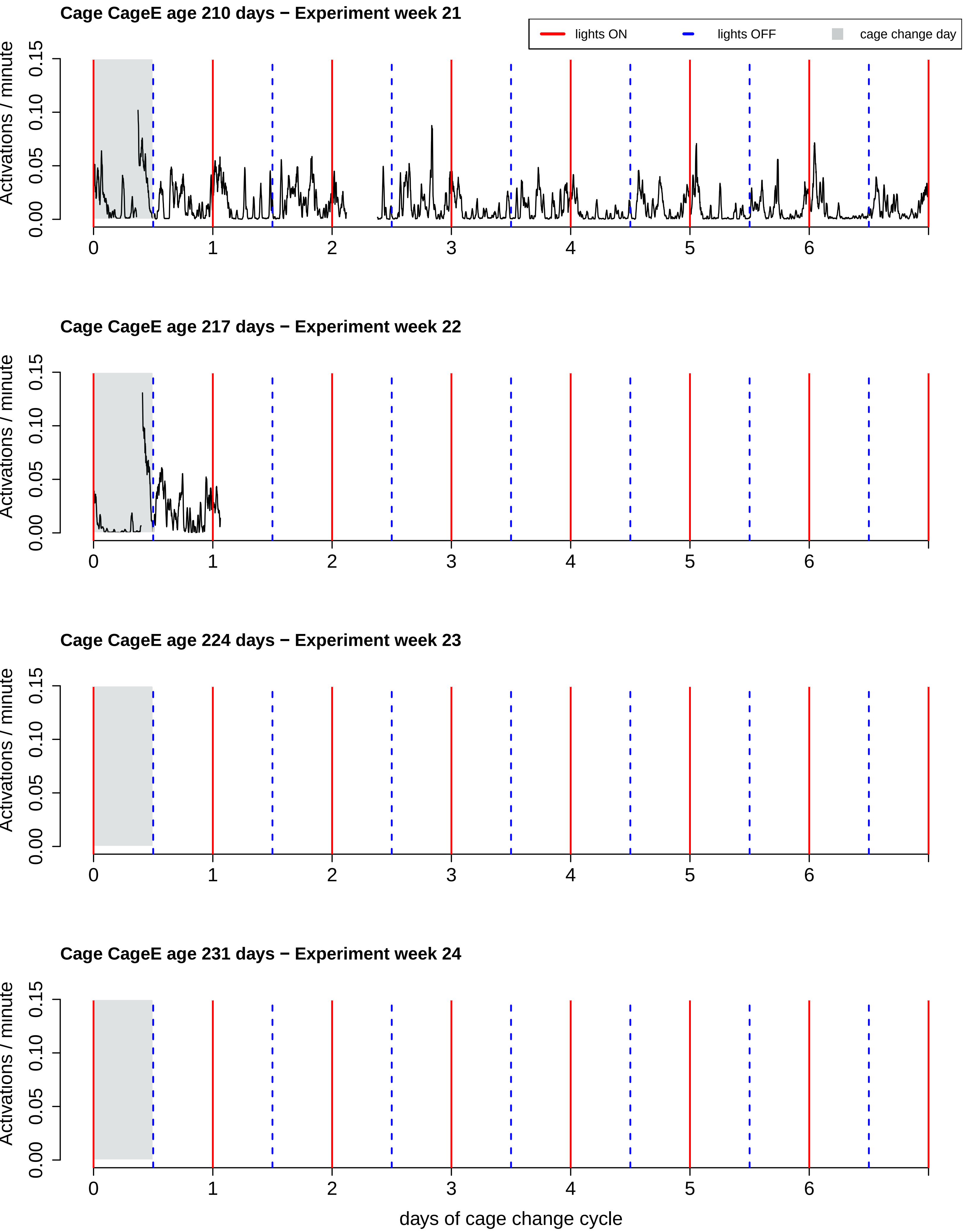
Cage CageE age 210 days − Experiment week 21
lights ON
lights OFF
cage change day
Activations / minute
0.00
0.05
0.10
0.15
0
1
2
3
4
5
6
Cage CageE age 217 days − Experiment week 22
Cage CageE age 224 days − Experiment week 23
Cage CageE age 231 days − Experiment week 24
days of cage change cycle

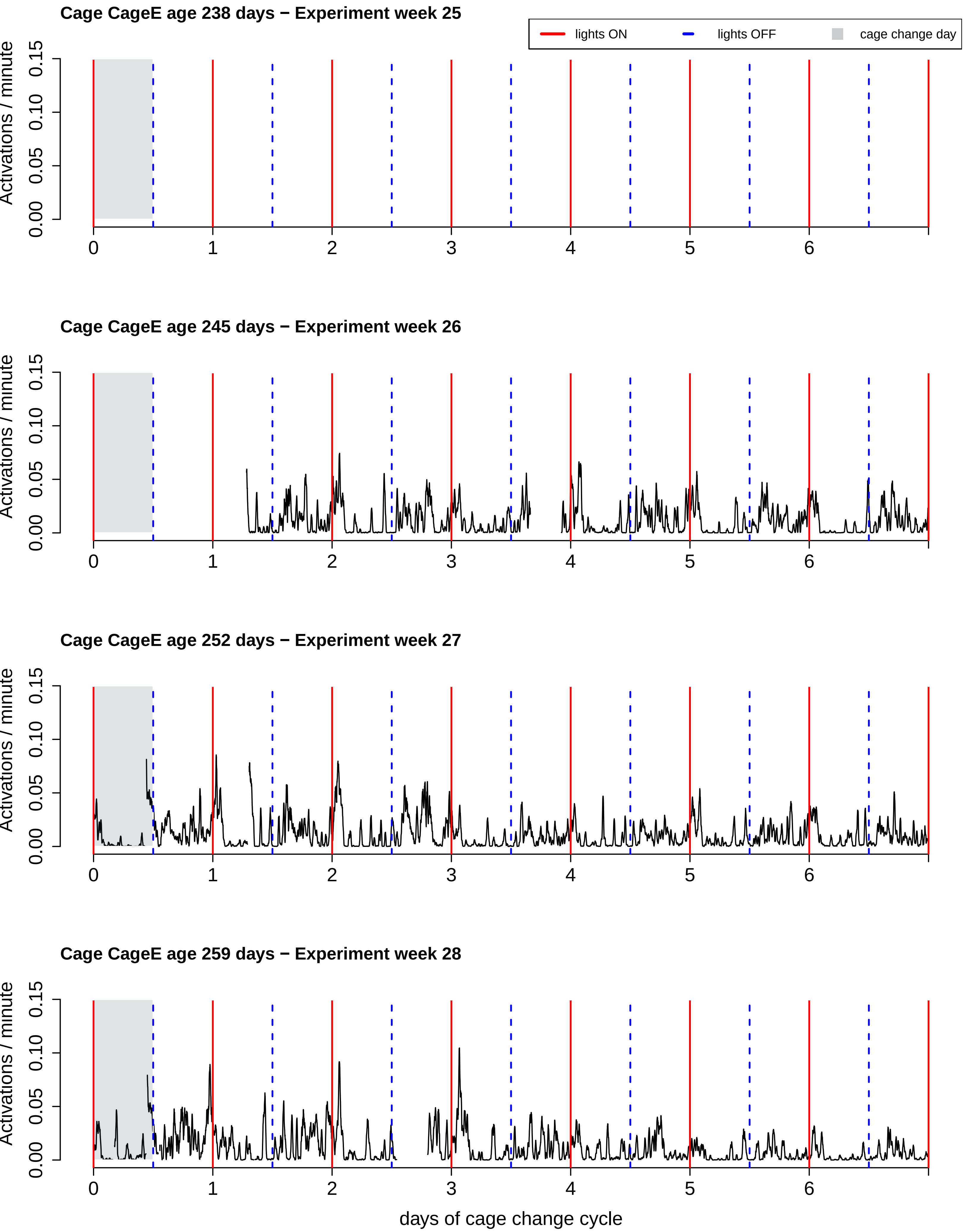

Cage CageE age 238 days − Experiment week 25
lights ON
lights OFF
cage change day
Activations / minute
0.00
0.05
0.10
0.15
0
1
2
3
4
5
6
Cage CageE age 245 days − Experiment week 26
Cage CageE age 252 days − Experiment week 27
Cage CageE age 259 days − Experiment week 28
days of cage change cycle

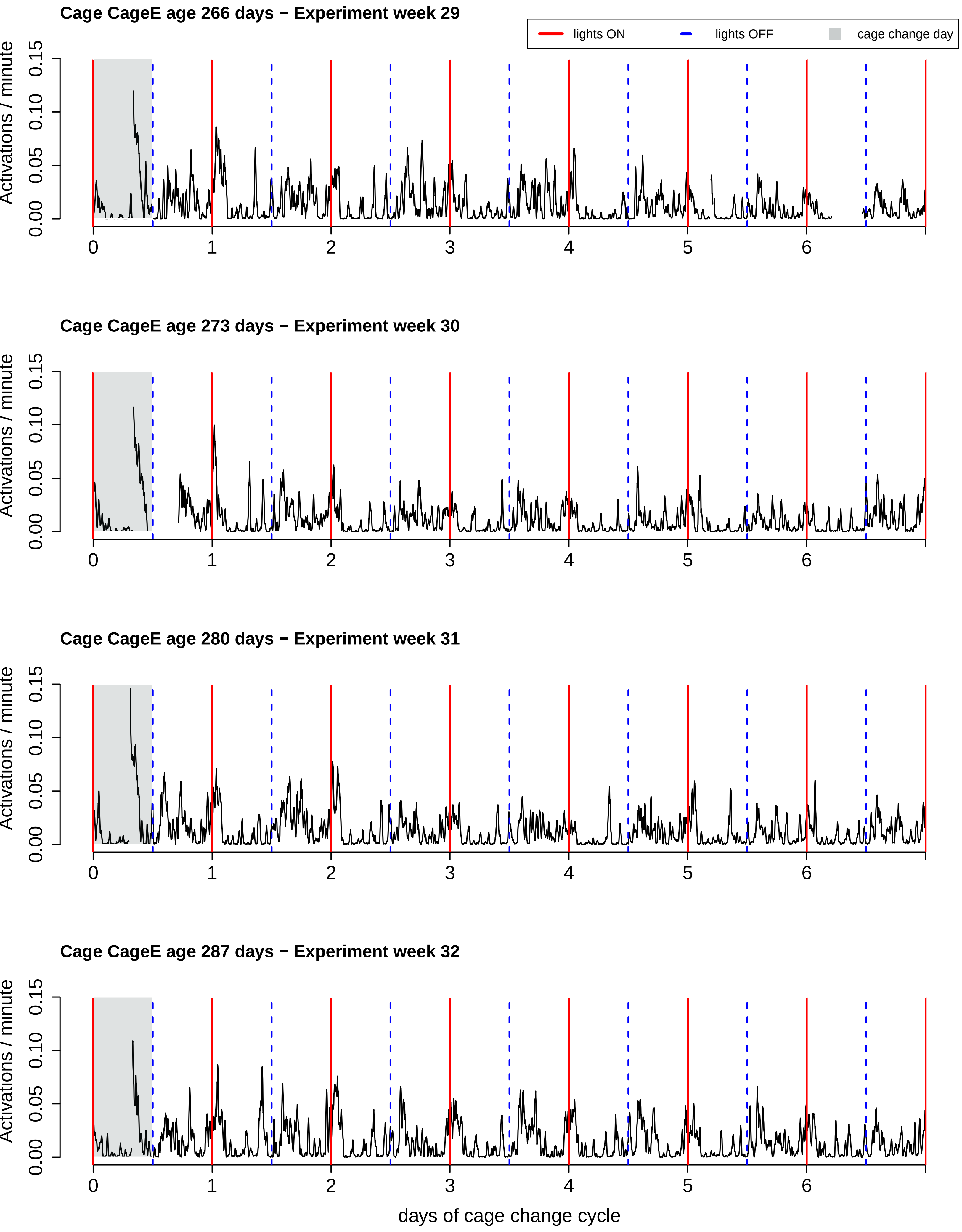
Cage CageE age 266 days − Experiment week 29
lights ON
lights OFF
cage change day
Activations / minute
0.00
0.05
0.10
0.15
0
1
2
3
4
5
6
Cage CageE age 273 days − Experiment week 30
Cage CageE age 280 days − Experiment week 31
Cage CageE age 287 days − Experiment week 32
days of cage change cycle

Cage CageE age 294 days − Experiment week 33

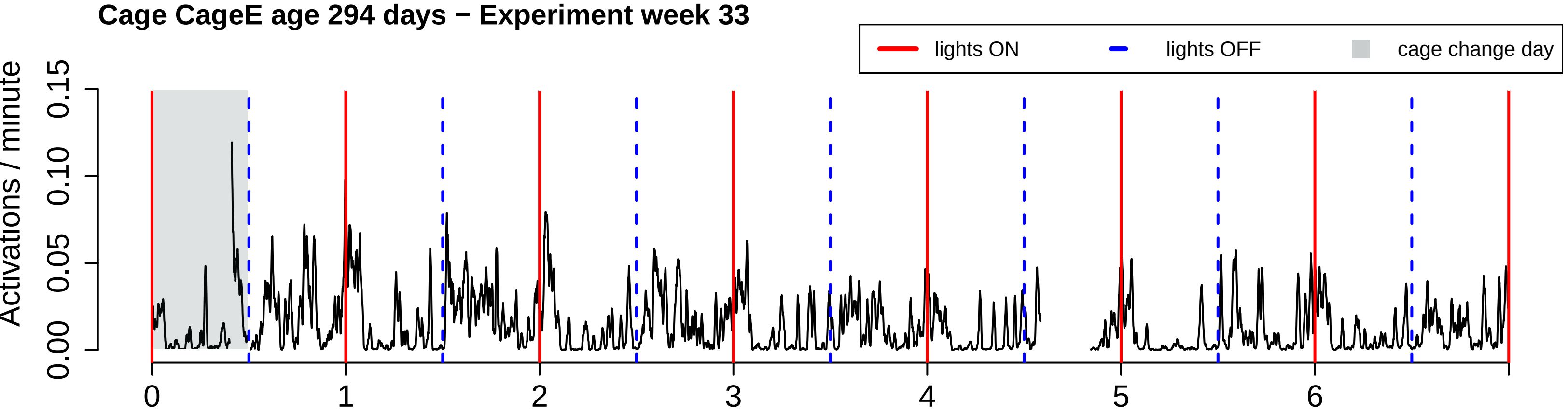

Cage CageE age 301 days − Experiment week 34

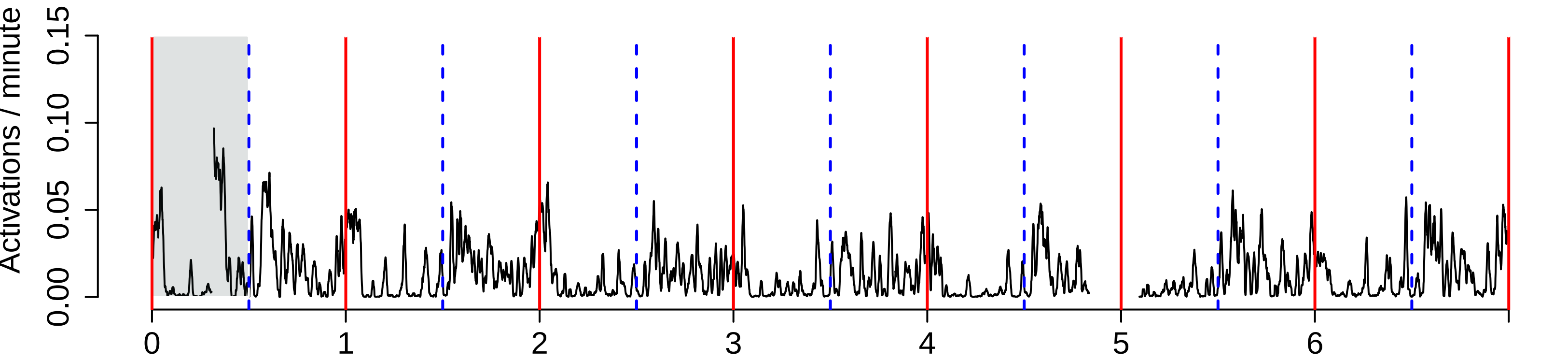

Cage CageE age 308 days − Experiment week 35

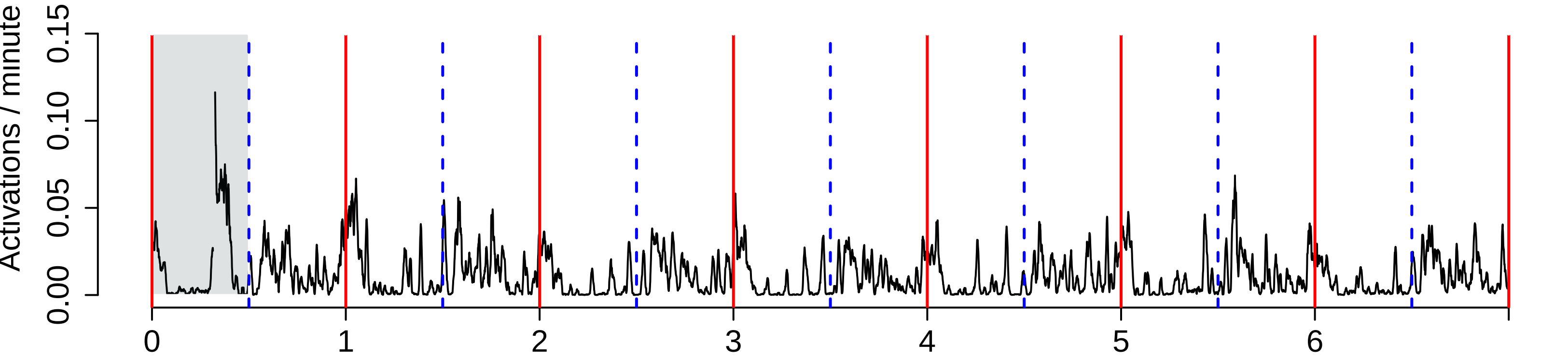

Cage CageE age 315 days − Experiment week 36

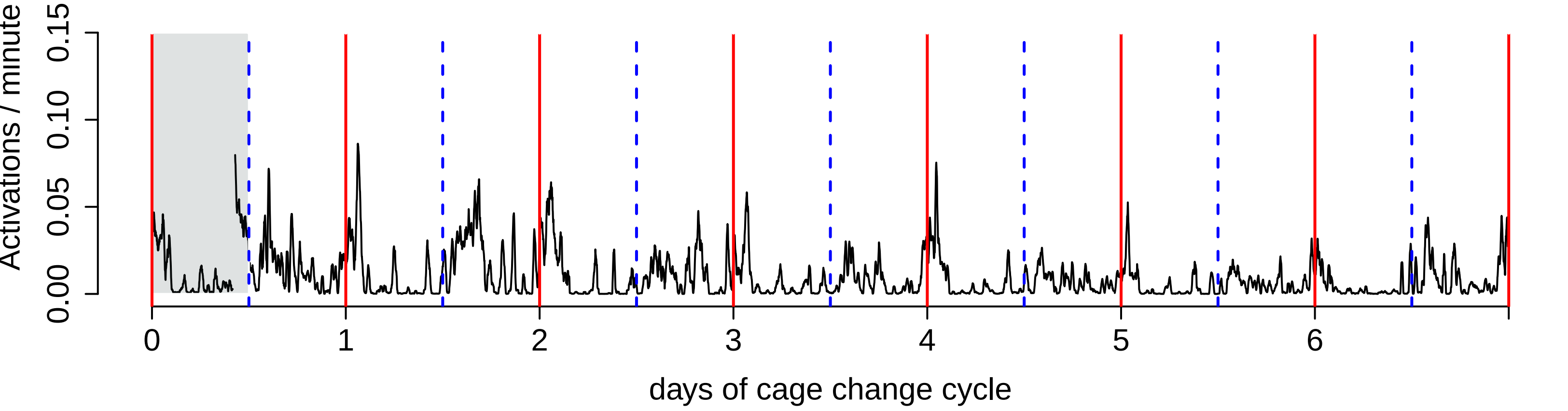

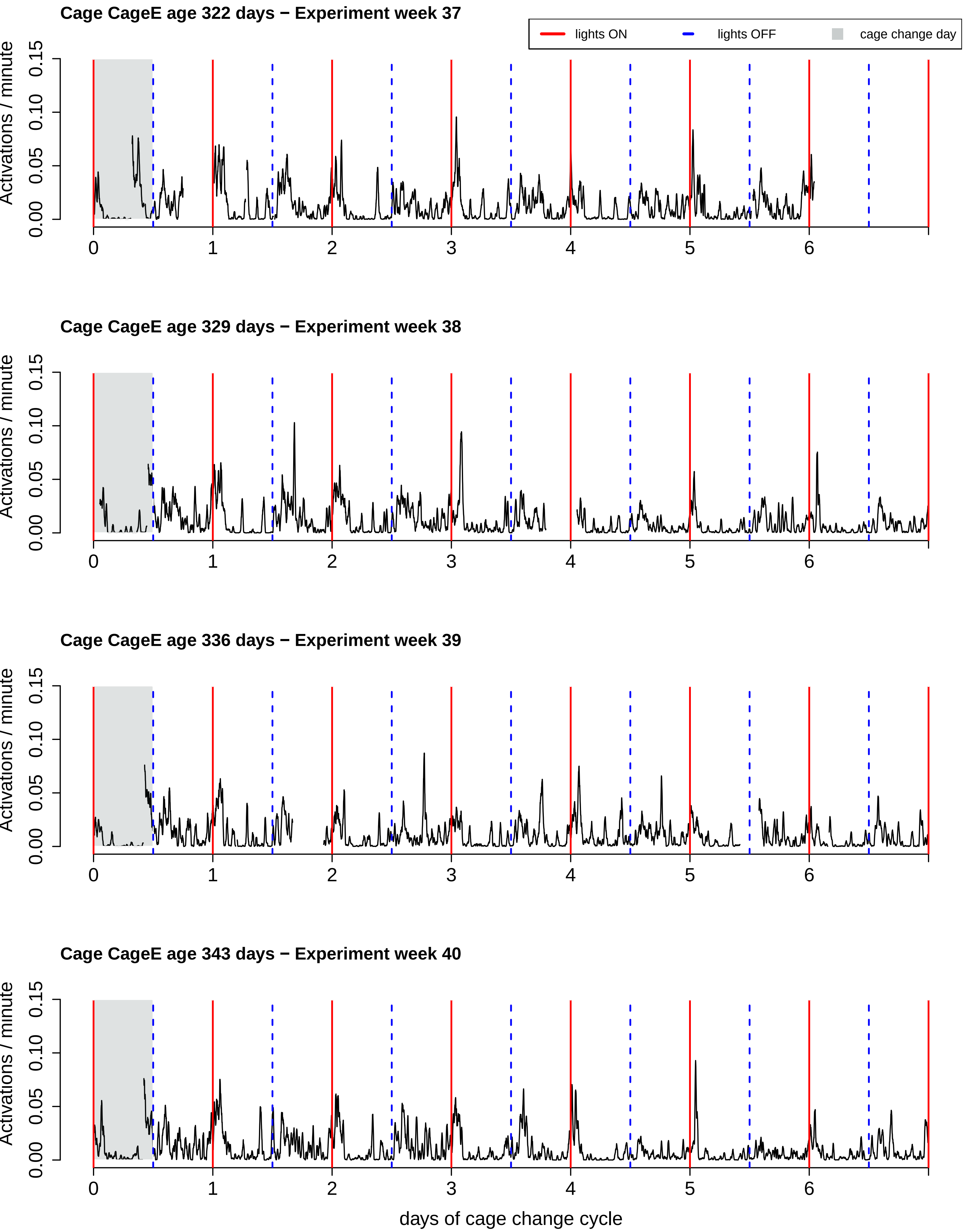
Cage CageE age 322 days − Experiment week 37
lights ON
lights OFF
cage change day
Activations / minute
0.00
0.05
0.10
0.15
0
1
2
3
4
5
6
Cage CageE age 329 days − Experiment week 38
Activations / minute
Cage CageE age 336 days − Experiment week 39
Activations / minute
Cage CageE age 343 days − Experiment week 40
Activations / minute
days of cage change cycle

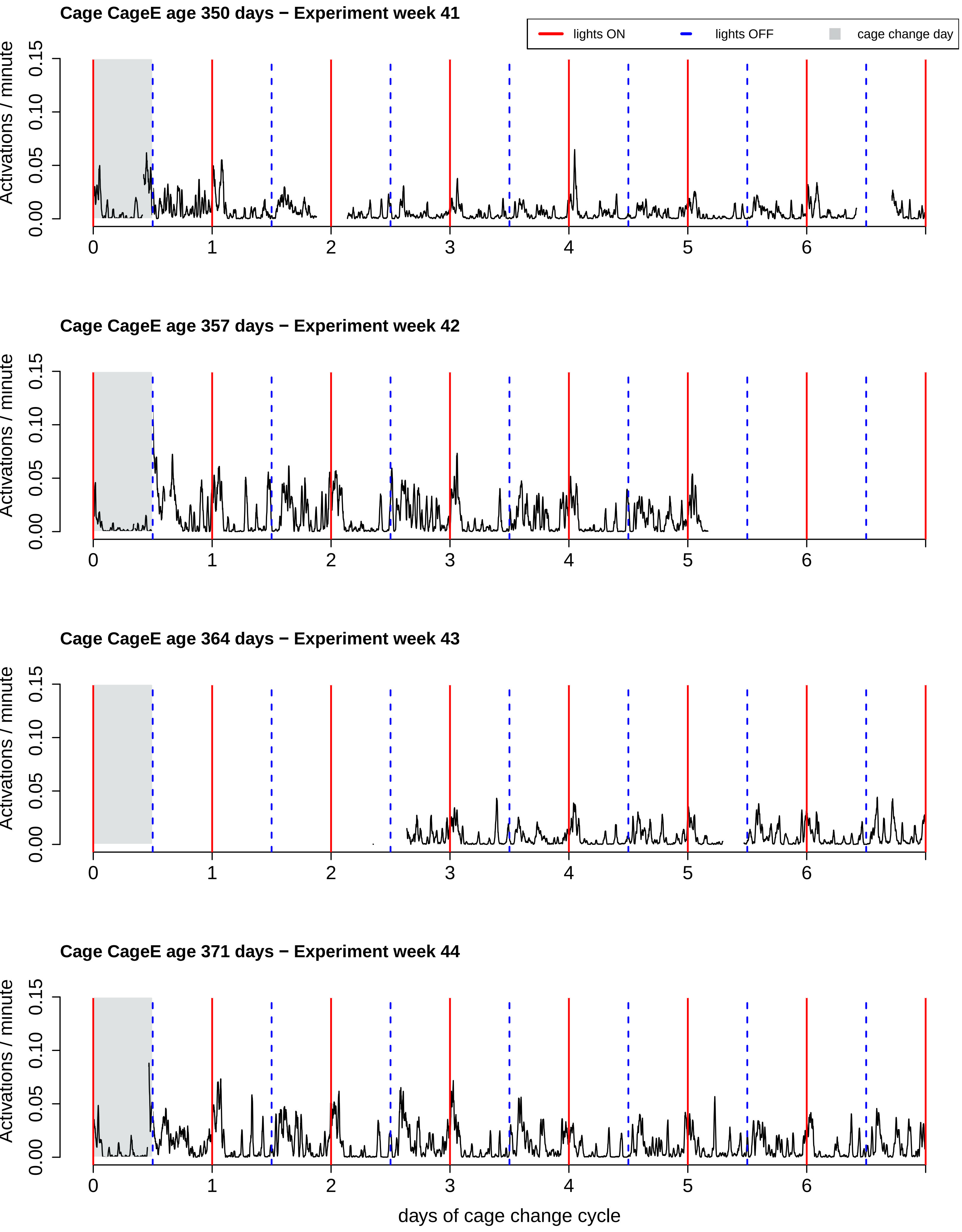
Cage CageE age 350 days − Experiment week 41
lights ON
lights OFF
cage change day
Activations / minute
0.00
0.05
0.10
0.15
0
1
2
3
4
5
6
Cage CageE age 357 days − Experiment week 42
Activations / minute
Cage CageE age 364 days − Experiment week 43
Activations / minute
Cage CageE age 371 days − Experiment week 44
Activations / minute
days of cage change cycle

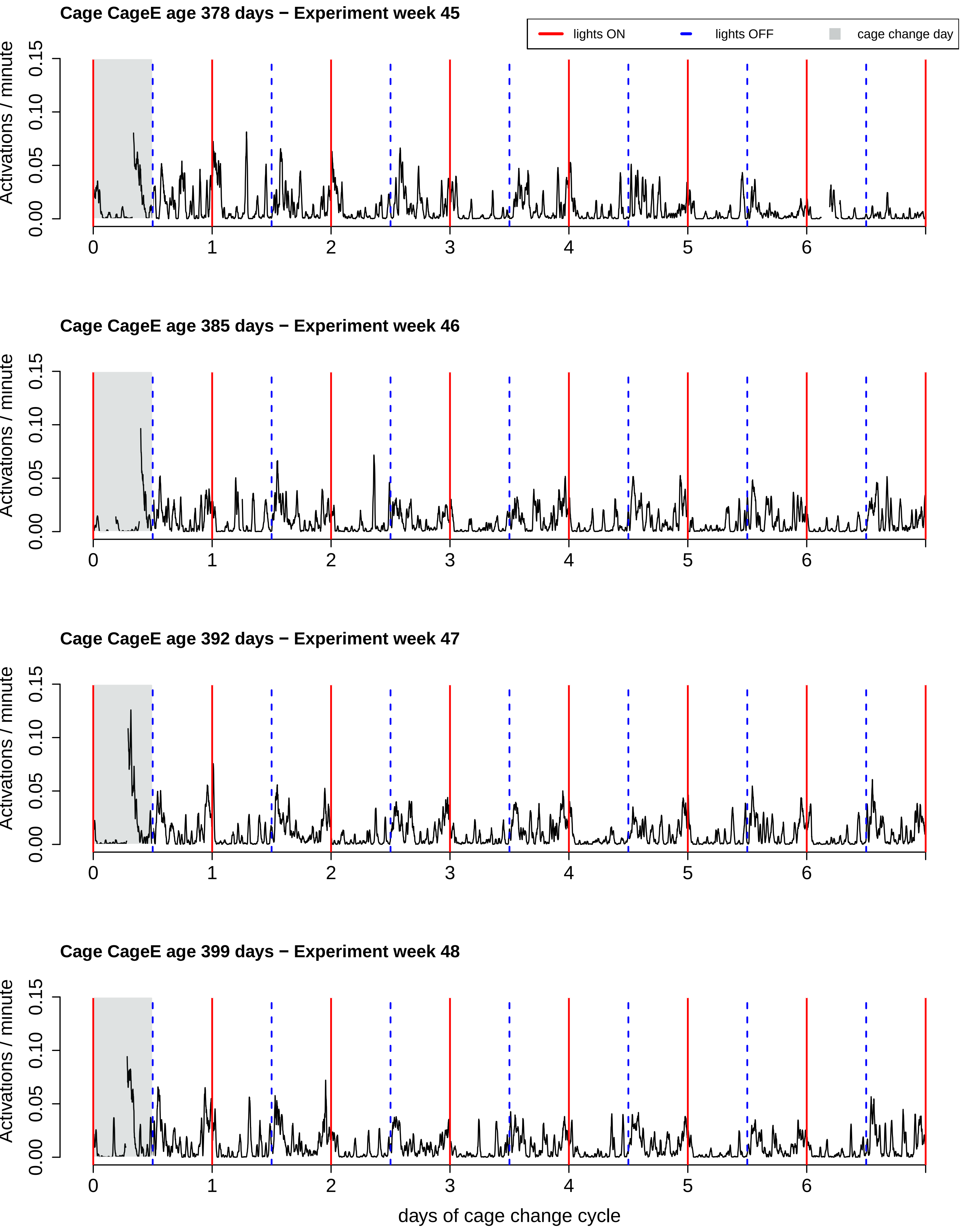
Cage CageE age 378 days − Experiment week 45
lights ON
lights OFF
cage change day
Activations / minute
0.00
0.05
0.10
0.15
0
1
2
3
4
5
6
Cage CageE age 385 days − Experiment week 46
Cage CageE age 392 days − Experiment week 47
Cage CageE age 399 days − Experiment week 48
days of cage change cycle

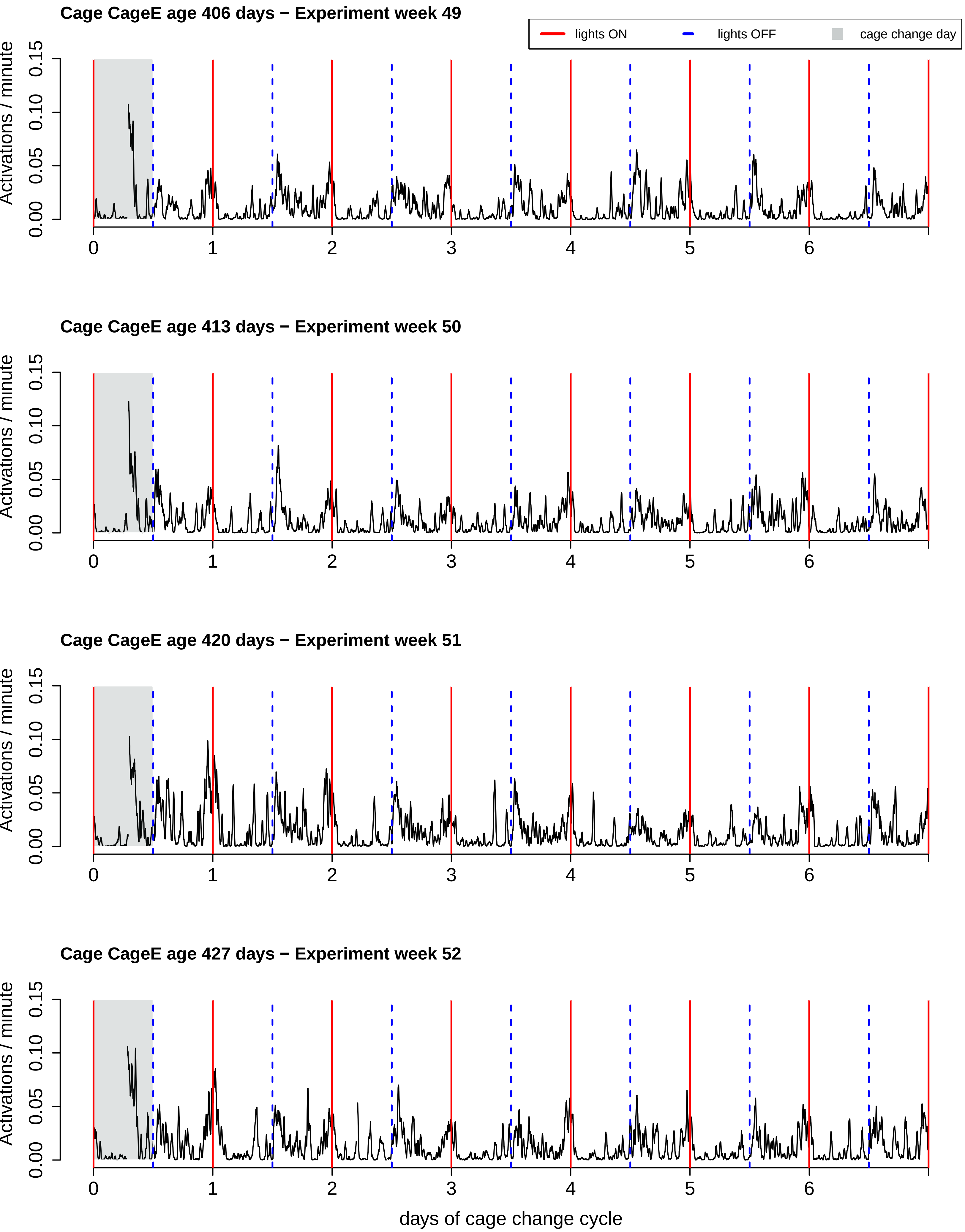
Cage CageE age 406 days − Experiment week 49
lights ON
lights OFF
cage change day
Activations / minute
0.00
0.05
0.10
0.15
0
1
2
3
4
5
6
Cage CageE age 413 days − Experiment week 50
Cage CageE age 420 days − Experiment week 51
Cage CageE age 427 days − Experiment week 52
days of cage change cycle

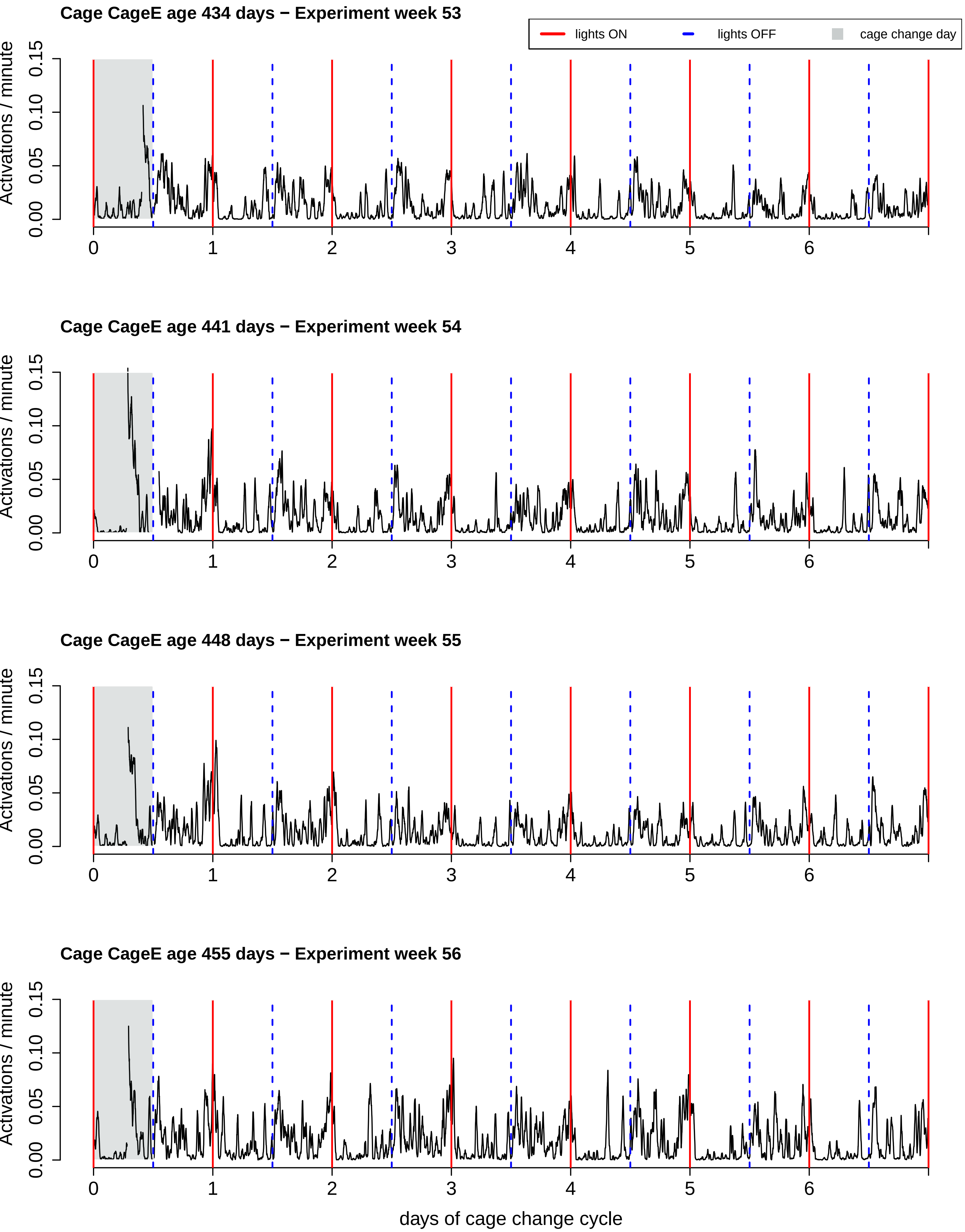

Cage CageE age 434 days − Experiment week 53
lights ON
lights OFF
cage change day
Cage CageE age 441 days − Experiment week 54
Cage CageE age 448 days − Experiment week 55
Cage CageE age 455 days − Experiment week 56
Activations / minute
0.00
0.05
0.10
0.15
0
1
2
3
4
5
6
days of cage change cycle

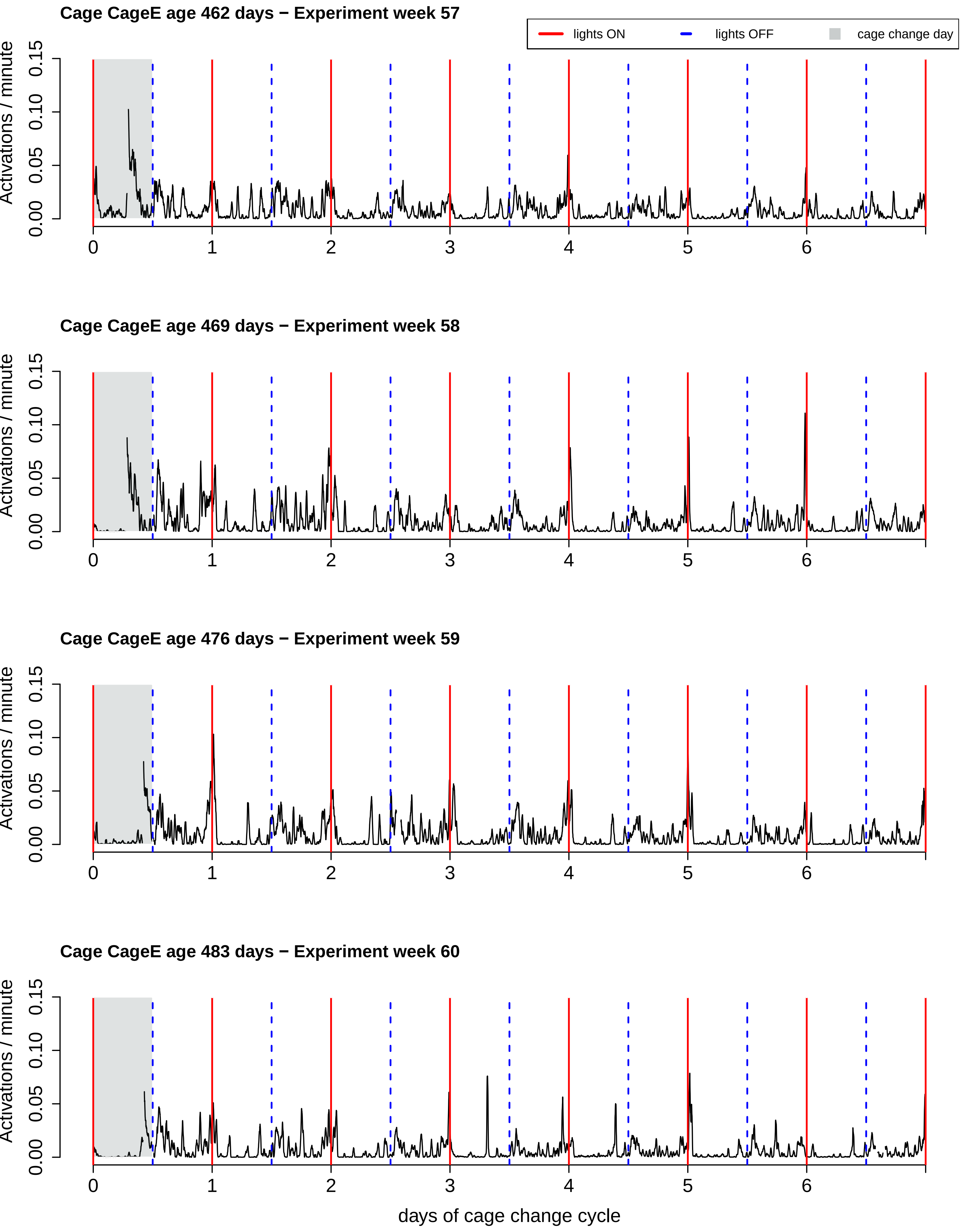

Cage CageE age 462 days − Experiment week 57
lights ON
lights OFF
cage change day
Activations / minute
0.00
0.05
0.10
0.15
0
1
2
3
4
5
6
Cage CageE age 469 days − Experiment week 58
Cage CageE age 476 days − Experiment week 59
Cage CageE age 483 days − Experiment week 60
days of cage change cycle

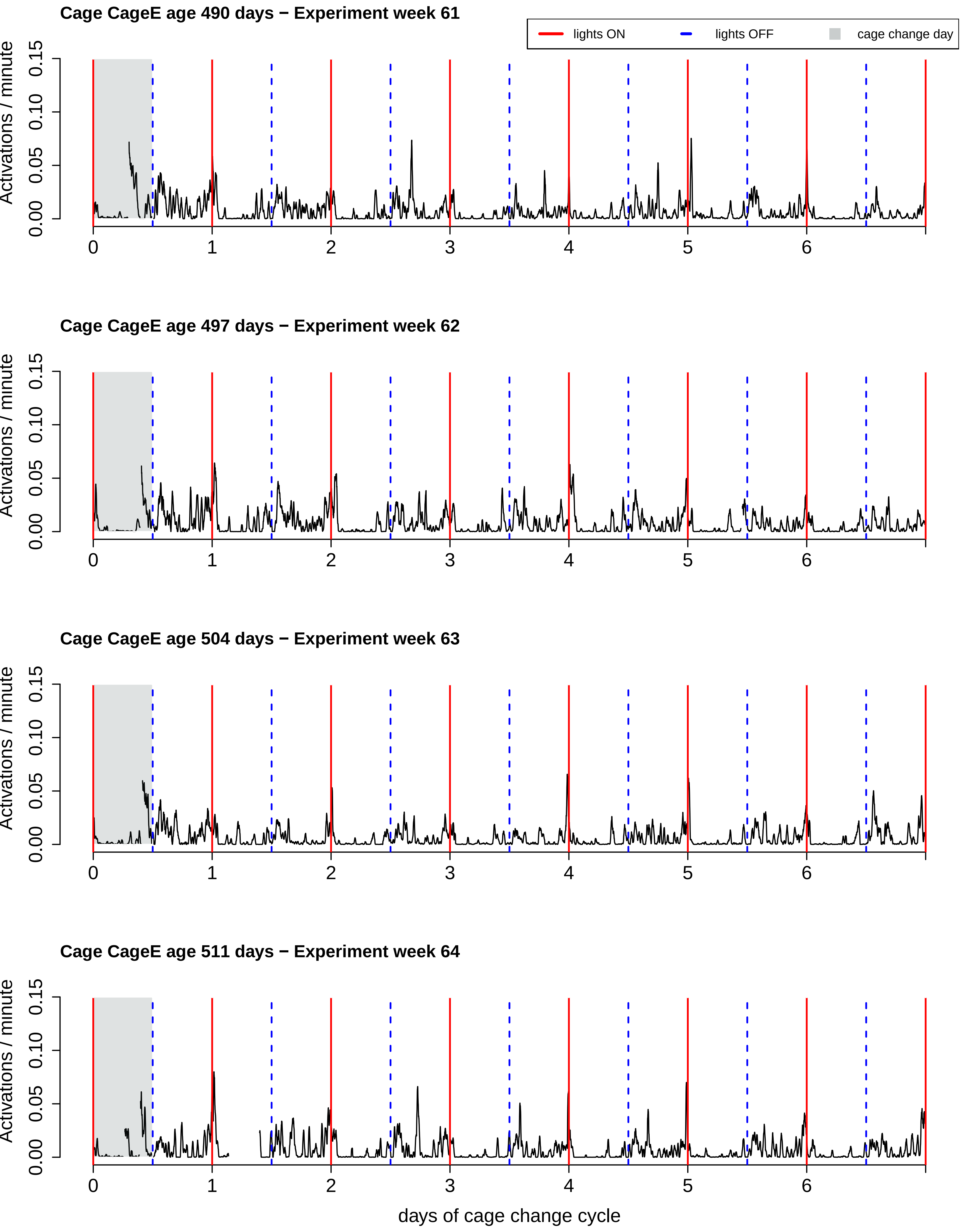

Cage CageE age 490 days − Experiment week 61
lights ON
lights OFF
cage change day
Activations / minute
0.00
0.05
0.10
0.15
0
1
2
3
4
5
6
Cage CageE age 497 days − Experiment week 62
Cage CageE age 504 days − Experiment week 63
Cage CageE age 511 days − Experiment week 64
days of cage change cycle

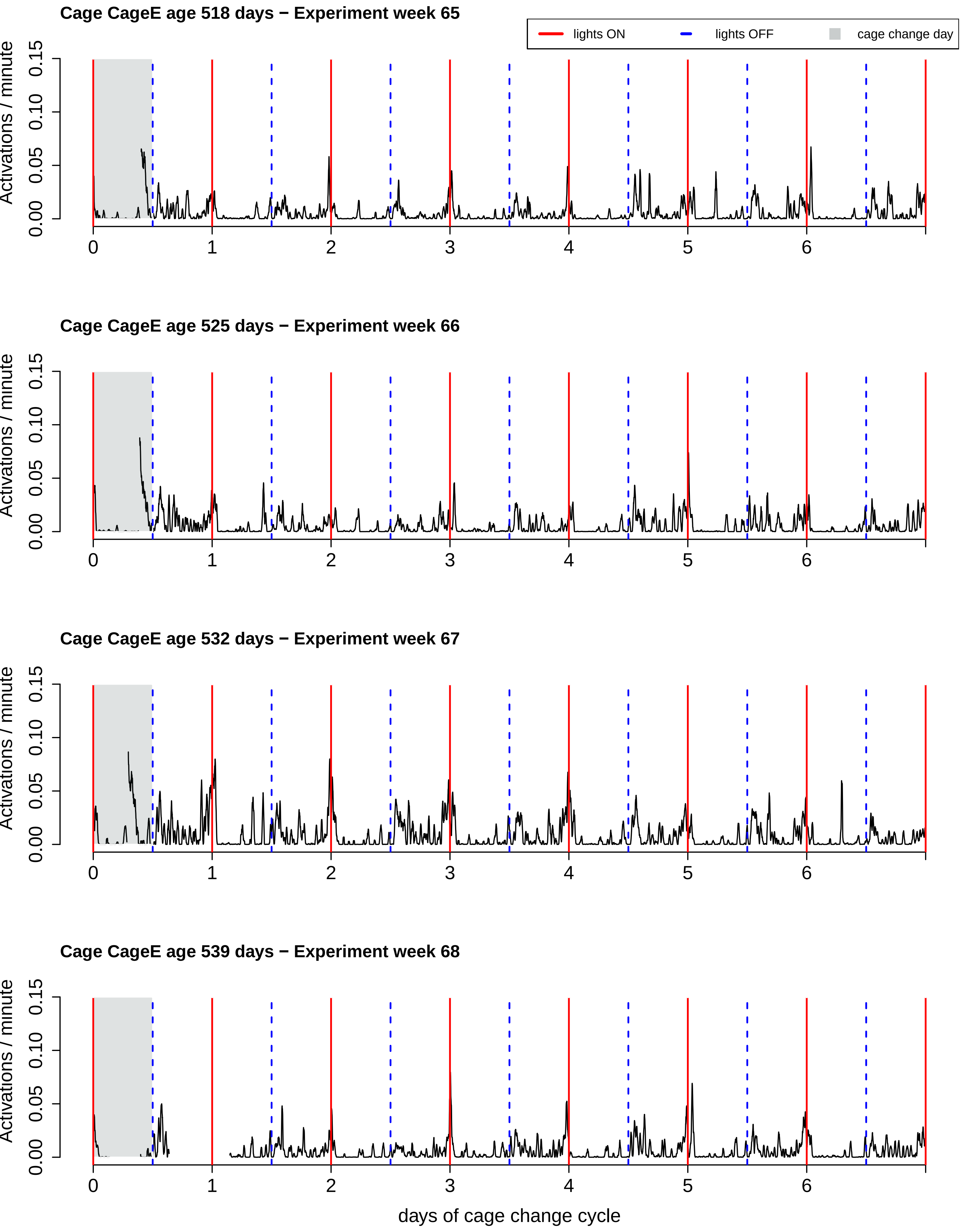
Cage CageE age 518 days − Experiment week 65
lights ON
lights OFF
cage change day
Activations / minute
0.00
0.05
0.10
0.15
0
1
2
3
4
5
6
Cage CageE age 525 days − Experiment week 66
Cage CageE age 532 days − Experiment week 67
Cage CageE age 539 days − Experiment week 68
days of cage change cycle

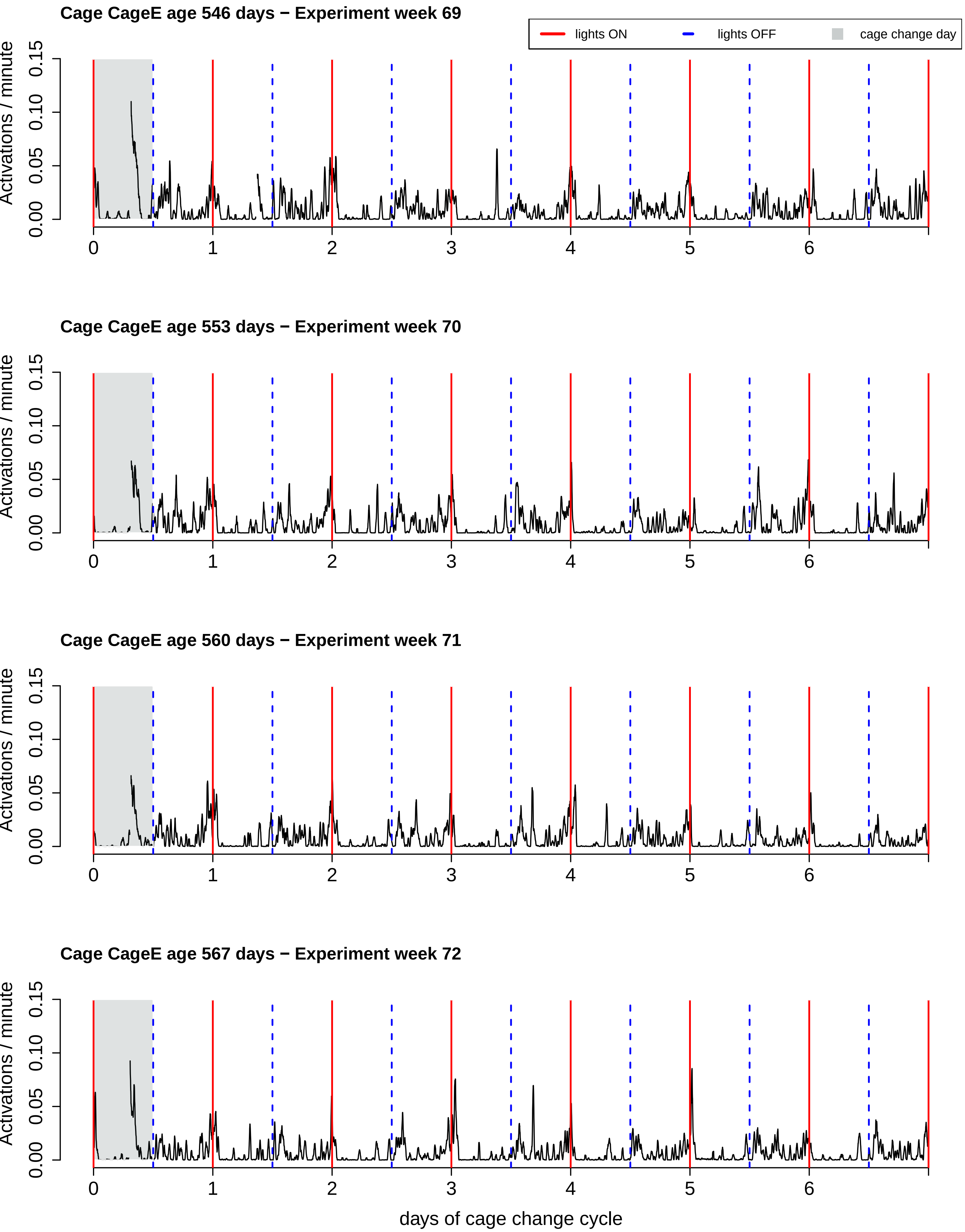
Cage CageE age 546 days − Experiment week 69
lights ON
lights OFF
cage change day
Activations / minute
0.00
0.05
0.10
0.15
0
1
2
3
4
5
6
Cage CageE age 553 days − Experiment week 70
Cage CageE age 560 days − Experiment week 71
Cage CageE age 567 days − Experiment week 72
days of cage change cycle

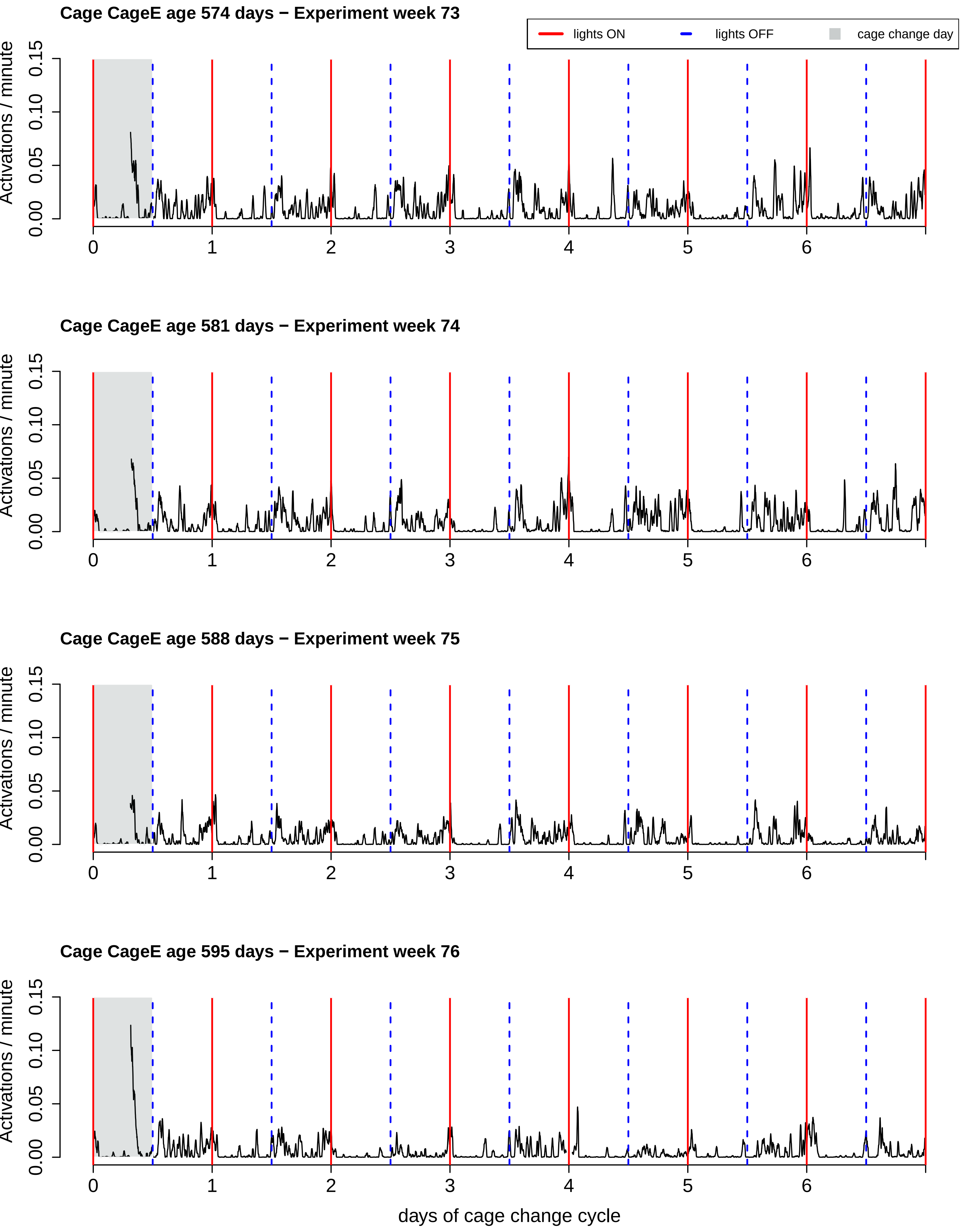
Cage CageE age 574 days − Experiment week 73
lights ON
lights OFF
cage change day
Activations / minute
0.00
0.05
0.10
0.15
0
1
2
3
4
5
6
Cage CageE age 581 days − Experiment week 74
Cage CageE age 588 days − Experiment week 75
Cage CageE age 595 days − Experiment week 76
days of cage change cycle

Cage CageE age 602 days − Experiment week 77

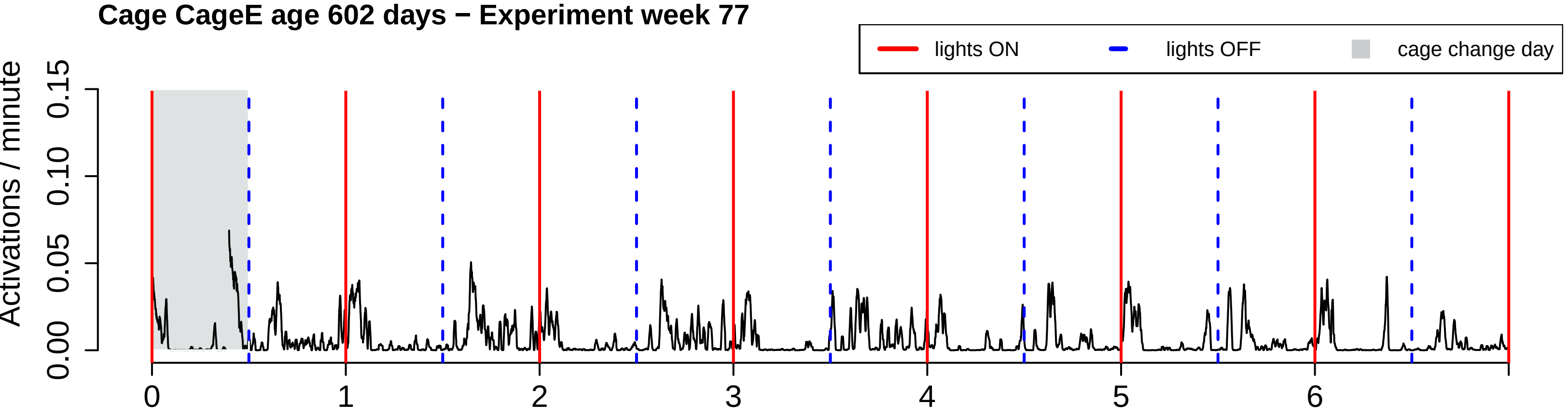

Cage CageE age 609 days − Experiment week 78

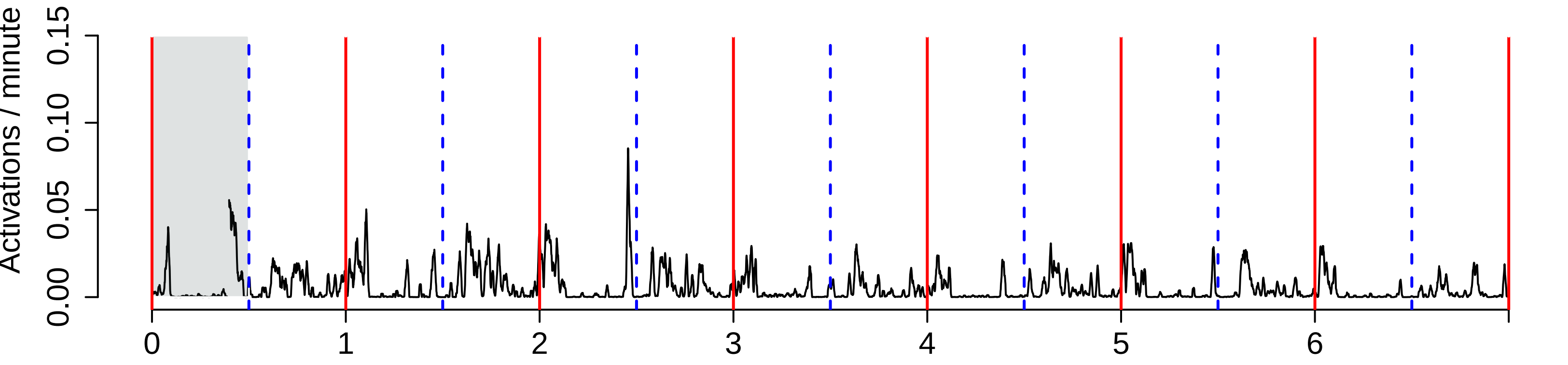

Cage CageE age 616 days − Experiment week 79

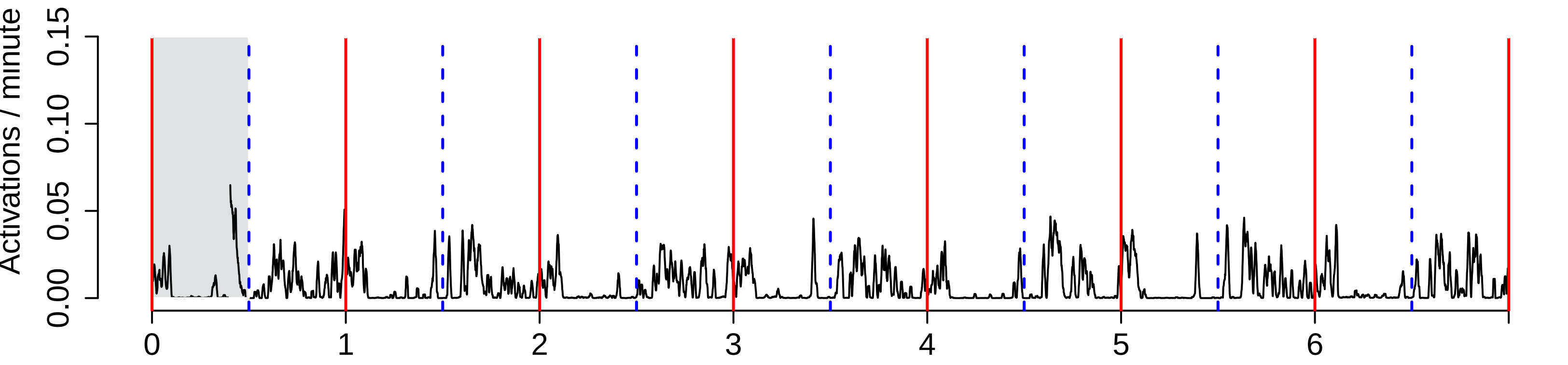

Cage CageE age 623 days − Experiment week 80

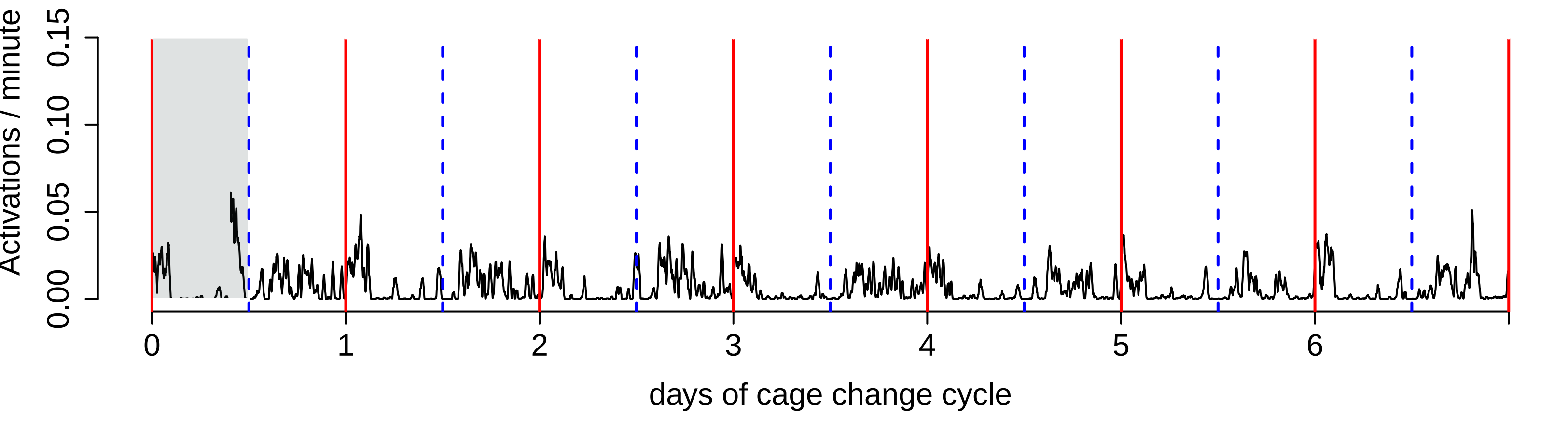

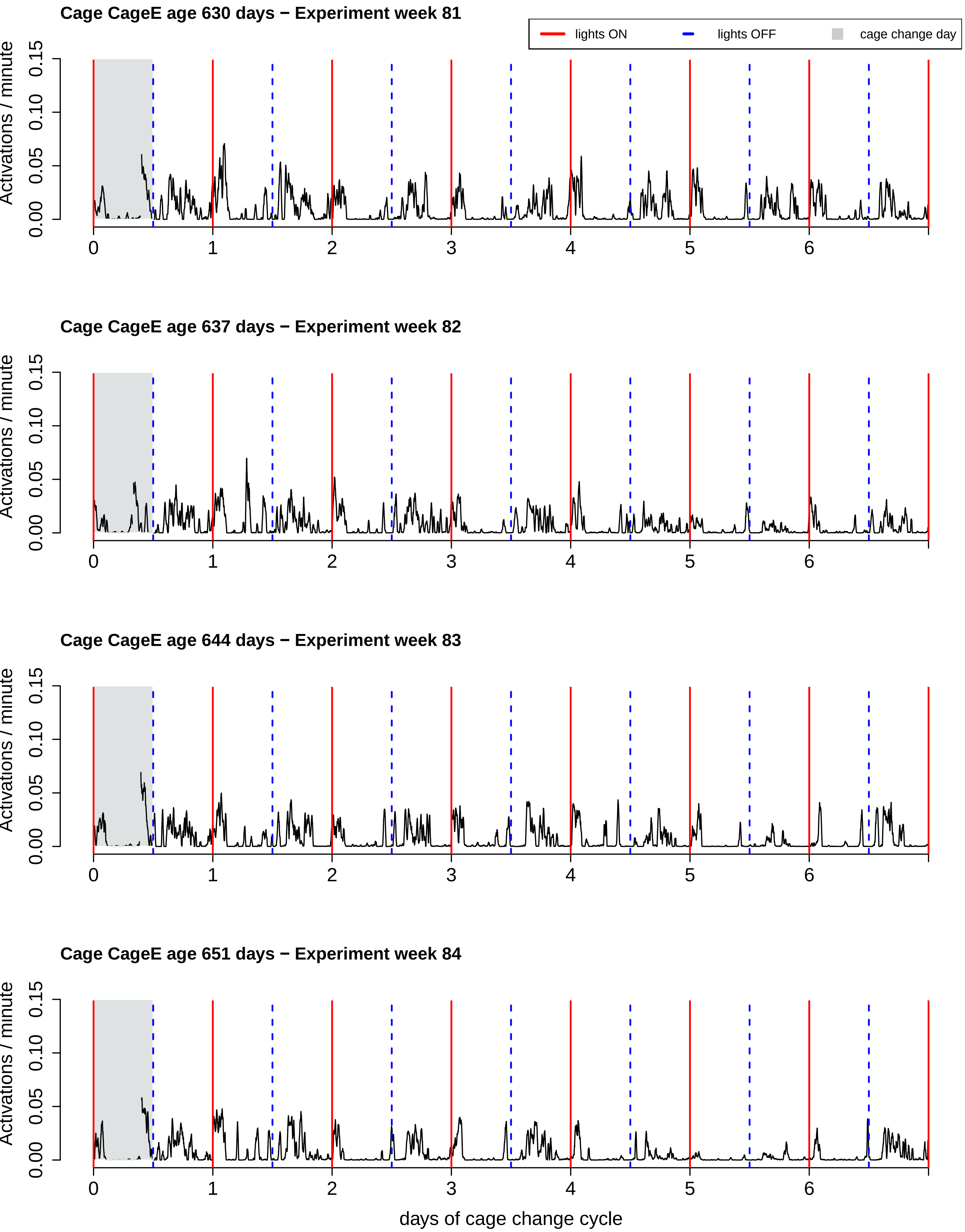

Cage CageE age 630 days − Experiment week 81
Cage CageE age 637 days − Experiment week 82
Cage CageE age 644 days − Experiment week 83
Cage CageE age 651 days − Experiment week 84
lights ON
lights OFF
cage change day
Activations / minute
0.00
0.05
0.10
0.15
0
1
2
3
4
5
6
days of cage change cycle

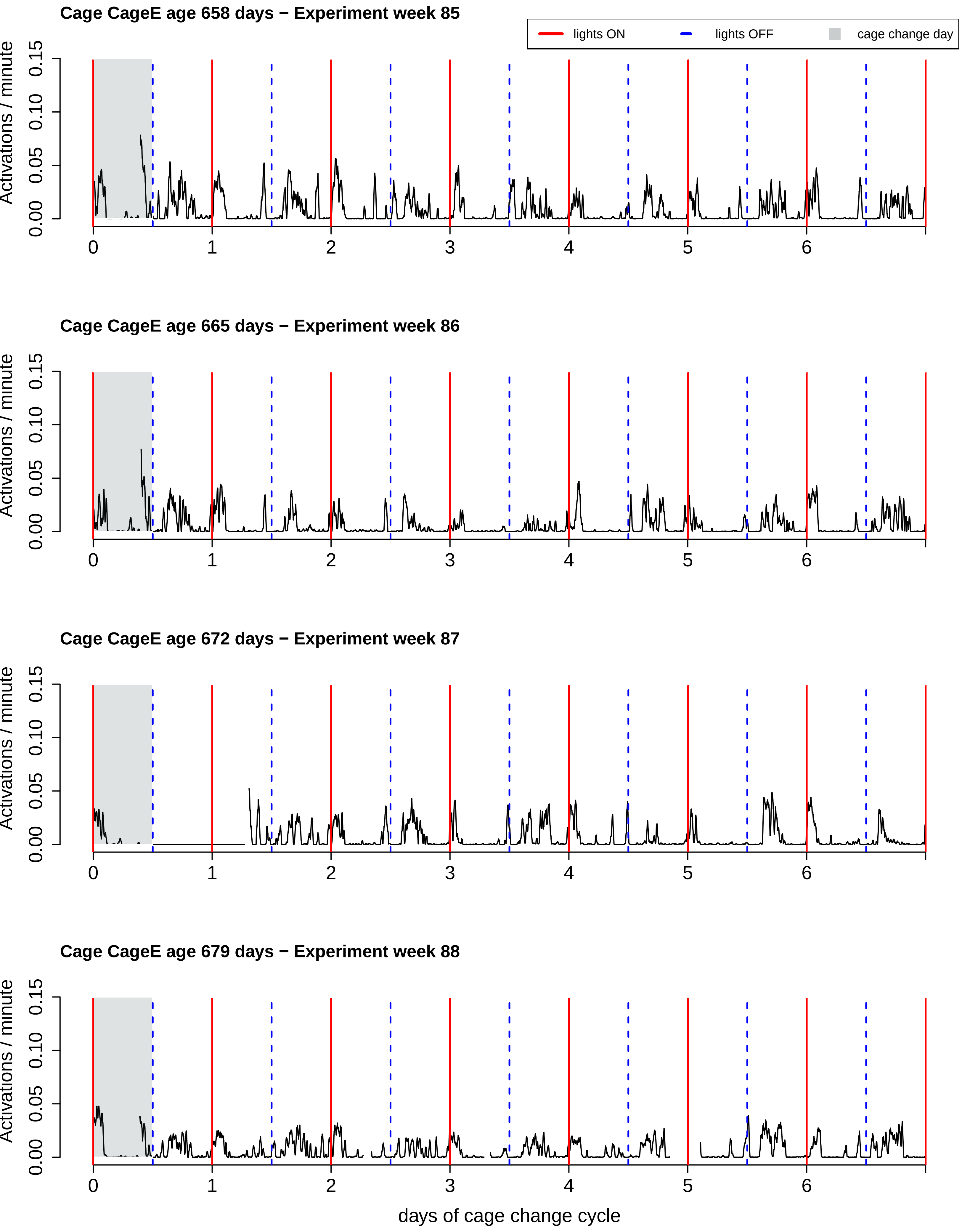
Cage CageE age 658 days − Experiment week 85
lights ON
lights OFF
cage change day
Activations / minute
0.00
0.05
0.10
0.15
0
1
2
3
4
5
6
Cage CageE age 665 days − Experiment week 86
Cage CageE age 672 days − Experiment week 87
Cage CageE age 679 days − Experiment week 88
days of cage change cycle

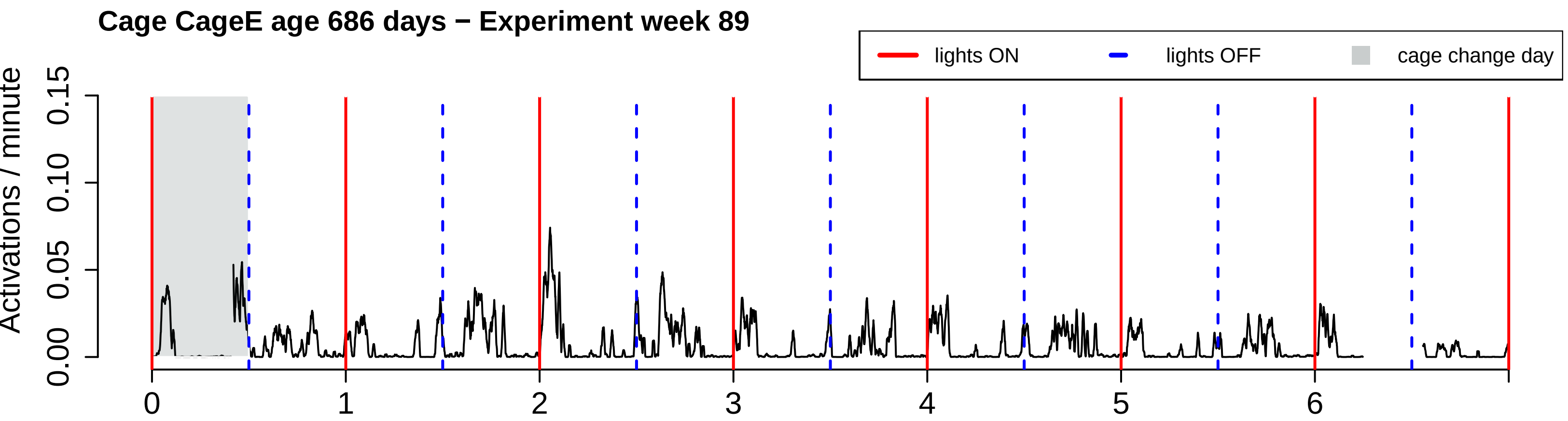
Cage CageE age 686 days − Experiment week 89
lights ON
lights OFF
cage change day
Activations / minute
0.00
0.05
0.10
0.15
0
1
2
3
4
5
6

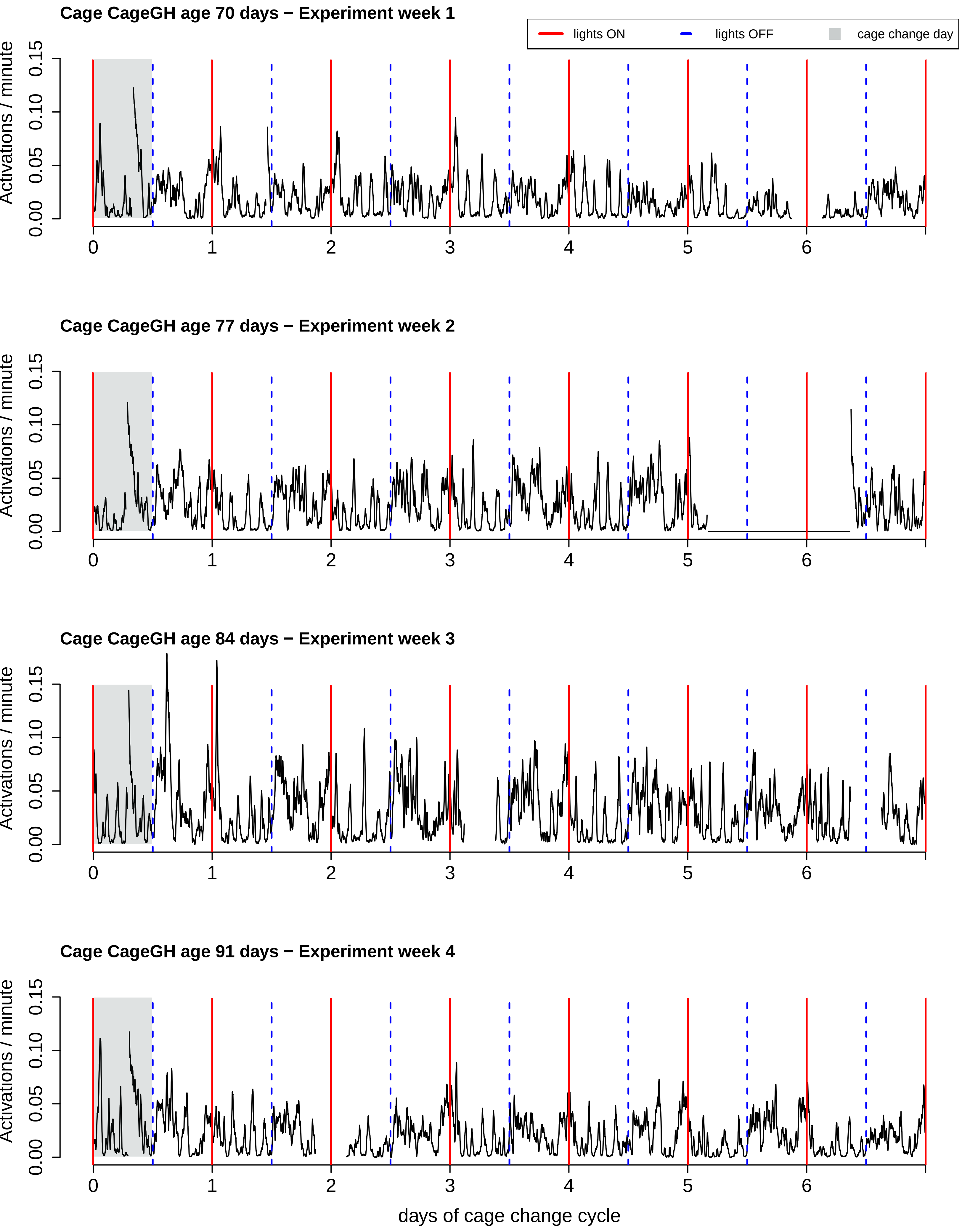
Cage CageGH age 70 days − Experiment week 1
lights ON
lights OFF
cage change day
Activations / minute
0.00
0.05
0.10
0.15
0
1
2
3
4
5
6
Cage CageGH age 77 days − Experiment week 2
Cage CageGH age 84 days − Experiment week 3
Cage CageGH age 91 days − Experiment week 4
days of cage change cycle

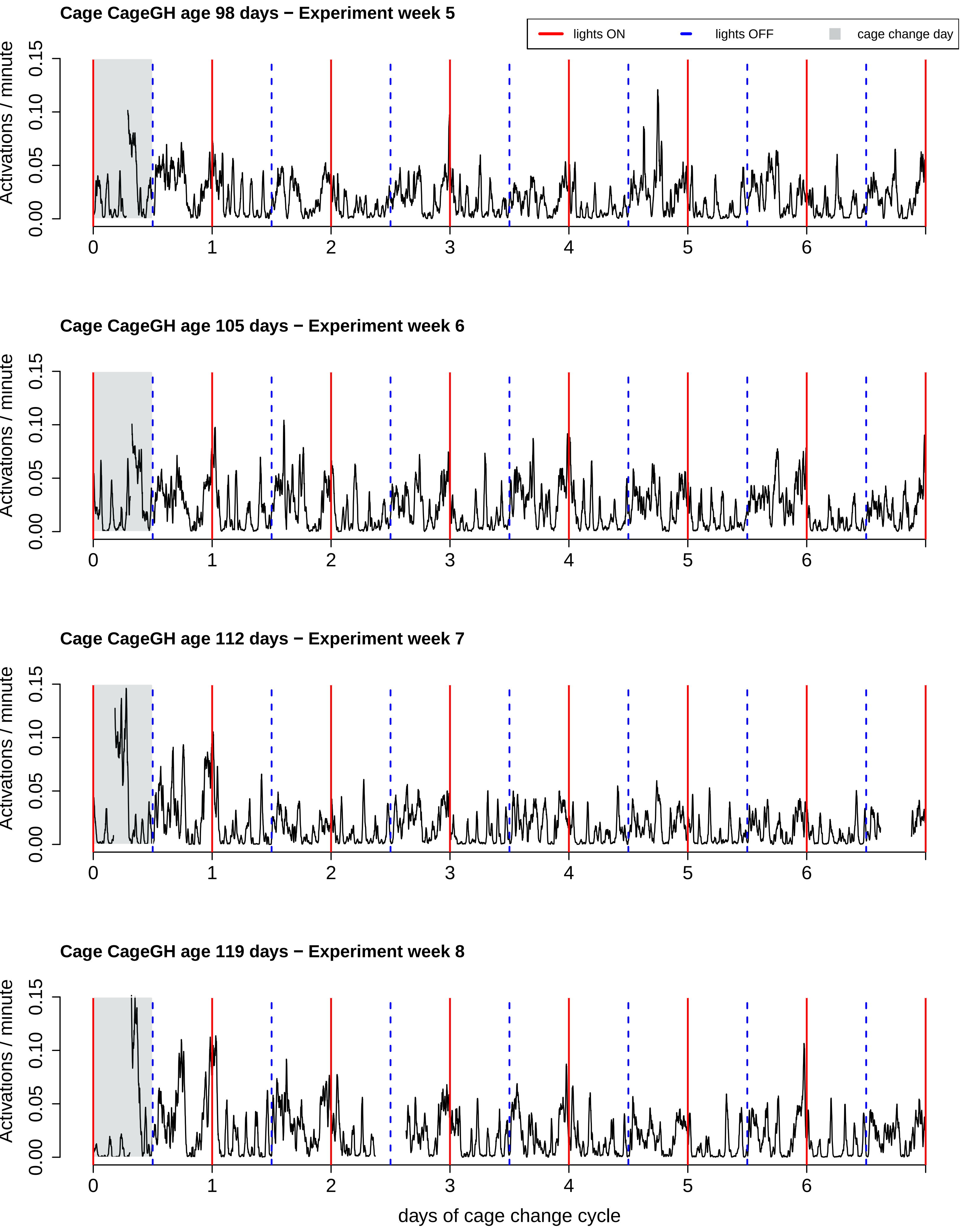

Cage CageGH age 98 days − Experiment week 5
lights ON
lights OFF
cage change day
Activations / minute
0.00
0.05
0.10
0.15
0
1
2
3
4
5
6
Cage CageGH age 105 days − Experiment week 6
Cage CageGH age 112 days − Experiment week 7
Cage CageGH age 119 days − Experiment week 8
days of cage change cycle

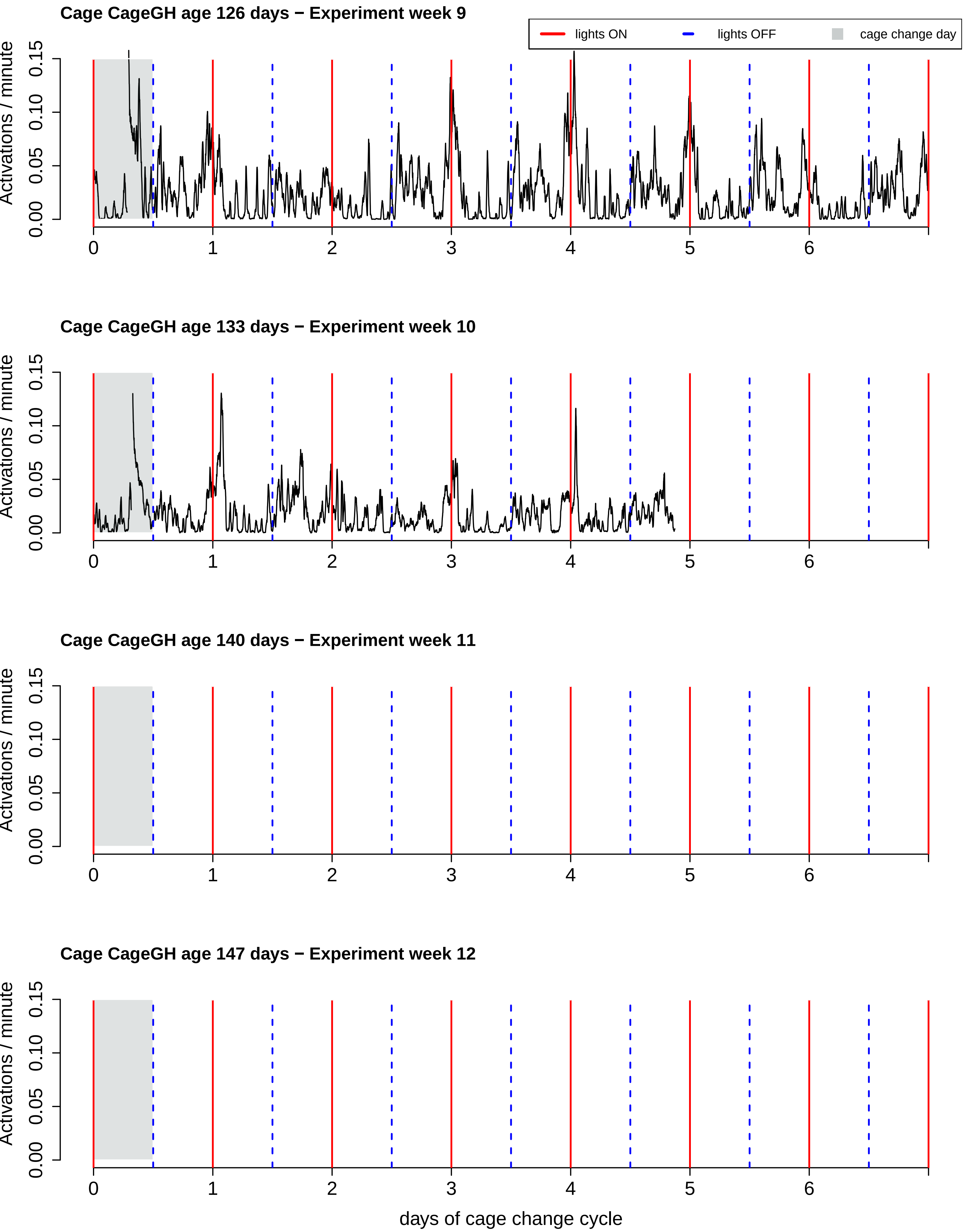
Cage CageGH age 126 days − Experiment week 9
lights ON
lights OFF
cage change day
Activations / minute
0.00
0.05
0.10
0.15
0
1
2
3
4
5
6
Cage CageGH age 133 days − Experiment week 10
Cage CageGH age 140 days − Experiment week 11
Cage CageGH age 147 days − Experiment week 12
days of cage change cycle

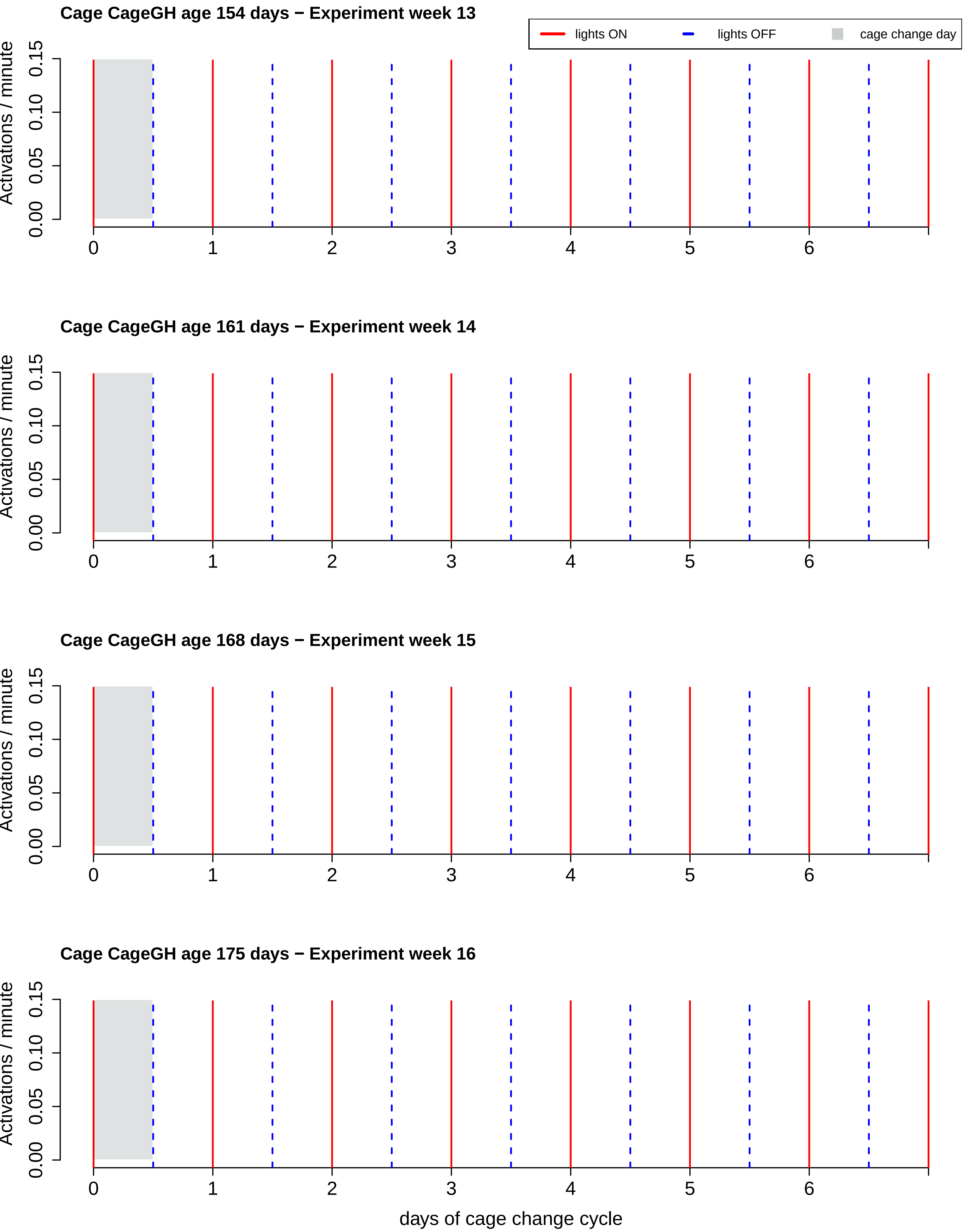
Cage CageGH age 154 days − Experiment week 13
lights ON
lights OFF
cage change day
Activations / minute
0.00
0.05
0.10
0.15
0
1
2
3
4
5
6
Cage CageGH age 161 days − Experiment week 14
Cage CageGH age 168 days − Experiment week 15
Cage CageGH age 175 days − Experiment week 16
days of cage change cycle

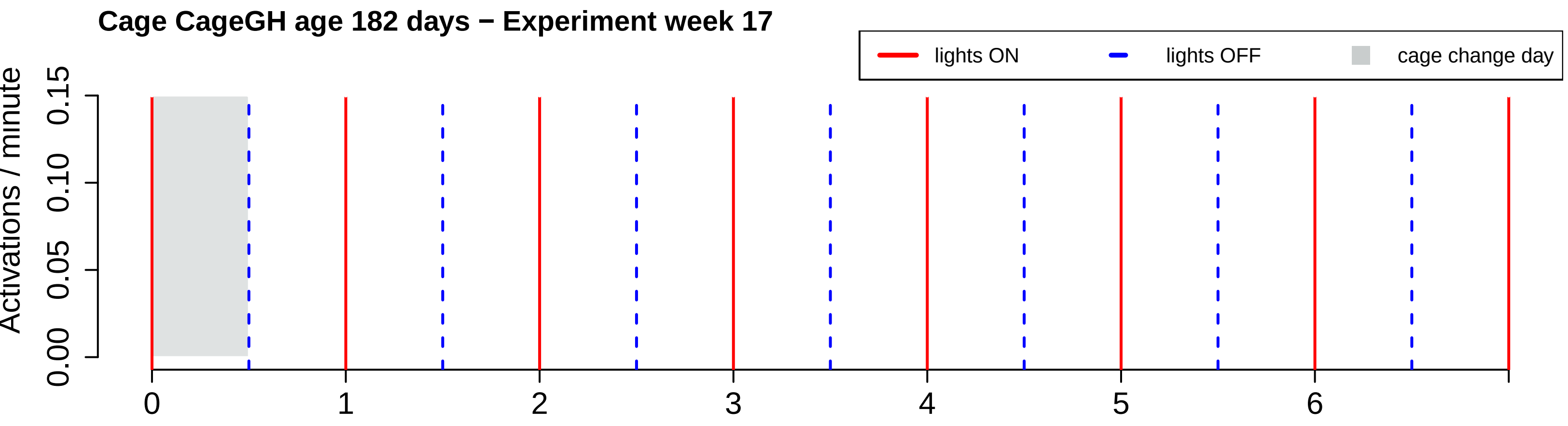

Cage CageGH age 182 days − Experiment week 17
lights ON
lights OFF
cage change day
Activations / minute
0.00
0.05
0.10
0.15
0 1 2 3 4 5 6

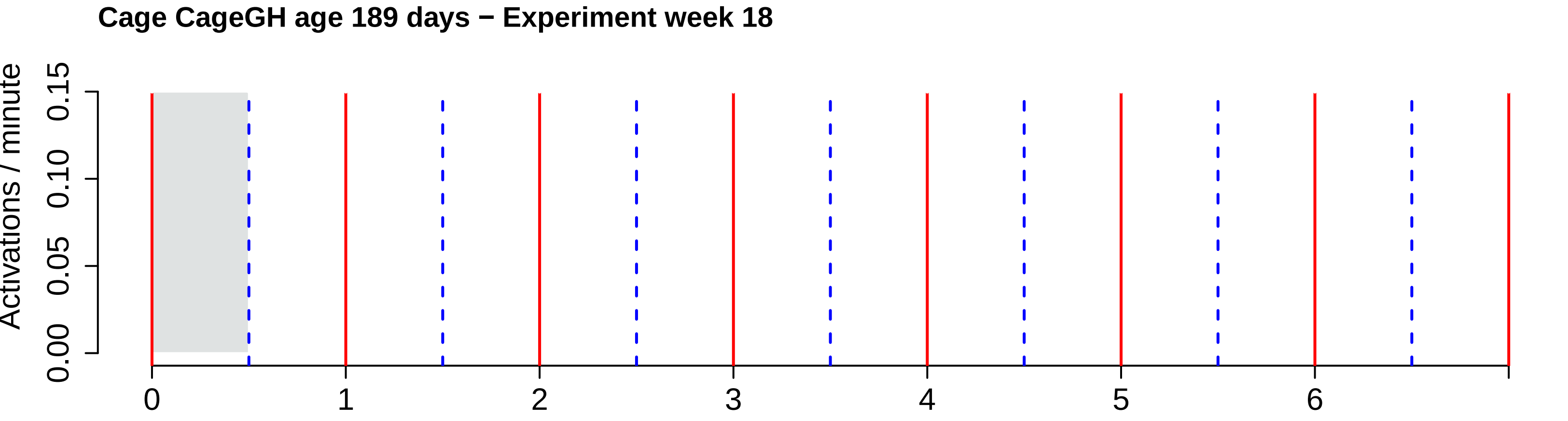

Cage CageGH age 189 days − Experiment week 18
Activations / minute
0.00
0.05
0.10
0.15
0 1 2 3 4 5 6

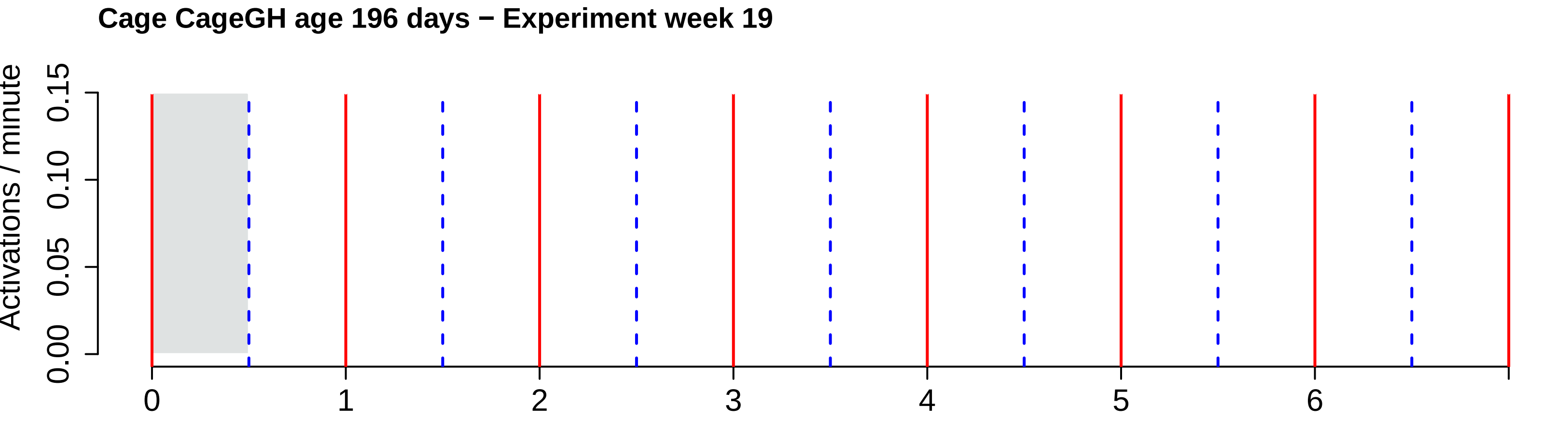

Cage CageGH age 196 days − Experiment week 19
Activations / minute
0.00
0.05
0.10
0.15
0 1 2 3 4 5 6

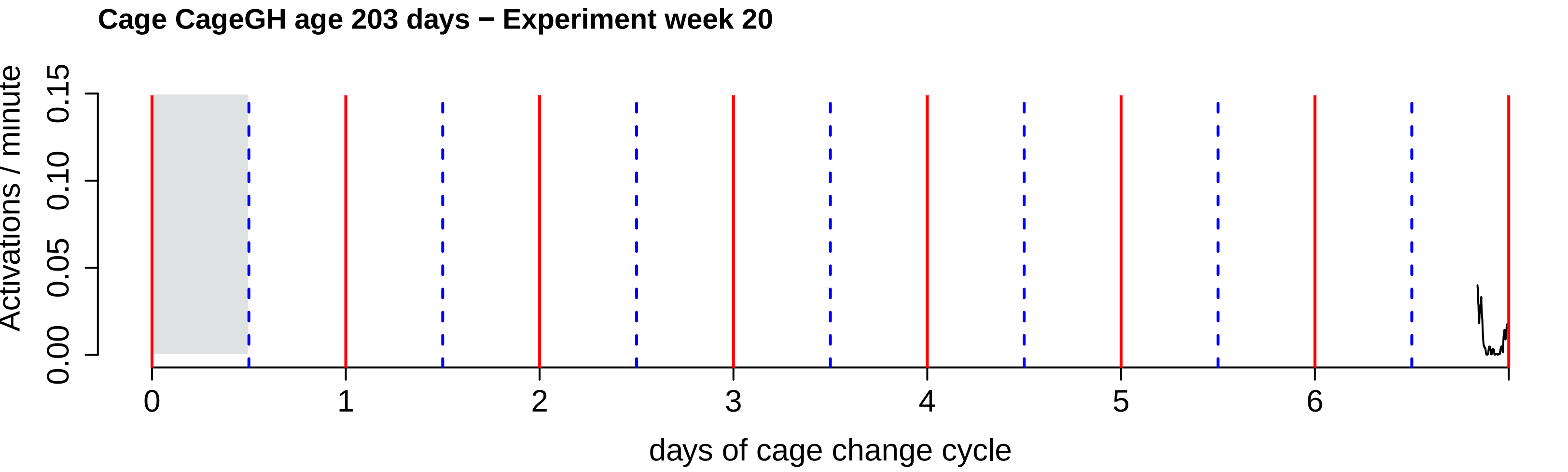

Cage CageGH age 203 days − Experiment week 20
Activations / minute
0.00
0.05
0.10
0.15
0 1 2 3 4 5 6
days of cage change cycle

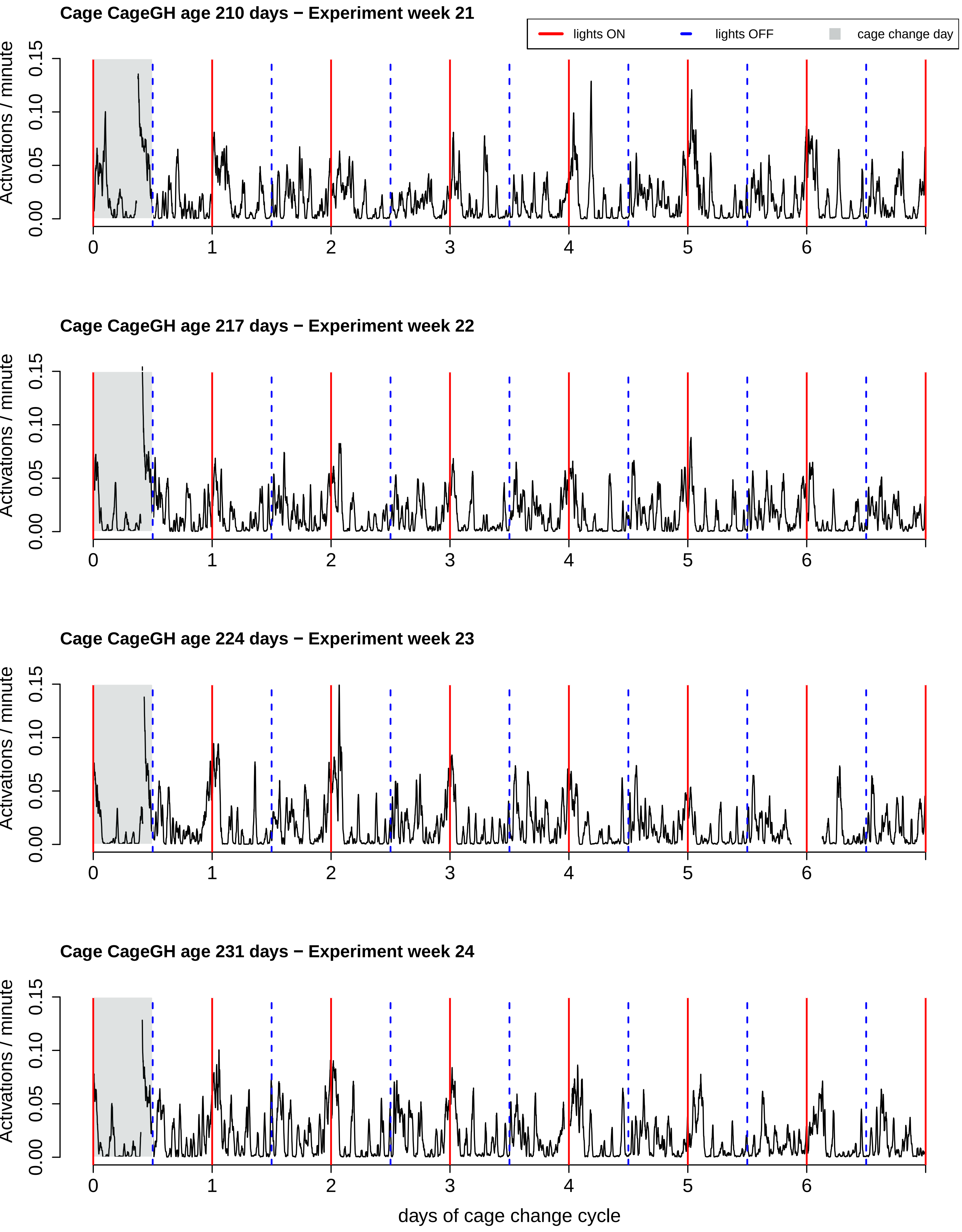

Cage CageGH age 210 days − Experiment week 21
lights ON
lights OFF
cage change day
Activations / minute
0.00
0.05
0.10
0.15
0
1
2
3
4
5
6
Cage CageGH age 217 days − Experiment week 22
Cage CageGH age 224 days − Experiment week 23
Cage CageGH age 231 days − Experiment week 24
days of cage change cycle

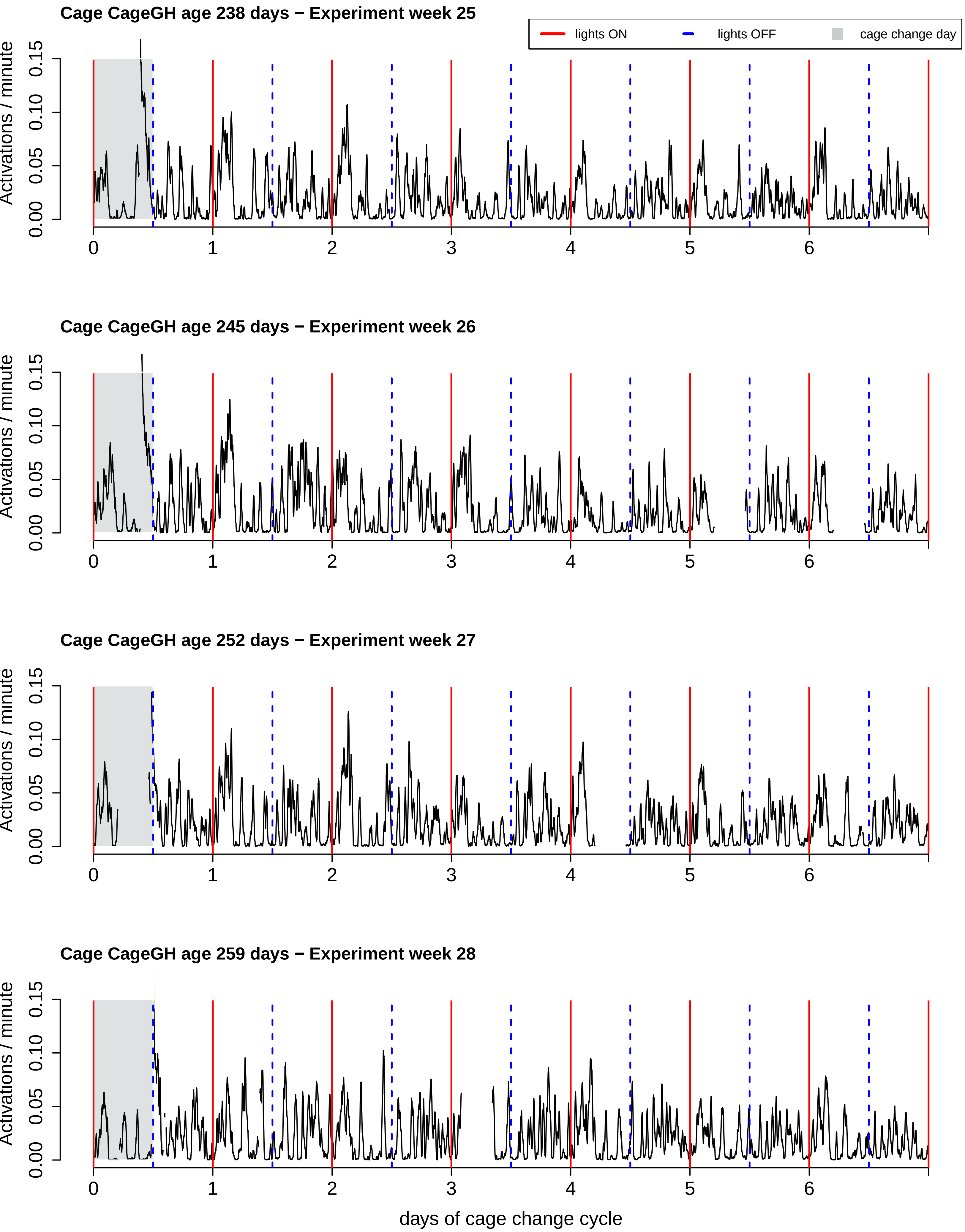
Cage CageGH age 238 days − Experiment week 25
lights ON
lights OFF
cage change day
Activations / minute
0.00
0.05
0.10
0.15
0
1
2
3
4
5
6
Cage CageGH age 245 days − Experiment week 26
Cage CageGH age 252 days − Experiment week 27
Cage CageGH age 259 days − Experiment week 28
days of cage change cycle

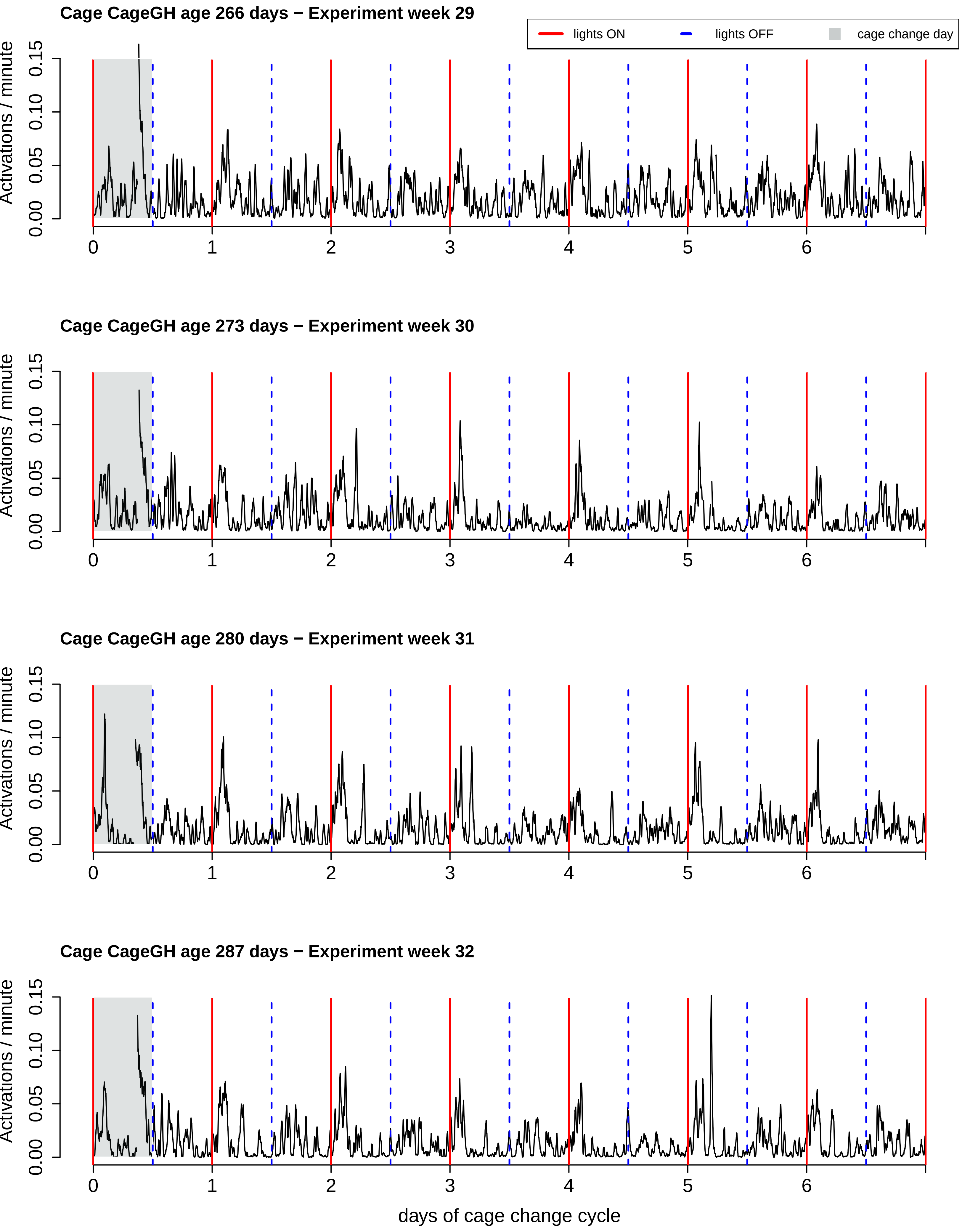

Cage CageGH age 266 days − Experiment week 29
lights ON
lights OFF
cage change day
Cage CageGH age 273 days − Experiment week 30
Cage CageGH age 280 days − Experiment week 31
Cage CageGH age 287 days − Experiment week 32
Activations / minute
0.00
0.05
0.10
0.15
0
1
2
3
4
5
6
days of cage change cycle

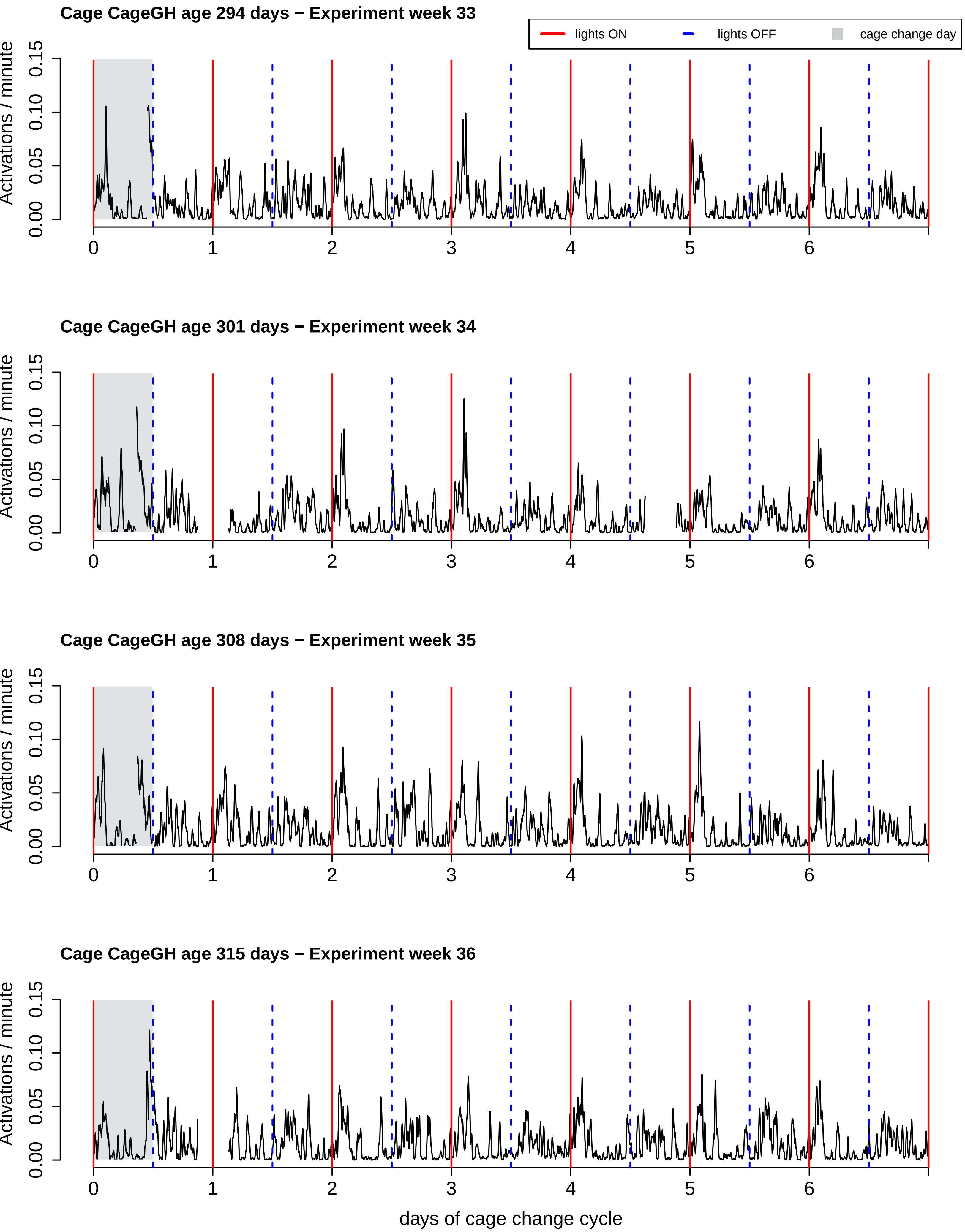
Cage CageGH age 294 days − Experiment week 33
lights ON
lights OFF
cage change day
Activations / minute
0.00
0.05
0.10
0.15
0
1
2
3
4
5
6
Cage CageGH age 301 days − Experiment week 34
Cage CageGH age 308 days − Experiment week 35
Cage CageGH age 315 days − Experiment week 36
days of cage change cycle

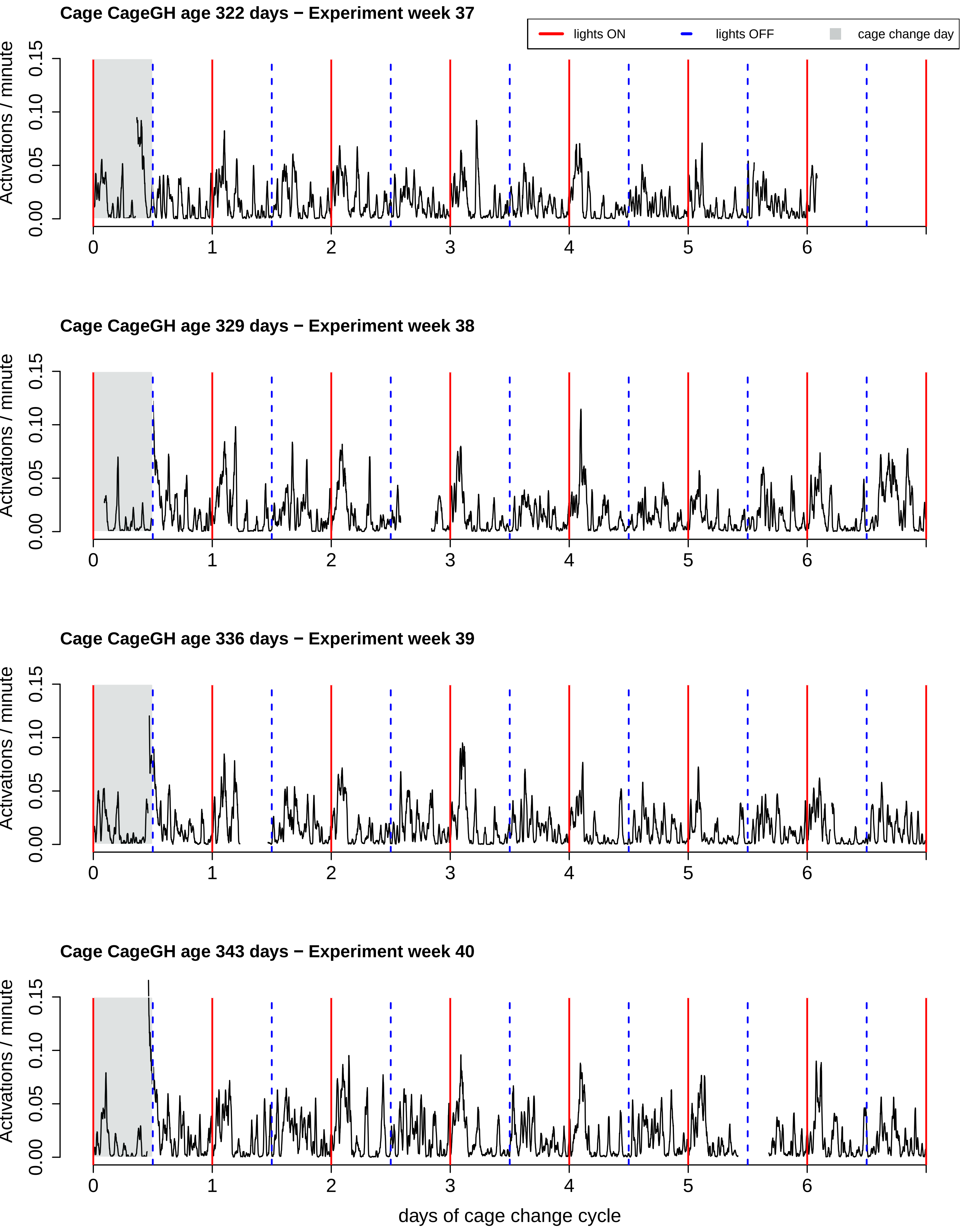
Cage CageGH age 322 days − Experiment week 37
lights ON
lights OFF
cage change day
Activations / minute
0.00
0.05
0.10
0.15
0
1
2
3
4
5
6
Cage CageGH age 329 days − Experiment week 38
Activations / minute
0.00
0.05
0.10
0.15
0
1
2
3
4
5
6
Cage CageGH age 336 days − Experiment week 39
Activations / minute
0.00
0.05
0.10
0.15
0
1
2
3
4
5
6
Cage CageGH age 343 days − Experiment week 40
Activations / minute
0.00
0.05
0.10
0.15
0
1
2
3
4
5
6
days of cage change cycle

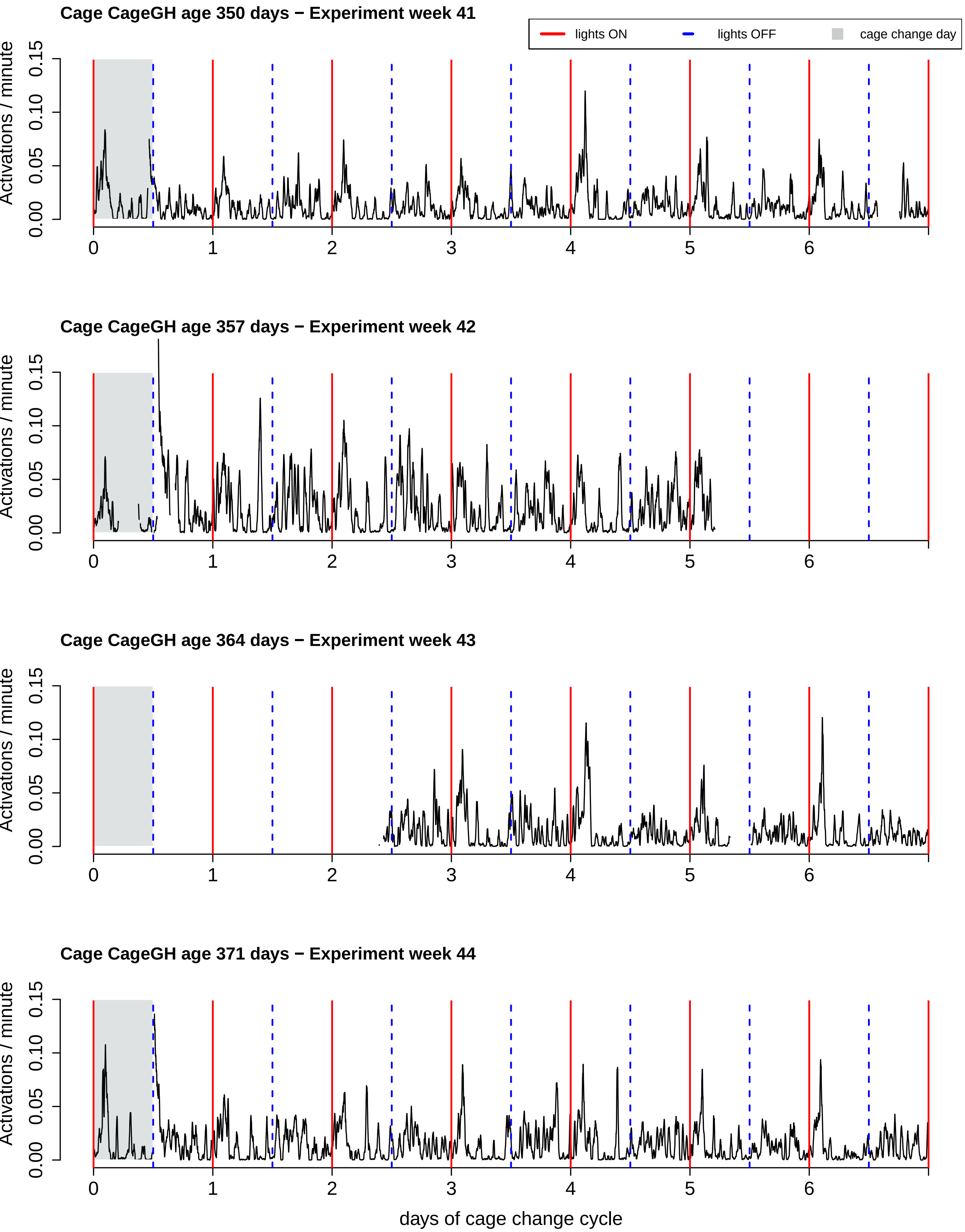
Cage CageGH age 350 days − Experiment week 41
lights ON
lights OFF
cage change day
Activations / minute
0.00
0.05
0.10
0.15
0
1
2
3
4
5
6
Cage CageGH age 357 days − Experiment week 42
Cage CageGH age 364 days − Experiment week 43
Cage CageGH age 371 days − Experiment week 44
days of cage change cycle

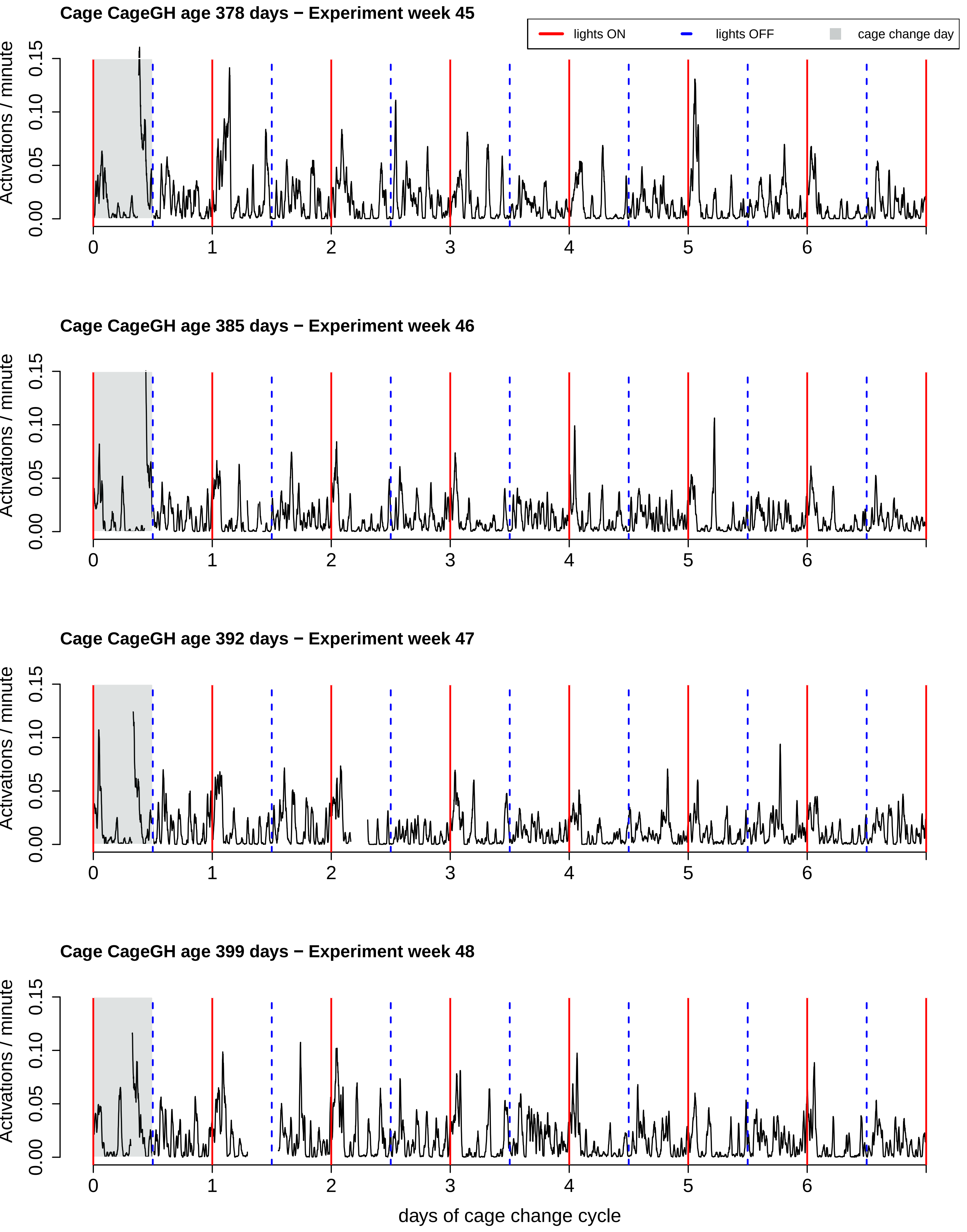
Cage CageGH age 378 days − Experiment week 45
lights ON
lights OFF
cage change day
Activations / minute
Cage CageGH age 385 days − Experiment week 46
Cage CageGH age 392 days − Experiment week 47
Cage CageGH age 399 days − Experiment week 48
days of cage change cycle

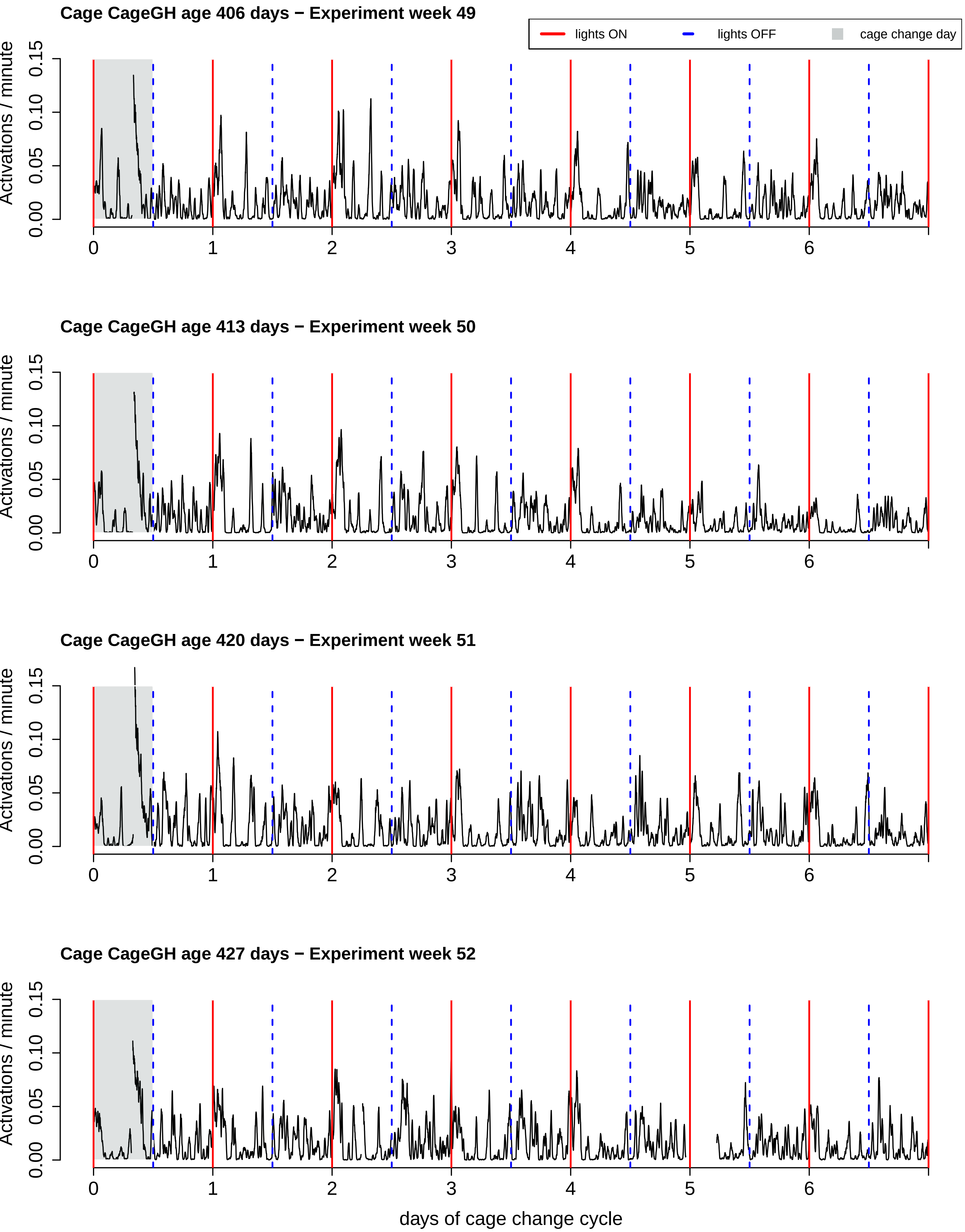

Cage CageGH age 406 days − Experiment week 49
lights ON
lights OFF
cage change day
Activations / minute
0.00
0.05
0.10
0.15
0
1
2
3
4
5
6
Cage CageGH age 413 days − Experiment week 50
Cage CageGH age 420 days − Experiment week 51
Cage CageGH age 427 days − Experiment week 52
days of cage change cycle

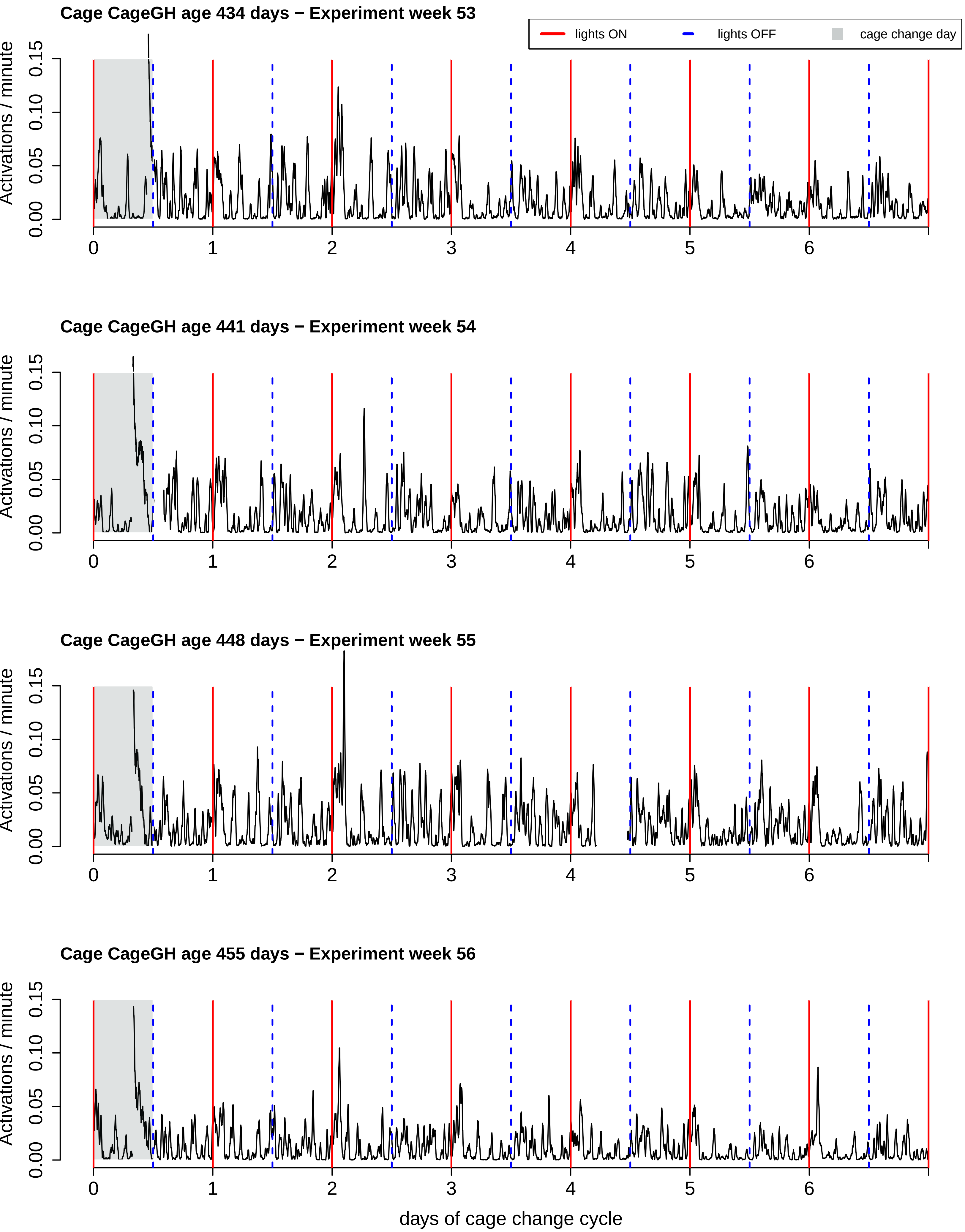

Cage CageGH age 434 days − Experiment week 53
lights ON
lights OFF
cage change day
Activations / minute
0.00
0.05
0.10
0.15
0
1
2
3
4
5
6
Cage CageGH age 441 days − Experiment week 54
Cage CageGH age 448 days − Experiment week 55
Cage CageGH age 455 days − Experiment week 56
days of cage change cycle

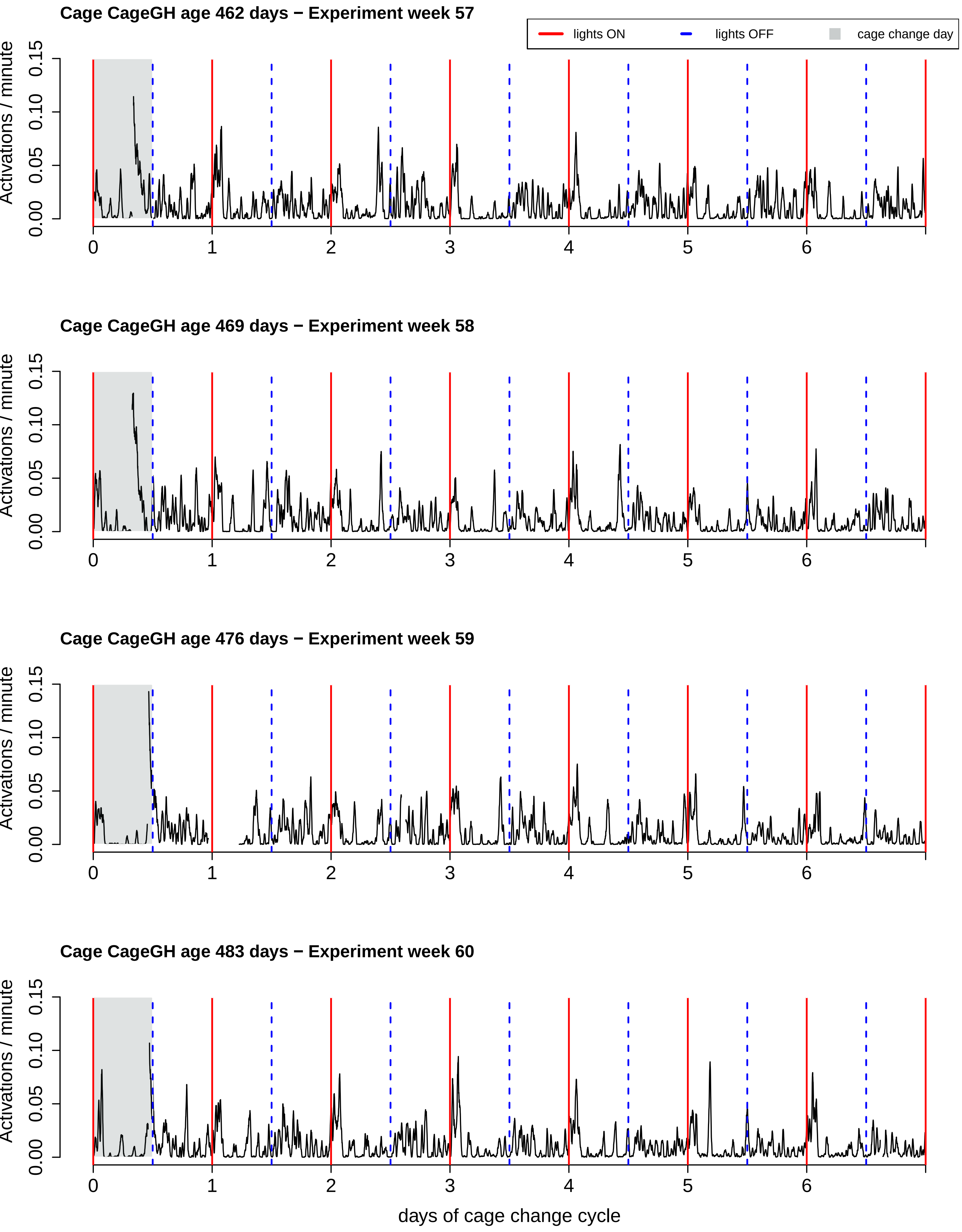
Cage CageGH age 462 days − Experiment week 57
lights ON
lights OFF
cage change day
Activations / minute
0.00
0.05
0.10
0.15
0
1
2
3
4
5
6
Cage CageGH age 469 days − Experiment week 58
Cage CageGH age 476 days − Experiment week 59
Cage CageGH age 483 days − Experiment week 60
days of cage change cycle

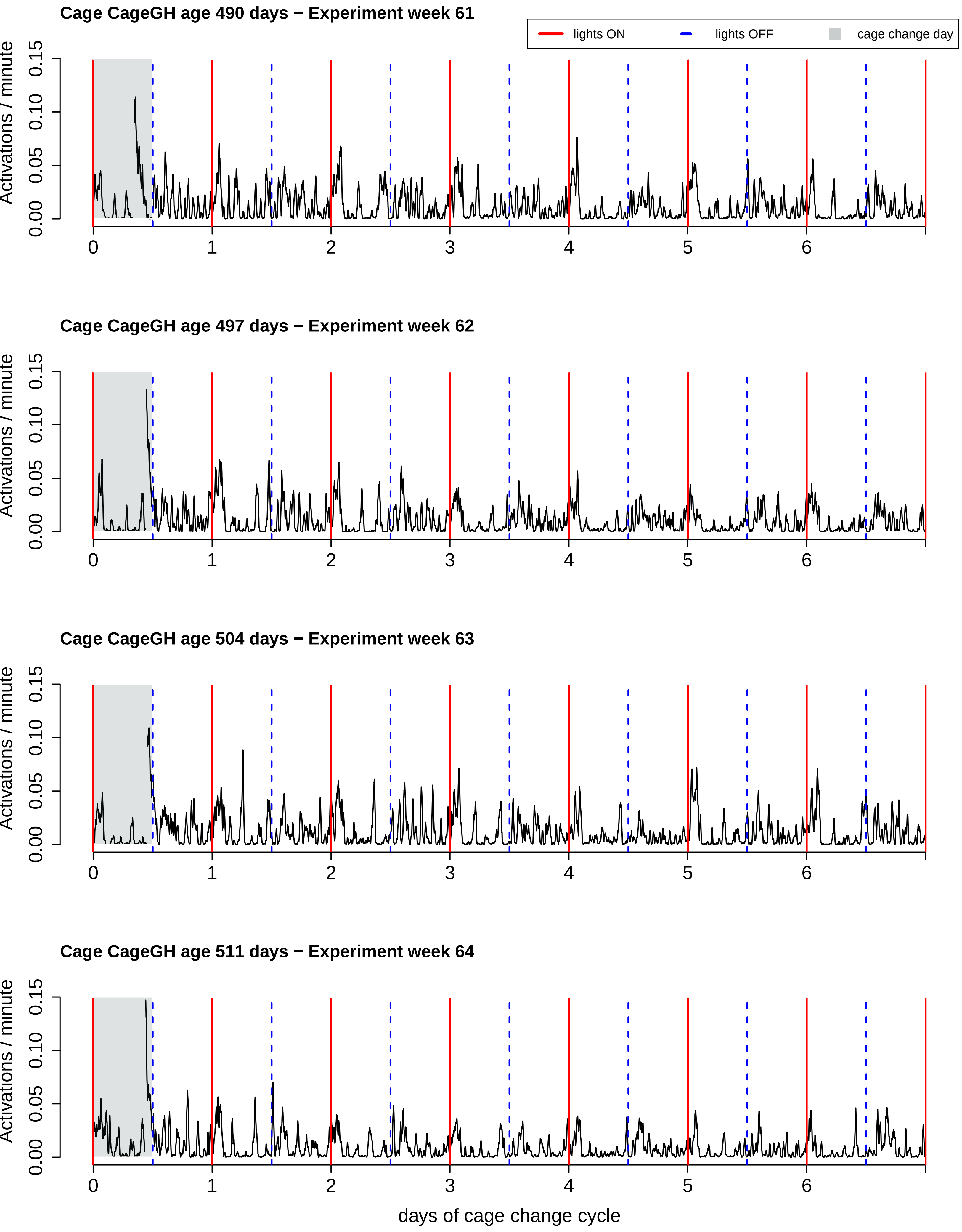
Cage CageGH age 490 days − Experiment week 61
lights ON
lights OFF
cage change day
Activations / minute
0.00
0.05
0.10
0.15
0
1
2
3
4
5
6
Cage CageGH age 497 days − Experiment week 62
Cage CageGH age 504 days − Experiment week 63
Cage CageGH age 511 days − Experiment week 64
days of cage change cycle

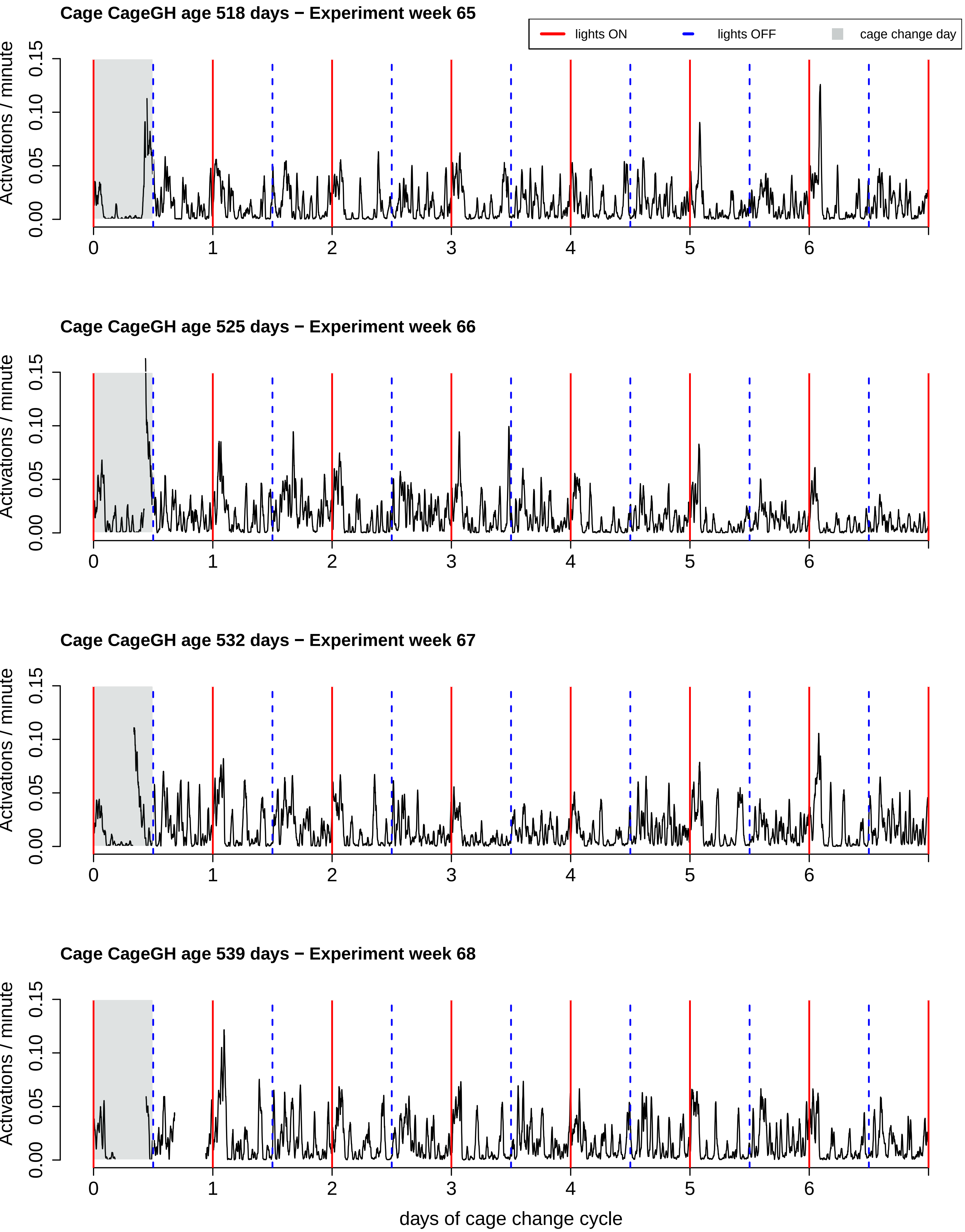

Cage CageGH age 518 days − Experiment week 65
lights ON
lights OFF
cage change day
Activations / minute
0.00
0.05
0.10
0.15
0
1
2
3
4
5
6
Cage CageGH age 525 days − Experiment week 66
Activations / minute
0.00
0.05
0.10
0.15
0
1
2
3
4
5
6
Cage CageGH age 532 days − Experiment week 67
Activations / minute
0.00
0.05
0.10
0.15
0
1
2
3
4
5
6
Cage CageGH age 539 days − Experiment week 68
Activations / minute
0.00
0.05
0.10
0.15
0
1
2
3
4
5
6
days of cage change cycle

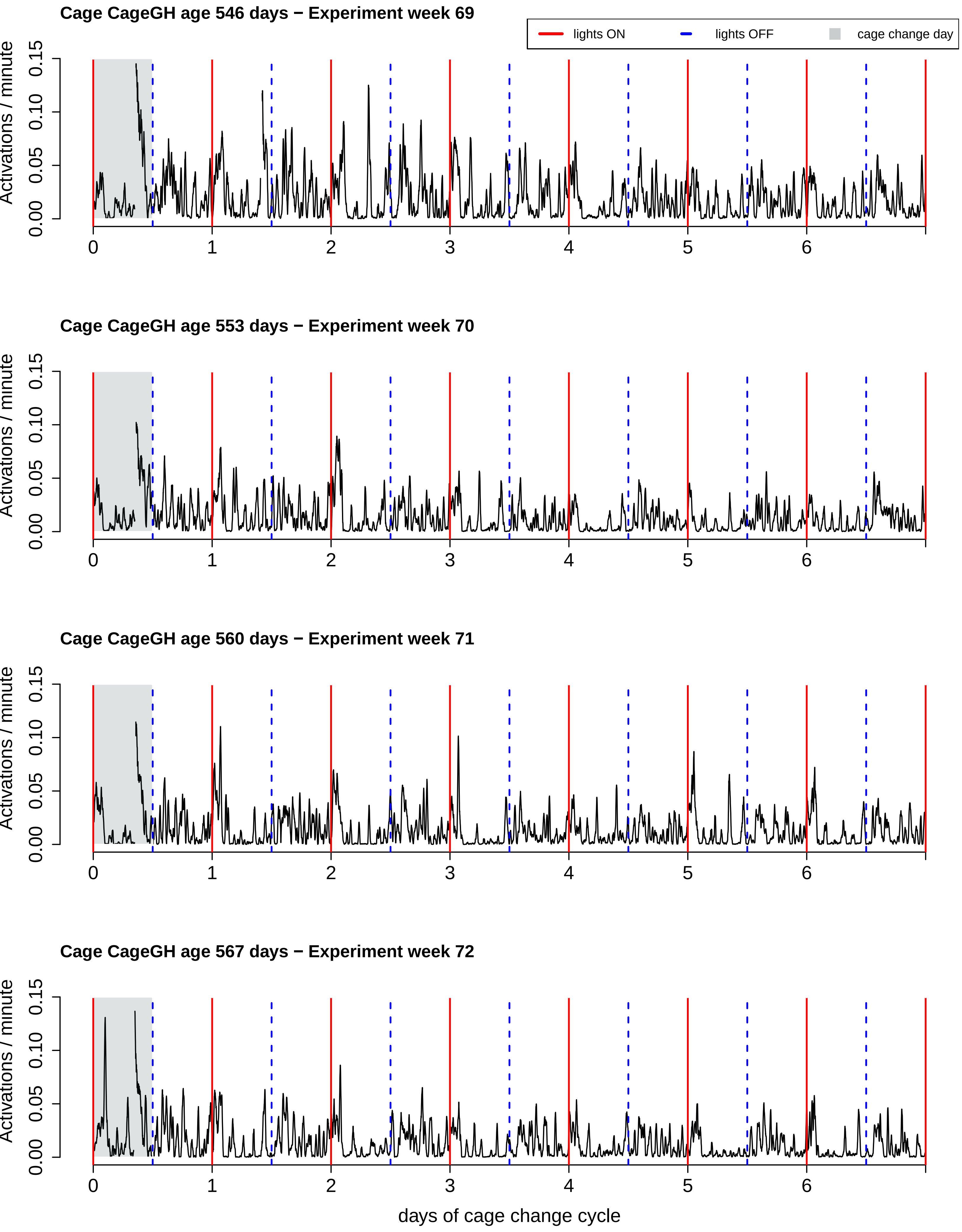

Cage CageGH age 546 days − Experiment week 69
lights ON
lights OFF
cage change day
Activations / minute
0.00
0.05
0.10
0.15
0
1
2
3
4
5
6
Cage CageGH age 553 days − Experiment week 70
Cage CageGH age 560 days − Experiment week 71
Cage CageGH age 567 days − Experiment week 72
days of cage change cycle

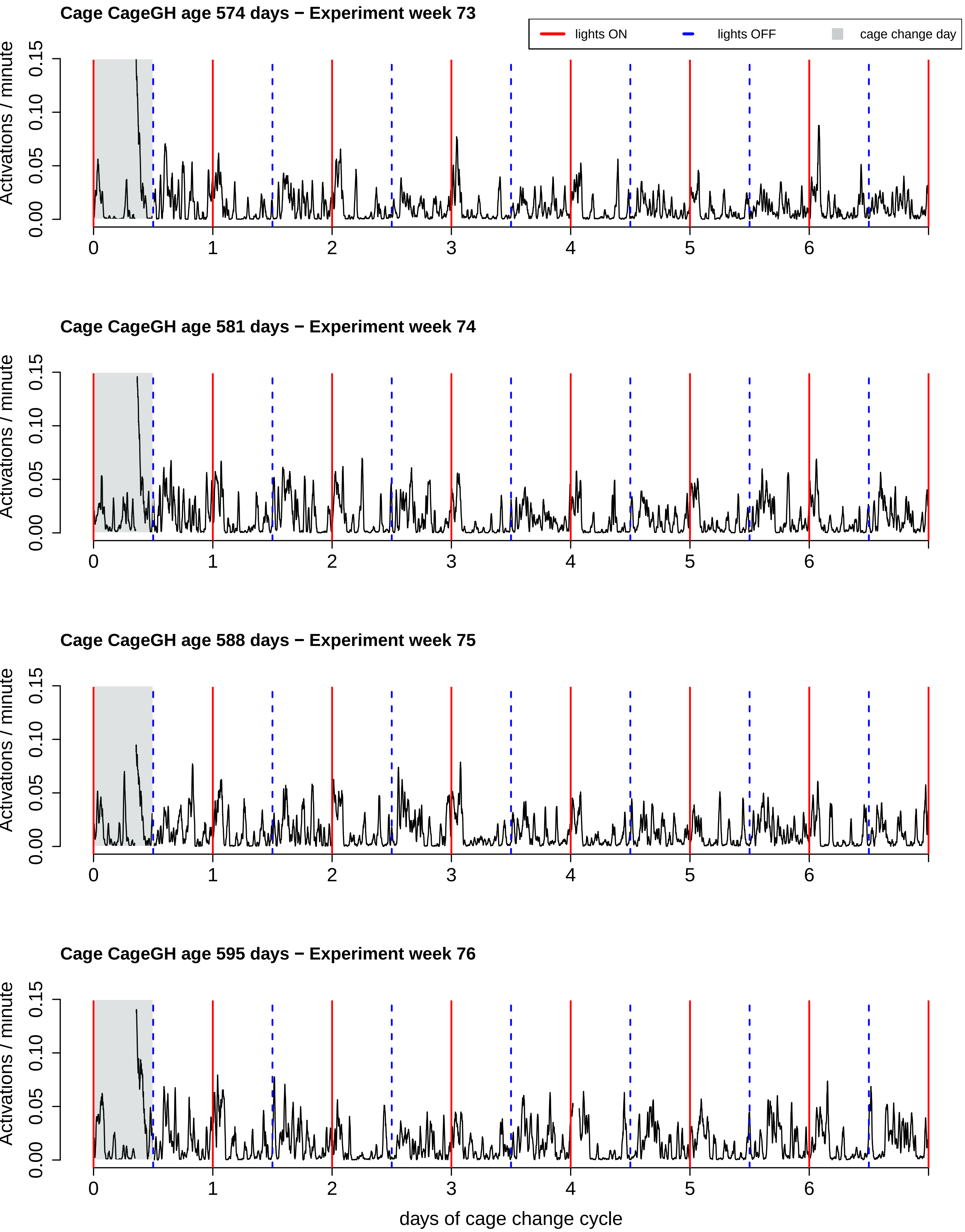
Cage CageGH age 574 days − Experiment week 73
lights ON
lights OFF
cage change day
Activations / minute
0.00
0.05
0.10
0.15
0
1
2
3
4
5
6
Cage CageGH age 581 days − Experiment week 74
Cage CageGH age 588 days − Experiment week 75
Cage CageGH age 595 days − Experiment week 76
days of cage change cycle

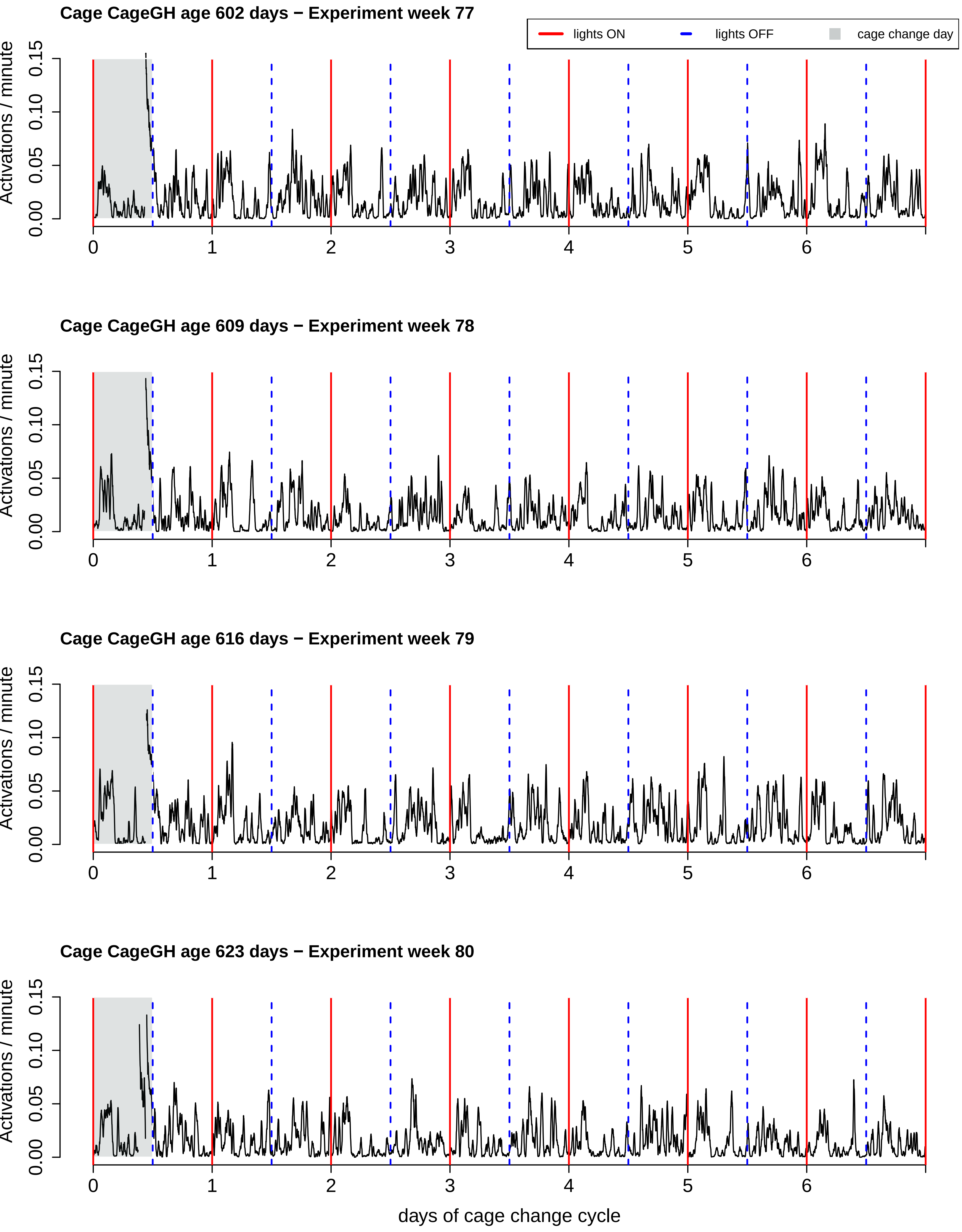

Cage CageGH age 602 days − Experiment week 77
lights ON
lights OFF
cage change day
Cage CageGH age 609 days − Experiment week 78
Cage CageGH age 616 days − Experiment week 79
Cage CageGH age 623 days − Experiment week 80
Activations / minute
0.00
0.05
0.10
0.15
0
1
2
3
4
5
6
days of cage change cycle

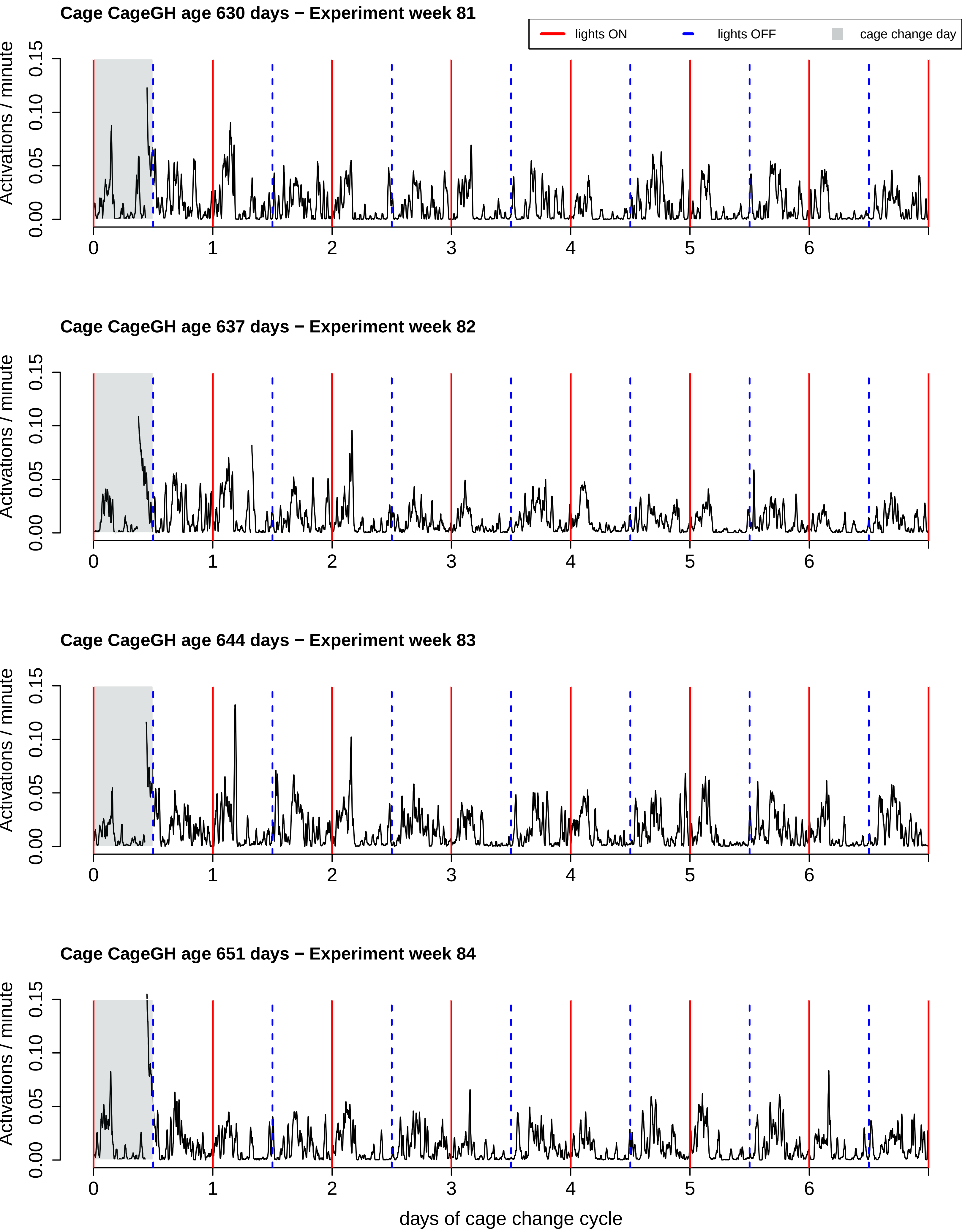
Cage CageGH age 630 days − Experiment week 81
lights ON
lights OFF
cage change day
Activations / minute
0.00
0.05
0.10
0.15
0
1
2
3
4
5
6
Cage CageGH age 637 days − Experiment week 82
Cage CageGH age 644 days − Experiment week 83
Cage CageGH age 651 days − Experiment week 84
days of cage change cycle

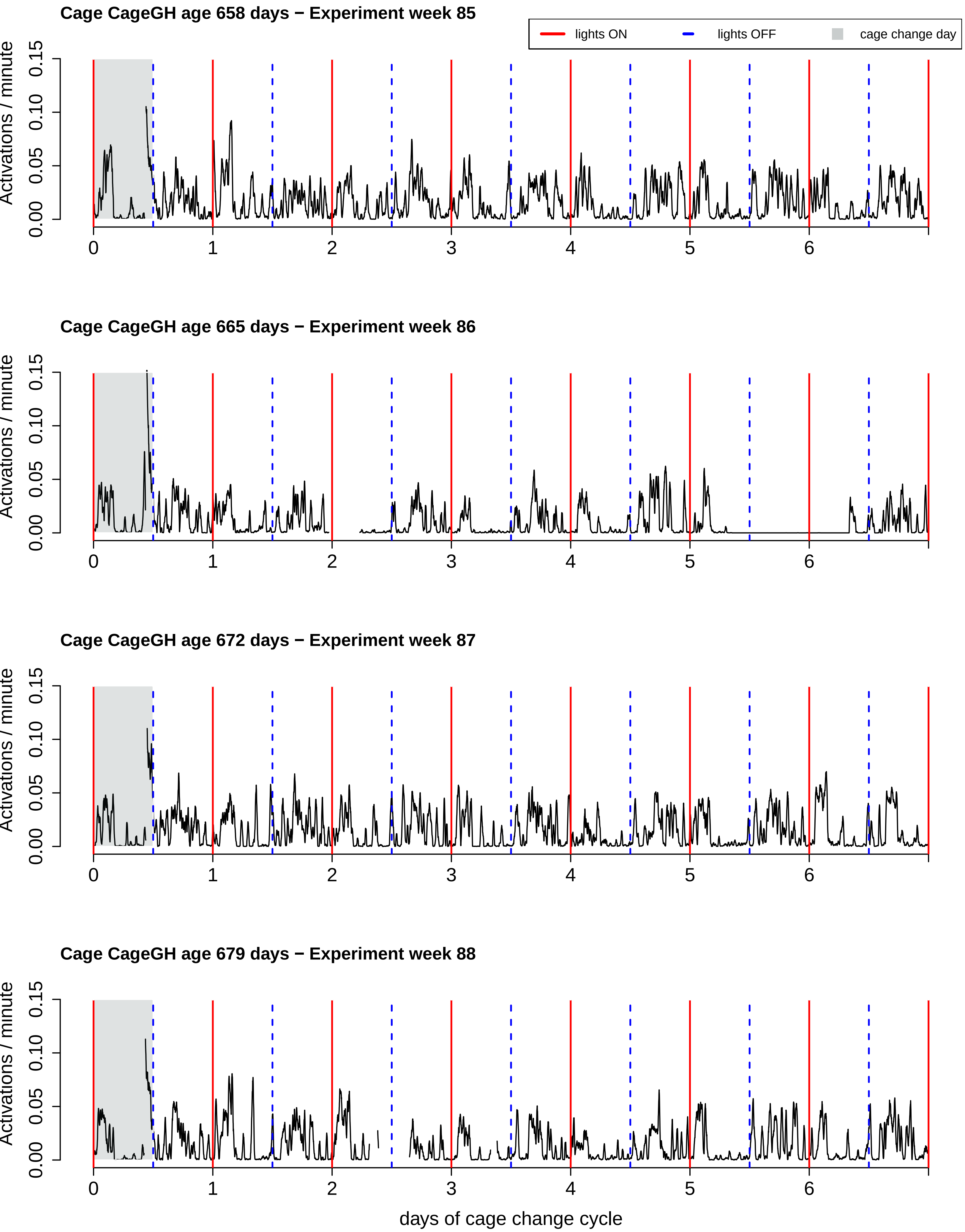
Cage CageGH age 658 days − Experiment week 85
lights ON
lights OFF
cage change day
Activations / minute
0.00
0.05
0.10
0.15
0
1
2
3
4
5
6
Cage CageGH age 665 days − Experiment week 86
Cage CageGH age 672 days − Experiment week 87
Cage CageGH age 679 days − Experiment week 88
days of cage change cycle

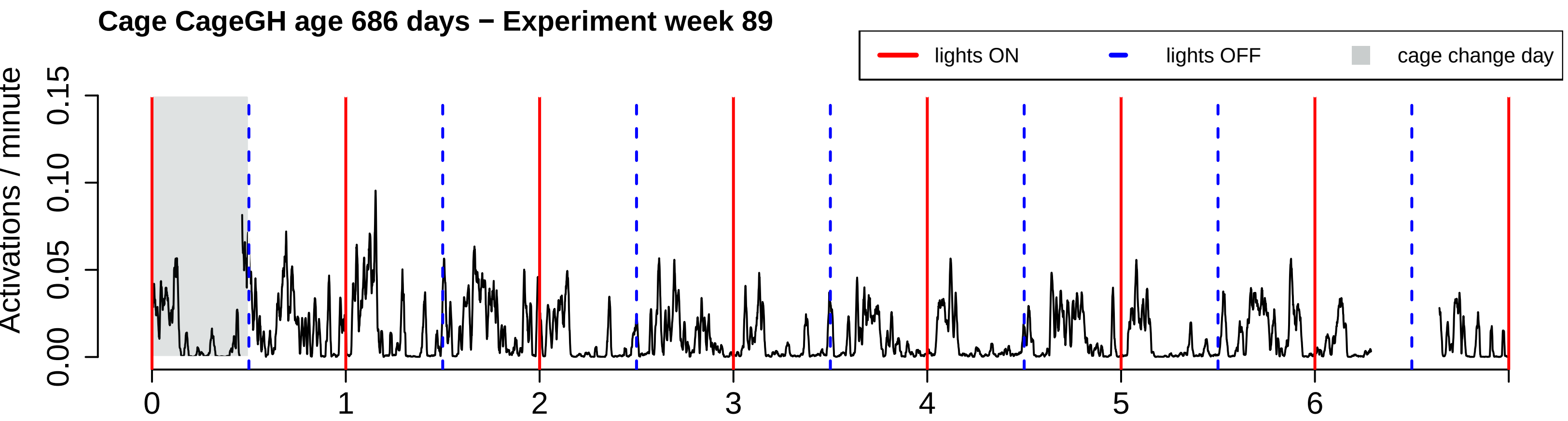

Cage CageGH age 686 days − Experiment week 89
lights ON
lights OFF
cage change day
Activations / minute
0.00
0.05
0.10
0.15
0
1
2
3
4
5
6

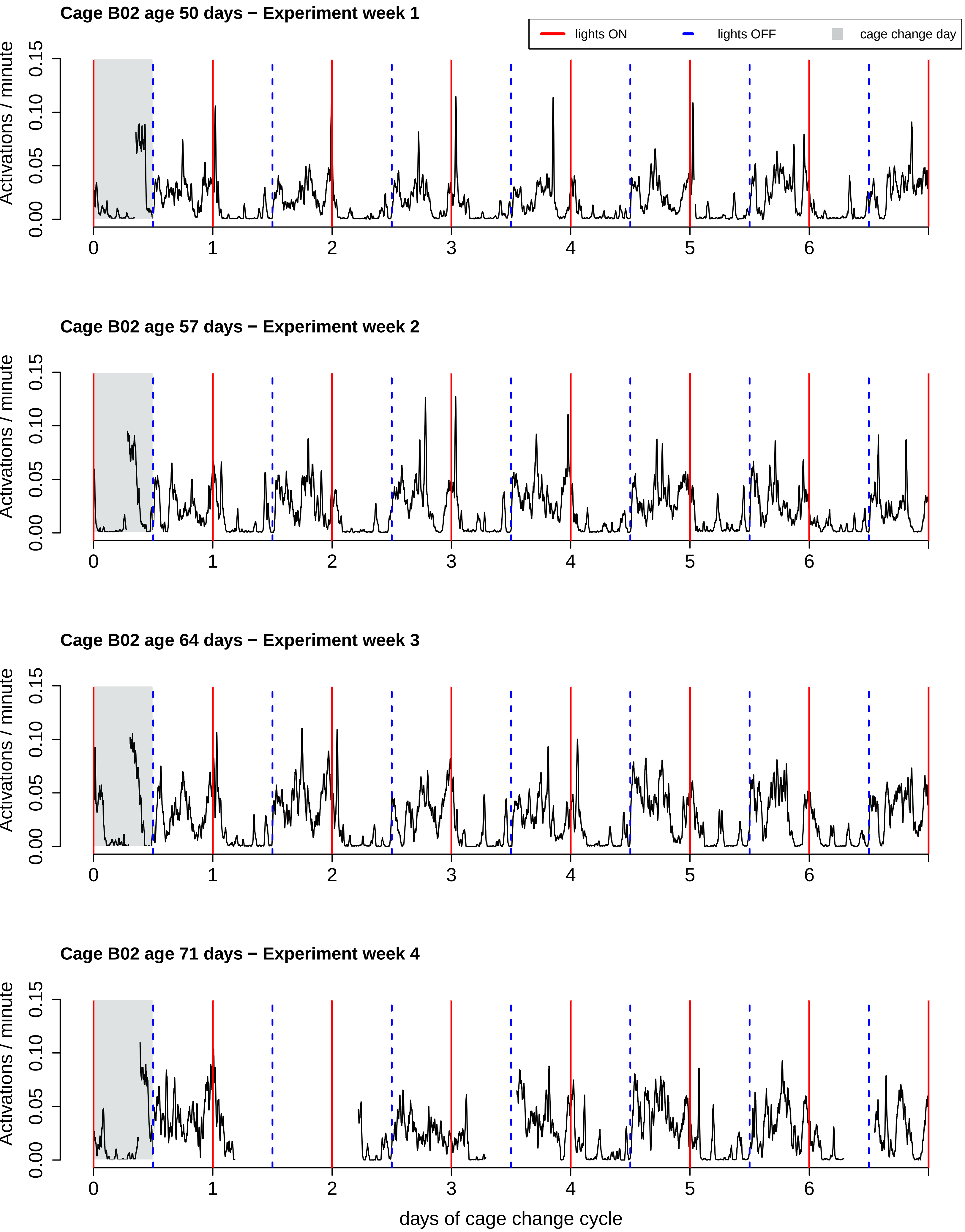

Cage B02 age 50 days − Experiment week 1
lights ON
lights OFF
cage change day
Activations / minute
0.00
0.05
0.10
0.15
0
1
2
3
4
5
6
Cage B02 age 57 days − Experiment week 2
Cage B02 age 64 days − Experiment week 3
Cage B02 age 71 days − Experiment week 4
days of cage change cycle

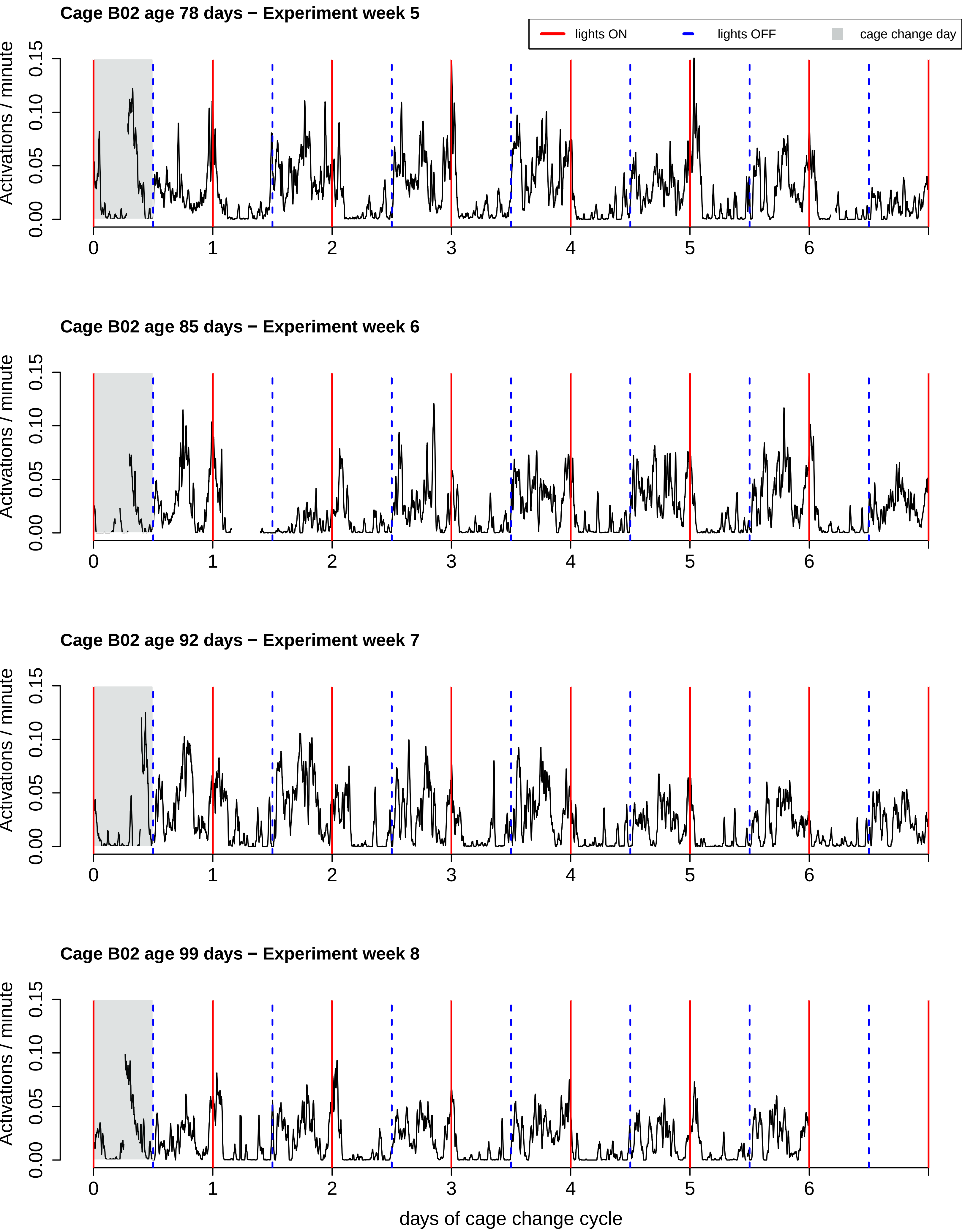
Cage B02 age 78 days − Experiment week 5
lights ON
lights OFF
cage change day
Cage B02 age 85 days − Experiment week 6
Cage B02 age 92 days − Experiment week 7
Cage B02 age 99 days − Experiment week 8
Activations / minute
0.00
0.05
0.10
0.15
0
1
2
3
4
5
6
days of cage change cycle

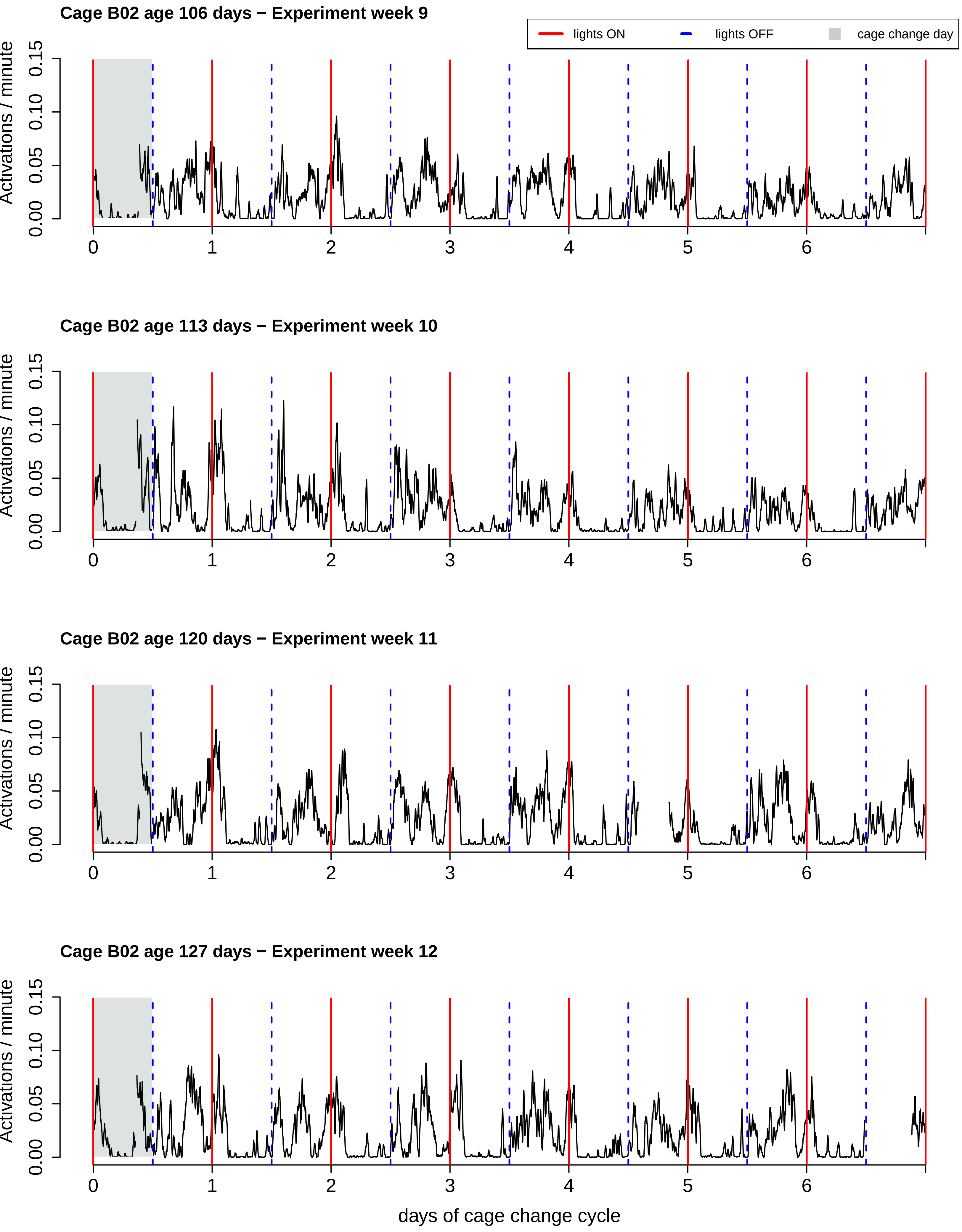
Cage B02 age 106 days − Experiment week 9
lights ON
lights OFF
cage change day
Activations / minute
0.00
0.05
0.10
0.15
0
1
2
3
4
5
6
Cage B02 age 113 days − Experiment week 10
Cage B02 age 120 days − Experiment week 11
Cage B02 age 127 days − Experiment week 12
days of cage change cycle

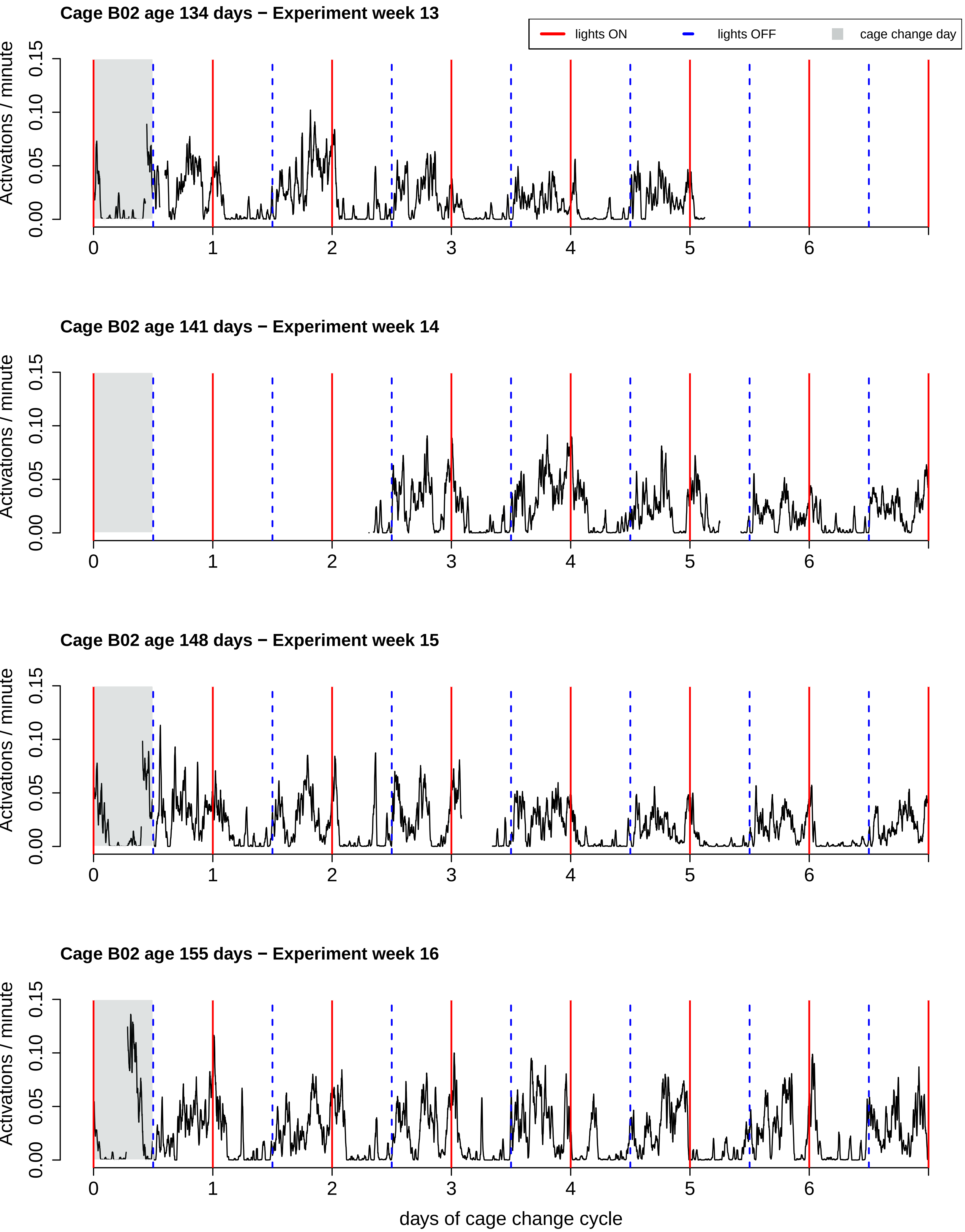
Cage B02 age 134 days − Experiment week 13
lights ON
lights OFF
cage change day
Activations / minute
0.00
0.05
0.10
0.15
0
1
2
3
4
5
6
Cage B02 age 141 days − Experiment week 14
Activations / minute
Cage B02 age 148 days − Experiment week 15
Activations / minute
Cage B02 age 155 days − Experiment week 16
Activations / minute
days of cage change cycle

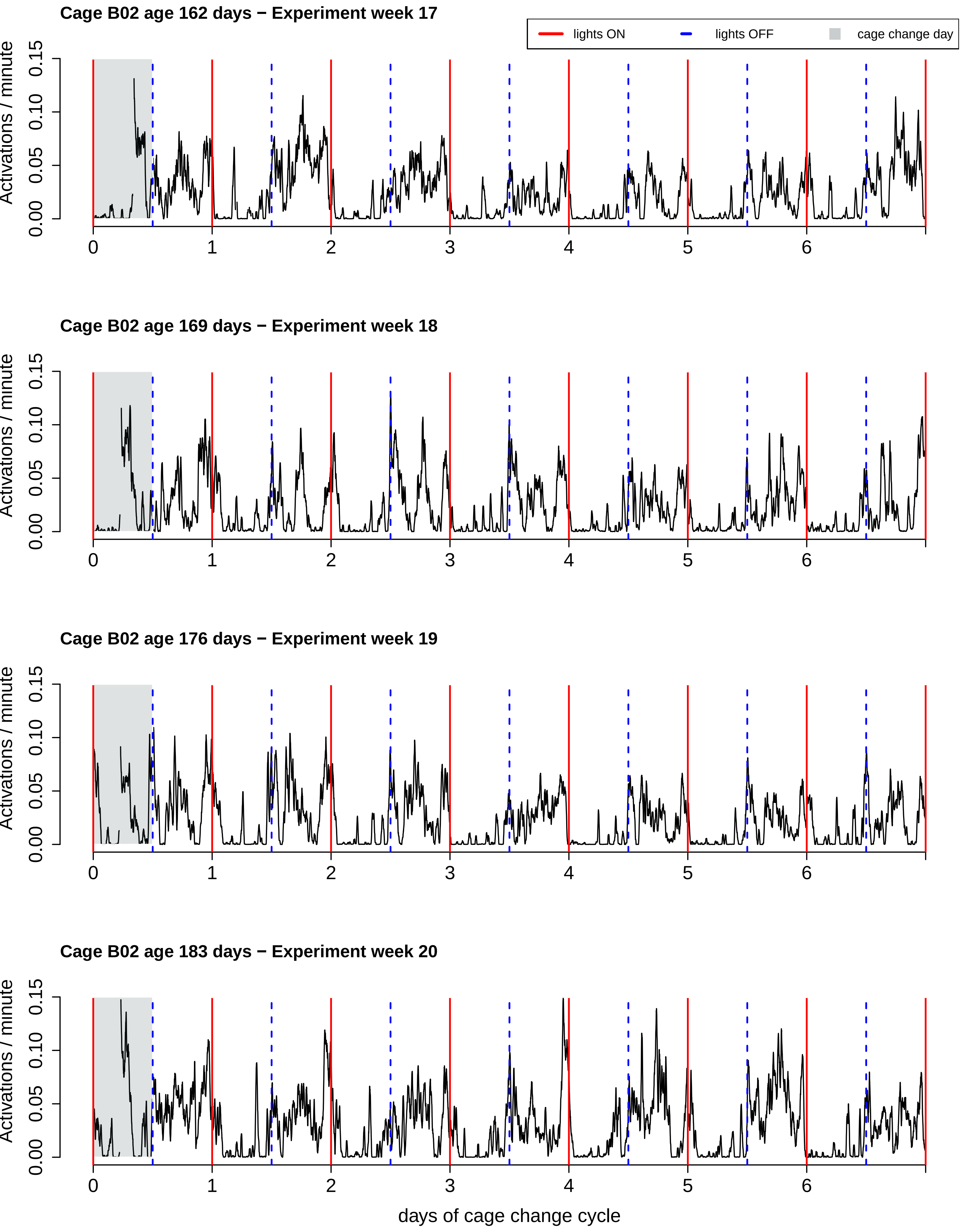

Cage B02 age 162 days − Experiment week 17
lights ON
lights OFF
cage change day
Activations / minute
0.00
0.05
0.10
0.15
0
1
2
3
4
5
6
Cage B02 age 169 days − Experiment week 18
Cage B02 age 176 days − Experiment week 19
Cage B02 age 183 days − Experiment week 20
days of cage change cycle

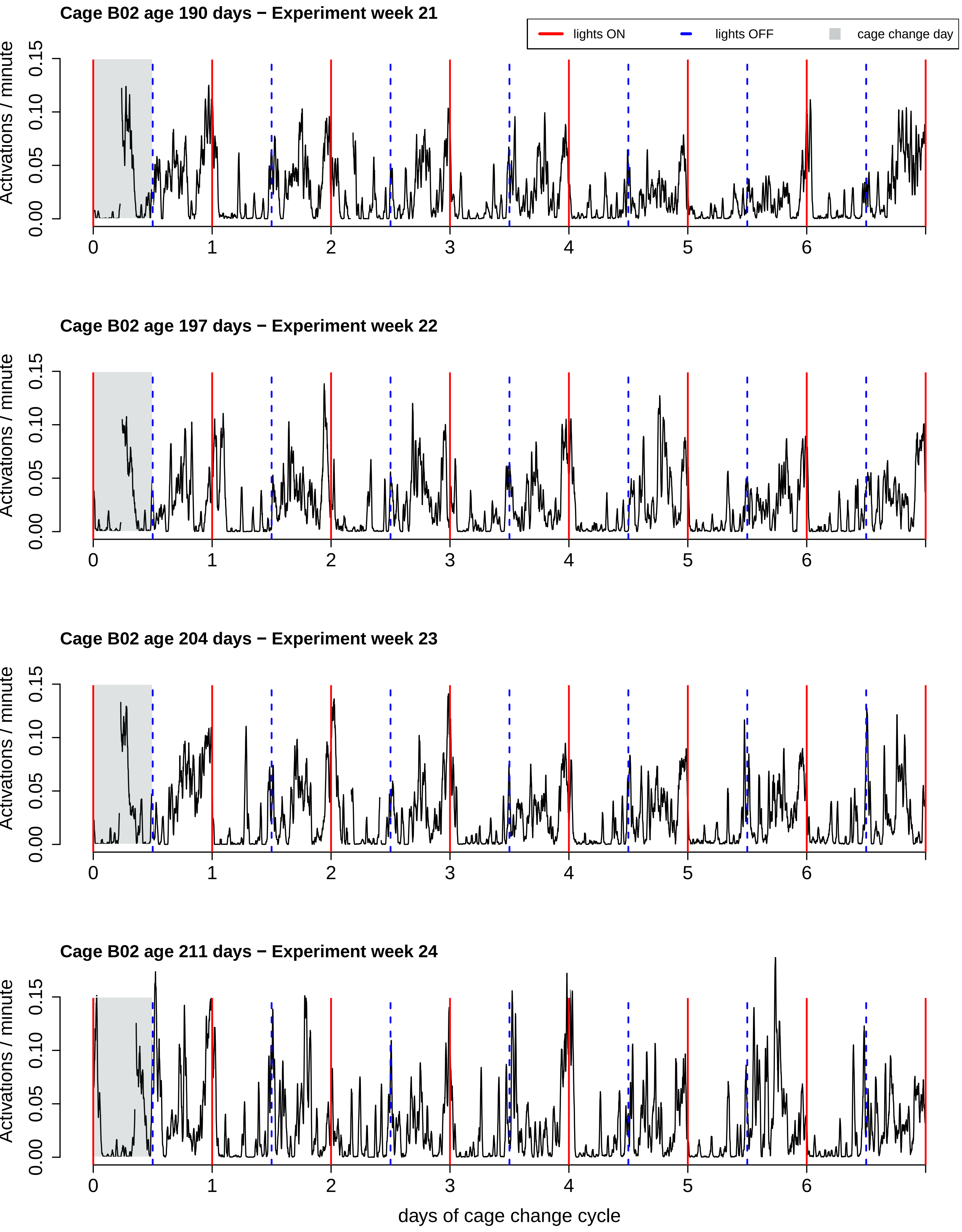

Cage B02 age 190 days − Experiment week 21
lights ON
lights OFF
cage change day
Activations / minute
0.00
0.05
0.10
0.15
0
1
2
3
4
5
6
Cage B02 age 197 days − Experiment week 22
Cage B02 age 204 days − Experiment week 23
Cage B02 age 211 days − Experiment week 24
days of cage change cycle

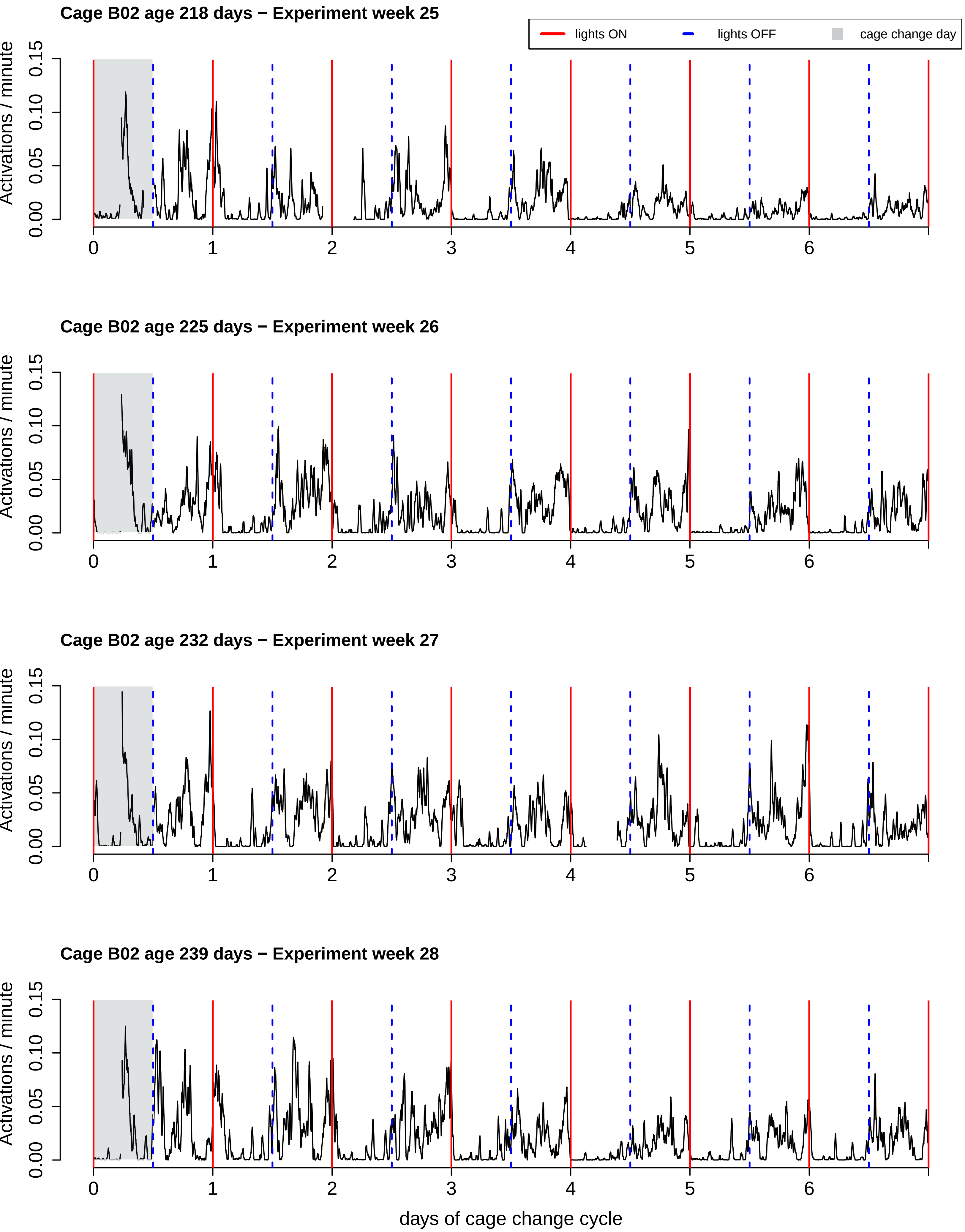
Cage B02 age 218 days − Experiment week 25
lights ON
lights OFF
cage change day
Activations / minute
0.00
0.05
0.10
0.15
0
1
2
3
4
5
6
Cage B02 age 225 days − Experiment week 26
Cage B02 age 232 days − Experiment week 27
Cage B02 age 239 days − Experiment week 28
days of cage change cycle

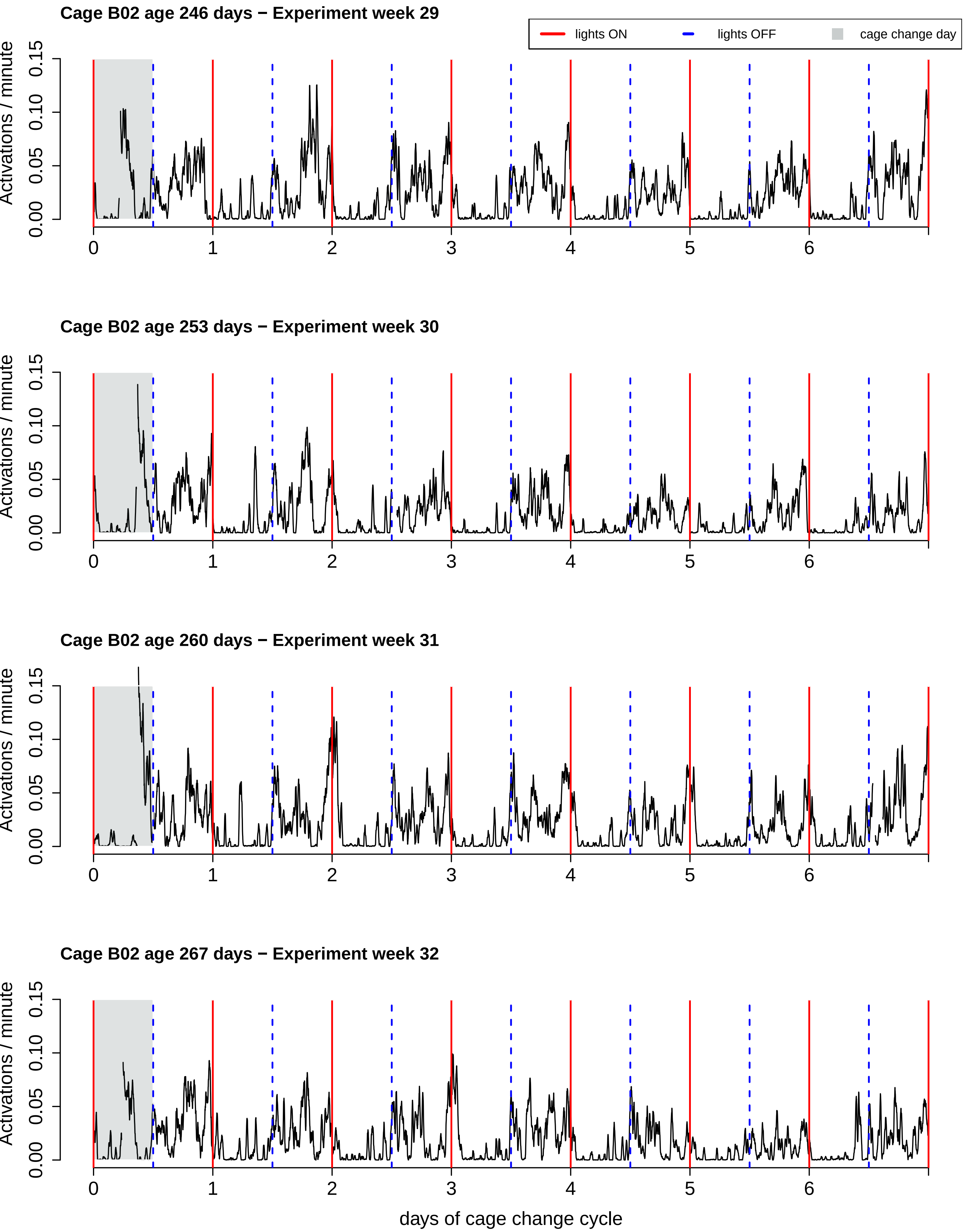
Cage B02 age 246 days − Experiment week 29
lights ON
lights OFF
cage change day
Cage B02 age 253 days − Experiment week 30
Cage B02 age 260 days − Experiment week 31
Cage B02 age 267 days − Experiment week 32
Activations / minute
0.00
0.05
0.10
0.15
0
1
2
3
4
5
6
days of cage change cycle

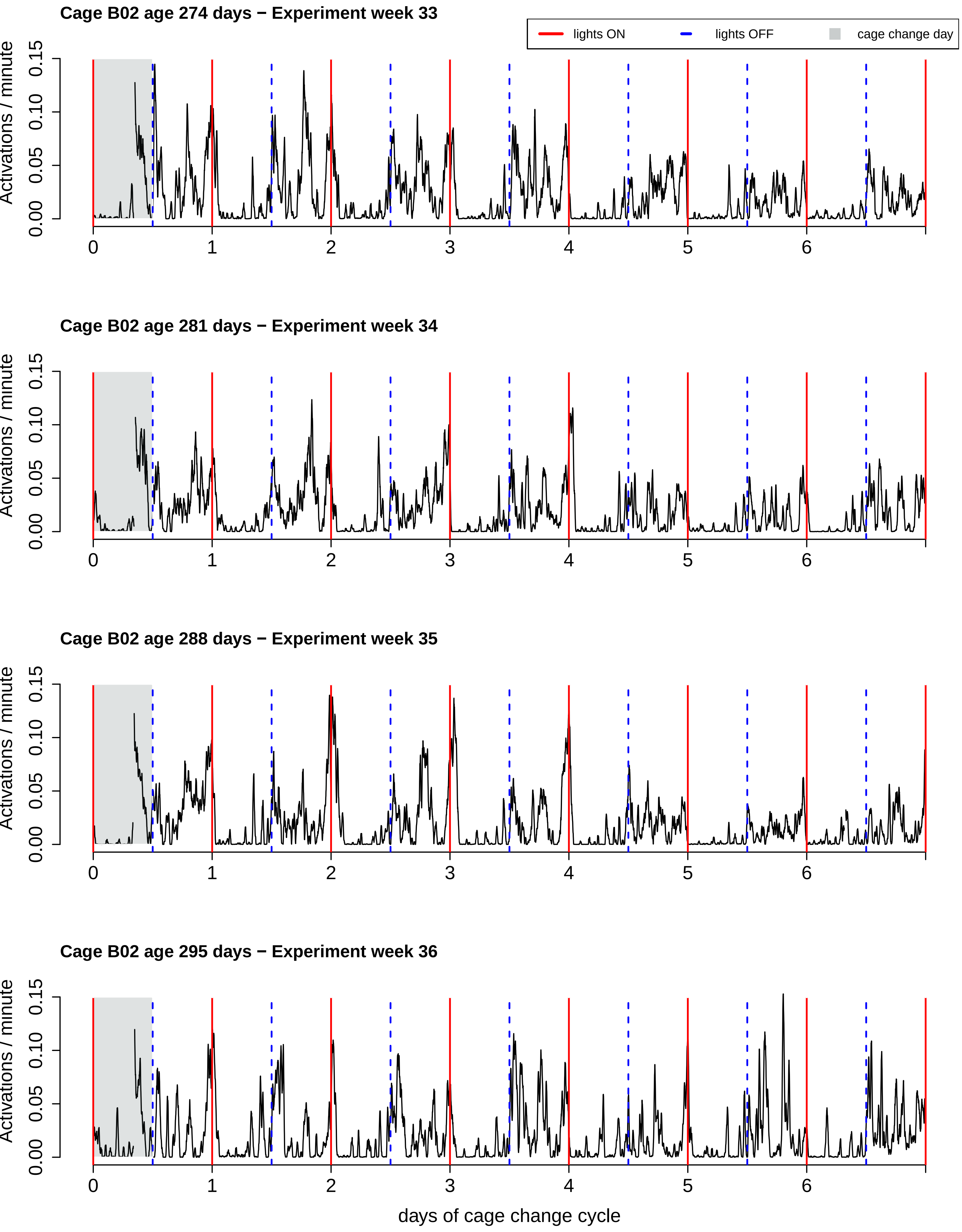

Cage B02 age 274 days − Experiment week 33
lights ON
lights OFF
cage change day
Activations / minute
0.00
0.05
0.10
0.15
0
1
2
3
4
5
6
Cage B02 age 281 days − Experiment week 34
Cage B02 age 288 days − Experiment week 35
Cage B02 age 295 days − Experiment week 36
days of cage change cycle

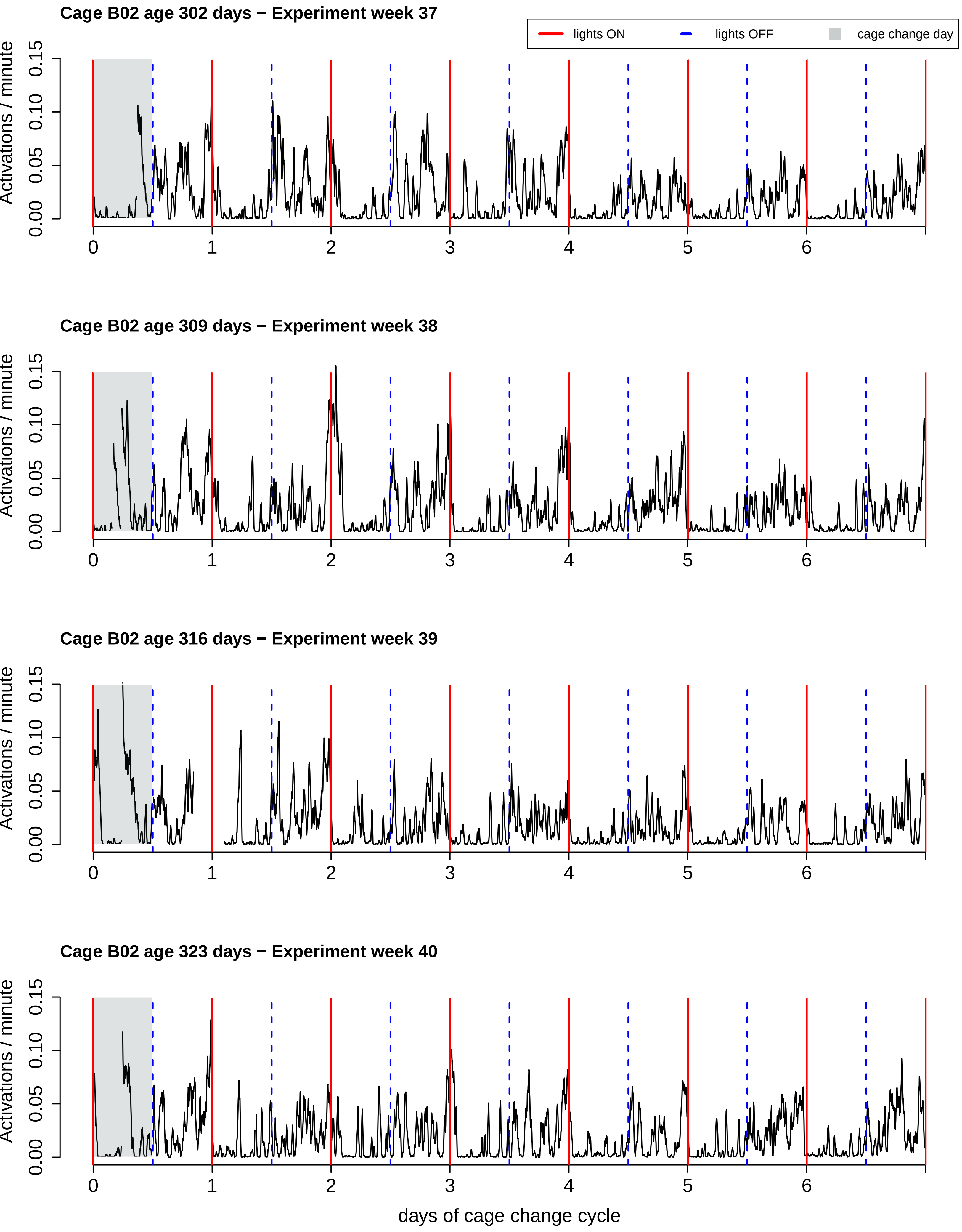

Cage B02 age 302 days − Experiment week 37
lights ON
lights OFF
cage change day
Activations / minute
0.00
0.05
0.10
0.15
0
1
2
3
4
5
6
Cage B02 age 309 days − Experiment week 38
Cage B02 age 316 days − Experiment week 39
Cage B02 age 323 days − Experiment week 40
days of cage change cycle

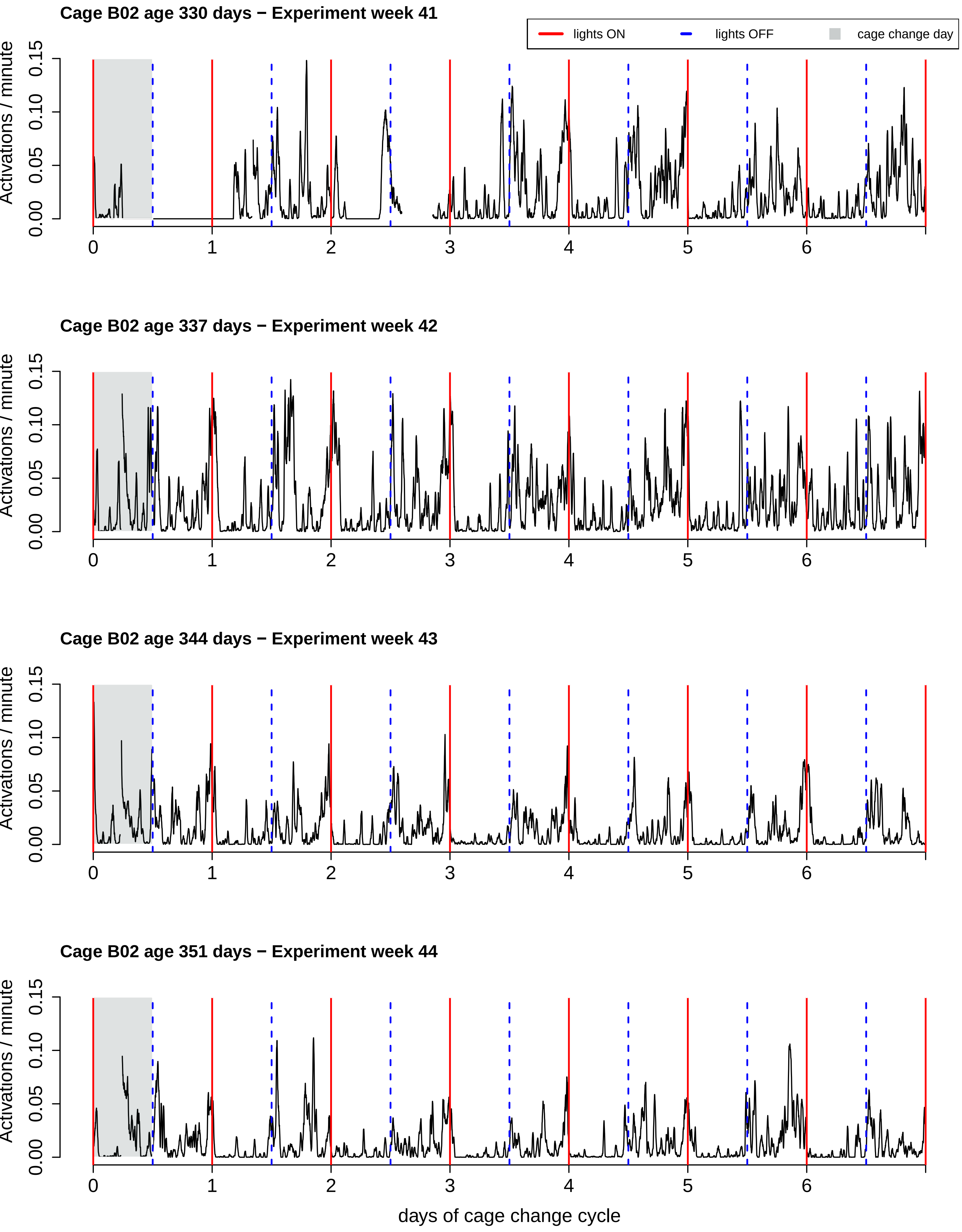
Cage B02 age 330 days − Experiment week 41
lights ON
lights OFF
cage change day
Activations / minute
0.00
0.05
0.10
0.15
0
1
2
3
4
5
6
Cage B02 age 337 days − Experiment week 42
Cage B02 age 344 days − Experiment week 43
Cage B02 age 351 days − Experiment week 44
days of cage change cycle

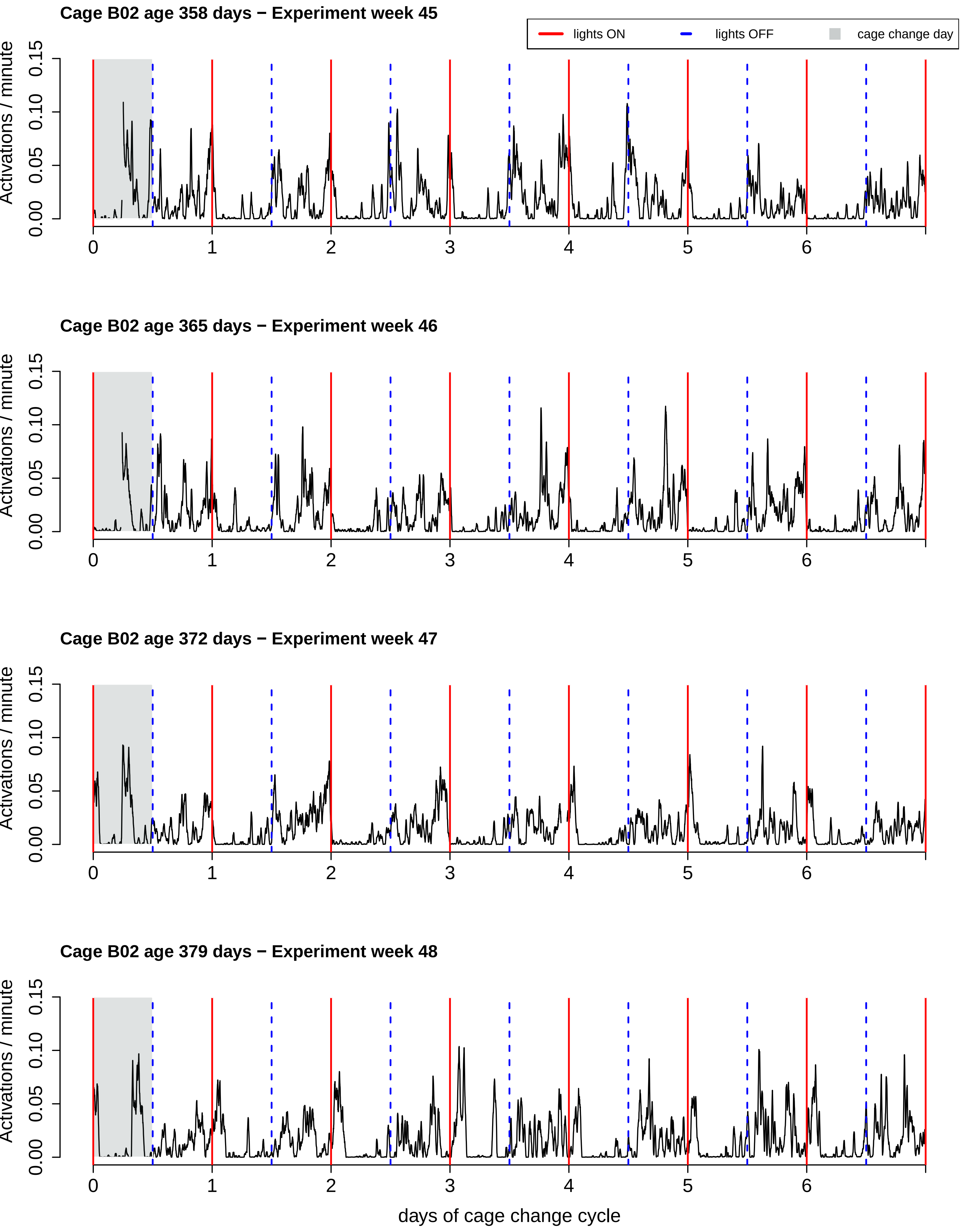

Cage B02 age 358 days − Experiment week 45
lights ON
lights OFF
cage change day
Activations / minute
0.00
0.05
0.10
0.15
0
1
2
3
4
5
6
Cage B02 age 365 days − Experiment week 46
Cage B02 age 372 days − Experiment week 47
Cage B02 age 379 days − Experiment week 48
days of cage change cycle

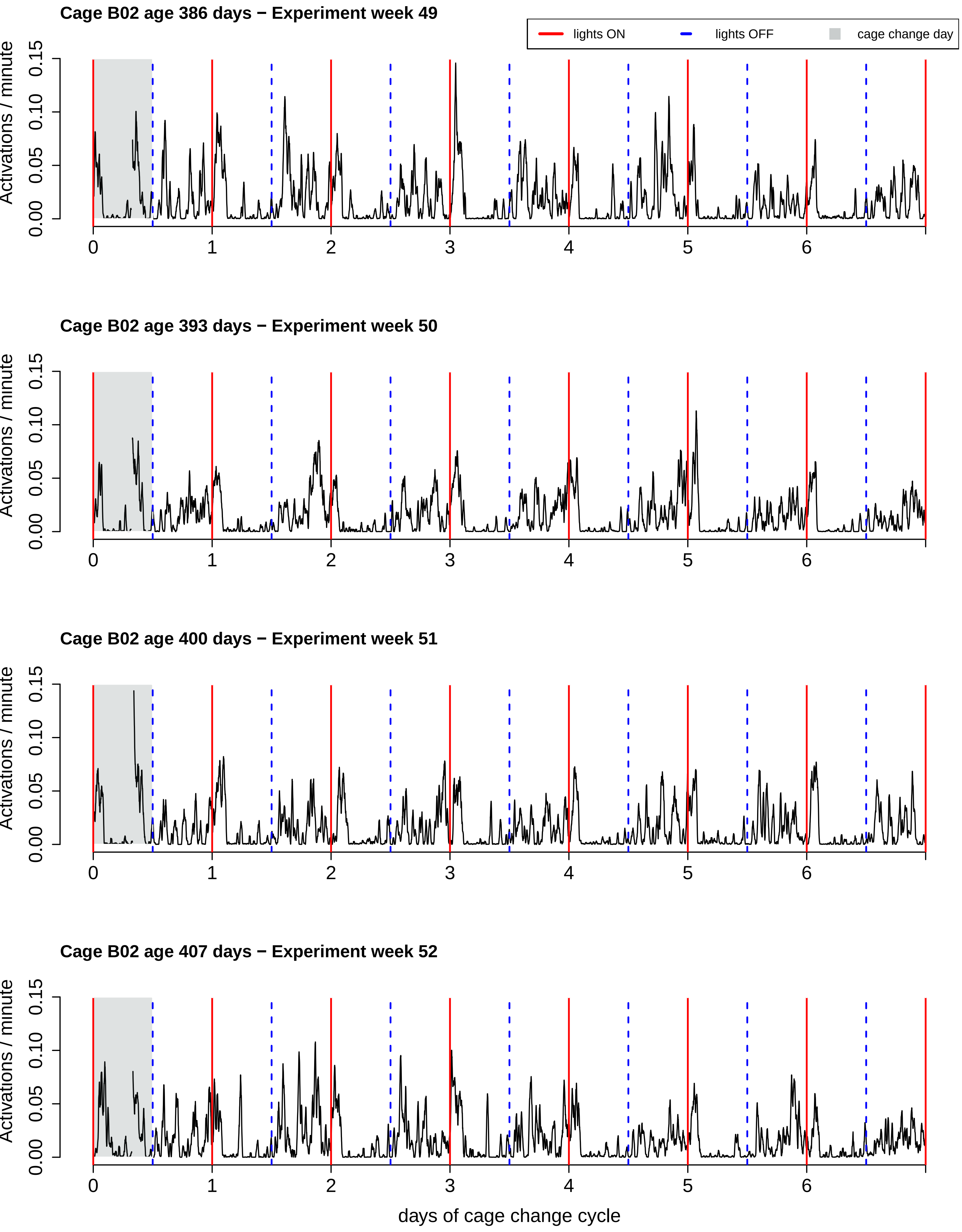
Cage B02 age 386 days − Experiment week 49
lights ON
lights OFF
cage change day
Activations / minute
0.00
0.05
0.10
0.15
0
1
2
3
4
5
6
Cage B02 age 393 days − Experiment week 50
Cage B02 age 400 days − Experiment week 51
Cage B02 age 407 days − Experiment week 52
days of cage change cycle

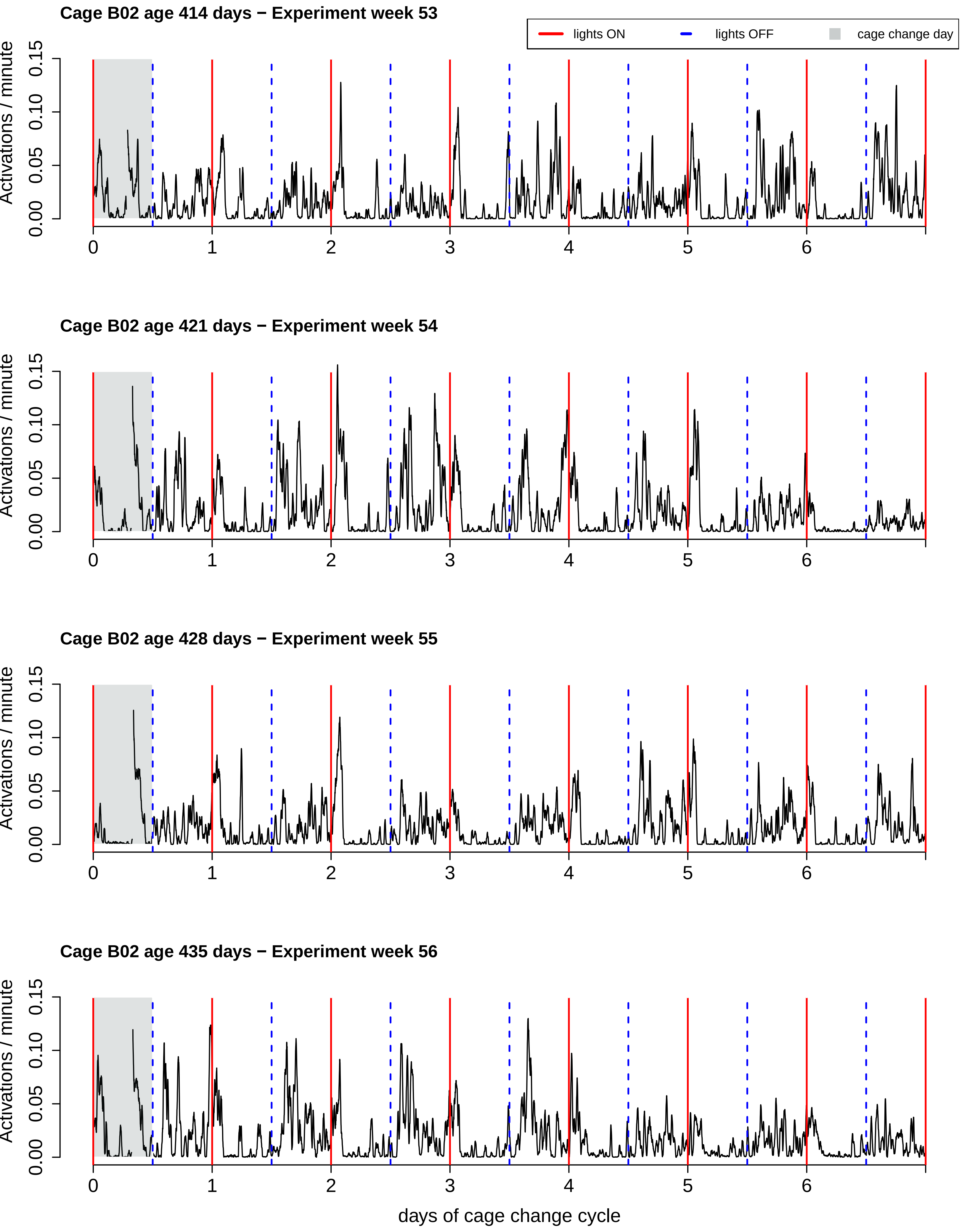

Cage B02 age 414 days − Experiment week 53
lights ON
lights OFF
cage change day
Activations / minute
0.00
0.05
0.10
0.15
0
1
2
3
4
5
6
Cage B02 age 421 days − Experiment week 54
Activations / minute
Cage B02 age 428 days − Experiment week 55
Activations / minute
Cage B02 age 435 days − Experiment week 56
Activations / minute
days of cage change cycle

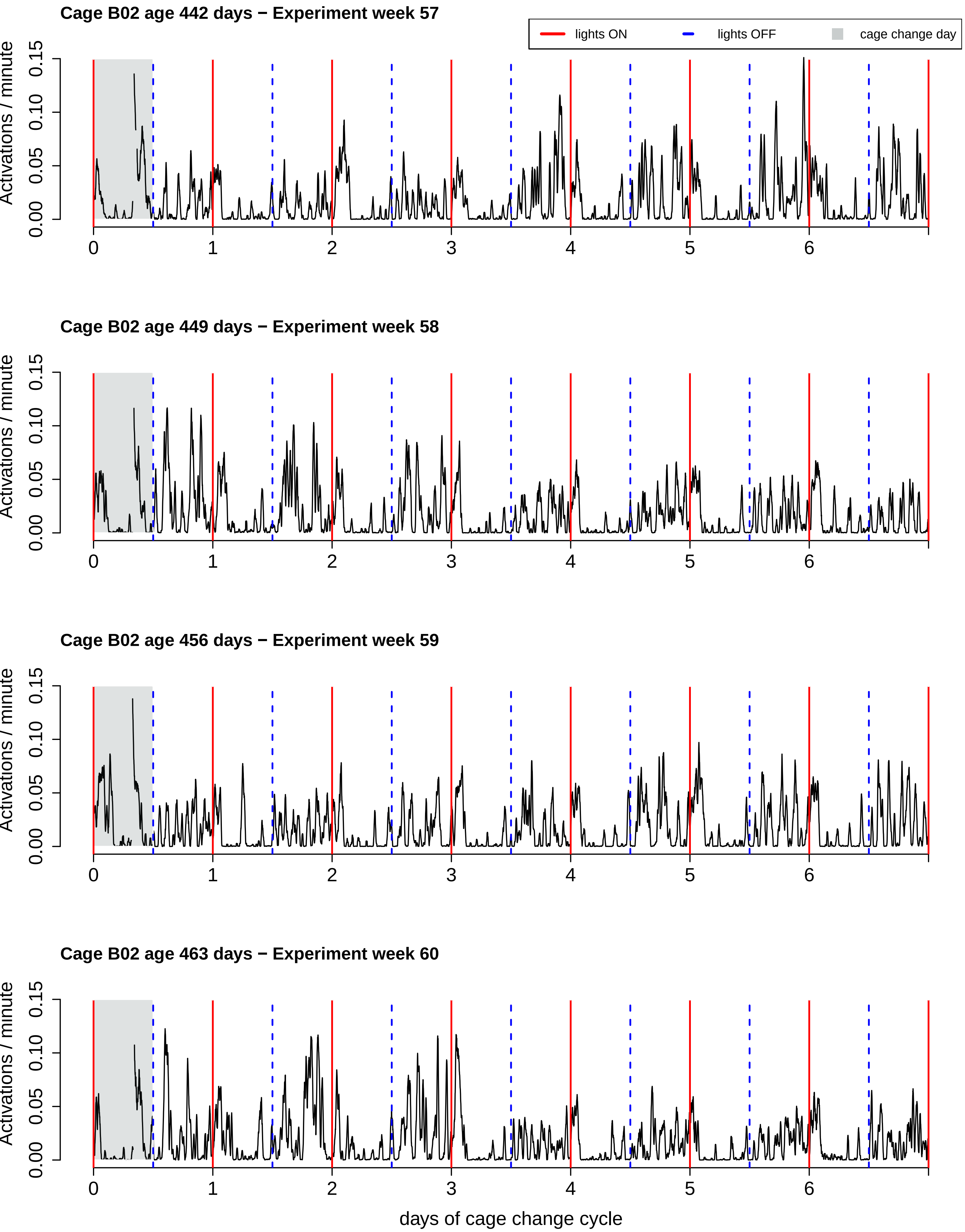
Cage B02 age 442 days − Experiment week 57
lights ON
lights OFF
cage change day
Activations / minute
Cage B02 age 449 days − Experiment week 58
Cage B02 age 456 days − Experiment week 59
Cage B02 age 463 days − Experiment week 60
days of cage change cycle

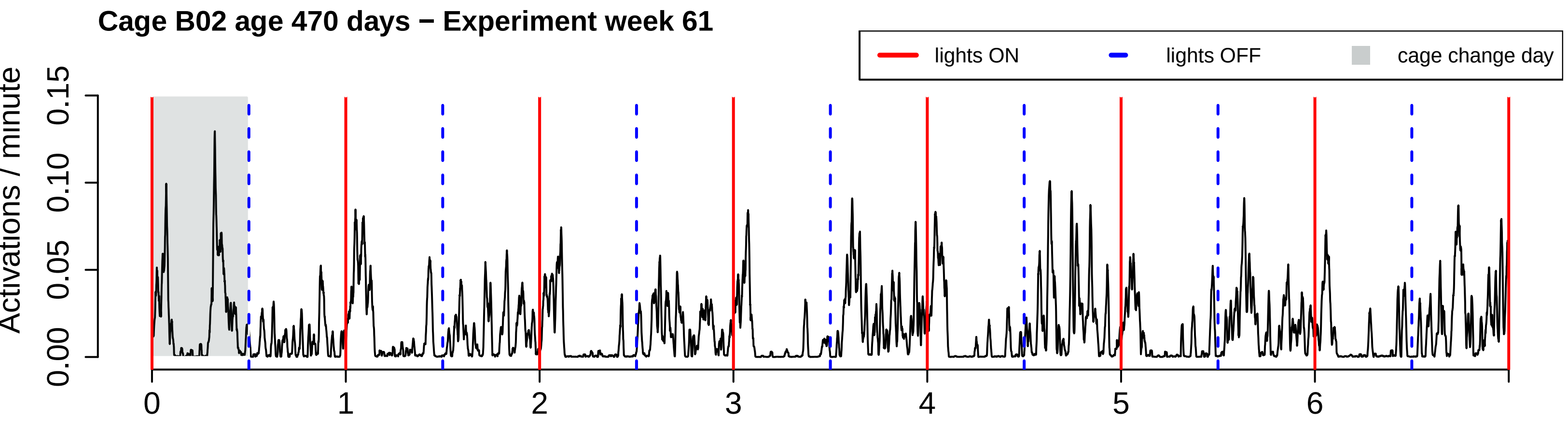

Cage B02 age 470 days − Experiment week 61
lights ON
lights OFF
cage change day
Activations / minute
0.00
0.05
0.10
0.15
0
1
2
3
4
5
6

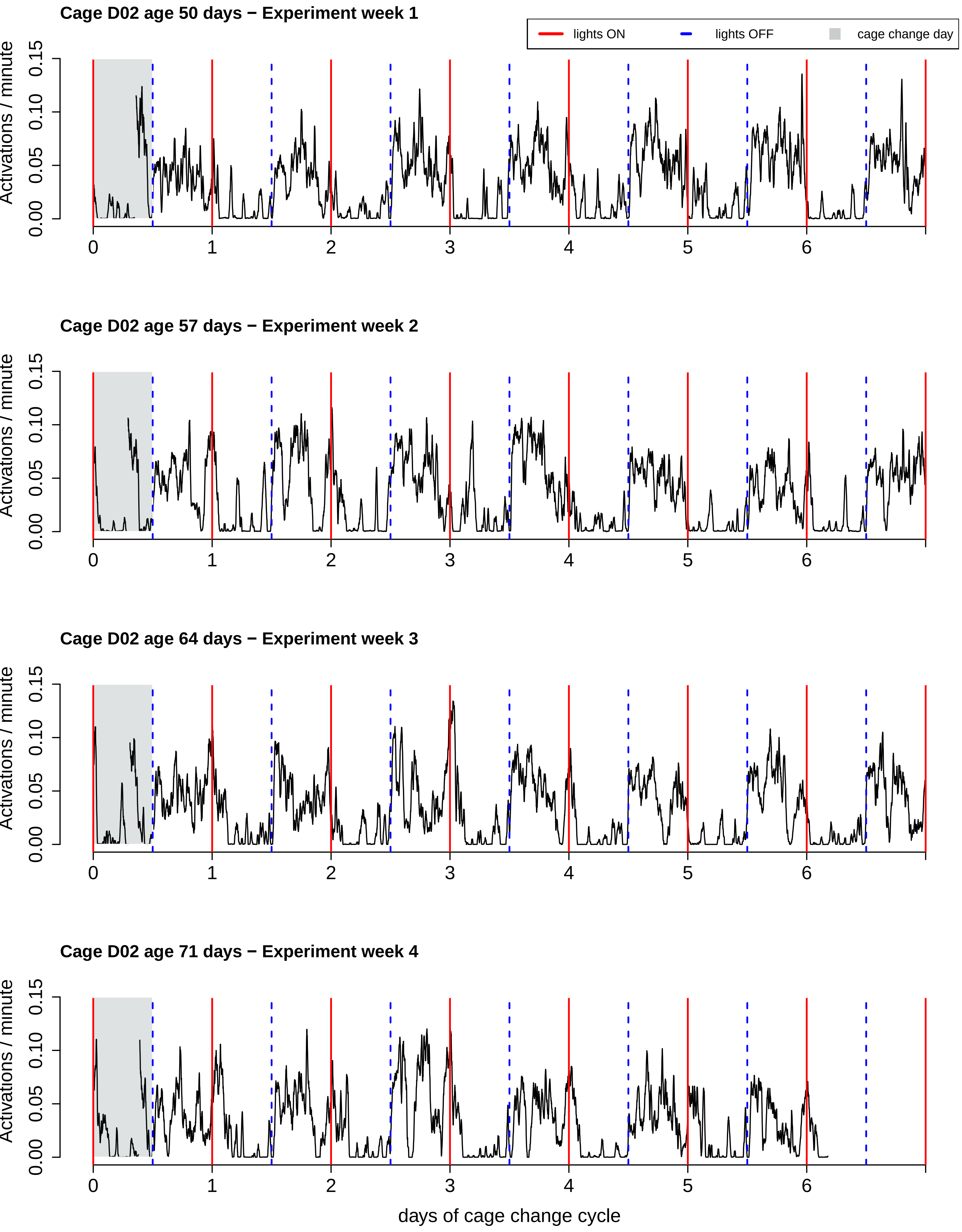
Cage D02 age 50 days − Experiment week 1
lights ON
lights OFF
cage change day
Cage D02 age 57 days − Experiment week 2
Cage D02 age 64 days − Experiment week 3
Cage D02 age 71 days − Experiment week 4
Activations / minute
0.00
0.05
0.10
0.15
0
1
2
3
4
5
6
days of cage change cycle

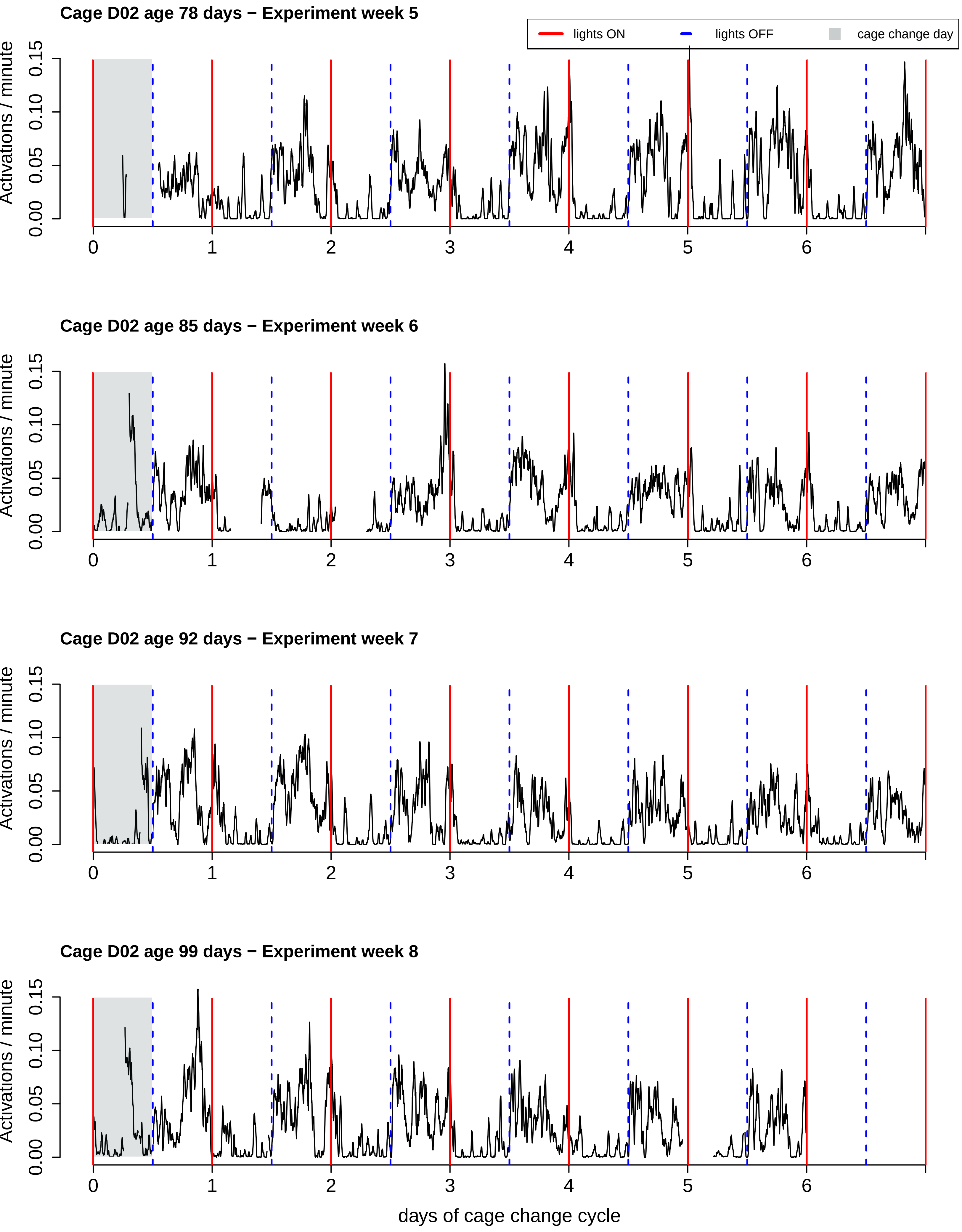

Cage D02 age 78 days − Experiment week 5
lights ON
lights OFF
cage change day
Activations / minute
0.00
0.05
0.10
0.15
0
1
2
3
4
5
6
Cage D02 age 85 days − Experiment week 6
Cage D02 age 92 days − Experiment week 7
Cage D02 age 99 days − Experiment week 8
days of cage change cycle

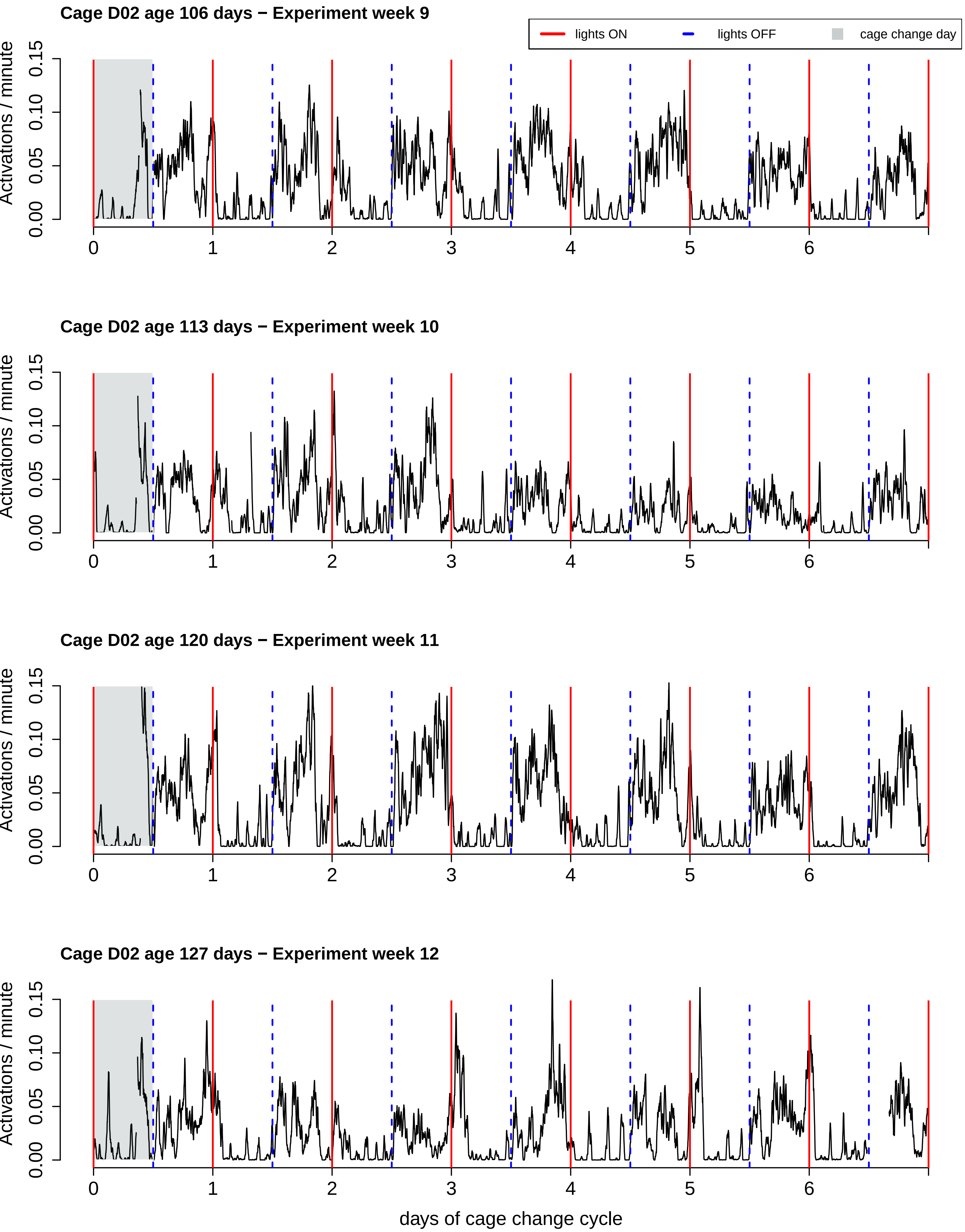
Cage D02 age 106 days − Experiment week 9
lights ON
lights OFF
cage change day
Activations / minute
0.00
0.05
0.10
0.15
0 1 2 3 4 5 6
Cage D02 age 113 days − Experiment week 10
Cage D02 age 120 days − Experiment week 11
Cage D02 age 127 days − Experiment week 12
days of cage change cycle

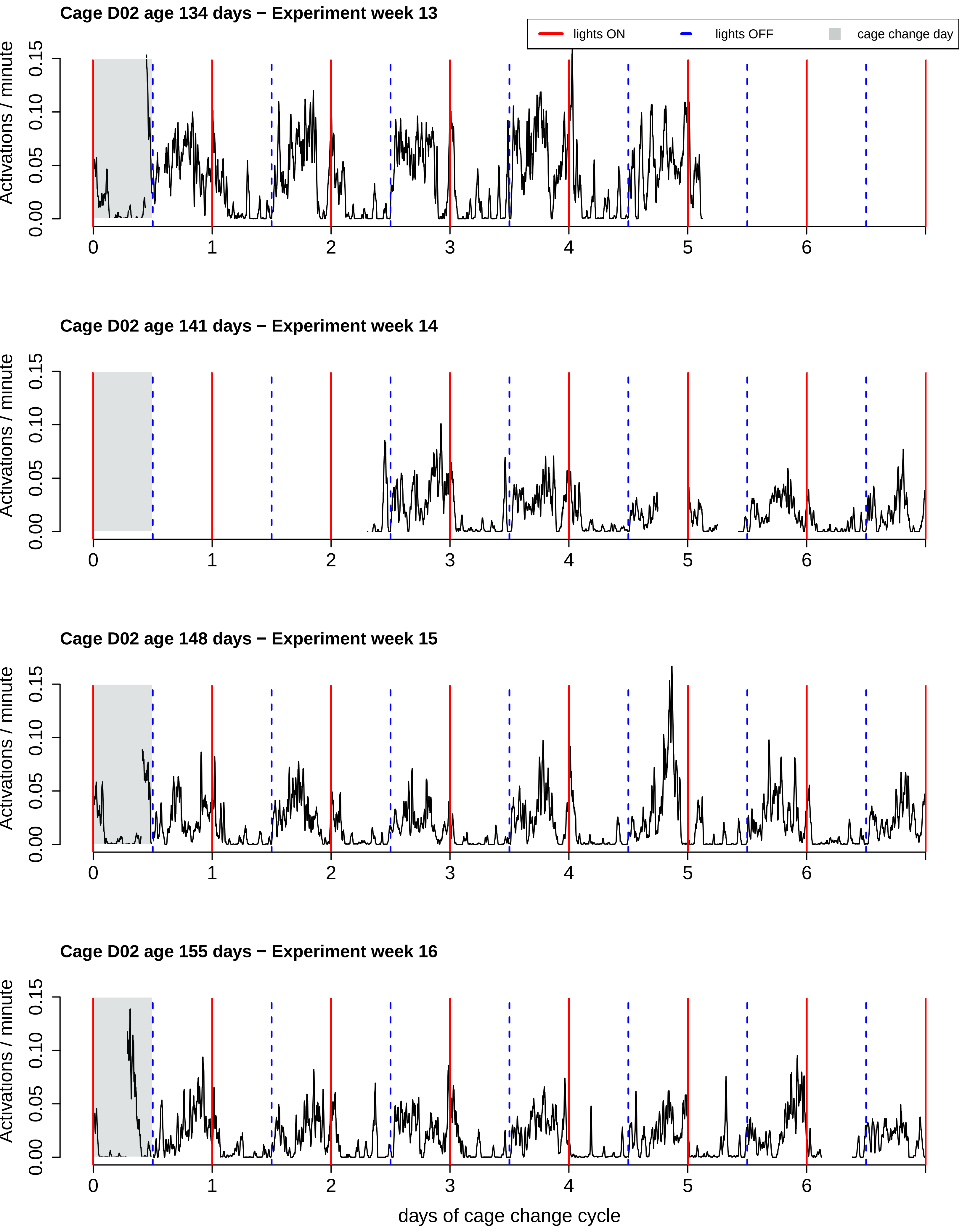
Cage D02 age 134 days − Experiment week 13
lights ON
lights OFF
cage change day
Activations / minute
0.00
0.05
0.10
0.15
0
1
2
3
4
5
6
Cage D02 age 141 days − Experiment week 14
Cage D02 age 148 days − Experiment week 15
Cage D02 age 155 days − Experiment week 16
days of cage change cycle

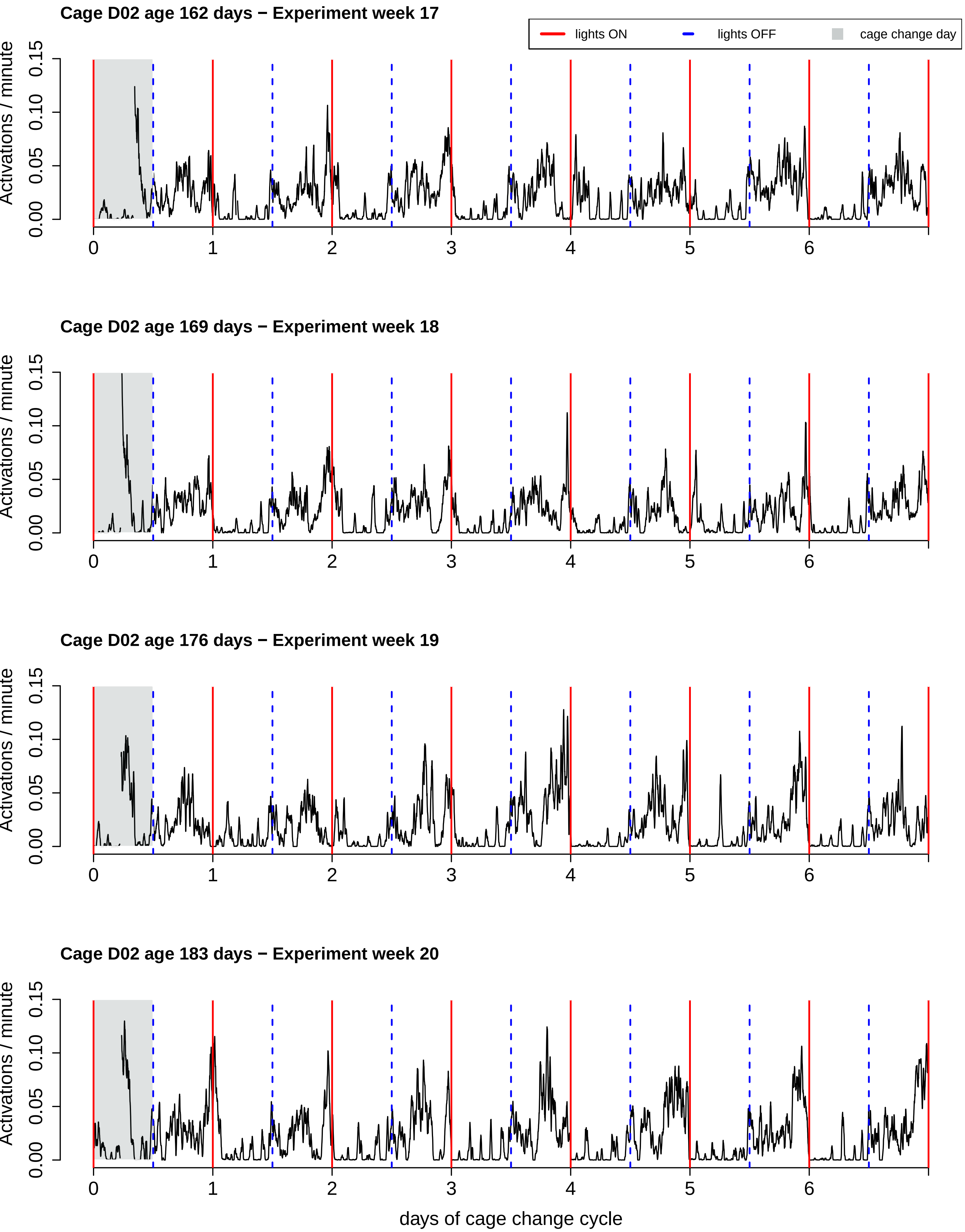

Cage D02 age 162 days − Experiment week 17
lights ON
lights OFF
cage change day
Activations / minute
0.00
0.05
0.10
0.15
0
1
2
3
4
5
6
Cage D02 age 169 days − Experiment week 18
Cage D02 age 176 days − Experiment week 19
Cage D02 age 183 days − Experiment week 20
days of cage change cycle

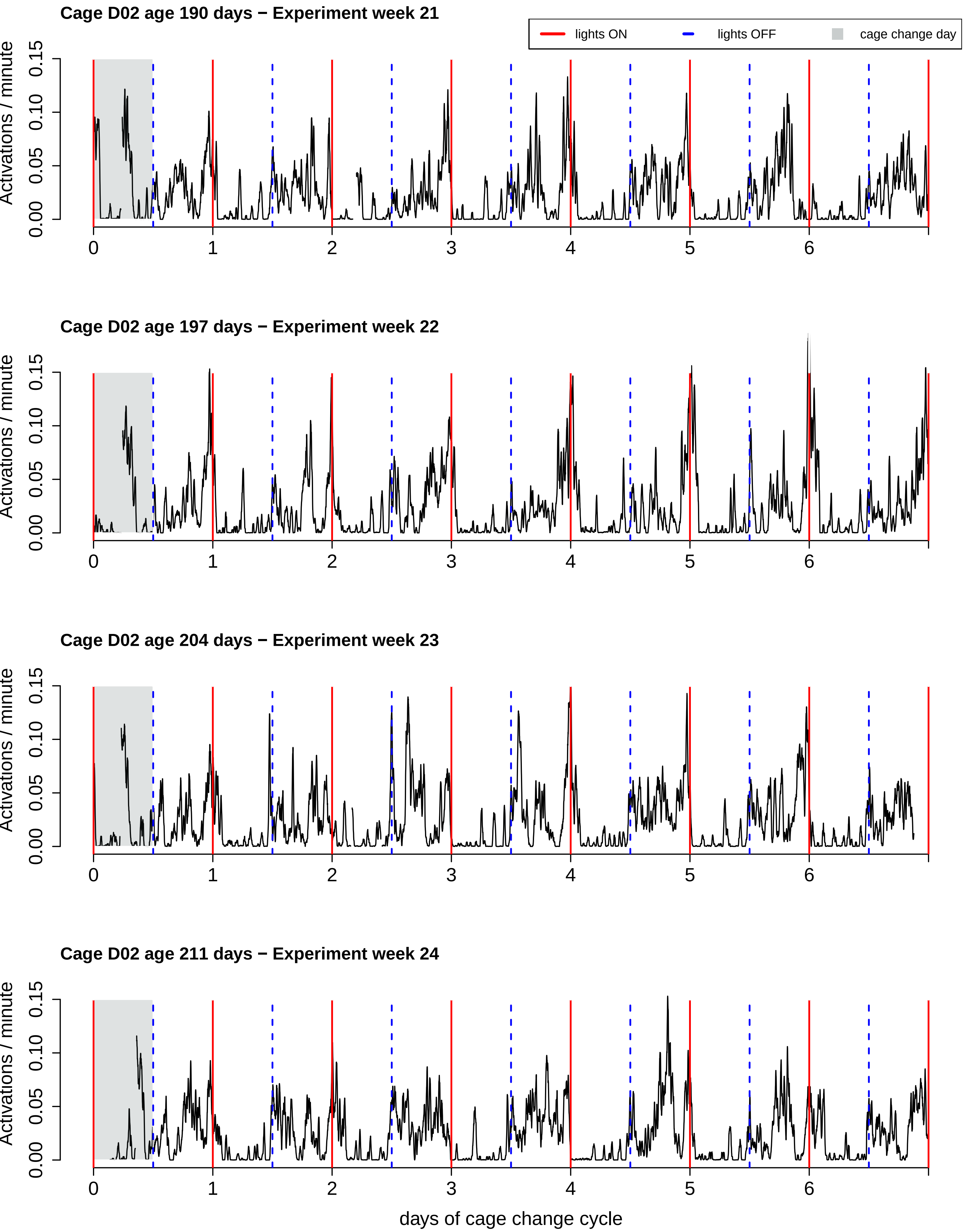
Cage D02 age 190 days − Experiment week 21
lights ON
lights OFF
cage change day
Activations / minute
0.00
0.05
0.10
0.15
0
1
2
3
4
5
6
Cage D02 age 197 days − Experiment week 22
Activations / minute
0.00
0.05
0.10
0.15
0
1
2
3
4
5
6
Cage D02 age 204 days − Experiment week 23
Activations / minute
0.00
0.05
0.10
0.15
0
1
2
3
4
5
6
Cage D02 age 211 days − Experiment week 24
Activations / minute
0.00
0.05
0.10
0.15
0
1
2
3
4
5
6
days of cage change cycle

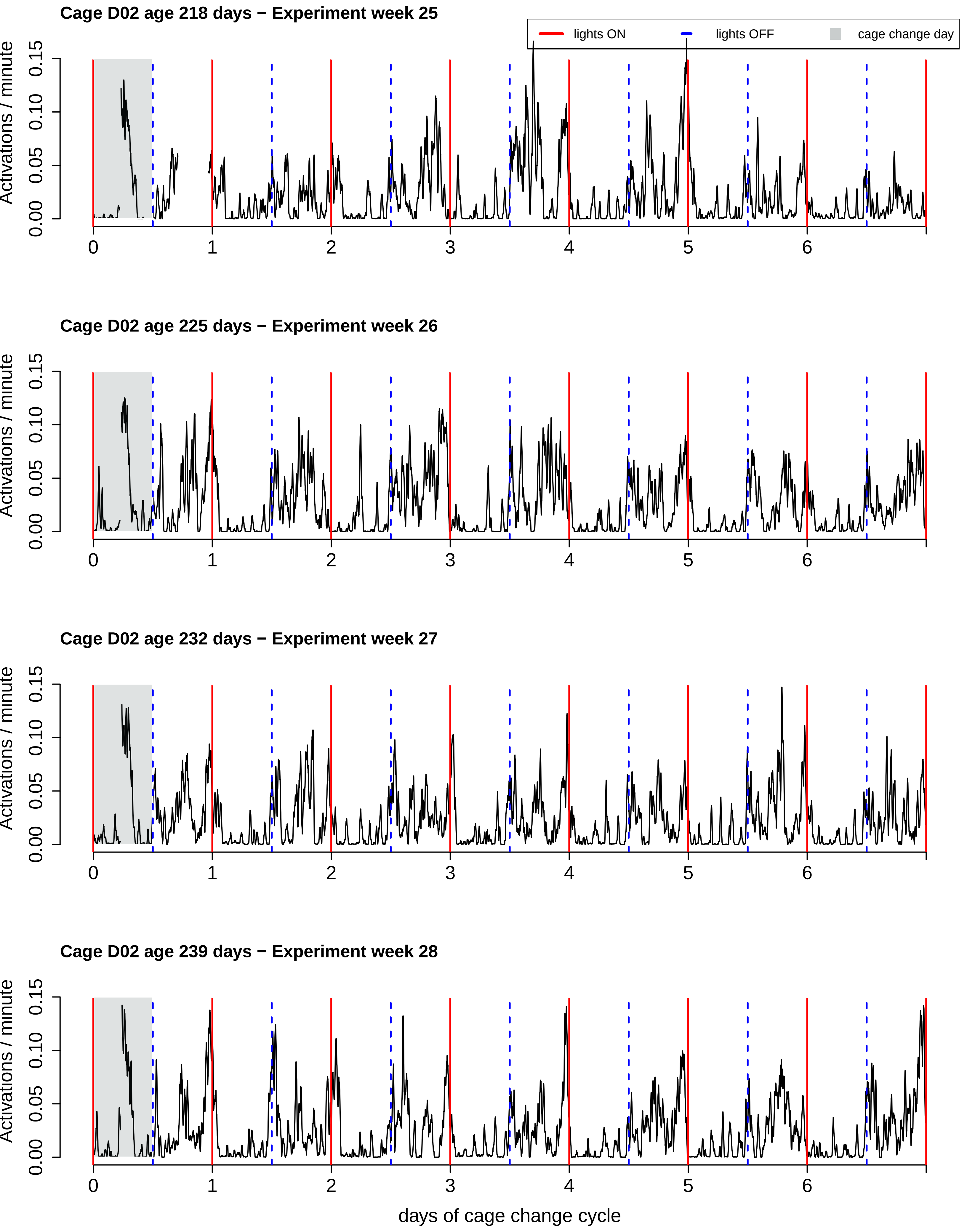
Cage D02 age 218 days − Experiment week 25
lights ON
lights OFF
cage change day
Cage D02 age 225 days − Experiment week 26
Cage D02 age 232 days − Experiment week 27
Cage D02 age 239 days − Experiment week 28
Activations / minute
0.00
0.05
0.10
0.15
0
1
2
3
4
5
6
days of cage change cycle

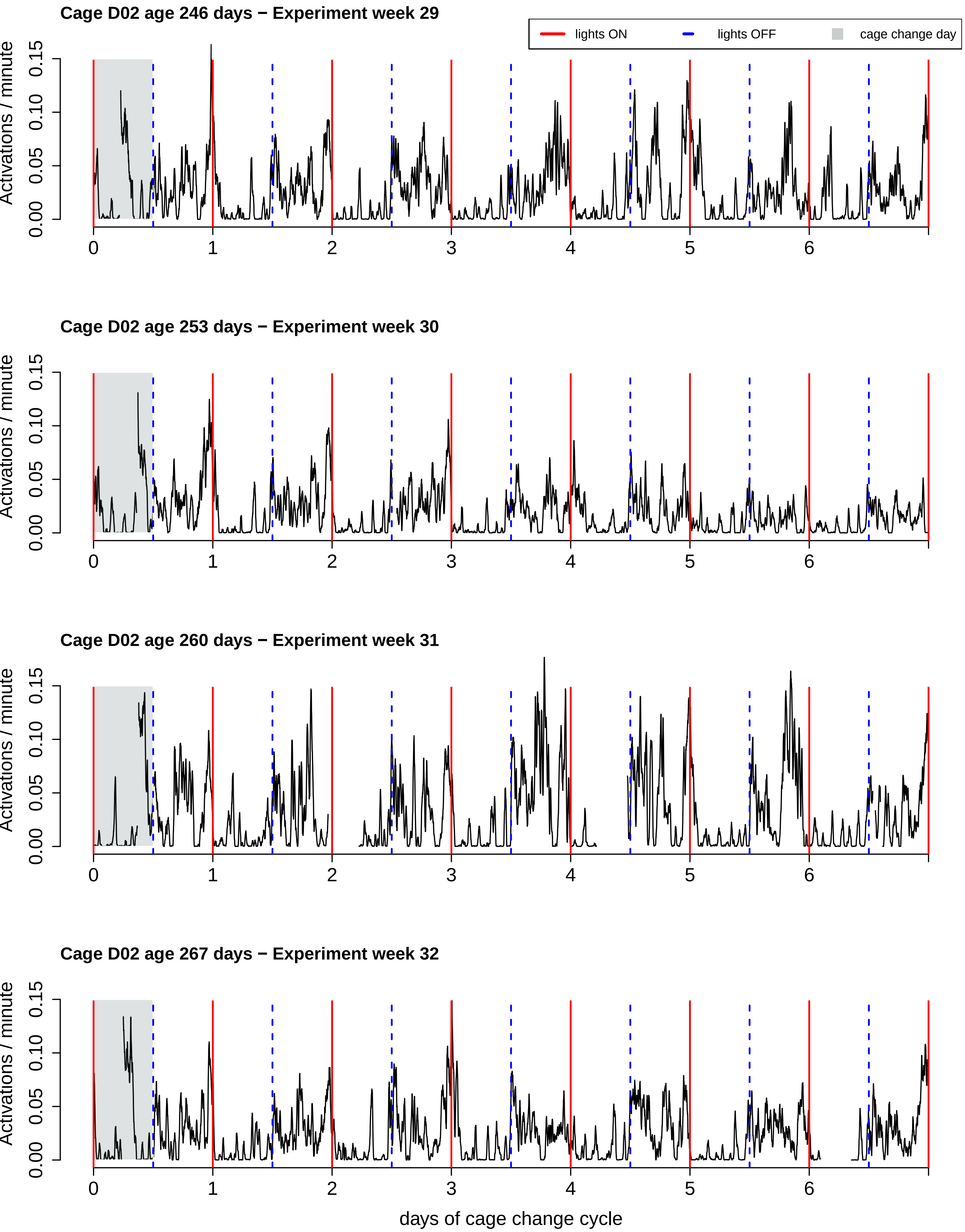
Cage D02 age 246 days − Experiment week 29
lights ON
lights OFF
cage change day
Activations / minute
Cage D02 age 253 days − Experiment week 30
Cage D02 age 260 days − Experiment week 31
Cage D02 age 267 days − Experiment week 32
days of cage change cycle

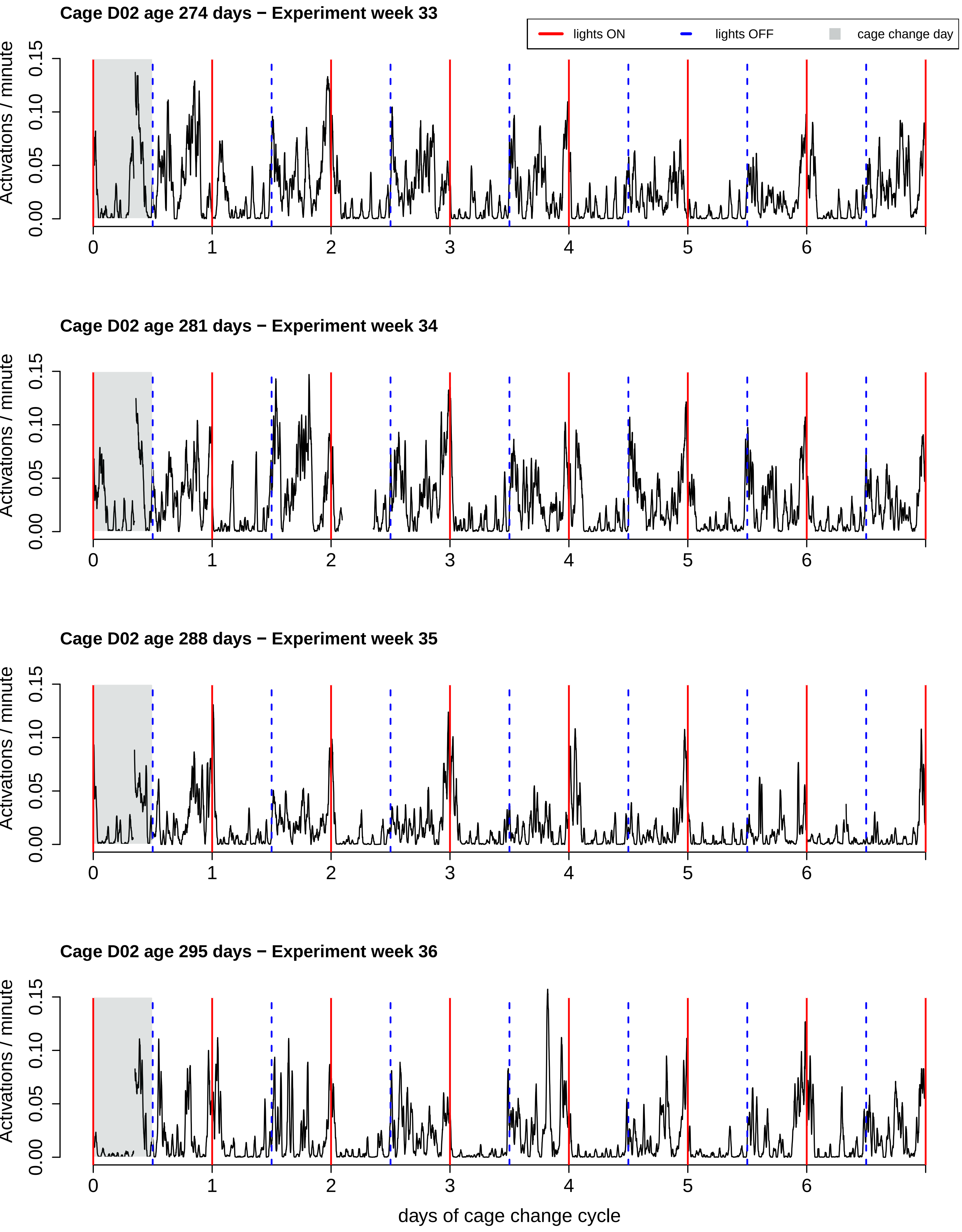

Cage D02 age 274 days − Experiment week 33
lights ON
lights OFF
cage change day
Activations / minute
0.00
0.05
0.10
0.15
0
1
2
3
4
5
6
Cage D02 age 281 days − Experiment week 34
Cage D02 age 288 days − Experiment week 35
Cage D02 age 295 days − Experiment week 36
days of cage change cycle

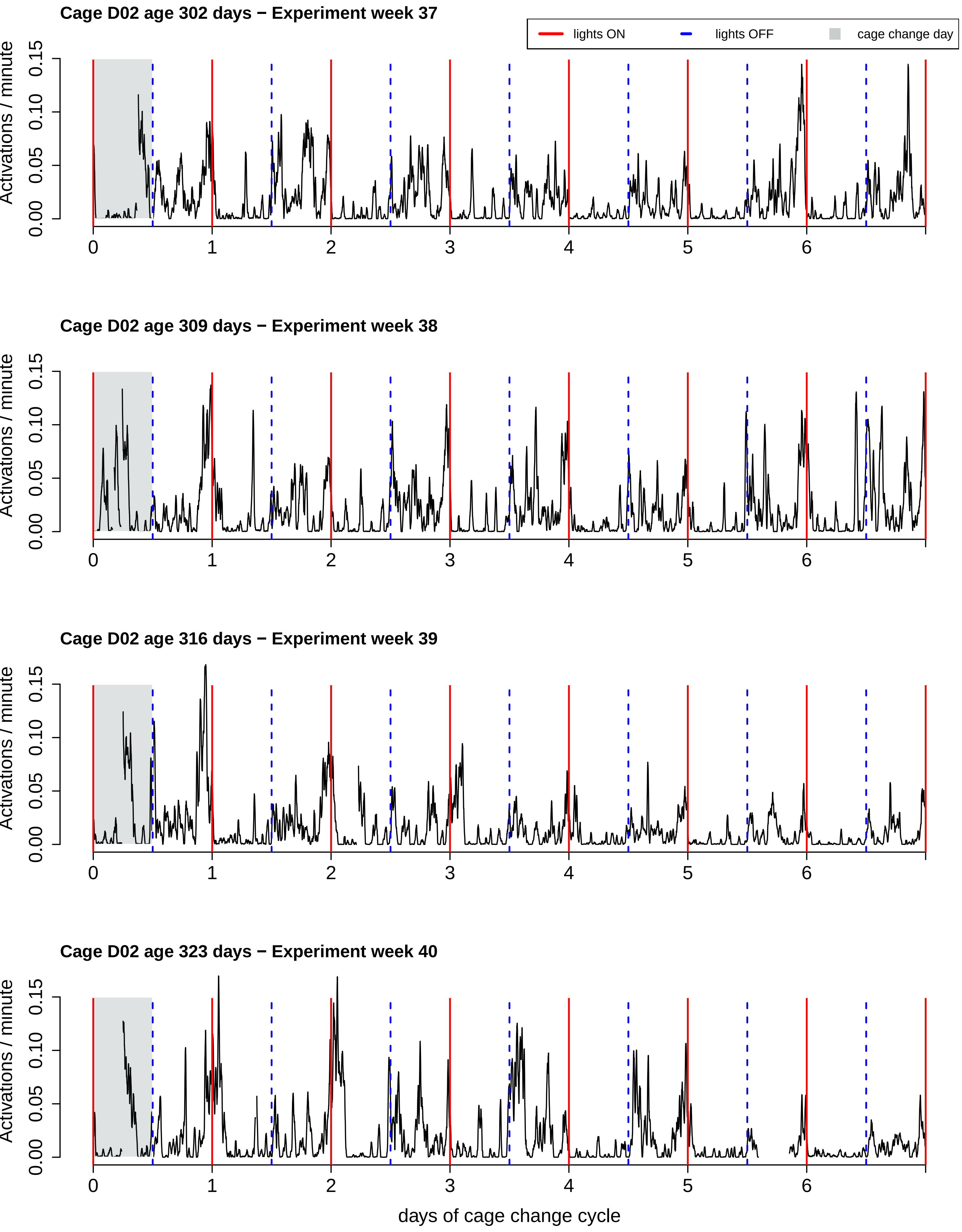
Cage D02 age 302 days − Experiment week 37
lights ON
lights OFF
cage change day
Activations / minute
0.00
0.05
0.10
0.15
0
1
2
3
4
5
6
Cage D02 age 309 days − Experiment week 38
Cage D02 age 316 days − Experiment week 39
Cage D02 age 323 days − Experiment week 40
days of cage change cycle

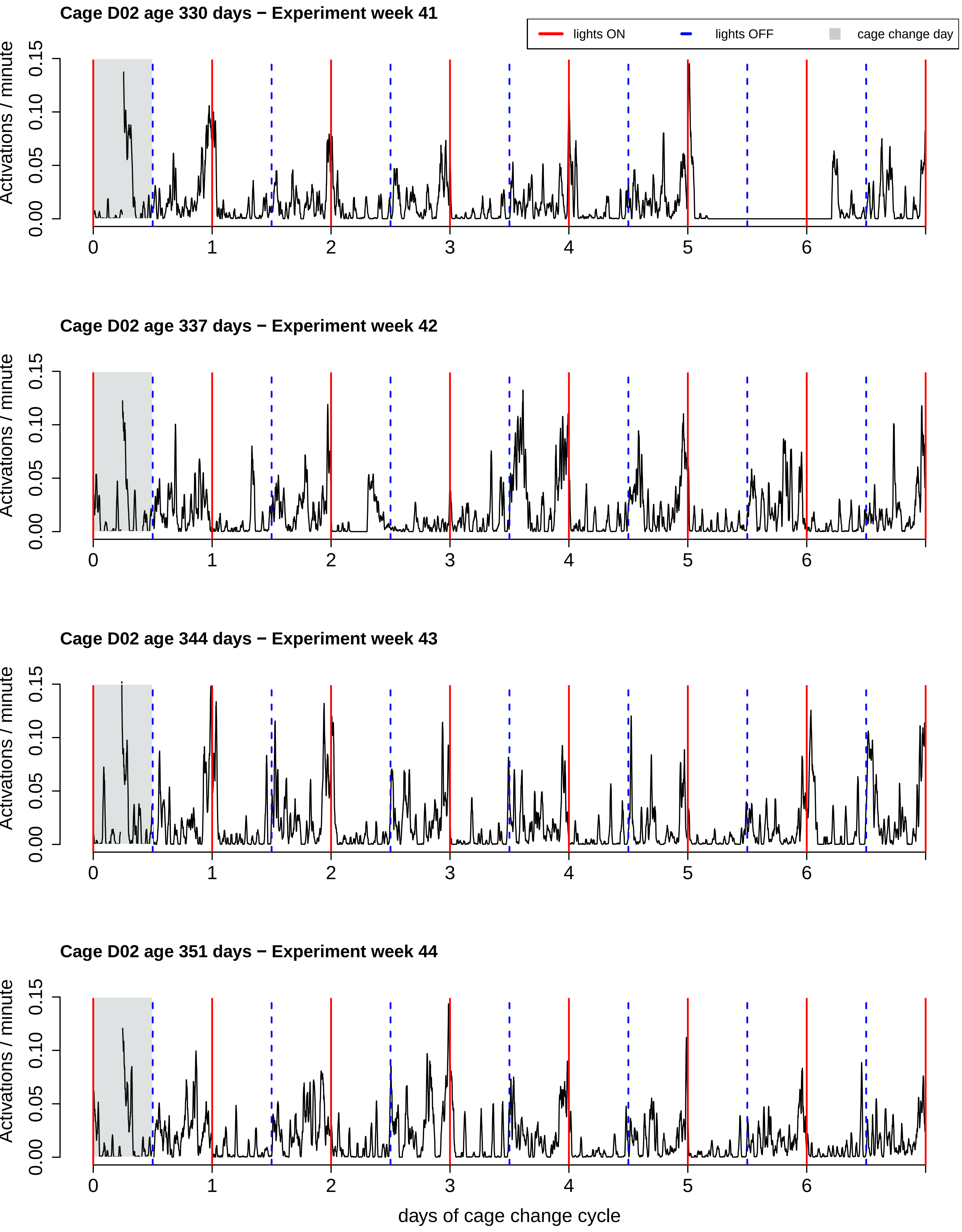
Cage D02 age 330 days − Experiment week 41
lights ON
lights OFF
cage change day
Activations / minute
0.00
0.05
0.10
0.15
0
1
2
3
4
5
6
Cage D02 age 337 days − Experiment week 42
Cage D02 age 344 days − Experiment week 43
Cage D02 age 351 days − Experiment week 44
days of cage change cycle

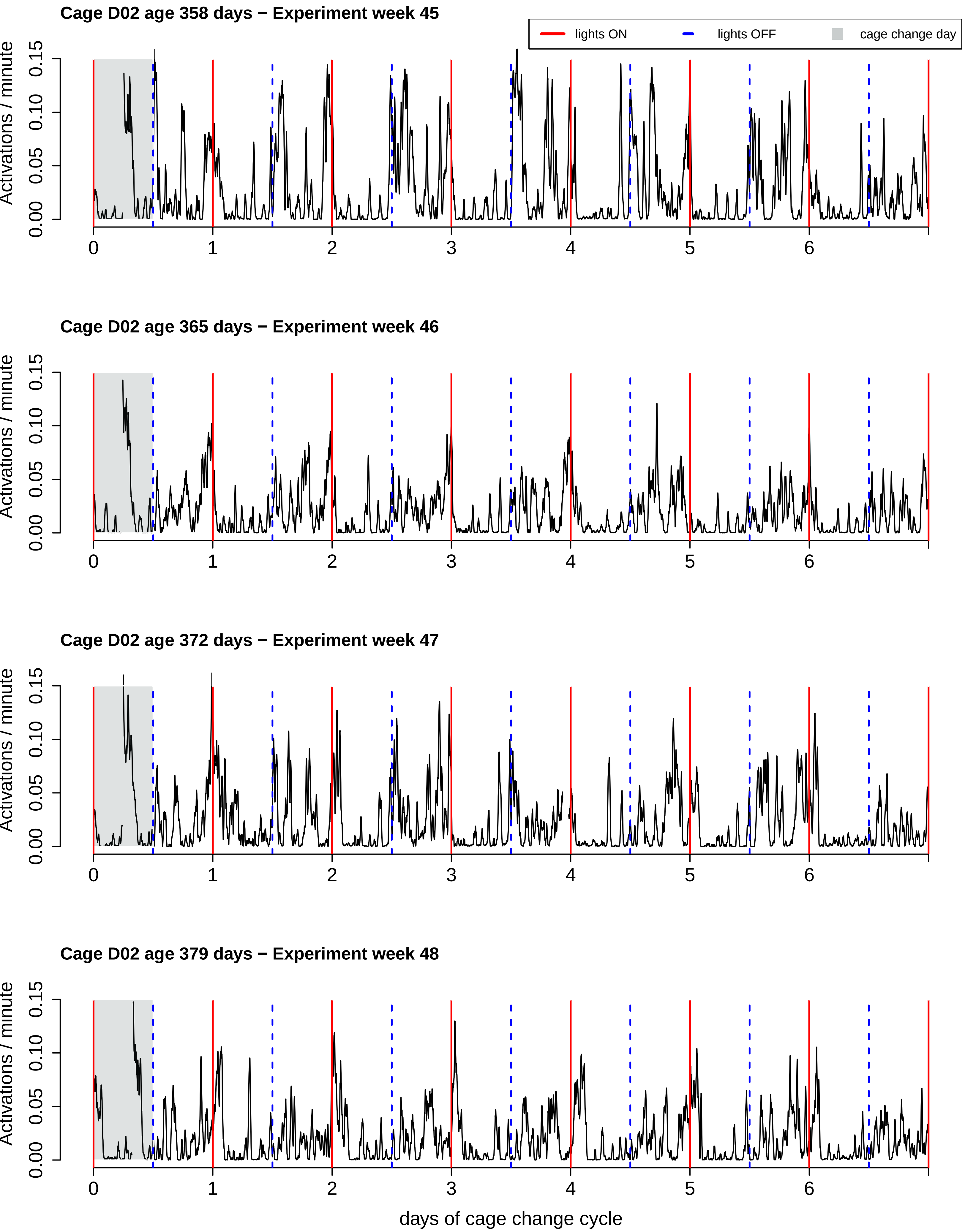

Cage D02 age 358 days − Experiment week 45
lights ON
lights OFF
cage change day
Activations / minute
Cage D02 age 365 days − Experiment week 46
Activations / minute
Cage D02 age 372 days − Experiment week 47
Activations / minute
Cage D02 age 379 days − Experiment week 48
Activations / minute
days of cage change cycle

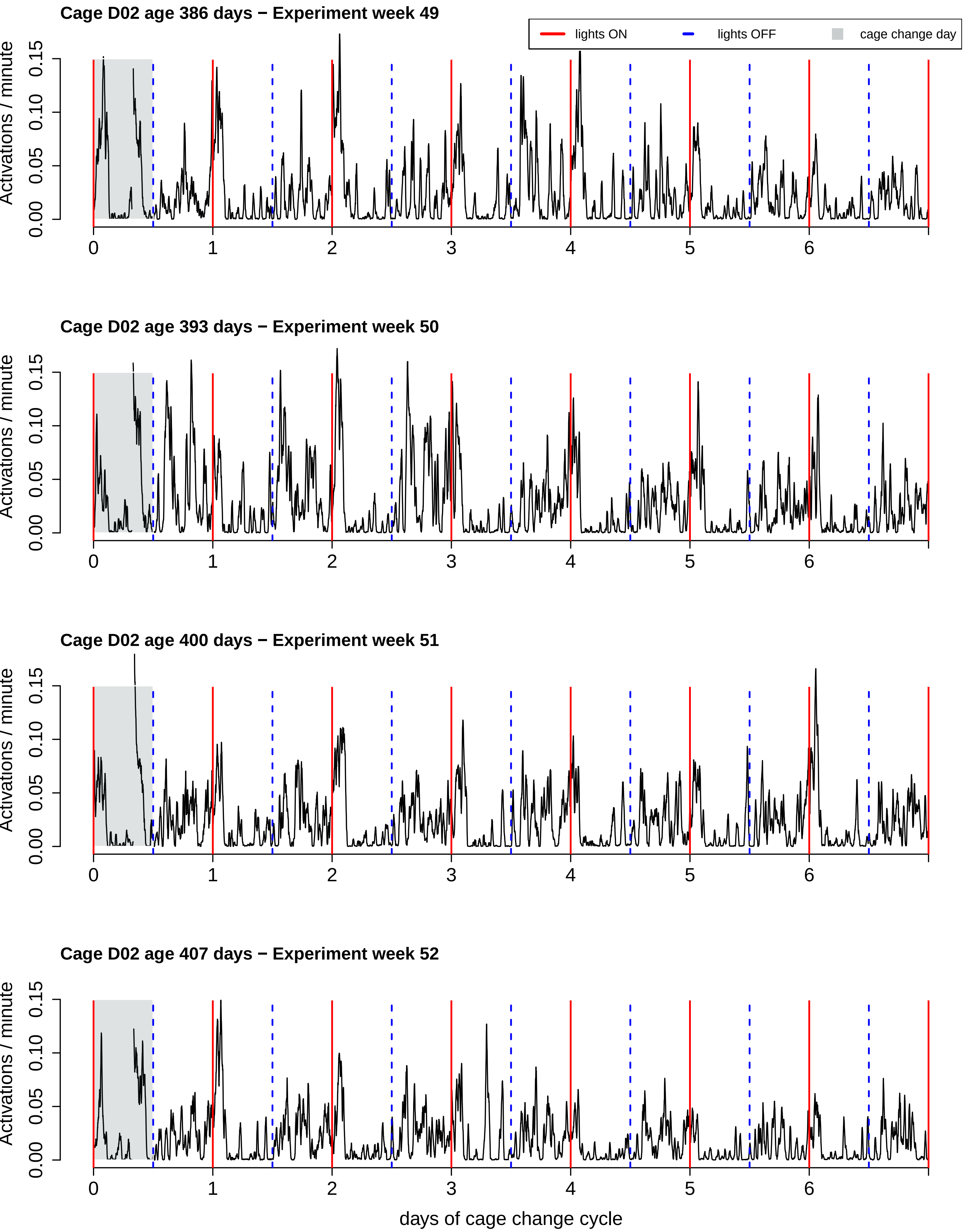

Cage D02 age 386 days − Experiment week 49
lights ON
lights OFF
cage change day
Activations / minute
0.00
0.05
0.10
0.15
0
1
2
3
4
5
6
Cage D02 age 393 days − Experiment week 50
Cage D02 age 400 days − Experiment week 51
Cage D02 age 407 days − Experiment week 52
days of cage change cycle

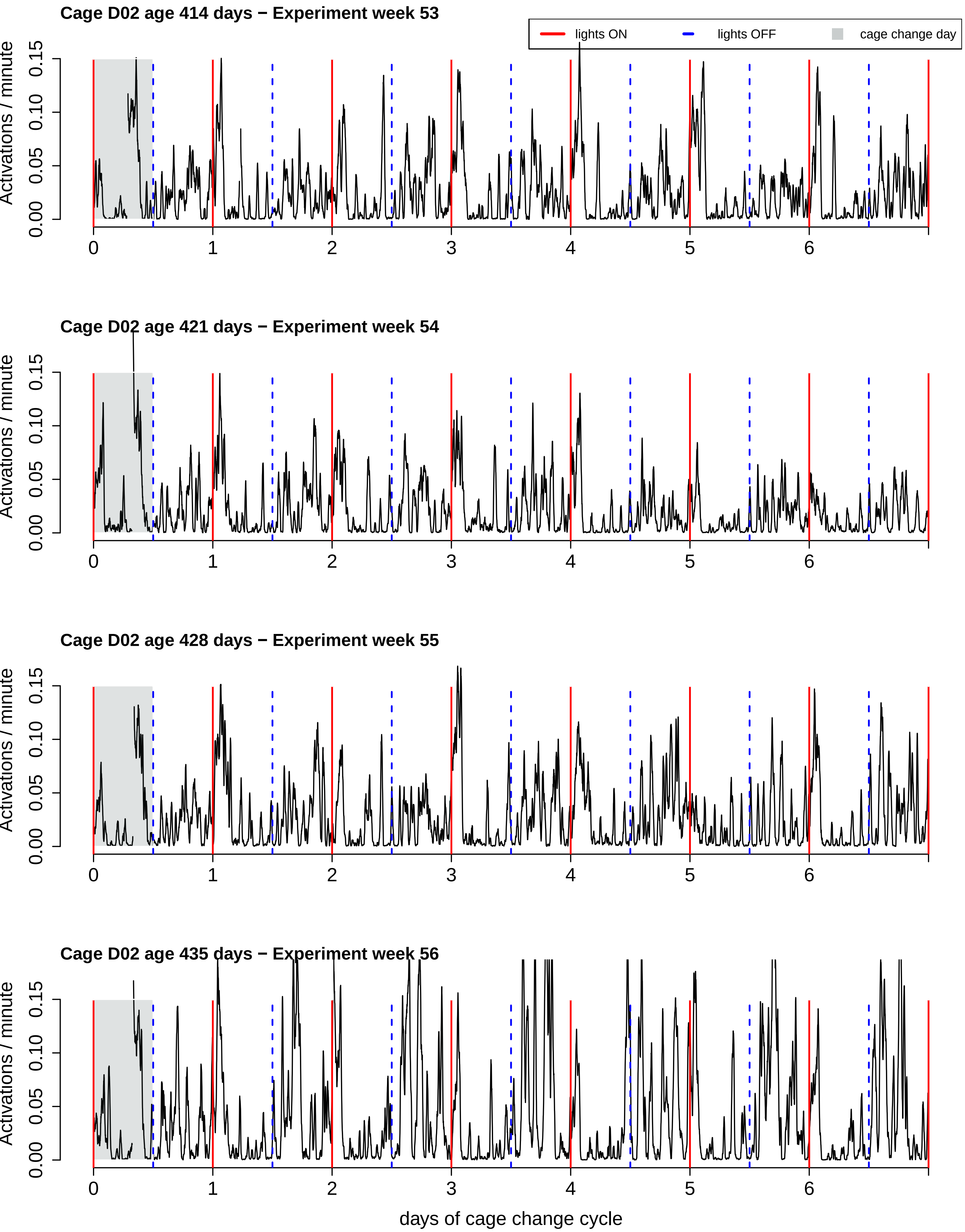
Cage D02 age 414 days − Experiment week 53
lights ON
lights OFF
cage change day
Activations / minute
0.00
0.05
0.10
0.15
0
1
2
3
4
5
6
Cage D02 age 421 days − Experiment week 54
Activations / minute
0.00
0.05
0.10
0.15
0
1
2
3
4
5
6
Cage D02 age 428 days − Experiment week 55
Activations / minute
0.00
0.05
0.10
0.15
0
1
2
3
4
5
6
Cage D02 age 435 days − Experiment week 56
Activations / minute
0.00
0.05
0.10
0.15
0
1
2
3
4
5
6
days of cage change cycle

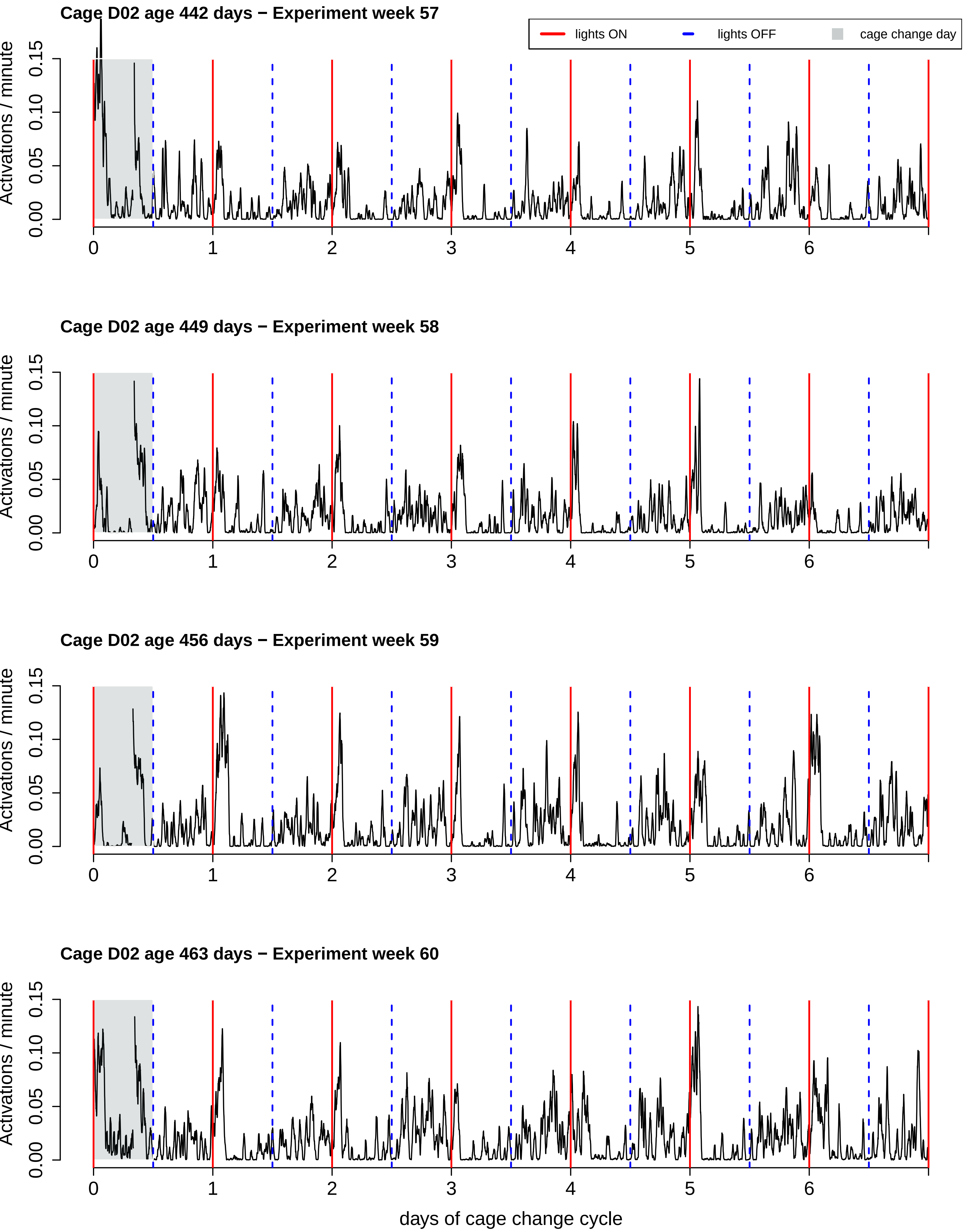
Cage D02 age 442 days − Experiment week 57
lights ON
lights OFF
cage change day
Activations / minute
0.00
0.05
0.10
0.15
0
1
2
3
4
5
6
Cage D02 age 449 days − Experiment week 58
Activations / minute
0.00
0.05
0.10
0.15
0
1
2
3
4
5
6
Cage D02 age 456 days − Experiment week 59
Activations / minute
0.00
0.05
0.10
0.15
0
1
2
3
4
5
6
Cage D02 age 463 days − Experiment week 60
Activations / minute
0.00
0.05
0.10
0.15
0
1
2
3
4
5
6
days of cage change cycle

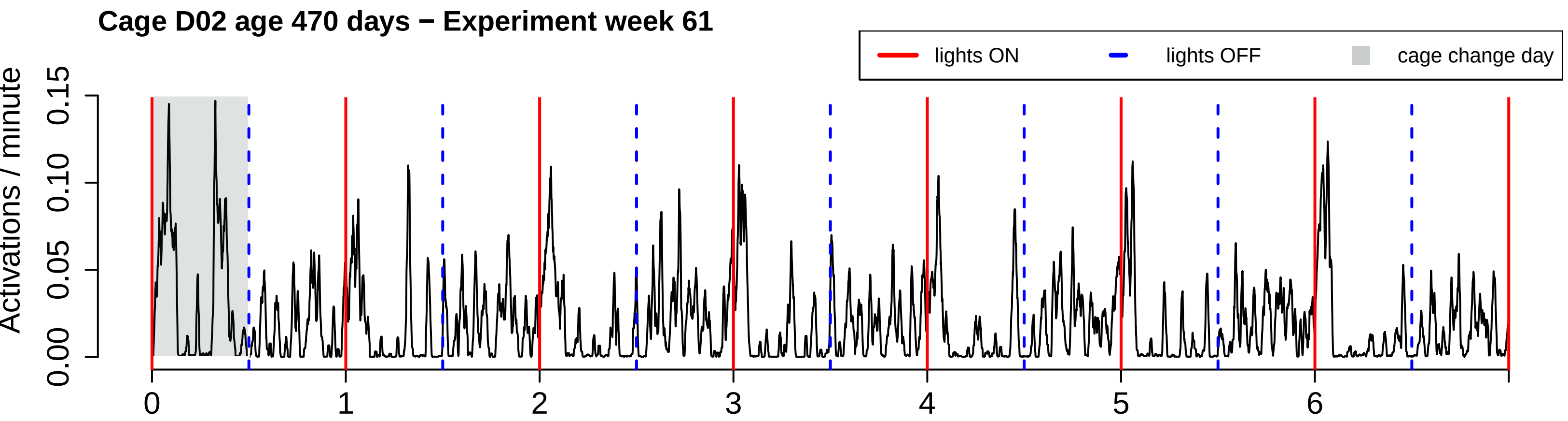

Cage D02 age 470 days − Experiment week 61
lights ON
lights OFF
cage change day
Activations / minute
0.00
0.05
0.10
0.15
0
1
2
3
4
5
6

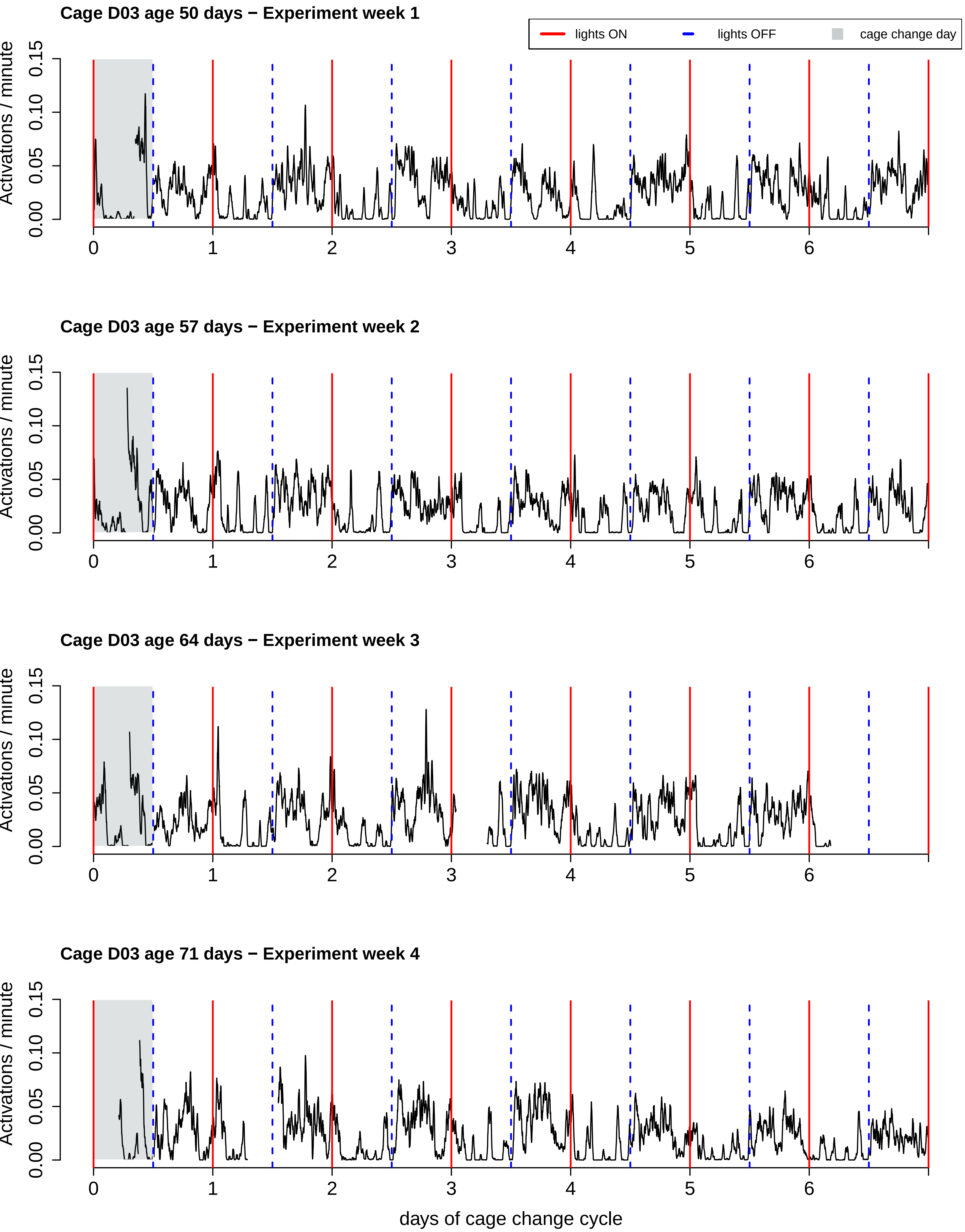
Cage D03 age 50 days − Experiment week 1
lights ON
lights OFF
cage change day
Activations / minute
0.00
0.05
0.10
0.15
0
1
2
3
4
5
6
Cage D03 age 57 days − Experiment week 2
Cage D03 age 64 days − Experiment week 3
Cage D03 age 71 days − Experiment week 4
days of cage change cycle

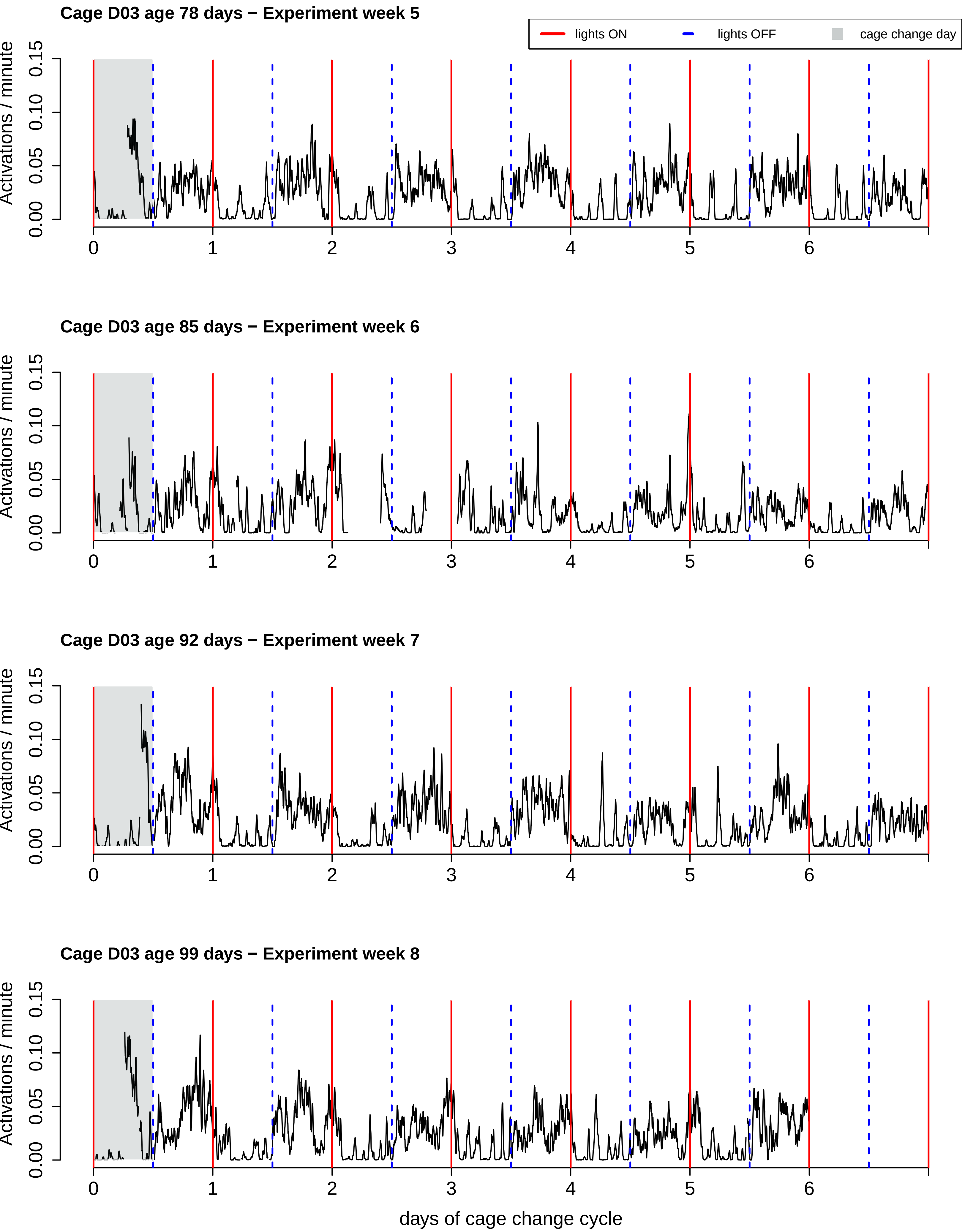

Cage D03 age 78 days − Experiment week 5
lights ON
lights OFF
cage change day
Activations / minute
0.00
0.05
0.10
0.15
0
1
2
3
4
5
6
Cage D03 age 85 days − Experiment week 6
Cage D03 age 92 days − Experiment week 7
Cage D03 age 99 days − Experiment week 8
days of cage change cycle

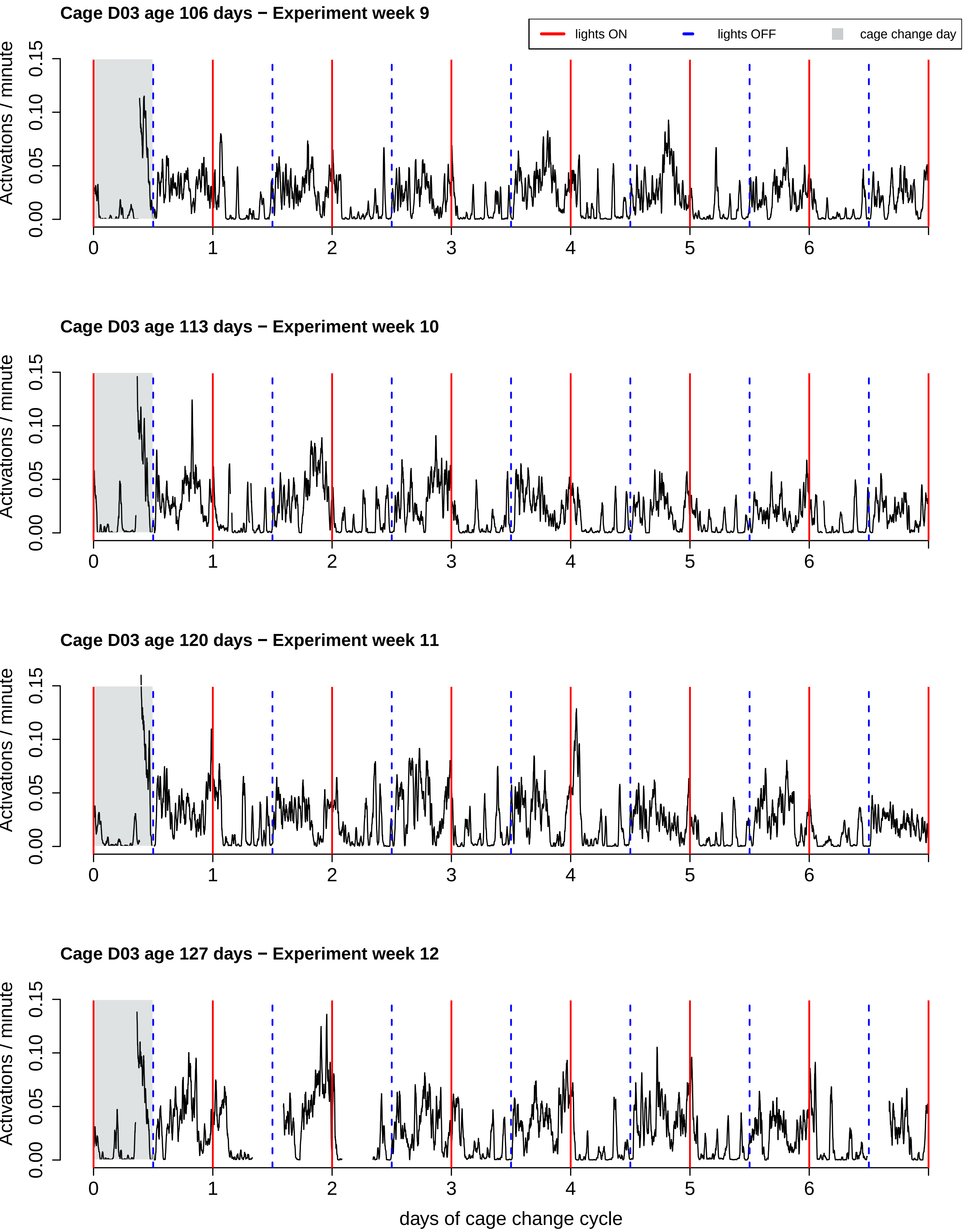

Cage D03 age 106 days − Experiment week 9
lights ON
lights OFF
cage change day
Activations / minute
0.00
0.05
0.10
0.15
0
1
2
3
4
5
6
Cage D03 age 113 days − Experiment week 10
Cage D03 age 120 days − Experiment week 11
Cage D03 age 127 days − Experiment week 12
days of cage change cycle

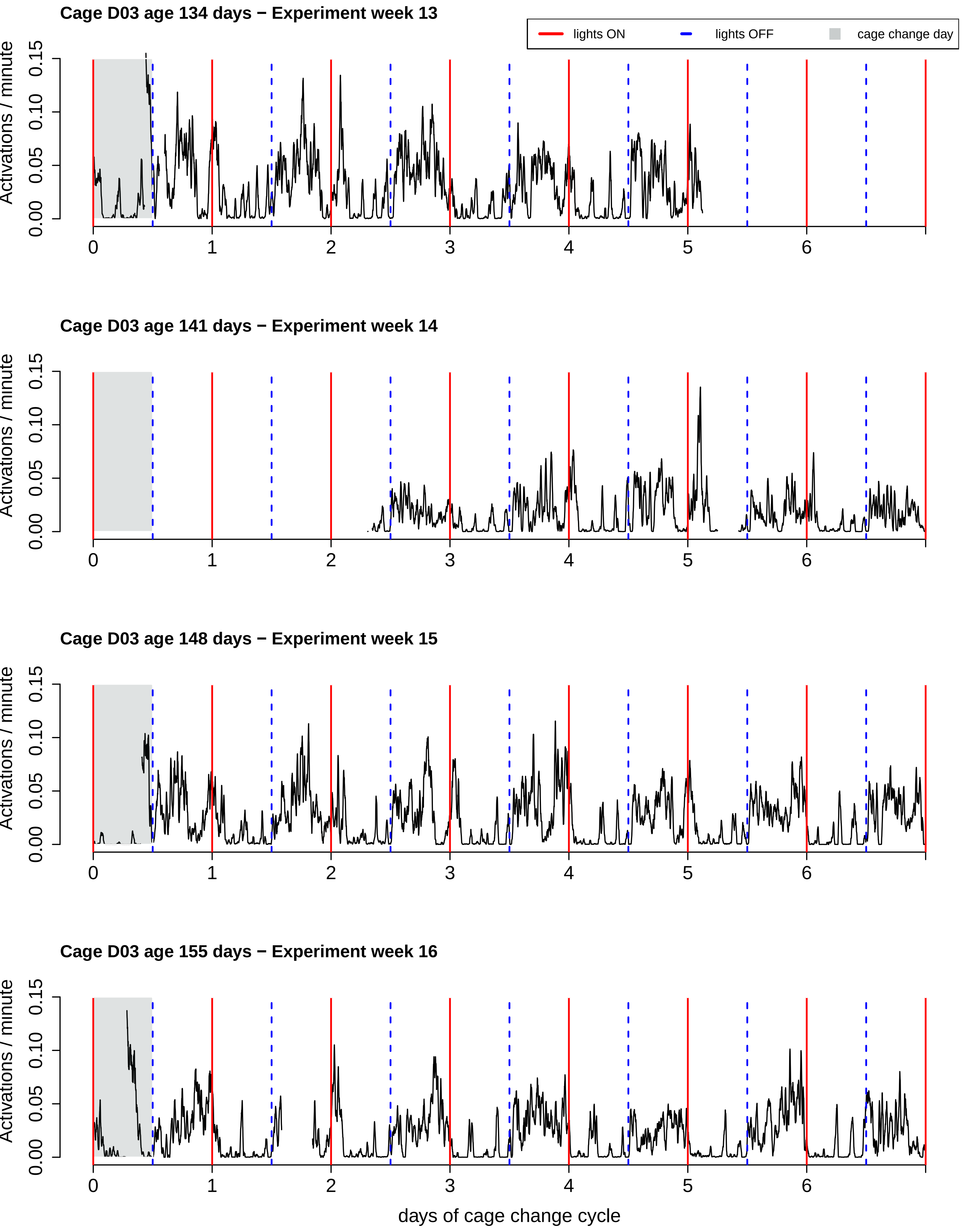
Cage D03 age 134 days − Experiment week 13
lights ON
lights OFF
cage change day
Activations / minute
0.00
0.05
0.10
0.15
0
1
2
3
4
5
6
Cage D03 age 141 days − Experiment week 14
Cage D03 age 148 days − Experiment week 15
Cage D03 age 155 days − Experiment week 16
days of cage change cycle

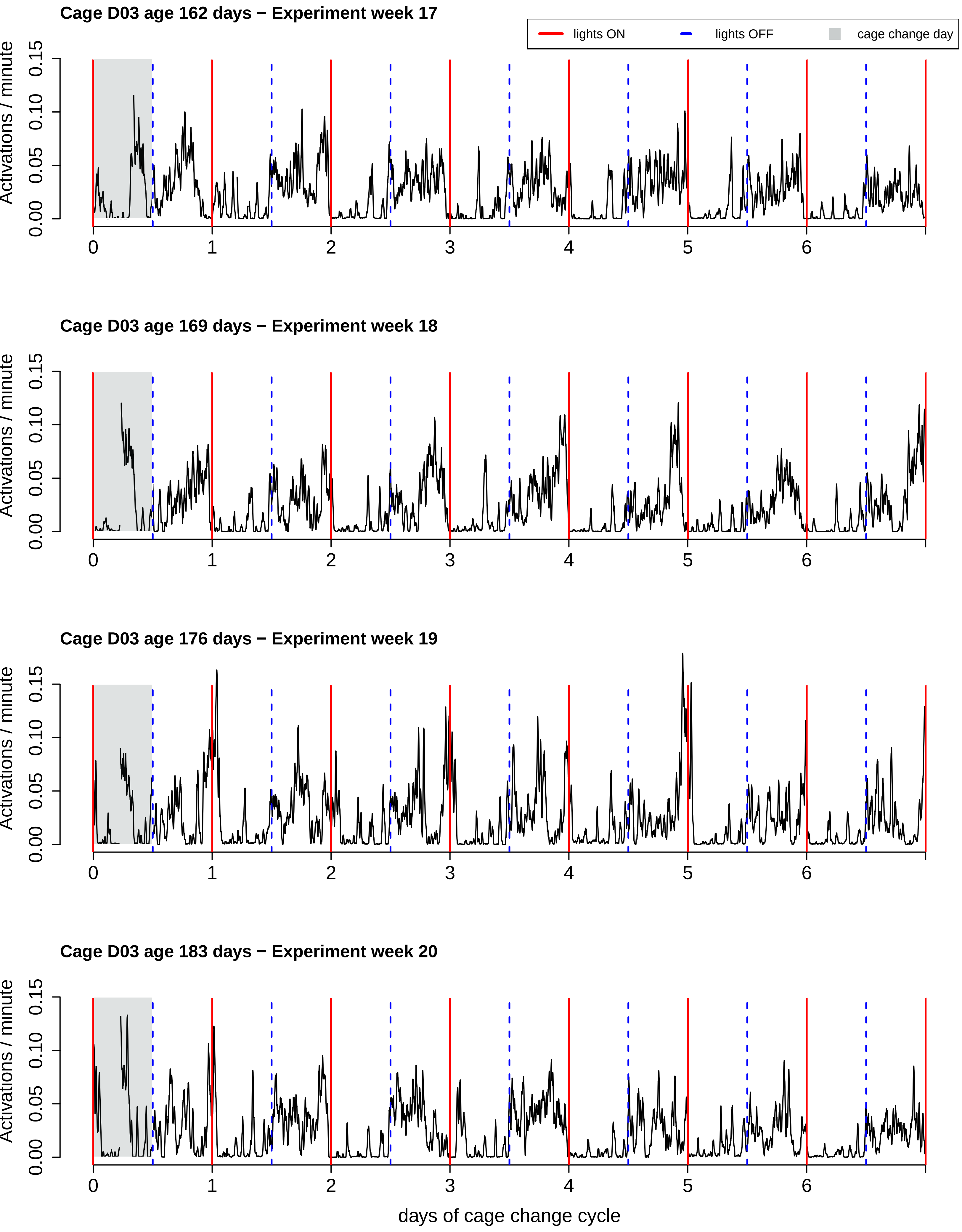
Cage D03 age 162 days − Experiment week 17
lights ON
lights OFF
cage change day
Activations / minute
0.00
0.05
0.10
0.15
0
1
2
3
4
5
6
Cage D03 age 169 days − Experiment week 18
Activations / minute
0.00
0.05
0.10
0.15
0
1
2
3
4
5
6
Cage D03 age 176 days − Experiment week 19
Activations / minute
0.00
0.05
0.10
0.15
0
1
2
3
4
5
6
Cage D03 age 183 days − Experiment week 20
Activations / minute
0.00
0.05
0.10
0.15
0
1
2
3
4
5
6
days of cage change cycle

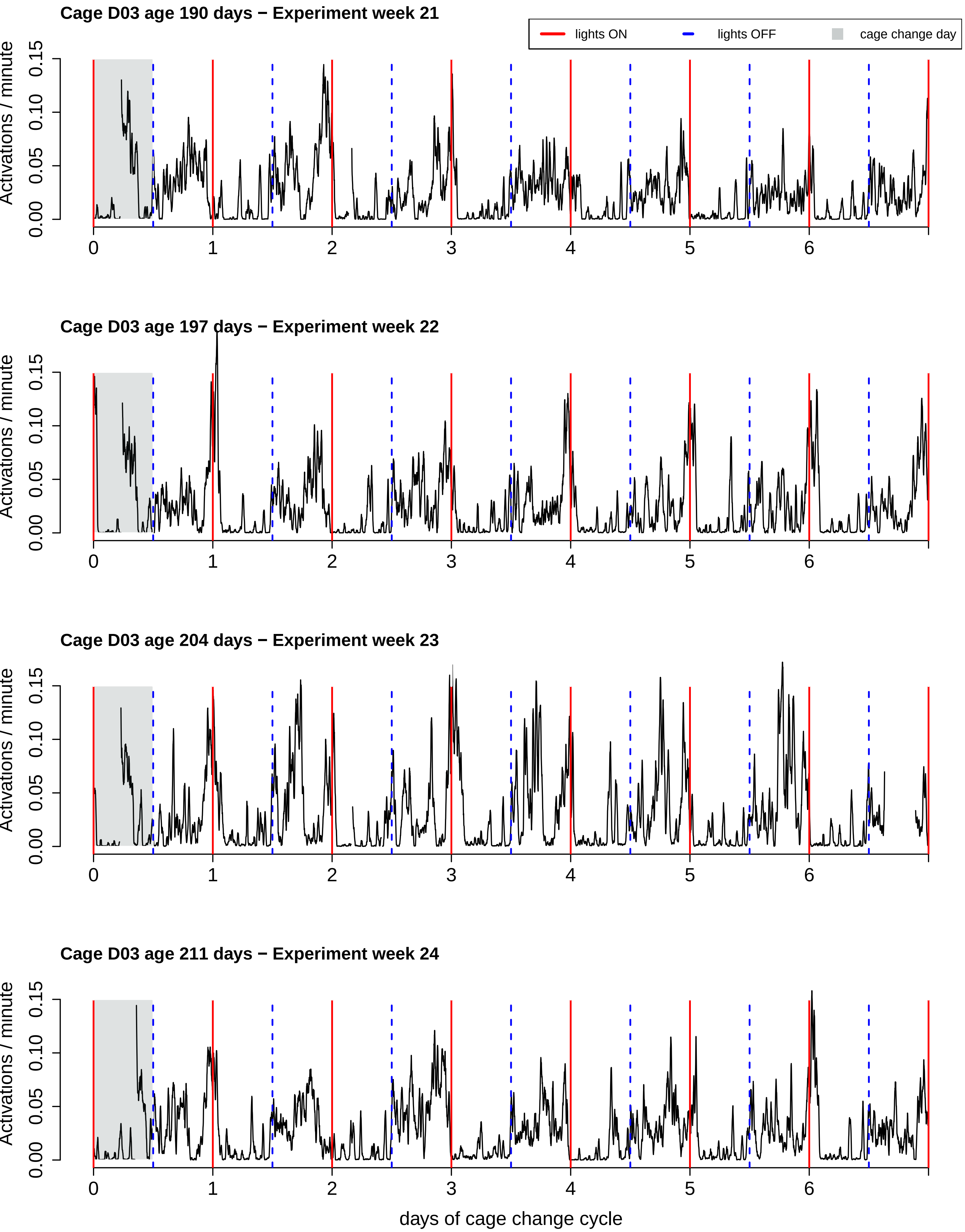
Cage D03 age 190 days − Experiment week 21
lights ON
lights OFF
cage change day
Activations / minute
0.00
0.05
0.10
0.15
0
1
2
3
4
5
6
Cage D03 age 197 days − Experiment week 22
Cage D03 age 204 days − Experiment week 23
Cage D03 age 211 days − Experiment week 24
days of cage change cycle

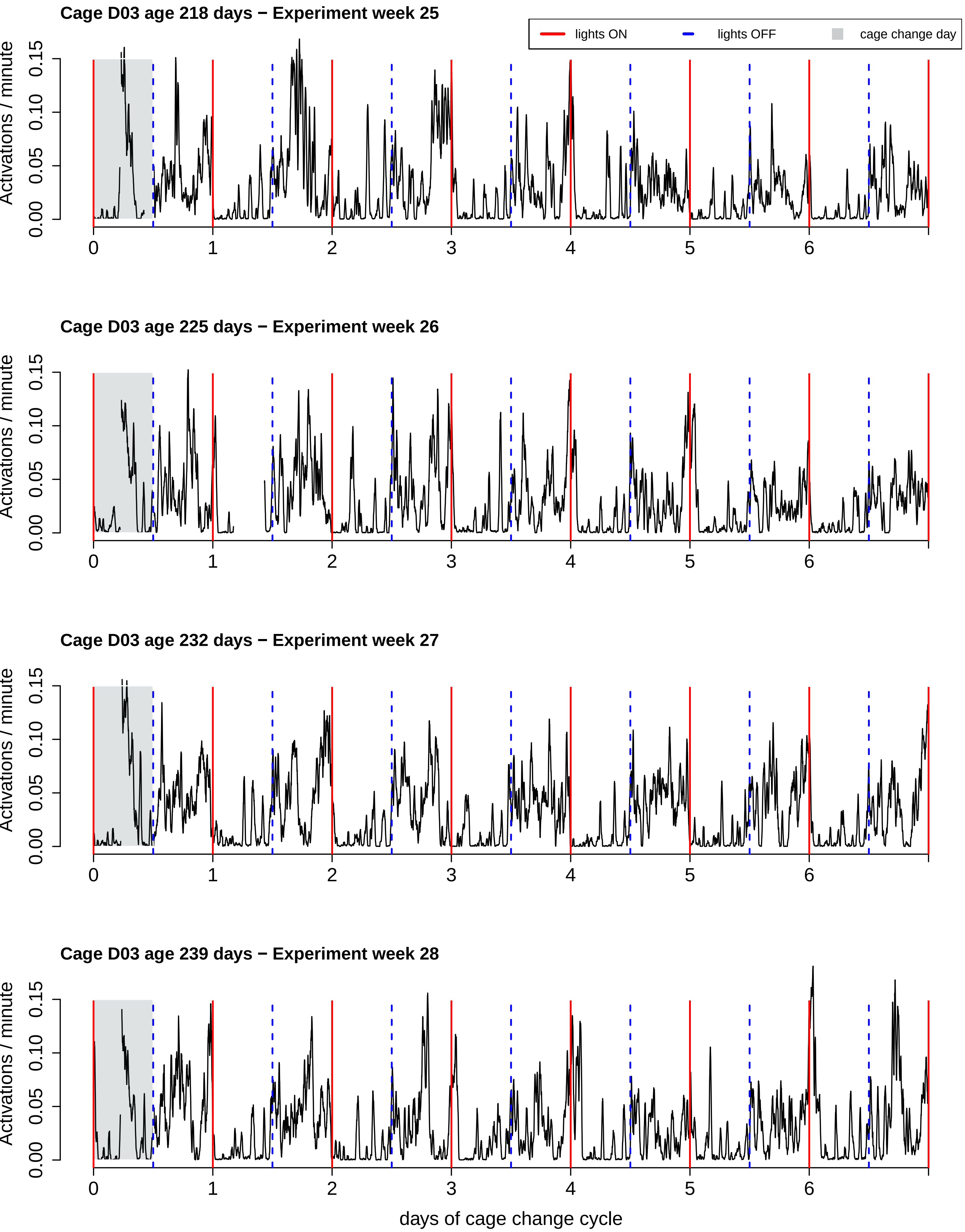

Cage D03 age 218 days − Experiment week 25
lights ON
lights OFF
cage change day
Activations / minute
Cage D03 age 225 days − Experiment week 26
Cage D03 age 232 days − Experiment week 27
Cage D03 age 239 days − Experiment week 28
days of cage change cycle

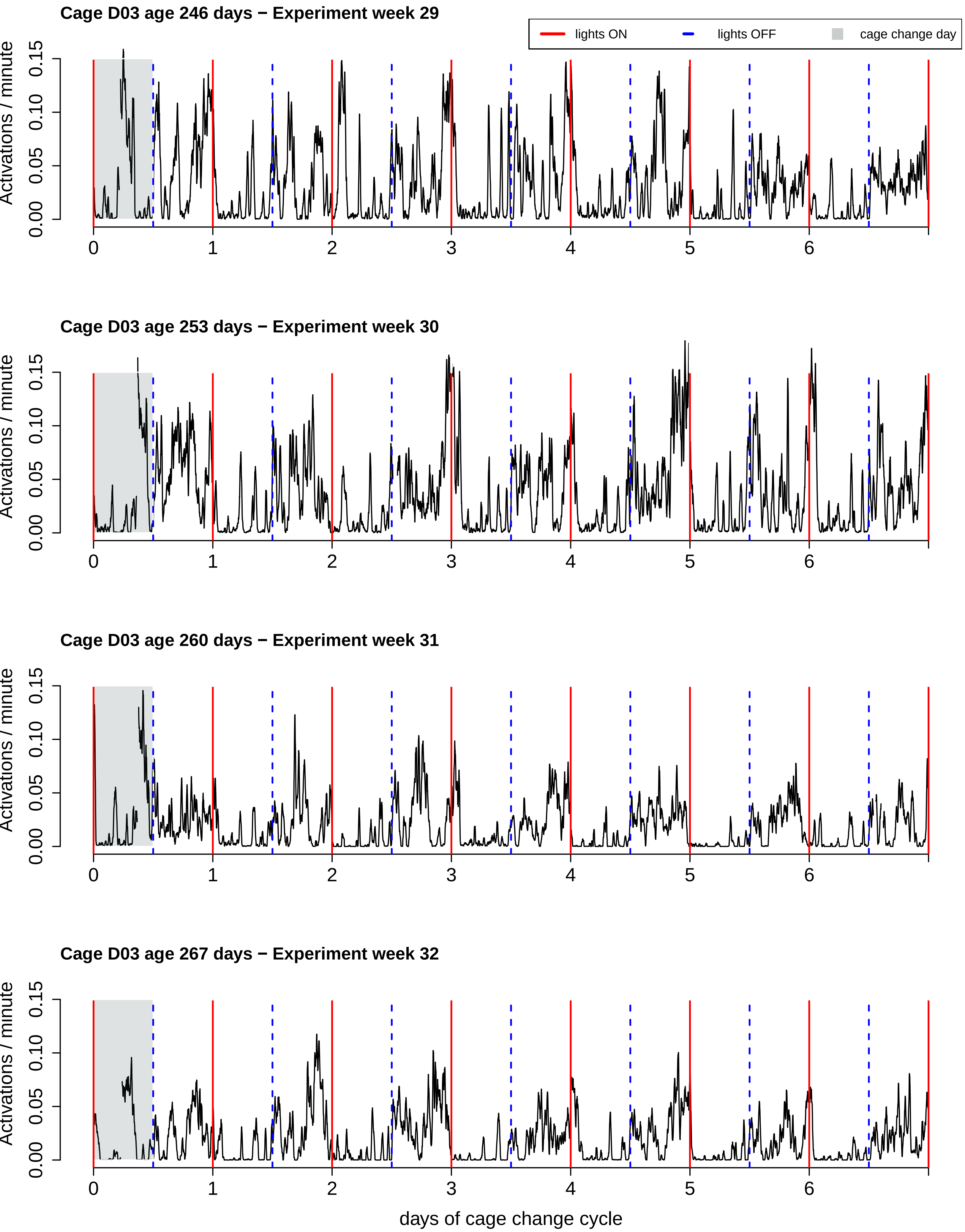

Cage D03 age 246 days − Experiment week 29
lights ON
lights OFF
cage change day
Activations / minute
0.00
0.05
0.10
0.15
0
1
2
3
4
5
6
Cage D03 age 253 days − Experiment week 30
Activations / minute
Cage D03 age 260 days − Experiment week 31
Activations / minute
Cage D03 age 267 days − Experiment week 32
Activations / minute
days of cage change cycle

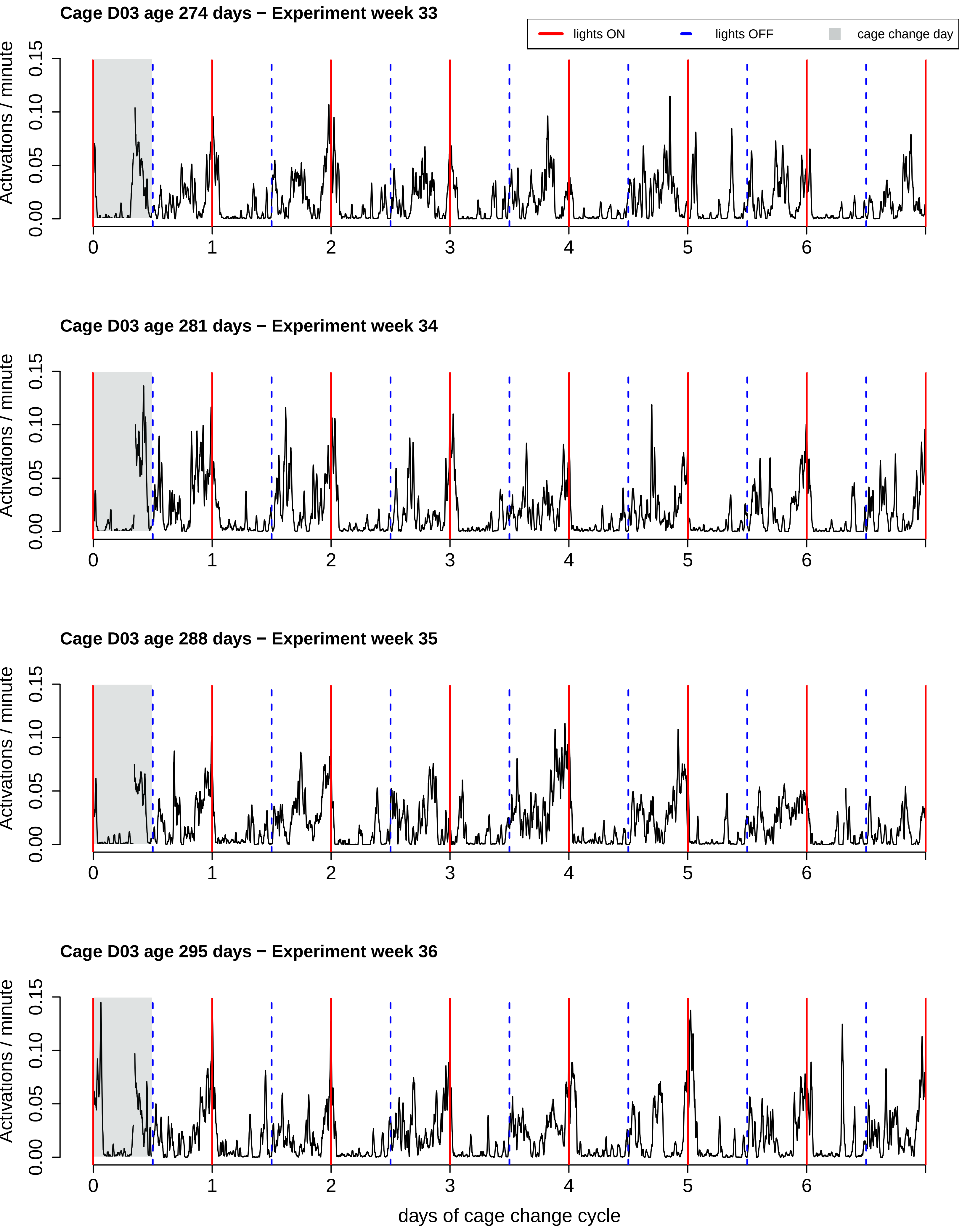
Cage D03 age 274 days − Experiment week 33
lights ON
lights OFF
cage change day
Activations / minute
Cage D03 age 281 days − Experiment week 34
Activations / minute
Cage D03 age 288 days − Experiment week 35
Activations / minute
Cage D03 age 295 days − Experiment week 36
Activations / minute
0.00
0.05
0.10
0.15
0
1
2
3
4
5
6
days of cage change cycle

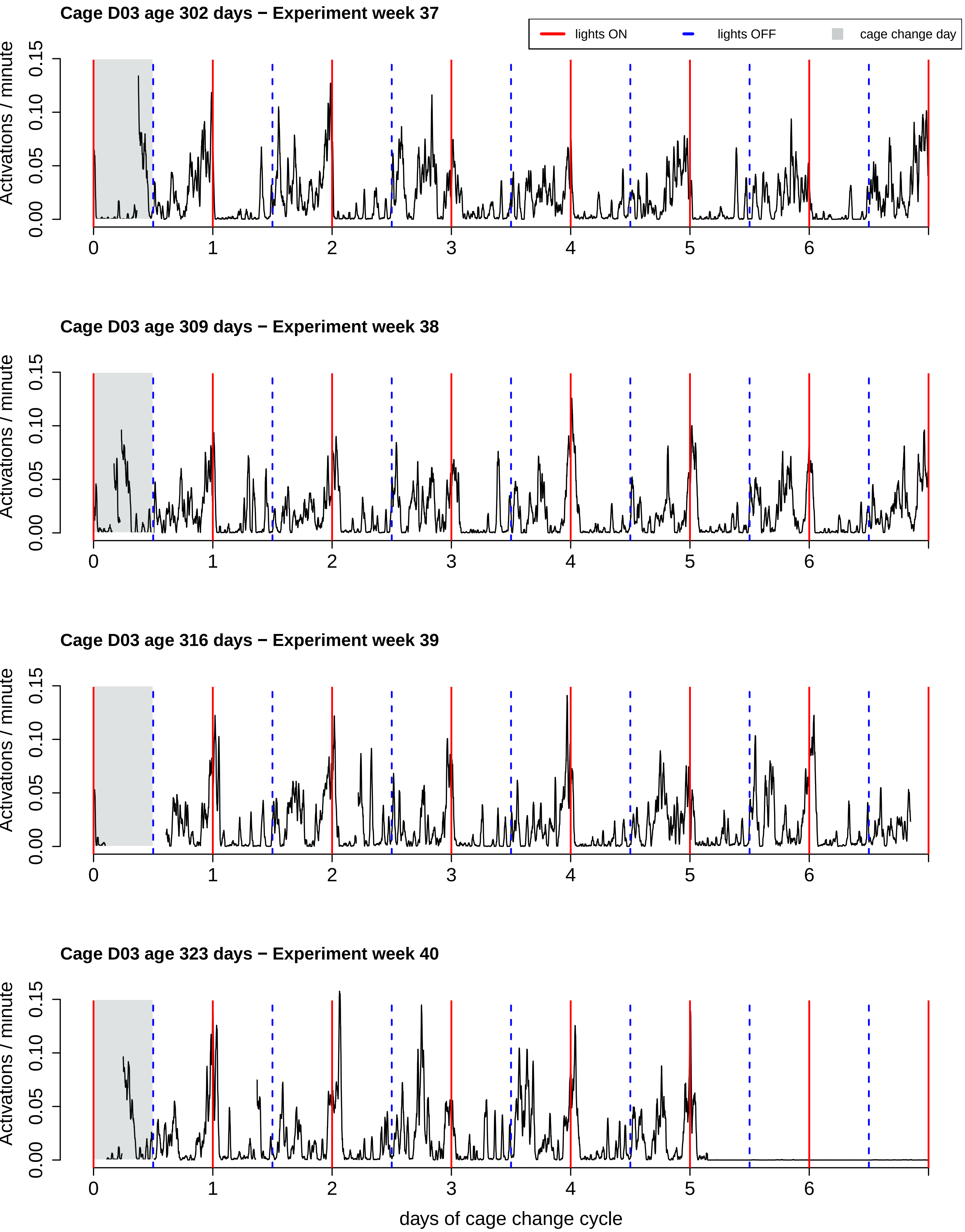
Cage D03 age 302 days − Experiment week 37
lights ON
lights OFF
cage change day
Activations / minute
0.00
0.05
0.10
0.15
0
1
2
3
4
5
6
Cage D03 age 309 days − Experiment week 38
Cage D03 age 316 days − Experiment week 39
Cage D03 age 323 days − Experiment week 40
days of cage change cycle

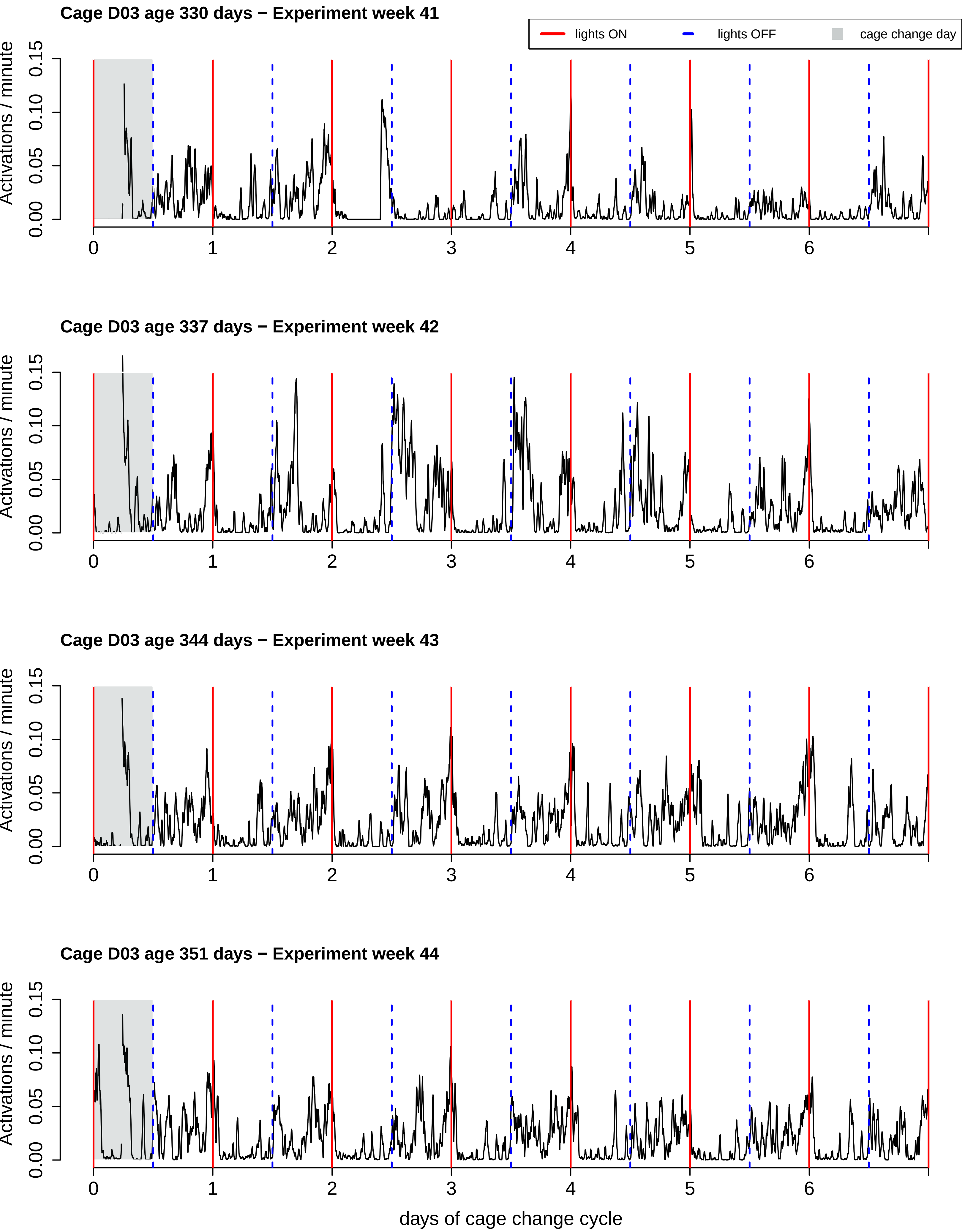

Cage D03 age 330 days − Experiment week 41
lights ON
lights OFF
cage change day
Activations / minute
0.00
0.05
0.10
0.15
0
1
2
3
4
5
6
Cage D03 age 337 days − Experiment week 42
Activations / minute
0.00
0.05
0.10
0.15
0
1
2
3
4
5
6
Cage D03 age 344 days − Experiment week 43
Activations / minute
0.00
0.05
0.10
0.15
0
1
2
3
4
5
6
Cage D03 age 351 days − Experiment week 44
Activations / minute
0.00
0.05
0.10
0.15
0
1
2
3
4
5
6
days of cage change cycle

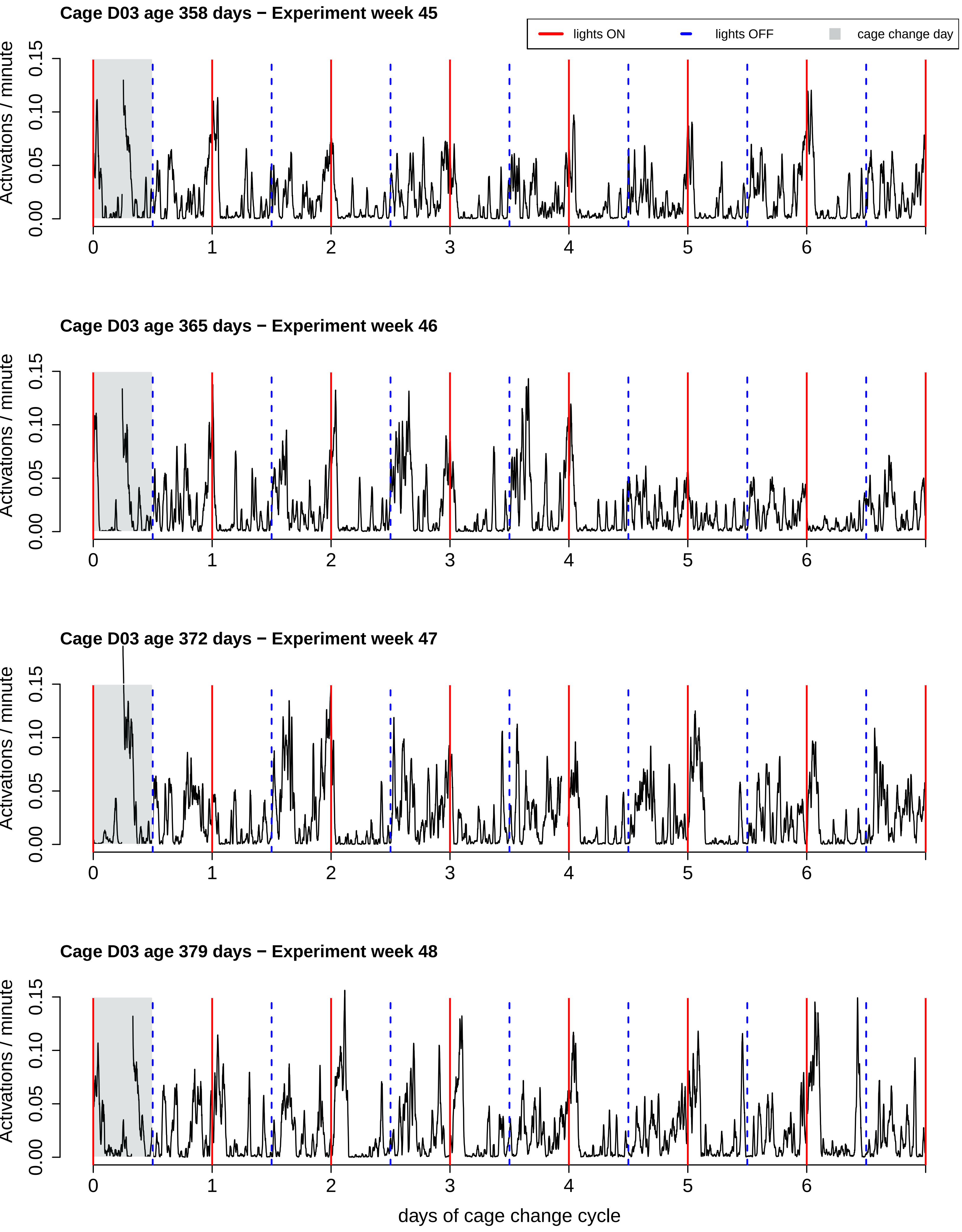
Cage D03 age 358 days − Experiment week 45
lights ON
lights OFF
cage change day
Activations / minute
0.00
0.05
0.10
0.15
0
1
2
3
4
5
6
Cage D03 age 365 days − Experiment week 46
Cage D03 age 372 days − Experiment week 47
Cage D03 age 379 days − Experiment week 48
days of cage change cycle

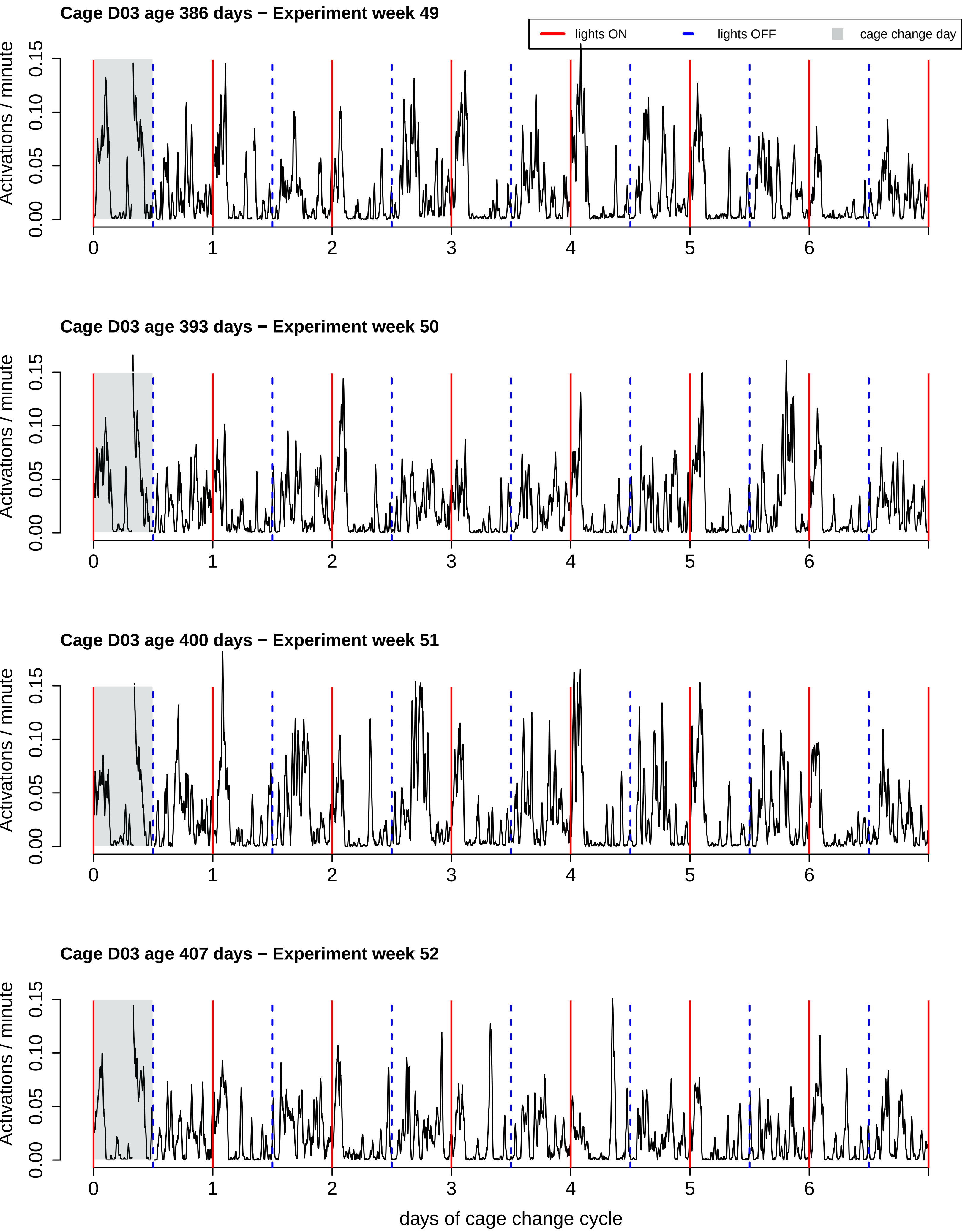

Cage D03 age 386 days − Experiment week 49
lights ON
lights OFF
cage change day
Activations / minute
0.00
0.05
0.10
0.15
0
1
2
3
4
5
6
Cage D03 age 393 days − Experiment week 50
Cage D03 age 400 days − Experiment week 51
Cage D03 age 407 days − Experiment week 52
days of cage change cycle

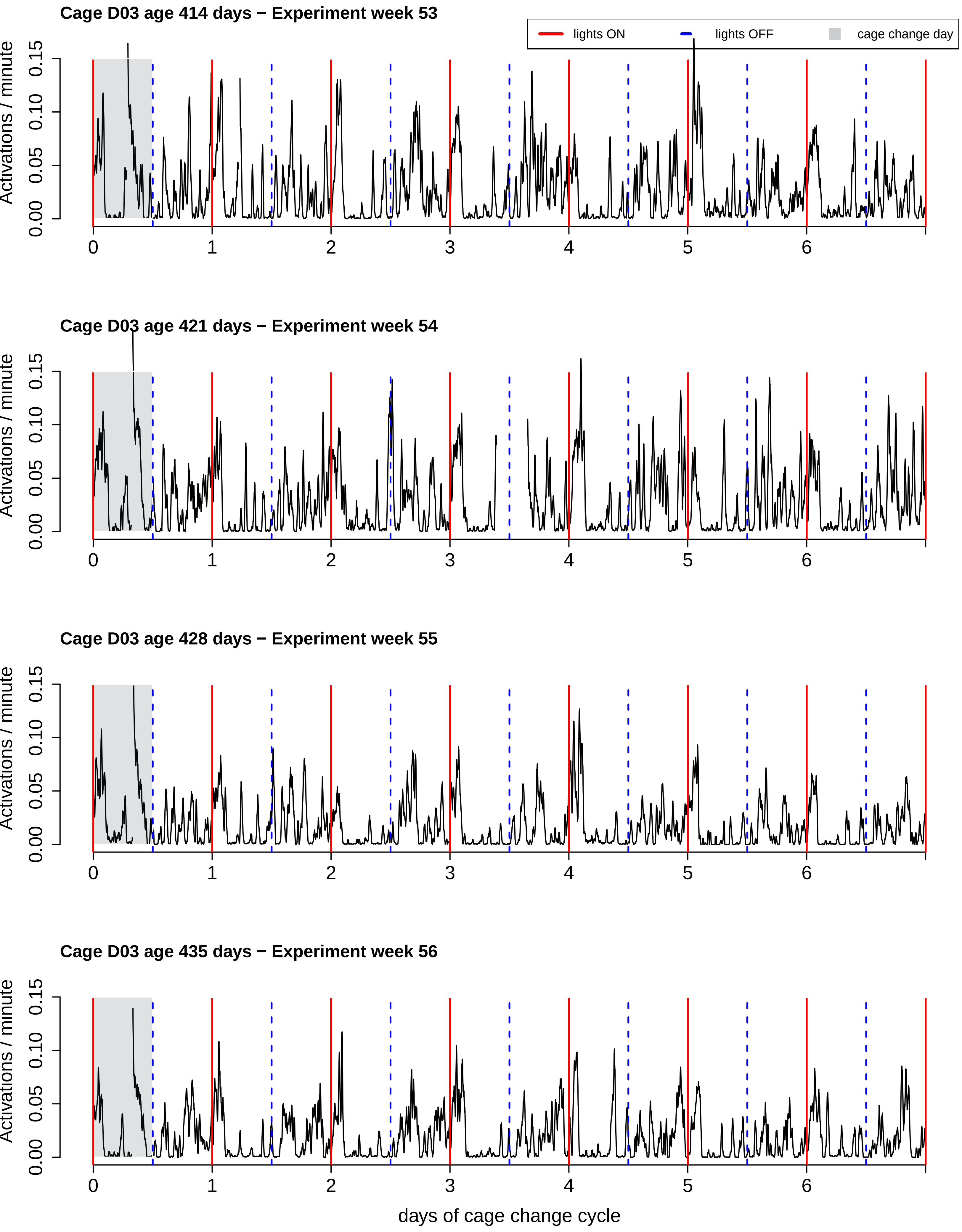
Cage D03 age 414 days − Experiment week 53
lights ON
lights OFF
cage change day
Cage D03 age 421 days − Experiment week 54
Cage D03 age 428 days − Experiment week 55
Cage D03 age 435 days − Experiment week 56
Activations / minute
0.00
0.05
0.10
0.15
0
1
2
3
4
5
6
days of cage change cycle

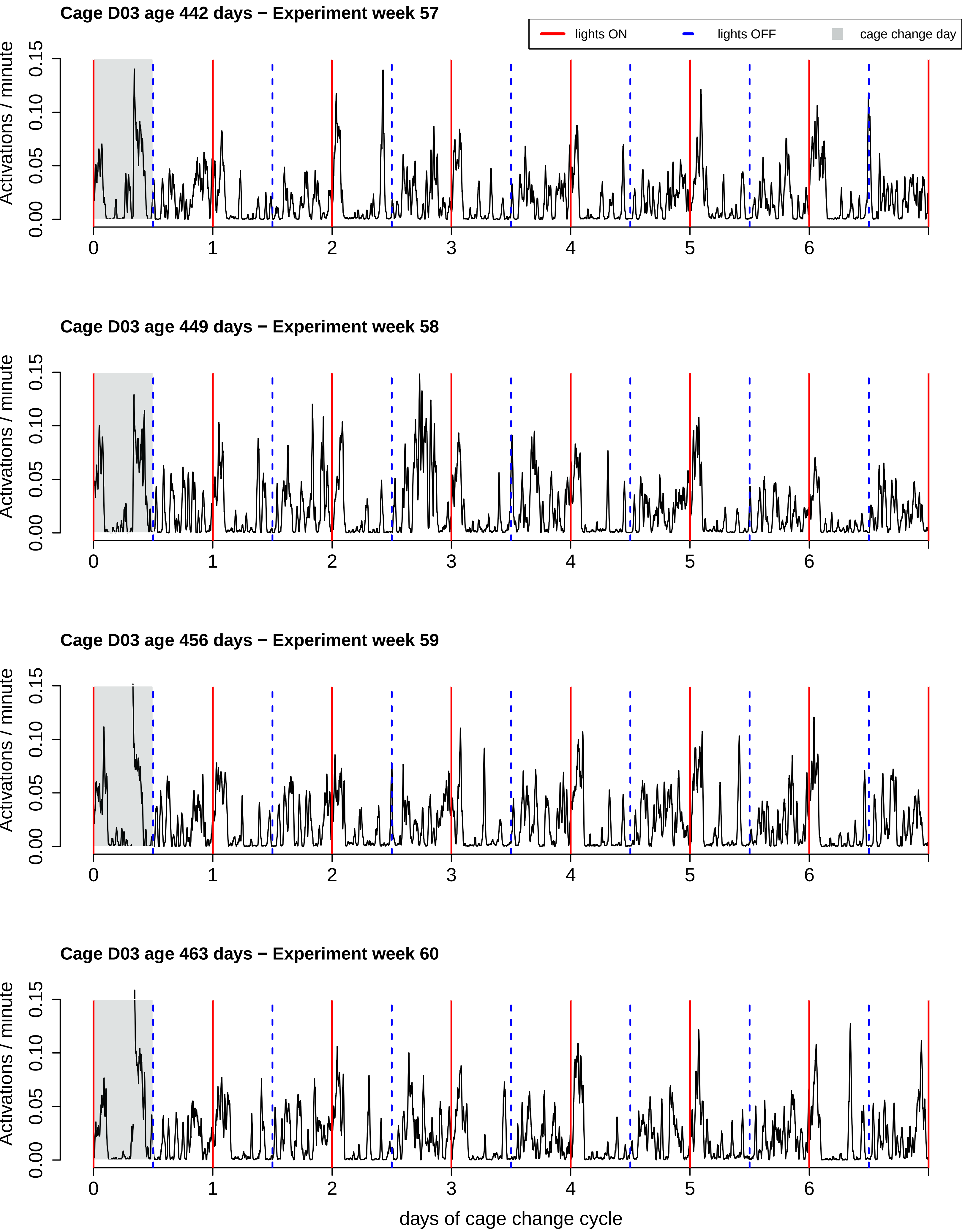
Cage D03 age 442 days − Experiment week 57
lights ON
lights OFF
cage change day
Cage D03 age 449 days − Experiment week 58
Cage D03 age 456 days − Experiment week 59
Cage D03 age 463 days − Experiment week 60
Activations / minute
days of cage change cycle

Cage D03 age 470 days − Experiment week 61

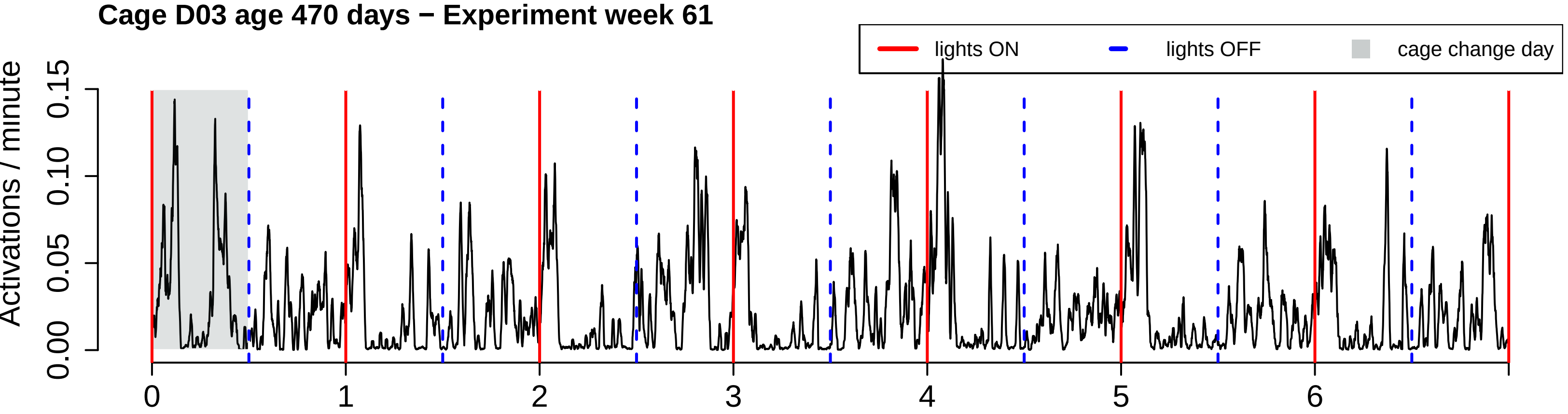

Supplement: Supplementary file 2 — Supplementary Information 2. [file 41598_2021_84141_MOESM2_ESM.pdf]
